# Bis-CF_3_‑bipyridine Ligands for the Iridium-Catalyzed Borylation of *N*‑Methylamides

**DOI:** 10.1021/acscatal.5c00933

**Published:** 2025-04-16

**Authors:** Daniel Marcos-Atanes, Gonzalo Jiménez-Osés, José L. Mascareñas

**Affiliations:** † Centro Singular de Investigación en Química Biolóxica e Materiais Moleculares (CiQUS) and Departamento de Química Orgánica. 16780 Universidade de Santiago de Compostela, Santiago de Compostela 15782, Spain; § Basque Research and Technology Alliance (BRTA), Center for Cooperative Research in Biosciences (CIC bioGUNE), Derio 48160, Spain; ‡ Ikerbasque, Basque Foundation for Science, Bilbao 48013, Spain

**Keywords:** C−H activation, iridium catalysis, borylation, methylamides, bipyridine

## Abstract

Bipyridine and phenanthroline are well-established neutral ligands for promoting iridium-catalyzed borylations of aromatic C–H bonds. However, their use with aliphatic substrates is almost uncharted. Herein we demonstrate that introducing CF substituents at the 5- and 5′-positions of bipyridine generates ligands that enable an efficient and regioselective iridium-catalyzed borylation of the methyl group in a broad variety of methylamides. The reaction shows broad functional group tolerance and exhibits remarkable selectivity, offering a powerful approach for the borylation of challenging aliphatic C–H bonds. Mechanistic investigations, including computational analysis, suggest that the accelerating effect of the ligand is likely associated with the formation of non-covalent dispersion interactions between the carbonyl amide of the substrates and the trifluoromethylated pyridine rings of the ligand.

## Graphical abstract

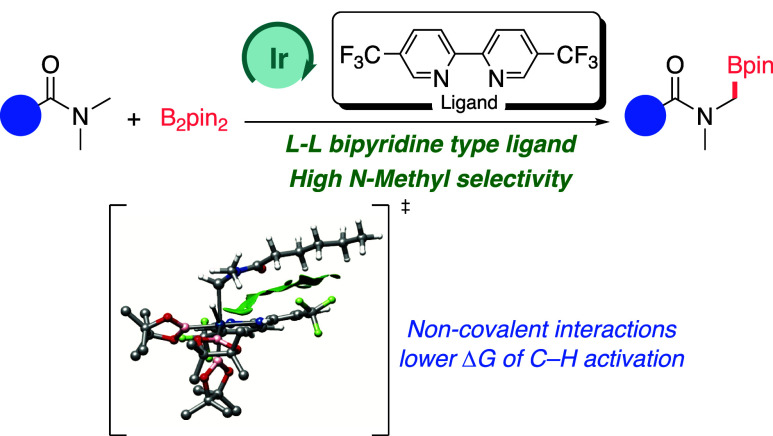

## Introduction

The functionalization of C–H bonds using transition metal-catalysis has emerged as one of the most powerful tools in the field of organic synthesis.^1^ Among the different functionalization reactions so far developed, C–H borylations are especially attractive, due to the well-established potential of group 9 metal complexes, particularly iridium, to catalyze this type of transformations, and because of the synthetic versatility of the resulting borylated products.^2^ A major, enduring challenge in these reactions is controlling their regioselectivity, given the ubiquity of C–H bonds in organic substrates.^3^ In this context, recent years have witnessed impressive advances in the development of methods for the chemo- and regioselective borylation of aromatic C–H bonds.^4^ Initial contributions to this topic relied on the use of bipyridine or phenanthroline iridium ligands, which led to regioselectivities mainly controlled by sterics.^5^ Over the years, many other ligands allowing different types of reactivity and regioselectivity have been designed.^6^ Our own group has recently discovered that introducing CF_3_ substituents at the 5-position of a 2,2’-bipyridine (bipy) ligand induces a complete change in regioselectivity in the borylation of aromatic amides, from *meta/para* to *ortho*, yielding monoborylated products with excellent yields and selectivities.^7^

Another significant challenge in the field of iridium-catalyzed C–H borylations is the functionalization of less reactive alkyl C­(*sp* ^3^)–H bonds. Generally, these reactions require high temperatures and the use of superstoichiometric amounts of the substrates,^8^ such as in the report of Schley and co-workers on the borylation of various hydrocarbons using 2,2′-dipyridylarylmethane as iridium ligand (Figure 1a, **A**).^9^ The Kuninobu group introduced a silyl-phenanthroline pincer ligand **B** for a comparable reaction, again requiring an excess of the substrate.^10^ More recently, Hartwig and co-workers demonstrated that 2-substituted phenanthrolines (**C**) enable the iridium-catalyzed borylation of alkyl C–H bonds at milder temperatures, using the substrates as the limiting reagents.^11^

**Figure 1:**
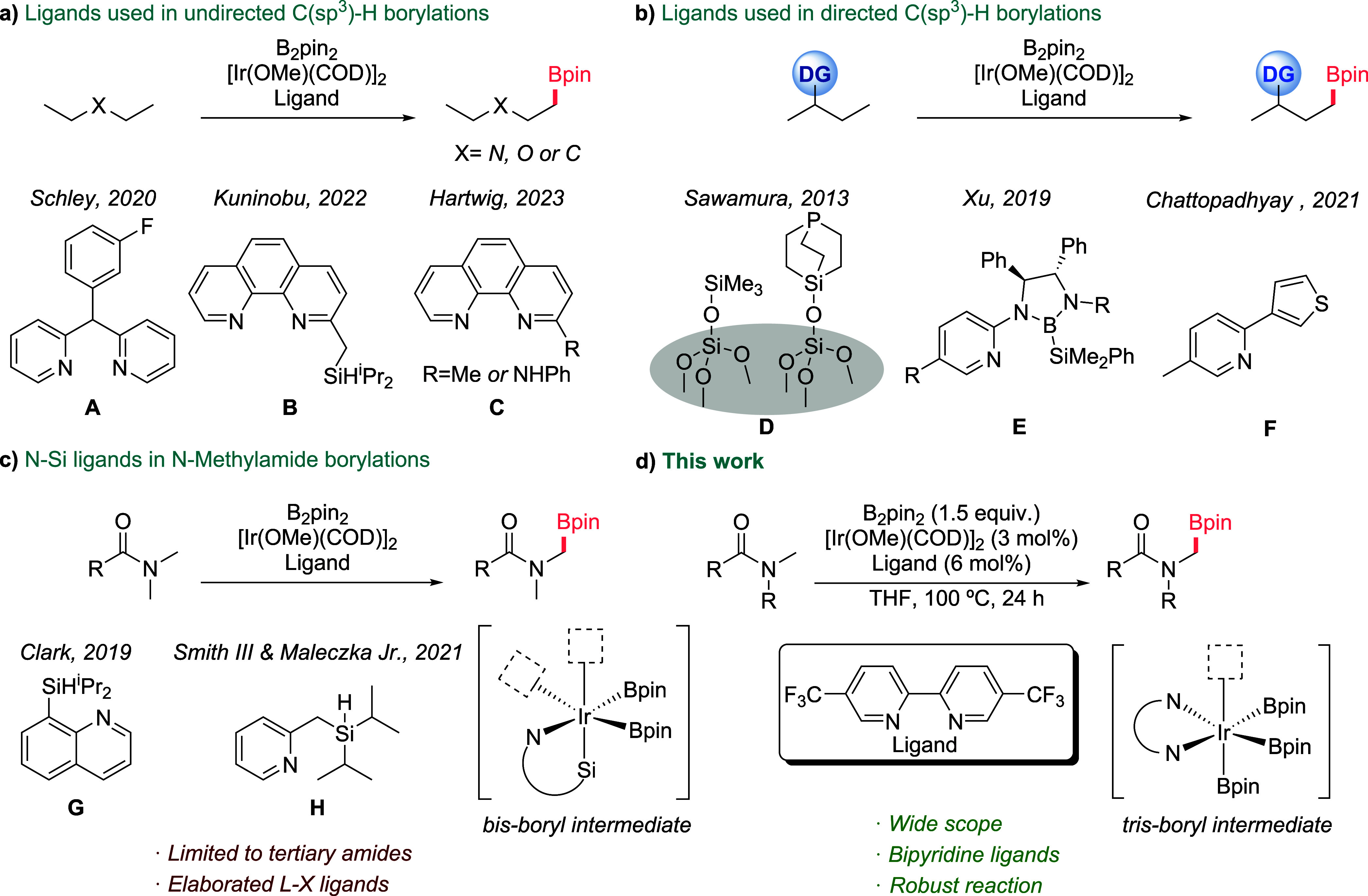
a) Overview of prior studies on Ir-catalyzed undirected borylation of C­(*sp* ^3^)–H bonds; b) C­(*sp* ^3^)–H borylations in substrates with DGs; c) C­(*sp* ^3^)–H borylations of *N*-methylamides; d) This work: borylations with designed L–L ligands.

The presence of directing functional groups (DG) in the precursors can be leveraged for selective C­(*sp* ^3^)–H borylations at relatively mild temperatures. These reactions require the use of monodentate (L), or bidentate L-X type of ligands, which enable the opening of two coordination sites at the iridium center. For example, the Sawamura group has used silica supported phosphines (Si-SMAP or Si-TRIP) to achieve regioselective β borylation of various aliphatic substrates featuring pyridines, imidazoles or oxazoles as internal coordinating moieties (Figure 1b, **D**).^12^ The group of S. Xu demonstrated that chiral bidentate silyl boryl ligands (L-X) facilitate the enantioselective borylation of cyclic and linear alkyl chains at the β or even γ positions relative to the directing groups such as amides, carbamates, pyrazoles, benzoxazoles or benzothiazoles (Figure 1b, **E**).^13,14^ Similarly, the group of Chattopadhyay utilized a pyridine-thienyl ligand (L-X) to achieve efficient borylation of various aliphatic substrates featuring pyridine as the directing group, enabling selective C­(*sp* ^3^)–H bond borylations (Figure 1b, **F**).

Related bidentate monoanionic ligands have also been used for the borylation of methyl groups in *N*,*N*-dimethylamides (Figure 1c).^15^ These reactions are very attractive owing to the pharmacological relevance of α*-*amidoboronic acids, and their potential for further modification.^16^ However, their success is limited to a few tertiary amides, and to substrates lacking other functional groups. The use of bidentate monoanionic ligands (L-X) is key for the reaction, because they generate iridium­(III)-*bis*-boryl intermediates with two vacant sites at the metal center (Figure 1c, **G**, **H**), one for coordinating the carbonyl of the amide and the other for the C–H activation.

Our recent discovery that bidentate neutral bipyridine ligands (L-L) can be used for the *ortho*-borylation of benzamides when CF_3_ substituents are present at the 5-position of the pyridine units,^7^ raised the question of whether such ligands could also be effective for the borylation of alkylamides. Our previous studies suggested that the *ortho*-regiocontrol in aromatic precursors originates from unusual non-covalent dispersion interactions between the benzamide group of the substrate and the polarized ring(s) of the bipyridine ligand. Therefore, it was intriguing to know whether similar interactions could also be harnessed for promoting the borylation of C­(*sp* ^3^)–H bonds.

Herein, we demonstrate that using 5,5′-bis-CF_3_-bipyridine as ligand enables the iridium-catalyzed selective *N*-methyl borylation of a broad range of *N*-methylamides, with excellent regioselectivity and functional group tolerance. This stands in sharp contrast to the significantly lower reactivity observed with the parent 2,2′-bipyridine or 1,10-phenanthroline ligands.

## Methods

Our studies started by attempting the borylation of *N*,*N*-dimethylhexanamide (**1a**), screening different types of bipyridine ligands. To quickly assess the reactivity, we used GC/MS to analyze starting material (SM) to product ratios. Not surprisingly, heating **1a** at 100 °C in the presence of catalytic amounts of [Ir­(OMe)­COD]_2_, 2,2’-bipyridine (**L1**), and B_2_pin_2_ as the boron source, resulted in poor conversion and very low yield of product **2a**, after 24 h (Table 1, entry 1).

**Table 1:**
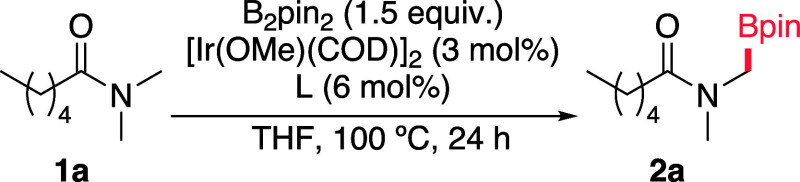
Ligand Screening for the Catalytic Borylation of **1a** ^a^

entry	**L**	ratio **1a**:**2a**
1	**L1**	93:7
2	**L2**	16:84
3	**L3**	95:5
4	**L4**	2:98
5	**L5**	98:2
6	**L6**	99:1
7	**L7**	87:13
8	–	98:2
9	**L8**	100:0
10	**L9**	100:0

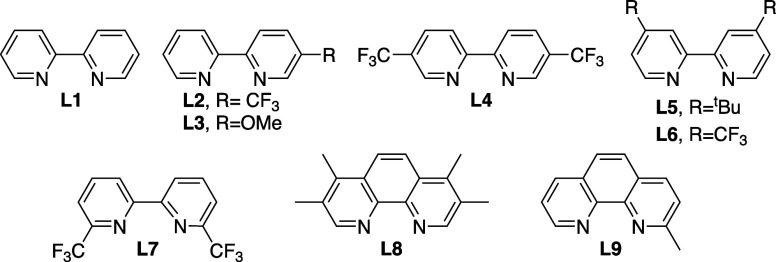

a Reaction conditions: **1a** (0.25 mmol), B_2_pin_2_ (0.375 mmol, 1.5 equiv), [Ir­(OMe)­(COD)]_2_ (3 mol %), **L** (6 mol %), THF (0.2 M), 100 °C, 24 h. Conversion was analyzed by GC-MS.

Remarkably, the introduction of a trifluoromethyl group at the 5′-position of one the pyridines (**L2**) significantly increased the amount of the α-amidomethylboronate ester **2a** (16:84 ratio between SM/product), in a rather clean reaction. In contrast, the use of a ligand with a methoxy instead of the CF_3_ group at the same position (**L3**) showed almost no conversion. The symmetrical CF_3_-disubstituted ligand **L4** exhibited an excellent reactivity, leading to a 96% isolated yield of product **2a**. Curiously, when using isomeric ligands **L6** or **L7**, which are akin to **L4** but with the CF_3_ groups at positions 4 or 6 of the pyridines, we observed a very poor reactivity, like that obtained with the parent bipyridine. These results align with our previous observations on the selective *ortho*-borylation of aromatic amides, and strongly suggest that the enhanced reactivity might be mainly driven by specific dispersive interactions between the CF_3_-pyridine moiety and the amide carbonyl. On the other hand, under the same conditions, but in the absence of any ligands, we observed no conversion (Table 1, entry 8).

Interestingly, phenanthroline ligands, such as **L8** and **L9**, which had been successfully used for the borylation of hydrocarbons,^11^ led to no conversion. Instead, we detected byproducts resulting from the borylation of THF. A solvent screening with the top-performing ligand (**L4**) revealed that ethereal solvents, especially THF, were the most effective for achieving higher conversions. Coordinating solvents such as NMP or MeCN failed to promote any measurable conversion (Table S1 in the Supporting Information). These solvent effects align with the results observed in our previous studies in the *ortho*-borylation of benzamides.^7^

Therefore, the most effective conditions involve using Ir­(OMe)­(COD)]_2_ (3 mol %), **L4** (6 mol %), 1.5 equiv of B_2_pin_2_, and 1 equiv of the *N*-methylamide in THF (0.2 M), and heating the mixture in a sealed tube at 100 °C. While initial reactions were conducted over 24 h, we later found that under these optimal conditions, full conversion is achieved within 4 h, obtaining a 96% yield of **2a**.

With the optimal conditions in hand, we investigated the scope of the reaction. Gratifyingly, a wide range of *N*-methylamides reacted successfully, leading to the expected monoborylated products (Scheme 1). The reaction proved effective across a variety of aliphatic amides exhibiting different alkyl chain lengths, and therefore products **2a**–**2e** (**2e** being a myristic acid derivative) were formed in good to excellent yields. Related precursors but with branched carbon tethers were also transformed into the expected products, such as **2f**–**2i**. The selective formation of **2f** is particularly significant, as the precursor presents two topologically similar methyl groups, yet only the *N*-Me moiety undergoes borylation; this result confirms the essential role of the amide nitrogen to facilitate the C–H activation step (see Figure S3 in the Supporting Information). We calculated the energy barriers for the C–H activation at the isopropyl methyl group and found the oxidative addition step to be considerably higher than that required for the C–H activation at the *N*-Me position, fully consistent with the experimentally observed regioselectivity (see Figure S11 in the Supporting Information).

**Scheme 1:**
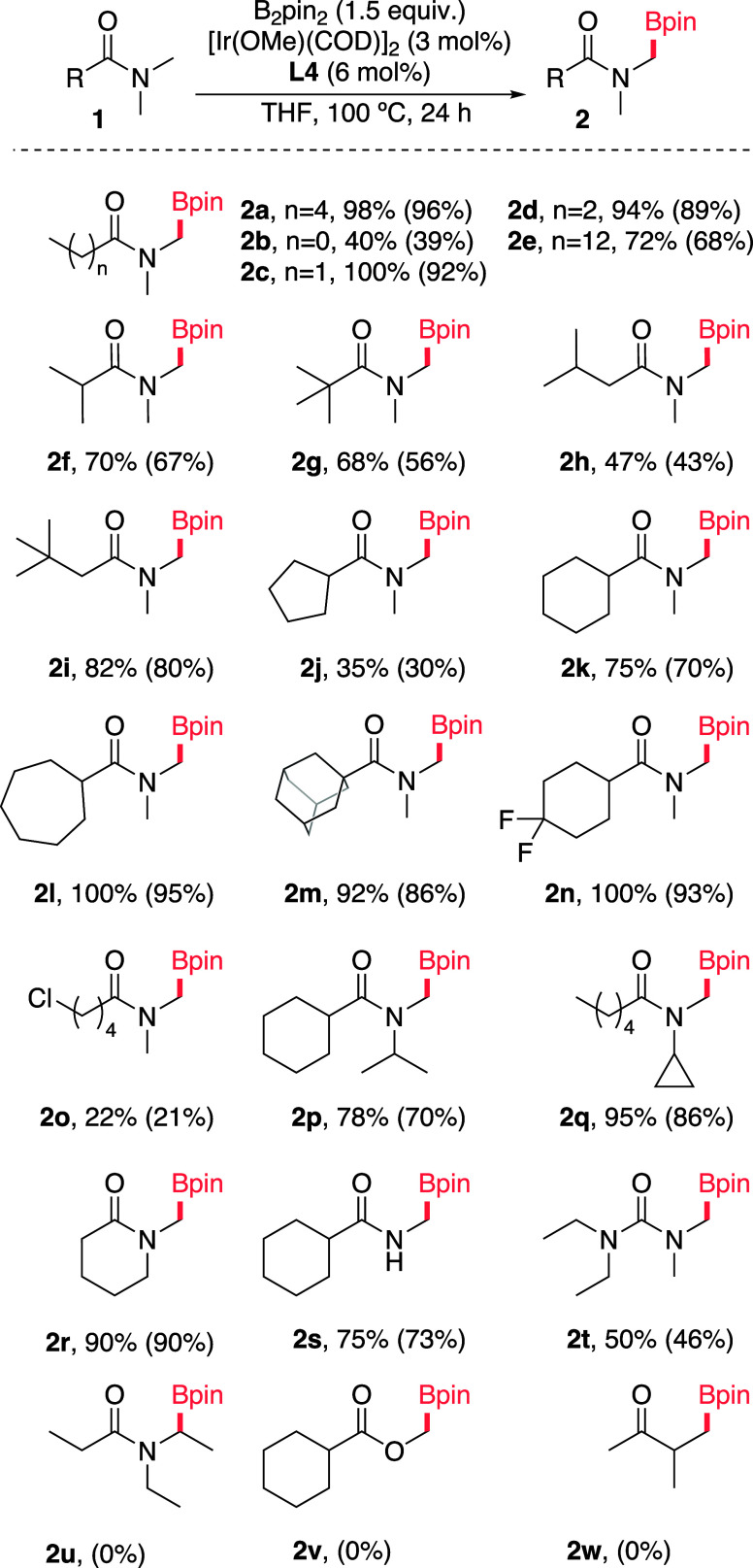
Scope in the Borylation of a Variety of *N*-Methylamides Using Ligand L4^a^

a Reaction conditions: substrate (0.25 mmol), B_2_pin_2_ (0.375 mmol, 1.5 equiv), [Ir­(OMe)­(COD)]_2_ (3 mol %), **L4** (6 mol %), THF (0.2 M), 100 °C, 24 h. ^1^H NMR yield using CH_2_Br_2_ as IS, isolated yields reported in parentheses.

The reaction smoothly accommodated cyclic alkyl amides of various ring sizes, from 5- to 7-membered rings, including adamantane (**2j**-**2m**). Notably, the borylation process tolerates the presence of halogens in the substrates, as evidenced by the successful synthesis of **2n** and **2o**, without any significant side-reaction. In amides featuring a methyl and a different alkyl substituent at the nitrogen, the catalyst exhibited complete regioselectivity toward the *N*-methyl position, as demonstrated by the formation of isopropyl and cyclopropyl derivatives **2p** and **2q**. Importantly, cyclic methylamides were also excellent substrates for the reaction, and products like lactam **2r** could be obtained in excellent yields. The robustness of the reaction was further demonstrated by its successful extension to secondary amides, with cyclohexylamide **2s** being obtained in a quite good yield. Similarly, the urea derivative **2t** was also produced in satisfactory yields, with no other borylated side-products.

Considering the exquisite selectivity of the above reactions, it was not a surprise that *N*,*N*-diethylamides failed to react to give products like **2u**. This lack of reactivity is aligned with the higher BDE of the C–H bond in *N*-ethyl vs *N*-methyl groups, together with presumable steric hindrance (Figure S3 and Figure S10). Similar outcomes were observed with substrates equipped with carbonyl-containing moieties other than amides; therefore, ester and ketone products **2v** and **2w** were not formed.

At this point we were curious to find out whether the excellent pairing between the ligand **L4** and the C–H borylation at the methyl group could also prevail in substrates bearing aromatic rings, which are intrinsically more reactive. Gratifyingly, this was the case, as demonstrated with *N*-phenylmethyl and *N*-benzylmethyl amide precursors that underwent successful borylations to exclusively yield the desired products **2x** and **2y** (Scheme 2).

**Scheme 2:**
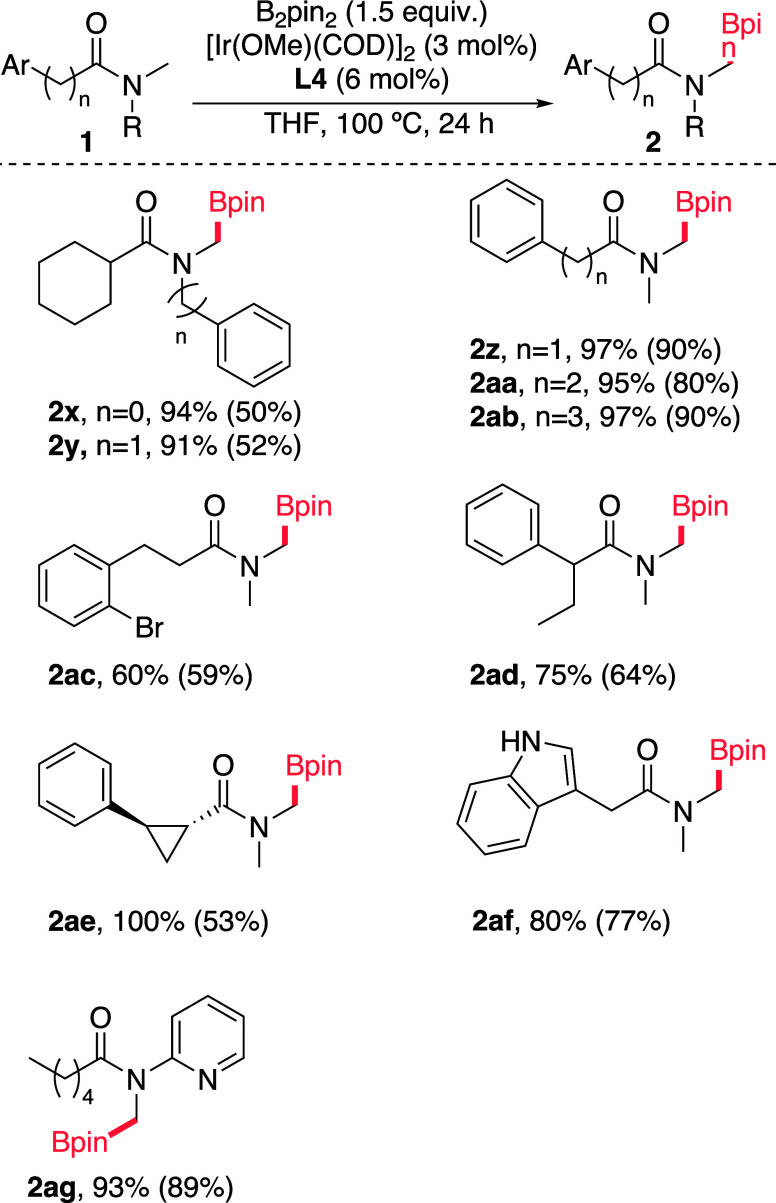
Borylation of *N*-Methylamides Containing Aromatic Moieties^a^

a Reaction conditions: substrate (0.25 mmol), B_2_pin_2_ (0.375 mmol, 1.5 equiv), [Ir­(OMe)­(COD)]_2_ (3 mol %), **L4** (6 mol %), THF (0.2 M), 100 °C, 24 h. ^1^H NMR yield using CH_2_Br_2_ as IS, isolated yields reported in parentheses.

Aromatic rings could be incorporated into the substrate’s alkyl chains and even be equipped with halogen substituents, and the reaction is still very efficient and selective to give the expected products **2z** and **2aa**-**2ad** in excellent yields. Chemoselectivity extends to substrates bearing strained cycles, such as cyclopropanes, as demonstrated by the successful synthesis of product **2ae** (Scheme 2). Furthermore, substrates featuring heteroarene moieties underwent selective borylation at the *N*-Me position, furnishing products **2af–2ag** (Scheme 2).

The significance of these results becomes clearer when considering that using bipyridine (**L1**) as a ligand, instead of **L4**, the reaction gives mixtures of products, with borylation occurring at the aromatic rings.

These results prompted well for the use of the methodology for a late-stage modifications of methylamides in more complex and biorelevant substrates. As shown in Scheme 3 several monoborylated derivatives of established bioactive compounds were readily made in good yields, providing products such as **2ah-2ak** that may be further modified by taking advantage the boronate handle. These results further highlight the selectivity and versatility of the reaction and underscores its significant potential for a broad application in medicinal chemistry.

**Scheme 3:**
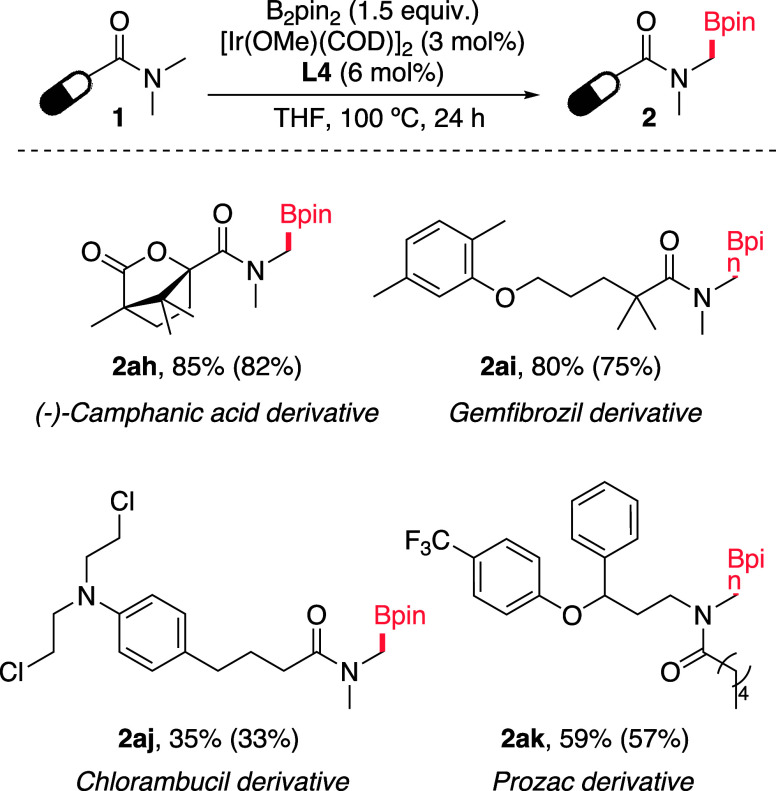
Borylation of More Complex, Biorelevant Substrates^a^

a Reaction conditions: substrate (0.25 mmol), B_2_pin_2_ (0.375 mmol, 1.5 equiv), [Ir­(OMe)­(COD)]_2_ (3 mol %), **L4** (6 mol %), THF (0.2 M), 100 °C, 24 h. ^1^H NMR yield using CH_2_Br_2_ as IS, isolated yield reported in parentheses.

As in the case of our previous results in the *ortho*-borylation of benzamides, we were intrigued by the fact that simply introducing CF_3_ groups in bipyridine, which is an L-L-type of ligand, can effectively drive the borylation reaction. Our previous computational studies discarded a mechanism based on an hemilabile behavior of the ligand,^7^ but we had not specifically evaluated the possibility that **L4** undergoes a rollover C–H activation to form a monoanionic L-X-type ligand. Therefore, we assessed whether these options could explain the different behavior of **L4** over the parent **L1**. However, NMR studies on mixtures of [Ir­(COD)­Cl]_2_ with ligand **L4**, and B_2_pin_2_, at 55 °C for 24 h, ruled out a rollover cyclometalation processes (Figure S3 in the Supporting Information).

A computational analysis was also consistent with these observations, as the activation energy associated with this putative C–H activation was high, and the barriers for the decoordination of the pyridine ring were comparable for ligands **L1** and **L4**. All these results, detailed in the Supporting Information (Figures S8–S9), further suggest that the impressive reactivity promoted by ligand **L4** likely arises from non-covalent, outer-sphere interactions involving the amide group of the substrates.

Therefore, a thorough computational study on the whole reaction pathway for the borylation of *N*,*N*-dimethylhexanamide was conducted at the SMD_THF_/M06/6–311G­(d,p);SDD­(Ir)]//M06/6–31G­(d);LANL2DZ­(Ir) level of theory, with special emphasis on the performance of unsubstituted (**L1**, bipy) vs mono- (**L2**) and *bis*-trifluoromethylated (**L4**) ligands. The minimum energy pathway (MEP) computed for the reaction with ligand **L4** is illustrated in Figure 2. According to these calculations, the C–H activation step occurring through oxidative addition is anticipated to have a ΔG^‡^ of ca. 28 kcal mol^–1^ (via IrB3_TS-OA) leading to the endergonic formation of intermediate IrB3_IC. The relatively high calculated activation barriers match the experimental need for prolonged heating at high temperatures. We initially explored the feasibility of a direct reductive elimination from this intermediate. However, this pathway was calculated to have a large activation barrier of around 41 kcal mol^–1^ (IrB3_TS-RE).

**Figure 2:**
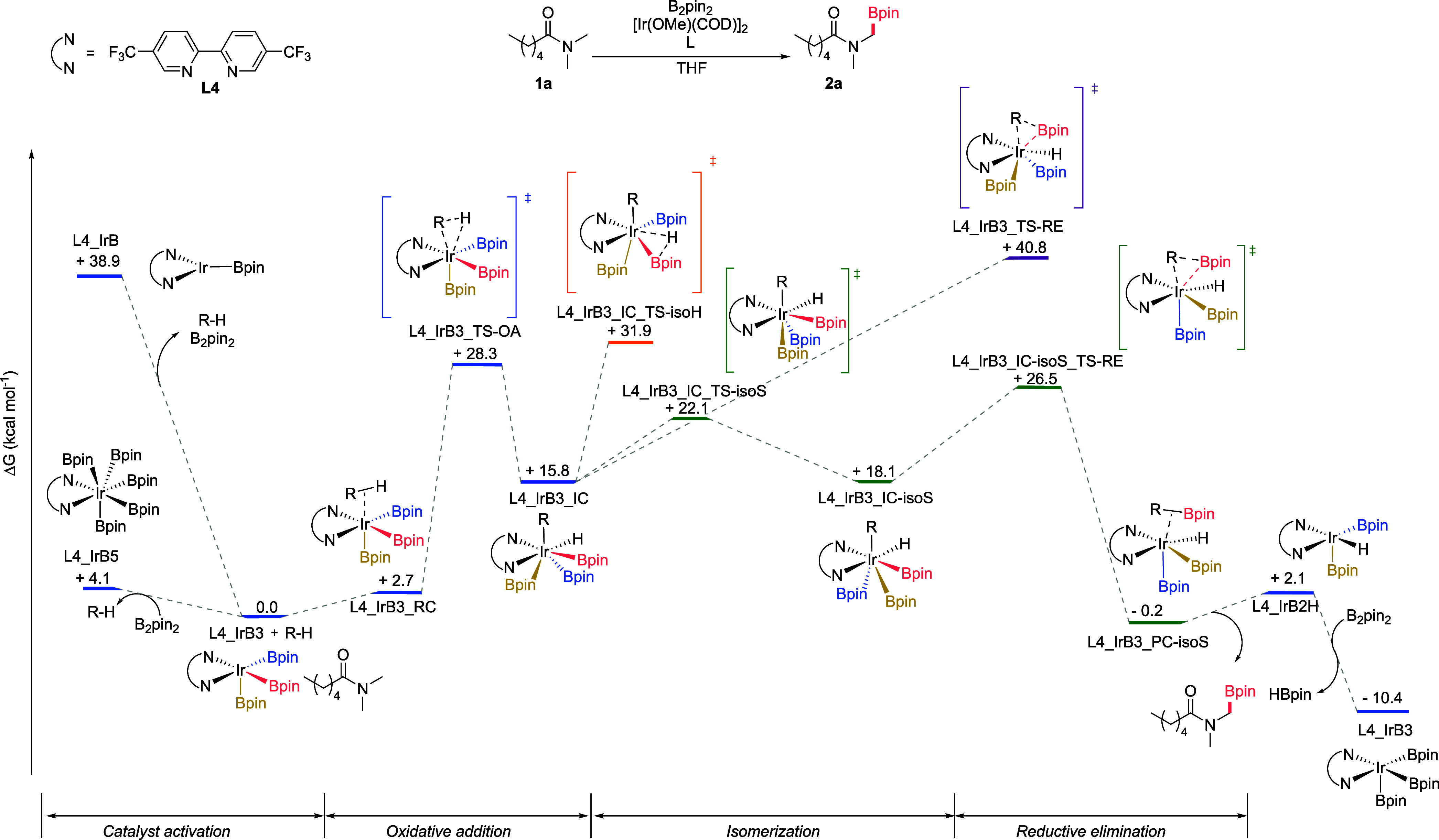
Minimum energy reaction pathway for the Ir-catalyzed borylation of *N*,*N*-dimethylhexanamide with **L4** as a ligand, calculated with SMD_THF_/M06/6–311G­(d,p);SDD­(Ir)]//M06/6–31G­(d);LANL2DZ­(Ir). The chemical structure of relevant stationary points is depicted. For pathways with ligands **L1** and **L2** see the Supporting Information (Figures S6–S7).

Given this high energy barrier for reductive elimination, we assessed alternative pathways considering previous computational studies in related borylations reported by Hartwig,^11^ and by Sakaki.^17^ These results, disclosed in detail in the Supporting Information (Figures S5–S7), led us to find that the more favorable path involves an isomerization akin to the one proposed by Sakaki and co-workers in other borylation processes. This pathway involves the rearrangement of the two boryl groups, and an alteration in the geometry of the complex, transitioning from one distorted pentagonal bipyramide structure to another. In IrB3_IC, the axial positions of the pentagonal bipyramidal structure are occupied by one of the N atoms of the ligand and a boryl group (Bpin in salmon in Figure 2 and Figure 3). Conversely, in IrB3_IC-isoS, the axial positions are occupied by the hydride ligand and the other N atom of the ligand, so that the alkyl and the boron groups are much better positioned for bond formation in the reductive elimination step (see Figure 3 and Figure S14).

**Figure 3:**
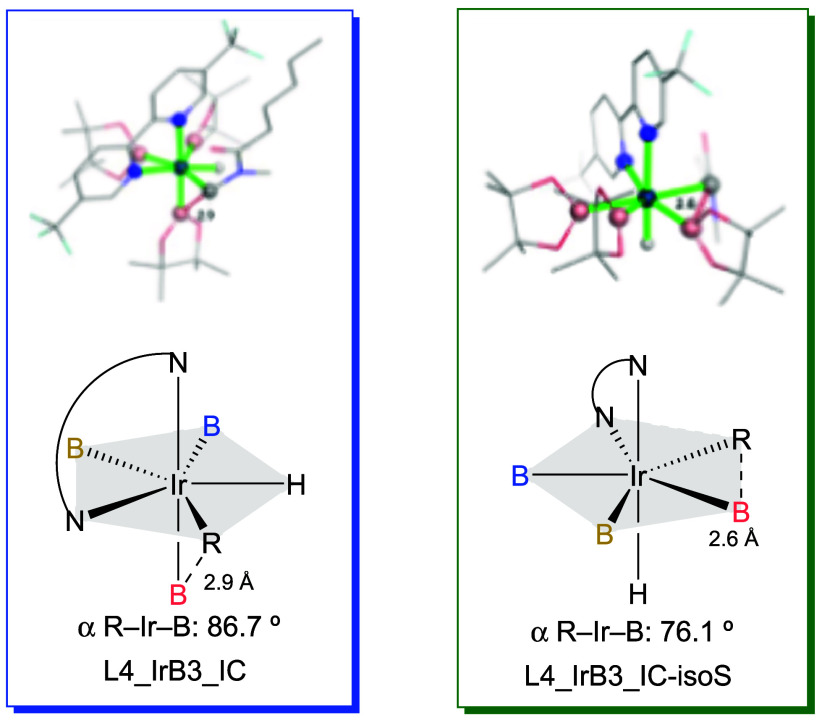
Pentagonal bipyramidal structures corresponding to intermediates IrB3_IC (left) and IrB3_IC-isoS (right).

This isomerization exhibits by far the lowest activation energy (ΔG^‡^ ≈ 22 kcal mol^–1^) among the three possible pathways after C–H activation, yielding a complex able to undergo an easier elimination via transition state IrB3_IC-isoS_TS-RE (ΔG^‡^ ≈ 26 kcal mol^–1^). Ultimately, the catalyst undergoes turnover with B_2_pin_2_, regenerating the Ir­(Bpin)_3_ complex and allowing it to re-enter the catalytic cycle.

This profile suggests that C–H activation dominates the reaction rate with a minor contribution from reductive elimination as both TS are within 2 kcal mol^–1^. Indeed, parallel competition experiments with **1a** and its deuterated equivalent, provided a relatively low KIE value of 1.9, which is in line with those previously observed for other related C­(*sp* ^3^)–H activations (Figure 4).

**Figure 4:**
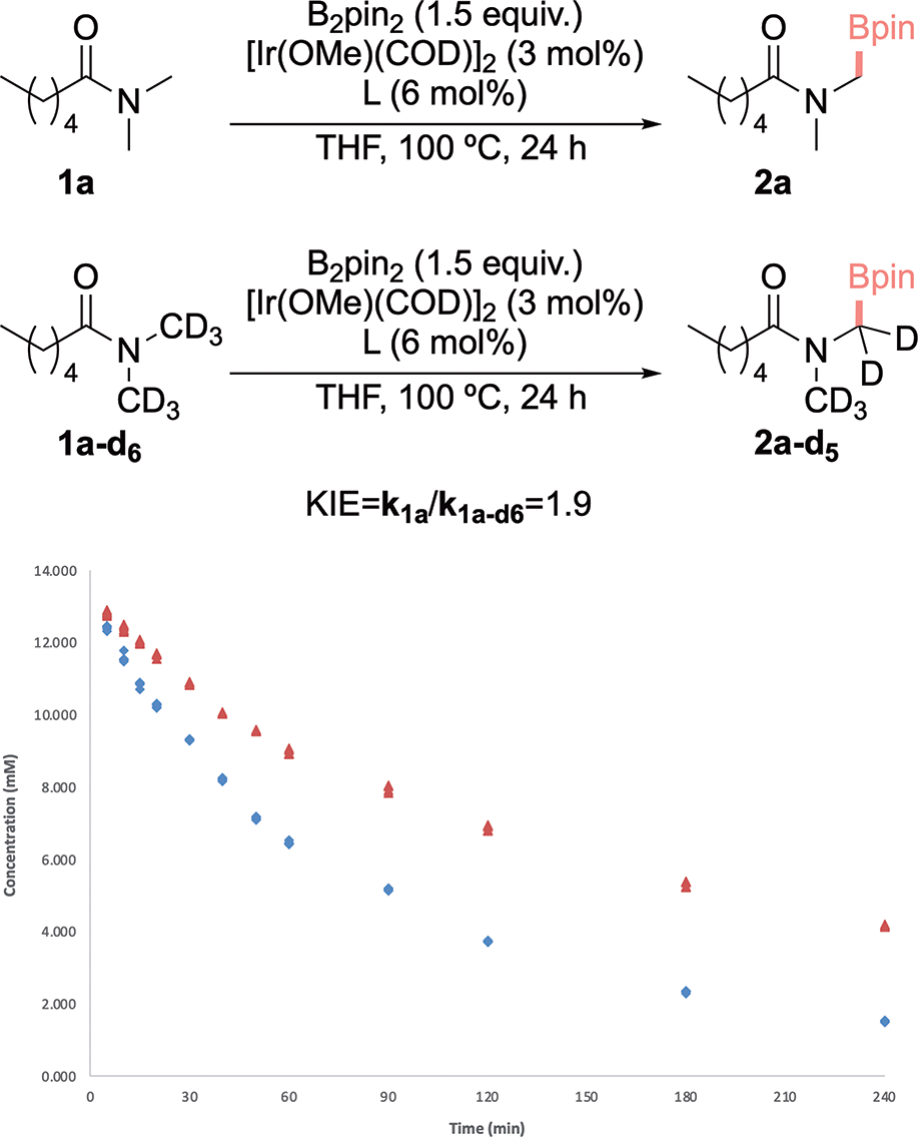
Time-course of the reaction of **1a** (blue markers) or **1a-d**_**6**_ (red markers) and B_2_pin_2_ catalyzed by the combination of [Ir­(OMe)­(COD)]_2_ and 5,5′-bis-CF_3_-bipyridine ligand **L4**. The values of the initial slopes for the reaction of **1a** and **1a-d**_**6**_ were −9 × 10^–3^ and −4.8 × 10^–3^ respectively, resulting in a KIE of 1.9.

Using bipyridine (**L1**) as a ligand, we observed that a similar reaction pathway exhibits higher energy barriers for both the C–H activation and the reductive elimination steps, over 2 kcal mol^–1^ with respect that with **L4**. Overall, the reactivity trend derived from the calculated C–H activation TSs qualitatively agrees with the experimental observations: **L4** (ΔG^‡^ ≈ 28 kcal mol^–1^) > **L2** (ΔG^‡^ ≈ 29 kcal mol^–1^) > **L1** (ΔG^‡^ ≈ 31 kcal mol^–1^).

A close inspection of the oxidative addition transition state structures with ligands **L1** and **L4** reveals important differences in the positioning of the substrate. In the lowest-energy conformer of the transition state with **L1**, the carbonyl is oriented away from the ligand. Conversely, with **L4**, the carbonyl is positioned above one of the pyridine rings of the ligand, in a conformation like that previously reported for the *ortho*-borylation of benzamides.^6^

This finding strongly supports the formation of outer-sphere interactions between the substrate and 5-trifluoromethylated ligands (**L2** and **L4**) in the oxidative addition transition state. Analysis of the non-covalent interaction maps unveiled an extended network of attractive van der Waals interactions between the carbonyl group of the alkyl amide and the CF_3_-pyridine ring of ligands **L2** and **L4** (Figure 5 and Figure S12 in the Supporting Information), which are essentially absent in the case of ligand **L1**.

**Figure 5:**
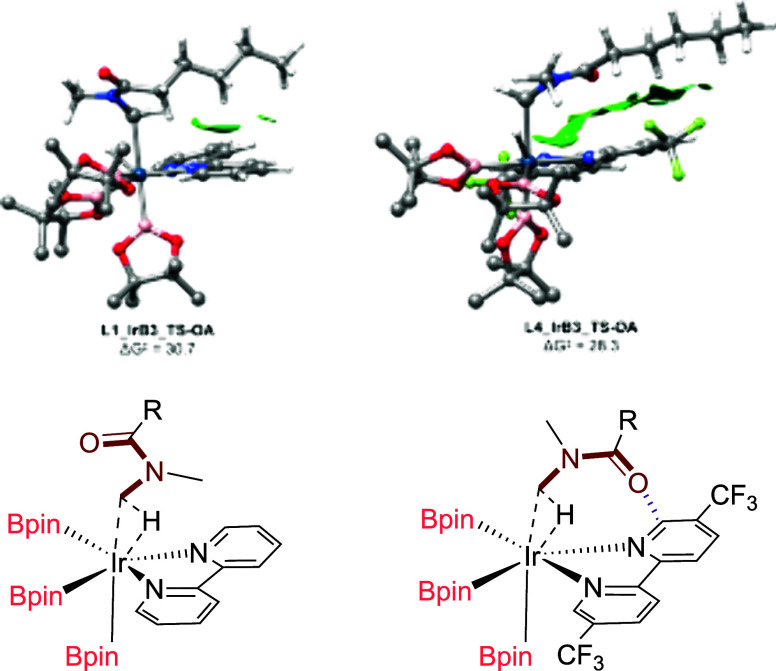
Substrate–ligand noncovalent interactions (NCI) occurring in the lowest energy outer-sphere transition states (TS) calculated for the oxidative addition step between **1a** and Ir^III^(ligand)­(Bpin)_3_ (ligand = **L1** (left) and **L4** (right)). Note the orientation of the carbonyl amide and the van der Waals NCI in the transition state with ligand **L4**. Hydrogens of the Bpin ligands have been omitted for clarity

## Conclusions

In summary, we have discovered that the incorporation of CF_3_ groups at the 5 position of bipyridine ligands enables highly selective *N*-methyl borylations across a diverse range of alkyl *N*-methylamides, with exceptional chemoselectivities. This discovery is particularly significant, as the parent bipyridine (**L1**) fails to yield any product under identical reaction conditions. Computational studies involving both CF_3_ and non-CF_3_ containing ligands provide support for a canonical Ir­(III)/Ir­(V) mechanism, and for lower activation barriers of key transition states with ligand **L4** than with the parent **L1** (bipy). Non-covalent interactions between the polarized ring of the bipyridine ligand and the alkyl amide seem to play a crucial role in stabilizing the C–H activation transition state, thereby facilitating the progress of the reaction. The importance of these dispersion non-covalent interactions, although rarely considered in the past, should be acknowledged for future investigations across various chemical reactions.

## Supplementary Material



## Notes

General information, detailed experimental procedures, characterizations, spectral data, and details of the computational methods (PDF)
