# Supplementary material for: Bis-CF3‑bipyridine Ligands for the Iridium-Catalyzed Borylation of N‑Methylamides
Source: ACS Catal. 2025 Apr 16;15(9):7112–20. doi: 10.1021/acscatal.5c00933 (PMC12128192; doi:10.1021/acscatal.5c00933)
Supplement: Supplementary file 1 [file cs5c00933_si_001.pdf]

Supporting Information

bis-CF_3-Bipyridine ligands for the iridium-catalyzed borylation of N-methylamides

Daniel Marcos-Atanes[†], *Gonzalo Jiménez-Osés*[§*], *José L. Mascareñas*[†*]

† Centro Singular de Investigación en Química Biolóxica e Materiais Moleculares (CiQUS) and Departamento de Química Orgánica. Universidade de Santiago de Compostela, 15782, Santiago de Compostela, Spain.

[§] Center for Cooperative Research in Biosciences (CIC bioGUNE), Basque Research and Technology Alliance (BRTA), Derio 48160, Spain; Ikerbasque, Basque Foundation for Science, Bilbao 48013, Spain

E-mail: joseluis.mascarenas@usc.es; gjoses@cicbiogune.es.

General experimental information ... 3
Computational details: quantum mechanical calculations ... 5
Solvent screening ... 7
Kinetic Isotope effects (KIE) ... 8
Experimental KIE ... 8
Calculated kinetic isotopic effects ... 9
NMR and HRMS studies of $[Ir(COD)Cl]_2$ and L4 complexes ... 10
Experimental procedures and characterization data ... 12
Synthesis of starting materials ... 12
Synthesis of bipyridine ligands ... 29
Procedure for N-Me borylation ... 32
Borylation of aromatic containing substrates with 2,2’-bipyridine (L1) ... 46
Computational analysis ... 48
Calculated Bond Dissociation Energies (BDEs) ... 48
DFT Profiles and alternate pathways ... 49
Non-Covalent Interactions (NCI) analysis ... 56
Energy Decomposition Analysis (EDA) ... 57
Pentagonal bipyramidal structures of key intermediates ... 58
Calculated structures ... 59
Energies, entropies, and lowest frequencies of optimized structures ... 62
References ... 65
NMR spectral data ... 67
Cartesian coordinates of calculated structures ... 195

General experimental information

Unless specified otherwise, all commercially available reagents and solvents were used as received. Reactions were conducted in dry solvents unless otherwise stated. Procedures for the synthesis of precursors were performed under a dry nitrogen atmosphere using vacuum-line and standard Schlenk techniques. Dry solvents were directly purchased from Sigma Aldrich or Across Organics (Extra Dry over Molecular Sieves), and used without further purification. Chemicals were purchased from Sigma Aldrich, Alfa Aesar, TCI, Fluorochem, Apollo Scientific or BLDpharm and used without further purification. All catalytic reactions were carried out with THF from a solvent purification system (MBraun, SPS-5) stored over Molecular Sieves.

Reaction mixtures were stirred using Teflon-coated magnetic stir bars. The abbreviation "r.t." refers to reactions carried out approximately between 21-25 °C. Temperature was maintained using Thermowatch-controlled heating blocks. Thin-layer chromatography (TLC) was performed on pre-coated silica gel F254 plates and components were visualized by observation under UV light and / or by treating the plates with $KMnO_4$ or *p*-anisaldehyde followed by heating. Flash chromatography was carried out on silica gel (40-63 μm) or using a Büchi Pure Chromatography system.

Concentration refers to the removal of volatile solvents via distillation using a rotary evaporator Büchi R-210 equipped with a thermostated bath B-491, a vacuum regulator V-850, followed by residual solvent removal under high vacuum.

NMR data was collected at room temperature or at 258 K at the CiQUS facility of the University of Santiago de Compostela on a Varian Mercury 300 MHz spectrometer and at the CACTI facility of the University of Vigo on a Bruker Advance 400 MHz or Bruker NEO 400 MHz spectrometer. Data are represented as follows: chemical shift, multiplicity (s = singlet, d = doublet, t = triplet, q = quartet, m = multiplet, br = broad signal, bs = broad singlet, dd = doublet of doublets, dt = doublet of triplets, dq = doublet of quartets, td = triplet of doublets, ddd = doublet of doublet of doublets, ddt = doublet of doublet of triplets, dtd = doublet of triplet of doublets, tt = triplet of triplets, pd = pentuplet of doublets, h=hexaplet dqd = doublet of quartets of doublets), coupling constants in Hertz (Hz). The chemical shifts for protons (δ) are reported in parts per million downfield from tetramethylsilane and are referenced to residual protium in the NMR solvent ($CHCl_3$ δ = 7.26). Chemical shifts for carbon are reported in parts per million downfield from tetramethylsilane and are referenced to the carbon resonances of the solvent ($CDCl_3$ δ = 77.0). In the ^{13}C NMR spectra of the borylated products, the carbon connected with the boron atom was not always observed due to quadrupolar relaxation. The chemical shifts for fluor (δ) are reported in parts per million relative to trichlorofluoromethane. The

chemical shifts for boron (δ) are reported in parts per million relative to $BF_3 \cdot OEt_2$. NMR spectra were analyzed using MestreNova© NMR data processing software (www.mestrelab.com).

High Resolution Mass Spectra (HRMS) were performed at the CACTI facility of the University of Vigo on a Bruker TIMS-TOF or a SOLARIX XR FT-ICR-MS 7T. Samples were prepared by adding a spray solution of 70:29.9:0.1 (v/v/v) CH_3OH/water/formic acid or 70:29.9:0.1 (v/v/v) CH_3CN/water/formic acid to a solution of the sample. M+Na masses of borylated products were obtained after diluting an aliquot in a saturated solution of NaCl in MeOH/CH_2Cl_2 1:1 (v/v). Mass spectrometry data was analyzed using Bruker DataAnalysis Version 5.1 data processing software.

GC/MS analysis (CI) were performed on an Agilent 5977B GC/MSD equipped with a 30 m HP-5MS column. Method: Initial temperature = 70 °C for 3 min, then 30 °C/min to 300 °C and hold for 8 min. Data was processed using Agilent MassHunter Qualitative Analysis 10.0 software.

Computational details: quantum mechanical calculations

Full geometry optimizations and transition structure (TS) searches were carried out with Gaussian 16[1] using the M06 hybrid functional,[2] the 6-31G(d)[3,4] basis set and the LANL2DZ[5] effective core potential for Ir, with ultrafine integration grids. The possibility of different conformations was considered for all structures. All stationary points were characterized by a frequency analysis performed at the same level used in the geometry optimizations from which thermal corrections were obtained at 298.15 K. The quasiharmonic approximations reported by Truhlar et al. was used to replace the rigid-rotor harmonic oscillator (RRHO) approximation for the calculation of the vibrational contribution to enthalpy and entropy.[6] Scaled frequencies were not considered. Mass-weighted intrinsic reaction coordinate (IRC) calculations were carried out using the Hessian-based predictor-corrector integrator scheme by Hratchian and Schlegel[7,8] in order to ensure that the TSs indeed connected the appropriate reactants and products. Single-point energies were calculated on the optimized geometries using the M06 functional and 6-311G(2d,p)[9] basis set together with the SDD[10] effective core potential for Ir, with ultrafine integration grids. Bulk solvent effects in tetrahydrofuran were considered implicitly through the SMD[11] polarizable continuum model. Gibbs free energies (ΔG) were used for the discussion on the relative stabilities of the considered structures unless otherwise stated. The lowest energy conformer for each calculated stationary point was considered in the discussion of calculated selectivity. Cartesian coordinates, electronic energies, entropies, enthalpies, Gibbs free energies, and lowest frequencies of the calculated structures are summarized below). All additional calculations can be obtained from the authors upon request.

Bond dissociation energies (BDEs) were estimated by performing single-point energy calculations using the correlated ab initio SCS-MP2 methods[12] in combination with the aug-cc-pVTZ basis set,[13] on the M06-2X/6-311+G(2d,p) optimized geometries of minimal model compounds. BDEs were defined as the difference in zero-point energies between the parent compound and the sum of the isolated radicals generated upon homolytic –H cleavage.

^{2}D/^{1}H Kinetic isotopic effects (KIE) were calculated at 298 K using Kinisot.py program[14] on the Gaussian output files corresponding to the lowest-energy optimized minima and transition states. The program diagonalizes the mass-weighted Hessian matrices to obtain harmonic vibrational frequencies and Bigeleisen-Mayer Reduced Isotopic Partition Function Ratios. The lowest six normal modes (translations and rotations) were not projected out. The one-dimensional tunnelling correction (Bell infinite-parabola model) was not used. Different scaling factors for vibrational frequencies in the range between 0.9 and 1.1 were tested.

Non-covalent interaction (NCI) analysis was performed with NCIPLOT3[15,16] using the promolecular density in the intermolecular mode. The NCI methodology is based on the analysis of the electron density (ρ) and its reduced density gradient (s). By mapping ρ and s on a three-dimensional grid centered on the molecule of interest, NCIs can be identified as the grid points in which ρ and s fall under a given threshold. Additionally, the sign of the second eigenvalue of the Hessian of ρ allows differentiation between van der Waals interactions, strong attractive interactions such as hydrogen bonds, and steric clashes.

Electrostatic (E_{elec}), exchange (E_{ex}), repulsion (E_{rep}), polarization (E_{pol}), electron correlation (E_{corr}, which implicitly accounts for dispersion for the M06 functional) and desolvation (E_{desol}) contributions to interaction energies (E_{int}) between the catalyst and the substrate were calculated through the Generalized Kohn-Sham Energy Decomposition Analysis (GKS-EDA)[17] as implemented in the XEDA program[18] based in GAMESS 2021 R2.[19]

Solvent screening

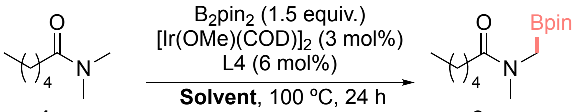

In a sealed tube $[Ir(COD)(OMe)]_2$ (5.0 mg, 0.008 mmol, 3 mol%.), B_2pin_2 (95.2 mg, 0.375 mmol, 1.5 equiv.), and **L4** (4.4 mg, 0.015 mmol, 6 mol%) were added. The tube was evacuated and refilled with N_2 three times, then under a positive flow of N_2, dry solvent (1.25 mL) and **1a** (35.8 mg, 0.250 mmol, 1.0 equiv.) were added. The tube was then sealed and heated at 100 °C for 24 h.

Table S1. Evaluation of different solvents for the N-Me borylation.[a]

Entry	**Solvent**	Ratio **1a**:**2a**
1	**THF**	0:100
2	**2-MeTHF**	40:60
3	**1,2-DME**	37:63
4	**2,2,4-Trimethylpentane**	10:90
5	**Methyl-^{t}Butylether**	0:100
6	**1,3-Dioxolane**	83:17
7	**Cyclopentyl-Methylether**	35:65
8	**Cyclooctane**	36:64
9	**Di-^{n}Butylether**	4:96
10	**^{t}Amyl-Methylether**	0:100
11	**NMP**	100:0
12	***p*-Xylene**	31:69
13	**1,2-DCE**	100:0
14	**1,4-Dioxane**	81:19
15	**MeCN**	100:0
16	**Heptane**	16:84
17	**Decahydronaphtalene**	5:95
18	**Isopropylcyclohexane**	6:94
19	**^{t}Butylcyclohexane**	10:90

[a] Ratio of each product was determined by GC/MS. THF= Tetrahydrofuran; 1,2-DME= 1,2-dimethoxyethane; NMP= 1-Methyl-2-pyrrolidinone; 1,2-DCE= 1,2-dichloroethane

Kinetic Isotope effects (KIE)

Experimental KIE

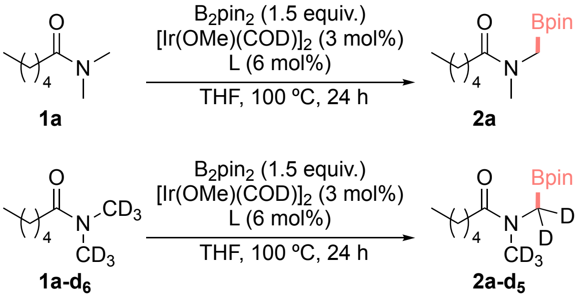

In a sealed tube $[Ir(COD)(OMe)]_2$ (5.0 mg, 0.008 mmol, 3 mol%.) and B_2pin_2 (95.2 mg 0.375 mmol, 1.5 equiv.) were added. The tube was evacuated and refilled with N_2 three times, then under a positive flow of N_2 the solids were dissolved in dry THF (1 mL). To that solution were added 125 μL of a solution 0.12 M of **L4** in THF (4.4 mg, 0.015 mmol, 6 mol%) and 125 μL of a solution 2 M of **1a** (35.8 mg, 0.250 mmol, 1.0 equiv.) or **1a-d₆** (37.3 mg, 0.250 mmol, 1.0 equiv.) in THF. The tube was then closed and heated at 100 °C for the amount of time necessary.

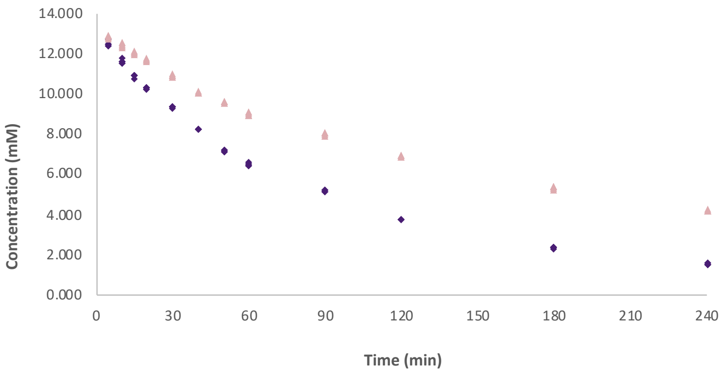

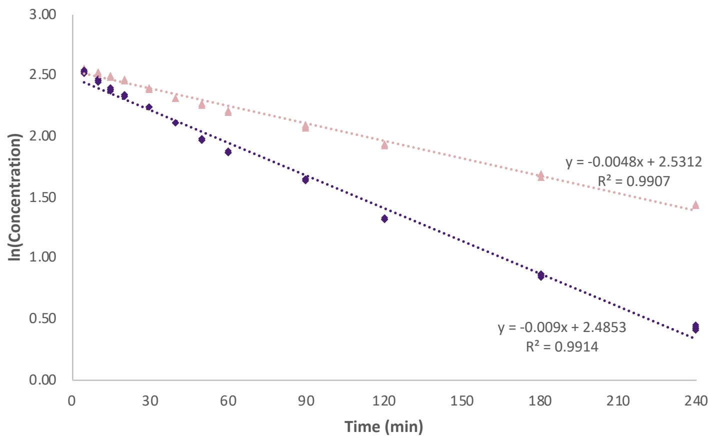

Figure S1. Profile of the reaction of **1a** (purple markers) or **1a-d₆** (pink markers) and B_2pin_2 catalyzed by the combination of $[Ir(OMe)(COD)]_2$ and 5,5'-bis-CF_3-bipyridine ligand **L4**. The values of the slopes for the reaction of **1a** and **1a-d₆** were -9×10^{-3} and -4.8×10^{-3} respectively, resulting in a KIE of 1.9.

Calculated kinetic isotopic effects

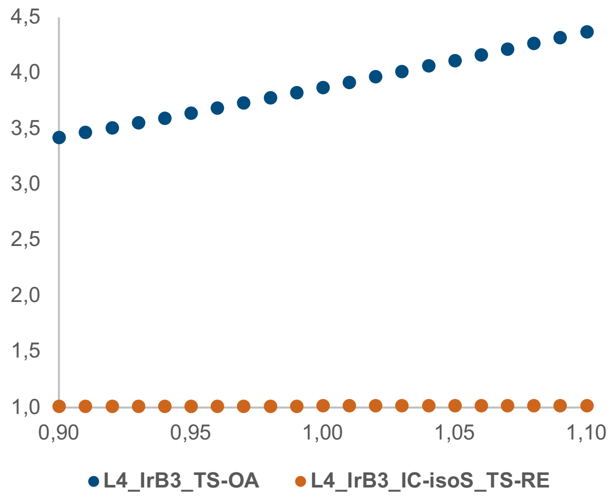

Figure S2. $^{2}D/^{1}H$ Kinetic isotopic effects (KIE) calculated at 298 K with M06/6-31G(d); LANL2DZ(Ir) for the oxidative addition (in blue) and reductive elimination (in orange) steps in the borylation of N,N-dimethylhexanamide (**1a**) catalyzed by IrIII(**L4**)(Bpin)$_3$., using different scaling factors for vibrational frequencies.

NMR and HRMS studies of $[Ir(COD)Cl]_2$ and L4 complexes

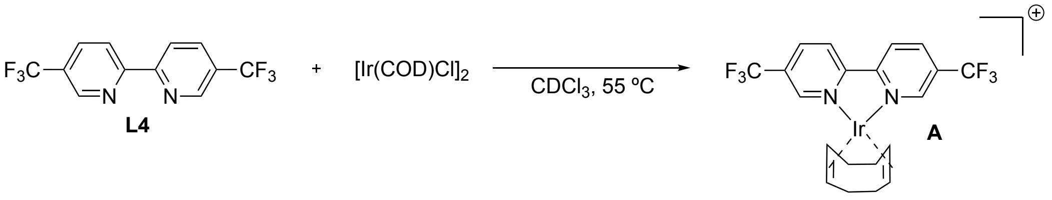

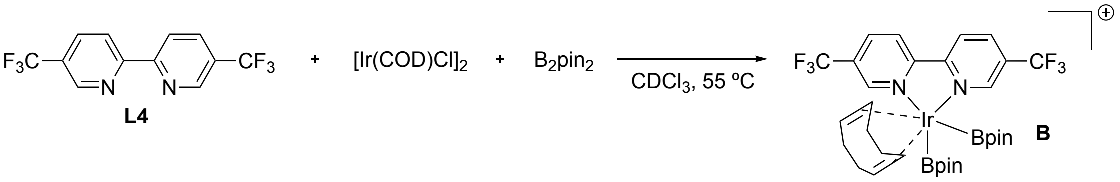

An NMR tube equipped with a screw-cap and septum was charged with either **L4** (top spectra), **L4** (2 equiv.) and $[Ir(COD)Cl]_2$ (1 equiv.) (middle spectra) or **L4** (2 equiv.) and $[Ir(COD)Cl]_2$ (1 equiv.) and B_2pin_2 (1.5 equiv.) (bottom spectra). The tubes were evacuated and refilled with Ar 3 times and the solids were dissolved in dry $CDCl_3$ (0.5 mL). Those tubes were heated up to 55 ºC and the reactions were monitored until no evolution was observed by NMR spectra.

The HRMS spectra samples were prepared by taking an aliquot from the correponding NMR tubes after no evolution was observed. The aliquot was diluted with dry THF and injected into a SOLARIX XR FT-ICR-MS 7T spectrometer. The fact that we can observe the M^+ ion of our compound even without any ionization agent included in the sprayed solution indicates that we have successfully obtained a cationic complex

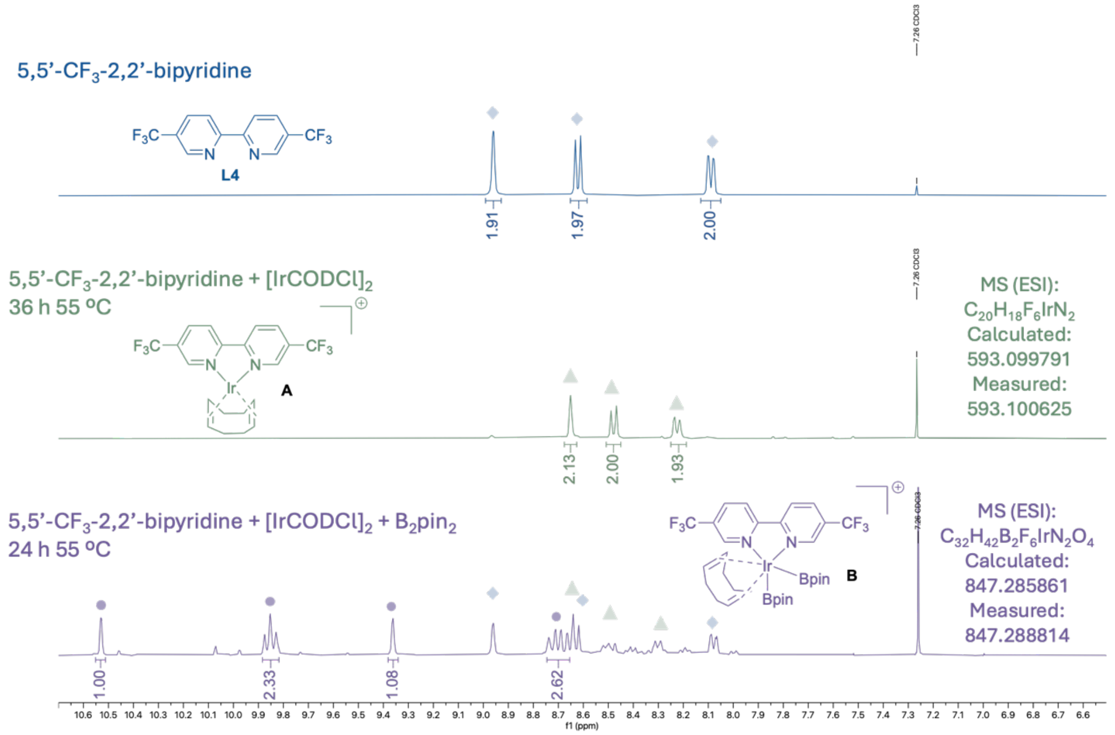

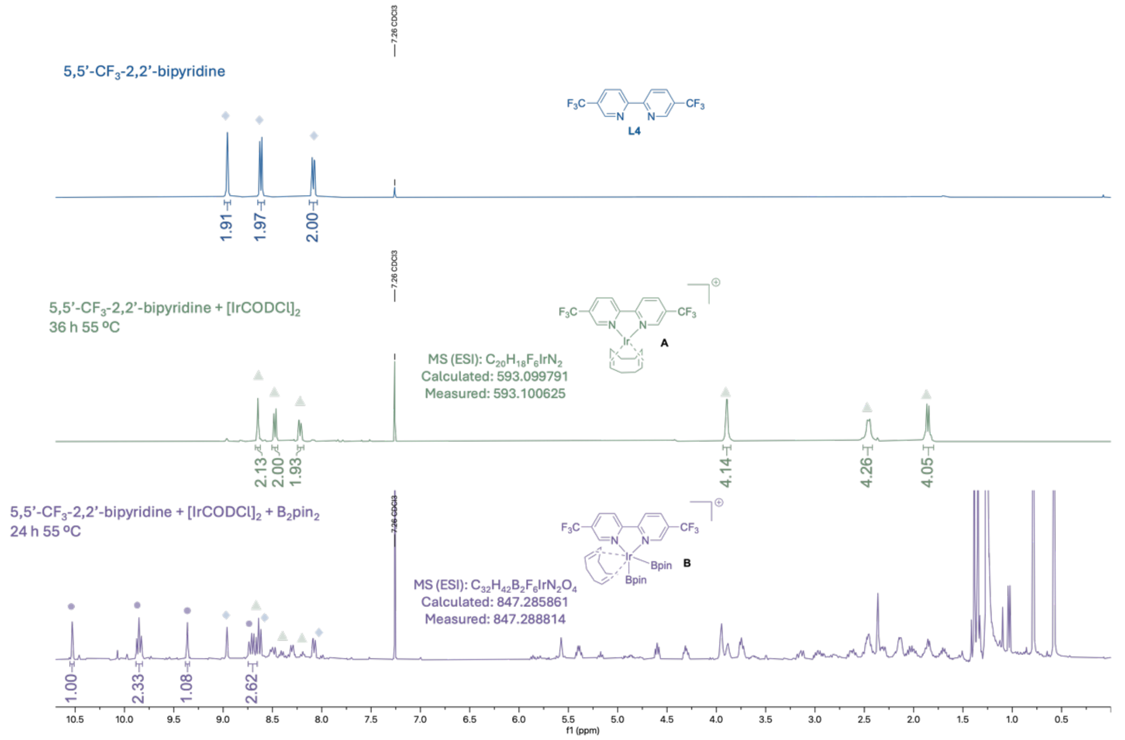

Figure S3. NMR and HRMS studies on the formation of complex **A** and **B**. Expansion of the aromatic region (top) and full NMR spectra (bottom). Blue markers correspond to signals for **L4**, green markers to signals from complex **A** and purple markers to signals from complex **B**.

Experimental procedures and characterization data

Synthesis of starting materials

Preparation of N,N-dimethylhexanamide (1a)

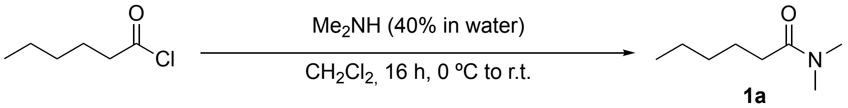

Prepared following an adapted procedure: To a solution of hexanoyl chloride (0.70 mL, 5.0 mmol, 1 equiv.) in CH_2Cl_2 (10 mL) at 0 °C, was added dropwise Me_2NH (40 w/w% in water; 2.5 mL, 20.0 mmol, 4 equiv.). The reaction mixture was stirred continuously at room temperature overnight.

The reaction was then quenched with water, and organic materials were extracted twice with CH_2Cl_2, washed first with 1M aqueous HCl solution followed by a second wash with saturated aqueous $NaHCO_3$ solution and brine. The combined organic fractions were dried over $MgSO_4$ and the solvent was removed in vacuo to obtain the *N,N*- dimethylhexanamide in a 92% yield (659 mg).

^{1}H NMR (400 MHz, $CDCl_3$, 258 K) δ 2.97 (s, 3H), 2.90 (s, 3H), 2.31 – 2.23 (m, 2H), 1.57 (dt, *J* = 15.4, 7.5 Hz, 2H), 1.26 (q, *J* = 2.7 Hz, 4H), 0.84 (t, *J* = 6.8 Hz, 3H).
^{13}C NMR (101 MHz, $CDCl_3$, 258 K) δ 173.43 (CO), 37.41 (N-CH_3), 35.37 (N-CH_3), 33.47 (CH_2), 31.67 (CH_2), 24.94 (CH_2), 22.60 (CH_2), 14.16 (CH_3).
HRMS (ESI). Calculated for $C_8H_{18}NO^+$: 144.1383 Obtained: 144.1383

Preparation of N,N-dimethylpropionamide (1c)

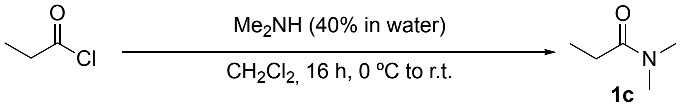

Prepared following an adapted procedure: To a solution of propionyl chloride (0.44 mL, 5.0 mmol, 1 equiv.) in CH_2Cl_2 (20 mL) at 0 °C, was added dropwise Me_2NH (40 w/w% in water; 2.5 mL, 20.0 mmol, 4 equiv.). The reaction mixture was stirred continuously at room temperature overnight.

The reaction was then quenched with water, and organic materials were extracted twice with CH_2Cl_2, washed first with 1M aqueous HCl solution followed by a second wash with saturated aqueous $NaHCO_3$ solution and brine. The combined organic fractions were dried over $MgSO_4$ and the solvent was removed in vacuo to obtain the *N,N*-dimethylpropionamide in a 68% yield (343 mg).

^{1}H NMR (400 MHz, $CDCl_3$, 258 K) δ 2.98 (s, 3H), 2.92 (s, 3H), 2.32 (q, *J* = 7.5 Hz, 2H), 1.10 (t, *J* = 7.5 Hz, 3H).
^{13}C NMR (101 MHz, $CDCl_3$, 258 K) δ 173.97 (CO), 37.22 (N-CH_3), 35.45 (N-CH_3), 26.66 (CH_2), 9.47 (CH_3).
HRMS (ESI). Calculated for $C_5H_{12}NO^+$: 102.0913 Obtained: 102.0916

Preparation of N,N-dimethylbutyramide (1d)

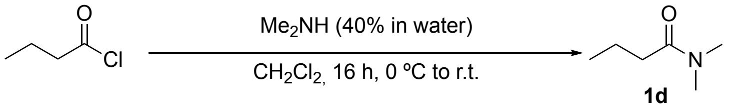

1d

Prepared following an adapted procedure: To a solution of butyryl chloride (0.52 mL, 5.0 mmol, 1 equiv.) in CH_2Cl_2 (20 mL) at 0 ºC, was added dropwise Me_2NH (40 w/w% in water; 2.5 mL, 20.0 mmol, 4 equiv.). The reaction mixture was stirred continuously at room temperature overnight.

The reaction was then quenched with water, and organic materials were extracted twice with CH_2Cl_2, washed first with 1M aqueous HCl solution followed by a second wash with saturated aqueous $NaHCO_3$ solution and brine. The combined organic fractions were dried over $MgSO_4$ and the solvent was removed in vacuo to obtain the *N,N*-dimethylbutyramide in a 67% yield (385 mg).

^{1}H NMR (400 MHz, $CDCl_3$, 258 K) δ 2.97 (s, 3H), 2.90 (s, 3H), 2.31 – 2.22 (m, 2H), 1.60 (dq, *J* = 14.8, 7.4 Hz, 2H), 0.91 (t, *J* = 7.4 Hz, 3H).
^{13}C NMR (101 MHz, $CDCl_3$, 258 K) δ 173.25 (CO), 37.39 (N-CH_3), 35.39 (CH_2), 35.35 (N-CH_3), 18.61 (CH_2), 14.14 (CH_2).
HRMS (ESI). Calculated for $C_6H_{14}NO^+$: 116.10699 Obtained: 116.10708

Preparation of N,N-dimethylmyristoylamide (1e)

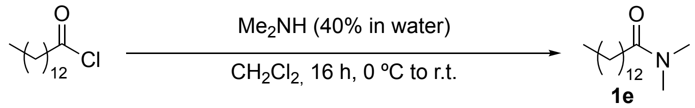

1e

Prepared following an adapted procedure: To a solution of myristoyl chloride (0.93 mL, 3.4 mmol, 1 equiv.) in CH_2Cl_2 (20 mL) at 0 ºC, were added dropwise Me_2NH (40 w/w% in water; 0.6 mL, 5.1 mmol, 1.5 equiv.) and triethylamine (1.4 mL, 10.2 mmol, 3 equiv.). The reaction mixture was stirred continuously at room temperature overnight.

The reaction was then quenched with water, and organic materials were extracted twice with CH_2Cl_2, washed first with 1M aqueous HCl solution followed by a second wash with saturated aqueous $NaHCO_3$ solution and brine. The combined organic fractions were dried over $MgSO_4$ and the solvent was removed in vacuo to obtain the *N,N*-dimethylmyristoylamide in a 86% yield (749 mg).

^{1}H NMR (400 MHz, $CDCl_3$, 258 K) δ 3.01 (s, 3H), 2.95 (s, 3H), 2.37 – 2.25 (m, 2H), 1.66 – 1.53 (m, 2H), 1.27 (s, 6H), 1.21 (s, 14H), 0.91 – 0.80 (m, 3H).
^{13}C NMR (101 MHz, $CDCl_3$, 258 K) δ 173.74 (CO), 37.57 (N-CH_3), 35.57 (N-CH_3), 33.52 (CH_2), 32.05 (CH_2), 29.83 (CH_2), 29.81 (CH_2), 29.78 (CH_2), 29.67 (CH_2), 29.63 (CH_2), 29.61 (CH_2), 29.55 (CH_2), 25.34 (CH_2), 22.87 (CH_2), 14.40 (CH_3).
HRMS (ESI). Calculated for $C_{16}H_{34}NO^+$: 256.26349 Obtained: 256.26423

Preparation of N,N-dimethylpivalamide (1g)

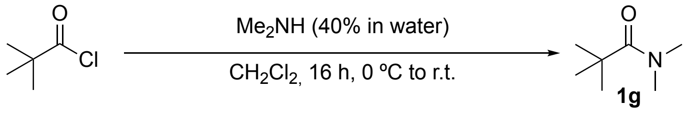

Prepared following an adapted procedure: To a solution of pivaloyl chloride (0.61 mL, 5.0 mmol, 1 equiv.) in CH_2Cl_2 (20 mL) at 0 °C, was added dropwise Me_2NH (40 w/w% in water; 2.5 mL, 20.0 mmol, 4 equiv.). The reaction mixture was stirred continuously at room temperature overnight.

The reaction was then quenched with water, and organic materials were extracted twice with CH_2Cl_2, washed first with 1M aqueous HCl solution followed by a second wash with saturated aqueous $NaHCO_3$ solution and brine. The combined organic fractions were dried over $MgSO_4$ and the solvent was removed in vacuo to obtain the *N,N*-dimethylpivalamide in a 66% yield (426 mg).

^{1}H NMR (400 MHz, $CDCl_3$, 258 K) δ 3.09 (s, 3H), 2.93 (s, 3H), 1.25 (s, 9H).
^{13}C NMR (101 MHz, $CDCl_3$, 258 K) δ 177.61 (CO), 38.76 (N-CH_3), 38.63 (C), 38.07 (N-CH_3), 28.16 (CH_3).
HRMS (ESI). Calculated for $C_7H_{16}NO^+$: 130.1226 Obtained: 130.1226

Preparation of N,N,3-trimethylbutanamide (1h)

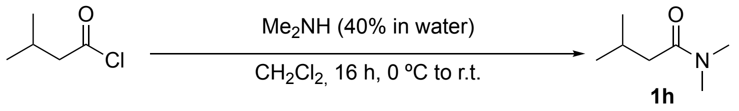

Prepared following an adapted procedure: To a solution of 3-methylbutanoyl chloride (0.61 mL, 5.0 mmol, 1 equiv.) in CH_2Cl_2 (20 mL) at 0 °C, was added dropwise Me_2NH (40 w/w% in water; 2.5 mL, 20.0 mmol, 4 equiv.). The reaction mixture was stirred continuously at room temperature overnight.

The reaction was then quenched with water, and organic materials were extracted twice with CH_2Cl_2, washed first with 1M aqueous HCl solution followed by a second wash with saturated aqueous $NaHCO_3$ solution and brine. The combined organic fractions were dried over $MgSO_4$ and the solvent was removed in vacuo to obtain the *N,N,3*-trimethylbutanamide in a 64% yield (413 mg).

^{1}H NMR (400 MHz, $CDCl_3$, 258 K) δ 2.98 (s, 3H), 2.91 (s, 3H), 2.16 (d, *J* = 6.5 Hz, 2H), 2.08 (ddd, *J* = 12.8, 7.9, 6.4 Hz, 1H), 0.91 (d, *J* = 6.5 Hz, 6H).
^{13}C NMR (101 MHz, $CDCl_3$, 258 K) δ 172.79 (CO), 42.06 (CH_2), 37.63 (N-CH_3), 35.38 (N-CH_3), 25.67 (CH), 22.78 (CH_3).
HRMS (ESI). Calculated for $C_7H_{15}NO^+$: 130.1226 Obtained: 130.1228

Preparation of N,N,3,3-tetramethylbutanamide (1i)

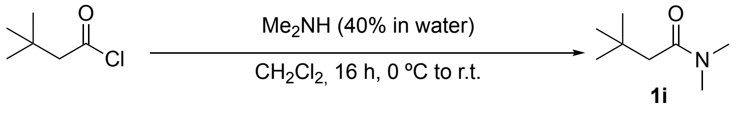

Prepared following an adapted procedure: To a solution of pivaloyl chloride (0.61 mL, 5.0 mmol, 1 equiv.) in CH_2Cl_2 (20 mL) at 0 ºC, was added dropwise Me_2NH (40 w/w% in water; 2.5 mL, 20.0 mmol, 4 equiv.). The reaction mixture was stirred continuously at room temperature overnight.

The reaction was then quenched with water, and organic materials were extracted twice with CH_2Cl_2, washed first with 1M aqueous HCl solution followed by a second wash with saturated aqueous $NaHCO_3$ solution and brine. The combined organic fractions were dried over $MgSO_4$ and the solvent was removed in vacuo to obtain the *N,N*-dimethylpivalamide in a 66% yield (426 mg).

^{1}H NMR (400 MHz, $CDCl_3$, 258 K) δ 3.09 (s, 3H), 2.93 (s, 3H), 1.25 (s, 9H).
^{13}C NMR (101 MHz, $CDCl_3$, 258 K) δ 177.61 (CO), 38.76 (N-CH_3), 38.63 (C), 38.07 (N-CH_3), 28.16 (CH_3).
HRMS (ESI). Calculated for $C_7H_{16}NO^+$: 130.1226 Obtained: 130.1226

Preparation of N,N-dimethylcyclopentanecarboxamide (1j)

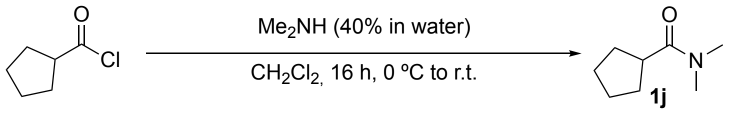

Prepared following an adapted procedure: To a solution of cyclopentanecarbonyl chloride (0.41 mL, 3.4 mmol, 1 equiv.) in CH_2Cl_2 (20 mL) at 0 ºC, were added dropwise Me_2NH (40 w/w% in water; 0.8 mL, 6.8 mmol, 2 equiv.) and triethylamine (1.4 mL, 10.2 mmol, 3 equiv.). The reaction mixture was stirred continuously at room temperature overnight.

The reaction was then quenched with water, and organic materials were extracted twice with CH_2Cl_2, washed first with 1M aqueous HCl solution followed by a second wash with saturated aqueous $NaHCO_3$ solution and brine. The combined organic fractions were dried over $MgSO_4$ and the solvent was removed in vacuo to obtain the *N,N*-dimethylcyclopentanecarboxamide in a 92% yield (440 mg).

^{1}H NMR (400 MHz, $CDCl_3$, 258 K) δ 3.03 (s, 3H), 2.92 (s, 3H), 2.86 (q, *J* = 7.9 Hz, 1H), 1.86 – 1.63 (m, 6H), 1.59 – 1.45 (m, 2H).
^{13}C NMR (101 MHz, $CDCl_3$, 258 K) δ 176.31 (CO), 41.11 (CH), 37.29 (N-CH_3), 35.68 (N-CH_3), 29.96 (CH_2), 26.04 (CH_2).
HRMS (ESI). Calculated for $C_8H_{16}NO^+$: 142.12264 Obtained: 142.12257

Preparation of N,N-dimethylcyclohexanecarboxamide (1k)

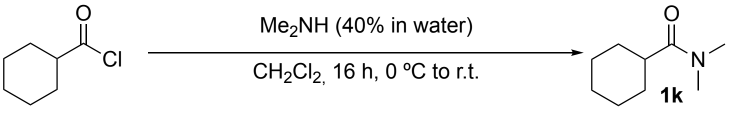

Prepared following an adapted procedure: To a solution of cyclohexanecarbonyl chloride (0.67 mL, 5.0 mmol, 1 equiv.) in CH_2Cl_2 (20 mL) at 0

°C, was added dropwise Me_2NH (40 w/w% in water; 2.5 mL, 20.0 mmol, 4 equiv.). The reaction mixture was stirred continuously at room temperature overnight.

The reaction was then quenched with water, and organic materials were extracted twice with CH_2Cl_2, washed first with 1M aqueous HCl solution followed by a second wash with saturated aqueous $NaHCO_3$ solution and brine. The combined organic fractions were dried over $MgSO_4$ and the solvent was removed in vacuo to obtain the *N,N*-dimethylcyclohexanecarboxamide in a 73% yield (566 mg).

^{1}H NMR (400 MHz, $CDCl_3$, 258 K) δ 2.99 (s, 3H), 2.87 (s, 3H), 2.43 (tt, *J* = 11.7, 3.3 Hz, 1H), 1.77 – 1.58 (m, 5H), 1.41 (qd, *J* = 12.1, 3.0 Hz, 2H), 1.28 – 1.08 (m, 3H).
^{13}C NMR (101 MHz, $CDCl_3$, 258 K) δ 176.15 (CO), 40.46 (CH), 37.11 (N-CH_3), 35.45 (N-CH_3), 28.97 (CH_2), 25.75(CH_2), 25.70(CH_2).
HRMS (ESI). Calculated for $C_9H_{18}NO^+$: 156.13829 Obtained: 156.13832

Preparation of N,N-dimethylcycloheptanecarboxamide (1l)

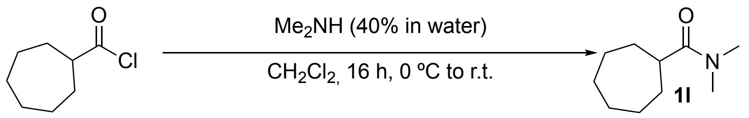

Prepared following an adapted procedure: To a solution of cycloheptanecarbonyl chloride (546 mg, 3.4 mmol, 1 equiv.) in CH_2Cl_2 (20 mL) at 0 °C, was added dropwise Me_2NH (40 w/w% in water; 1.7 mL, 13.6 mmol, 4 equiv.). The reaction mixture was stirred continuously at room temperature overnight.

The reaction was then quenched with water, and organic materials were extracted twice with CH_2Cl_2, washed first with 1M aqueous HCl solution followed by a second wash with saturated aqueous $NaHCO_3$ solution and brine. The combined organic fractions were dried over $MgSO_4$ and the solvent was removed in vacuo to obtain the *N*,*N*-dimethylcycloheptanecarboxamide in a 86% yield (496 mg).

^{1}H NMR (400 MHz, $CDCl_3$, 258 K) δ 3.01 (s, 3H), 2.90 (s, 3H), 2.61 (ddt, *J* = 11.6, 9.9, 3.6 Hz, 1H), 1.79 – 1.68 (m, 4H), 1.67 – 1.56 (m, 2H), 1.55 – 1.46 (m, 4H), 1.46 – 1.33 (m, 2H).
^{13}C NMR (101 MHz, $CDCl_3$, 258 K) δ 177.46 (CO), 41.60 (CH), 37.34 (N-CH_3), 35.57 (N-CH_3), 31.01 (CH_2), 28.03 (CH_2), 26.68 (CH_2).
HRMS (ESI). Calculated for $C_{10}H_{20}NO^+$: 170.15394 Obtained: 170.15392

Preparation of N,N-dimethyladamantane-1-carboxamide (1m)

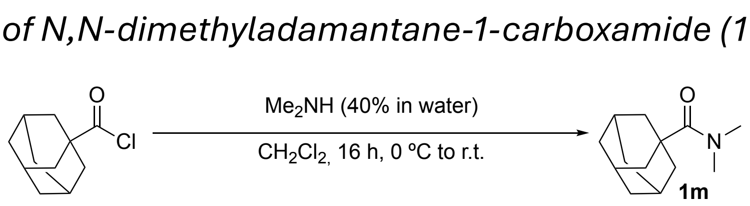

Prepared following an adapted procedure: To a solution of adamantane-1-carbonyl chloride (678 mg, 3.4 mmol, 1 equiv.) in CH_2Cl_2 (20 mL) at 0 °C, were added dropwise Me_2NH (40 w/w% in water; 0.6 mL, 5.1 mmol, 1.5 equiv.) and triethylamine (1.4 mL, 10.2 mmol, 3 equiv.). The reaction mixture was stirred continuously at room temperature overnight.

The reaction was then quenched with water, and organic materials were extracted twice with CH_2Cl_2, washed first with 1M aqueous HCl solution followed by a second wash with saturated aqueous $NaHCO_3$ solution and brine. The combined organic fractions were dried over $MgSO_4$ and the solvent was removed in vacuo to obtain the *N,N*-dimethyladamantane-1-carboxamide in a 83% yield (587 mg).

^{1}H NMR (400 MHz, $CDCl_3$, 258 K) δ 3.05 (s, 6H), 2.02 (s, 3H), 1.98 (d, *J* = 2.9 Hz, 6H), 1.68 (d, *J* = 2.0 Hz, 7H).
^{13}C NMR (101 MHz, $CDCl_3$, 258 K) δ 177.05 (CO), 41.40 (C), 38.66 (N-CH_3), 38.57 (CH_2), 36.54 (CH_2), 28.32 (CH).
HRMS (ESI). Calculated for $C_{13}H_{22}NO^+$: 208.16959 Obtained: 208.16997

Preparation of 4,4-difluoro-N,N-dimethylcyclohexane-1-carboxamide (1n)

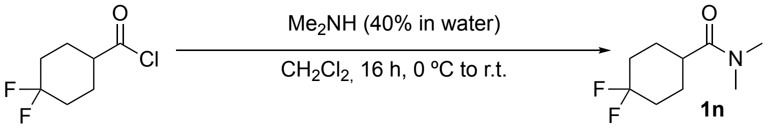

Prepared following an adapted procedure: To a solution of 4,4-difluorocyclohexane-1-carbonyl chloride (620 mg, 3.4 mmol, 1 equiv.) in CH_2Cl_2 (20 mL) at 0 °C, was added dropwise Me_2NH (40 w/w% in water; 1.7 mL, 13.6 mmol, 4 equiv.). The reaction mixture was stirred continuously at room temperature overnight.

The reaction was then quenched with water, and organic materials were extracted twice with CH_2Cl_2, washed first with 1M aqueous HCl solution followed by a second wash with saturated aqueous $NaHCO_3$ solution and brine. The combined organic fractions were dried over $MgSO_4$ and the solvent was removed in vacuo to obtain the 4,4-difluoro-*N,N*-dimethylcyclohexane-1-carboxamide in a 77% yield (503 mg).

^{1}H NMR (400 MHz, $CDCl_3$, 258 K) δ 3.05 (s, 3H), 2.93 (s, 3H), 2.61 – 2.52 (m, 1H), 2.23 – 2.10 (m, 2H), 1.88 – 1.63 (m, 6H).
^{13}C NMR (101 MHz, $CDCl_3$, 258 K) δ 174.21 (d, *J* = 2.0 Hz), 122.88 (t, *J* = 241.1 Hz), 38.14, 37.27, 35.66, 33.34 – 32.51 (m), 25.40 (d, *J* = 9.6 Hz).
^{19}F NMR (376 MHz, $CDCl_3$, 258 K) δ -92.31 (d, *J* = 236.4 Hz), -100.83 (d, *J* = 236.2 Hz).
HRMS (ESI). Calculated for $C_9H_{15}F_2NO^+$: 192.11945 Obtained: 192.11943

Preparation of 5-chloro-N,N-dimethylpentanamide (1o)

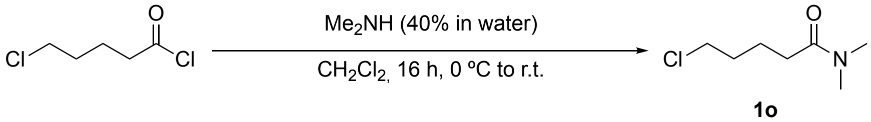

Prepared following an adapted procedure: To a solution of 5-chloropentanoyl chloride (0.32 mL, 2.5 mmol, 1 equiv.) in CH_2Cl_2 (20 mL) at 0 °C, was added dropwise

Me_2NH (40 w/w% in water; 1.3 mL, 10.0 mmol, 4 equiv.). The reaction mixture was stirred continuously at room temperature overnight.

The reaction was then quenched with water, and organic materials were extracted twice with CH_2Cl_2, washed first with 1M aqueous HCl solution followed by a second wash with saturated aqueous $NaHCO_3$ solution and brine. The combined organic fractions were dried over $MgSO_4$ and the solvent was removed in vacuo to obtain the 5-chloro-*N*,*N*-dimethylpentanamide in a 40% yield (164 mg).

^{1}H NMR (400 MHz, $CDCl_3$, 258 K) δ 3.55 (t, *J* = 6.3 Hz, 2H), 2.99 (s, 3H), 2.92 (s, 3H), 2.33 (t, *J* = 7.1 Hz, 2H), 1.86 – 1.69 (m, 4H).
^{13}C NMR (101 MHz, $CDCl_3$, 258 K) δ 172.49 (CO), 45.06 (CH_2), 37.34 (N-CH_3), 35.45 (N-CH_3), 32.44 (CH_2), 32.10 (CH_2), 22.32 (CH_2).
HRMS (ESI). Calculated for $C_7H_{14}ClNO^+$: 164.08367 Obtained: 164.08380

Preparation of N-isopropyl-N-methylcyclohexanecarboxamide (1p)

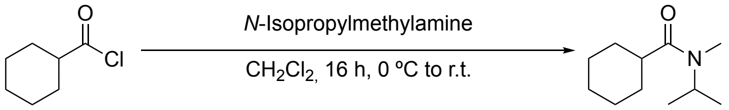

1p

Prepared following an adapted procedure: To a solution of cyclohexanecarbonyl chloride (0.70 mL, 5.0 mmol, 1 equiv.) in CH_2Cl_2 (20 mL) at 0 °C, was added dropwise *N*-Isopropylmethylamine (2.1 mL, 20.0 mmol, 4 equiv.). The reaction mixture was stirred continuously at room temperature overnight.

The reaction was then quenched with water, and organic materials were extracted twice with CH_2Cl_2, washed first with 1M aqueous HCl solution followed by a second wash with saturated aqueous $NaHCO_3$ solution and brine. The combined organic fractions were dried over $MgSO_4$ and the solvent was removed in vacuo to obtain the *N*-isopropyl-*N*-methylcyclohexanecarboxamide in a 79% yield (724 mg).

^{1}H NMR (400 MHz, $CDCl_3$, 258 K) δ 4.86 (p, *J* = 6.8 Hz, 0.5H), 4.09 (p, *J* = 6.6 Hz, 0.5H), 2.80 (s, 1.5H), 2.71 (s, 1.5H), 2.50 – 2.33 (m, 1H), 1.78 – 1.72 (m, 2H), 1.72 – 1.59 (m, 3H), 1.46 (td, *J* = 12.2, 3.9 Hz, 2H), 1.20 (d, *J* = 8.2 Hz, 3H), 1.15 (d, *J* = 6.6 Hz, 3H), 1.02 (d, *J* = 6.8 Hz, 3H).
^{13}C NMR (101 MHz, $CDCl_3$, 258 K) δ 175.62 (CO), 47.29 (N-CH), 43.32 (N-CH), 41.23 (CH), 40.72 (CH), 29.58 (CH_2), 28.97 (CH_2), 27.96 (N-CH_3), 26.06 (N-CH_3), 25.87 (CH_2), 25.85 (CH_2), 25.77 (CH_2), 20.81 (CH_3), 19.50 (CH_3).
HRMS (ESI). Calculated for $C_{11}H_{22}NO^+$: 184.1696 Obtained: 184.1691

Preparation of N-cyclopropyl-N-methylhexanamide (1q)

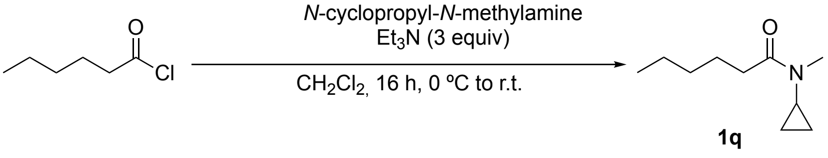

1q

Prepared following an adapted procedure: To a solution of hexanoyl chloride (0.21 mL, 1.5 mmol, 1 equiv.) in CH_2Cl_2 (20 mL) at 0 °C, were added *N*-cyclopropyl-

N-methylamine hydrochloride (1.6 g, 6.0 mmol, 4 equiv.) and triethylamine (0.8 mL, 6.0 mmol, 4 equiv.). The reaction mixture was stirred continuously at room temperature overnight.

The reaction was then quenched with water, and organic materials were extracted twice with CH_2Cl_2, washed first with 1M aqueous HCl solution followed by a second wash with saturated aqueous $NaHCO_3$ solution and brine. The combined organic fractions were dried over $MgSO_4$ and the solvent was removed in vacuo to obtain the *N*-cyclopropyl-*N*-methylhexanamide in a 55% yield (270 mg).

^{1}H NMR (400 MHz, $CDCl_3$, 258 K) δ 2.89 (s, 3H), 2.65 (dt, *J* = 7.1, 3.4 Hz, 1H), 2.49 (t, *J* = 7.7 Hz, 2H), 1.61 (p, *J* = 7.4 Hz, 2H), 1.29 (d, *J* = 3.7 Hz, 4H), 0.93 – 0.80 (m, 5H), 0.70 (dd, *J* = 4.1, 2.0 Hz, 2H).
^{13}C NMR (101 MHz, $CDCl_3$, 258 K) δ 176.51 (CO), 34.29 (N-CH_3), 34.08 (CH_2), 31.83 (CH_2), 31.29 (CH), 25.01 (CH_2), 22.72 (CH_2), 14.24 (CH_3), 9.25 (CH_2).
HRMS (ESI). Calculated for $C_{10}H_{20}NO^+$: 170.15394 Obtained: 170.15408

Preparation of N-methylcyclohexanecarboxamide (1s)

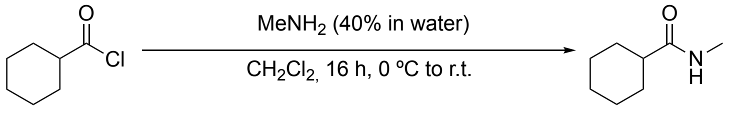

1s

Prepared following an adapted procedure: To a solution of cyclohexanecarbonyl chloride (0.67mL, 5.0 mmol, 1 equiv.) in CH_2Cl_2 (20 mL) at 0 °C, was added dropwise $MeNH_2$ (40 w/w% in water; 1.8 mL, 20.0 mmol, 4 equiv.). The reaction mixture was stirred continuously at room temperature overnight.

The reaction was then quenched with water, and organic materials were extracted twice with CH_2Cl_2, washed first with 1M aqueous HCl solution followed by a second wash with saturated aqueous $NaHCO_3$ solution and brine. The combined organic fractions were dried over $MgSO_4$ and the solvent was removed in vacuo to obtain the *N*-methylcyclohexanecarboxamide in a 40% yield (276 mg).

^{1}H NMR (400 MHz, $CDCl_3$, 258 K) δ 5.93 (s, 1H), 2.79 (d, *J* = 4.8 Hz, 3H), 2.08 (tt, *J* = 12.0, 3.4 Hz, 1H), 1.87 – 1.79 (m, 2H), 1.79 – 1.71 (m, 2H), 1.68 – 1.60 (m, 1H), 1.39 (qd, *J* = 12.1, 3.1 Hz, 2H), 1.28 – 1.13 (m, 3H).
^{13}C NMR (101 MHz, $CDCl_3$, 258 K) δ 177.30 (CO), 45.45 (N-CH_z), 29.60 (CH_2), 26.45 (CH), 25.71 (CH_2), 25.66 (CH_2).
HRMS (ESI). Calculated for $C_8H_{16}NO^+$: 142.1226 Obtained: 142.1229

Preparation of 1,1-diethyl-3,3-dimethylurea (1t)

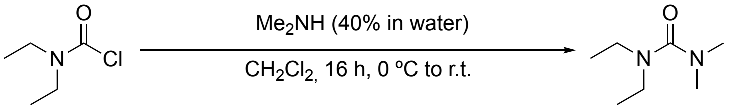

1t

Prepared following an adapted procedure: To a solution of diethylcarbamic chloride (0.43 mL, 3.4 mmol, 1 equiv.) in CH_2Cl_2 (20 mL) at 0 °C, were added

dropwise Me_2NH (40 w/w% in water; 0.6 mL, 5.1 mmol, 1.5 equiv.) and triethylamine (1.4 mL, 10.2 mmol, 3 equiv.). The reaction mixture was stirred continuously at room temperature overnight.

The reaction was then quenched with water, and organic materials were extracted twice with CH_2Cl_2, washed first with 1M aqueous HCl solution followed by a second wash with saturated aqueous $NaHCO_3$ solution and brine. The combined organic fractions were dried over $MgSO_4$ and the solvent was removed in vacuo to obtain the 1,1-diethyl-3,3-dimethylurea in a 55% yield (270 mg).

^{1}H NMR (400 MHz, $CDCl_3$, 300 K) δ 2.93 (q, *J* = 7.1 Hz, 4H), 2.56 (s, 6H), 0.87 (t, *J* = 7.1 Hz, 6H).
^{13}C NMR (101 MHz, $CDCl_3$, 300 K) δ 164.83 (CO), 41.62 (N-CH_2), 38.32 (N-CH_3), 12.86 (CH_3).
HRMS (ESI). Calculated for $C_7H_{17}N_2O^+$: 145.13354 Obtained: 145.13366

Preparation of N,N-diethylpropionamide (1u)

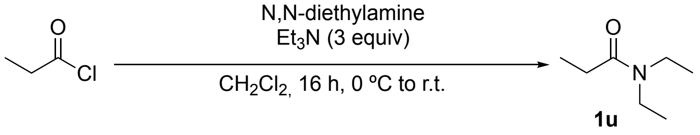

Prepared following an adapted procedure: To a solution of propionyl chloride (0.29 mL, 3.4 mmol, 1 equiv.) in CH_2Cl_2 (20 mL) at 0 °C, were added dropwise Et_2NH (0.5 mL, 5.1 mmol, 1.5 equiv.) and triethylamine (1.4 mL, 10.2 mmol, 3 equiv.). The reaction mixture was stirred continuously at room temperature overnight.

The reaction was then quenched with water, and organic materials were extracted twice with CH_2Cl_2, washed first with 1M aqueous HCl solution followed by a second wash with saturated aqueous $NaHCO_3$ solution and brine. The combined organic fractions were dried over $MgSO_4$ and the solvent was removed in vacuo to obtain the *N,N*-diethylpropionamide in a 66% yield (290 mg).

^{1}H NMR (400 MHz, $CDCl_3$, 258 K) δ 3.35 (q, *J* = 7.1 Hz, 2H), 3.28 (q, *J* = 7.1 Hz, 2H), 2.33 (q, *J* = 7.4 Hz, 2H), 1.18 – 1.07 (m, 9H).
^{13}C NMR (101 MHz, $CDCl_3$, 258 K) δ 173.09 (CO), 41.81 (N-CH_2), 40.05 (N-CH_2), 26.28 (CH_2), 14.29 (CH_3), 13.18 (CH_3), 9.78 (CH_3).
HRMS (ESI). Calculated for $C_7H_{16}NO^+$: 130.12264 Obtained: 130.12272

Preparation of N-methyl-N-phenylcyclohexanecarboxamide (1x)

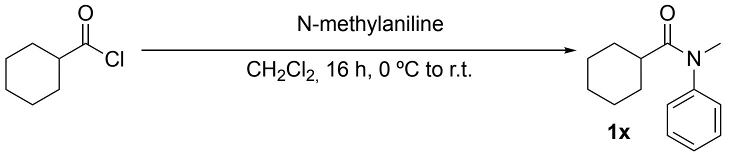

Prepared following an adapted procedure: To a solution of cyclohexanecarbonyl chloride (0.70 mL, 5.0 mmol, 1 equiv.) in CH_2Cl_2 (20 mL) at 0 °C, was added dropwise *N*-Methylaniline (2.2 mL, 20.0 mmol, 4 equiv.). The reaction mixture was stirred continuously at room temperature overnight.

The reaction was then quenched with water, and organic materials were extracted twice with CH_2Cl_2, washed first with 1M aqueous HCl solution followed by a second wash with saturated aqueous $NaHCO_3$ solution and brine. The combined organic fractions were dried over $MgSO_4$ and the solvent was removed in vacuo to obtain the *N-methyl-N*-phenylcyclohexanecarboxamide in a 89% yield (965 mg).

^{1}H NMR (400 MHz, $CDCl_3$, 258 K) δ 7.46 – 7.39 (m, 2H), 7.38 – 7.31 (m, 1H), 7.19 – 7.12 (m, 2H), 3.22 (s, 3H), 2.13 (tt, *J* = 11.9, 3.3 Hz, 1H), 1.62 (d, *J* = 10.1 Hz, 4H), 1.50 (td, *J* = 12.5, 2.9 Hz, 3H), 1.22 – 1.06 (m, 1H), 0.99 – 0.84 (m, 2H).
^{13}C NMR (101 MHz, $CDCl_3$, 258 K) δ 176.59 (CO), 143.94 (C, Ar), 129.81 (CH, Ar), 127.82 (CH, Ar), 127.21 (CH, Ar), 41.16 (CH), 37.51 (N-CH_3), 29.34 (CH_2), 25.49 (CH_2), 25.40 (CH_2).
HRMS (ESI). Calculated for $C_{14}H_{20}NO^+$: 218.1539 Obtained: 218.1539

Preparation of N-benzyl-N-methylcyclohexanecarboxamide (1y)

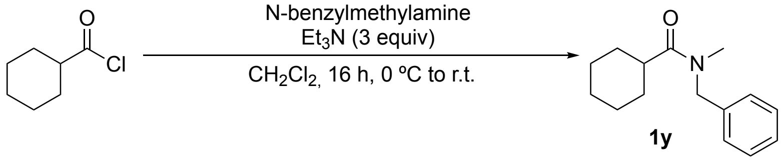

1y

Prepared following an adapted procedure: To a solution of cyclohexanecarbonyl chloride (0.45 mL, 3.4 mmol, 1 equiv.) in CH_2Cl_2 (5 mL) at 0 ºC, were added dropwise *N*-Benzylmethylamine (0.88 mL, 6.8 mmol, 2 equiv.) and triethylamine (0.71 mL, 5.1 mmol, 1.5 equiv.). The reaction mixture was stirred continuously at room temperature overnight.

The reaction was then quenched with water, and organic materials were extracted twice with CH_2Cl_2, washed first with 1M aqueous HCl solution followed by a second wash with saturated aqueous $NaHCO_3$ solution and brine. The combined organic fractions were dried over $MgSO_4$ and the solvent was removed in vacuo to obtain the *N*-benzyll-*N*-methylcyclohexanecarboxamide in a 87% yield (686 mg).

^{1}H NMR (400 MHz, $CDCl_3$, 258 K) δ 7.40 – 7.34 (m, 1H), 7.34 – 7.25 (m, 2H), 7.23 – 7.18 (m, 1H), 7.17 – 7.12 (m, 1H), 4.58 (s, 1H), 4.57 (s, 1H), 2.94 (s, 1.5H), 2.90 (s, 1.5H), 2.58 – 2.44 (m, 1H), 1.83 – 1.49 (m, 7H), 1.24 (dt, *J* = 23.3, 10.6 Hz, 3H).
^{13}C NMR (101 MHz, $CDCl_3$, 258 K) δ 176.94 (CO), 176.38 (CO), 137.51 (C, Ar), 136.79 (C, Ar), 128.94 (CH, Ar), 128.60 (CH, Ar), 127.84 (CH, Ar), 127.58 (CH, Ar), 127.25 (CH, Ar), 126.18 (CH, Ar), 52.92 (CH_2), 50.52 (CH_2), 40.68 (CH), 40.55 (CH), 34.69 (N-CH_3), 33.90 (N-CH_3), 29.57 (CH_2), 29.10 (CH_2), 25.83 (CH_2), 25.78 (CH_2), 25.72 (CH_2), 25.66 (CH_2).
HRMS (ESI). Calculated for $C_{15}H_{22}NO^+$: 232.16959 Obtained: 232.16981

Preparation of N,N-dimethyl-2-phenylacetamide (1z)

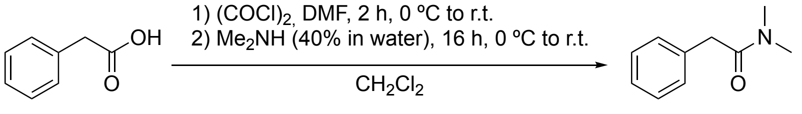

1z

Prepared following an adapted procedure: To a solution of 2-phenylacetic acid (680.7 mg, 5.0 mmol, 1 equiv.) in CH_2Cl_2 (20 mL) at room temperature, were

added carefully $(COCl)_2$ (0.64 mL, 7.5 mmol, 1.5 equiv.) and DMF (5 drops) in that order. The reaction mixture was stirred at room temperature for 2 h. After that time, the mixture was cooled down to 0 ºC and Me_2NH (40 w/w% in water; 2.5 mL, 20.0 mmol, 4 equiv.) was added dropwise. The reaction mixture was stirred continuously at room temperature overnight.

The reaction was then quenched with water, and organic materials were extracted twice with CH_2Cl_2, washed first with 1M aqueous HCl solution followed by a second wash with saturated aqueous $NaHCO_3$ solution and brine. The combined organic fractions were dried over $MgSO_4$ and the solvent was removed in vacuo to obtain the *N,N*-dimethyl-2-phenylacetamide in a 94% yield (767 mg).

^{1}H NMR (400 MHz, $CDCl_3$, 258 K) δ 7.35 – 7.29 (m, 2H), 7.27 – 7.22 (m, 3H), 3.73 (s, 2H), 2.99 (s, 3H), 2.96 (s, 3H).
^{13}C NMR (101 MHz, $CDCl_3$, 258 K) δ 171.21 (CO), 134.84 (C, Ar), 128.73 (CH, Ar), 128.70 (CH, Ar), 126.77 (CH, Ar), 40.98 (CH_2), 37.82 (N-CH_3), 35.69 (N-CH_3).
HRMS (ESI). Calculated for $C_{10}H_{14}NO^+$: 164. 1070 Obtained: 164. 1070

Preparation of (1aa)

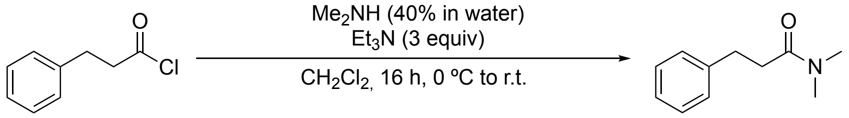

1aa

Prepared following an adapted procedure: To a solution of 3-phenylpropanoyl chloride (0.5 mL, 3.4 mmol, 1 equiv.) in CH_2Cl_2 (20 mL) at 0 ºC, were added dropwise Me_2NH (40 w/w% in water; 0.9 mL, 6.8 mmol, 2 equiv.) and triethylamine (1.4 mL, 10.2 mmol, 3 equiv.). The reaction mixture was stirred continuously at room temperature overnight.

The reaction was then quenched with water, and organic materials were extracted twice with CH_2Cl_2, washed first with 1M aqueous HCl solution followed by a second wash with saturated aqueous $NaHCO_3$ solution and brine. The combined organic fractions were dried over $MgSO_4$ and the solvent was removed in vacuo to obtain the *N*,*N*-dimethyl-3-phenylpropanamide in a 98% yield (595 mg).

^{1}H NMR (400 MHz, $CDCl_3$, 258 K) δ 7.33 – 7.27 (m, 2H), 7.25 – 7.19 (m, 3H), 2.94 (d, *J* = 8.9 Hz, 8H), 2.64 – 2.58 (m, 2H).
^{13}C NMR (101 MHz, $CDCl_3$, 258 K) δ 172.21 (CO), 141.39 (C, Ar), 128.49 (CH, Ar), 128.46 (CH, Ar), 126.12 (CH, Ar), 37.21 (CH_2), 35.51 (N-CH_3), 35.46 (CH_2), 31.28 (N-CH_3).
HRMS (ESI). Calculated for $C_{11}H_{16}NO^+$: 178.122641 Obtained: 178.122724

Preparation of (1ab)

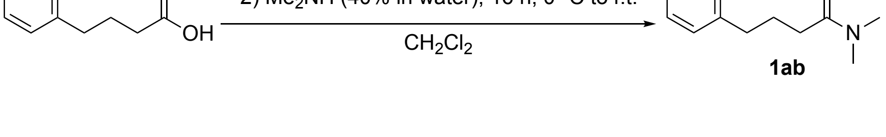

1ab

Prepared following an adapted procedure: To a solution of 4-phenylbutanoic acid (1.0 g, 6.0 mmol, 1 equiv.) in CH_2Cl_2 (25 mL) at room temperature, were added carefully $(COCl)_2$ (0.78 mL, 9.1 mmol, 1.5 equiv.) and DMF (5 drops) in that order. The reaction mixture was stirred at room temperature for 2 h. After that time, the mixture was cooled down to 0 ºC and Me_2NH (40 w/w% in water; 3.1 mL, 24.4 mmol, 4 equiv.) was added dropwise. The reaction mixture was stirred continuously at room temperature overnight.

The reaction was then quenched with water, and organic materials were extracted twice with CH_2Cl_2, washed first with 1M aqueous HCl solution followed by a second wash with saturated aqueous $NaHCO_3$ solution and brine. The combined organic fractions were dried over $MgSO_4$ and the solvent was removed in vacuo to obtain the *N,N*-dimethyl-4-phenylbutanamide in a 89% yield (1 g).

^{1}H NMR (400 MHz, $CDCl_3$, 258 K) δ 7.33 – 7.26 (m, 2H), 7.22 – 7.16 (m, 3H), 2.93 (s, 6H), 2.66 (t, *J* = 7.6 Hz, 2H), 2.34 – 2.28 (m, 2H), 1.95 (ddd, *J* = 15.2, 8.2, 6.9 Hz, 2H).
^{13}C NMR (101 MHz, $CDCl_3$, 258 K) δ 172.84 (CO), 141.77 (C, Ar), 128.50 (CH, Ar), 128.34 (CH, Ar), 125.85 (CH, Ar), 37.27 (N-CH_3), 35.38 (N-CH_3), 35.26 (CH_2), 32.49 (CH_2), 26.57 (CH_2).
HRMS (ESI). Calculated for $C_{12}H_{17}NNaO^+$: 214.120235 Obtained: 214.120347

Preparation of 3-(2-bromophenyl)-N,N-dimethylpropanamide (1ac)

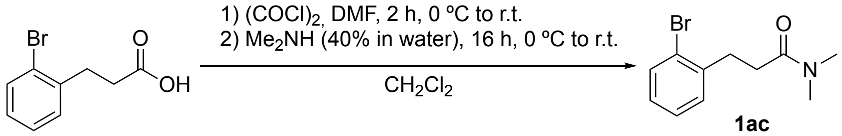

1ac

Prepared following an adapted procedure: To a solution of 3-(2-bromophenyl)propanoic acid (572 mg, 2.5 mmol, 1 equiv.) in CH_2Cl_2 (20 mL) at room temperature, were added carefully $(COCl)_2$ (0.32 mL, 3.75 mmol, 1.5 equiv.) and DMF (5 drops) in that order. The reaction mixture was stirred at room temperature for 2 h. After that time, the mixture was cooled down to 0 ºC and Me_2NH (40 w/w% in water; 1.3 mL, 10.0 mmol, 4 equiv.) was added dropwise. The reaction mixture was stirred continuously at room temperature overnight.

The reaction was then quenched with water, and organic materials were extracted twice with CH_2Cl_2, washed first with 1M aqueous HCl solution followed by a second wash with saturated aqueous $NaHCO_3$ solution and brine. The combined organic fractions were dried over $MgSO_4$ and the solvent was removed in vacuo to obtain the 3-(2-bromophenyl)-*N,N*-dimethylpropanamide in a 80% yield (515 mg).

^{1}H NMR (400 MHz, $CDCl_3$, 258 K) δ 7.56 (d, *J* = 7.9 Hz, 1H), 7.30 (dt, *J* = 14.9, 7.5 Hz, 2H), 7.12 (t, *J* = 7.6 Hz, 1H), 3.09 (t, *J* = 8.1 Hz, 2H), 2.99 (d, *J* = 1.6 Hz, 6H), 2.65 (t, *J* = 8.1 Hz, 2H).
^{13}C NMR (101 MHz, $CDCl_3$, 258 K) δ 172.05 (CO), 140.56 (C, Ar), 132.78 (CH, Ar), 130.98 (CH, Ar), 128.10 (CH, Ar), 127.74 (CH, Ar), 124.31 (C, Ar), 37.35 (N-CH_3), 35.56 (N-CH_3), 33.52 (CH_2), 32.03 (CH_2).
HRMS (ESI). Calculated for $C_{11}H_{14}BrNONa^+$: 278.01510 Obtained: 278.01516

Preparation of N,N-dimethyl-2-phenylbutanamide (1ad)

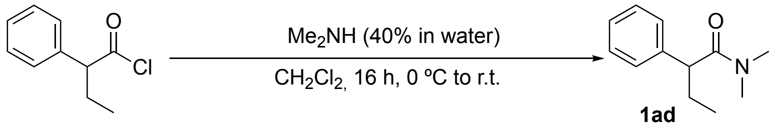

1ad

Prepared following an adapted procedure: To a solution of 2-phenylbutanoyl chloride (0.57 mL, 3.4 mmol, 1 equiv.) in CH_2Cl_2 (20 mL) at 0 °C, were added dropwise Me_2NH (40 w/w% in water; 0.6 mL, 5.1 mmol, 1.5 equiv.) and triethylamine (1.4 mL, 10.2 mmol, 3 equiv.). The reaction mixture was stirred continuously at room temperature overnight.

The reaction was then quenched with water, and organic materials were extracted twice with CH_2Cl_2, washed first with 1M aqueous HCl solution followed by a second wash with saturated aqueous $NaHCO_3$ solution and brine. The combined organic fractions were dried over $MgSO_4$ and the solvent was removed in vacuo to obtain the *N,N*-dimethyl-2-phenylbutanamide in a 76% yield (495 mg).

^{1}H NMR (400 MHz, $CDCl_3$, 258 K) δ 7.34 – 7.27 (m, 4H), 7.24 (m, 1H), 3.58 (t, *J* = 7.3 Hz, 1H), 2.94 (s, 6H), 2.14 – 2.00 (m, 1H), 1.78 – 1.65 (m, 1H), 0.84 (t, *J* = 7.4 Hz, 3H).
^{13}C NMR (101 MHz, $CDCl_3$, 258 K) δ 173.25 (CO), 140.01 (C, Ar), 128.74 (CH, Ar), 127.99 (CH, Ar), 126.88 (CH, Ar), 50.59 (CH), 37.28 (N-CH_3), 35.96 (N-CH_3), 28.17 (CH_2), 12.62 (CH_3).
HRMS (ESI). Calculated for $C_{12}H_{18}NO^+$: 192.13829 Obtained: 192.13860

Preparation of (1R,2R)-N,N-dimethyl-2-phenylcyclopropane-1-carboxamide (1ae)

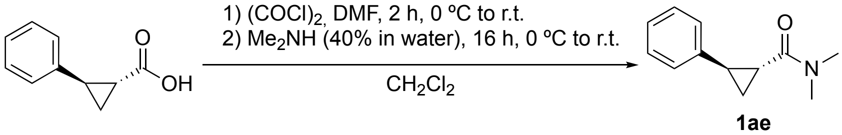

1ae

Prepared following an adapted procedure: To a solution of (1*R*,2*R*)-2-phenylcyclopropane-1-carboxylic acid (500 mg, 3.0 mmol, 1 equiv.) in CH_2Cl_2 (20 mL) at room temperature, were added carefully $(COCl)_2$ (0.39 mL, 4.6 mmol, 1.5 equiv.) and DMF (5 drops) in that order. The reaction mixture was stirred at room temperature for 2 h. After that time, the mixture was cooled down to 0 °C and Me_2NH (40 w/w% in water; 1.6 mL, 12.3 mmol, 4 equiv.) was added dropwise. The reaction mixture was stirred continuously at room temperature overnight.

The reaction was then quenched with water, and organic materials were extracted twice with CH_2Cl_2, washed first with 1M aqueous HCl solution followed by a second wash with saturated aqueous $NaHCO_3$ solution and brine. The combined organic fractions were dried over $MgSO_4$ and the solvent was removed in vacuo to obtain the *N,N*-dimethyl-2-phenylacetamide in a 91% yield (530 mg).

^{1}H NMR (400 MHz, $CDCl_3$, 258 K) δ 7.32 – 7.27 (m, 2H), 7.25 – 7.16 (m, 1H), 7.14 – 7.09 (m, 2H), 3.12 (s, 3H), 3.00 (s, 2H), 2.47 (ddd, *J* = 9.0, 6.3, 4.1 Hz, 1H), 2.00 (ddd, *J* = 8.2, 5.3, 4.2 Hz, 1H), 1.65 (ddd, *J* = 9.0, 5.3, 4.3 Hz, 1H), 1.29 (ddd, *J* = 8.2, 6.3, 4.3 Hz, 1H).

^{13}C NMR (101 MHz, $CDCl_3$, 258 K) δ 172.04 (CO), 141.08 (C, Ar), 128.51 (CH, Ar), 126.25 (CH, Ar), 125.98 (CH, Ar), 37.50 (N-CH_3), 36.05 (N-CH_3), 25.69 (CH), 23.39 (CH), 16.72 (CH_2).
HRMS (ESI). Calculated for $C_{12}H_{16}NO^+$: 190.12264 Obtained: 190.12265

Preparation of 2-(1H-indol-3-yl)-N,N-dimethylacetamide (1af)

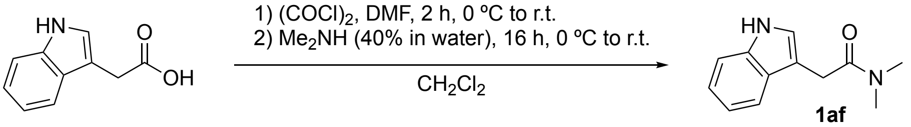

Prepared following an adapted procedure: To a solution of Indole-3-acetic acid (875 mg, 5.0 mmol, 1 equiv.) in CH_2Cl_2 (10 mL) at room temperature, were added carefully $(COCl)_2$ (0.64 mL, 7.5 mmol, 1.5 equiv.) and DMF (5 drops) in that order. The reaction mixture was stirred at room temperature for 2 h. After that time, the mixture was cooled down to 0 °C and Me_2NH (40 w/w% in water; 2.5 mL, 20.0 mmol, 4 equiv.) was added dropwise. The reaction mixture was stirred continuously at room temperature overnight.

The reaction was then quenched with water, and organic materials were extracted twice with CH_2Cl_2, washed with saturated aqueous $NaHCO_3$ solution and brine. The combined organic fractions were dried over $MgSO_4$, the solvent was removed in vacuo and the mixture was purified by column chromatography on silica gel (Hex/EtOAc 40%) to obtain 2-(1H-indol-3-yl)-N,N-dimethylacetamide (700 mg, 69% yield) as a off-white solid.

^{1}H NMR (400 MHz, $CDCl_3$, 258 K) δ 8.53 (s, 1H), 7.63 (ddd, J = 7.8, 1.3, 0.7 Hz, 1H), 7.36 – 7.29 (m, 1H), 7.18 (ddd, J = 8.2, 7.0, 1.3 Hz, 1H), 7.11 (ddd, J = 8.1, 7.0, 1.1 Hz, 1H), 7.03 – 6.99 (m, 1H), 3.82 (d, J = 1.0 Hz, 2H), 3.02 (s, 3H), 2.98 (s, 3H).
^{13}C NMR (101 MHz, $CDCl_3$, 258 K) δ 171.89 (CO), 136.34 (C, Ar), 127.32 (C, Ar), 122.81 (CH, Ar), 122.12 (CH, Ar), 119.56 (CH, Ar), 118.81 (CH, Ar), 111.39 (CH, Ar), 109.12 (C, Ar), 37.97 (N-CH_3), 35.80 (N-CH_3), 31.36 (CH_2).
HRMS (ESI). Calculated for $C_{12}H_{15}N_2O^+$: 203.117890 Obtained: 203.117946

Preparation of N-methyl-N-(pyridin-2-yl)hexanamide (1ag)

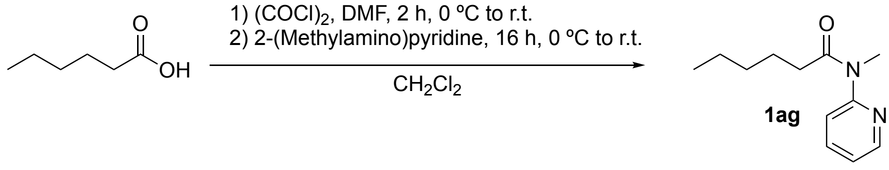

Prepared following an adapted procedure: To a solution of hexanoic acid (875 mg, 5.0 mmol, 1 equiv.) in CH_2Cl_2 (10 mL) at room temperature, were added carefully $(COCl)_2$ (0.64 mL, 7.5 mmol, 1.5 equiv.) and DMF (5 drops) in that order. The reaction mixture was stirred at room temperature for 2 h. After that time, the mixture was cooled down to 0 °C and Me_2NH (40 w/w% in water; 2.5 mL, 20.0 mmol, 4 equiv.) was added dropwise. The reaction mixture was stirred continuously at room temperature overnight.

The reaction was then quenched with water, and organic materials were extracted twice with CH_2Cl_2, washed with saturated aqueous $NaHCO_3$ solution and brine. The combined organic fractions were dried over $MgSO_4$, the solvent was removed in vacuo and the mixture was purified by column chromatography on silica gel (Hex/EtOAc 40%) to obtain N-methyl-N-(pyridin-2-yl)hexanamide (343 mg, 33% yield) as a clear oil.

^{1}H NMR (400 MHz, $CDCl_3$, 258 K) δ 8.50 (ddd, *J* = 4.9, 2.0, 0.8 Hz, 1H), 7.75 (ddd, *J* = 8.1, 7.4, 2.0 Hz, 1H), 7.30 (d, *J* = 8.1 Hz, 1H), 7.20 (ddd, *J* = 7.4, 4.9, 1.0 Hz, 1H), 3.37 (s, 3H), 2.32 – 2.24 (m, 2H), 1.65 – 1.58 (m, 2H), 1.28 – 1.17 (m, 4H), 0.87 – 0.81 (m, 3H).
^{13}C NMR (101 MHz, $CDCl_3$, 258 K) δ 173.72 (CO), 156.32 (C, Ar), 148.94 (CH, Ar), 138.43 (CH, Ar), 121.88 (CH, Ar), 121.05 (CH, Ar), 35.63 (N-CH_3), 34.94 (CH_2), 31.62 (CH_2), 25.16 (CH_2), 22.53 (CH_2), 14.04 (CH_3).
HRMS (ESI). Calculated for $C_{12}H_{18}N_2NaO^+$: 229.131134 Obtained: 229.131080

Preparation of (1S)-N,N,4,7,7-pentamethyl-3-oxo-2-oxabicyclo[2.2.1]heptane-1-carboxamide (1ah)

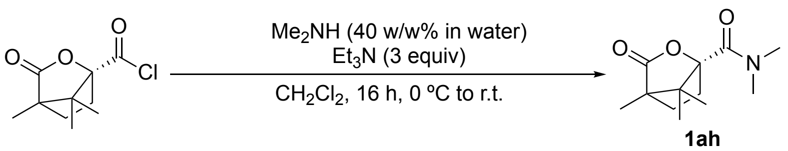

1ah

Prepared following an adapted procedure: To a solution of (1S)-4,7,7-trimethyl-3-oxo-2-oxabicyclo[2.2.1]heptane-1-carbonyl chloride (500 mg, 2.3 mmol, 1 equiv.) in CH_2Cl_2 (20 mL) at 0 °C, were added dropwise Me_2NH (40 w/w% in water; 1.2 mL, 9.2 mmol, 4 equiv.) and triethylamine (0.9 mL, 6.9 mmol, 3 equiv.). The reaction mixture was stirred continuously at room temperature overnight.

The reaction was then quenched with water, and organic materials were extracted twice with CH_2Cl_2, washed first with 1M aqueous HCl solution followed by a second wash with saturated aqueous $NaHCO_3$ solution and brine. The combined organic fractions were dried over $MgSO_4$ and the solvent was removed in vacuo to obtain the (1*S*)-*N*,*N*,4,7,7-pentamethyl-3-oxo-2-oxabicyclo[2.2.1]heptane-1-carboxamide in a 37% yield (191 mg).

^{1}H NMR (400 MHz, $CDCl_3$, 258 K) δ 3.19 (s, 3H), 2.97 (s, 3H), 2.36 (ddd, *J* = 13.6, 10.8, 4.3 Hz, 1H), 2.06 (ddd, *J* = 13.7, 9.3, 4.5 Hz, 1H), 1.91 (ddd, *J* = 13.1, 10.9, 4.5 Hz, 1H), 1.69 (ddd, *J* = 13.4, 9.3, 4.3 Hz, 1H), 1.17 – 1.13 (m, 3H), 1.08 (s, 3H), 0.99 (s, 3H).
^{13}C NMR (101 MHz, $CDCl_3$, 258 K) δ 179.28 (CO), 166.79 (CO), 92.60 (C), 55.18 (C), 53.81 (C), 38.09 (N-CH_3), 37.02 (N-CH_3), 30.88 (CH_2), 29.17 (CH_2), 17.80 (CH_3), 16.84 (CH_3), 9.77 (CH_3).
HRMS (ESI). Calculated for $C_{12}H_{20}NO_3^+$: 226.14377 Obtained: 226.14372

Preparation of 5-(2,5-dimethylphenoxy)-N,N,2,2-tetramethylpentanamide (1ai)

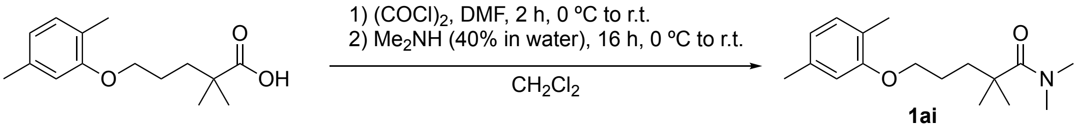

Prepared following an adapted procedure: To a solution of Gemfibrozil (625 mg, 2.5 mmol, 1 equiv.) in CH_2Cl_2 (20 mL) at room temperature, were added carefully $(COCl)_2$ (0.32 mL, 3.75 mmol, 1.5 equiv.) and DMF (5 drops) in that order. The reaction mixture was stirred at room temperature for 2 h. After that time, the mixture was cooled down to 0 ºC and Me_2NH (40 w/w% in water; 1.3 mL, 10.0 mmol, 4 equiv.) was added dropwise. The reaction mixture was stirred continuously at room temperature overnight.

The reaction was then quenched with water, and organic materials were extracted twice with CH_2Cl_2, washed first with 1M aqueous HCl solution followed by a second wash with saturated aqueous $NaHCO_3$ solution and brine. The combined organic fractions were dried over $MgSO_4$ and the solvent was removed in vacuo to obtain the 5-(2,5-dimethylphenoxy)-*N*,*N*,2,2-tetramethylpentanamide in a 40% yield (277 mg).

^{1}H NMR (400 MHz, $CDCl_3$, 258 K) δ 7.01 (d, *J* = 7.4 Hz, 1H), 6.66 (d, *J* = 7.5 Hz, 1H), 6.60 (s, 1H), 3.92 (t, *J* = 5.7 Hz, 2H), 3.15 (s, 3H), 2.96 (s, 3H), 2.30 (s, 3H), 2.15 (s, 3H), 1.86 – 1.68 (m, 4H), 1.30 (s, 6H).
^{13}C NMR (101 MHz, $CDCl_3$, 258 K) δ 176.63 (CO), 156.75 (C, Ar), 136.66 (C, Ar), 130.27 (CH, Ar), 123.17 (C, Ar), 120.50 (CH, Ar), 111.44 (CH, Ar), 67.44 (CH_2), 42.36 (C), 38.28 (N-CH_3), 37.01 (CH_2), 27.06 (CH_3), 25.24 (CH_2), 21.61 (CH_3), 16.15 (CH_3).
HRMS (ESI). Calculated for $C_{17}H_{28}NO_2^+$: 278.21146 Obtained: 278.21189

Preparation of 4-(4-(bis(2-chloroethyl)amino)phenyl)-N,N-dimethylbutanamide (1aj)

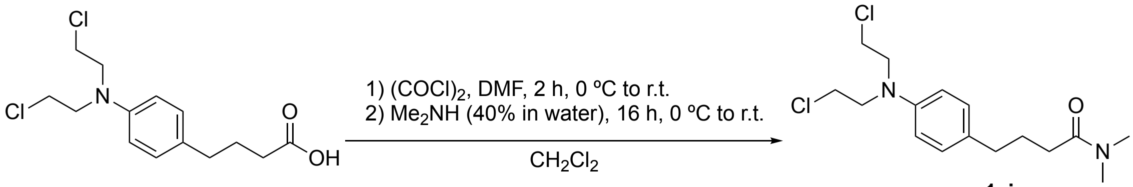

Prepared following an adapted procedure: To a solution of Chlorambucil (304 mg, 1.0 mmol, 1 equiv.) in CH_2Cl_2 (20 mL) at room temperature, were added carefully $(COCl)_2$ (0.13 mL, 1.5 mmol, 1.5 equiv.) and DMF (5 drops) in that order. The reaction mixture was stirred at room temperature for 2 h. After that time, the mixture was cooled down to 0 ºC and Me_2NH (40 w/w% in water; 0.5 mL, 4.0 mmol, 4 equiv.) was added dropwise. The reaction mixture was stirred continuously at room temperature overnight.

The reaction was then quenched with water, and organic materials were extracted twice with CH_2Cl_2, washed first with 1M aqueous HCl solution followed by a second wash with saturated aqueous $NaHCO_3$ solution and brine. The combined organic fractions were dried over $MgSO_4$ and the solvent was removed in vacuo to obtain the 4-(4-(bis(2-chloroethyl)amino)phenyl)-*N*,*N*-dimethylbutanamide in a 61% yield (204 mg).

^{1}H NMR (400 MHz, $CDCl_3$, 258 K) δ 7.16 – 7.03 (m, 2H), 6.62 (s, 2H), 3.69 (d, *J* = 6.7 Hz, 4H), 3.66 – 3.56 (m, 4H), 2.97 (d, *J* = 1.1 Hz, 3H), 2.94 (s, 3H), 2.58 (t, *J* = 7.6 Hz, 2H), 2.32 (t, *J* = 7.6 Hz, 2H), 1.90 (p, *J* = 7.6 Hz, 2H).
^{13}C NMR (101 MHz, $CDCl_3$, 258 K) δ 172.98 (CO), 129.86 (CH, Ar), 128.21 (C, Ar), 111.56 (C, Ar), 53.43 (CH_2), 40.39 (CH_2), 37.41 (N-CH_3), 35.49 (N-CH_3), 34.25 (CH_2), 32.65 (CH_2), 26.88 (CH_2).
HRMS (ESI). Calculated for $C_{16}H_{25}Cl_2N_2O^+$: 331.13385 Obtained: 331.13397

Preparation of N-methyl-N-(3-phenyl-3-(4-(trifluoromethyl)phenoxy)propyl)hexanamide (1ak)

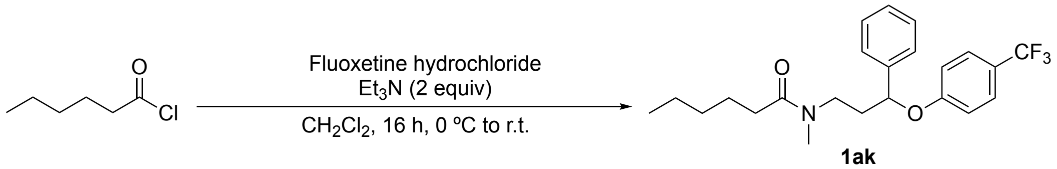

Prepared following an adapted procedure: To a solution of hexanoyl chloride (0.14 mL, 1.0 mmol, 1 equiv.) in CH_2Cl_2 (5 mL) at 0 °C, were added Fluoxetine hydrochloride (345 mg, 1.0 mmol, 1 equiv.) and triethylamine (0.3 mL, 2.0 mmol, 2 equiv.). The reaction mixture was stirred continuously at room temperature overnight.

The reaction was then quenched with water, and organic materials were extracted twice with CH_2Cl_2, washed first with 1M aqueous HCl solution followed by a second wash with saturated aqueous $NaHCO_3$ solution and brine. The combined organic fractions were dried over $MgSO_4$ and the solvent was removed in vacuo to obtain the *N*-methyl-*N*-(3-phenyl-3-(4-(trifluoromethyl)phenoxy)propyl)hexanamide in a 55% yield (216 mg).

^{1}H NMR (400 MHz, DMSO, 398 K) δ 7.53 (d, *J* = 8.5 Hz, 2H), 7.42 (d, *J* = 8.0 Hz, 2H), 7.36 (t, *J* = 7.6 Hz, 2H), 7.28 (t, *J* = 7.2 Hz, 1H), 7.07 (d, *J* = 8.5 Hz, 2H), 5.43 (dd, *J* = 7.8, 4.8 Hz, 1H), 3.46 (td, *J* = 6.9, 2.0 Hz, 2H), 2.88 (s, 3H), 2.27 – 2.15 (m, 3H), 2.15 – 2.04 (m, 1H), 1.49 (p, *J* = 7.2 Hz, 2H), 1.35 – 1.17 (m, 4H), 0.86 (t, *J* = 6.7 Hz, 3H).
^{13}C NMR (101 MHz, DMSO, 398 K) δ 171.31 (CO), 160.04 (C, Ar), 140.00 (C, Ar), 127.80 (CH, Ar), 127.02 (CH, Ar), 125.94 (q, *J* = 3.9 Hz, CH, Ar), 125.36 (CH, Ar), 123.81 (q, *J* = 270.4 Hz, CF_3), 121.28 (q, *J* = 32.2 Hz, C, Ar), 115.79 (CH, Ar), 77.50 (CH), 44.14 (CH_2), 35.31 (CH_2), 33.29 (N-CH_3), 31.77 (CH_2), 30.32 (CH_2), 23.68 (CH_2), 21.02 (CH_2), 12.68 (CH_3).
^{19}F NMR (376 MHz, $CDCl_3$, 298 K) δ -61.52, -61.61.
HRMS (ESI). Calculated for $C_{23}H_{29}F_3NO_2^+$: 408.21449 Obtained: 408.21456

Synthesis of bipyridine ligands

Preparation of 5-(trifluoromethyl)-2,2'-bipyridine (L2)

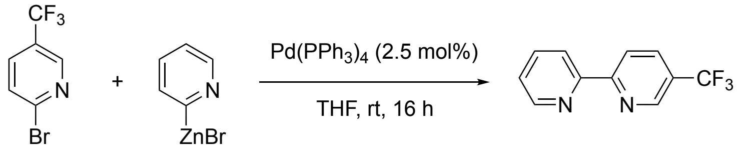

Prepared following an adapted procedure: Under a nitrogen atmosphere, $Pd(PPh_3)_4$ (134.6 mg, 0.1 mmol, 2.5 mol%) and 2-bromo-5(trifluoromethyl)pyridine (1 g, 4.4 mmol, 1 eq.) were added to a dry two-neck round-bottom flask and cooled to 0 ºC. Upon addition of 2-pyridylzinc bromide (10.62 ml, 5.3 mmol, 1.2 eq) in THF (0.5M) the reaction mixture turned brown in colour. The mixture was stirred overnight at r.t. under an inert atmosphere during which time a white precipitate formed.

Subsequently, the reaction mixture was poured into saturated aqueous solution of 1:1 EDTA/Na_2CO_3 (500 mL), and the mixture was stirred until the white solid turned completely yellow. The aqueous suspension was extracted with CH_2Cl_2 (3 x 100 mL), the combined organic layers were dried over anhydrous $MgSO_4$ and the solvent was evaporated under reduced pressure. The mixture was purified by column chromatography on silica gel (Hex/Et_2O 5%) to afford 5-trifluoro-2,2'-bipyridine (807 mg, 81% yield) as a white solid.

^{1}H NMR (400 MHz, $CDCl_3$) δ 8.93 (dt, *J* = 2.4, 0.9 Hz, 1H), 8.71 (ddd, *J* = 4.8, 1.8, 0.9 Hz, 1H), 8.58 (dt, *J* = 8.4, 0.8 Hz, 1H), 8.47 (dt, *J* = 8.0, 1.1 Hz, 1H), 8.05 (ddq, *J* = 8.4, 2.2, 0.7 Hz, 1H), 7.87 (ddd, *J* = 8.0, 7.5, 1.8 Hz, 1H), 7.38 (ddd, *J* = 7.5, 4.8, 1.2 Hz, 1H).
^{13}C NMR (101 MHz, $CDCl_3$) δ 159.08 (C, Ar), 154.65 (C, Ar), 149.31 (CH, Ar), 146.28 (q, *J* = 4.1 Hz, CH, Ar), 137.53 (CH, Ar), 134.25 (q, *J* = 3.5 Hz, CH, Ar), 126.50 (q, *J* = 33.1 Hz, C, Ar), 124.82 (CH, Ar), 123.81 (q, *J* = 272.5 Hz, CF_3), 121.96 (CH, Ar), 120.90 (CH, Ar).
^{19}F NMR (376 MHz, $CDCl_3$) δ -62.35.
HRMS (ESI). Calculated for $C_{11}H_8F_3N_2^+$: 225.0634 Obtained: 225.0632

Preparation of 5-methoxy-2,2'-bipyridine (L3)

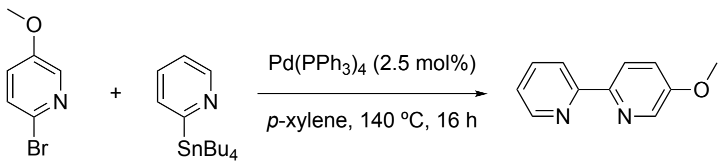

Prepared following an adapted procedure: 2-(tributylstannyl)pyridine (0.52 mL, 1.38 mmol, 1.3 eq.), 2-Bromo-5-methoxypyridine (200 mg, 1.1 mmol, 1.0 eq.) and $Pd(PPh_3)_4$ (122.5 mg, 0.1 mmol, 10 mol%) were sequentially added to degassed p-xylene (33 mL, 0.03 M). The mixture was stirred for 16 h at 140 °C under an argon atmosphere.

Then, the solution was poured onto 2.0 M aq. KOH (50 mL) and vigorously stirred for 15 min. The phases were separated, and the aqueous phase extracted with toluene. The combined organic layers were washed with 0.5 M EDTA solution

(pH=7), dried, filtrated and evaporated. The mixture was purified by column chromatography on silica gel (Hex/Et_2O 5% to Hex/Et_2O 30%) to afford 5-methoxy-2,2'-bipyridine (168 mg, 85% yield) as a colorless oil.

^{1}H NMR (400 MHz, $CDCl_3$) δ 8.61 (ddd, *J* = 4.8, 1.8, 0.9 Hz, 1H), 8.36 – 8.27 (m, 3H), 7.75 (ddd, *J* = 8.0, 7.5, 1.8 Hz, 1H), 7.28 (dd, *J* = 8.7, 3.0 Hz, 1H), 7.22 (ddd, *J* = 7.5, 4.9, 1.2 Hz, 1H), 3.87 (s, 3H).
^{13}C NMR (101 MHz, $CDCl_3$) δ 156.18 (C, Ar), 155.97 (C, Ar), 148.96 (CH, Ar), 148.80 (C, Ar), 137.05 (CH, Ar), 137.04 (CH, Ar), 123.01 (CH, Ar), 121.82 (CH, Ar), 120.97 (CH, Ar), 120.54 (CH, Ar), 55.73 (O-CH_3).
HRMS (ESI). Calculated for $C_{11}H_{11}N_2O^+$: 187.0866 Obtained: 187.0866

Preparation of 5,5'-bis(trifluoromethyl)-2,2'-bipyridine (L4)

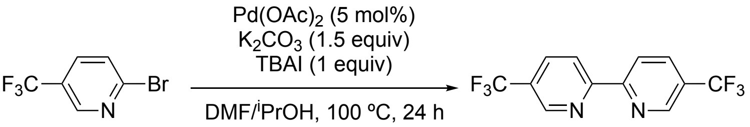

Prepared following an adapted procedure: To a round-bottom flask charged with a magnetic stir bar were added, $Pd(OAc)_2$ (99.3 mg, 0.4 mmol, 5 mol%), TBAI (3.3 g, 8.8 mmol, 1 equiv.) and K_2CO_3 (1.8 g, 1.3 mmol, 1.5 eq.). The flask was then evacuated and backfilled with N_2 three times. To this flask were added 2-bromo-5-(trifluoromethyl)pyridine (2 g, 8.8 mmol, 1 equiv.), ^{i}PrOH (1.5 mL, 6.5 M) and DMF (30 mL, 0.3 M) and the reaction was heated at 100 °C for 24 h.

The reaction mixture was then cooled to room temperature and filtered, washing copiously with EtOAc. The organics were washed three times with 40 mL of 10% (w/w) aqueous LiCl. The aqueous layer was backwashed once with EtOAc (50 mL) and the combined organics washed with water (40 mL) and brine (40 mL) before drying over $MgSO_4$. Following removal of the solvent in vacuo, the crude residue was purified by silica gel chromatography (gradient from 100% hexane to 5% EtOAc/hexane) to afford 5,5'-bis(trifluoromethyl)-2,2'-bipyridine (1.2 g, 99% yield) as a white solid.

^{1}H NMR (400 MHz, $CDCl_3$) δ 8.96 (s, 2H), 8.62 (d, *J* = 8.3 Hz, 2H), 8.08 (dd, *J* = 8.3, 1.5 Hz, 2H).
^{13}C NMR (101 MHz, $CDCl_3$) δ 157.83 (C, Ar), 146.46 (q, *J* = 4.0 Hz, CH, Ar), 134.52 (q, *J* = 3.5 Hz, CH, Ar), 127.32 (q, *J* = 33.0 Hz, C, Ar), 123.65 (q, *J* = 272.4 Hz, CF_3), 121.46 (CH, Ar).
^{19}F NMR (376 MHz, $CDCl_3$) δ -62.45.
HRMS (ESI). Calculated for $C_{12}H_7F_6N_2^+$: 293.050794 Obtained: 293.051312

Preparation of 4,4'-bis(trifluoromethyl)-2,2'-bipyridine (L6)

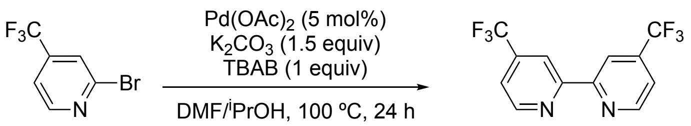

Prepared following an adapted procedure: To a round-bottom flask charged with a magnetic stir bar were added, $Pd(OAc)_2$ (25 mg, 0.11 mmol, 5 mol%), TBAB

(709 mg, 2.2 mmol, 1 eq.) and K_2CO_3 (456 mg, 3.3 mmol, 1.5 eq.). The flask was then evacuated and backfilled with N_2 three times. To this flask were added 2-bromo-4-(trifluoromethyl)pyridine (497 mg, 2.2 mmol, 1 eq.), ^{i}PrOH (0.34 mL, 6.5 M) and DMF (7.5 ml, 0.3 M) and the reaction was heated at 100 °C for 24 h.

The reaction mixture was then cooled to room temperature and filtered, washing copiously with EtOAc. The organics were washed three times with 40 mL of 10% (w/w) aqueous LiCl. The aqueous layer was backwashed once with EtOAc (50 mL) and the combined organics washed with water (40 mL) and brine (40 mL) before drying over MgSO4. Following removal of the solvent in vacuo, the crude residue was purified by silica gel chromatography (gradient from 100% hexane to 5% EtOAc/hexane) to afford 4,4'-bis(trifluoromethyl)-2,2'-bipyridine (292 mg, 90% yield) as a white solid.

^{1}H NMR (400 MHz, $CDCl_3$) δ 8.88 (dt, *J* = 5.0, 0.7 Hz, 1H), 8.76 – 8.70 (m, 1H), 7.58 (ddd, *J* = 5.1, 1.7, 0.8 Hz, 1H).
^{13}C NMR (101 MHz, $CDCl_3$) δ 156.19 (C, Ar), 150.40 (CH, Ar), 139.81 (C, Ar, q, *J* = 34.3 Hz), 122.98 (CF_3, q, *J* = 273.3 Hz), 120.08 (CH, Ar, q, *J* = 3.4 Hz), 117.34 (CH, Ar, q, *J* = 3.7 Hz).
^{19}F NMR (376 MHz, $CDCl_3$) δ -64.87.
HRMS (ESI). Calculated for $C_{12}H_7F_6N_2^+$: 293.0508 Obtained: 293.0508

Preparation of 6,6'-bis(trifluoromethyl)-2,2'-bipyridine (L7)

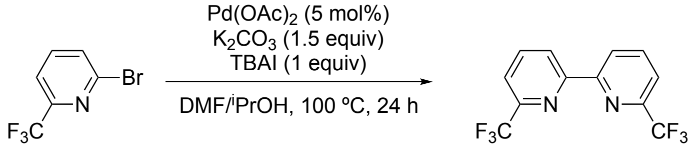

Prepared following an adapted procedure: To a round-bottom flask charged with a magnetic stir bar were added, $Pd(OAc)_2$ (9.9 mg, 0.04 mmol, 5 mol%), TBAI (327 mg, 0.88 mmol, 1 eq.) and K_2CO_3 (183 mg, 1.3 mmol, 1.5 eq.). The flask was then evacuated and backfilled with N_2 three times. To this flask were added 2-bromo-6-(trifluoromethyl)pyridine (200 mg, 0.88 mmol, 1 eq.), ^{i}PrOH (0.1 mL, 6.5 M) and DMF (3 ml, 0.3 M) and the reaction was heated at 100 °C for 24 h.

The reaction mixture was then cooled to room temperature and filtered, washing copiously with EtOAc. The organics were washed three times with 40 mL of 10% (w/w) aqueous LiCl. The aqueous layer was backwashed once with EtOAc (50 mL) and the combined organics washed with water (40 mL) and brine (40 mL) before drying over MgSO4. Following removal of the solvent in vacuo, the crude residue was purified by silica gel chromatography (gradient from 100% hexane to 5% EtOAc/hexane) to afford 6,6'-bis(trifluoromethyl)-2,2'-bipyridine (206 mg, 80% yield) as a white solid.

^{1}H NMR (400 MHz, $CDCl_3$) δ 8.73 (d, *J* = 8.1 Hz, 1H), 8.03 (td, *J* = 7.9, 0.7 Hz, 2H), 7.74 (dd, *J* = 7.8, 1.0 Hz, 2H).
^{13}C NMR (101 MHz, $CDCl_3$) δ 155.20 (C, Ar), 147.97 (q, *J* = 34.9 Hz, C, Ar), 138.71 (CH, Ar), 124.16 (CH, Ar), 121.61 (q, *J* = 274.3 Hz, CF_3), 121.02 (q, *J* = 2.8 Hz, CH, Ar).

^{19}**F NMR** (376 MHz, $CDCl_3$) δ -68.07.
HRMS (ESI). Calculated for $C_{12}H_7F_6N_2^+$: 293.05079 Obtained: 293.05088

Procedure for N-Me borylation

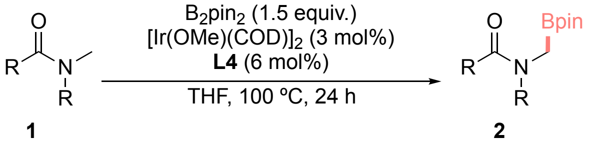

Following an adapted procedure: In a sealed tube $[Ir(COD)(OMe)]_2$ (5 mg, 0.008 mmol, 3 mol%.), B_2pin_2 (95.2 mg, 0.375 mmol, 1.5 equiv.), and **L4** (4.4 mg, 0.015 mmol, 6 mol%) were added. The tube was evacuated and refilled with N_2 three times, then under a positive flow of N_2, dry THF (1.25 mL) and substrate (0.250 mmol, 1.0 equiv.) were added. The tube was then sealed and heated for the specified time. After that, volatiles were removed under vacuum and CH_2Br_2 (internal standard) was added, the yield was determined by ^{1}H NMR spectroscopy. To isolate the product, the solvent was removed under reduced pressure, and the residue was purified by column chromatography on silica gel.

N-methyl-N-((4,4,5,5-tetramethyl-1,3,2-dioxaborolan-2-yl)methyl)hexanamide (2a)

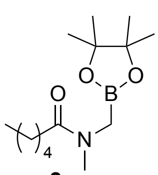

2a

Following the general procedure: **1a** (35.8 mg, 0.250 mmol, 1.0 eq.), $[Ir(COD)(OMe)]_2$ (5 mg, 0.008 mmol, 3 mol%), B_2pin_2 (95.2 mg, 0.375 mmol, 1.5 eq.), **L4** (4.4 mg, 0.015 mmol, 6 mol%) in THF (1.25 mL) were heated at 100 °C for 24 h. The crude mixture was purified by column chromatography (Hex/EtOAc 0% - EtOAc 100% - EtOAc/MeOH 20%) to obtain 65 mg (96% yield) of **2a**.

^{1}**H NMR** (600 MHz, $CDCl_3$, 258 K) δ 3.03 (s, 3H), 2.36 (s, 2H), 2.32 (t, *J* = 7.9 Hz, 2H), 1.64 (dq, *J* = 12.1, 7.5 Hz, 2H), 1.30 (dt, *J* = 7.3, 3.9 Hz, 4H), 1.15 (s, 12H), 0.89 – 0.84 (m, 3H).
^{13}**C NMR** (101 MHz, $CDCl_3$, 258 K) δ 176.94 (CO), 79.82 (C), 43.78 (N-CH_2-B), 35.79 (N-CH_3), 31.28 (CH_2), 28.42 (CH_2), 25.11 (CH_3), 24.23 (CH_2), 22.27 (CH_2), 13.99 (CH_3).
^{11}**B NMR** (128 MHz, $CDCl_3$, 258 K) δ 12.55.
HRMS (ESI). Calculated for $C_{14}H_{29}BNO_3^+$: 270.223501 Obtained: 270.223812

N-methyl-N-((4,4,5,5-tetramethyl-1,3,2-dioxaborolan-2-yl)methyl)acetamide (2b)

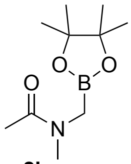

2b

Following the general procedure: **1b** (21.6 mg, 0.250 mmol, 1.0 eq.), $[Ir(COD)(OMe)]_2$ (5 mg, 0.008 mmol, 3 mol%), B_2pin_2 (95.2 mg, 0.375 mmol, 1.5 eq.), **L4** (4.4 mg, 0.015 mmol, 6 mol%) in THF (1.25 mL) were heated at 100 °C for 24 h. The crude mixture was purified by column chromatography (Hex/EtOAc 0% - EtOAc 100% - EtOAc/MeOH 20%) to obtain 21 mg (39% yield) of **2b**.

^{1}H NMR (600 MHz, $CDCl_3$, 258 K) δ 3.07 (s, 3H), 2.40 (s, 2H), 2.16 – 2.11 (m, 3H), 1.18 (s, 12H).
^{13}C NMR (101 MHz, $CDCl_3$, 258 K) δ 174.56 (CO), 80.20 (C), 43.72 (N-CH_2-B), 36.26 (N-CH_3), 25.24 (CH_3), 15.67 (CH_3).
^{11}B NMR (128 MHz, $CDCl_3$, 258 K) δ 12.61.
HRMS (ESI). Calculated for $C_{10}H_{20}BNNaO_3^+$: 236.142845 Obtained: 236.142964

N-methyl-N-((4,4,5,5-tetramethyl-1,3,2-dioxaborolan-2-yl)methyl)propionamide (2c)

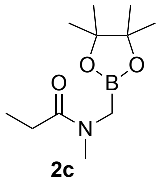

2c

Following the general procedure: **1c** (25.3 mg, 0.250 mmol, 1.0 eq.), $[Ir(COD)(OMe)]_2$ (5 mg, 0.008 mmol, 3 mol%), B_2pin_2 (95.2 mg, 0.375 mmol, 1.5 eq.), **L4** (4.4 mg, 0.015 mmol, 6 mol%) in THF (1.25 mL) were heated at 100 °C for 24 h. The crude mixture was purified by column chromatography (Hex/EtOAc 0% - EtOAc 100% - EtOAc/MeOH 20%) to obtain 52 mg (92% yield) of **2c**.

^{1}H NMR (600 MHz, $CDCl_3$, 258 K) δ 3.01 (s, 3H), 2.41 – 2.31 (m, 4H), 1.18 (dd, *J* = 6.1, 1.3 Hz, 3H), 1.14 (s, 12H).
^{13}C NMR (101 MHz, $CDCl_3$, 258 K) δ 177.62 (CO), 80.00 (C), 44.36 (N-CH_2-B), 35.60 (N-CH_3), 25.17 (CH_3), 22.13 (CH_2), 8.76 (CH_3).
^{11}B NMR (128 MHz, $CDCl_3$, 258 K) δ 12.59
HRMS (ESI). Calculated for $C_{11}H_{22}BNNaO_3^+$: 250.158495 Obtained: 250.158519

N-methyl-N-((4,4,5,5-tetramethyl-1,3,2-dioxaborolan-2-yl)methyl)butyramide (2d)

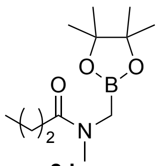

2d

Following the general procedure: **1d** (28.8 mg, 0.250 mmol, 1.0 eq.), $[Ir(COD)(OMe)]_2$ (5 mg, 0.008 mmol, 3 mol%), B_2pin_2 (95.2 mg, 0.375 mmol, 1.5 eq.), **L4** (4.4 mg, 0.015 mmol, 6 mol%) in THF (1.25 mL) were heated at 100 °C for 24 h. The crude mixture was purified by column chromatography (Hex/EtOAc 0% - EtOAc 100% - EtOAc/MeOH 20%) to obtain 54 mg (89% yield) of **2d**.

^{1}H NMR (600 MHz, $CDCl_3$, 258 K) δ 3.04 (s, 3H), 2.37 (s, 2H), 2.32 (t, *J* = 7.5 Hz, 2H), 1.69 (h, *J* = 7.4 Hz, 2H), 1.16 (s, 12H), 0.97 (t, *J* = 7.4 Hz, 3H).
^{13}C NMR (101 MHz, $CDCl_3$, 258 K) δ 176.97 (CO), 80.01 (C), 44.20 (N-CH_2-B), 35.81 (N-CH_3), 30.45 (CH_2), 25.25 (CH_3), 18.32 (CH_2), 13.91 (CH_3).
^{11}B NMR (128 MHz, $CDCl_3$, 258 K) δ 12.61
HRMS (ESI). Calculated for $C_{12}H_{24}BNNaO_3^+$: 264.174145 Obtained: 264.174192

N,2-dimethyl-N-((4,4,5,5-tetramethyl-1,3,2-dioxaborolan-2-yl)methyl)tridecanamide (2e)

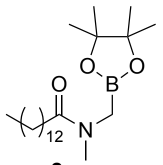
2e

Following the general procedure: **1e** (63.9 mg, 0.250 mmol, 1.0 eq.), $[Ir(COD)(OMe)]_2$ (5 mg, 0.008 mmol, 3 mol%), B_2pin_2 (95.2 mg, 0.375 mmol, 1.5 eq.), **L4** (4.4 mg, 0.015 mmol, 6 mol%) in THF (1.25 mL) were heated at 100 °C for 24 h. The crude mixture was purified by column chromatography (Hex/EtOAc 0% - EtOAc 100% - EtOAc/MeOH 20%) to obtain 65 mg (68% yield) of **2e**.

^{1}H NMR (600 MHz, $CDCl_3$, 258 K) δ 3.01 (s, 3H), 2.34 (s, 2H), 2.33 – 2.28 (m, 2H), 1.62 (dq, *J* = 8.8, 7.0 Hz, 2H), 1.21 (s, 20H), 1.14 (s, 12H), 0.87 – 0.80 (m, 3H).
^{13}C NMR (101 MHz, $CDCl_3$, 258 K) δ 177.09 (CO), 79.93 (C), 43.97 (N-CH_2-B), 35.76 (N-CH_3), 31.97 (CH_2), 29.70 (CH_2), 29.68 (CH_2), 29.61 (CH_2), 29.42 (CH_2), 29.40 (CH_2), 29.24 (CH_2), 29.20 (CH_2), 28.53 (CH_2), 25.20 (CH_3), 24.63 (CH_2), 22.74 (CH_2), 14.17 (CH_3).
^{11}B NMR (128 MHz, $CDCl_3$, 258 K) δ 12.62.
HRMS (ESI). Calculated for $C_{22}H_{45}BNO_3^+$: 382.348701 Obtained: 382.348974

N-methyl-N-((4,4,5,5-tetramethyl-1,3,2-dioxaborolan-2-yl)methyl)isobutyramide (2f)

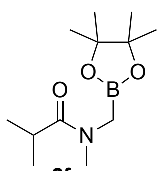
2f

Following the general procedure: **1f** (28.8 mg, 0.250 mmol, 1.0 eq.), $[Ir(COD)(OMe)]_2$ (5 mg, 0.008 mmol, 3 mol%), B_2pin_2 (95.2 mg, 0.375 mmol, 1.5 eq.), **L4** (4.4 mg, 0.015 mmol, 6 mol%) in THF (1.25 mL) were heated at 100 °C for 24 h. The crude mixture was purified by column chromatography (Hex/EtOAc 0% - EtOAc 100% - EtOAc/MeOH 20%) to obtain 41 mg (67% yield) of **2f**.

^{1}H NMR (600 MHz, $CDCl_3$, 258 K) δ 3.04 (s, 3H), 2.73 (p, *J* = 6.8 Hz, 1H), 2.34 (s, 2H), 1.16 (s, 3H), 1.15 (s, 3H), 1.14 (s, 12H).
^{13}C NMR (101 MHz, $CDCl_3$, 258 K) δ 180.32 (CO), 79.91 (C), 44.50 (N-CH_2-B), 35.52 (N-CH_3), 27.44 (CH), 25.16 (CH_3), 18.54 (CH_3).
^{11}B NMR (128 MHz, $CDCl_3$, 258 K) δ 12.37
HRMS (ESI). Calculated for $C_{12}H_{24}BNNaO_3^+$: 264.174145 Obtained: 264.174077

N-methyl-N-((4,4,5,5-tetramethyl-1,3,2-dioxaborolan-2-yl)methyl)pivalamide (2g)

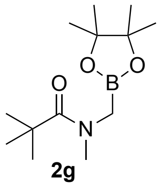
2g

Following the general procedure: **1g** (32.3 mg, 0.250 mmol 1.0 eq.), $[Ir(COD)(OMe)]_2$ (5 mg, 0.008 mmol, 3 mol%), B_2pin_2 (95.2 mg, 0.375 mmol, 1.5 eq.), **L4** (4.4 mg, 0.015 mmol, 6 mol%) in THF (1.25 mL) were heated at 100 °C for 24 h. The crude mixture was purified by column chromatography (Hex/EtOAc 0% - EtOAc 100% - EtOAc/MeOH 20%) to obtain 36 mg (56% yield) of **2g**.

^{1}H NMR (600 MHz, $CDCl_3$, 258 K) δ 3.20 (s, 3H), 2.44 (s, 2H), 1.29 (s, 9H), 1.15 (s, 12H).

^{13}C NMR (101 MHz, $CDCl_3$, 258 K) δ 181.10 (CO), 79.73 (C), 47.02 (N-CH_2-B), 37.73 (N-CH_3), 35.63 (C), 27.18 (CH_3), 25.12 (CH_3).
^{11}B NMR (128 MHz, $CDCl_3$, 258 K) δ 11.71.
HRMS (ESI). Calculated for $C_{13}H_{26}BNNaO_3^+$: 278.189795 Obtained: 278.190164

N,3-dimethyl-N-((4,4,5,5-tetramethyl-1,3,2-dioxaborolan-2-yl)methyl)butanamide (2h)

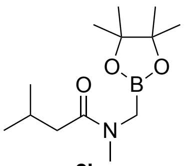

2h

Following the general procedure: **1h** (32.3 mg, 0.250 mmol, 1.0 eq.), $[Ir(COD)(OMe)]_2$ (5 mg, 0.008 mmol, 3 mol%), B_2pin_2 (95.2 mg, 0.375 mmol, 1.5 eq.), **L4** (4.4 mg, 0.015 mmol, 6 mol%) in THF (1.25 mL) were heated at 100 °C for 24 h. The crude mixture was purified by column chromatography (Hex/EtOAc 0% - EtOAc 100% - EtOAc/MeOH 20%) to obtain 28 mg (43% yield) of **2h.**

^{1}H NMR (600 MHz, $CDCl_3$, 258 K) δ 3.07 (s, 3H), 2.39 (s, 2H), 2.25 (d, *J* = 7.6 Hz, 2H), 2.15 (dtd, *J* = 13.1, 6.5, 1.3 Hz, 1H), 1.16 (s, 12H), 0.96 (s, 3H), 0.95 (s, 3H).
^{13}C NMR (101 MHz, $CDCl_3$, 258 K) δ 176.39 (CO), 79.84 (C), 43.89 (N-CH_2-B), 36.81 (CH_2), 36.12 (N-CH_3), 26.08 (CH), 25.14 (CH_3), 22.65 (CH_3).
^{11}B NMR (128 MHz, $CDCl_3$, 258 K) δ 12.42.
HRMS (ESI). Calculated for $C_{13}H_{26}BNNaO_3^+$: 278.189795 Obtained: 278.190334

N,3,3-trimethyl-N-((4,4,5,5-tetramethyl-1,3,2-dioxaborolan-2-yl)methyl)butanamide (2i)

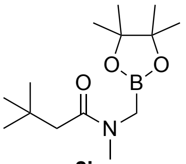

2i

Following the general procedure: **1i** (35.8 mg, 0.250 mmol, 1.0 eq.), $[Ir(COD)(OMe)]_2$ (5 mg, 0.008 mmol, 3 mol%), B_2pin_2 (95.2 mg, 0.375 mmol, 1.5 eq.), **L4** (4.4 mg, 0.015 mmol, 6 mol%) in THF (1.25 mL) were heated at 100 °C for 24 h. The crude mixture was purified by column chromatography (Hex/EtOAc 0% - EtOAc 100% - EtOAc/MeOH 20%) to obtain 54 mg (80% yield) of **2i**.

^{1}H NMR (600 MHz, $CDCl_3$, 258 K) δ 3.08 (s, 3H), 2.38 (s, 2H), 2.29 (s, 2H), 1.14 (s, 12H), 1.01 (s, 9H).
^{13}C NMR (101 MHz, $CDCl_3$, 258 K) δ 175.70 (CO), 79.73 (C), 43.89 (N-CH_2-B), 40.45 (CH_2), 36.82 (N-CH_3), 32.82 (C), 29.83 (CH_3), 25.11 (CH_3).
^{11}B NMR (128 MHz, $CDCl_3$, 258 K) δ 12.02.
HRMS (ESI). Calculated for $C_{14}H_{28}BNNaO_3^+$: 292.205445 Obtained: 292.206017

N-methyl-N-((4,4,5,5-tetramethyl-1,3,2-dioxaborolan-2-yl)methyl)cyclopentanecarboxamide (2j)

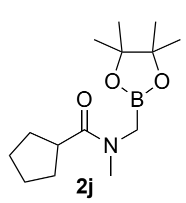

2j

Following the general procedure: **1j** (35.2 mg, 0.250 mmol, 1.0 eq.), $[Ir(COD)(OMe)]_2$ (5 mg, 0.008 mmol, 3 mol%), B_2pin_2 (95.2 mg, 0.375 mmol, 1.5 eq.), **L4** (4.4 mg, 0.015 mmol, 6 mol%) in THF (1.25 mL) were heated at 100 °C for 24 h. The crude mixture was

purified by column chromatography (Hex/EtOAc 0% - EtOAc 100% - EtOAc/MeOH 20%) to obtain 20 mg (30% yield) of **2j**.

^{1}H NMR (600 MHz, $CDCl_3$, 258 K) δ 3.06 (s, 3H), 2.82 (p, *J* = 8.1 Hz, 1H), 2.37 (s, 2H), 1.91 – 1.73 (m, 6H), 1.63 – 1.54 (m, 2H), 1.15 (s, 12H).
^{13}C NMR (101 MHz, $CDCl_3$, 258 K) δ 179.77 (CO), 79.77 (C), 44.13 (N-CH_2-B), 37.33 (CH), 35.75 (CH_3), 29.73 (CH_2), 25.90 (CH_2), 25.15 (CH_3).
^{11}B NMR (128 MHz, $CDCl_3$, 258 K) δ 12.32.
HRMS (ESI). Calculated for $C_{14}H_{26}BNNaO_3^{+}$: 290.189795 Obtained: 290.190580

N-methyl-N-((4,4,5,5-tetramethyl-1,3,2-dioxaborolan-2-yl)methyl)cyclohexanecarboxamide (2k)

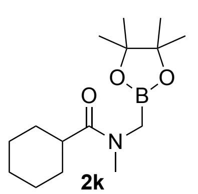
2k

Following the general procedure: **1k** (52.3 mg, 0.250 mmol, 1.0 eq.), $[Ir(COD)(OMe)]_2$ (5 mg, 0.008 mmol, 3 mol%), B_2pin_2 (95.2 mg, 0.375 mmol, 1.5 eq.), **L** (4.4 mg, 0.015 mmol, 6 mol%) in THF (1.25 mL) were heated at 100 °C for 24 h. The crude mixture was purified by column chromatography (Hex/EtOAc 0% - EtOAc 100% - EtOAc/MeOH 20%) to obtain 50 mg (70% yield) of **2k**.

^{1}H NMR (400 MHz, $CDCl_3$, 258 K) δ 3.05 (s, 3H), 2.42 (tt, *J* = 11.6, 3.5 Hz, 1H), 2.35 (s, 2H), 1.83 – 1.66 (m, 5H), 1.61 – 1.49 (m, 2H), 1.27 – 1.23 (m, 3H), 1.16 (s, 12H).
^{13}C NMR (101 MHz, $CDCl_3$, 258 K) δ 179.42 (CO), 79.90 (C), 43.93 (N-CH_2-B), 37.16 (CH), 35.58, (N-CH_3) 28.37 (CH_2), 25.50 (CH_2), 25.46 (CH_2), 25.25 (CH_3).
^{11}B NMR (128 MHz, $CDCl_3$, 258 K) δ 12.46.
HRMS (ESI). Calculated for $C_{15}H_{29}BNO_3^{+}$: 282.223501 Obtained: 282.223615

N-methyl-N-((4,4,5,5-tetramethyl-1,3,2-dioxaborolan-2-yl)methyl)cycloheptanecarboxamide (2l)

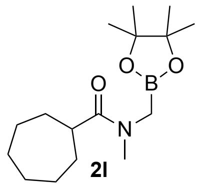
2l

Following the general procedure: **1l** (42.3 mg, 0.250 mmol, 1.0 eq.), $[Ir(COD)(OMe)]_2$ (5 mg, 0.008 mmol, 3 mol%), B_2pin_2 (95.2 mg, 0.375 mmol, 1.5 eq.), **L4** (4.4 mg, 0.015 mmol, 6 mol%) in THF (1.25 mL) were heated at 100 °C for 24 h. The crude mixture was purified by column chromatography (Hex/EtOAc 0% - EtOAc 100% - EtOAc/MeOH 20%) to obtain 70 mg (95% yield) of **2l**.

^{1}H NMR (600 MHz, $CDCl_3$, 258 K) δ 3.04 (s, 3H), 2.57 (ddd, *J* = 9.6, 7.5, 3.8 Hz, 1H), 2.33 (s, 2H), 1.82 – 1.65 (m, 6H), 1.52 (t, *J* = 3.8 Hz, 4H), 1.43 – 1.34 (m, 2H), 1.13 (s, 12H).
^{13}C NMR (101 MHz, $CDCl_3$, 258 K) δ 180.34 (CO), 79.72 (C), 43.97 (N-CH_2-B), 38.04 (CH), 35.70 (CH_3), 30.07 (CH_2), 28.09 (CH_2), 26.32 (CH_2), 25.09 (CH_3).
^{11}B NMR (128 MHz, $CDCl_3$, 258 K) δ 12.22.
HRMS (ESI). Calculated for $C_{16}H_{30}BNNaO_3^{+}$: 318.221095 Obtained: 318.221747

(3r,5r,7r)-N-methyl-N-((4,4,5,5-tetramethyl-1,3,2-dioxaborolan-2-yl)methyl)adamantane-1-carboxamide (2m)

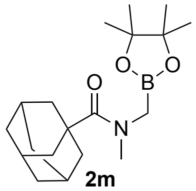

2m

Following the general procedure: **1m** (51.8 mg, 0.250 mmol, 1.0 eq.), $[Ir(COD)(OMe)]_2$ (5 mg, 0.008 mmol, 3 mol%), B_2pin_2 (95.2 mg, 0.375 mmol, 1.5 eq.), **L4** (4.4 mg, 0.015 mmol, 6 mol%) in THF (1.25 mL) were heated at 100 °C for 24 h. The crude mixture was purified by column chromatography (Hex/EtOAc 0% - EtOAc 100% - EtOAc/MeOH 20%) to obtain 72 mg (86% yield) of **2m**.

^{1}H NMR (600 MHz, $CDCl_3$, 258 K) δ 3.23 (s, 3H), 2.40 (s, 2H), 2.02 – 1.96 (m, 9H), 1.66 (dt, *J* = 6.7, 2.9 Hz, 6H), 1.14 (s, 12H).
^{13}C NMR (101 MHz, $CDCl_3$, 258 K) δ 179.96 (CO), 79.65 (C), 47.30 (N-CH_2-B), 38.13 (C), 37.80 (N-CH_3), 37.30 (CH_2), 36.01 (CH_2), 27.60 (CH), 25.10 (N-CH_3).
^{11}B NMR (128 MHz, $CDCl_3$, 258 K) δ 11.39.
HRMS (ESI). Calculated for $C_{19}H_{32}BNNaO_3^+$: 356.236745 Obtained: 356.238031

4,4-difluoro-N-methyl-N-((4,4,5,5-tetramethyl-1,3,2-dioxaborolan-2-yl)methyl)cyclohexane-1-carboxamide (2n)

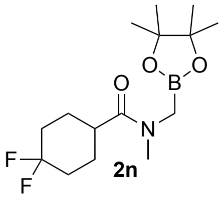

2n

Following the general procedure: **1n** (47.8 mg, 0.250 mmol, 1.0 eq.), $[Ir(COD)(OMe)]_2$ (5 mg, 0.008 mmol, 3 mol%), B_2pin_2 (95.2 mg, 0.375 mmol, 1.5 eq.), **L4** (4.4 mg, 0.015 mmol, 6 mol%) in THF (1.25 mL) were heated at 100 °C for 24 h. The crude mixture was purified by column chromatography (Hex/EtOAc 0% - EtOAc 100% - EtOAc/MeOH 20%) to obtain 74 mg (93% yield) of **2n**.

^{1}H NMR (600 MHz, $CDCl_3$, 258 K) δ 3.08 (s, 3H), 2.55 (dt, *J* = 10.2, 5.3 Hz, 1H), 2.36 (s, 2H), 2.24 – 2.14 (m, 2H), 1.93 – 1.69 (m, 6H), 1.13 (s, 12H).
^{13}C NMR (101 MHz, $CDCl_3$, 258 K) δ 177.56 (CO), 122.23 (t, *J* = 241.3 Hz, CF_2), 79.93 (C), 43.80 (N-CH_2-B), 35.76 (N-CH_3), 34.15 (CH), 32.31 (t, *J* = 24.7 Hz, CH_2), 25.10, 24.65 (d, *J* = 10.3 Hz, CH_2).
^{19}F NMR (376 MHz, $CDCl_3$, 258 K) δ -93.89 (d, *J* = 237.9 Hz), -100.06 (d, *J* = 237.9 Hz).
^{11}B NMR (128 MHz, $CDCl_3$, 258 K) δ 12.83
HRMS (ESI). Calculated for $C_{15}H_{26}BF_2NNaO_3^+$: 340.186601 Obtained: 340.187716

5-chloro-N-methyl-N-((4,4,5,5-tetramethyl-1,3,2-dioxaborolan-2-yl)methyl)pentanamide (2o)

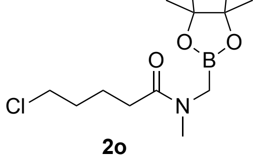

2o

Following the general procedure: **1o** (40.9 mg, 0.250 mmol, 1.0 eq.), $[Ir(COD)(OMe)]_2$ (5 mg, 0.008 mmol, 3 mol%), B_2pin_2 (95.2 mg, 0.375 mmol, 1.5 eq.), **L4** (4.4 mg, 0.015 mmol, 6 mol%) in THF (1.25 mL) were heated at 100 °C for 24 h. The crude mixture was purified by column chromatography (Hex/EtOAc 0% - EtOAc 100% - EtOAc/MeOH 20%) to obtain 15 mg (21% yield) of **2o**.

^{1}H NMR (600 MHz, $CDCl_3$, 258 K) δ 3.59 – 3.52 (m, 2H), 3.05 (s, 3H), 2.44 – 2.35 (m, 4H), 1.84 (dq, *J* = 6.6, 3.2 Hz, 4H), 1.17 (s, 12H).
^{13}C NMR (101 MHz, $CDCl_3$, 258 K) δ 176.22 (CO), 79.99 (C), 44.22 (CH_2), 43.61 (N-CH_2-B), 35.70 (N-CH_3), 31.54 (CH_2), 27.57 (CH_2), 25.12 (CH_3), 21.81 (CH_2).
^{11}B NMR (128 MHz, $CDCl_3$, 258 K) δ 12.92
HRMS (ESI). Calculated for $C_{13}H_{26}BClNO_3^+$: 290.168878 Obtained: 290.168893

N-isopropyl-N-((4,4,5,5-tetramethyl-1,3,2-dioxaborolan-2-yl)methyl)cyclohexanecarboxamide (2p)

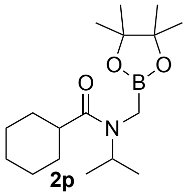

Following the general procedure: **1p** (45.8 mg, 0.250 mmol, 1.0 eq.), $[Ir(COD)(OMe)]_2$ (5 mg, 0.008 mmol, 3 mol%), B_2pin_2 (95.2 mg, 0.375 mmol, 1.5 eq.), **L4** (4.4 mg, 0.015 mmol, 6 mol%) in THF (1.25 mL) were heated at 100 °C for 24 h. The crude mixture was purified by column chromatography (Hex/EtOAc 0% - EtOAc 100% - EtOAc/MeOH 20%) to obtain 54 mg (70% yield) of **2p.**

^{1}H NMR (600 MHz, $CDCl_3$, 258 K) δ 4.00 (p, *J* = 6.6 Hz, 1H), 2.42 (tt, *J* = 11.8, 3.5 Hz, 1H), 2.20 (s, 2H), 1.83 – 1.74 (m, 2H), 1.69 (ddd, *J* = 15.2, 7.7, 4.4 Hz, 3H), 1.57 (dt, *J* = 12.4, 6.0 Hz, 2H), 1.24 – 1.20 (m, 3H), 1.18 (s, 6H), 1.16 (s, 12H).
^{13}C NMR (101 MHz, $CDCl_3$, 258 K) δ 178.20 (CO), 79.61 (C), 47.61 (N-CH), 37.27 (CH), 32.93 (N-CH2-B), 28.64 (CH_2), 25.42 (CH_2), 25.30 (CH_2), 20.63 (CH_3).
^{11}B NMR (128 MHz, $CDCl_3$, 258 K) δ 12.11
HRMS (ESI). Calculated for $C_{17}H_{32}BNNaO_3^+$: 332.236745 Obtained: 332.236742

N-cyclopropyl-N-((4,4,5,5-tetramethyl-1,3,2-dioxaborolan-2-yl)methyl)hexanamide (2q)

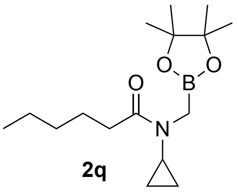

Following the general procedure: **1q** (42.3 mg, 0.250 mmol, 1.0 eq.), $[Ir(COD)(OMe)]_2$ (5 mg, 0.008 mmol, 3 mol%), B_2pin_2 (95.2 mg, 0.375 mmol, 1.5 eq.), **L4** (4.4 mg, 0.015 mmol, 6 mol%) in THF (1.25 mL) were heated at 100 °C for 24 h. The crude mixture was purified by column chromatography (Hex/EtOAc 0% - EtOAc 100% - EtOAc/MeOH 20%) to obtain 64 mg (86% yield) of **2q**.

^{1}H NMR (600 MHz, $CDCl_3$, 258 K) δ 2.67 (tt, *J* = 7.2, 4.0 Hz, 1H), 2.51 – 2.45 (m, 2H), 2.27 (s, 2H), 1.71 – 1.61 (m, 2H), 1.31 (ddd, *J* = 7.3, 4.3, 3.2 Hz, 4H), 1.14 (s, 12H), 0.90 – 0.81 (m, 5H), 0.77 – 0.71 (m, 2H).
^{13}C NMR (101 MHz, $CDCl_3$, 258 K) δ 179.93 (CO), 79.97 (C), 40.70 (N-CH_2-B), 31.45 (CH_2), 29.49 (CH_2), 29.46 (CH), 25.21 (CH_3), 24.14 (CH_2), 22.30 (CH_2), 13.89 (CH_3), 6.97 (CH_2).
^{11}B NMR (128 MHz, $CDCl_3$, 258 K) δ 12.77
HRMS (ESI). Calculated for $C_{16}H_{30}BNNaO_3^+$: 318.221095 Obtained: 318.221084

1-((4,4,5,5-tetramethyl-1,3,2-dioxaborolan-2-yl)methyl)piperidin-2-one (2r)

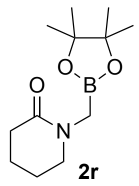

Following the general procedure: **1r** (28.3 mg, 0.250 mmol, 1.0 eq.), $[Ir(COD)(OMe)]_2$ (5 mg, 0.008 mmol, 3 mol%), B_2pin_2 (95.2 mg, 0.375 mmol, 1.5 eq.), **L4** (4.4 mg, 0.015 mmol, 6 mol%) in THF (1.25 mL) were heated at 100 °C for 24 h. The crude mixture was purified by column chromatography (Hex/EtOAc 0% - EtOAc 100% - EtOAc/MeOH 20%) to obtain 54 mg (90% yield) of **2r.**

^{1}H NMR (600 MHz, $CDCl_3$, 258 K) δ 3.30 (t, *J* = 5.4 Hz, 2H), 2.51 – 2.44 (m, 2H), 2.33 (d, *J* = 1.7 Hz, 2H), 1.84 – 1.75 (m, 4H), 1.17 (s, 12H).
^{13}C NMR (101 MHz, $CDCl_3$, 258 K) δ 174.13 (CO), 80.12 (C), 47.96 (CH_2), 42.54 (N-CH_2-B), 26.54 (CH_2), 25.24 (CH_2), 22.12 (CH_2), 19.57 (CH_3).
^{11}B NMR (128 MHz, $CDCl_3$, 258 K) δ 12.75.
HRMS (ESI). Calculated for $C_{12}H_{22}BNNaO_3^+$: 262.158495 Obtained: 262.166977

N-((4,4,5,5-tetramethyl-1,3,2-dioxaborolan-2-yl)methyl)cyclohexanecarboxamide (2s)

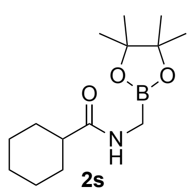

Following the general procedure: **1s** (35.3 mg, 0.250 mmol, 1.0 eq.), $[Ir(COD)(OMe)]_2$ (5 mg, 0.008 mmol, 3 mol%), B_2pin_2 (95.2 mg, 0.375 mmol, 1.5 eq.), **L4** (4.4 mg, 0.015 mmol, 6 mol%) in THF (1.25 mL) were heated at 100 °C for 24 h. The crude mixture was purified by column chromatography (Hex/EtOAc 0% - EtOAc 100% - EtOAc/MeOH 20%) to obtain 49 mg (73% yield) of **2s**.

^{1}H NMR (600 MHz, $CDCl_3$, 258 K) δ 5.74 (s, 1H), 2.76 (d, *J* = 4.7 Hz, 2H), 2.06 (tt, *J* = 11.8, 3.5 Hz, 1H), 1.85 – 1.72 (m, 4H), 1.67 – 1.60 (m, 1H), 1.46 – 1.32 (m, 2H), 1.22 (d, *J* = 9.3 Hz, 15H).
^{13}C NMR (101 MHz, $CDCl_3$, 258 K) δ 177.14 (CO), 82.98 (C), 45.55 (CH), 29.76 (CH_2), 25.84 (CH_2), 24.62 (CH_3).
^{11}B NMR (128 MHz, $CDCl_3$, 258 K) δ 22.19.
HRMS (ESI). Calculated for $C_{14}H_{27}BNO_3^+$: 268.207851 Obtained: 268.207982

1,1-diethyl-3-methyl-3-((4,4,5,5-tetramethyl-1,3,2-dioxaborolan-2-yl)methyl)urea (2t)

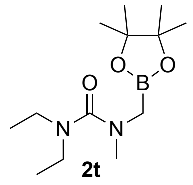

Following the general procedure: **1t** (36.1 mg, 0.250 mmol, 1.0 eq.), $[Ir(COD)(OMe)]_2$ (5 mg, 0.008 mmol, 3 mol%), B_2pin_2 (95.2 mg, 0.375 mmol, 1.5 eq.), **L4** (4.4 mg, 0.015 mmol, 6 mol%) in THF (1.25 mL) were heated at 100 °C for 24 h. The crude mixture was purified by column chromatography (Hex/EtOAc 0% - EtOAc 100% - EtOAc/MeOH 20%) to obtain 31 mg (46% yield) of **2t**.

^{1}H NMR (600 MHz, $CDCl_3$, 258 K) δ 3.25 (q, *J* = 7.1 Hz, 4H), 3.00 (s, 3H), 2.44 (s, 2H), 1.17 (t, *J* = 7.1 Hz, 6H), 1.15 (s, 12H).

^{13}C NMR (101 MHz, $CDCl_3$, 258 K) δ 164.29 (CO), 79.36 (C), 47.92 (N-CH_2-B), 43.22 (N-CH_2), 38.42 (N-CH_3), 25.17 (CH_3), 13.61 (CH_3).
^{11}B NMR (128 MHz, $CDCl_3$, 258 K) δ 10.91
HRMS (ESI). Calculated for $C_{13}H_{27}BN_2NaO_3^+$: 293.200694 Obtained: 293.200933

N-phenyl-N-((4,4,5,5-tetramethyl-1,3,2-dioxaborolan-2-yl)methyl)cyclohexanecarboxamide (2x)

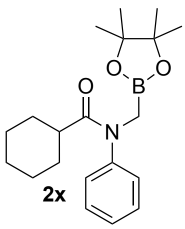

2x

Following the general procedure: **1x** (54.3 mg, 0.250 mmol, 1.0 eq.), $[Ir(COD)(OMe)]_2$ (5 mg, 0.008 mmol, 3 mol%), B_2pin_2 (95.2 mg, 0.375 mmol, 1.5 eq.), **L4** (4.4 mg, 0.015 mmol, 6 mol%) in THF (1.25 mL) were heated at 100 °C for 24 h. The crude mixture was purified by column chromatography (Hex/EtOAc 0% - EtOAc 100% - EtOAc/MeOH 20%) to obtain 43 mg (50% yield) of **2x.**

^{1}H NMR (600 MHz, $CDCl_3$, 258 K) δ 7.47 – 7.37 (m, 3H), 7.16 – 7.11 (m, 2H), 2.75 (s, 2H), 2.28 – 2.20 (m, 1H), 1.69 (d, J = 11.0 Hz, 4H), 1.62 – 1.52 (m, 3H), 1.27 – 1.22 (m, 3H), 1.21 (s, 12H).
^{13}C NMR (101 MHz, $CDCl_3$, 258 K) δ 180.21 (CO), 139.75 (C, Ar), 130.03 (CH, Ar), 128.78 (CH, Ar), 125.60 (CH, Ar), 80.03 (C), 46.74 (N-CH_2-B), 37.48 (CH), 28.74 (CH_2), 25.21 (CH_3), 25.02 (CH_2).
^{11}B NMR (128 MHz, $CDCl_3$, 258 K) δ 12.80.
HRMS (ESI). Calculated for $C_{20}H_{31}BNO_3^+$: 344.2392 Obtained: 344.2395

N-benzyl-N-((4,4,5,5-tetramethyl-1,3,2-dioxaborolan-2-yl)methyl)cyclohexanecarboxamide (2y)

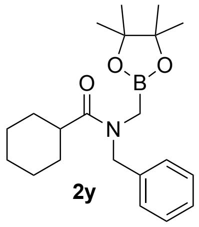

2y

Following the general procedure: **1y** (57.8 mg, 0.250 mmol, 1.0 eq.), $[Ir(COD)(OMe)]_2$ (5 mg, 0.008 mmol, 3 mol%), B_2pin_2 (95.2 mg, 0.375 mmol, 1.5 eq.), **L4** (4.4 mg, 0.015 mmol, 6 mol%) in THF (1.25 mL) were heated at 100 °C for 24 h. The crude mixture was purified by column chromatography (Hex/EtOAc 0% - EtOAc 100% - EtOAc/MeOH 20%) to obtain 47 mg (52% yield) of **2y**.

^{1}H NMR (600 MHz, $CDCl_3$, 258 K) δ 7.36 – 7.26 (m, 3H), 7.13 (dd, J = 7.9, 1.6 Hz, 2H), 4.52 (s, 2H), 2.55 (tt, J = 11.6, 3.4 Hz, 1H), 2.27 (s, 2H), 1.83 – 1.74 (m, 4H), 1.72 – 1.55 (m, 3H), 1.21 (s, 3H), 1.14 (s, 12H).
^{13}C NMR (101 MHz, $CDCl_3$, 258 K) δ 179.87 (CO), 133.95 (C, Ar), 129.06 (CH, Ar), 128.30 (CH, Ar), 127.43 (CH, Ar), 79.83 (C), 52.08 (N-CH_2), 41.00 (N-CH_2-B), 37.24 (CH), 28.71 (CH_2), 25.31 (CH_2), 25.27 (CH_2), 25.05 (CH_3).
^{11}B NMR (128 MHz, $CDCl_3$) δ 12.65.
HRMS (ESI). Calculated for $C_{21}H_{33}BNO_3^+$: 358.254801 Obtained: 358.255302

N-methyl-2-phenyl-N-((4,4,5,5-tetramethyl-1,3,2-dioxaborolan-2-yl)methyl)acetamide (2z)

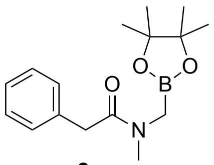

2z

Following the general procedure: **1z** (40.8 mg, 0.250 mmol, 1.0 eq.), [Ir(COD)(OMe)]$_2$ (5 mg, 0.008 mmol, 3 mol%), B$_2$pin$_2$ (95.2 mg, 0.375 mmol, 1.5 eq.), **L4** (4.4 mg, 0.015 mmol, 6 mol%) in THF (1.25 mL) were heated at 100 °C for 24 h. The crude mixture was purified by column chromatography (Hex/EtOAc 0% - EtOAc 100% - EtOAc/MeOH 20%) to obtain 65 mg (90% yield) of **2z** as an off-white solid.

^{1}H NMR (600 MHz, CDCl$_3$, 258 K) δ 7.33 – 7.26 (m, 3H), 7.22 (ddt, *J* = 7.3, 1.5, 0.7 Hz, 2H), 3.75 (s, 2H), 3.01 (s, 3H), 2.44 – 2.39 (m, 2H), 1.18 (s, 12H).
^{13}C NMR (101 MHz, CDCl$_3$, 258 K) δ 174.96 (CO), 132.07 (C, Ar), 129.13 (CH, Ar), 128.74 (CH, Ar), 127.70 (CH, Ar), 80.19 (C), 43.90 (N-CH$_2$-B), 36.21 (N-CH$_3$), 35.53 (CH$_2$), 25.22 (CH$_3$).
^{11}B NMR (128 MHz, CDCl$_3$, 258 K) δ 13.12.
HRMS (ESI). Calculated for $C_{16}H_{24}BNNaO_3^+$: 312.174145 Obtained: 312.174990

N-methyl-3-phenyl-N-((4,4,5,5-tetramethyl-1,3,2-dioxaborolan-2-yl)methyl)propanamide (2aa)

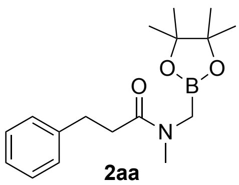

2aa

Following the general procedure: **1aa** (44.3 mg, 0.250 mmol, 1.0 eq.), [Ir(COD)(OMe)]$_2$ (5 mg, 0.008 mmol, 3 mol%), B$_2$pin$_2$ (95.2 mg, 0.375 mmol, 1.5 eq.), **L4** (4.4 mg, 0.015 mmol, 6 mol%) in THF (1.25 mL) were heated at 100 °C for 24 h. The crude mixture was purified by column chromatography (Hex/EtOAc 0% - EtOAc 100% - EtOAc/MeOH 20%) to obtain 61 mg (80% yield) of **2aa**.

^{1}H NMR (600 MHz, CDCl$_3$, 258 K) δ 7.32 – 7.27 (m, 2H), 7.25 – 7.17 (m, 3H), 3.04 – 2.97 (m, 2H), 2.93 (s, 3H), 2.69 – 2.60 (m, 2H), 2.39 (t, *J* = 0.9 Hz, 2H), 1.21 (s, 12H).
^{13}C NMR (101 MHz, CDCl$_3$, 258 K) δ 176.05 (CO), 139.87 (C, Ar), 128.89 (CH, Ar), 128.46 (CH, Ar), 126.81 (CH, Ar), 80.17 (C), 43.79 (N-CH$_2$-B), 35.70 (N-CH$_3$), 30.89 (CH$_2$), 30.77 (CH$_2$), 25.30 (CH$_3$).
^{11}B NMR (128 MHz, CDCl$_3$, 258 K) δ 12.86.
HRMS (ESI). Calculated for $C_{17}H_{26}BNNaO_3^+$: 326.189795 Obtained: 326.190588

N-methyl-4-phenyl-N-((4,4,5,5-tetramethyl-1,3,2-dioxaborolan-2-yl)methyl)butanamide (2ab)

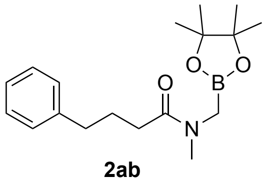

2ab

Following the general procedure: **1ab** (47.8 mg, 0.250 mmol, 1.0 eq.), [Ir(COD)(OMe)]$_2$ (5 mg, 0.008 mmol, 3 mol%), B$_2$pin$_2$ (95.2 mg, 0.375 mmol, 1.5 eq.), **L4** (4.4 mg, 0.015 mmol, 6 mol%) in THF (1.25 mL) were heated at 100 °C for 24 h. The crude mixture was purified by column chromatography (Hex/EtOAc 0% - EtOAc 100% - EtOAc/MeOH 20%) to obtain 71.8 mg (90% yield) of **2ab**.

^{1}H NMR (600 MHz, $CDCl_3$, 258 K) δ 7.30 – 7.23 (m, 2H), 7.22 – 7.09 (m, 3H), 2.96 (s, 3H), 2.68 (t, *J* = 7.5 Hz, 2H), 2.37 (s, 2H), 2.36 – 2.25 (m, 3H), 2.00 (tt, *J* = 8.5, 6.9 Hz, 2H), 1.18 (s, 12H).
^{13}C NMR (101 MHz, $CDCl_3$, 258 K) δ 176.71 (CO), 140.83 (C, Ar), 128.61 (CH, Ar), 128.56 (CH, Ar), 126.30 (CH, Ar), 80.08 (C), 43.87 (N-CH_2-B), 35.72 (N-CH_3), 34.94 (CH_2), 27.58 (CH_2), 25.92 (CH_2), 25.23 (CH_3).
^{11}B NMR (128 MHz, $CDCl_3$, 258 K) δ 12.74.
HRMS (ESI). Calculated for $C_{18}H_{28}BNNaO_3^+$: 340.205445 Obtained: 340.215715

3-(2-bromophenyl)-N-methyl-N-((4,4,5,5-tetramethyl-1,3,2-dioxaborolan-2-yl)methyl)propanamide (2ac)

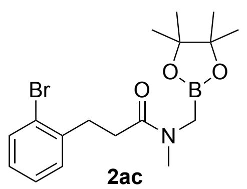

2ac

Following the general procedure: **1ac** (64 mg, 0.250 mmol, 1.0 eq.), $[Ir(COD)(OMe)]_2$ (5 mg, 0.008 mmol, 3 mol%), B_2pin_2 (95.2 mg, 0.375 mmol, 1.5 eq.), **L4** (4.4 mg, 0.015 mmol, 6 mol%) in THF (1.25 mL) were heated at 100 °C for 24 h. The crude mixture was purified by column chromatography (Hex/EtOAc 0% - EtOAc 100% - EtOAc/MeOH 20%) to obtain 57 mg (59% yield) of **2ac**.

^{1}H NMR (600 MHz, $CDCl_3$, 258 K) δ 7.55 (dd, *J* = 8.0, 1.1 Hz, 1H), 7.29 (ddd, *J* = 5.0, 3.5, 1.6 Hz, 2H), 7.12 (ddd, *J* = 7.8, 6.7, 2.4 Hz, 1H), 3.18 – 3.12 (m, 2H), 2.99 (s, 3H), 2.71 (dd, *J* = 8.7, 7.1 Hz, 2H), 2.43 (s, 2H), 1.25 (s, 12H).
^{13}C NMR (101 MHz, $CDCl_3$, 258 K) δ 175.84 (CO), 139.00 (C, Ar), 133.07 (CH, Ar), 131.13 (CH, Ar), 128.74 (CH, Ar), 128.11 (CH, Ar), 124.21 (C, Ar), 80.31 (C), 43.89 (N-CH_2-B), 35.79 (N-CH_3), 31.45 (CH_2), 28.82 (CH_2), 25.25 (CH_3).
^{11}B NMR (128 MHz, $CDCl_3$, 258 K) δ 13.00
HRMS (ESI). Calculated for $C_{17}H_{26}BBrNO_3^+$: 382.118363 Obtained: 382.118276

3-(2-bromophenyl)-N-methyl-N-((4,4,5,5-tetramethyl-1,3,2-dioxaborolan-2-yl)methyl)propanamide (2ad)

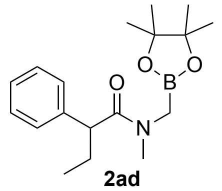

2ad

Following the general procedure: **1ad** (47.8 mg, 0.250 mmol, 1.0 eq.), $[Ir(COD)(OMe)]_2$ (5 mg, 0.008 mmol, 3 mol%), B_2pin_2 (95.2 mg, 0.375 mmol, 1.5 eq.), **L4** (4.4 mg, 0.015 mmol, 6 mol%) in THF (1.25 mL) were heated at 100 °C for 24 h. The crude mixture was purified by column chromatography (Hex/EtOAc 0% - EtOAc 100% - EtOAc/MeOH 20%) to obtain 51 mg (64% yield) of **2ad**.

^{1}H NMR (600 MHz, $CDCl_3$, 258 K) δ 7.34 – 7.28 (m, 2H), 7.27 – 7.21 (m, 3H), 3.52 (t, *J* = 7.4 Hz, 1H), 2.92 (s, 3H), 2.41 (d, *J* = 13.8 Hz, 1H), 2.32 (d, *J* = 13.8 Hz, 1H), 2.10 (dt, *J* = 13.8, 7.3 Hz, 1H), 1.82 (dt, *J* = 13.8, 7.4 Hz, 1H), 1.21 (d, *J* = 2.0 Hz, 12H), 0.87 (t, *J* = 7.4 Hz, 3H).
^{13}C NMR (101 MHz, $CDCl_3$, 258 K) δ 177.13 (CO), 137.48 (C, Ar), 129.18 (CH, Ar), 128.02 (CH, Ar), 127.77 (CH, Ar), 80.07 (C), 47.20 (CH), 43.90 (N-CH_2-B), 35.73 (N-CH_3), 27.48 (CH_2), 25.29 (CH_2), 24.74 (CH_3), 12.03 (CH_3).
^{11}B NMR (128 MHz, $CDCl_3$) δ 13.15
HRMS (ESI). Calculated for $C_{18}H_{29}BNO_3^+$: 318.223501 Obtained: 318.223326

(1R,2R)-N-methyl-2-phenyl-N-((4,4,5,5-tetramethyl-1,3,2-dioxaborolan-2-yl)methyl)cyclopropane-1-carboxamide (2ae)

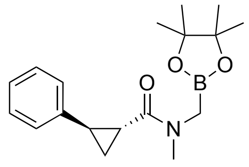

2ae

Following the general procedure: **1ae** (47.3 mg, 0.250 mmol, 1.0 eq.), $[Ir(COD)(OMe)]_2$ (5 mg, 0.008 mmol, 3 mol%), B_2pin_2 (95.2 mg, 0.375 mmol, 1.5 eq.), **L4** (4.4 mg, 0.015 mmol, 6 mol%) in THF (1.25 mL) were heated at 100 °C for 24 h. The crude mixture was purified by column chromatography (Hex/EtOAc 0% - EtOAc 100% - EtOAc/MeOH 20%) to obtain 42 mg (53% yield) of **2ae**.

^{1}H NMR (600 MHz, $CDCl_3$, 258 K) δ 7.30 (t, *J* = 7.4 Hz, 3H), 7.14 – 7.09 (m, 2H), 3.14 (s, 3H), 2.80 – 2.74 (m, 1H), 2.43 (s, 2H), 1.85 (dd, *J* = 9.2, 4.8 Hz, 2H), 1.54 (td, *J* = 7.3, 4.6 Hz, 1H), 1.20 – 1.15 (m, 12H).
^{13}C NMR (101 MHz, $CDCl_3$, 258 K) δ 175.47 (CO), 139.25 (C, Ar), 128.75 (CH, Ar), 126.98 (CH, Ar), 126.20 (CH, Ar), 79.92 (C), 44.60 (N-CH_2-B), 35.82 (N-CH_3), 27.19 (CH), 25.22 (CH_3), 19.68 (CH), 17.55 (CH_2).
^{11}B NMR (128 MHz, $CDCl_3$, 258 K) δ 12.24
HRMS (ESI). Calculated for $C_{18}H_{27}BNO_3^+$: 316.2079 Obtained: 316.2080

2-(1H-indol-3-yl)-N-methyl-N-((4,4,5,5-tetramethyl-1,3,2-dioxaborolan-2-yl)methyl)acetamide (2af)

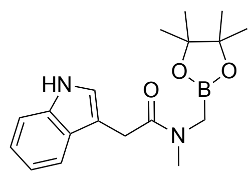

2af

Following the general procedure: **1af** (50.5 mg, 0.250 mmol, 1.0 eq.), $[Ir(COD)(OMe)]_2$ (5 mg, 0.008 mmol, 3 mol%), B_2pin_2 (95.2 mg, 0.375 mmol, 1.5 eq.), **L4** (4.4 mg, 0.015 mmol, 6 mol%) in THF (1.25 mL) were heated at 100 °C for 24 h. The crude mixture was purified by column chromatography (Hex/EtOAc 0% - EtOAc 100% - EtOAc/MeOH 20%) to obtain 63 mg (77% yield) of **2af**.

^{1}H NMR (400 MHz, $CDCl_3$, 258 K) δ 8.61 (s, 1H), 7.94 (dd, *J* = 8.1, 1.0 Hz, 1H), 7.31 – 7.27 (m, 1H), 7.19 (ddd, *J* = 8.2, 6.9, 1.2 Hz, 1H), 7.07 (ddd, *J* = 8.0, 6.9, 1.1 Hz, 1H), 4.19 (s, 2H), 3.12 (s, 3H), 2.35 (s, 2H), 1.33 (s, 12H).
^{13}C NMR (101 MHz, $CDCl_3$, 258 K) δ 175.42 (CO), 138.45 (C, Ar), 128.03 (C, Ar), 124.41 (CH, Ar), 121.74 (CH, Ar), 120.08 (CH, Ar), 118.46 (C, Ar), 111.17 (CH, Ar), 79.93 (C), 44.47 (N-CH_2), 36.07 (N-CH_3), 26.58 (CH_2), 25.26 (CH_3).
^{11}B NMR (128 MHz, $CDCl_3$, 258 K) δ 12.72.
HRMS (ESI). Calculated for $C_{18}H_{26}BN_2O_3^+$: 329.203099 Obtained: 329.203018

N-(pyridin-2-yl)-N-((4,4,5,5-tetramethyl-1,3,2-dioxaborolan-2-yl)methyl)hexanamide (2ag)

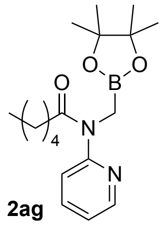

2ag

Following the general procedure: **1ag** (51.6 mg, 0.250 mmol, 1.0 eq.), $[Ir(COD)(OMe)]_2$ (5 mg, 0.008 mmol, 3 mol%), B_2pin_2 (95.2 mg, 0.375 mmol, 1.5 eq.), **L4** (4.4 mg, 0.015 mmol, 6 mol%) in THF (1.25 mL) were heated at 100 °C for 24 h. The crude mixture was purified by column chromatography (Hex/EtOAc 0% - EtOAc 100% - EtOAc/MeOH 20%) to obtain 73 mg (89% yield) of **2ag**.

^{1}H NMR (400 MHz, $CDCl_3$, 258 K) δ 8.66 (dd, *J* = 8.8, 1.0 Hz, 1H), 8.33 (ddd, *J* = 5.9, 1.8, 0.9 Hz, 1H), 7.88 (ddd, *J* = 8.9, 7.2, 1.7 Hz, 1H), 7.12 (ddd, *J* = 7.1, 5.9, 1.1 Hz, 1H), 2.90 (s, 2H), 2.65 – 2.57 (m, 2H), 2.27 – 2.14 (m, 4H), 1.71 – 1.62 (m, 2H), 1.25 (s, 12H), 0.89 (td, *J* = 5.7, 2.8 Hz, 3H).
^{13}C NMR (101 MHz, $CDCl_3$, 258 K) δ 175.85 (CO), 153.92 (C, Ar), 143.46 (CH, Ar), 139.66 (CH, Ar), 118.52 (CH, Ar), 116.23 (CH, Ar), 80.06 (C), 42.97 (N-CH_2), 36.38 (CH_2), 31.44 (CH_2), 24.93 (CH_3), 24.09 (CH_2), 22.59 (CH_2), 14.04 (CH_3).
^{11}B NMR (128 MHz, $CDCl_3$, 258 K) δ 9.79.
HRMS (ESI). Calculated for $C_{18}H_{30}BN_2O_3^+$: 333.234400 Obtained: 333.234372

(1S)-N,4,7,7-tetramethyl-3-oxo-N-((4,4,5,5-tetramethyl-1,3,2-dioxaborolan-2-yl)methyl)-2-oxabicyclo[2.2.1]heptane-1-carboxamide (2ah)

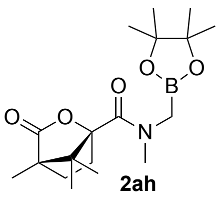

2ah

Following the general procedure: **1ah** (56.3 mg, 0.250 mmol, 1.0 eq.), $[Ir(COD)(OMe)]_2$ (5 mg, 0.008 mmol, 3 mol%), B_2pin_2 (95.2 mg, 0.375 mmol, 1.5 eq.), **L4** (4.4 mg, 0.015 mmol, 6 mol%) in THF (1.25 mL) were heated at 100 °C for 24 h. The crude mixture was purified by column chromatography (Hex/EtOAc 0% - EtOAc 100% - EtOAc/MeOH 20%) to obtain 72 mg (82% yield) of **2ah**.

^{1}H NMR (600 MHz, $CDCl_3$, 258 K) δ 3.27 (s, 3H), 2.52 – 2.40 (m, 3H), 2.00 (ddd, *J* = 13.5, 9.2, 4.5 Hz, 1H), 1.91 (ddd, *J* = 13.1, 10.8, 4.5 Hz, 1H), 1.66 (ddd, *J* = 13.3, 9.2, 4.2 Hz, 1H), 1.12 (d, *J* = 2.1 Hz, 12H), 1.09 (s, 3H), 1.06 (s, 3H), 0.95 (s, 3H).
^{13}C NMR (101 MHz, $CDCl_3$, 258 K) δ 177.71 (CO), 169.58 (CO), 89.88 (C), 80.03 (C), 55.83 (C), 53.85 (C), 45.55 (N-CH_2-B), 37.57 (N-CH_3), 31.24 (CH_2), 25.20 (CH_2), 24.87 (CH_3), 17.19 (CH_3), 16.52 (CH_3), 9.62 (CH_3).
^{11}B NMR (128 MHz, $CDCl_3$, 258 K) δ 13.61.
HRMS (ESI). Calculated for $C_{18}H_{30}BNNaO_5^+$: 374.210924 Obtained: 374.212029

(2,5-dimethylphenoxy)-N,2,2-trimethyl-N-((4,4,5,5-tetramethyl-1,3,2-dioxaborolan-2-yl)methyl)pentanamide (2ai)

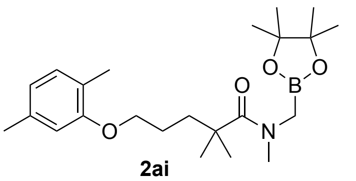

2ai

Following the general procedure: **1ai** (69.4 mg, 0.250 mmol, 1.0 eq.), $[Ir(COD)(OMe)]_2$ (5 mg, 0.008 mmol, 3 mol%), B_2pin_2 (95.2 mg, 0.375 mmol, 1.5 eq.), **L4** (4.4 mg, 0.015 mmol, 6 mol%) in THF (1.25 mL) were heated at 100 °C for 24 h. The crude mixture was purified by column chromatography (Hex/EtOAc 0% - EtOAc 100% - EtOAc/MeOH 20%) to obtain 75.8 mg (75% yield) of **2ai**.

^{1}H NMR (600 MHz, $CDCl_3$, 258 K) δ 7.01 – 6.95 (m, 1H), 6.68 – 6.63 (m, 1H), 6.60 – 6.56 (m, 1H), 3.91 (t, *J* = 5.4 Hz, 2H), 3.21 (s, 3H), 2.47 (s, 2H), 2.29 (s, 3H), 2.13 (s, 3H), 1.87 – 1.75 (m, 4H), 1.33 (s, 6H), 1.17 (s, 12H).
^{13}C NMR (101 MHz, $CDCl_3$, 258 K) δ 180.57 (CO), 156.81 (C, Ar), 136.56 (C, Ar), 130.32 (CH, Ar), 123.34 (C, Ar), 120.82 (CH, Ar), 111.91 (CH, Ar), 79.77 (C), 67.49 (CH2), 47.44 (N-CH2-B), 39.24 (C), 37.44 (N-CH3), 37.35 (CH2), 25.59 (CH3), 25.05 (CH3), 24.91 (CH2), 21.38 (CH3), 15.83 (CH3).
^{11}B NMR (128 MHz, $CDCl_3$, 258 K) δ 12.03
HRMS (ESI). Calculated for $C_{23}H_{39}BNO_4^+$: 404.296666 Obtained: 404.296746

4-(4-(bis(2-chloroethyl)amino)phenyl)-N-methyl-N-((4,4,5,5-tetramethyl-1,3,2-dioxaborolan-2-yl)methyl)butanamide (2aj)

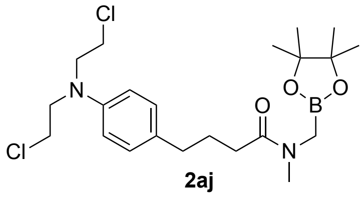

2aj

Following the general procedure: **1aj** (82.8 mg, 0.250 mmol, 1.0 eq.), $[Ir(COD)(OMe)]_2$ (5 mg, 0.008 mmol, 3 mol%), B_2pin_2 (95.2 mg, 0.375 mmol, 1.5 eq.), **L4** (4.4 mg, 0.015 mmol, 6 mol%) in THF (1.25 mL) were heated at 100 °C for 24 h. The crude mixture was purified by column chromatography (Hex/EtOAc 0% - EtOAc 100% - EtOAc/MeOH 20%) to obtain 38 mg (33% yield) of **2aj**.

^{1}H NMR (600 MHz, $CDCl_3$, 258 K) δ 7.04 (d, *J* = 8.6 Hz, 2H), 6.60 (d, *J* = 8.6 Hz, 2H), 3.68 (dd, *J* = 8.1, 6.2 Hz, 4H), 3.64 – 3.57 (m, 4H), 2.98 (s, 3H), 2.57 (t, *J* = 7.5 Hz, 2H), 2.37 (s, 2H), 2.33 (t, *J* = 7.6 Hz, 2H), 2.00 – 1.89 (m, 2H), 1.17 (s, 12H).
^{13}C NMR (101 MHz, $CDCl_3$, 258 K) δ 176.77 (CO), 144.58 (C, Ar), 129.98 (C, Ar), 129.75 (CH, Ar), 112.33 (CH, Ar), 79.99 (C), 53.67 (CH_2), 43.87 (N-CH_2-B), 40.60 (CH_2), 35.73 (N-CH_3), 33.86 (CH_2), 27.64 (CH_2), 26.12 (CH_2), 25.25 (CH_3).
^{11}B NMR (128 MHz, $CDCl_3$, 258 K) δ 12.95
HRMS (ESI). Calculated for $C_{22}H_{36}BCl_2N_2O_3^+$: 457.219055 Obtained: 457.218984

N-(3-phenyl-3-(4-(trifluoromethyl)phenoxy)propyl)-N-((4,4,5,5-tetramethyl-1,3,2-dioxaborolan-2-yl)methyl)hexanamide (2ak)

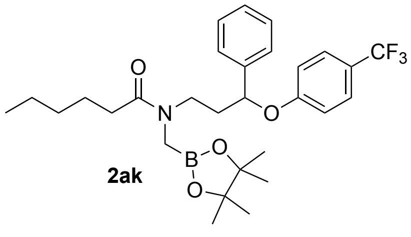

2ak

Following the general procedure: **1ak** (101.9 mg, 0.250 mmol, 1.0 eq.), [Ir(COD)(OMe)]$_2$ (5 mg, 0.008 mmol, 3 mol%), B$_2$pin$_2$ (95.2 mg, 0.375 mmol, 1.5 eq.), **L4** (4.4 mg, 0.015 mmol, 6 mol%) in THF (1.25 mL) were heated at 100 °C for 24 h. The crude mixture was purified by column chromatography (Hex/EtOAc 0% - EtOAc 100% - EtOAc/MeOH 20%) to obtain 76.3 mg (57% yield) of **2ak**.

^{1}H NMR (600 MHz, CDCl$_3$, 258 K) δ 7.33 (d, *J* = 8.7 Hz, 2H), 7.25 – 7.15 (m, 5H), 6.75 (d, *J* = 8.6 Hz, 2H), 5.02 (dd, *J* = 8.9, 3.8 Hz, 1H), 3.64 (dt, *J* = 13.9, 7.8 Hz, 1H), 3.30 (ddd, *J* = 14.0, 7.4, 4.5 Hz, 1H), 2.37 (d, *J* = 13.5 Hz, 1H), 2.21 – 2.03 (m, 5H), 1.52 – 1.36 (m, 2H), 1.09 (s, 12H), 1.06 – 0.94 (m, 4H), 0.71 (t, *J* = 7.0 Hz, 3H).
^{13}C NMR (101 MHz, CDCl$_3$, 258 K) δ 177.55 (CO), 159.84 (C, Ar), 139.64 (C, Ar), 129.18 (CH, Ar), 128.45 (CH, Ar), 127.04 (q, *J* = 3.7 Hz CH, Ar), 125.62 (CH, Ar), 124.31 (q, *J* = 271.1 Hz, CF$_3$), 123.56 (q, *J* = 32.8 Hz, C, Ar), 115.71 (CH, Ar), 80.07 (C), 76.53 (CH), 44.84 (N-CH$_2$), 40.30 (N-CH$_2$-B), 36.26 (CH$_2$), 31.27 (CH$_2$), 28.44 (CH$_2$), 25.24 (CH$_3$), 25.19 (CH$_3$), 24.41 (CH$_2$), 22.14 (CH$_2$), 13.80 (CH$_3$).
^{19}F NMR (376 MHz, CDCl$_3$, 258 K) δ -61.72.
^{11}B NMR (128 MHz, CDCl$_3$, 258 K) δ 13.10
HRMS (ESI). Calculated for $C_{29}H_{40}BF_3NO_4^+$: 534.299700 Obtained: 534.299523

Borylation of aromatic containing substrates with 2,2'-bipyridine (L1)

Following an adapted procedure: In a sealed tube [Ir(COD)(OMe)]$_2$ (5 mg, 0.008 mmol, 3 mol%.), B$_2$pin$_2$ (95.2 mg, 0.375 mmol, 1.5 equiv.), and **L1** (2.3 mg, 0.015 mmol, 6 mol%) were added. The tube was evacuated and refilled with N$_2$ three times, then under a positive flow of N$_2$, dry THF (1.25 mL) and substrate (0.250 mmol, 1.0 equiv.) were added. The tube was then sealed and heated for the specified time. After that, volatiles were removed under vacuum and CH$_2$Br$_2$ (internal standard) was added, the yield was determined by ^{1}H NMR spectroscopy.

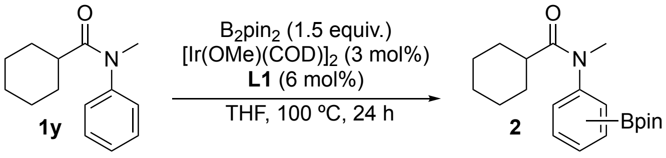

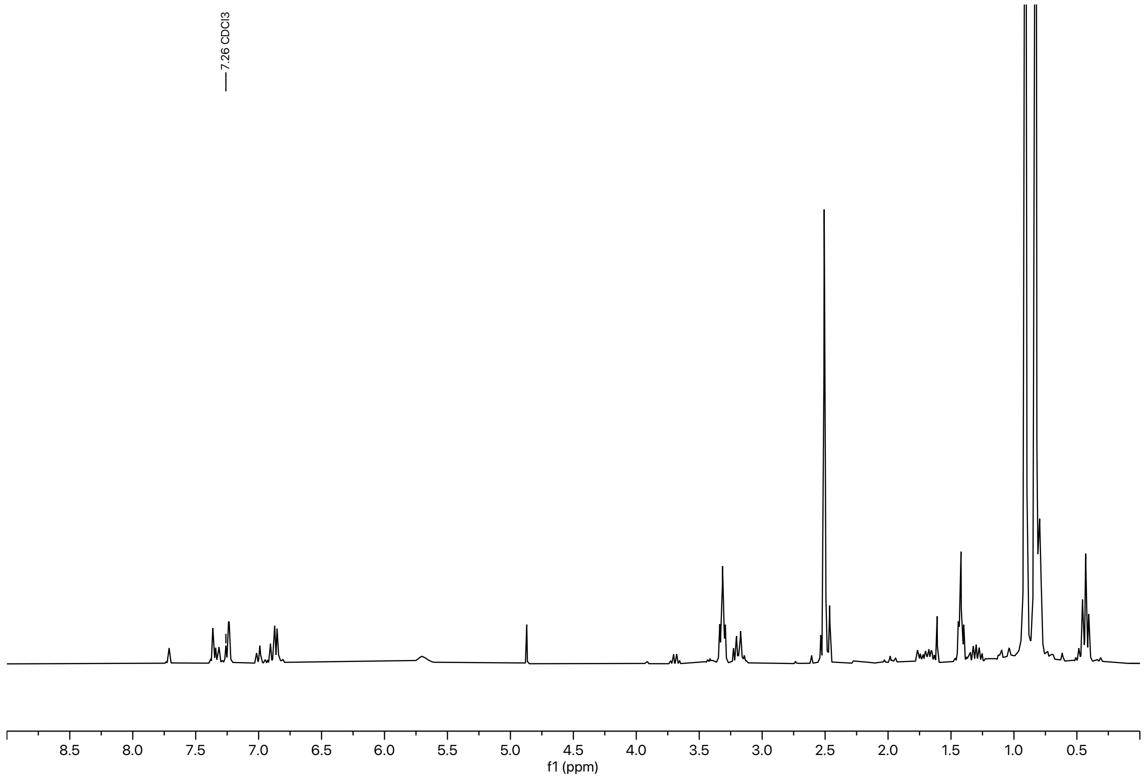

7.26 CDCl3
8.5
8.0
7.5
7.0
6.5
6.0
5.5
5.0
4.5
4.0
3.5
3.0
2.5
2.0
1.5
1.0
0.5
f1 (ppm)

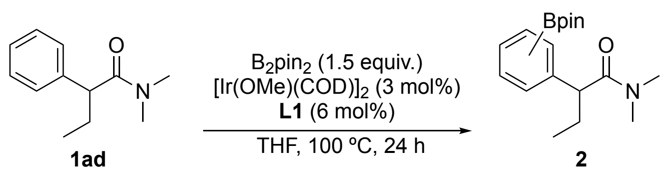

O
N
1ad
B_2pin_2 (1.5 equiv.)
$[Ir(OMe)(COD)]_2$ (3 mol%)
L1 (6 mol%)
THF, 100 ºC, 24 h
Bpin
O
N
2

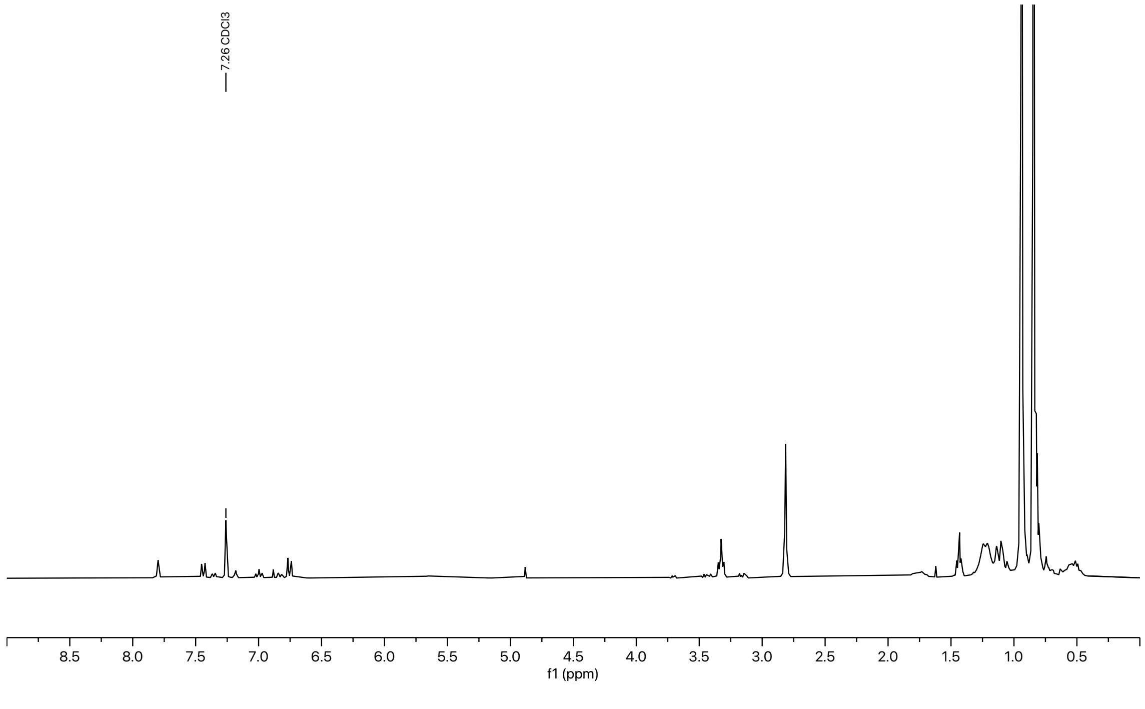

7.26 CDCl3
8.5
8.0
7.5
7.0
6.5
6.0
5.5
5.0
4.5
4.0
3.5
3.0
2.5
2.0
1.5
1.0
0.5
f1 (ppm)

Computational analysis

Calculated Bond Dissociation Energies (BDEs)

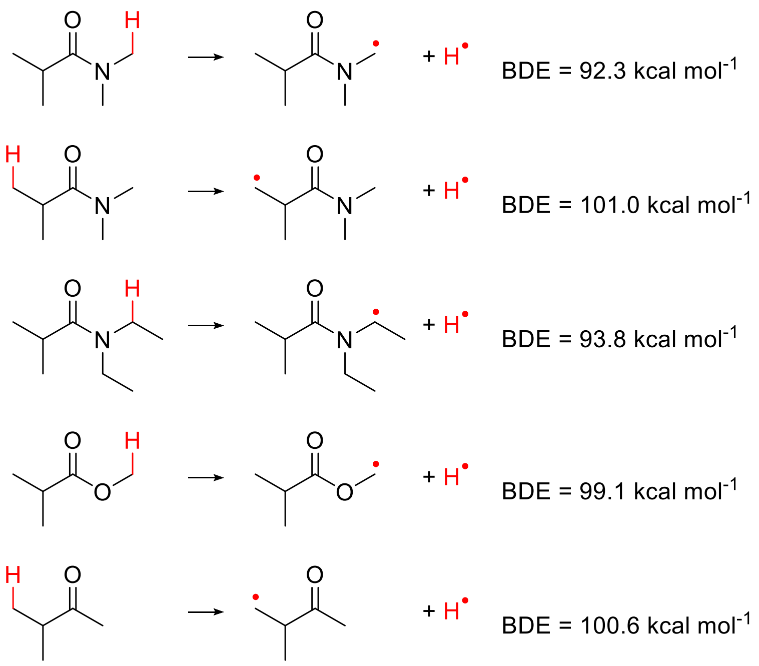

Figure S4. Calculated bond dissociation energies (BDEs, in kcal mol^{-1}) for the C–H bonds highligthed in red.

DFT Profiles and alternate pathways

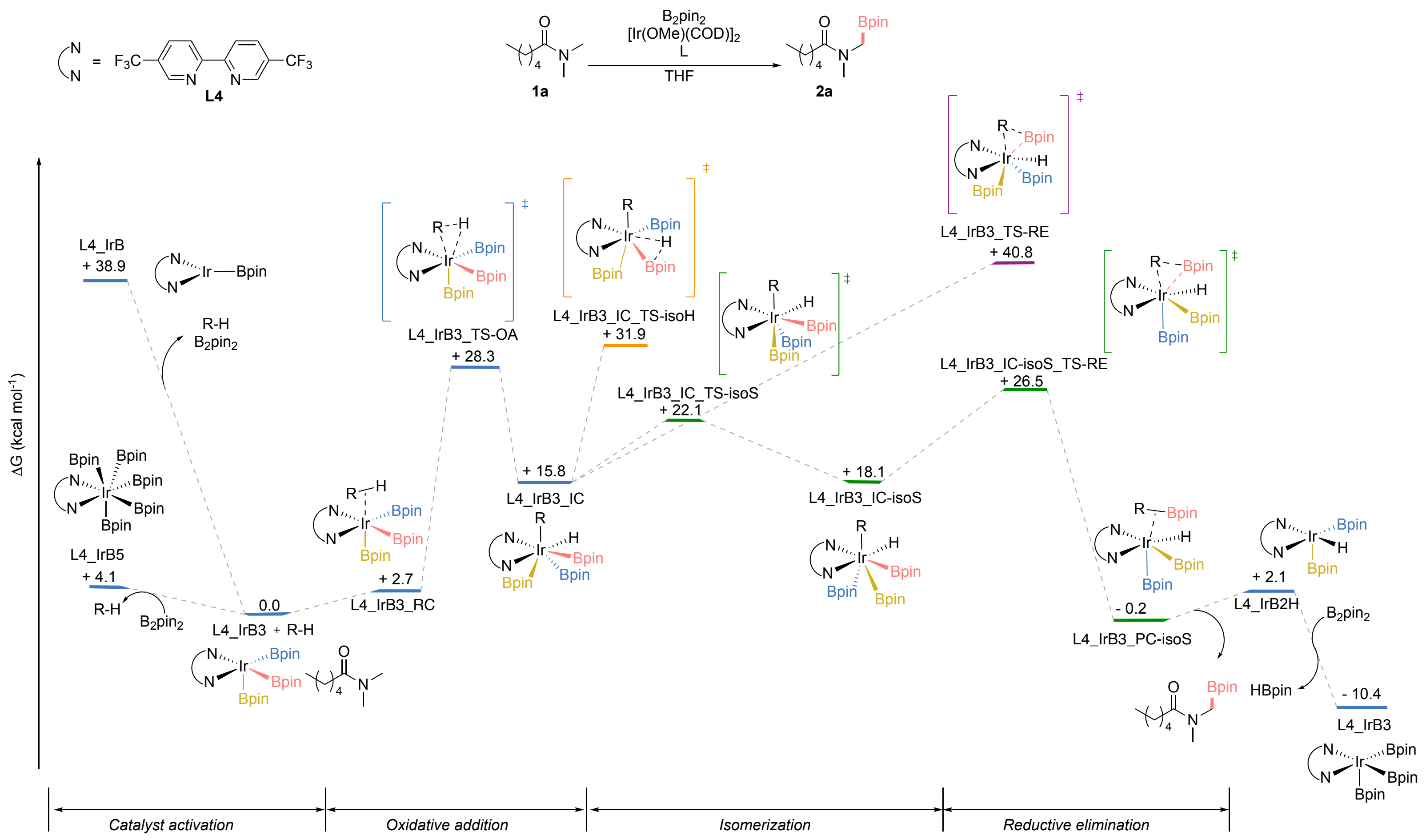

Figure S5. Minimum energy pathways (MEP) calculated with SMD_{THF}/M06/6-311G(d,p);SDD(Ir)]//M06/6-31G(d);LANL2DZ(Ir) for borylation reaction between *N*,*N*-dimethylhexylamide (**1a**) and Ir^{III}(**L4**)$(Bpin)_3$. Free energies relative to structure $(Ir(Bpin)_3$+R–H) (ΔG_{QH} in kcal mol^{-1}).

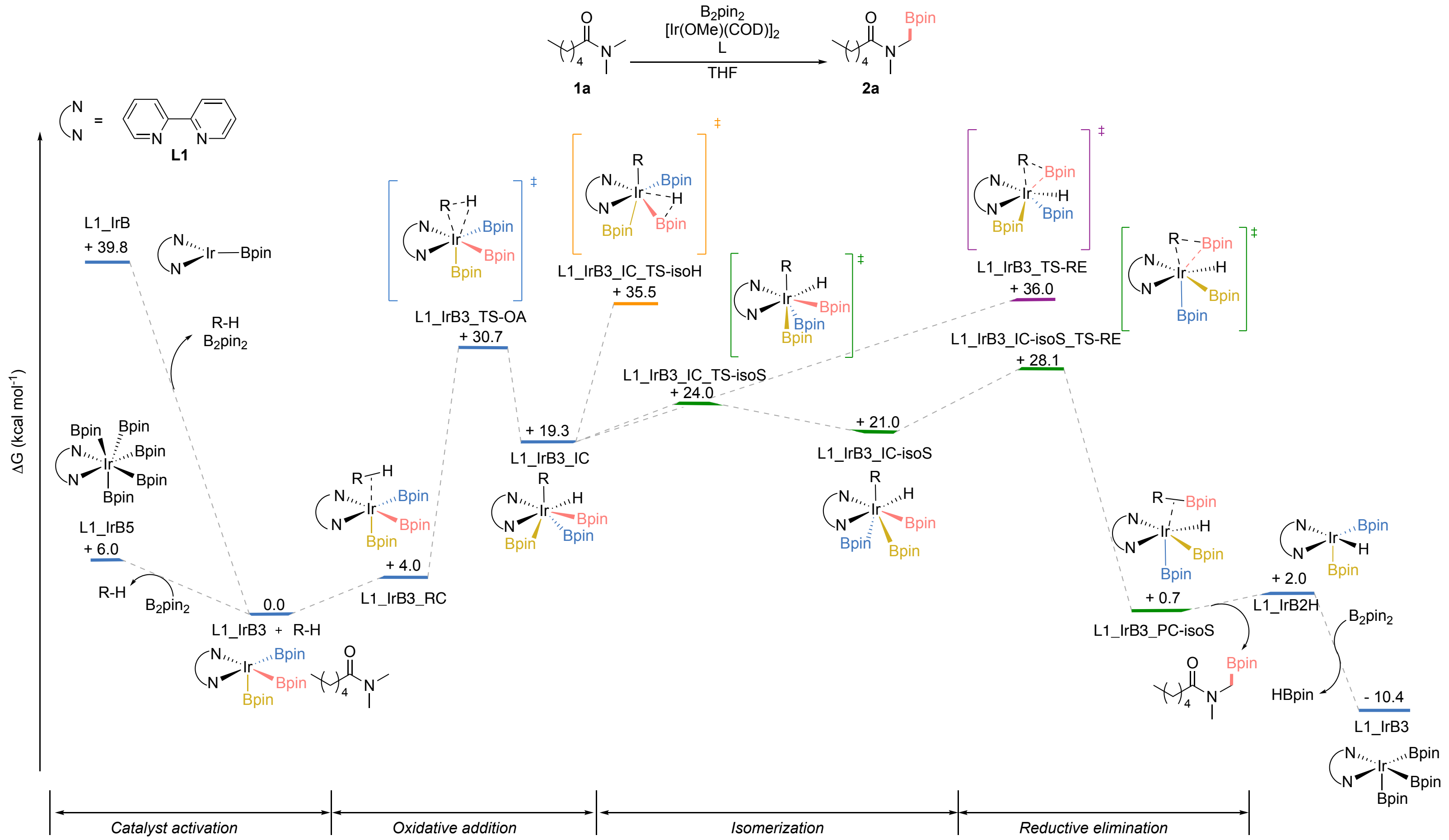

Figure S6. Minimum energy pathways (MEP) calculated with SMD$_{THF}$/M06/6-311G(d,p);SDD(Ir)]//M06/6-31G(d);LANL2DZ(Ir) for borylation reaction between *N*,*N*-dimethylhexylamide (**1a**) and Ir^{III}(**L1**)(Bpin)$_3$. Free energies relative to structure (Ir(Bpin)$_3$+R–H) (ΔG_{QH} in kcal mol^{-1}).

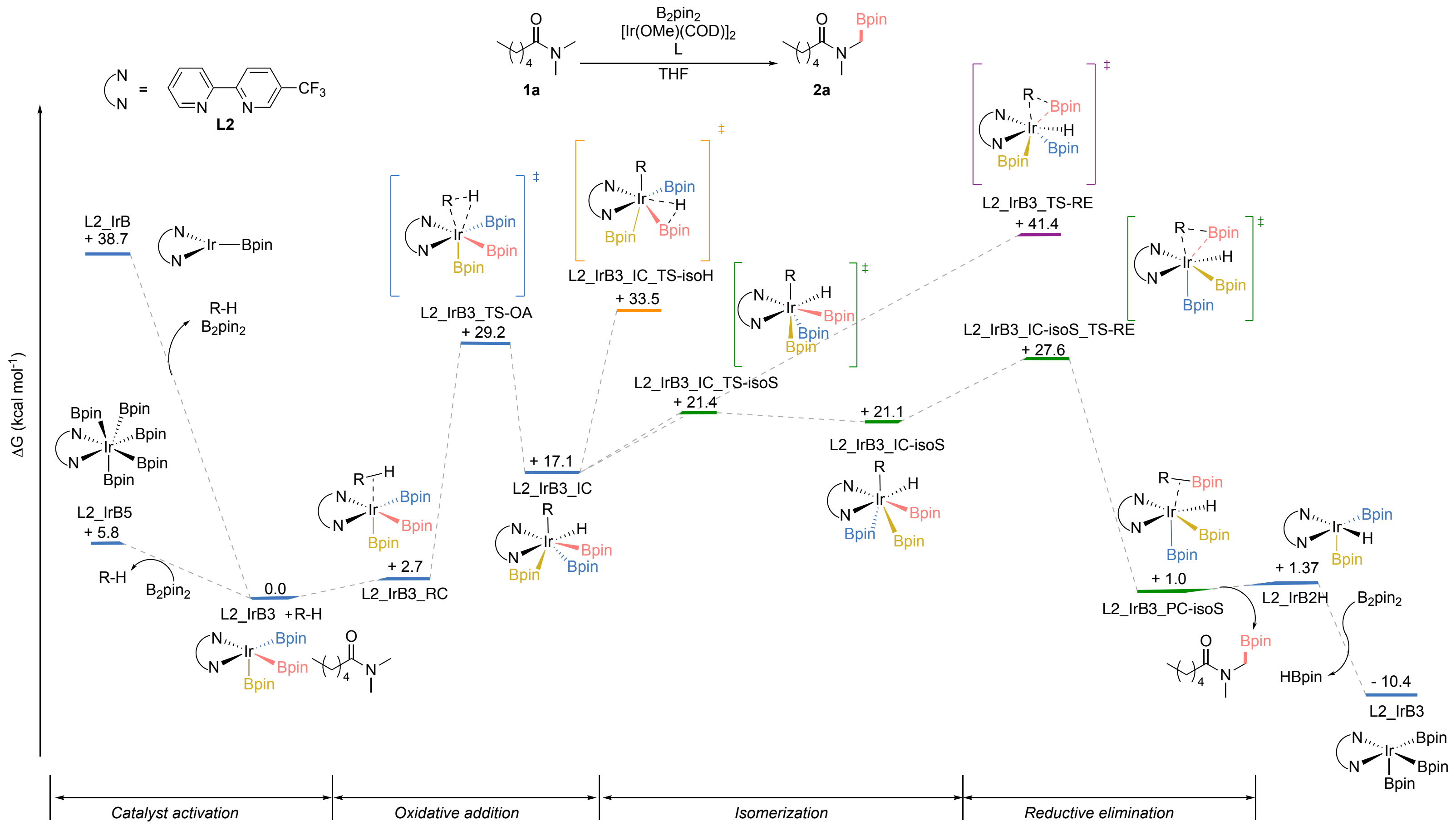

Figure S7. Minimum energy pathways (MEP) calculated with SMD$_{THF}$/M06/6-311G(d,p);SDD(Ir)]//M06/6-31G(d);LANL2DZ(Ir) for borylation reaction between *N*,*N*-dimethylhexylamide (**1a**) and Ir^{III}(**L2**)(Bpin)$_3$. Free energies relative to structure (Ir(Bpin)$_3$+R–H) (ΔG$_{QH}$ in kcal mol^{-1}).

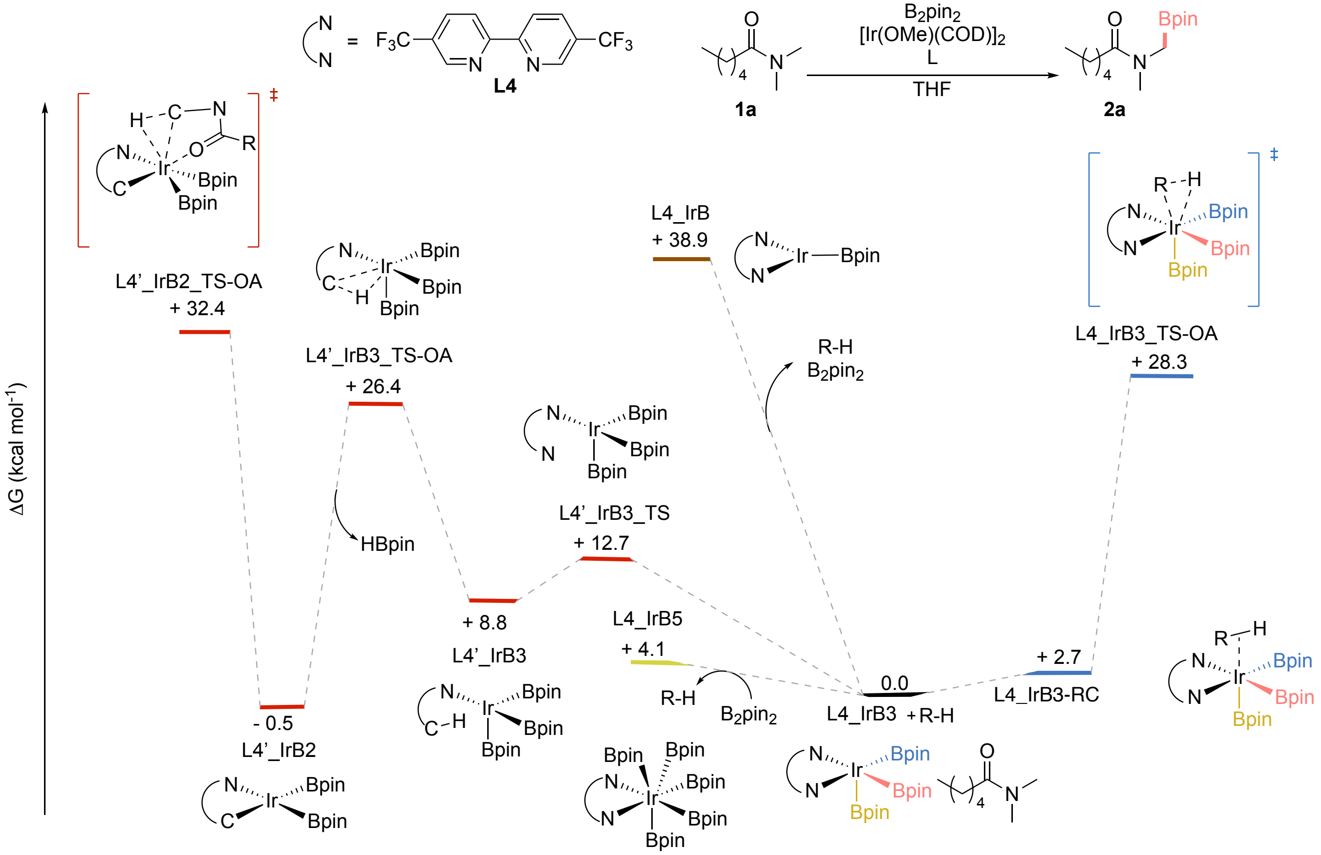

Figure S8. Minimum energy pathways (MEP) calculated with SMD$_{THF}$/M06/6-311G(d,p);SDD(Ir)]//M06/6-31G(d);LANL2DZ(Ir) for the oxidative addition step between *N*,*N*-dimethylhexylamide (**1a**) and Ir^{III}(**L4**)(Bpin)$_3$. Several mechanisms are compared: the canonical Ir(III) → Ir(V) involving a 16 e^- active catalyst (Ir(Bpin)$_3$), an alternative Ir(I) → Ir(III) mechanism involving a 14 e^- active catalyst (Ir(Bpin)) after B_2pin_2 cleavage and a mechanism involving the de-coordination of one pyridine ring followed via a rollover cyclometalation to form a L–X ligand which would be the active catalyst (Ir(LX)(Bpin)$_2$) in this scenario. Free energies relative to (Ir(Bpin)$_3$+R–H) (ΔG_{QH} in kcal mol^{-1}).

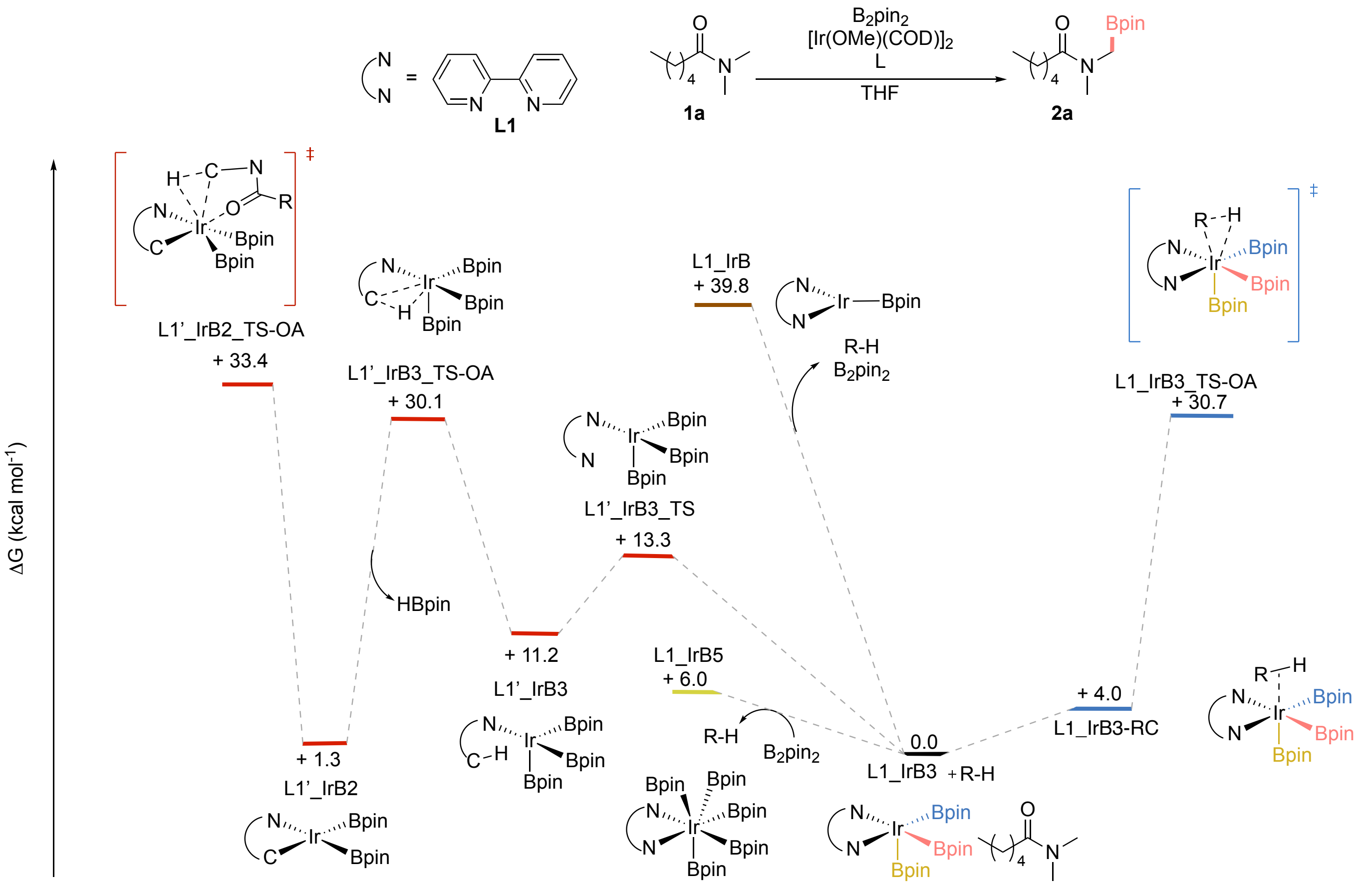

Figure S9. Minimum energy pathways (MEP) calculated with SMD$_{THF}$/M06/6-311G(d,p);SDD(Ir)]//M06/6-31G(d);LANL2DZ(Ir) for the oxidative addition step between *N*,*N*-dimethylhexylamide (**1a**) and Ir^{III}(**L1**)(Bpin)$_3$. Several mechanisms are compared: the canonical Ir(III) → Ir(V) involving a 16 e$^-$ active catalyst (Ir(Bpin)$_3$), an alternative Ir(I) → Ir(III) mechanism involving a 14 e$^-$ active catalyst (Ir(Bpin)) after B_2pin_2 cleavage and a mechanism involving the de-coordination of one pyridine ring followed via a rollover cyclometalation to form a L–X ligand which would be the active catalyst (Ir(LX)(Bpin)$_2$) in this scenario. Free energies relative to (Ir(Bpin)$_3$+R–H) (ΔG$_{QH}$ in kcal mol^{-1}).

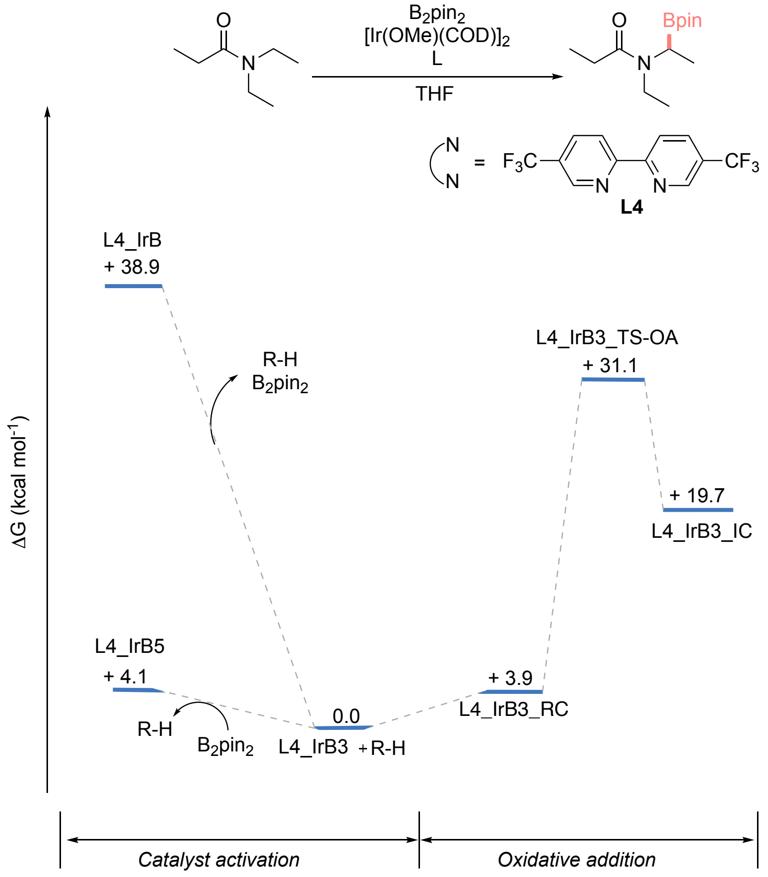

Figure S10. Minimum energy pathways (MEP) calculated with SMD_{THF}/M06/6-311G(d,p);SDD(Ir)]//M06/6-31G(d);LANL2DZ(Ir) for the oxidative addition step between *N*,*N*-diethylpropionamide (**1u**) and Ir^{III}(**L4**)$(Bpin)_3$. Free energies relative to $(Ir(Bpin)_3$+R–H) (ΔG_{QH} in kcal mol^{-1}).

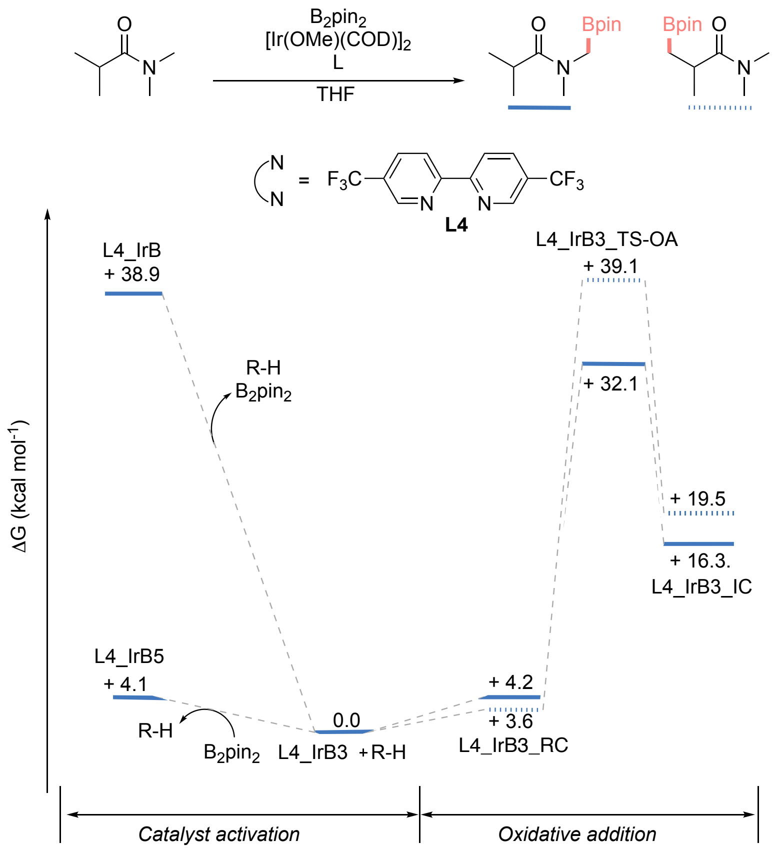

Figure S11. Minimum energy pathways (MEP) calculated with SMD_{THF}/M06/6-311G(d,p);SDD(Ir)]//M06/6-31G(d);LANL2DZ(Ir) for the oxidative addition step between *N,N*-dimethylisobutyramide (**1f**) and Ir^{III}(**L4**)(Bpin)$_3$. C–H activation at the N-CH_3 (solid lines) and C-CH_3 (dashed lines) positions of **1f** (topologically identical relative to the carbonyl group) are compared. Free energies relative to (Ir(Bpin)$_3$+R–H) (ΔG_{QH} in kcal mol^{-1}).

Non-Covalent Interactions (NCI) analysis

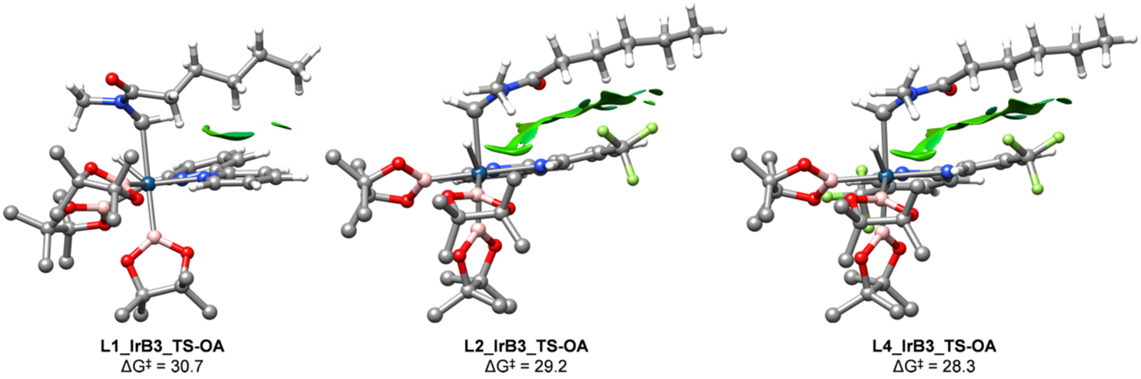

Figure S12. Substrate-ligand non-covalent interactions (NCI) occurring in low energy outer-sphere transition states (**TS**) calculated for the oxidative addition step between *N*,*N*-dimethylhexanamide (**1a**) and Ir^{III}(ligand)$(Bpin)_3$ (ligand = **L1, L2** and **L4**). Activation free energies ($\Delta G^{\ddagger}$) calculated with SMD_{THF}/M06/6-311G(d,p);SDD(Ir)]//M06/6-31G(d);LANL2DZ(Ir), are given in kcal mol^{-1}. Reduced density gradient (RDG) isosurfaces are plotted at a 0.25 level and color-coded based on sign(λ2)ρ: –3.0 (blue; strong attractive interactions) < 0.0 (green; weak van der Waals interactions) < 3.0 (red; strong repulsive interactions). Note the larger extension of non-covalent interactions in the borylation TS with 5-trifluoromethylated ligands **L2** and **L4**. Hydrogens of the Bpin ligands have been omitted for clarity

Energy Decomposition Analysis (EDA)

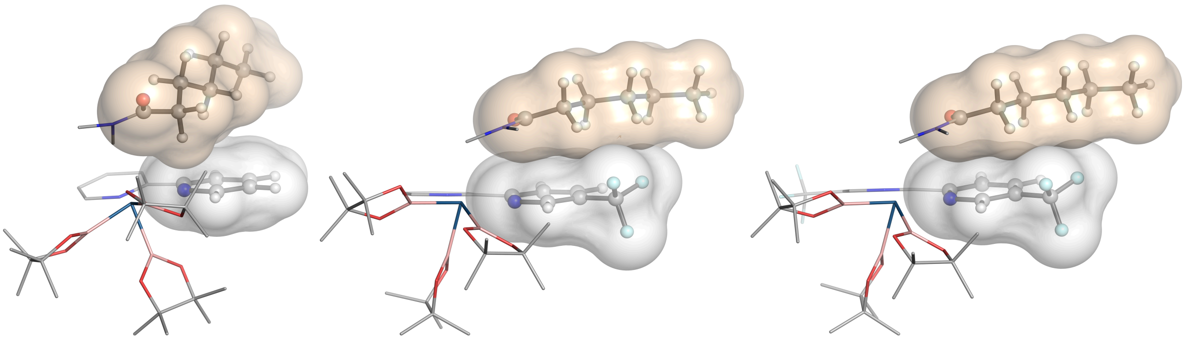

L1_IrB3_TS-OA ΔG‡ = 30.7 | **L2_IrB3_TS-OA** ΔG‡ = 29.2 | **L4_IrB3_TS-OA** ΔG‡ = 28.3

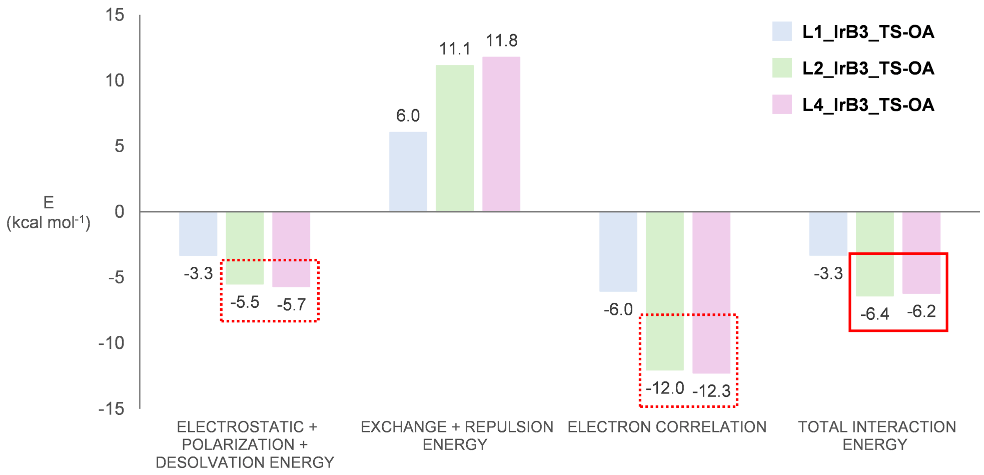

Figure S13. Generalized Kohn-Sham Energy Decomposition Analysis (GKS-EDA of the low energy outer-sphere transition states (**TS**) calculated for the oxidative addition step between *N*,*N*-dimethylhexanamide (**1a**) and Ir^{III}(ligand)(Bpin)$_3$ (ligand = **L1, L2** and **L4**). Activation free energies (ΔG‡) calculated with SMD_{THF}/M06/6-311G(d,p);SDD(Ir)]//M06/6-31G(d);LANL2DZ(Ir), are given in kcal mol^{-1}. In the graphs, different contributions to the total interaction energy between the *n*- hexanamide and (5-trifluoromethyl)pyridine fragments are shown. Contributions related to the polar character of the interactions (electrostatic, polarization and desolvation) have been added together for clarity. **The electron correlation component implicitly accounts for dispersion**. The larger values for the attractive interactions (both polar and especially dispersion) occurring in the borylation TS with 5-trifluoromethylated ligands **L2** and **L4**, resulting in a larger total interaction energy, are highlighted with red boxes.

Pentagonal bipyramidal structures of key intermediates

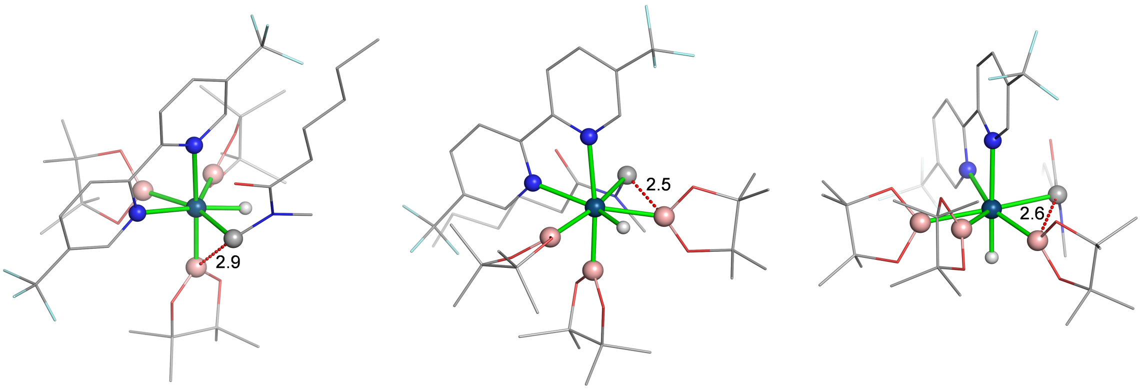

**L4_IrB3_IC**	**L4_IrB3_IC-isoH**	**L4_IrB3_IC-isoS**
28.3	31.9	18.1

Figure S14. Lowest energy conformers calculated with SMD_{THF}/M06/6-311G(d,p);SDD(Ir)]//M06/6-31G(d);LANL2DZ(Ir), for the intermediate complexes (**IC**) in the borylation of *N*,*N*-dimethylhexanamide (**1a**) catalyzed by Ir^{III}(**L4**)$(Bpin)_3$, right after oxidative addition (**OA**) and before reductive elimination (**RE**). The pentagonal bipyramid coordination around Ir is highlighted with green lines. Note the shorter distance (in angstrom; red dashed lines) between the reacting B and C atoms in the subsequent RE step when both ligands occupy equatorial positions (**isoH** and **isoS** structures). **B**: Bpin. Non-reactive hydrogens have been omitted for clarity. Free energies (ΔG in kcal mol^{-1}) relative to the isolated reactants (**L4_IrB3** + **1a**) are shown.

Calculated structures

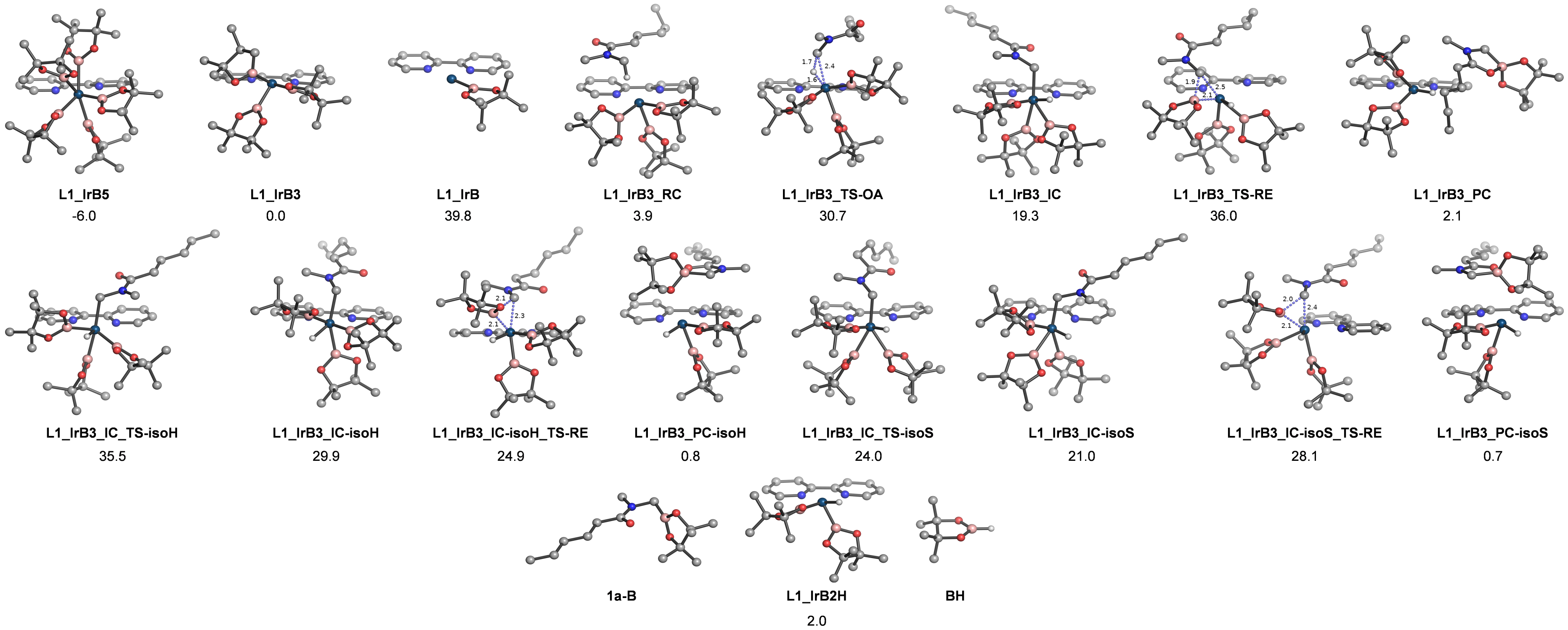

Figure S15. Lowest energy conformers calculated with SMD_{THF}/M06/6-311G(d,p);SDD(Ir)]//M06/6-31G(d);LANL2DZ(Ir), for the borylation of *N*,*N*-dimethylhexanamide (**1a**) catalyzed by Ir^{III}(**L1**)$(Bpin)_3$. **B**: Bpin; **RC**: reactive complex; **IC**: intermediate complex; **PC**: product complex. Non-reactive hydrogens have been omitted for clarity. Free energies (ΔG in kcal mol^{-1}) relative to the isolated reactants (**L1_IrB3** + **1a**) are shown.

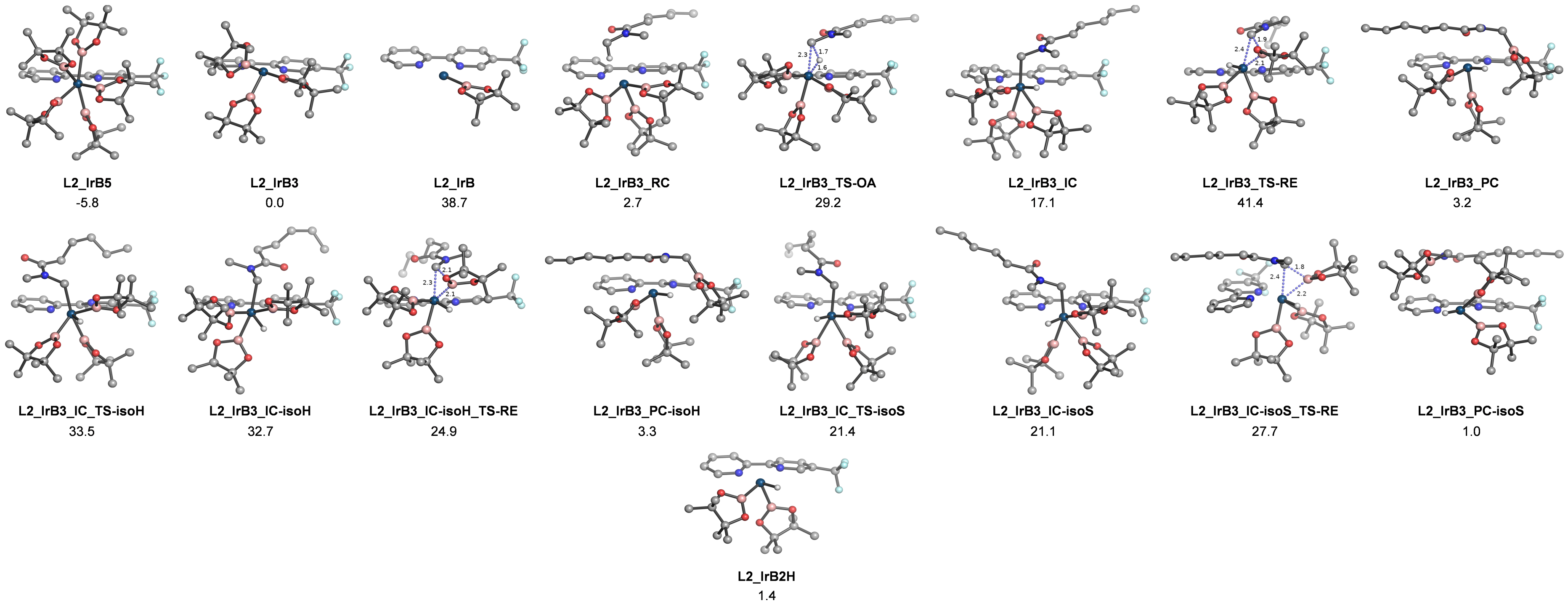

Figure S16. Lowest energy conformers calculated with SMD_{THF}/M06/6-311G(d,p);SDD(Ir)]//M06/6-31G(d);LANL2DZ(Ir), for the borylation of *N*,*N*-dimethylhexanamide (**1a**) catalyzed by Ir^{III}(**L2**)(Bpin)$_3$. **B**: Bpin; **RC**: reactive complex; **IC**: intermediate complex; **PC**: product complex. Non-reactive hydrogens have been omitted for clarity. Free energies (ΔG in kcal mol^{-1}) relative to the isolated reactants (**L2_IrB3** + **1a**) are shown.

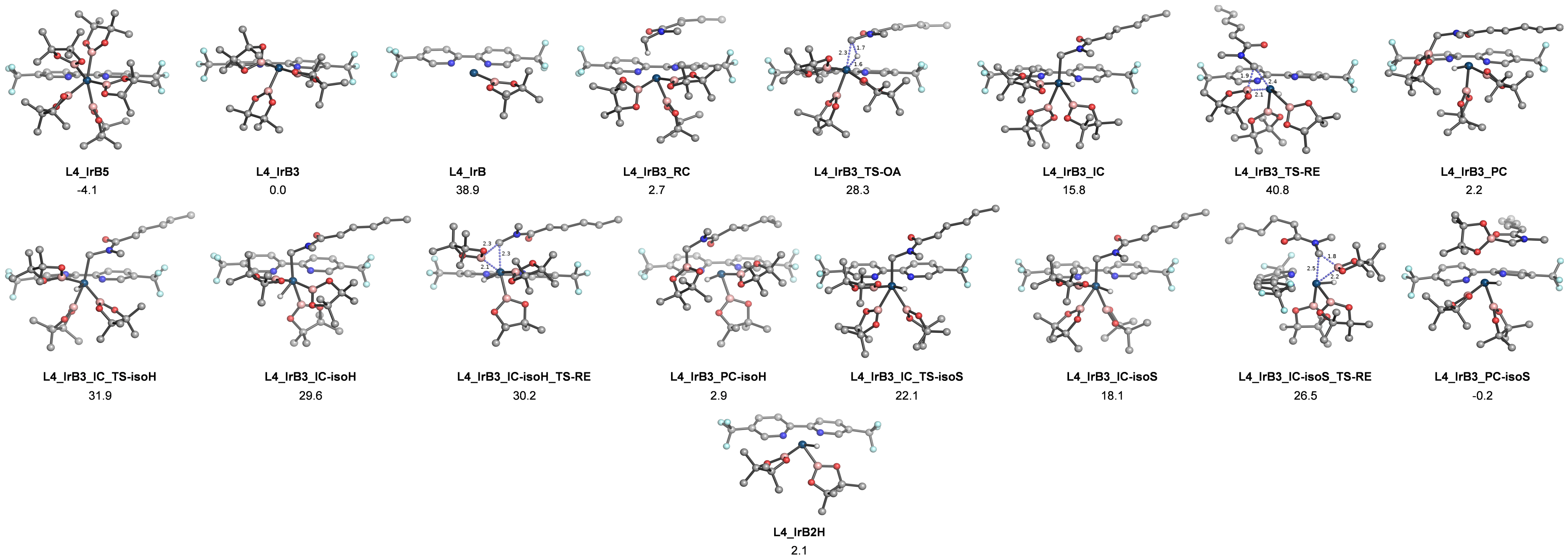

Figure S17. Lowest energy conformers calculated with SMD_{THF}/M06/6-311G(d,p);SDD(Ir)]//M06/6-31G(d);LANL2DZ(Ir), for the borylation of *N*,*N*-dimethylhexanamide (**1a**) catalyzed by Ir^{III}(**L4**)(Bpin)$_3$. **B**: Bpin; **RC**: reactive complex; **IC**: intermediate complex; **PC**: product complex. Non-reactive hydrogens have been omitted for clarity. Free energies (ΔG in kcal mol^{-1}) relative to the isolated reactants (**L4_IrB3** + **1a**) are shown.

Energies, entropies, and lowest frequencies of optimized structures

Table S2. Energies, entropies, and lowest frequencies of the lowest energy calculated structures for borylation reactions.[a]

Structure	E_{elec} (Hartree)[b]	E_{elec} + ZPE (Hartree)[c]	H (Hartree)[d]	S (cal mol^{-1} K^{-1})[e]	G (Hartree)[f]	Lowest freq. (cm^{-1})[e]	# of imag. freq.
**B2**	–822.257076	–821.892793	–821.872410	141.6	–821.936152	15.2	0
**1a**	–444.892150	–444.648080	–444.634110	114.4	–444.686478	39.2	0
**L1_IrB5**	–2655.273208	–2654.200570	–2654.138316	316.9	–2654.280001	23.8	0
**L1_IrB3**	–1832.995480	–1832.289387	–1832.246638	245.3	–1832.353381	16.0	0
**L1_IrB**	–1010.648393	–1010.307169	–1010.285419	153.2	–1010.353770	18.3	0
**L1_IrB3_RC**	–2277.909321	–2276.957291	–2276.900514	311.8	–2277.033570	12.3	0
**L1_IrB3_TS-OA**	–2277.864853	–2276.916024	–2276.860836	296.4	–2276.990991	–778.0	1
**L1_IrB3_IC**	–2277.884368	–2276.933964	–2276.878539	297.8	–2277.009084	9.7	0
**L1_IrB3_TS-RE**	–2277.858586	–2276.908367	–2276.853975	288.6	–2276.982427	–138.1	1
**L1_IrB3_PC**	–2277.912788	–2276.961584	–2276.906295	301.5	–2277.036536	15.2	0
**L1_IrB3_IC_TS-isoH**	–2277.858145	–2276.908552	–2276.853295	300.7	–2276.983257	–26.6	1
**L1_IrB3_IC-isoH**	–2277.878395	–2276.927951	–2276.872773	295.4	–2276.992141	18.9	0
**L1_IrB3_IC-isoH_TS-RE**	–2277.876219	–2276.926376	–2276.871627	295.4	–2277.000238	–71.9	1
**L1_IrB3_PC-isoH**	–2277.915494	–2276.964103	–2276.909250	294.4	–2277.038570	21.2	0
**L1_IrB3_IC_TS-isoS**	–2277.878854	–2276.927709	–2276.873615	288.0	–2277.001541	–16.8	1
**L1_IrB3_IC-isoS**	–2277.881862	–2276.931397	–2276.876034	297.7	–2277.006409	14.2	0
**L1_IrB3_IC-isoS_TS-RE**	–2277.870378	–2276.920363	–2276.865451	296.2	–2276.995032	–49.2	1
**L1_IrB3_PC-isoS**	–2277.915437	–2276.964229	–2276.909210	297.2	–2277.038773	16.4	0
**1a-B**	–855.441634	–855.025196	–855.001799	160.4	–855.072610	10.9	0
**L1_IrB2H**	–1422.440074	–1421.908467	–1421.875779	203.4	–1421.964017	8.7	0
**BH**	–411.724734	–411.533796	–411.523489	90.8	–411.566608	116.3	0
**L2_IrB5**	–2992.308747	–2991.229718	–2991.164407	329.3	–2991.312079	25.8	0
**L2_IrB3**	–2170.029339	–2169.318016	–2169.271591	266.7	–2169.385198	9.1	0
**L2_IrB**	–1347.683615	–1347.336969	–1347.311590	171.7	–1347.387377	19.5	0
**L2_IrB3_RC**	–2614.945996	–2613.988078	–2613.928343	319.2	–2614.067330	19.1	0
**L2_IrB3_TS-OA**	–2614.902578	–2613.947476	–2613.889051	310.0	–2614.025139	–770.0	1
**L2_IrB3_IC**	–2614.923530	–2613.966902	–2613.908260	313.2	–2614.044399	11.3	0
**L2_IrB3_TS-RE**	–2614.894253	–2613.938665	–2613.880792	305.6	–2614.005662	–150.9	1
**L2_IrB3_PC**	–2614.945212	–2613.988315	–2613.929421	316.0	–2614.066577	12.9	0
**L2_IrB3_IC_TS-isoH**	–2614.896805	–2613.940566	–2613.882473	306.3	–2614.018263	–74.2	1
**L2_IrB3_IC-isoH**	–2614.909936	–2613.953177	–2613.894701	309.5	–2614.019613	18.9	0

**L2_IrB3_IC-isoH_TS-RE**	–2614.910845	–2613.954989	–2613.896883	309.8	–2614.031997	–92.3	1
**L2_IrB3_PC-isoH**	–2614.945870	–2613.988207	–2613.929697	310.3	–2614.066491	19.0	0
**L2_IrB3_IC_TS-isoS**	–2614.916525	–2613.960219	–2613.902625	302.6	–2614.037586	–11.5	1
**L2_IrB3_IC-isoS**	–2614.915844	–2613.959664	–2613.900743	315.1	–2614.038015	13.0	0
**L2_IrB3_IC-isoS_TS-RE**	–2614.905224	–2613.949663	–2613.891230	313.3	–2614.027577	–65.5	1
**L2_IrB3_PC-isoS**	–2614.951201	–2613.992460	–2613.934499	303.5	–2614.070075	29.9	0
**1a-B**	–855.441634	–855.025196	–855.001799	160.4	–855.072610	10.9	0
**L2_IrB2H**	–1759.476118	–1758.938198	–1758.902509	213.7	–1758.996882	16.4	0
**L4_IrB5**	–3329.343838	–3328.259496	–3328.190378	350.1	–3328.345135	14.3	0
**L4_IrB3**	–2507.062902	–2506.345409	–2506.295857	279.0	–2506.415568	13.4	0
**L4_IrB**	–1684.715231	–1684.363582	–1684.334521	191.6	–1684.417372	17.2	0
**L4_IrB3_RC**	–2951.979175	–2951.015481	–2950.952236	336.9	–2951.097738	14.2	0
**L4_IrB3_TS-OA**	–2951.938204	–2950.976480	–2950.914999	322.5	–2951.057011	–771.3	1
**L4_IrB3_IC**	–2951.958323	–2950.996097	–2950.934085	326.3	–2951.076868	17.9	0
**L4_IrB3_TS-RE**	–2951.928824	–2950.967300	–2950.906127	319.9	–2951.036960	–152.4	1
**L4_IrB3_PC**	–2951.980531	–2951.017305	–2950.955072	331.2	–2951.098510	14.4	0
**L4_IrB3_IC_TS-isoH**	–2951.932426	–2950.970199	–2950.908636	323.7	–2951.051148	–75.5	1
**L4_IrB3_IC-isoH**	–2951.947966	–2950.985204	–2950.923555	322.2	–2951.054946	19.7	0
**L4_IrB3_IC-isoH_TS-RE**	–2951.947940	–2950.985725	–2950.924722	319.9	–2951.053910	–59.1	1
**L4_IrB3_PC-isoH**	–2951.978910	–2951.016209	–2950.953992	331.3	–2951.097379	13.8	0
**L4_IrB3_IC_TS-isoS**	–2951.949692	–2950.986562	–2950.925597	319.7	–2951.066777	–7.7	1
**L4_IrB3_IC-isoS**	–2951.954020	–2950.991834	–2950.929670	326.7	–2951.073136	16.5	0
**L4_IrB3_IC-isoS_TS-RE**	–2951.941742	–2950.979352	–2950.918282	319.5	–2951.059791	–77.0	1
**L4_IrB3_PC-isoS**	–2951.984559	–2951.021765	–2950.960002	326.9	–2951.102358	20.3	0
**1a-B**	–855.441634	–855.025196	–855.001799	160.4	–855.072610	10.9	0
**L4_IrB2H**	–2096.506517	–2095.963573	–2095.924143	232.7	–2096.026023	14.3	0

[a] 1 Hartree = 627.51 kcal mol^{-1}. Thermal corrections at 298.15 K.
[b] Calculated with SMD_{THF}/M06/6-311G(2d,p);SDD(Ir).
[c] Calculated as E_{elec}[SMD_{THF}/M06/6-311G(d,p);SDD(Ir)] + ZPE[M06/6-31G(d);LANL2DZ(Ir)], where ZPE is the zero-point energy obtained in the gas phase.
[d] Calculated as E_{elec}[SMD_{THF}/M06/6-311G(d,p);SDD(Ir)] + H_{corr}[M06/6-31G(d);LANL2DZ(Ir)], where H_{corr} is the thermal correction to enthalpy obtained in the gas phase.
[e] Calculated with M06/6-31G(d);LANL2DZ(Ir).
[f] Calculated as E_{elec}[SMD_{THF}/M06/6-311G(d,p);SDD(Ir)] + G_{corr}[M06/6-31G(d);LANL2DZ(Ir)], where G_{corr} is the thermal correction to Gibbs free energy obtained in the gas phase.

Table S3. Energies, entropies, and lowest frequencies of the lowest energy structures for BDE calculations.[a]

Structure	E_{elec} (Hartree)[b]	E_{elec} + ZPE (Hartree)[c]	H (Hartree)[d]	S (cal mol^{-1} K^{-1})[e]	G (Hartree)[f]	Lowest freq. (cm^{-1})[e]	# of imag. freq.
**H·**	–0.499821	–0.499821	–0.497461	27.4	–0.510475		
**iPrNMe2**	–365.730282	–365.541885	–365.530730	97.4	–365.576375	61.9	0
**iPrNMe2_N·**	–365.069171	–364.894949	–364.883706	98.2	–364.929898	60.6	0
**iPrNMe2_C·**	–365.054563	–364.881076	–364.869586	100.1	–364.916596	61.5	0
**iPrNEt2**	–444.189180	–443.943075	–443.929639	108.7	–443.980443	60.6	0
**iPrNEt2_N·**	–443.525955	–443.293842	–443.280318	109.9	–443.331590	52.8	0
**iPrOMe**	–346.373028	–346.225066	–346.215577	90.0	–346.257396	36.4	0
**iPrOMe_N·**	–345.700288	–345.567248	–345.557642	91.0	–345.600086	42.7	0
**iPrCO**	–271.241068	–271.099416	–271.090709	84.9	–271.130532	56.3	0
**iPrCO_C·**	–270.566047	–270.439324	–270.430269	87.9	–270.471467	54.8	0

[a] 1 Hartree = 627.51 kcal mol^{-1}. Thermal corrections at 298.15 K.

[b] Calculated with SCS-MP2/aug-cc-pVTZ.

[c] Calculated as E_{elec}[SCS-MP2/aug-cc-pVTZ] + ZPE[M06-2X/6-311+G(2d,p)], where ZPE is the zero-point energy.

[d] Calculated as E_{elec}[SCS-MP2/aug-cc-pVTZ] + H_{corr}[M06-2X/6-311+G(2d,p)], where H_{corr} is the thermal correction to enthalpy.

[e] Calculated with M06-2X/6-311+G(2d,p).

[f] Calculated as E_{elec}[SCS-MP2/aug-cc-pVTZ] + G_{corr}[M06-2X/6-311+G(2d,p)], where G_{corr} is the thermal correction to Gibbs free energy.

References

1. M. J. Frisch, G. W. Trucks, H. B. Schlegel, G. E. Scuseria, M. A. Robb, J. R. Cheeseman, G. Scalmani, V. Barone, G. A. Petersson, H. Nakatsuji, X. Li, M. Caricato, A. V. Marenich, J. Bloino, B. G. Janesko, R. Gomperts, B. Mennucci, H. P. Hratchian, J. V. Ortiz, A. F. Izmaylov, J. L. Sonnenberg, D. Williams-Young, F. Ding, F. Lipparini, F. Egidi, J. Goings, B. Peng, A. Petrone, T. Henderson, D. Ranasinghe, V. G. Zakrzewski, J. Gao, N. Rega, G. Zheng, W. Liang, M. Hada, M. Ehara, K. Toyota, R. Fukuda, J. Hasegawa, M. Ishida, T. Nakajima, Y. Honda, O. Kitao, H. Nakai, T. Vreven, K. Throssell, J. A. Montgomery Jr., J. E. Peralta, F. Ogliaro, M. J. Bearpark, J. J. Heyd, E. N. Brothers, K. N. Kudin, V. N. Staroverov, T. A. Keith, R. Kobayashi, J. Normand, K. Raghavachari, A. P. Rendell, J. C. Burant, S. S. Iyengar, J. Tomasi, M. Cossi, J. M. Millam, M. Klene, C. Adamo, R. Cammi, J. W. Ochterski, R. L. Martin, K. Morokuma, O. Farkas, J. B. Foresman, D. J. Fox, *Gaussian 16*, Revision C.01. Gaussian, Inc., Wallingford CT, 2016.
2. Y. Zhao, D. G. Truhlar, *Theor. Chem. Acc.* **2007**, *120*, 215–241.
3. P. C. Hariharan, J. A. Pople, *Theor. Chim. Acta* **1973**, *28*, 213–222.
4. W. J. Hehre, K. Ditchfield, J. A. Pople, *J. Chem. Phys.* **2003**, *56*, 2257–2261.
5. P. J. Hay, W. R. Wadt, *J. Chem. Phys.* **1998**, *82*, 299–310.
6. R. F. Ribeiro, A. V. Marenich, C. J. Cramer, D. G. Truhlar, *J. Phys. Chem. B* **2011**, *115*, 14556–14562.
7. H. P. Hratchian, H. B. Schlegel, *J. Chem. Phys.* **2004**, *120*, 9918–9924.
8. H. P. Hratchian, H. B. Schlegel, *J. Chem. Theory Comput.* **2005**, *1*, 61–69.
9. R. Krishnan, J. S. Binkley, R. Seeger, J. A. Pople, *J. Chem. Phys.* **1980**, *72*, 650–654.
10. D. Andrae, U. Hausermann, M. Dolg, H. Stoll, H. Preus, *Theor. Chim. Acta* **1990**, *77*, 123–141.
11. A. V. Marenich, C. J. Cramer, D. G. Truhlar, *J. Phys. Chem. B* **2009**, *113*, 6378–6396.
12. M. Gerenkamp, S. Grimme, *Chem. Phys. Lett.* **2004**, *392*, 229–235.
13. T. H. Dunning, *J. Chem. Phys.*, **1989**, *90*, 1007–1023.
14. R. Paton, *Patonlab/Kinisot: V2.0.1*, 2022. https://doi.org/10.5281/ZENODO.6831009.
15. E. R. Johnson, S. Keinan, P. Mori-Sanchez, J. Contreras-Garcia, A. J. Cohen, W. Yang, *J. Am. Chem. Soc.* **2010**, *132*, 6498–6506.
16. J. Contreras-Garcia, E. R. Johnson, S. Keinan, R. Chaudret, J. P. Piquemal, D. N. Beratan, W. Yang, *J. Chem. Theory Comput.* **2011**, *7*, 625–632.
17. P. Su, Z. Tang, W. Wu, *WIREs Comput. Mol. Sci.* **2020**, *10*, e1460.
18. Z. Tang, Y. Song, S. Zhang, W. Wang, Y. Xu, D. Wu, W. Wu, P. Su, *J. Comput. Chem.* **2021**, *42*, 2341–2351.

19. G. M. J. Barca, C. Bertoni, L. Carrington, D. Datta, N. De Silva, J. E. Deustua, D. G. Fedorov, J. R. Gour, A. O. Gunina, E. Guidez, T. Harville, S. Irle, J. Ivanic, K. Kowalski, S. S. Leang, H. Li, W. Li, J. J. Lutz, I. Magoulas, J. Mato, V. Mironov, H. Nakata, B. Q. Pham, P. Piecuch, D. Poole, S. R. Pruitt, A. P. Rendell, L. B. Roskop, K. Ruedenberg, T. Sattasathuchana, M. W. Schmidt, J. Shen, L. Slipchenko, M. Sosonkina, V. Sundriyal, A. Tiwari, J. L. Galvez Vallejo, B. Westheimer, M. Włoch, P. Xu, F. Zahariev, M. S. Gordon, *J. Chem. Phys.* **2020**, *152*, 154102.

NMR spectral data

^1H NMR (1a)

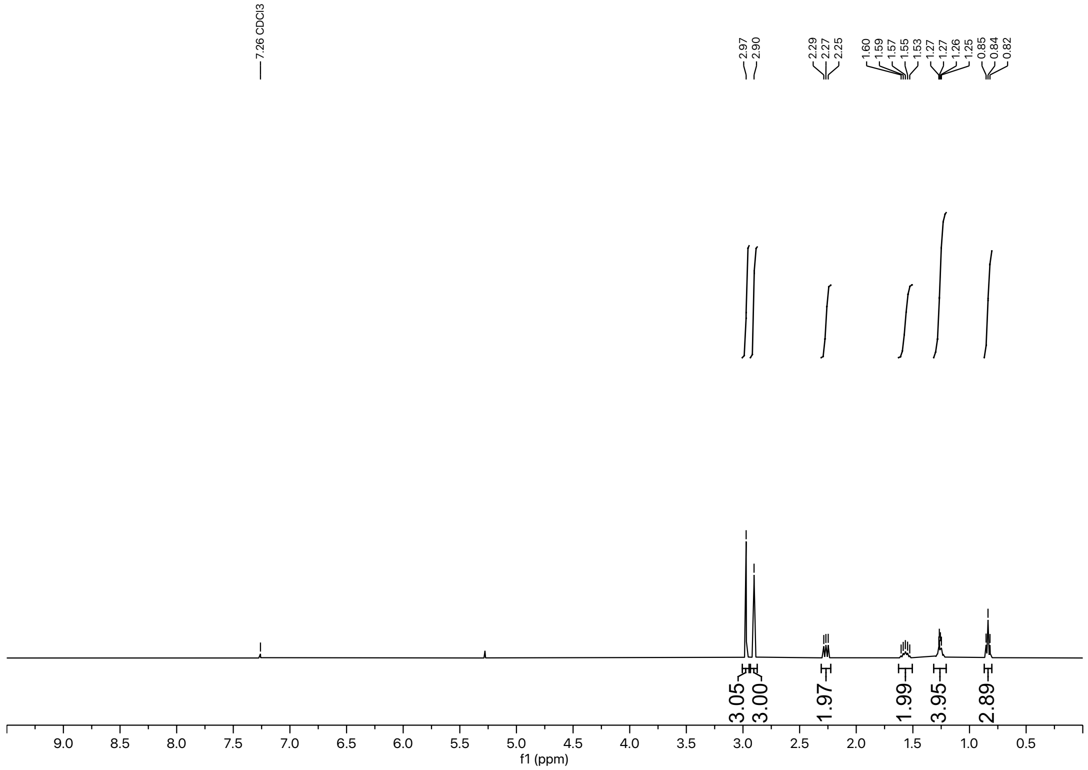

^{13}C NMR (1a)

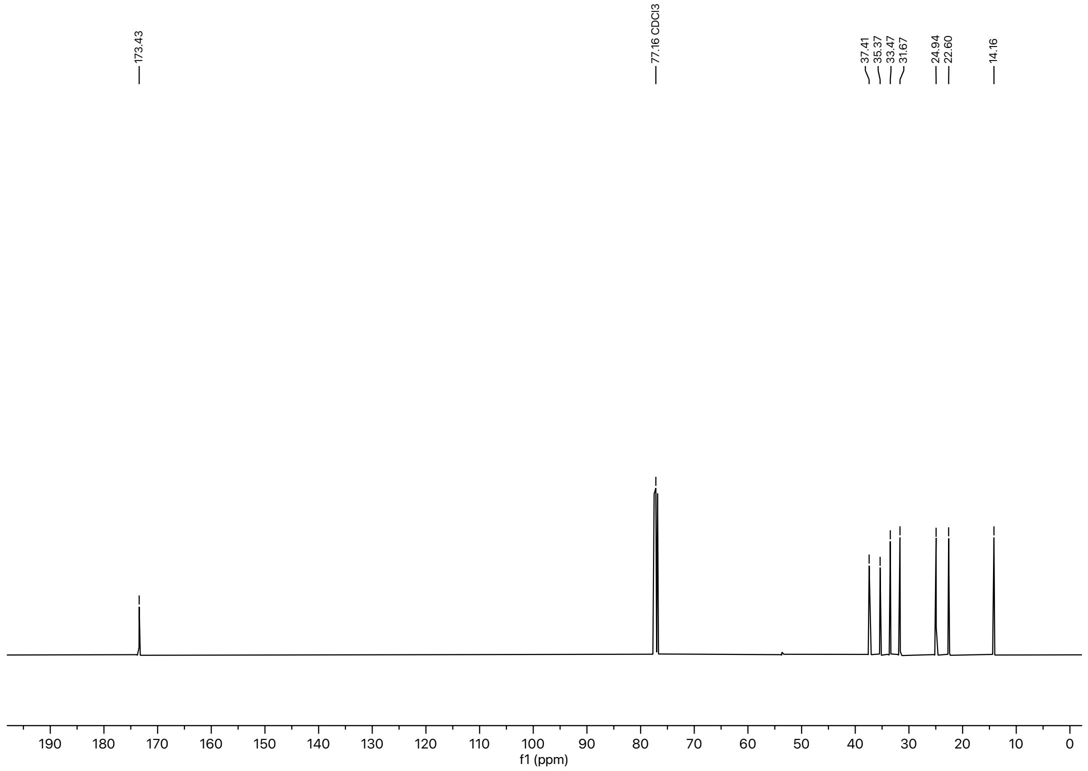

^{13}C DEPT-135 (1a)

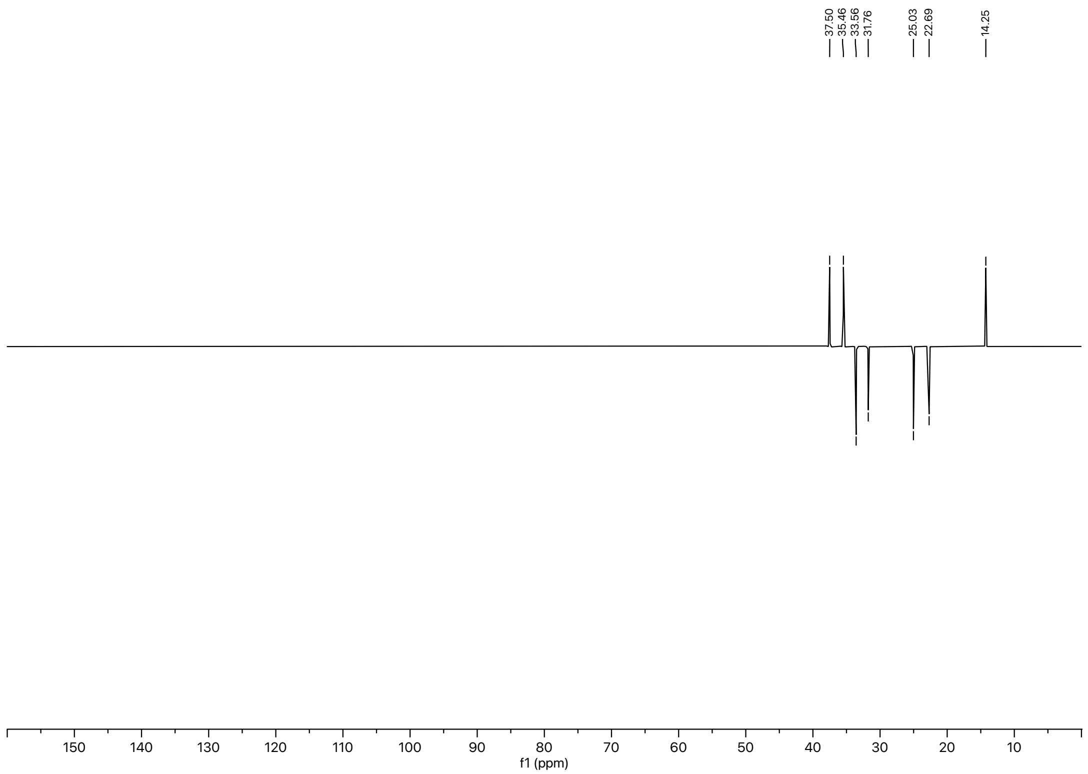

^{1}H NMR (1c)

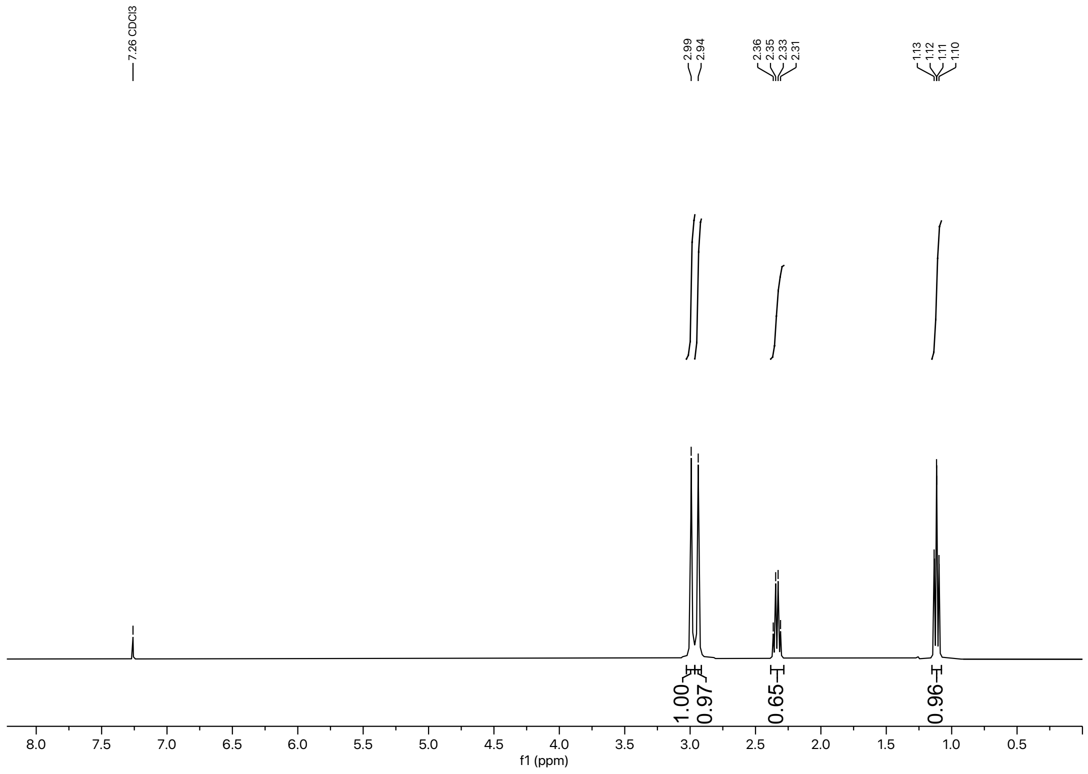

^{13}C NMR (1c)

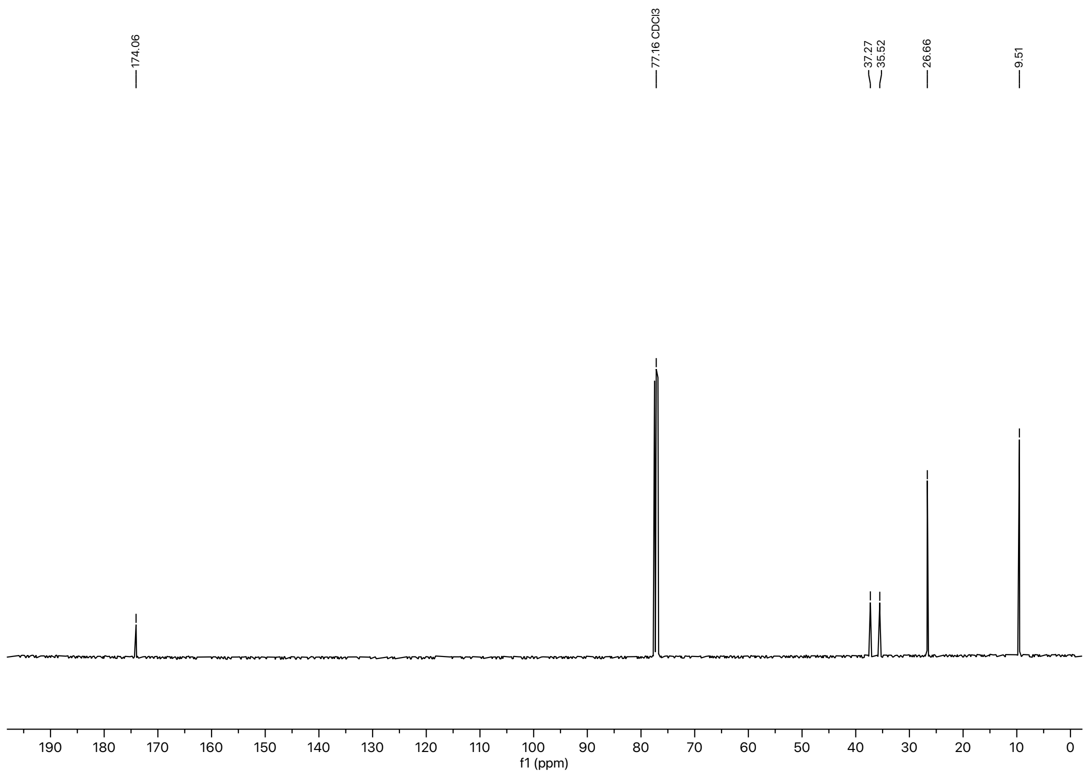

^{13}C DEPT-135 (1c)

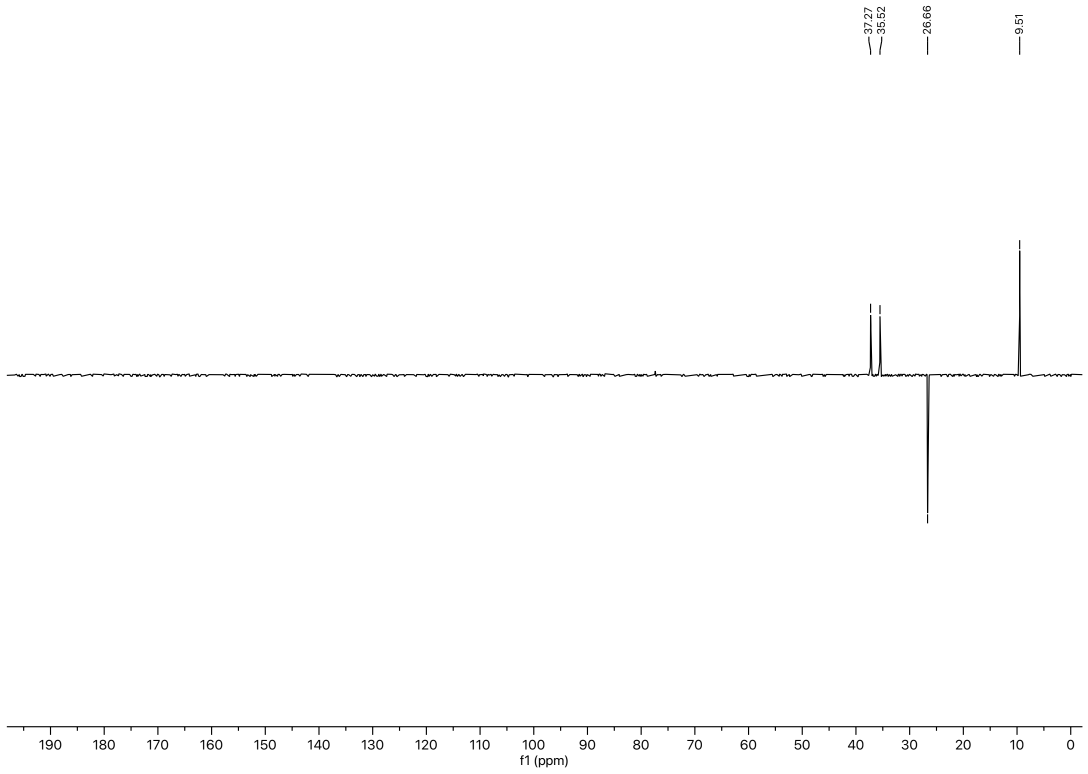

^{1}H NMR (1d)

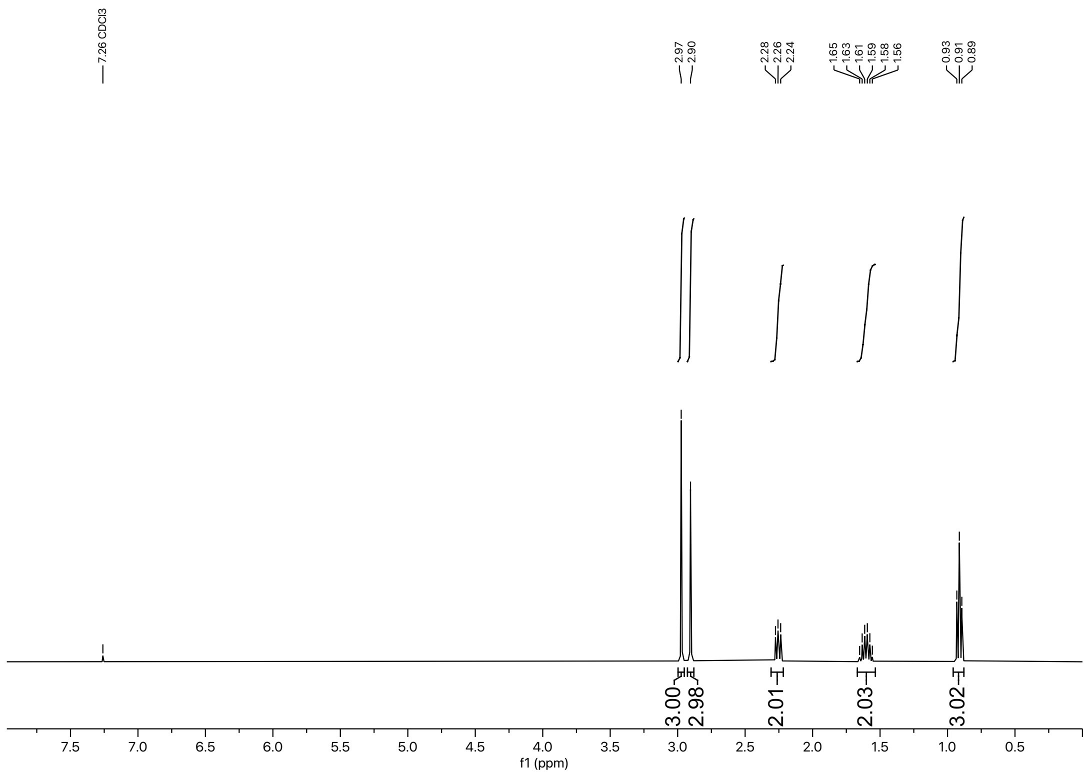

^{13}C NMR (1d)

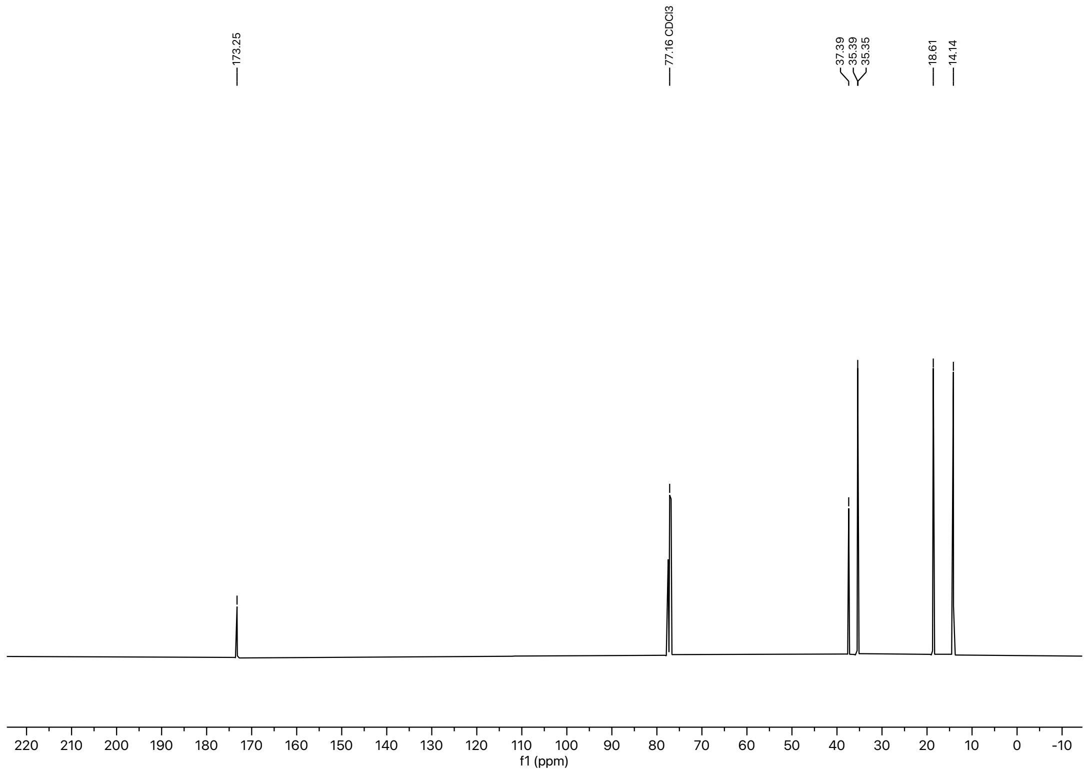

^{13}C DEPT-135 (1d)

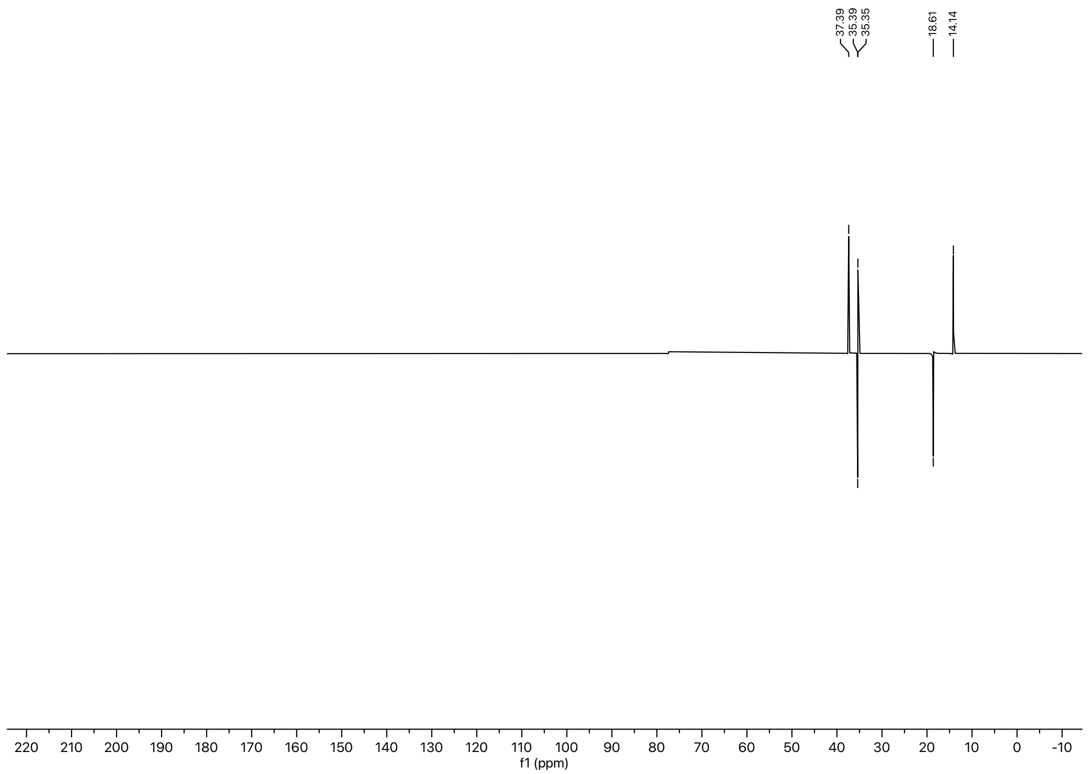

^{1}H NMR (1e)

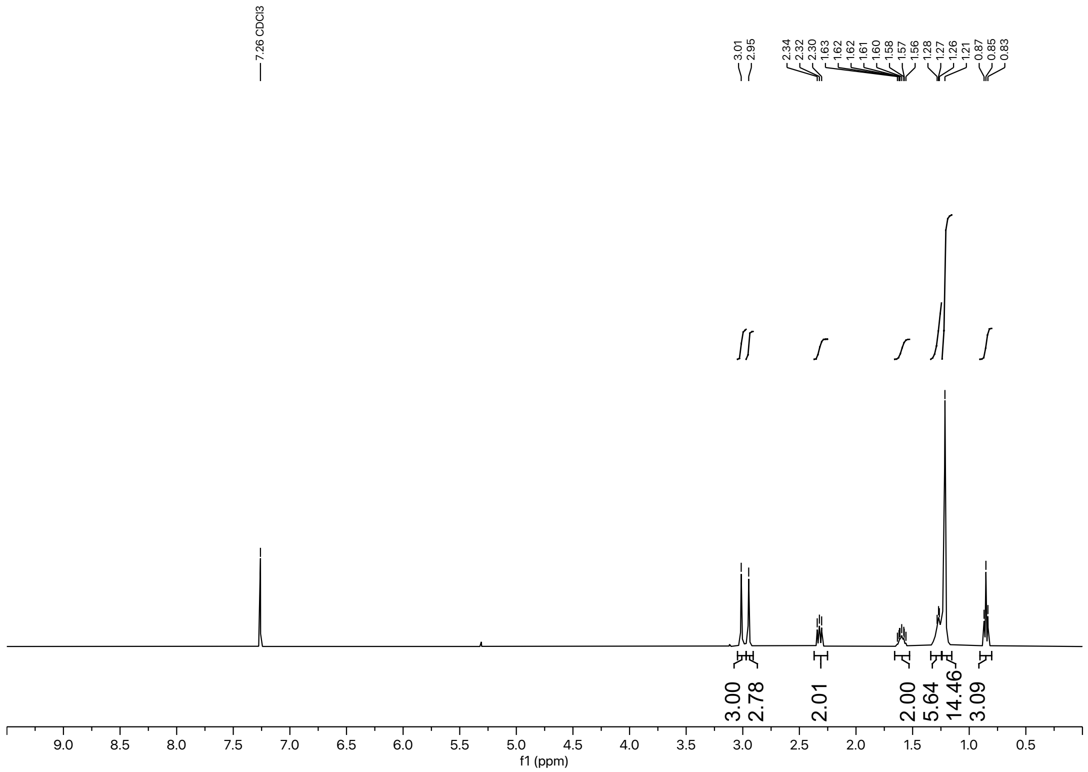

^{13}C NMR (1e)

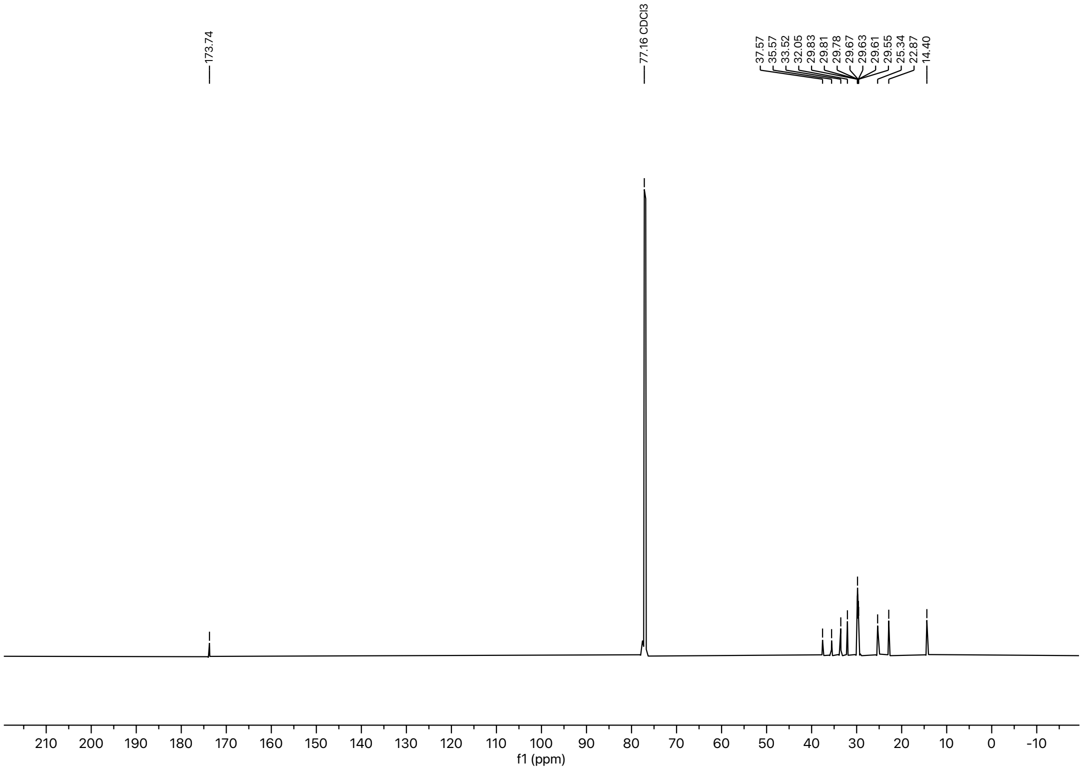

^{13}C DEPT-135 (1e)

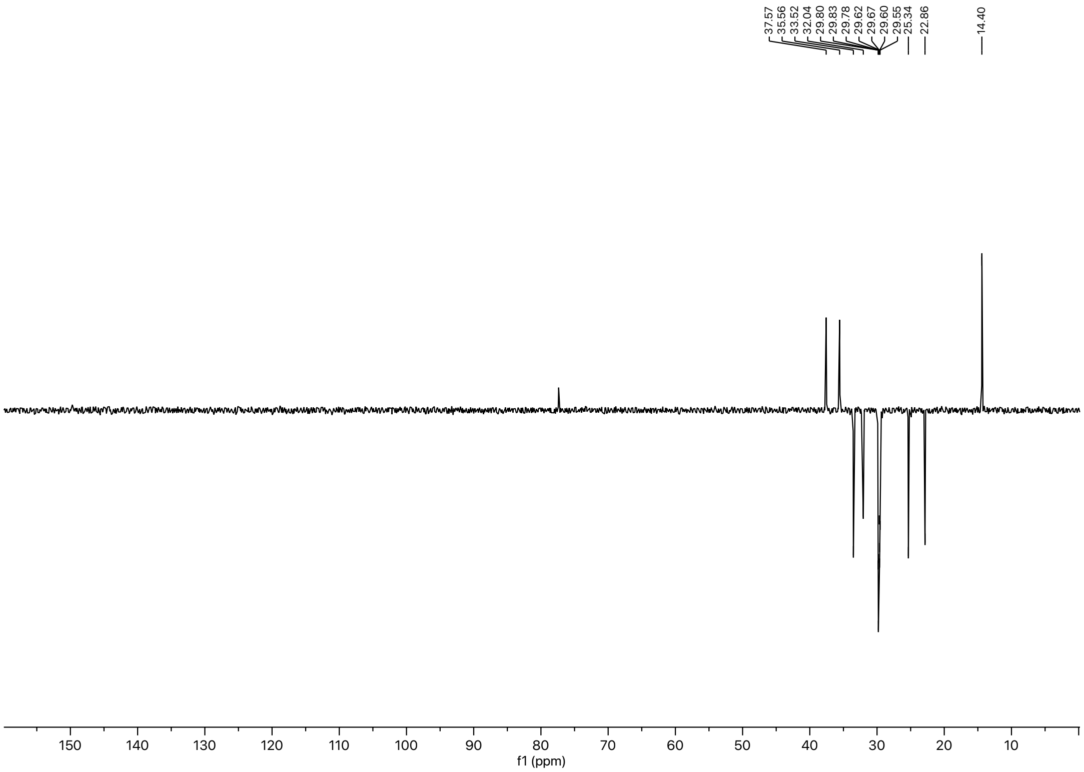

^{1}H NMR (1g)

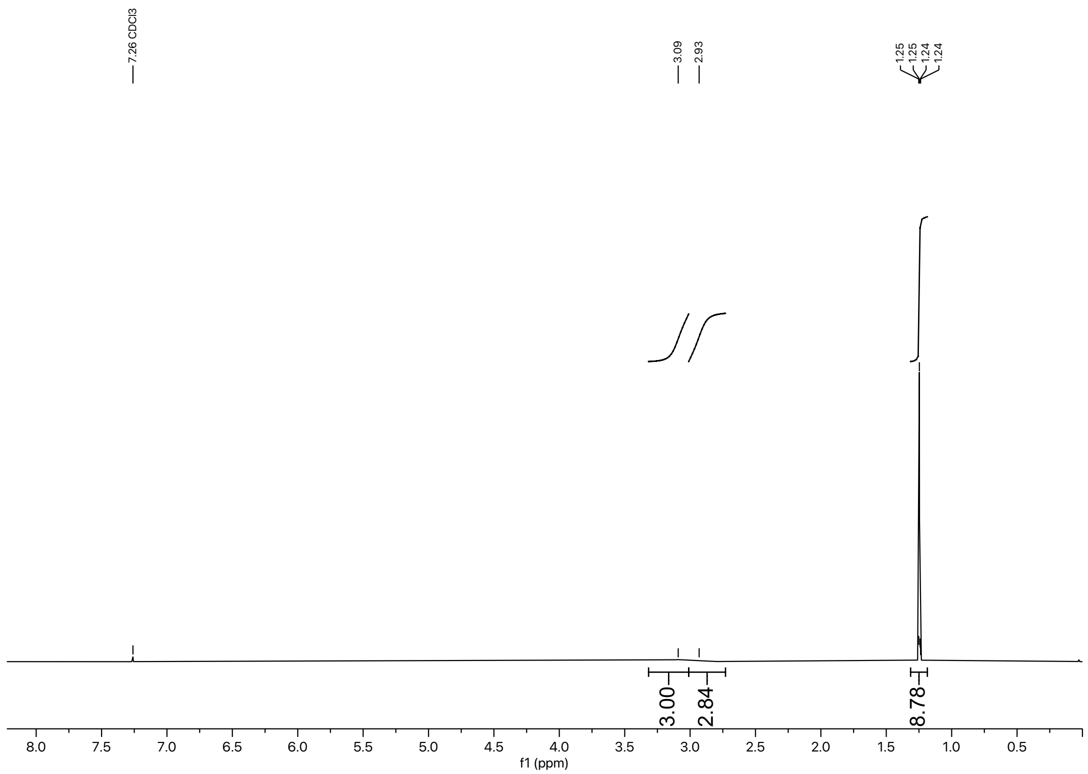

^{13}C NMR (1g)

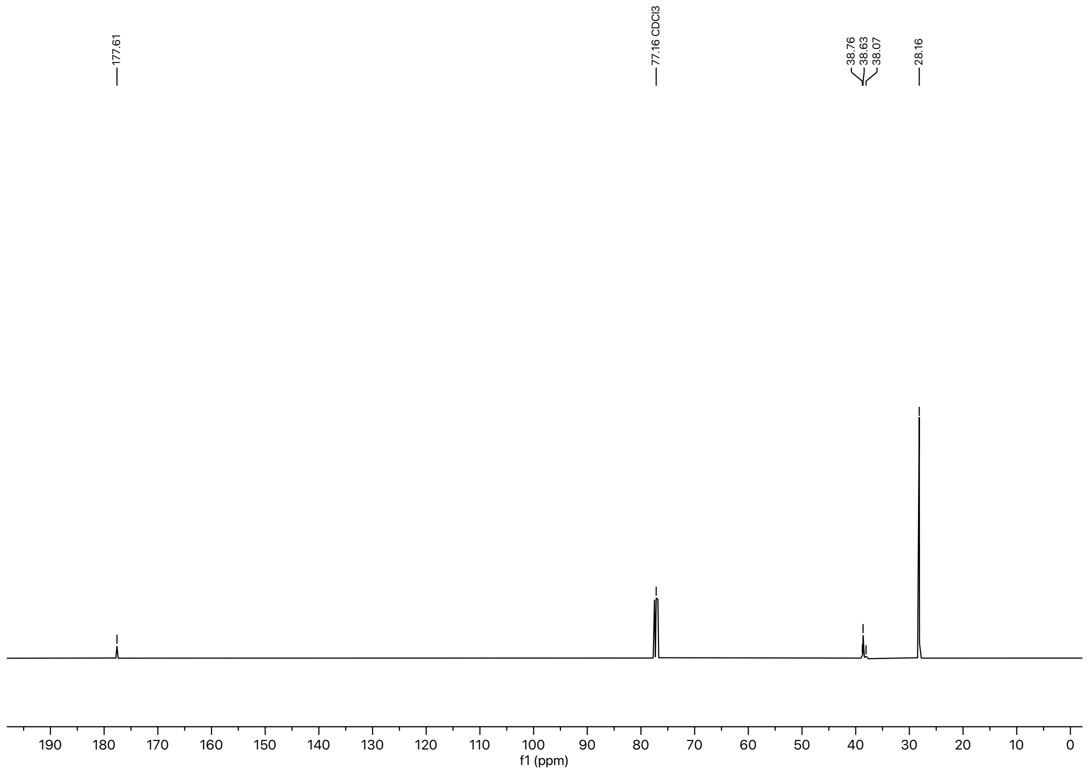

^{13}C DEPT-135 (1g)

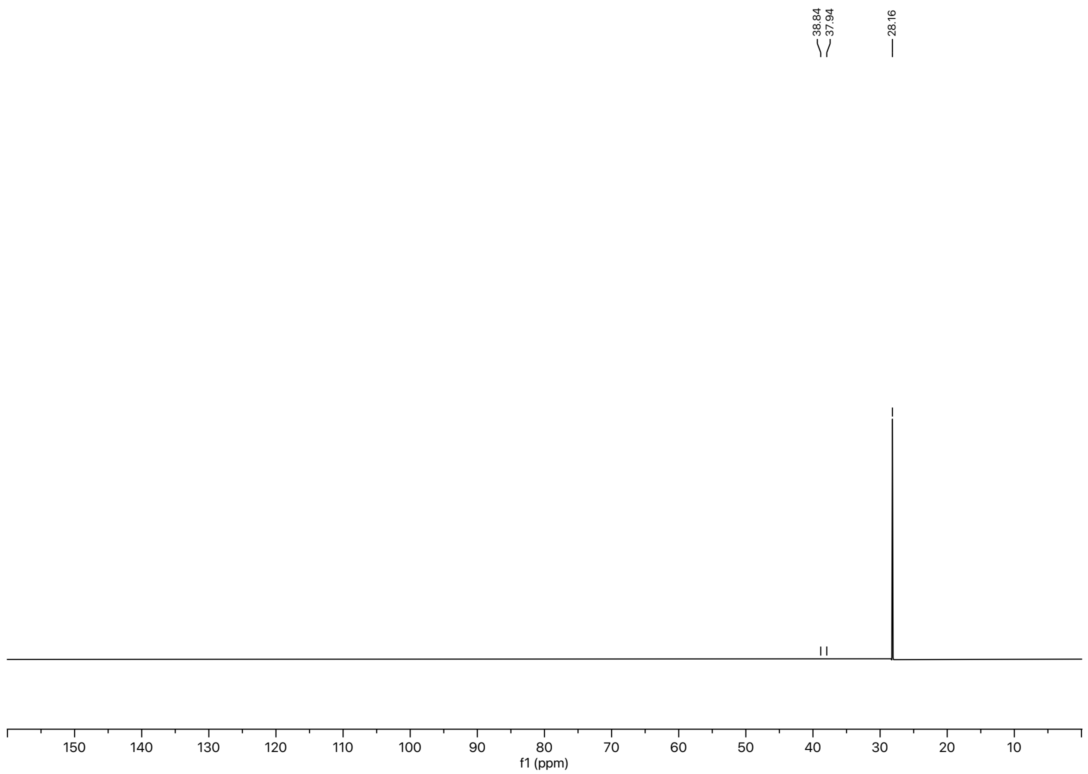

^{1}H NMR (1h)

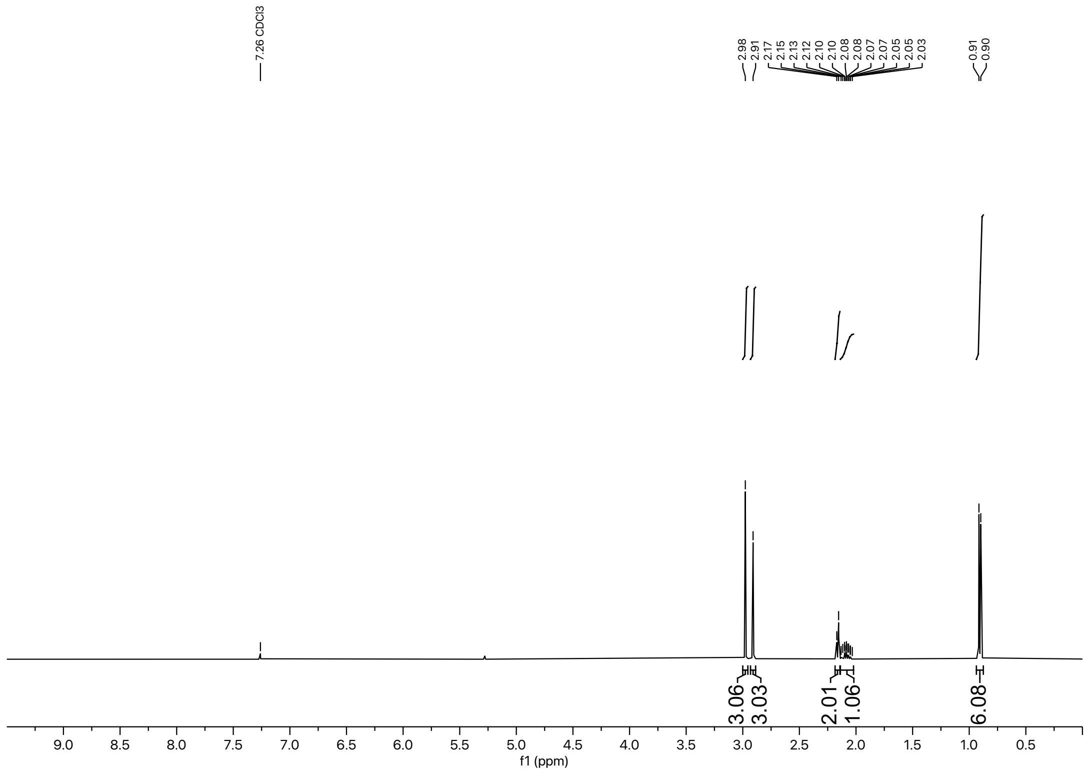

^{13}C NMR (1h)

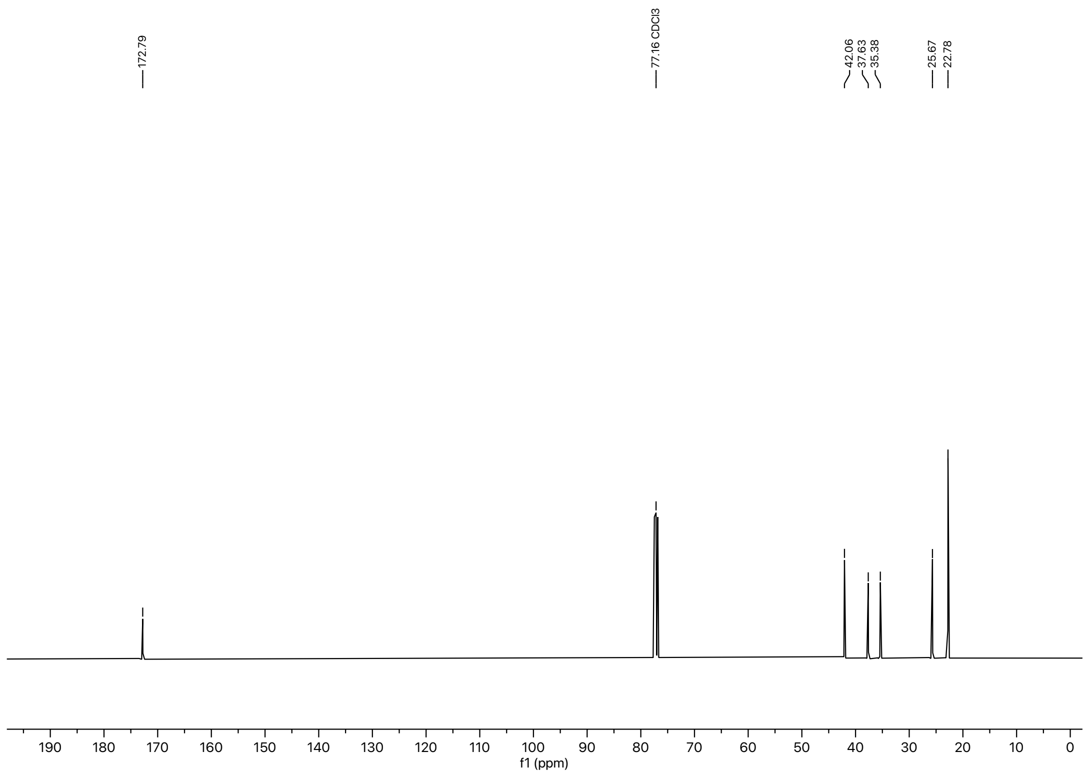

^{13}C DEPT-135 (1h)

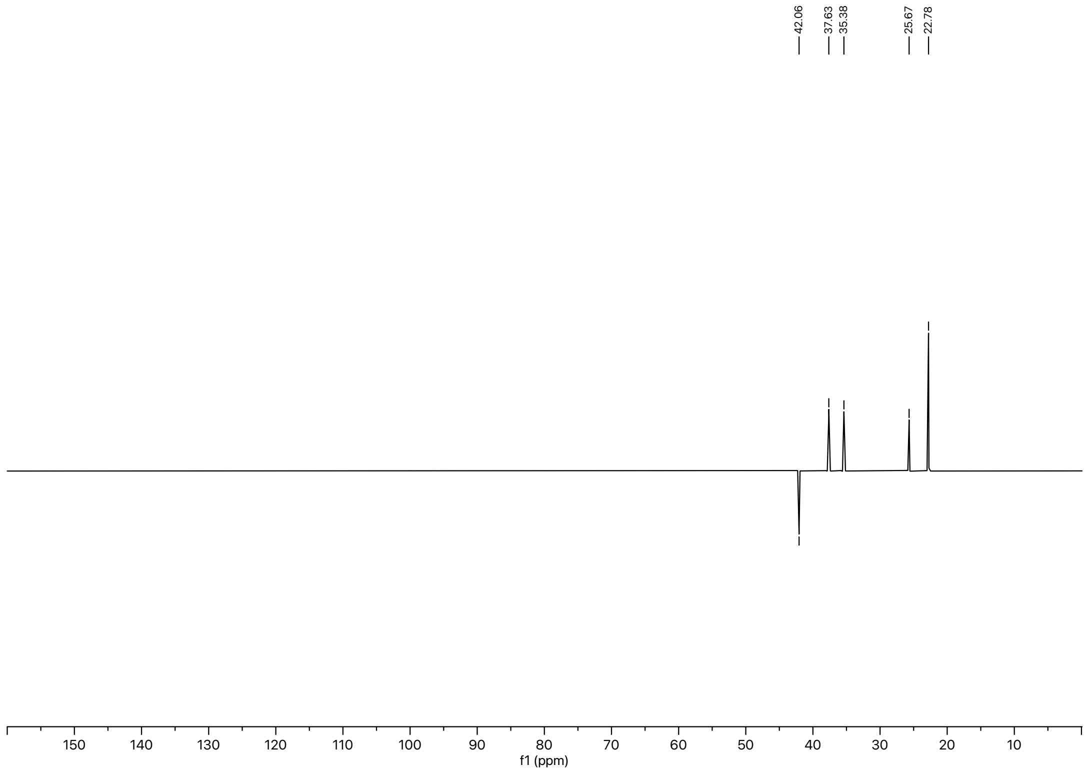

^{1}H NMR (1i)

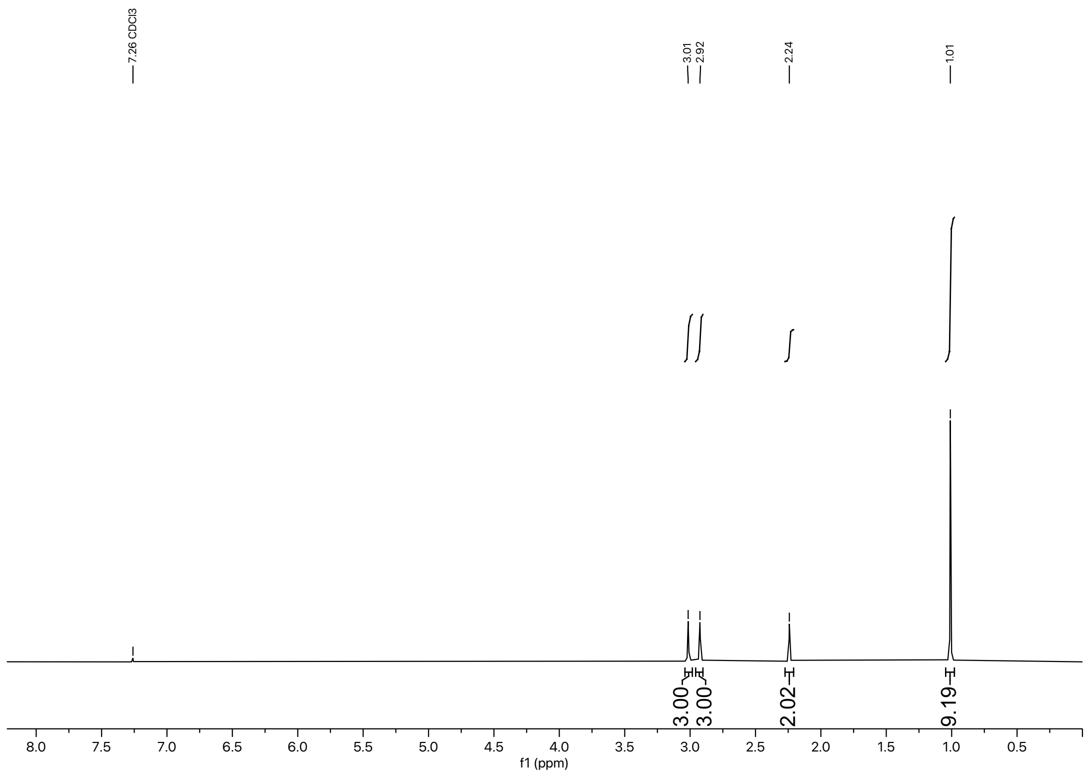

^{13}C NMR (1i)

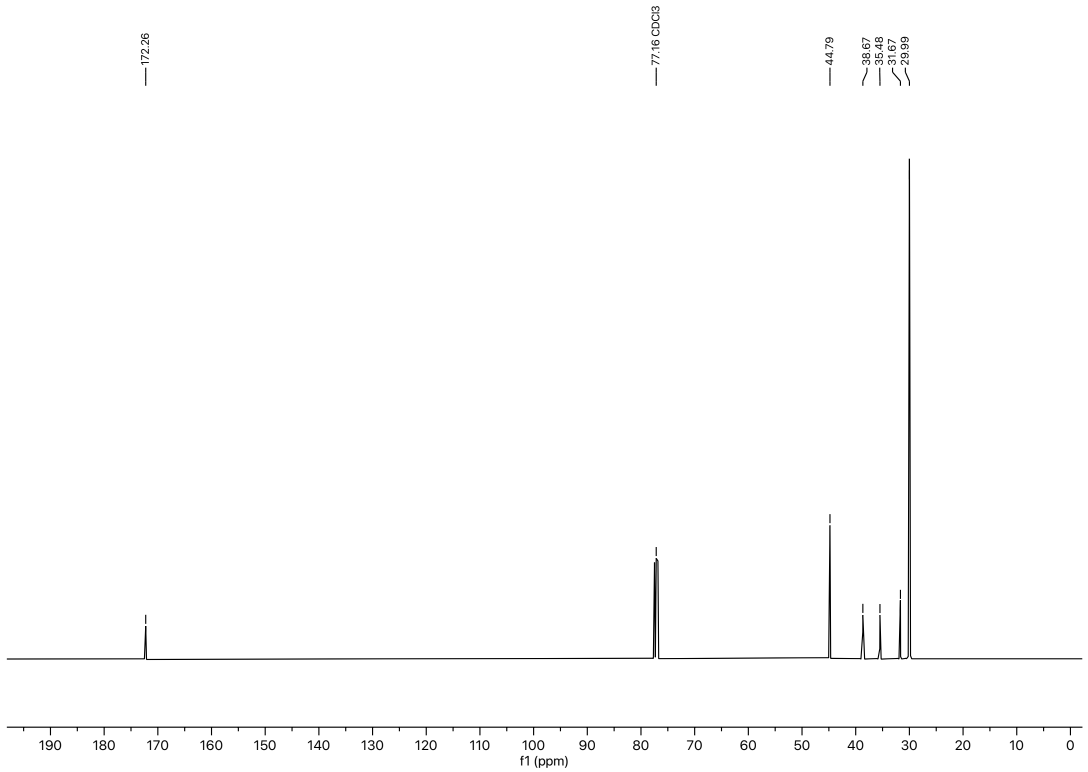

^{13}C DEPT-135 (1i)

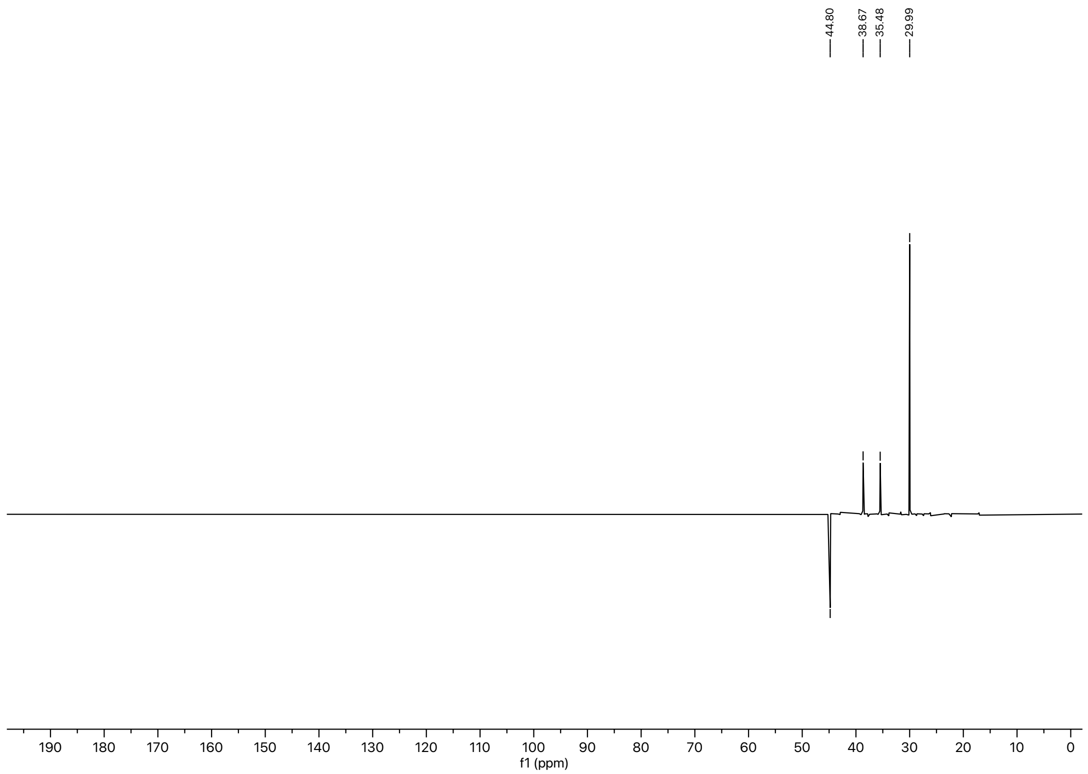

^{1}H NMR (1j)

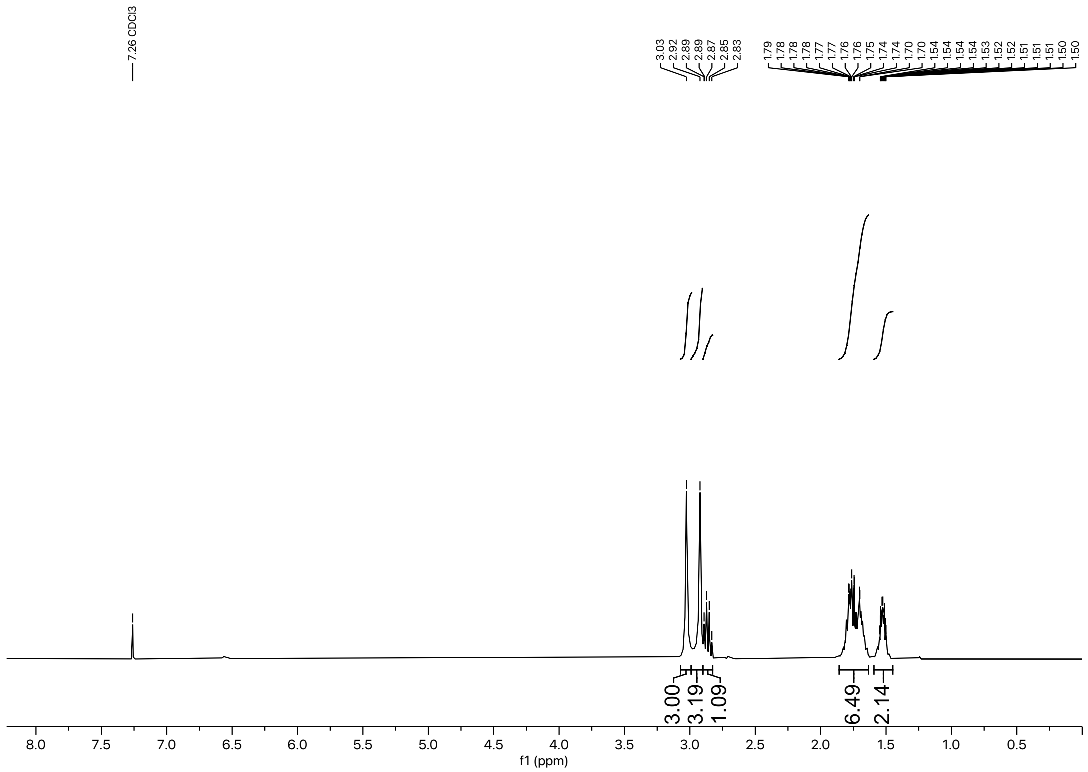

^{13}C NMR (1j)

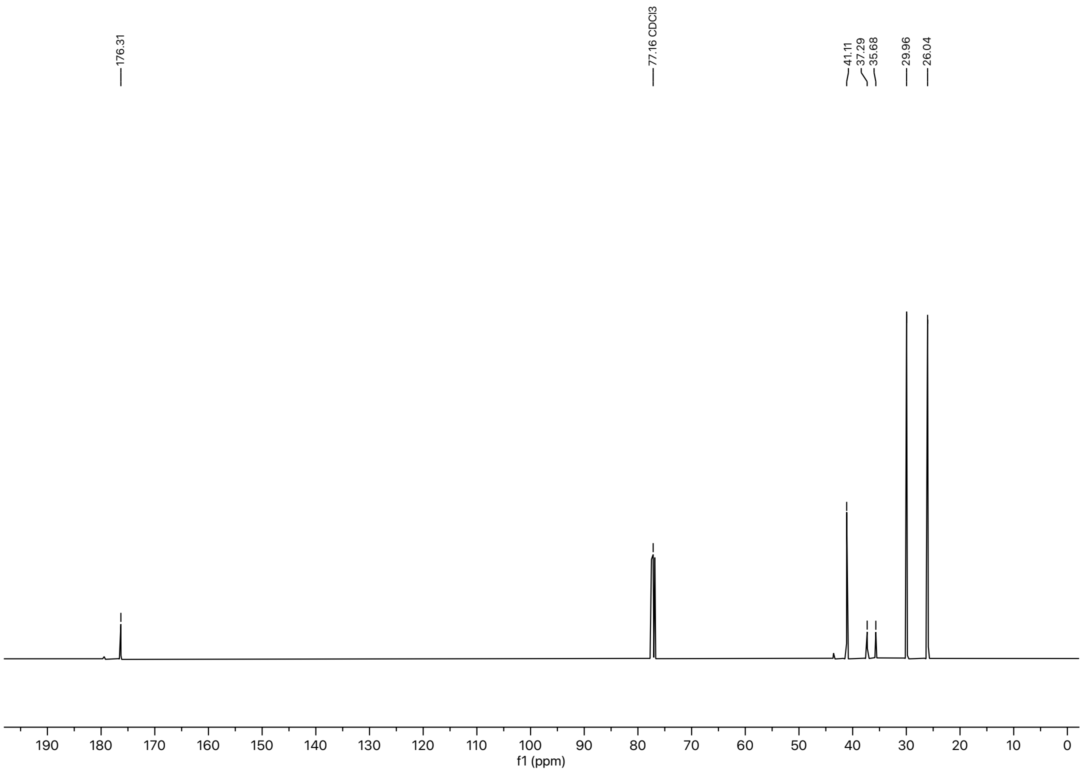

^{13}C DEPT-135 (1j)

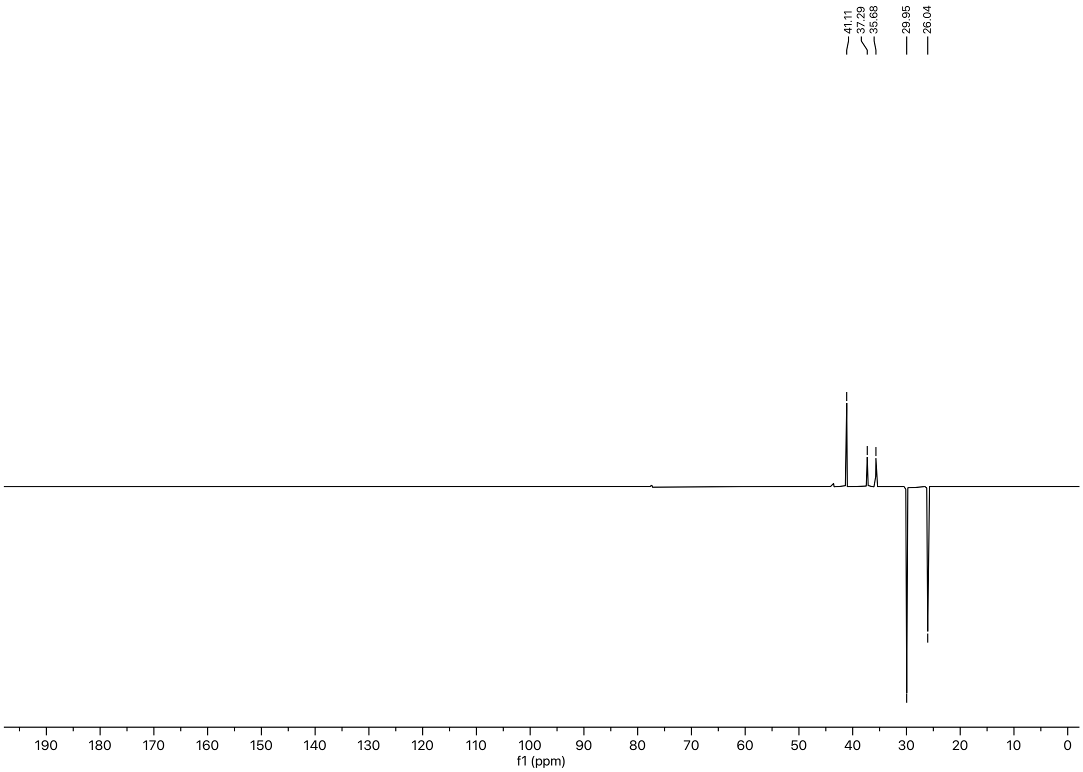

^{1}H NMR (1k)

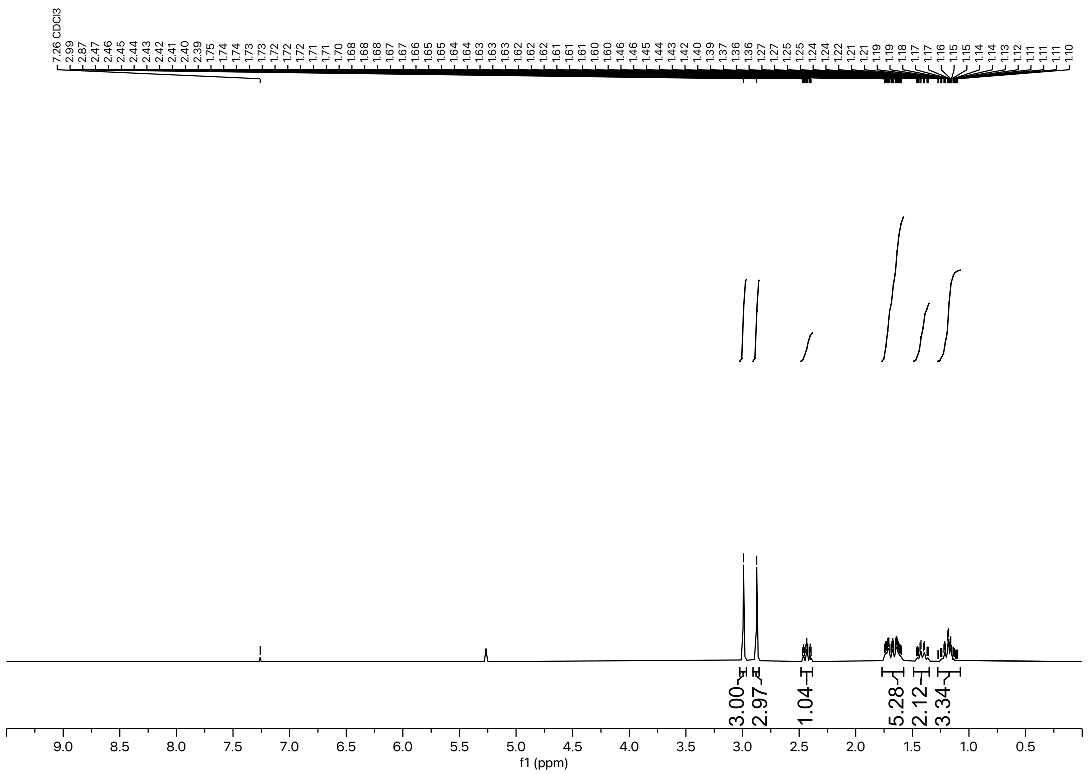

^{13}C NMR (1k)

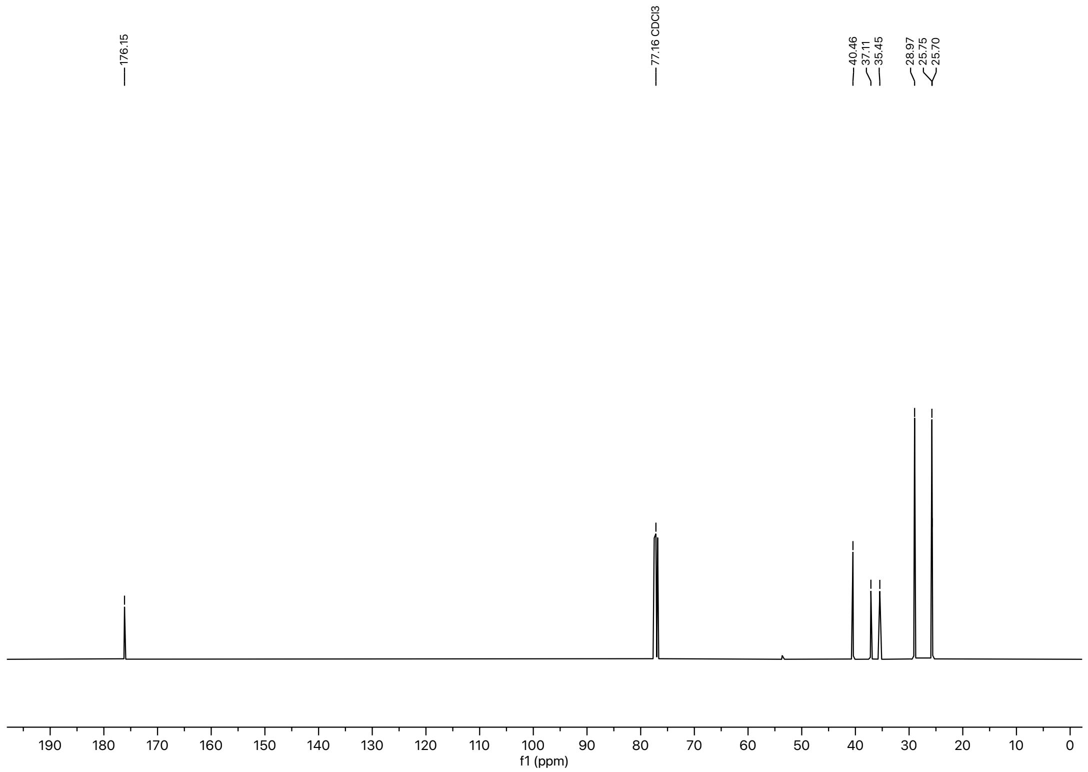

^{13}C DEPT-135 (1k)

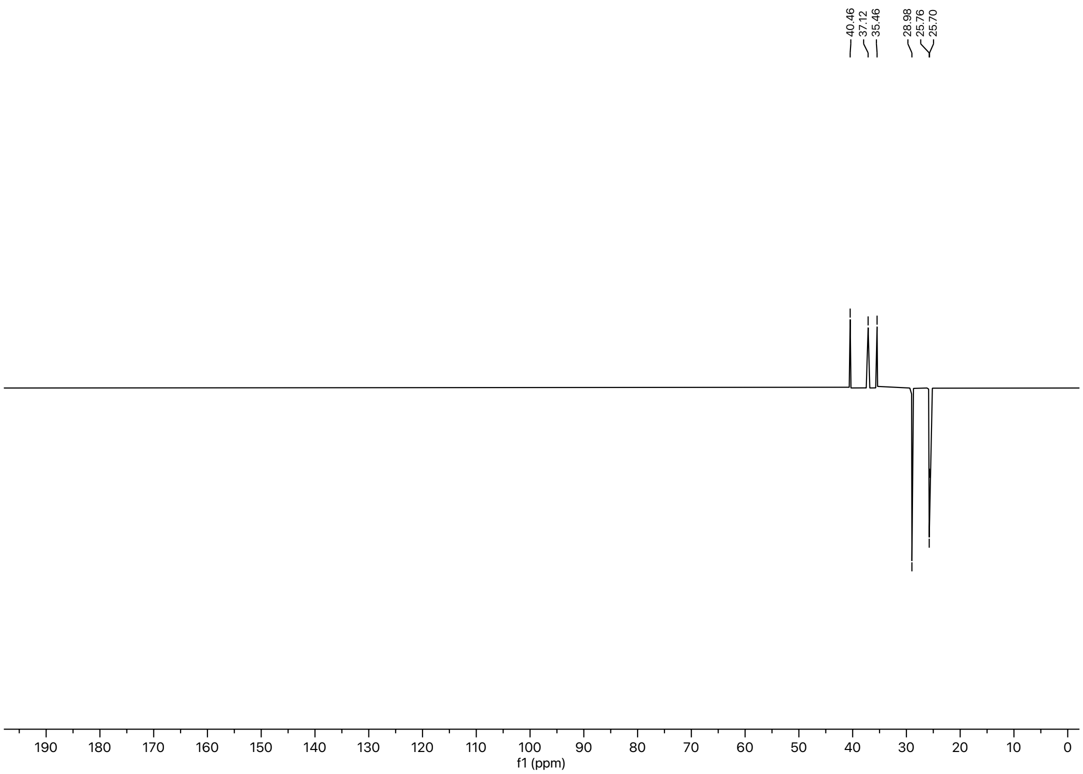

^{1}H NMR (1l)

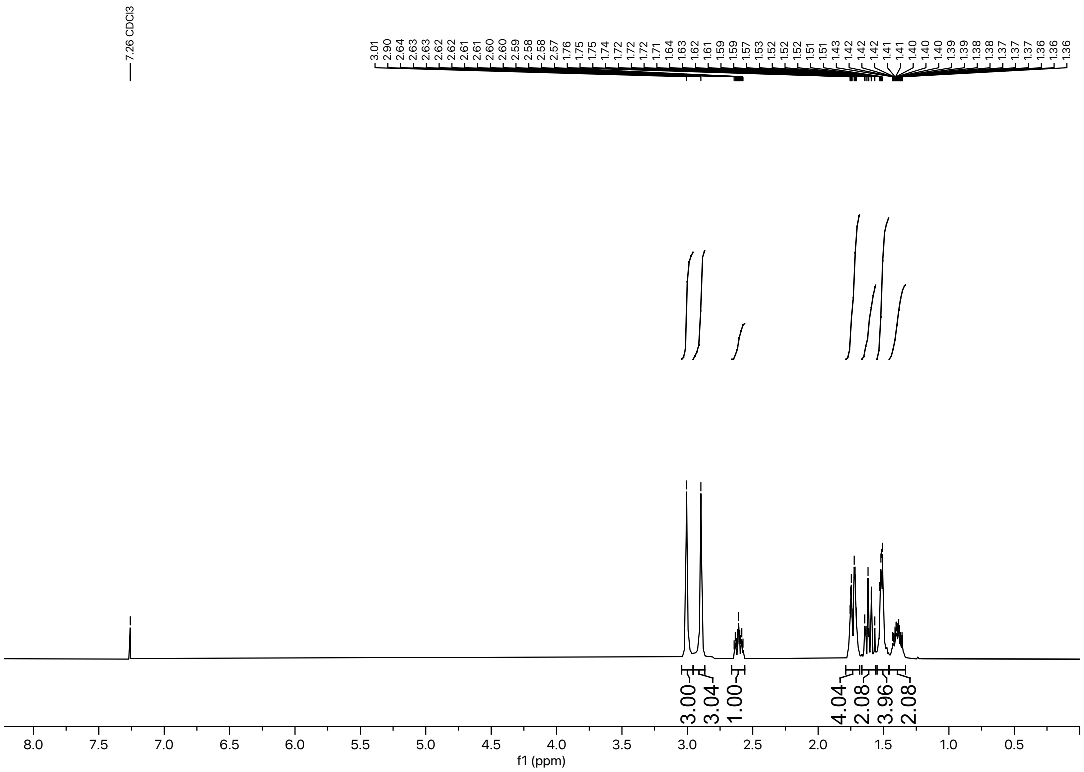

^{13}C NMR (1l)

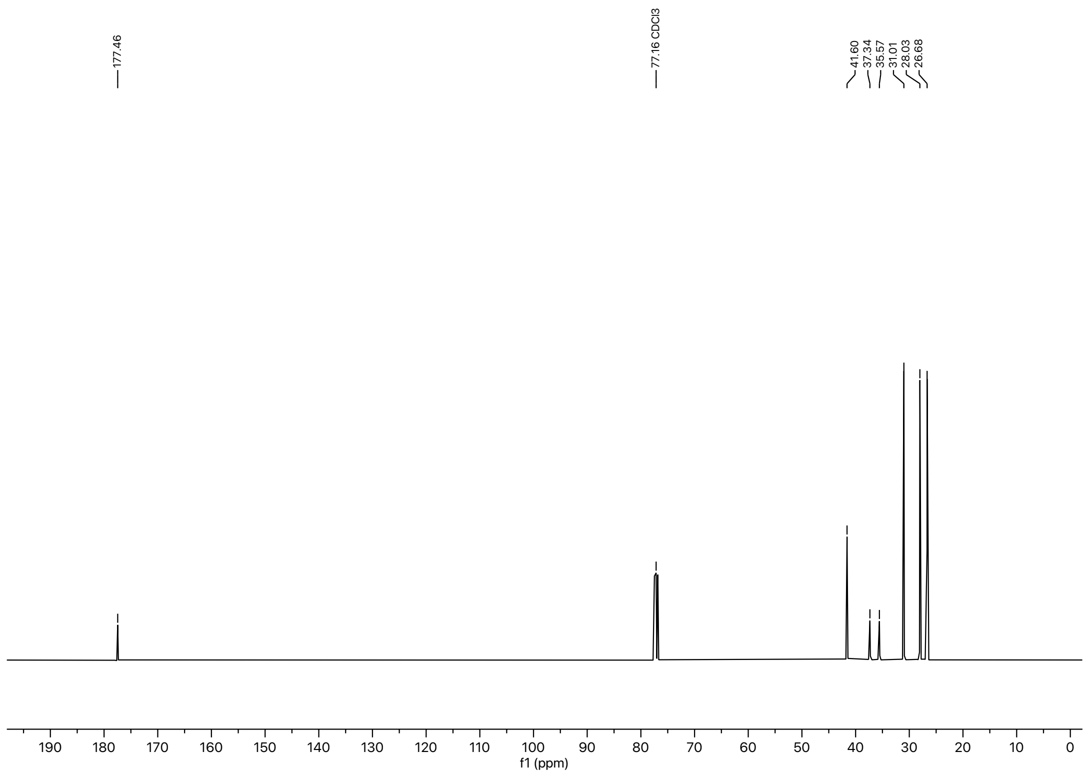

^{13}C DEPT-135 (1l)

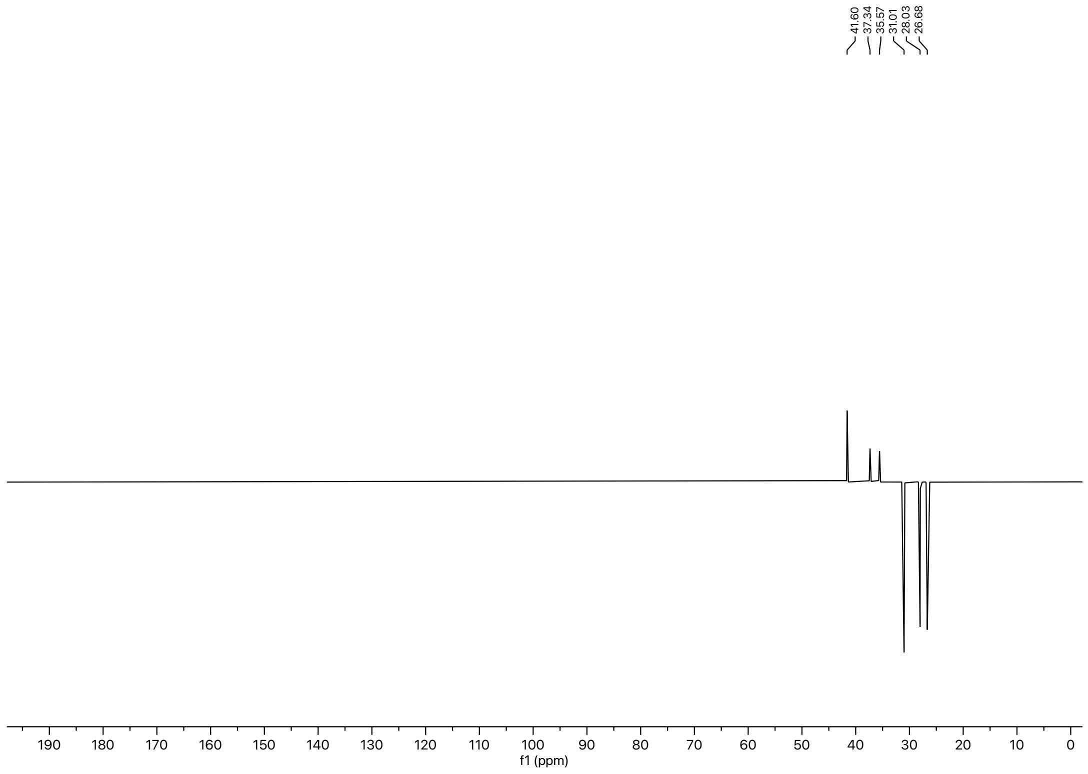

^1H NMR (1m)

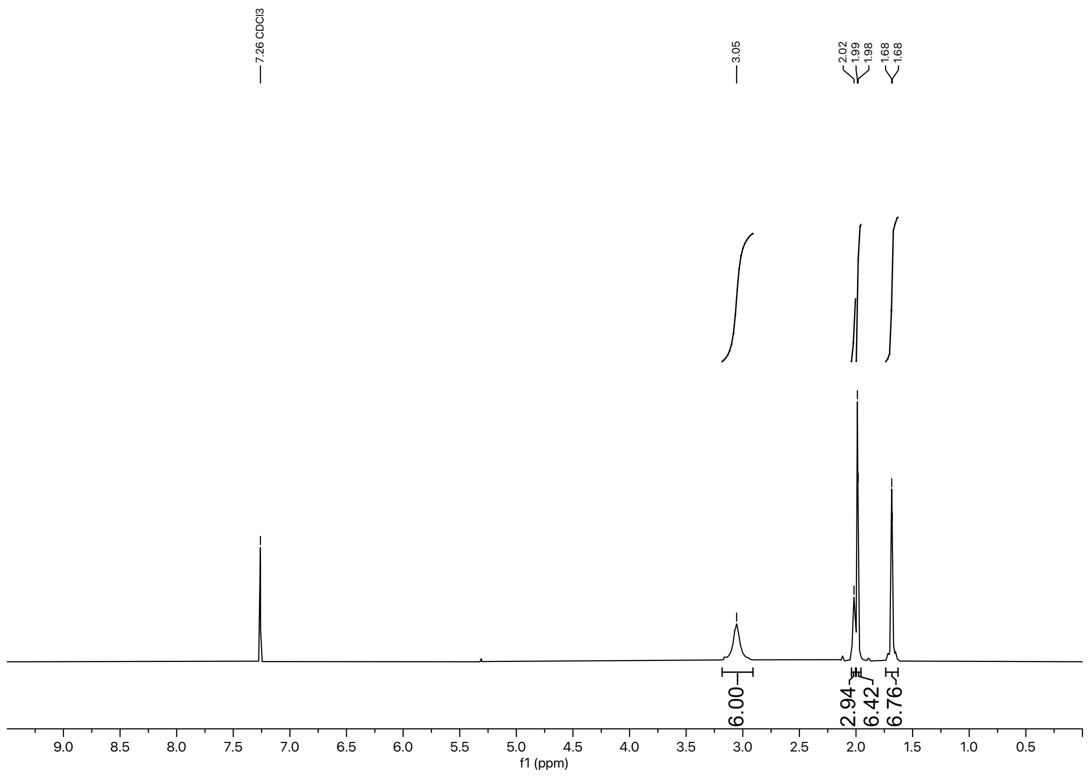

^{13}C NMR (1m)

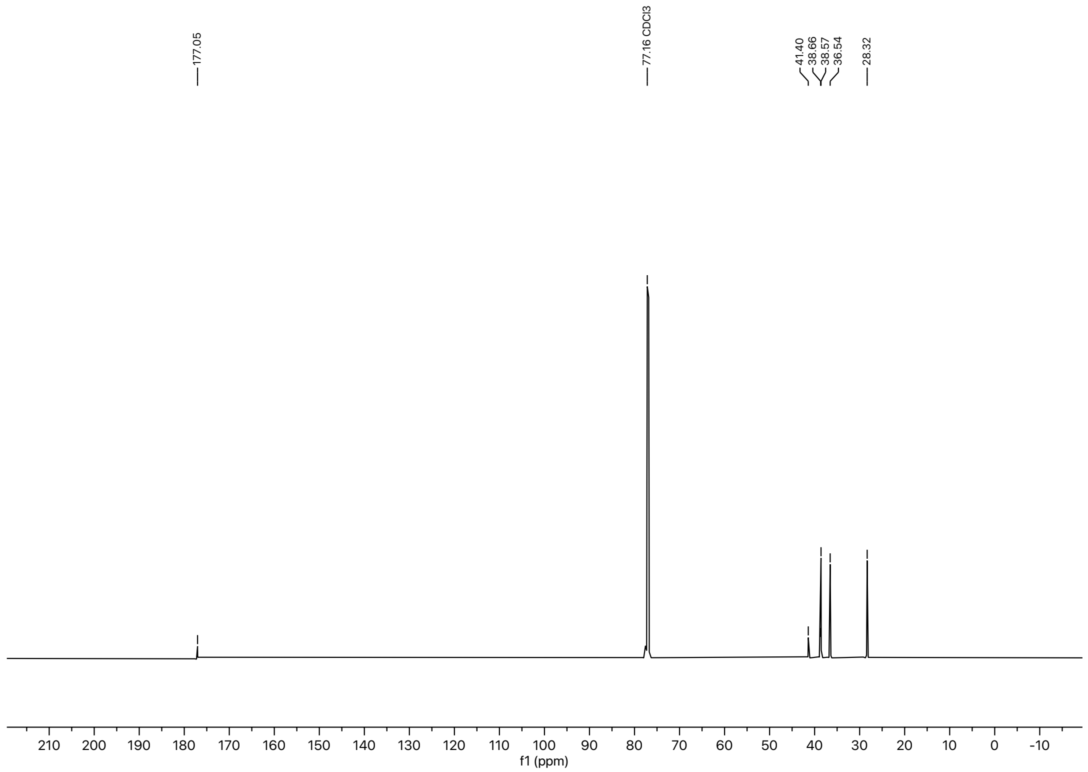

^{13}C DEPT-135 (1m)

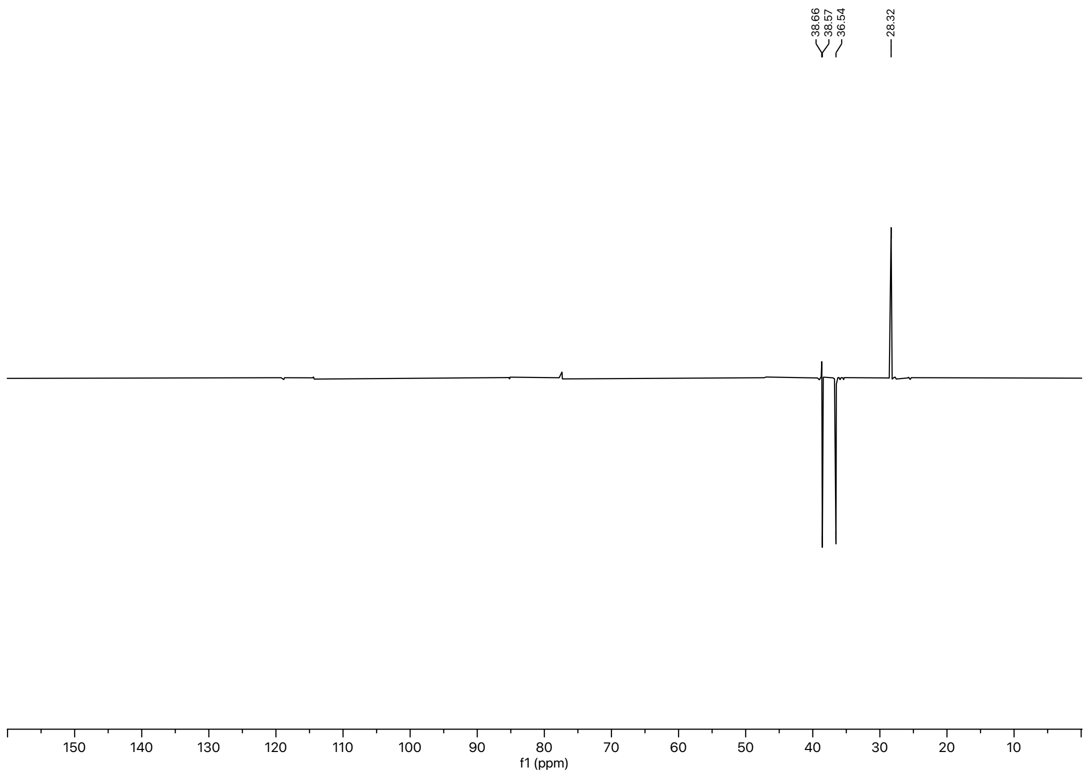

^{1}H NMR (1n)

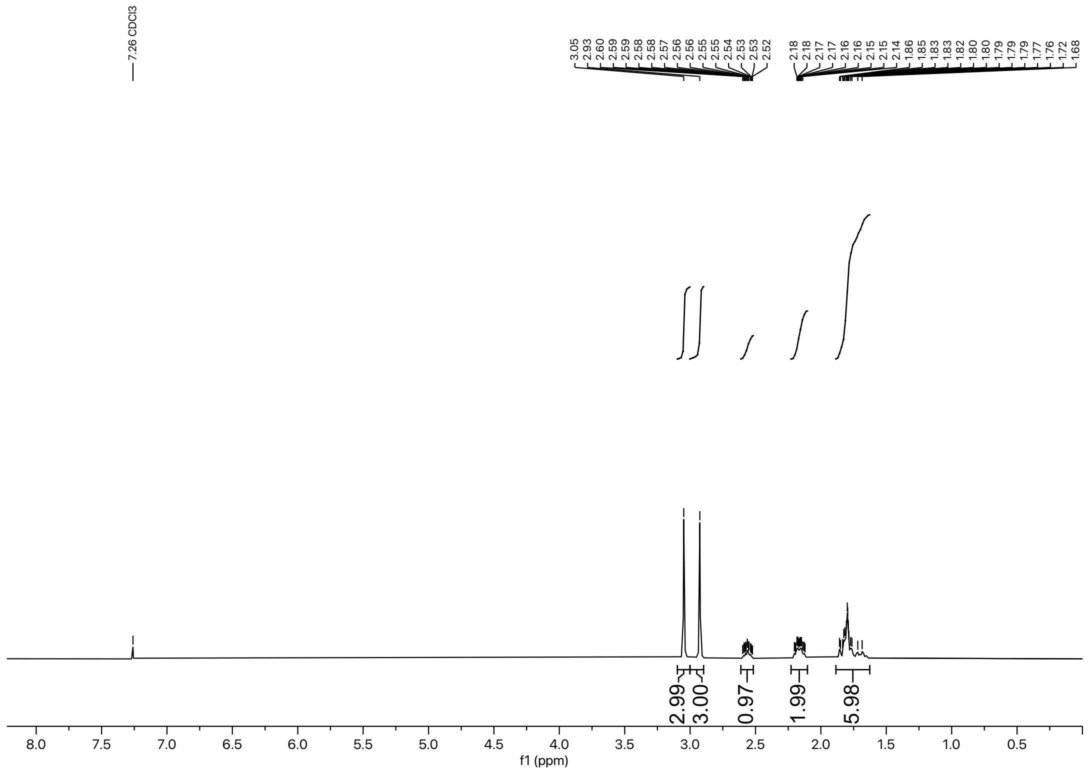

^{13}C NMR (1n)

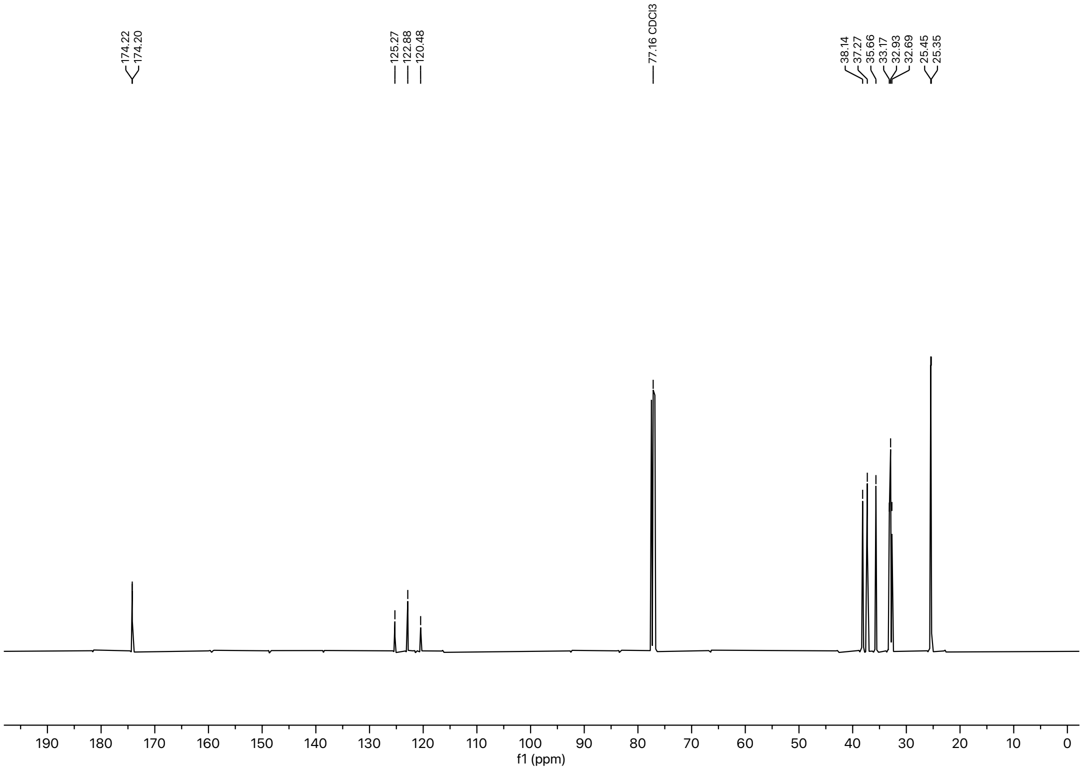

^{13}C DEPT-135 (1n)

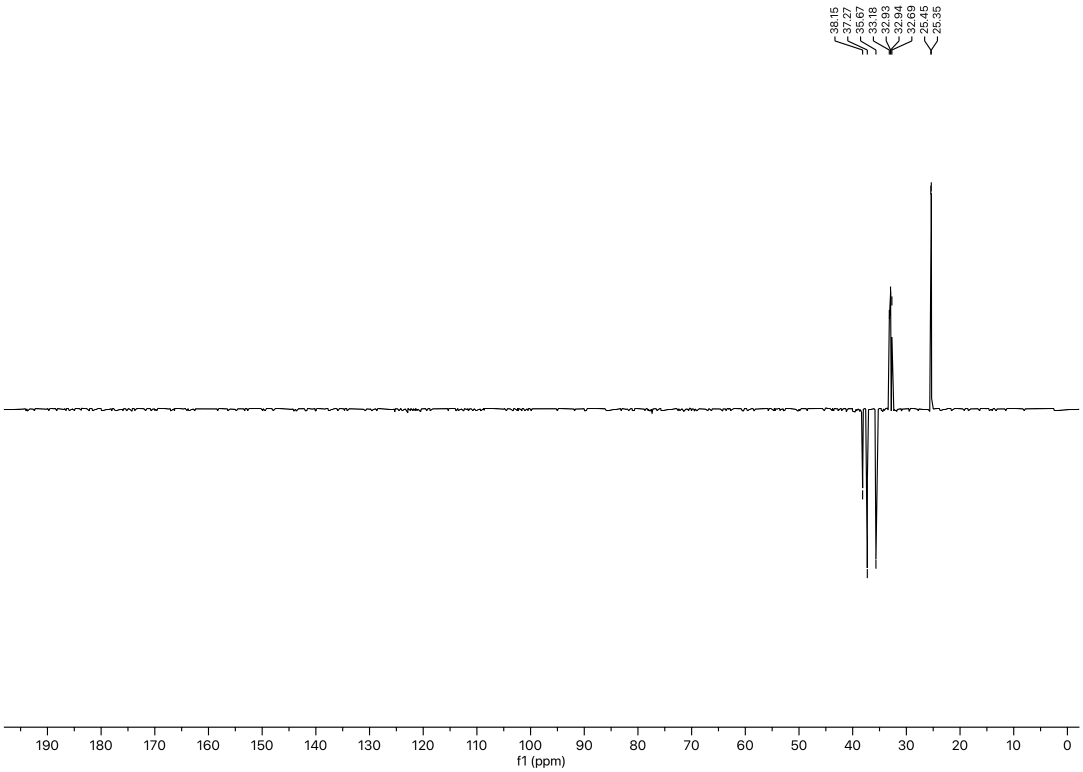

^{19}F NMR (1n)

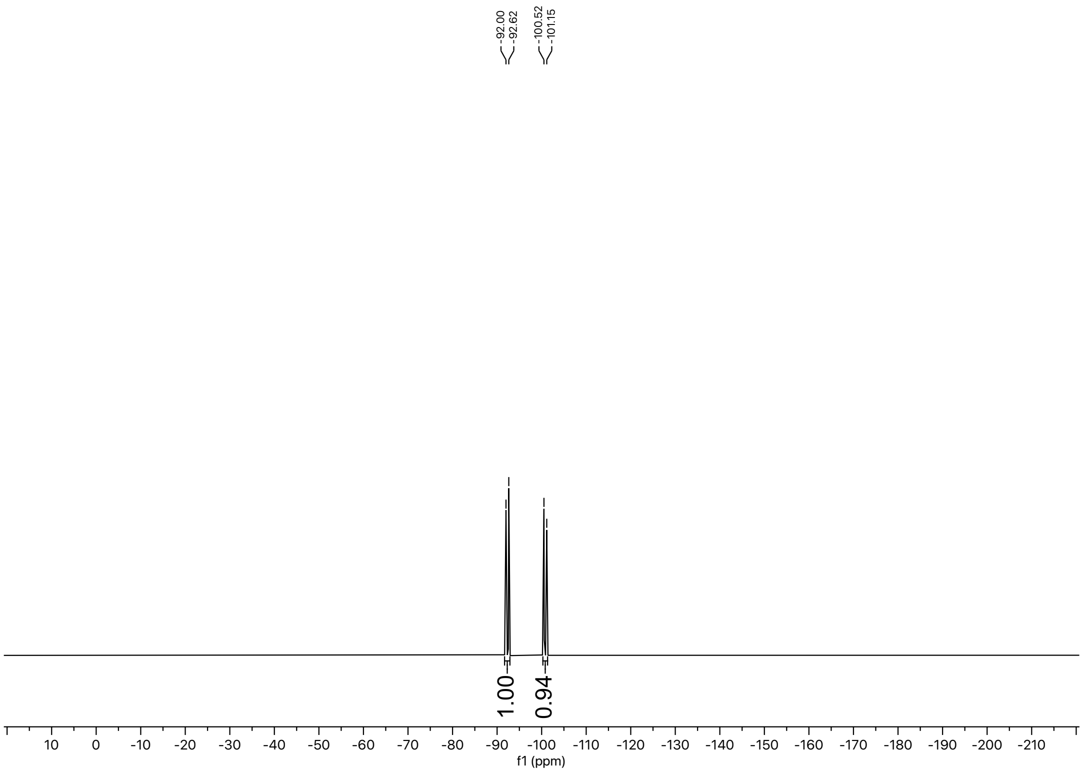

^{1}H NMR (1o)

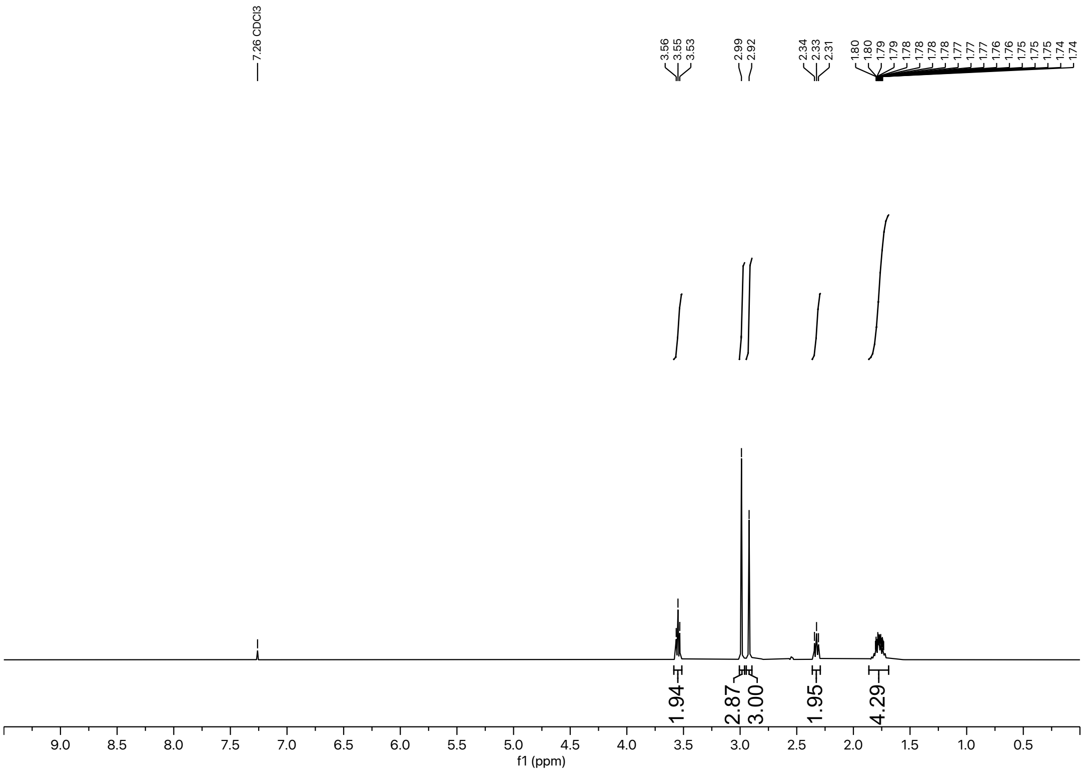

^{13}C NMR (1o)

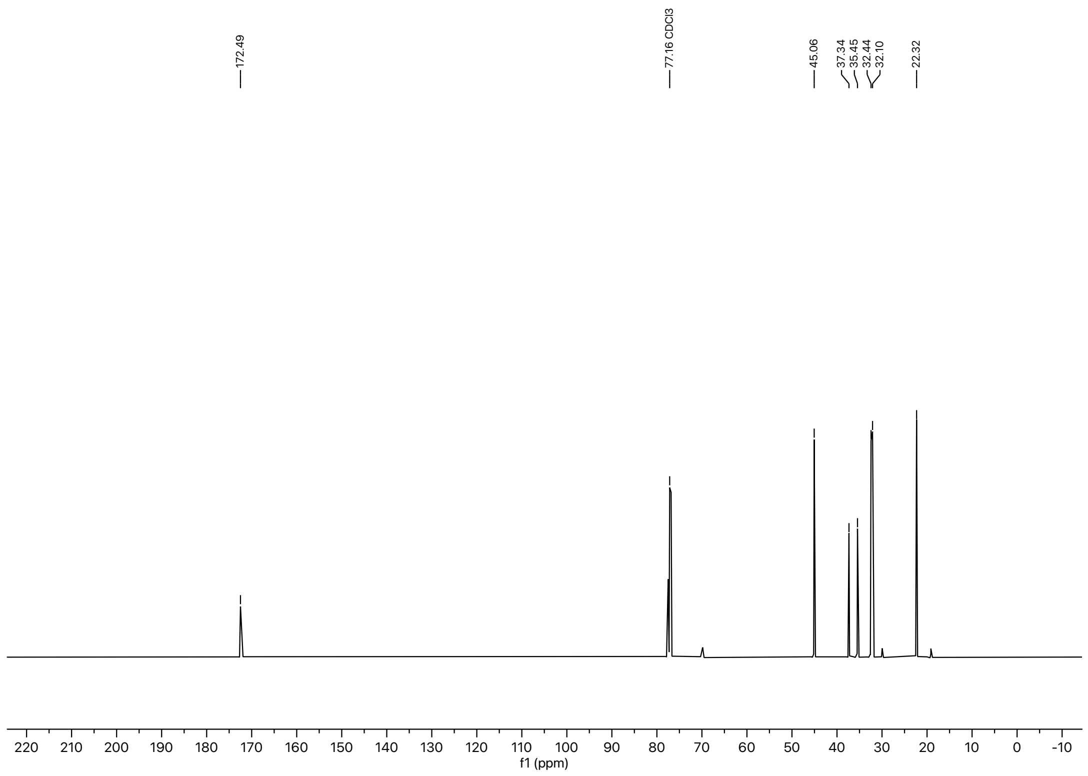

^{13}C DEPT-135 (1o)

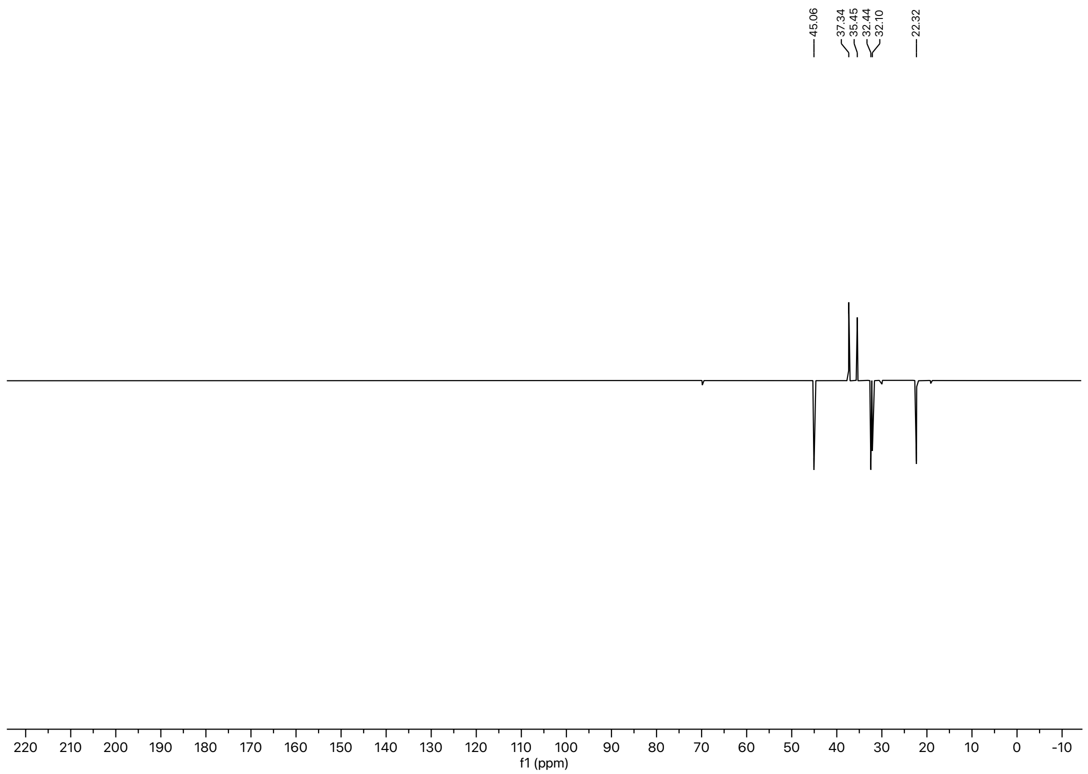

^1H NMR (1p)

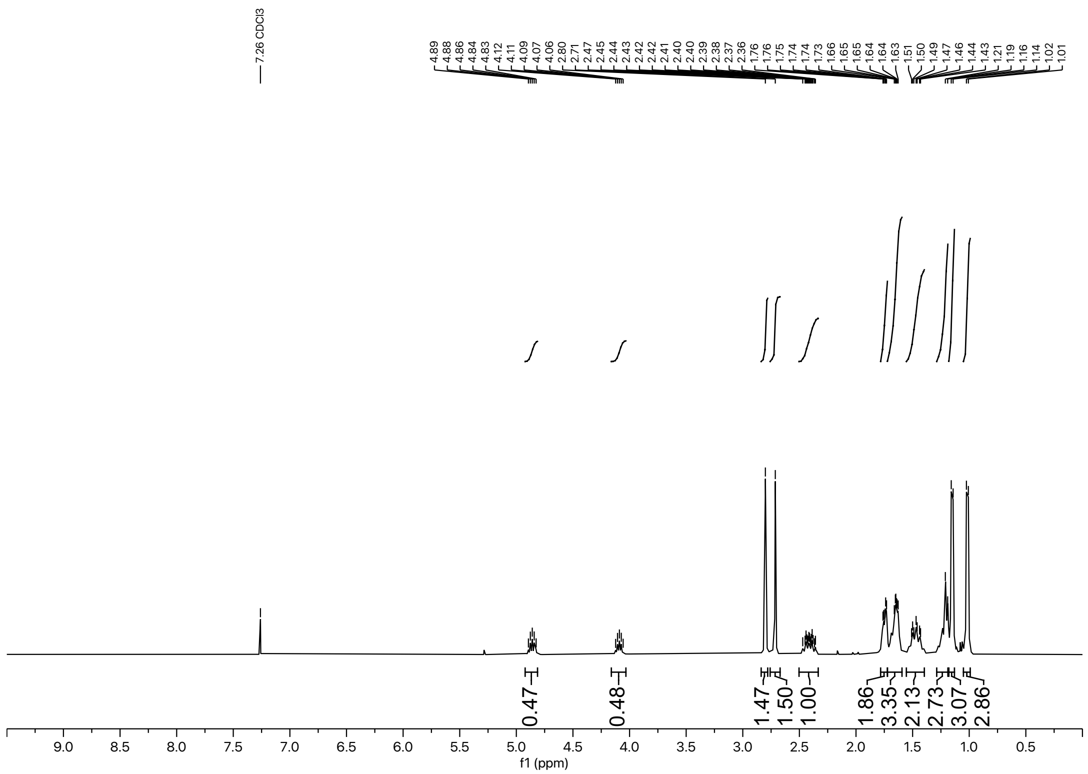

^{13}C NMR (1p)

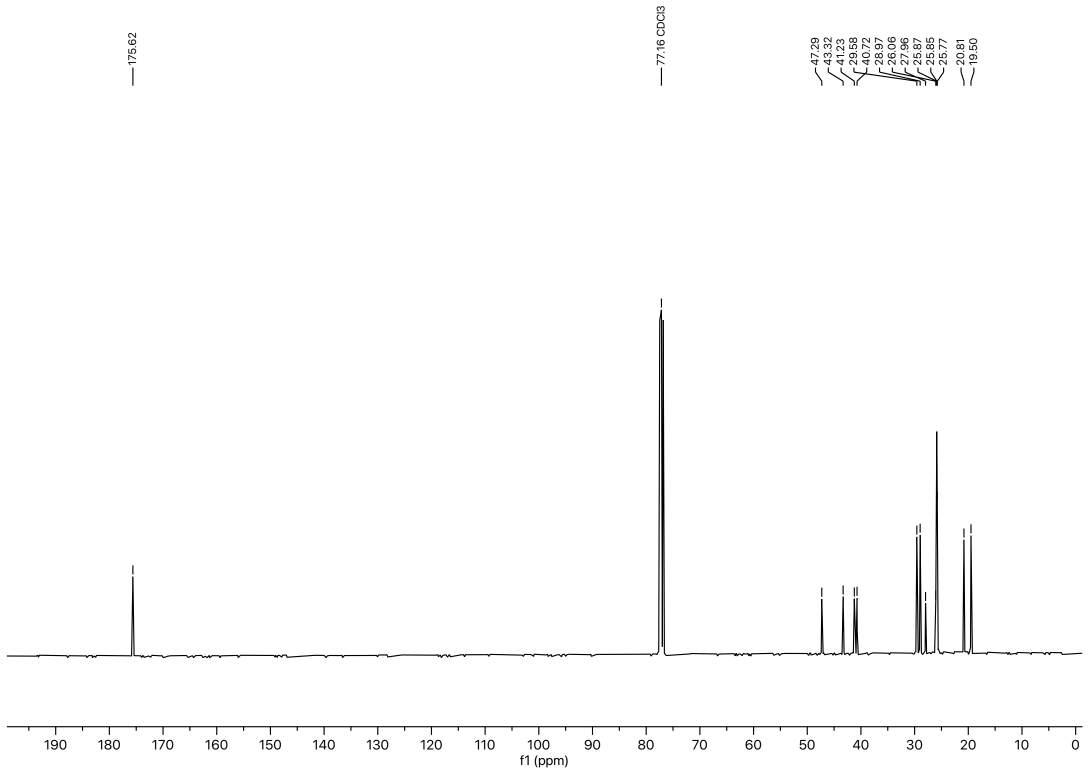

^{13}C DEPT-135 (1p)

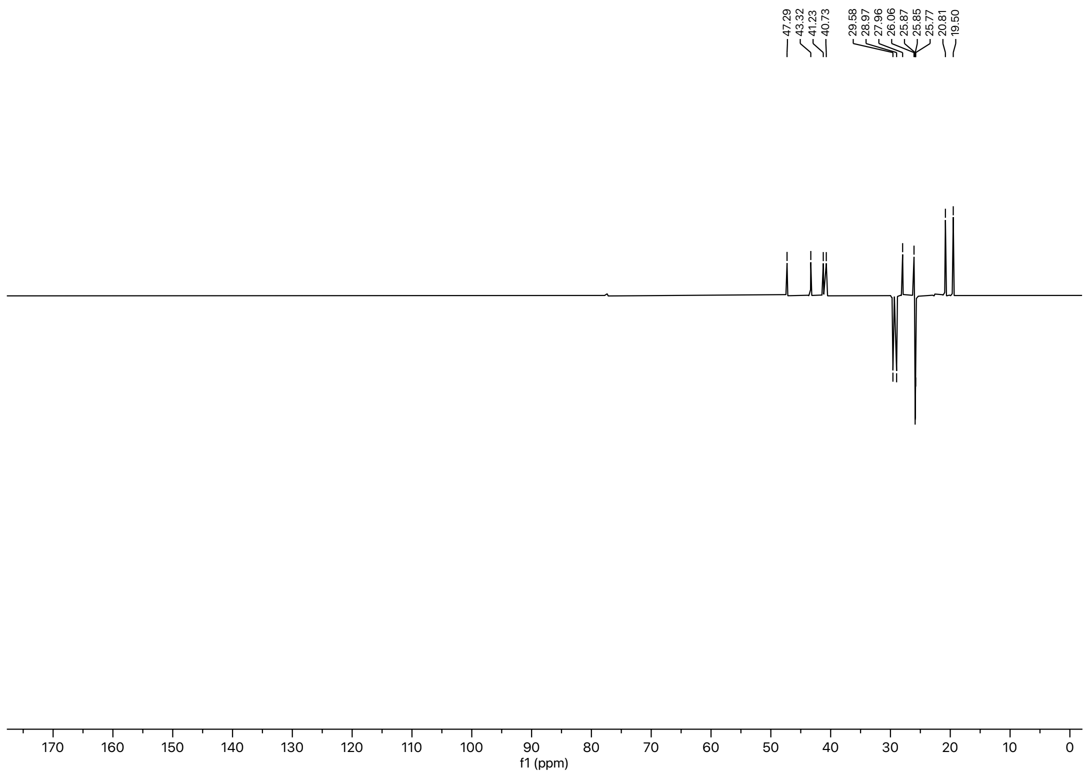

^{1}H NMR (1q)

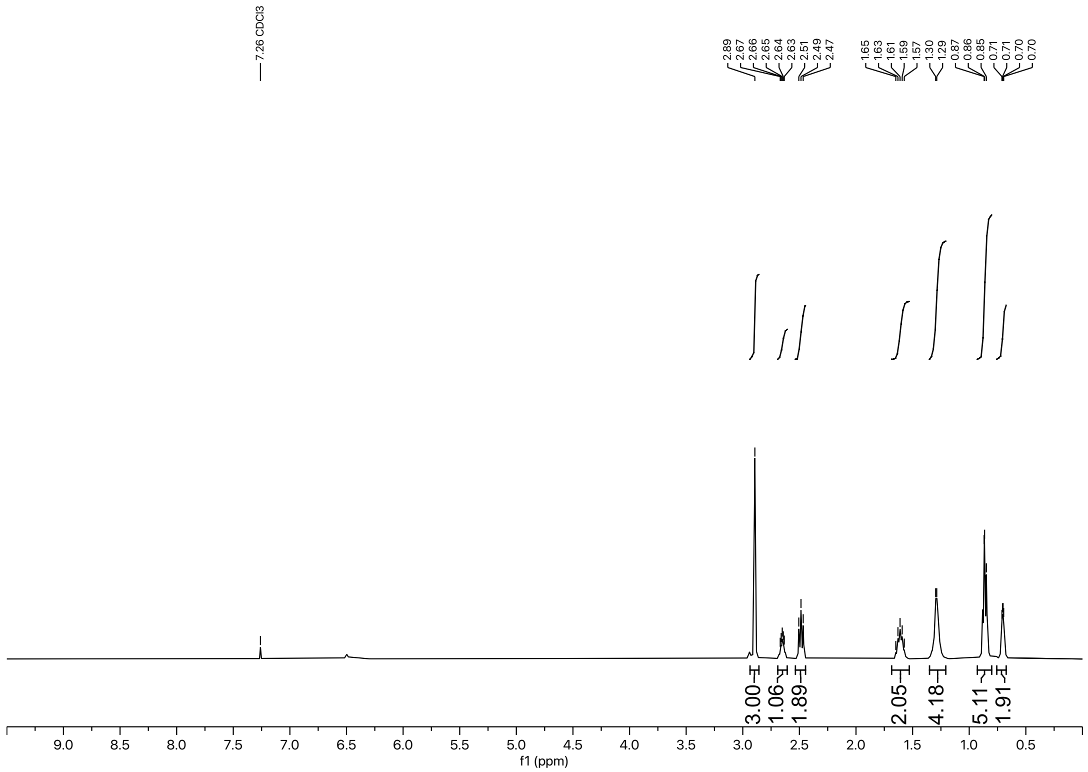

^{13}C NMR (1q)

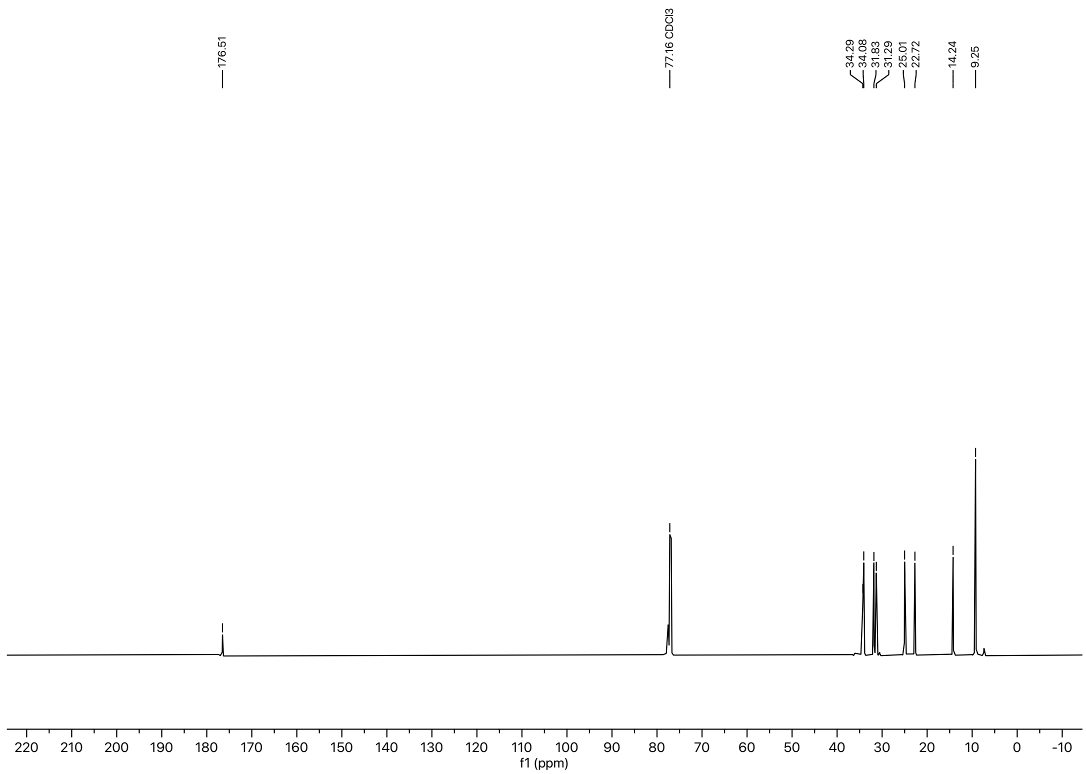

^{13}C DEPT-135 (1q)

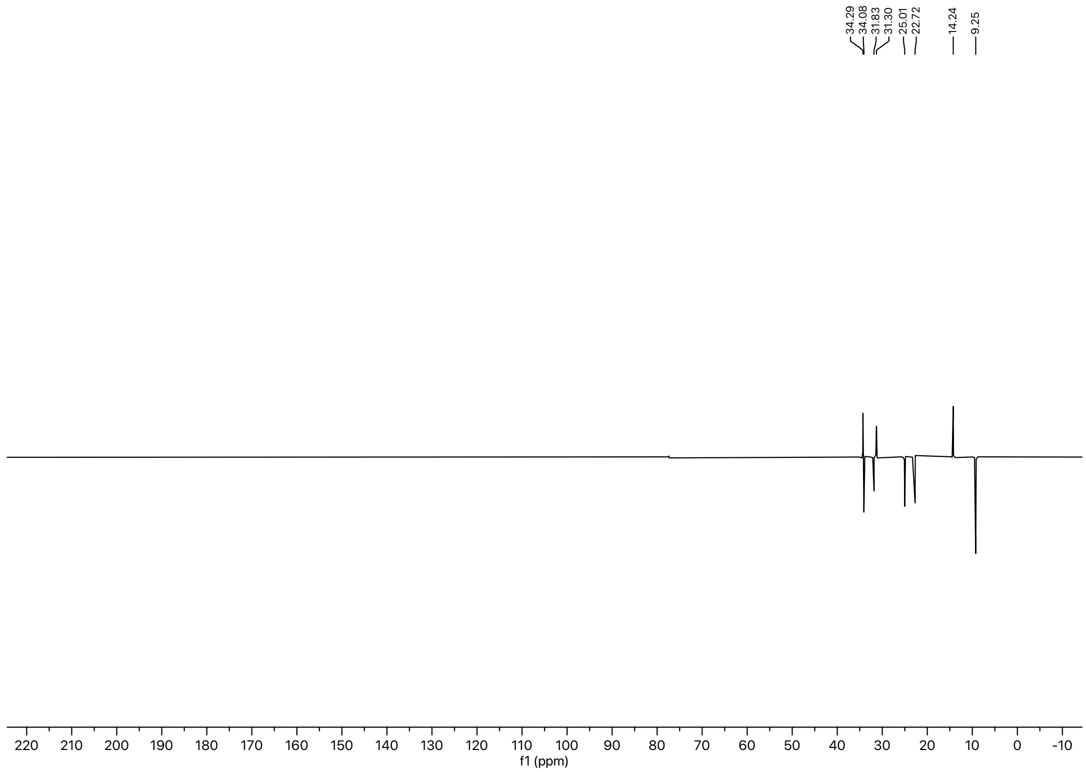

^{1}H NMR (1s)

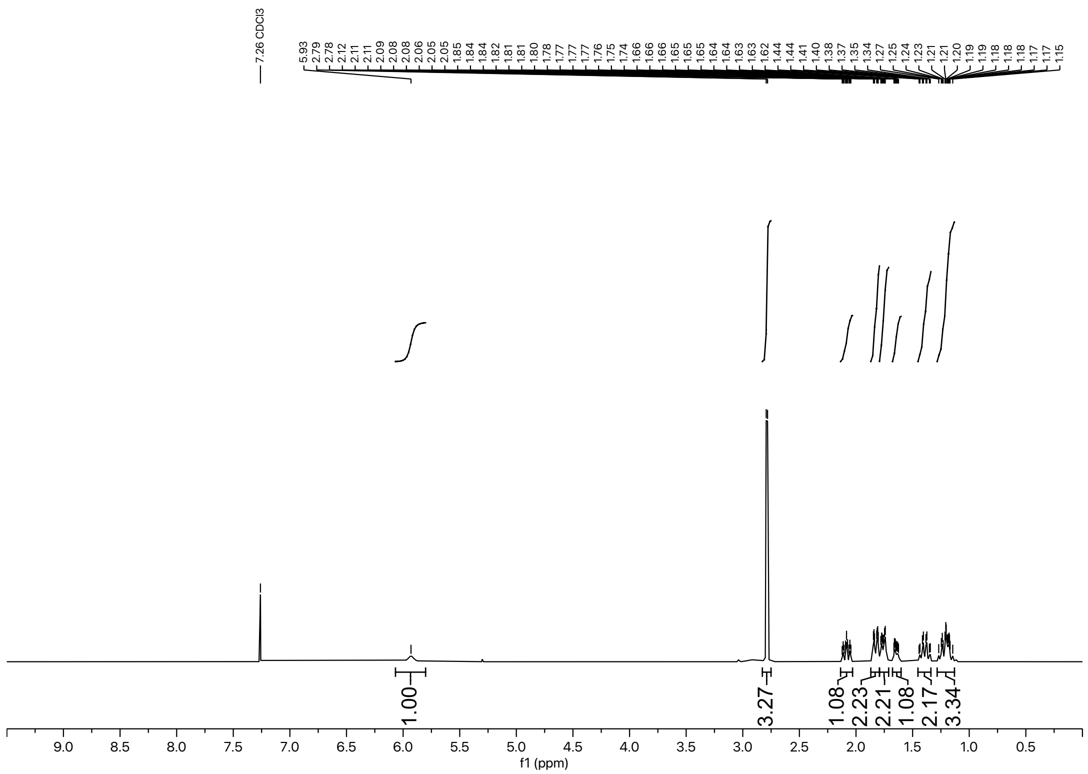

^{13}C NMR (1s)

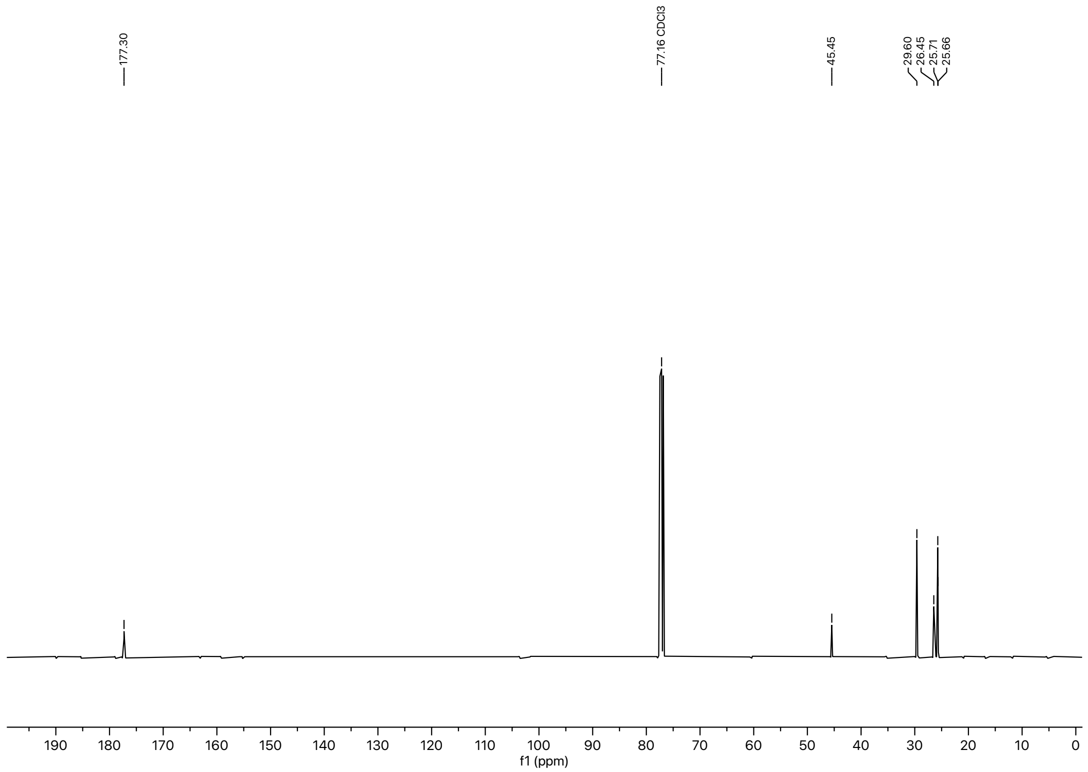

^{13}C DEPT-135 (1s)

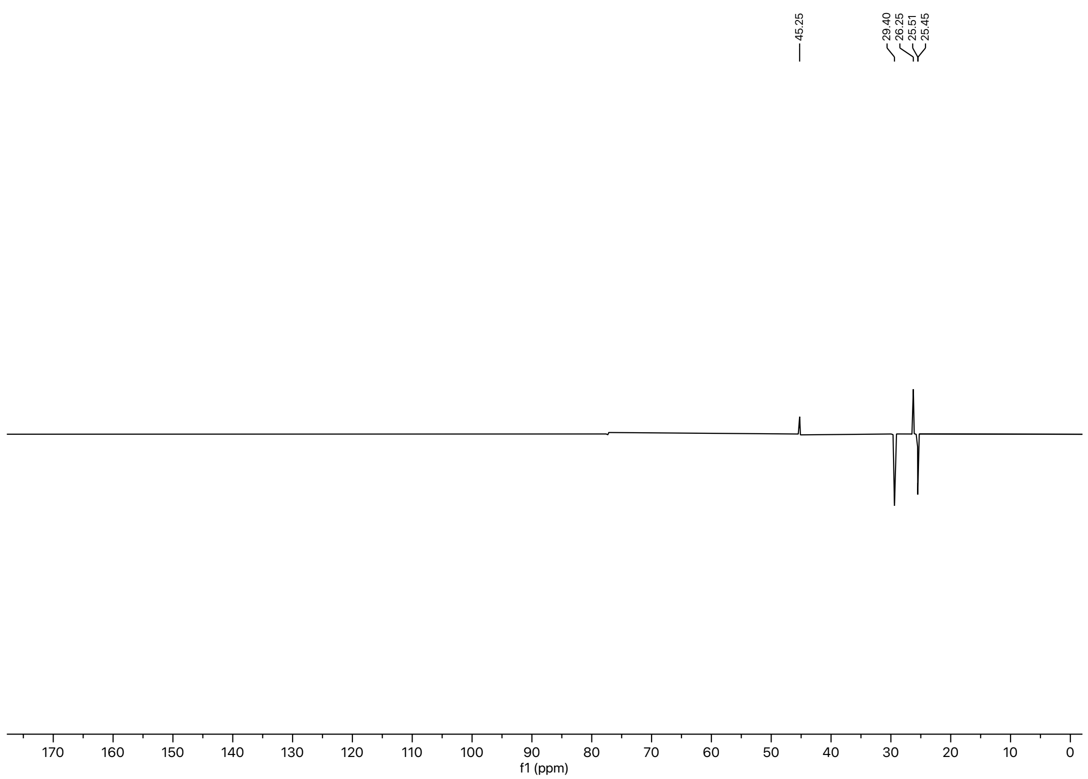

^{1}H NMR (1t)

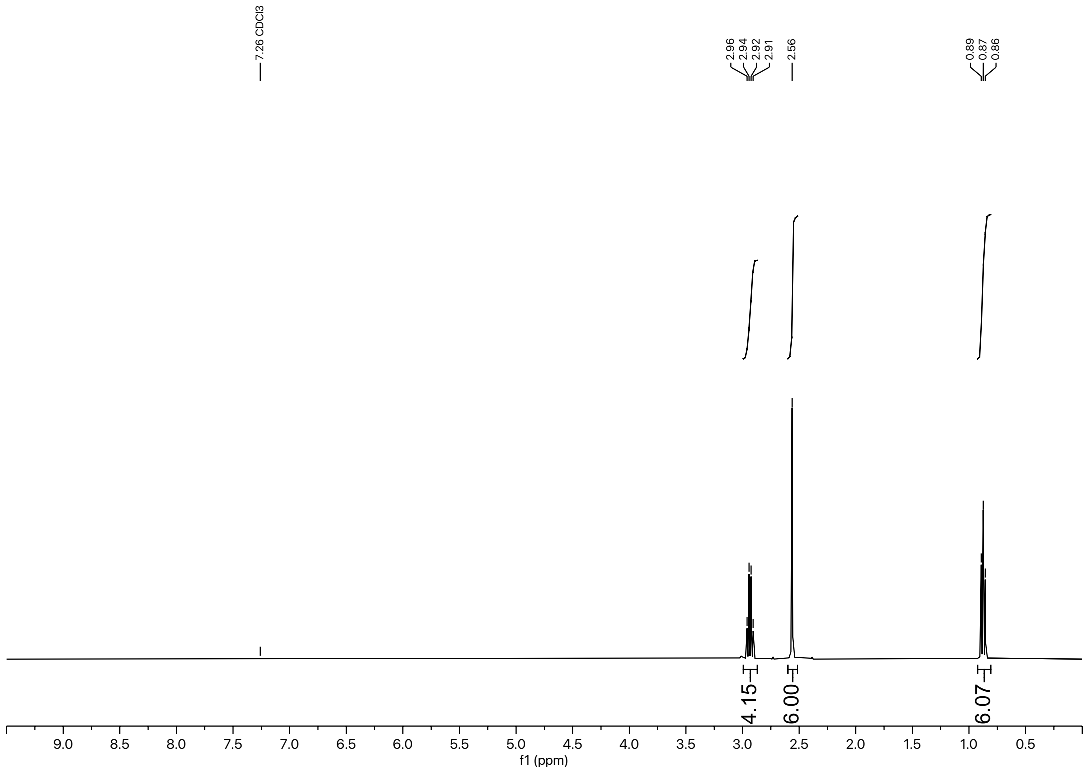

^{13}C NMR (1t)

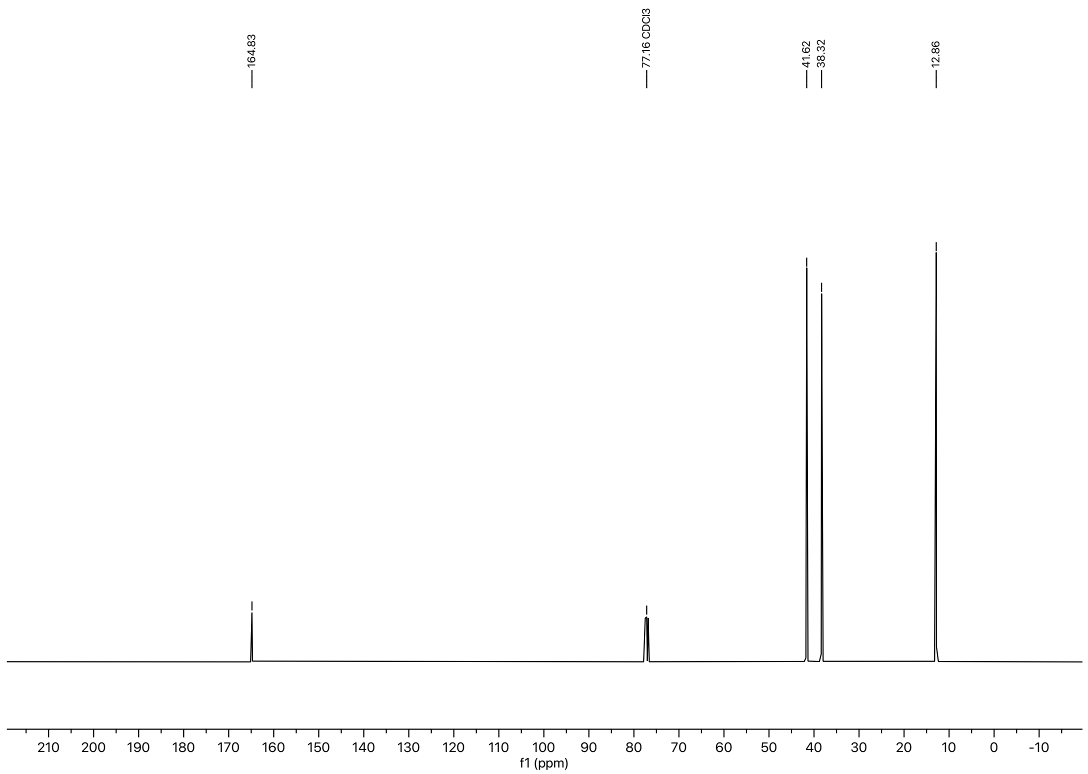

^{13}C DEPT-135 (1t)

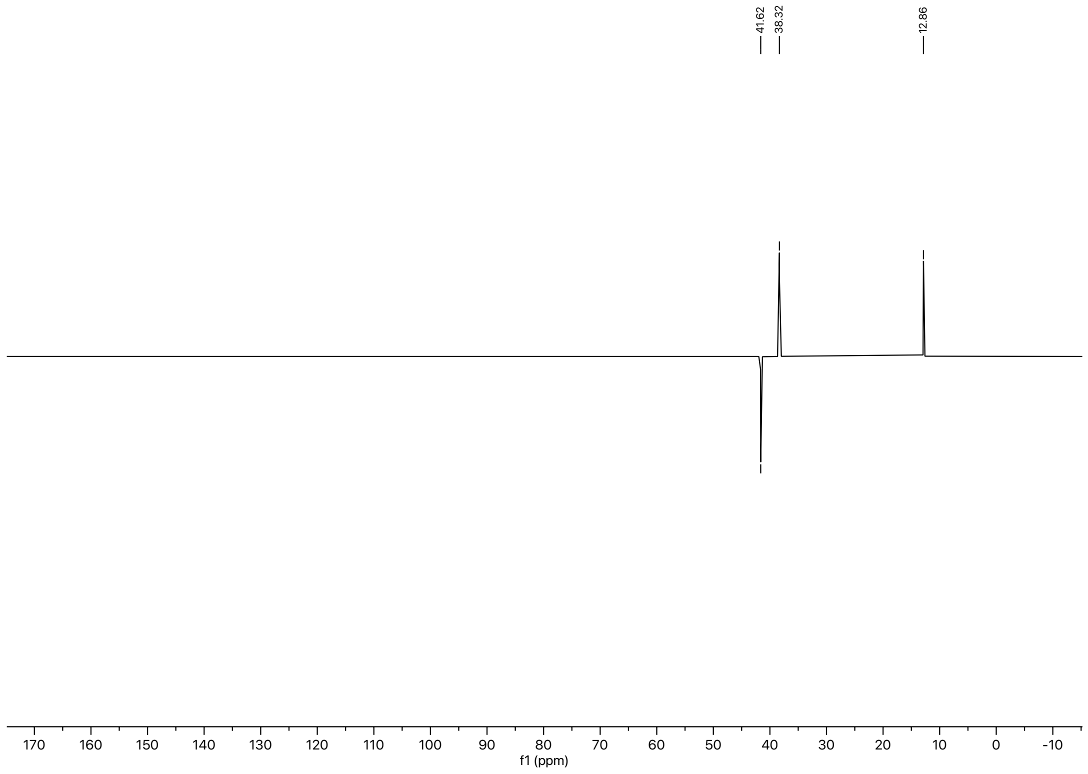

^{1}H NMR (1u)

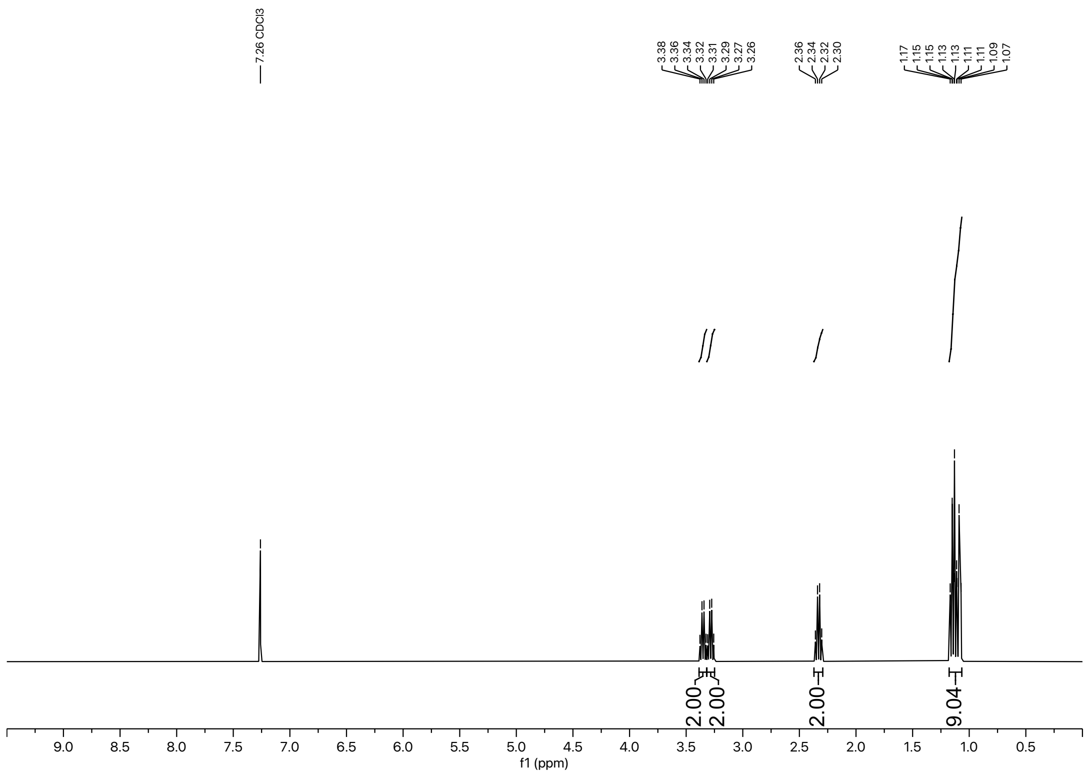

^{13}C NMR (1u)

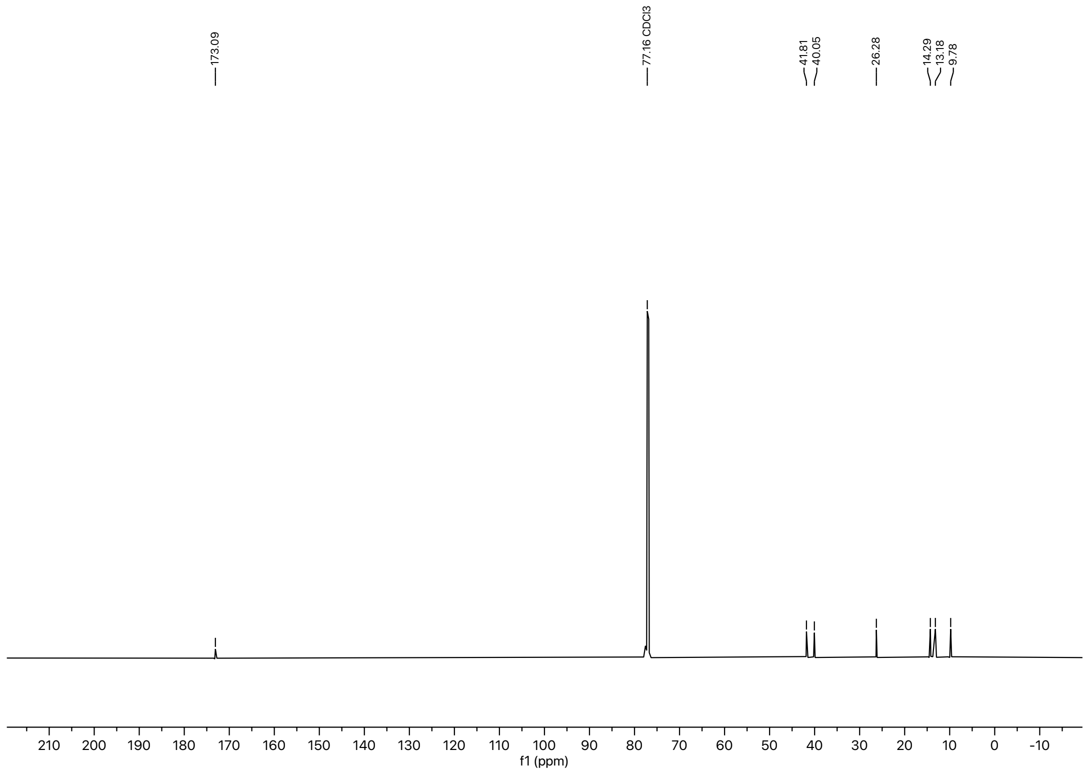

^{13}C DEPT-135 (1u)

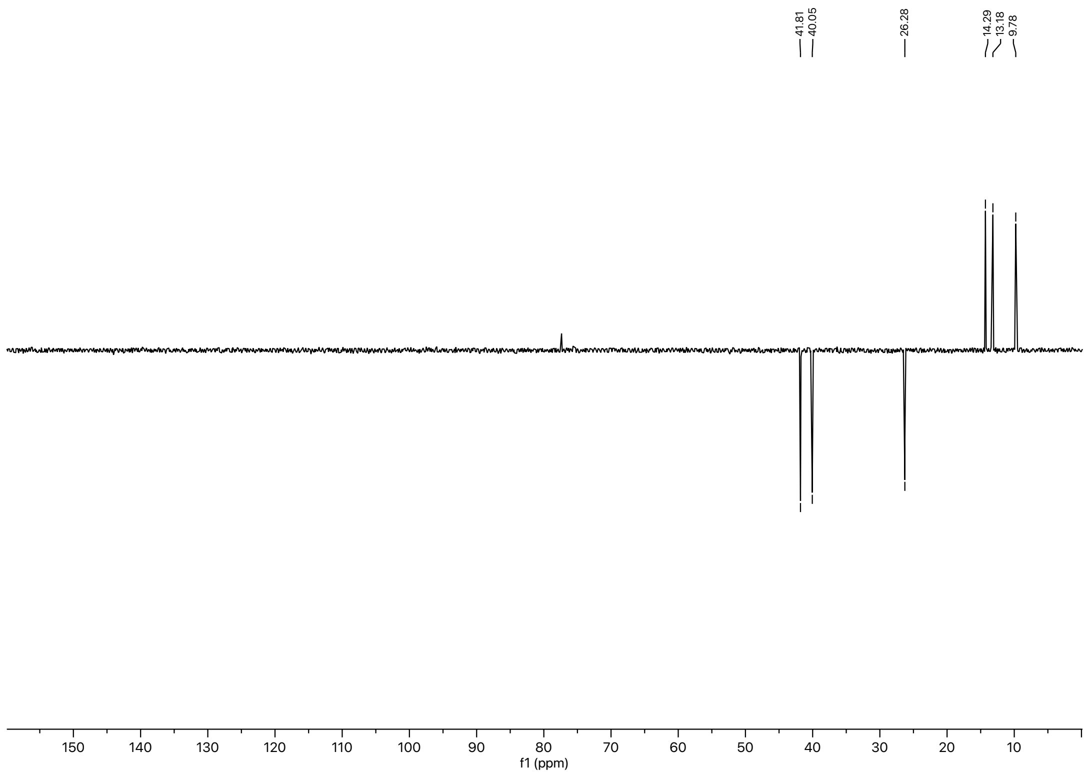

^{1}H NMR (1x)

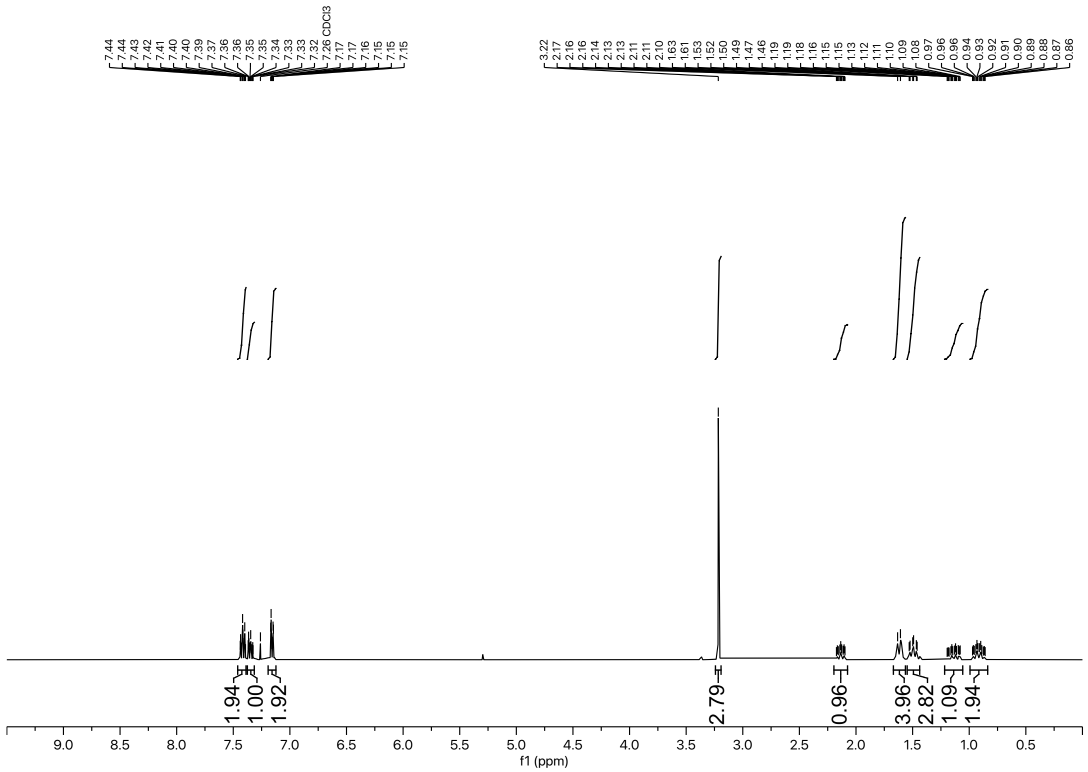

^{13}C NMR (1x)

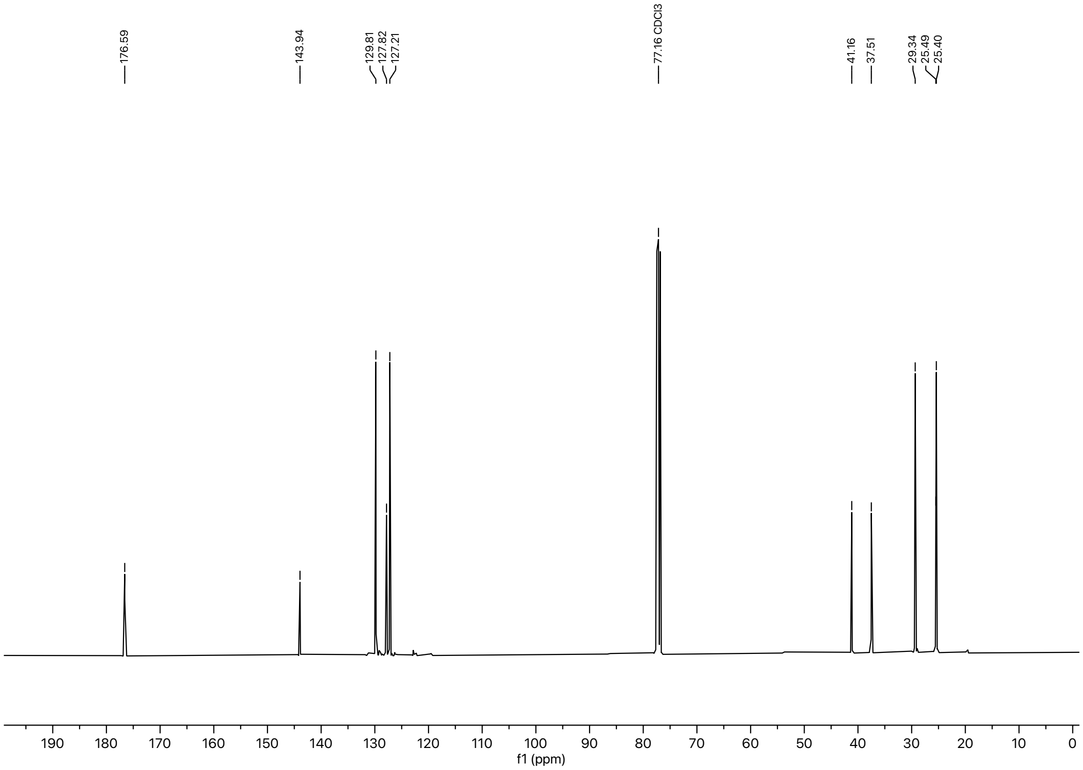

^{13}C DEPT-135 (1x)

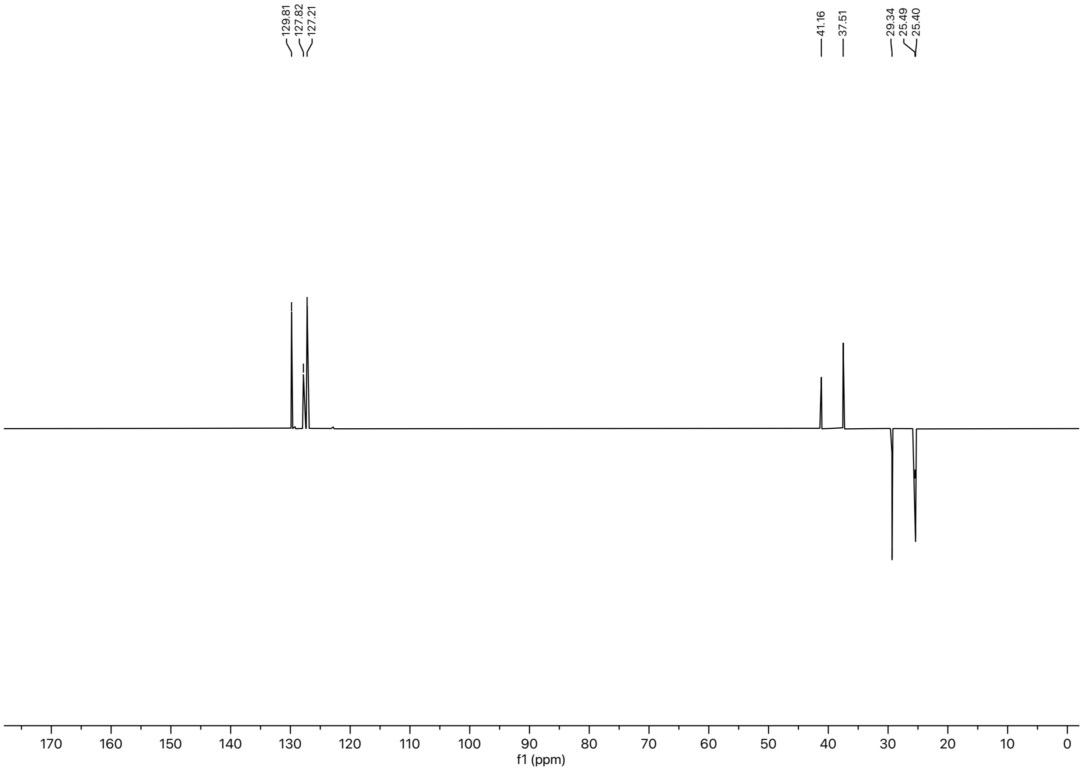

^{1}H NMR (1y)

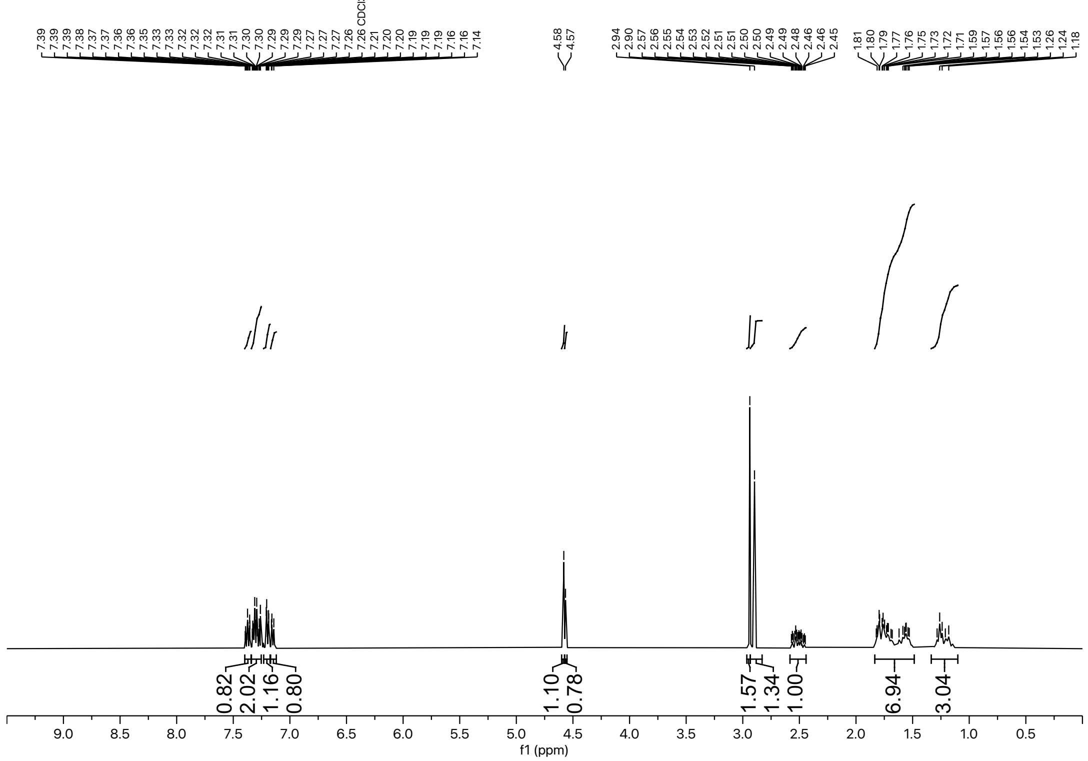

^{13}C NMR (1y)

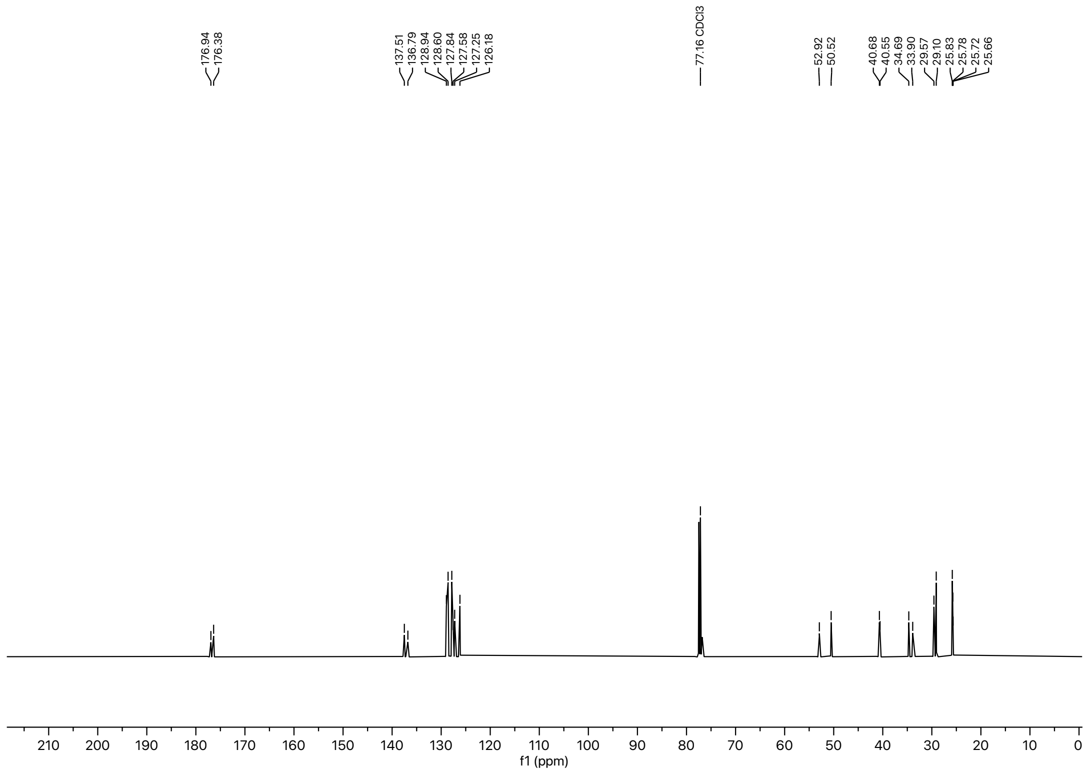

^{13}C DEPT-135 (1y)

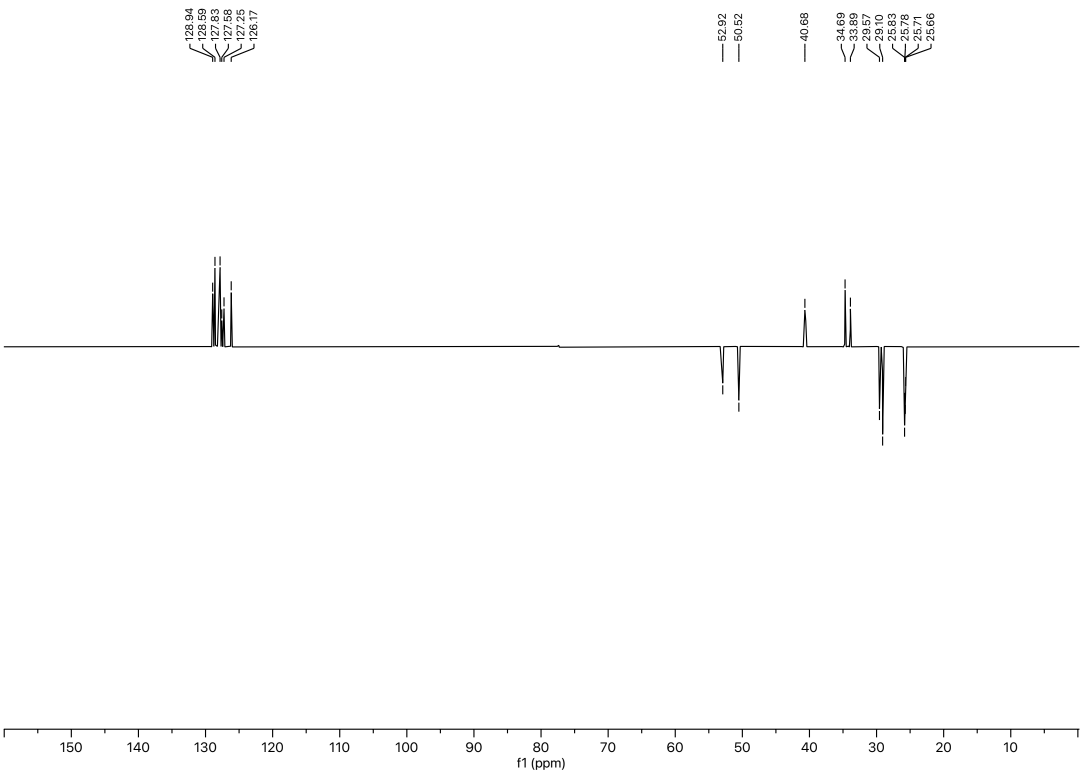

^{1}H NMR (1z)

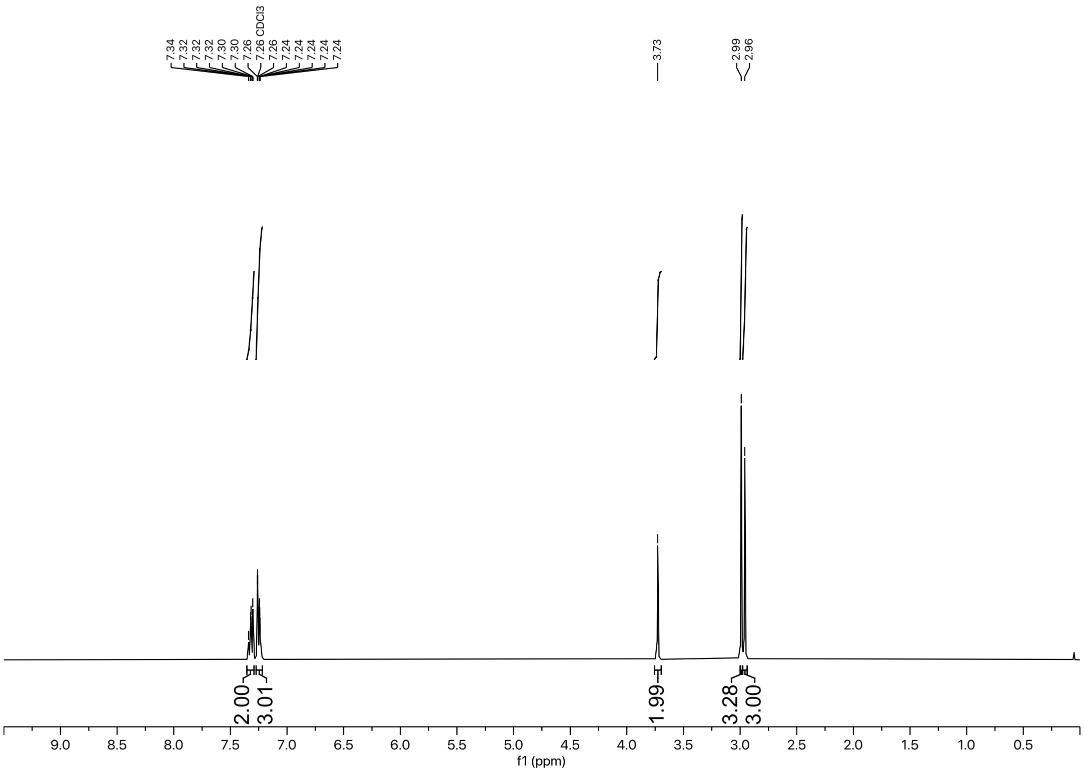

^{13}C NMR (1z)

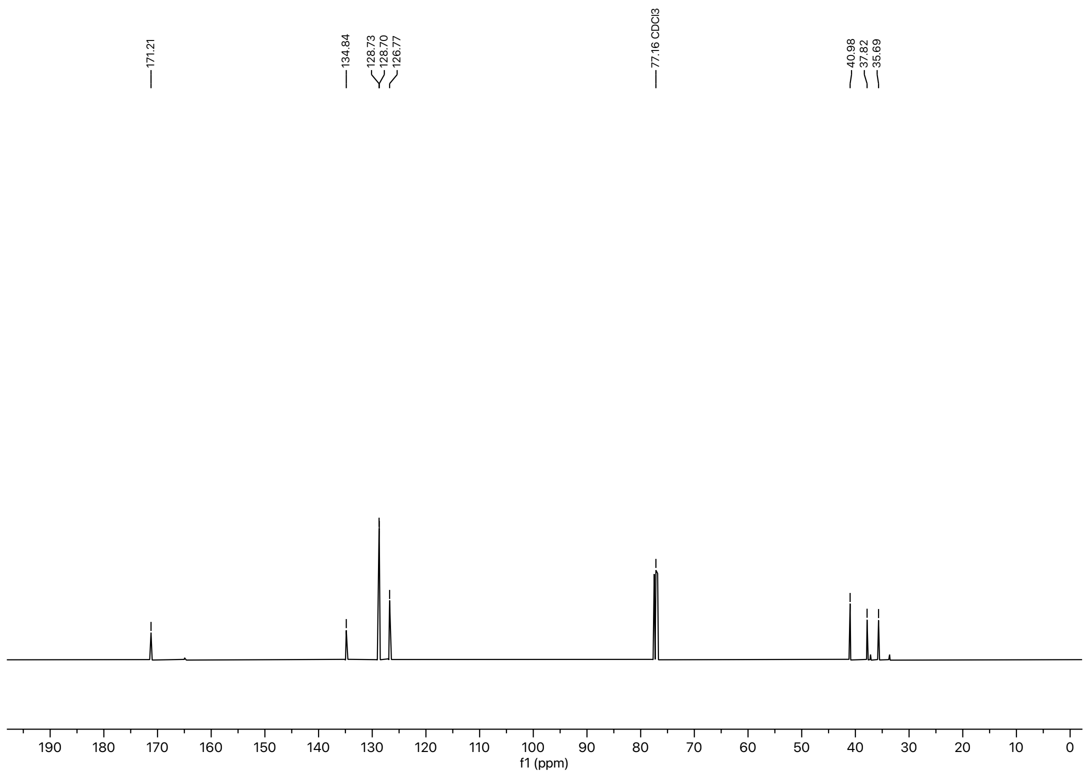

^{13}C DEPT-135 (1z)

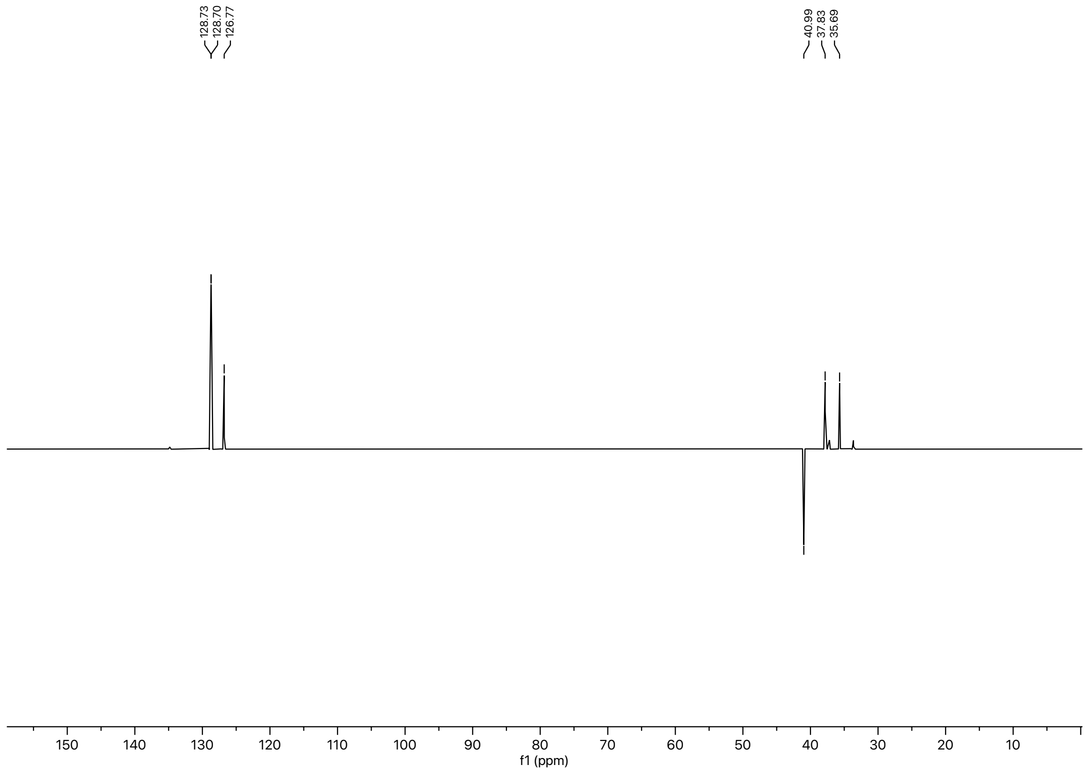

^{1}H NMR (1aa)

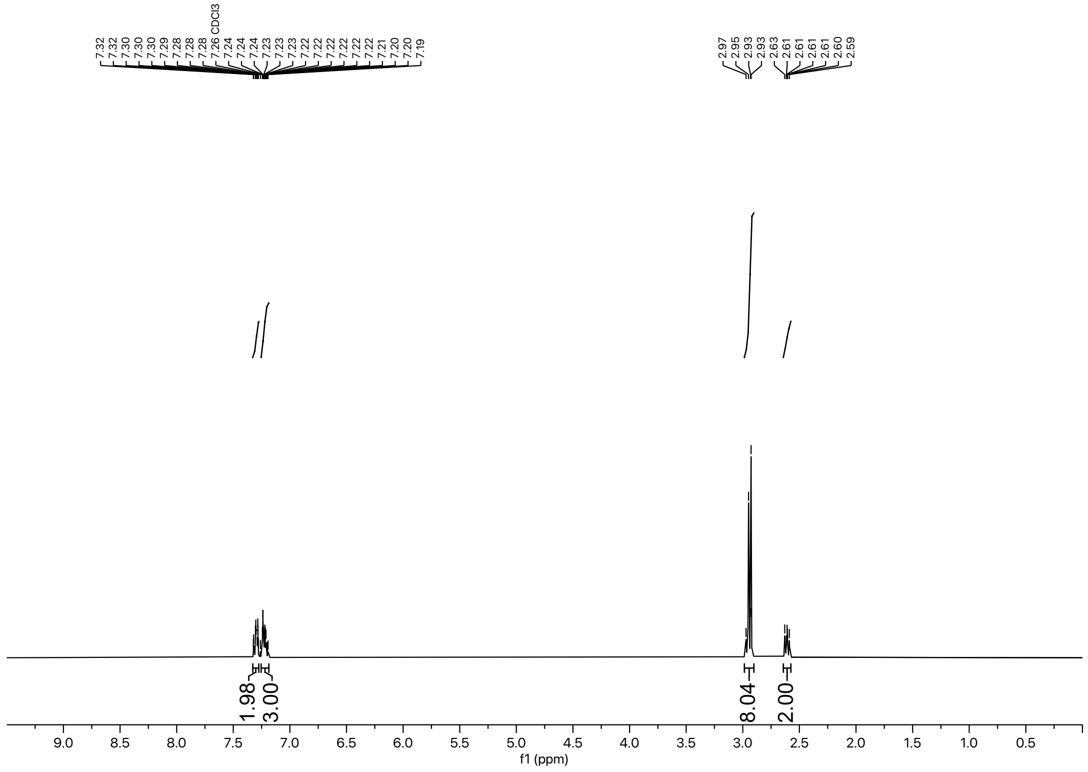

^{13}C NMR (1aa)

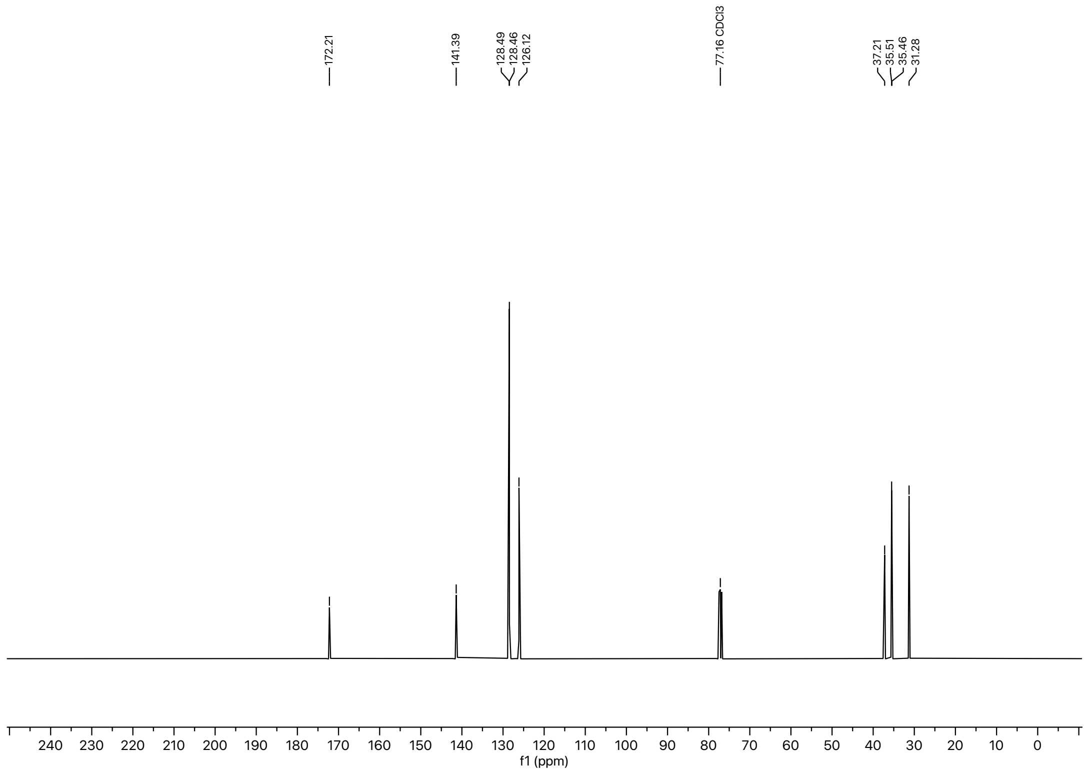

^{13}C DEPT-135 (1aa)

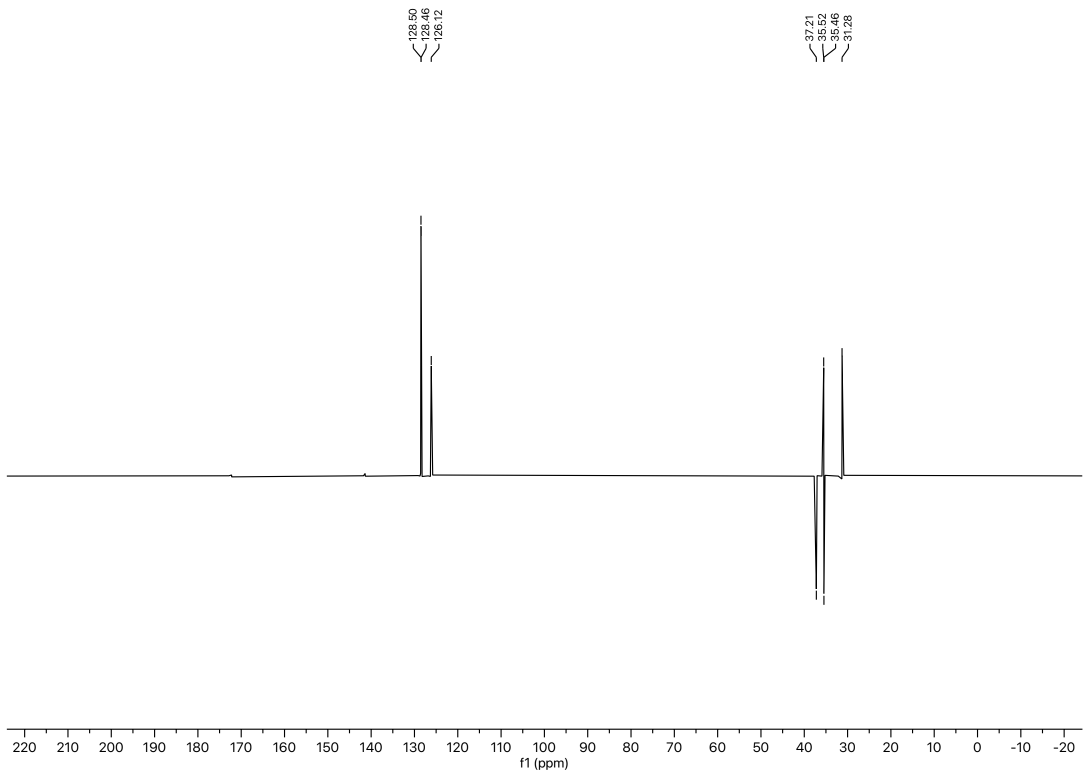

^{1}H NMR (1ab)

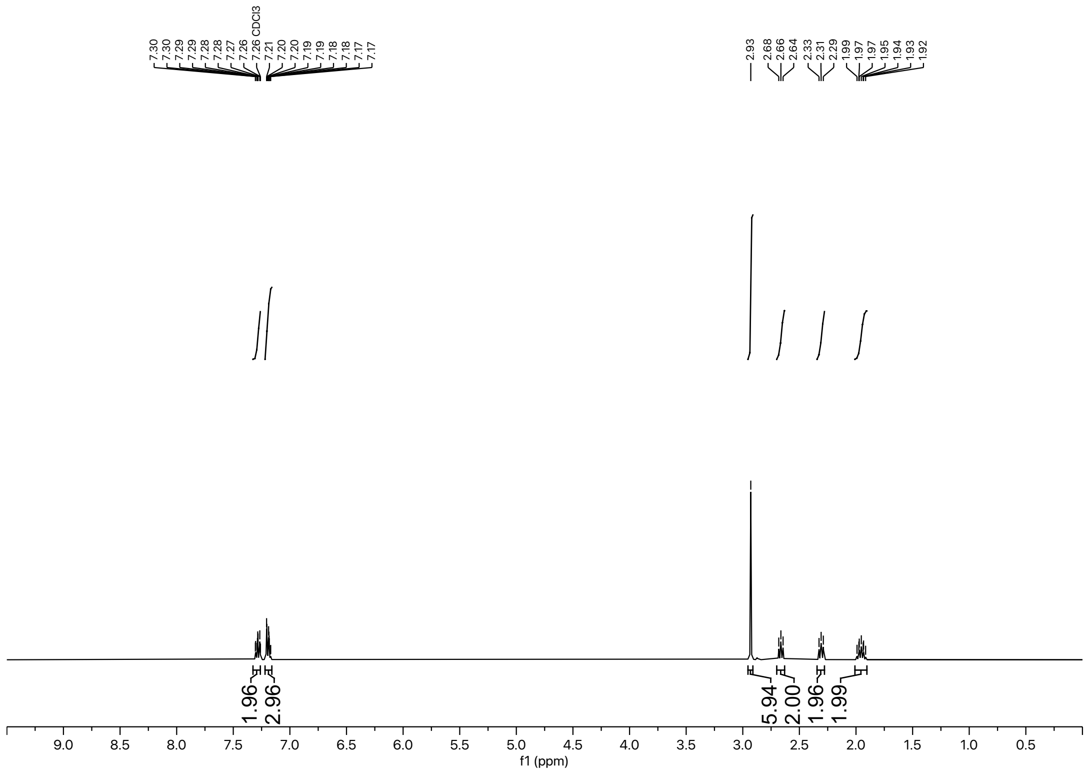

^{13}C NMR (1ab)

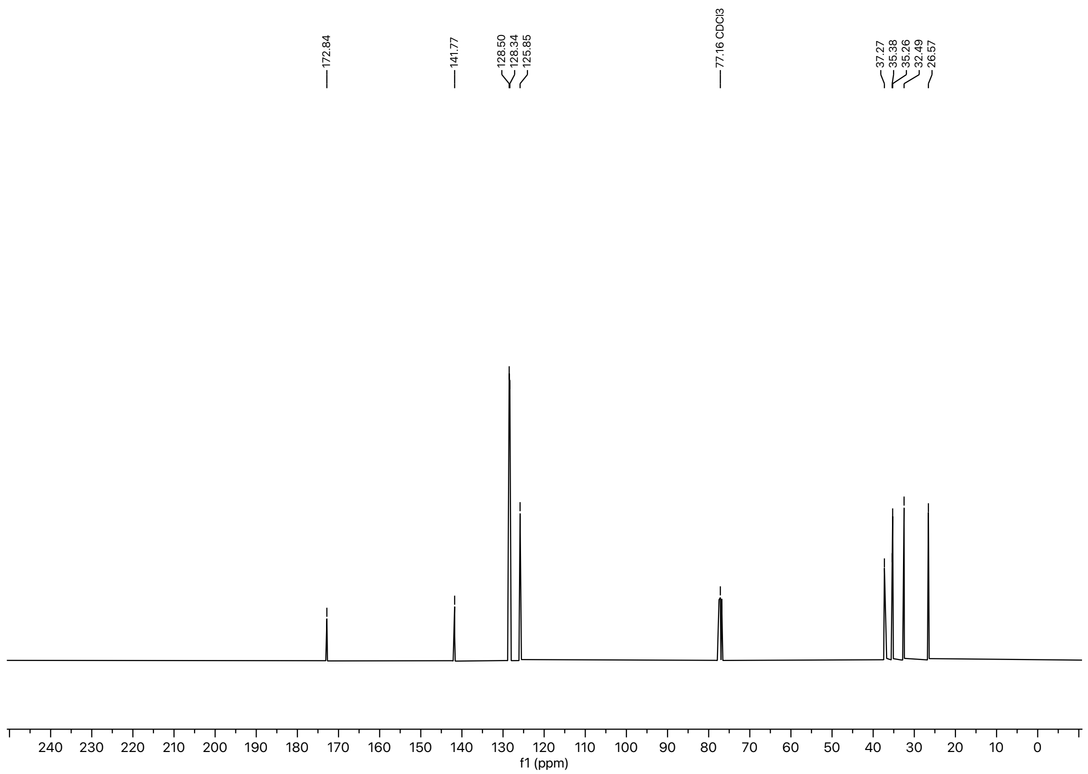

^{13}C DEPT-135 (1ab)

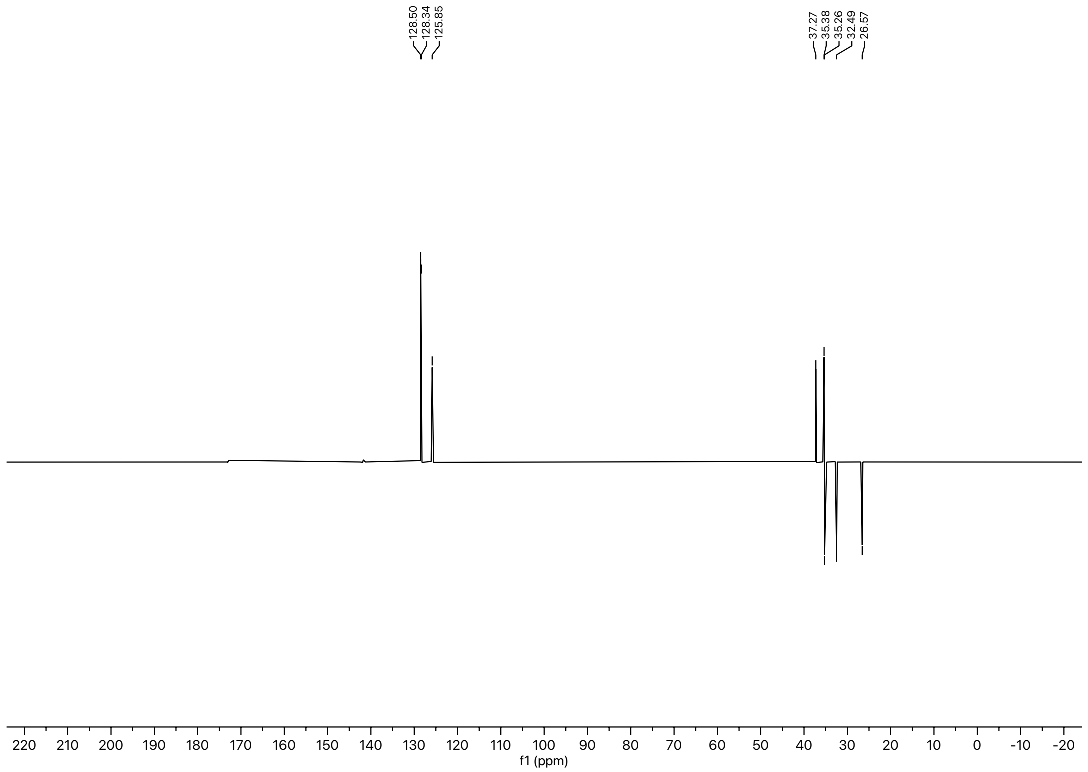

^1H NMR (1ac)

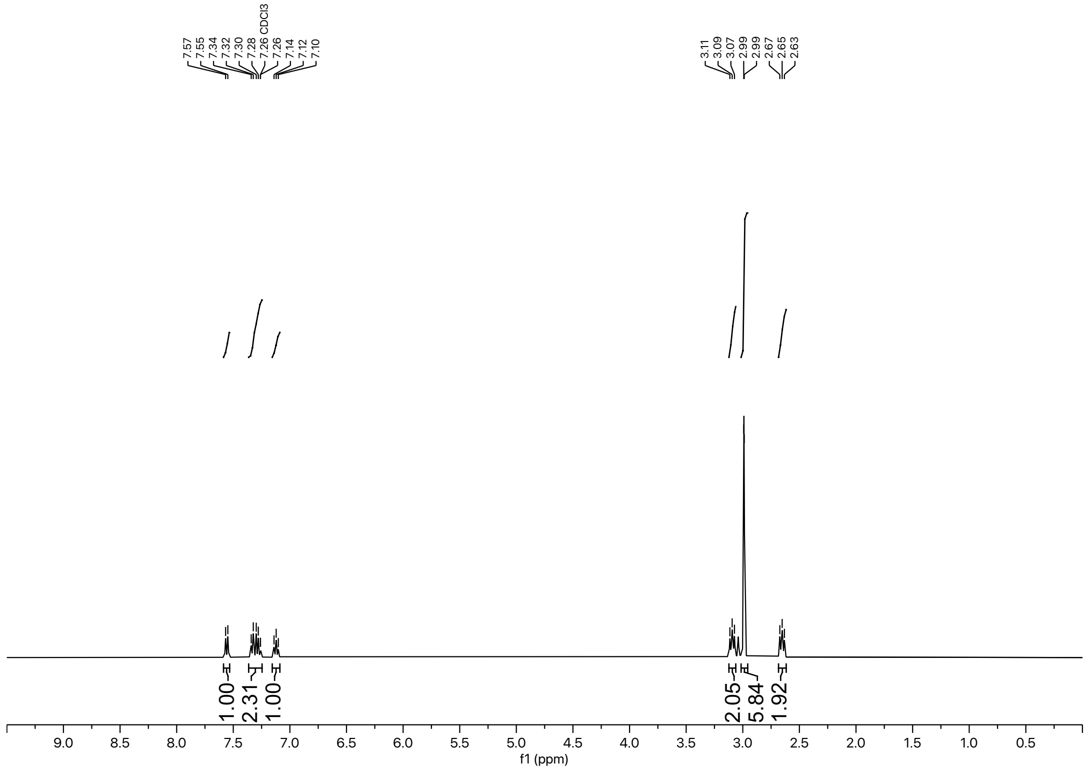

^{13}C NMR (1ac)

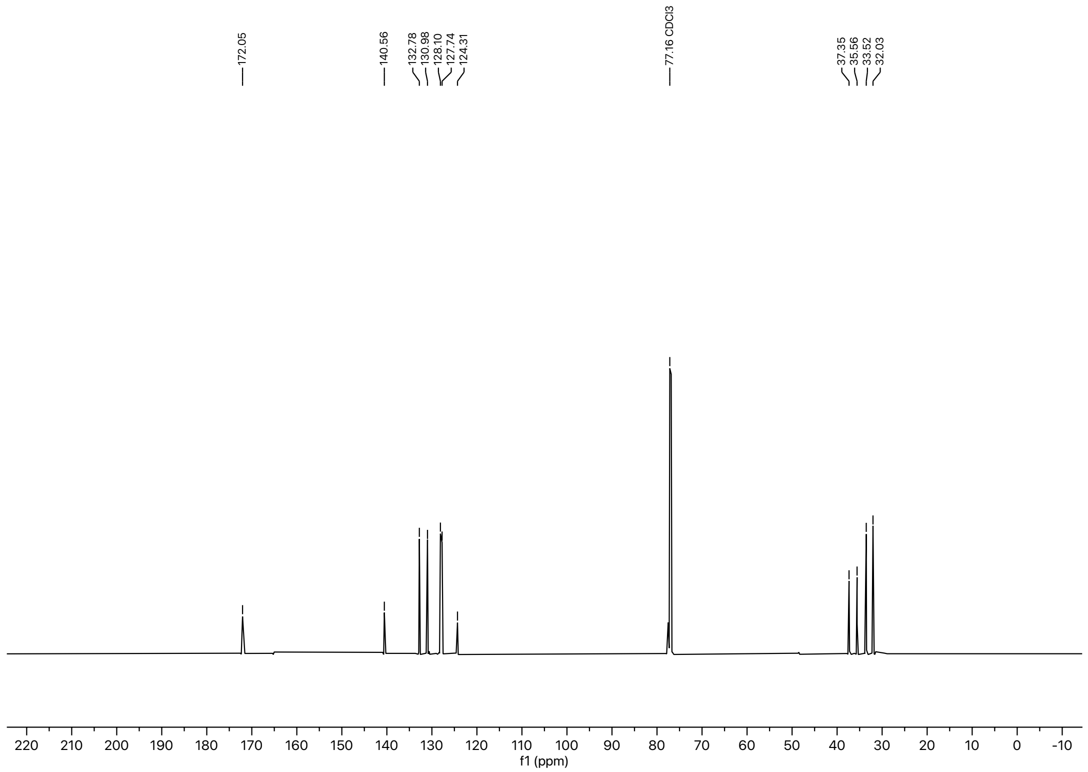

^{13}C DEPT-135 (1ac)

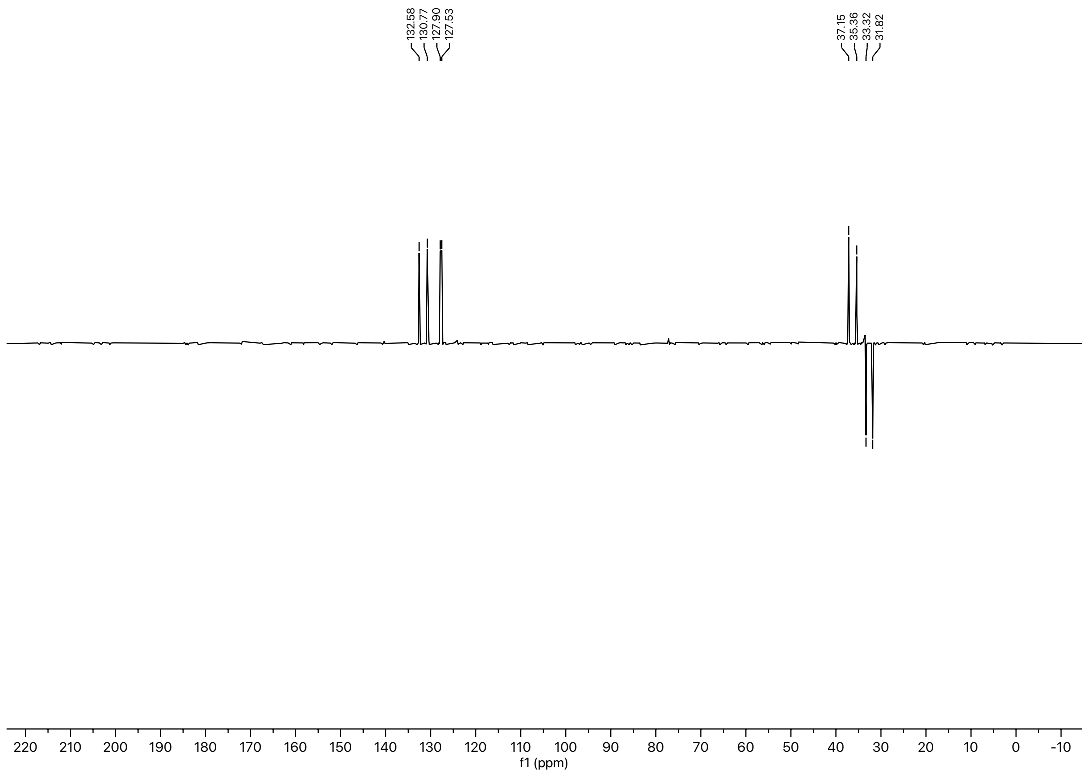

^{1}H NMR (1ad)

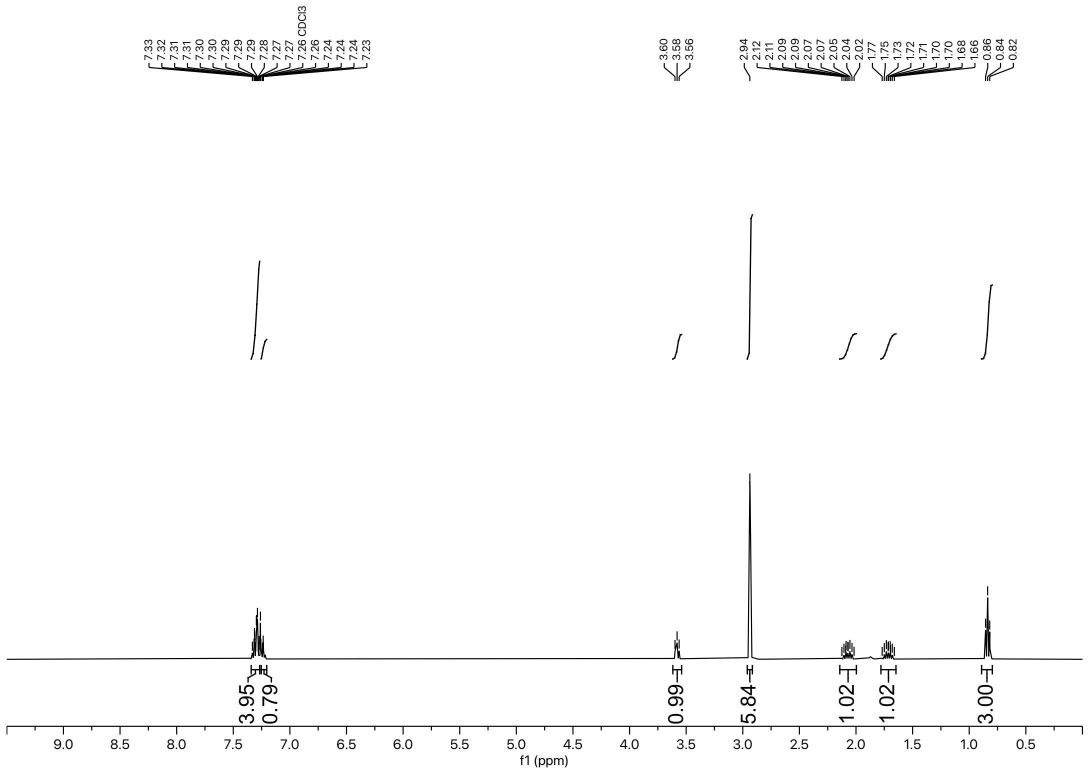

^{13}C NMR (1ad)

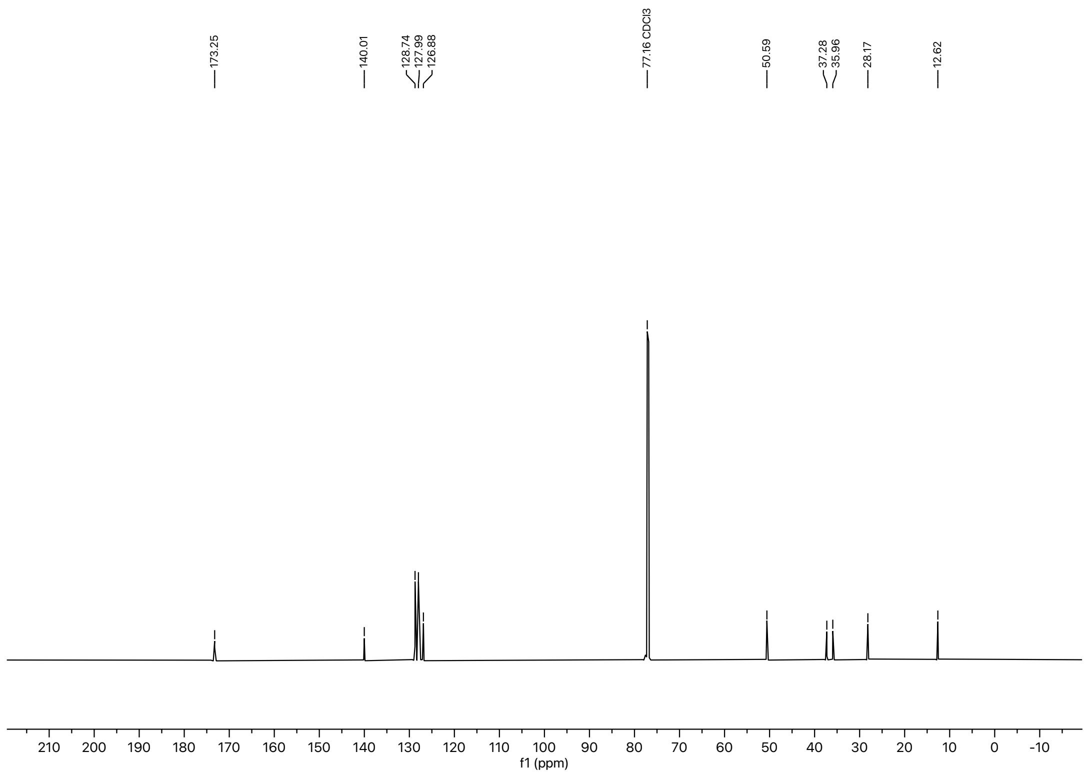

^{13}C DEPT-135 (1ad)

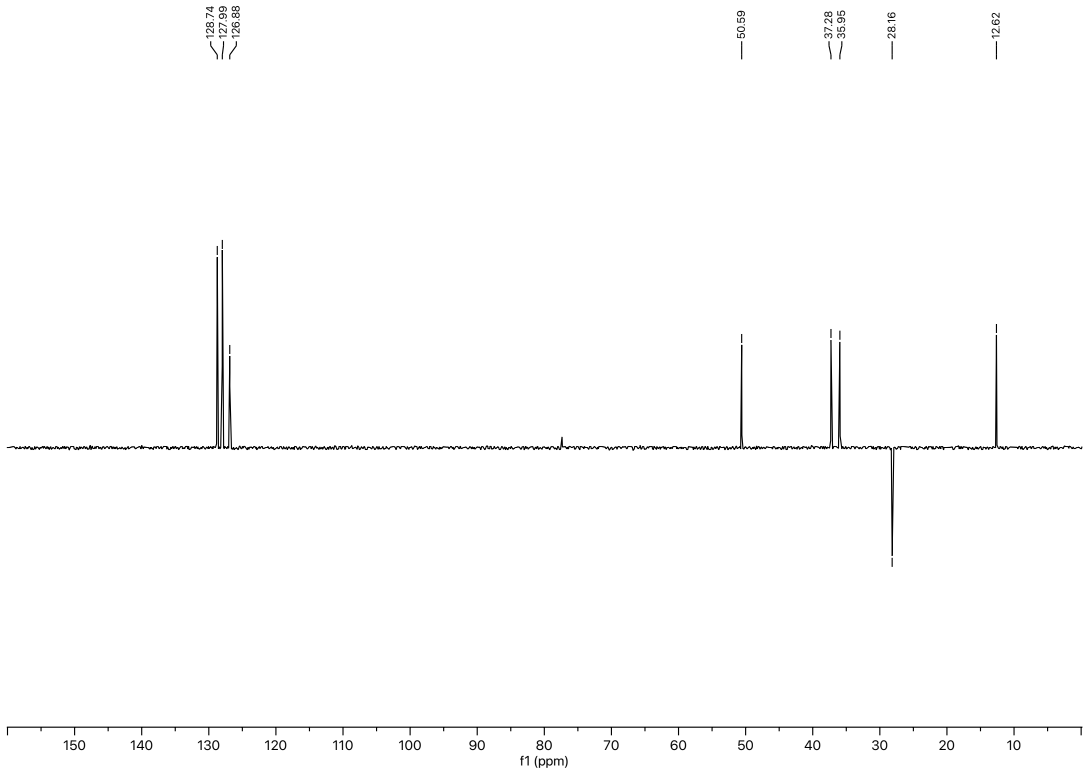

^{1}H NMR (1ae)

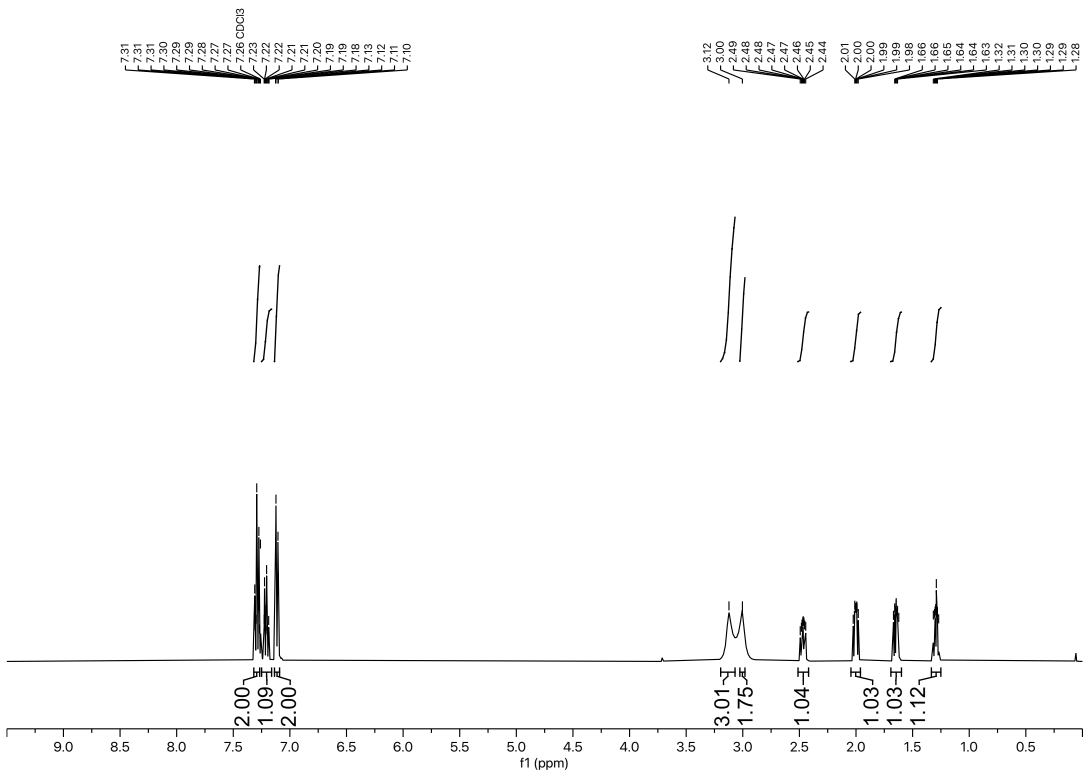

^{13}C NMR (1ae)

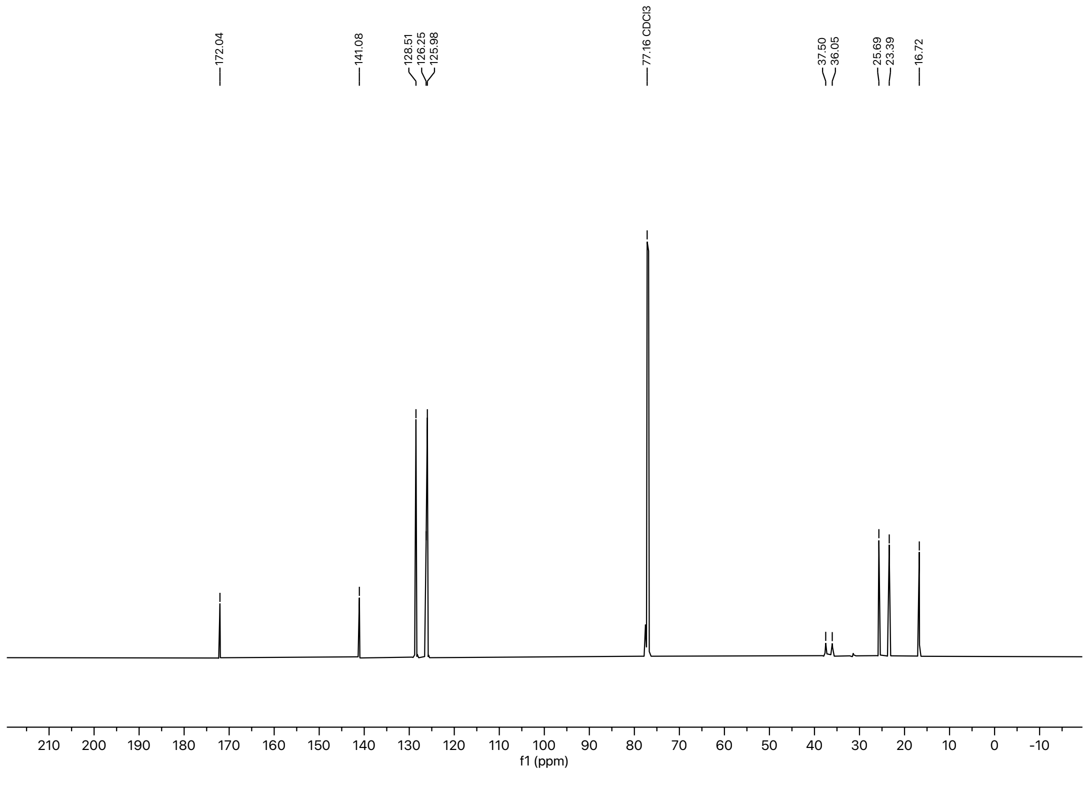

^{13}C DEPT-135 (1ae)

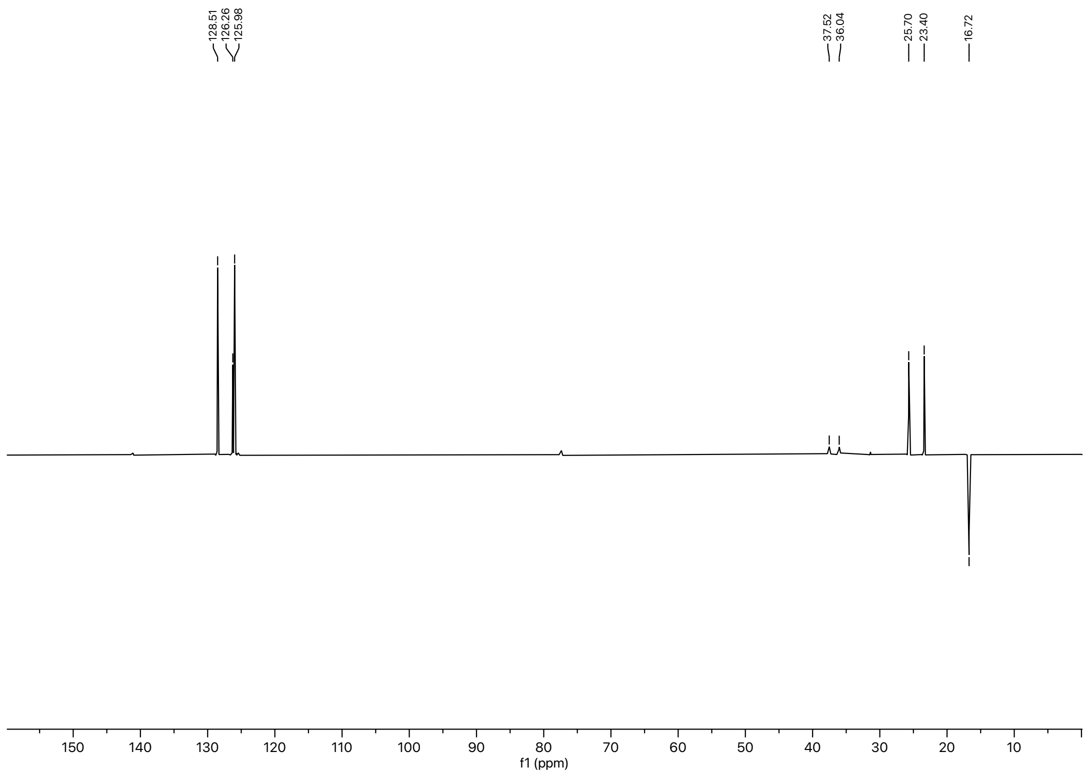

^{1}H NMR (1af)

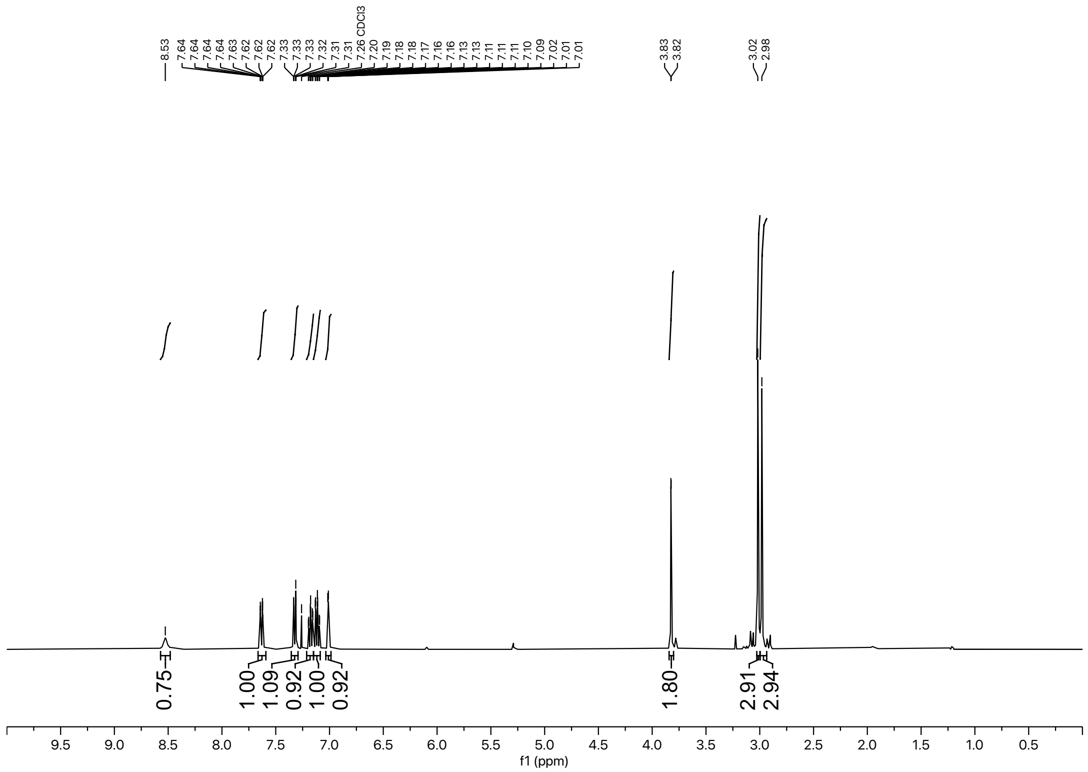

^{13}C NMR (1af)

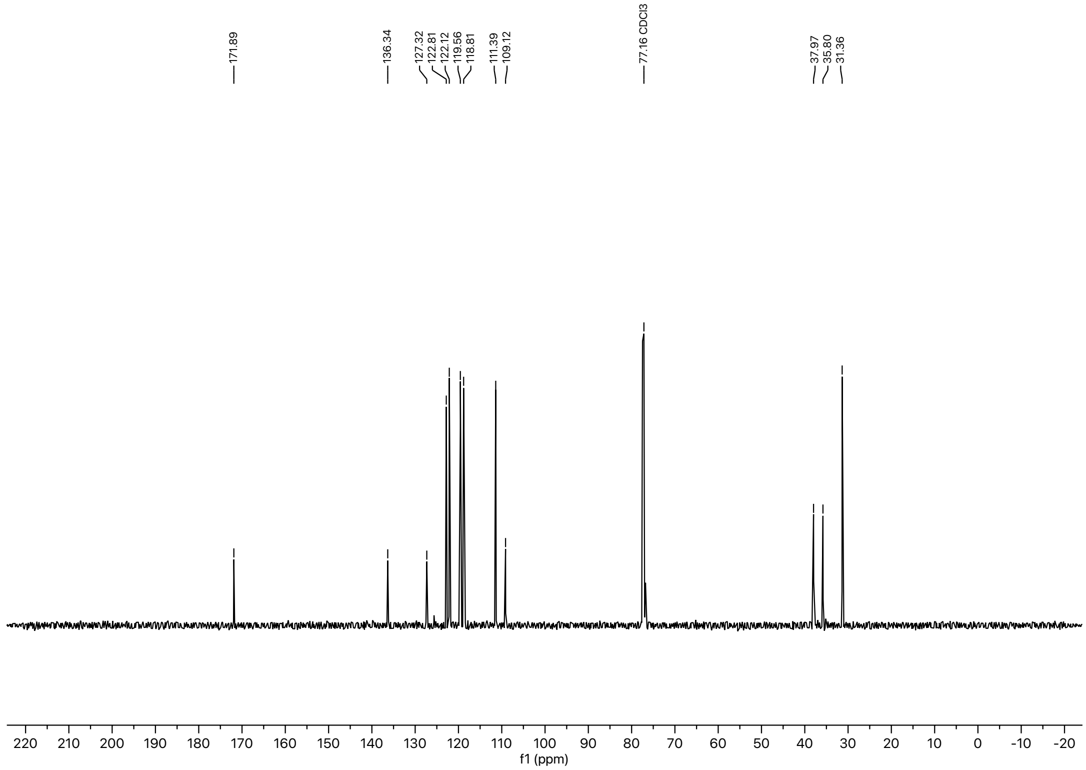

^{13}C DEPT-135 (1af)

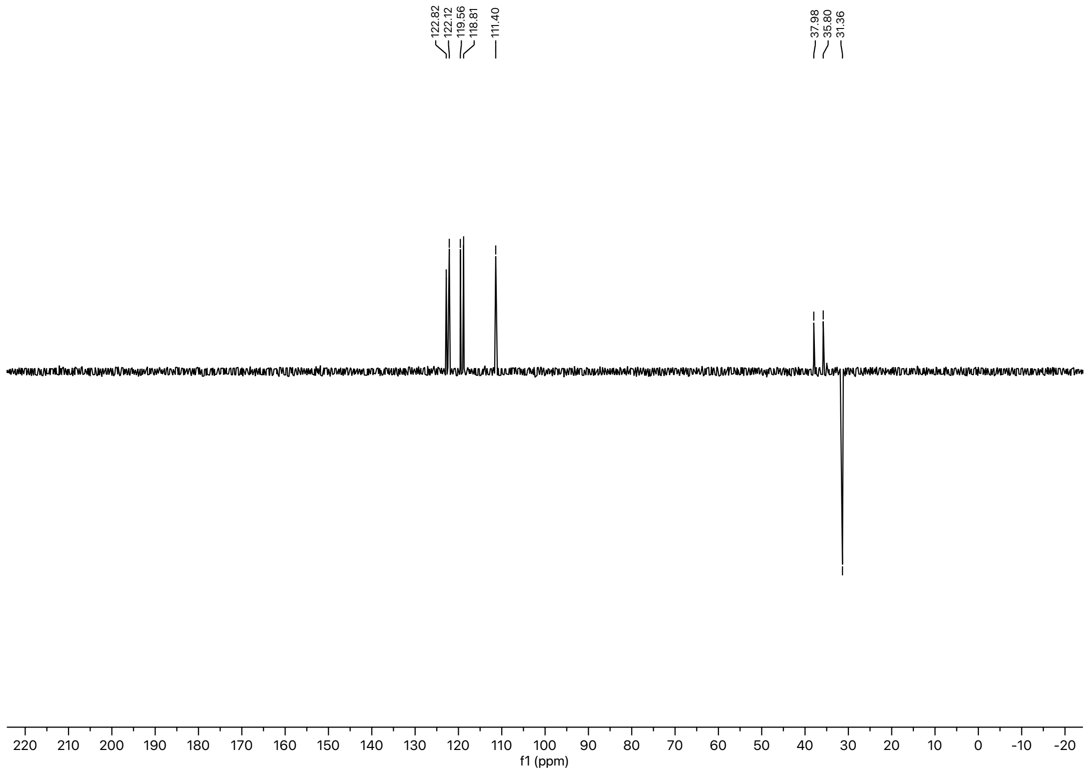

^{1}H NMR (1ag)

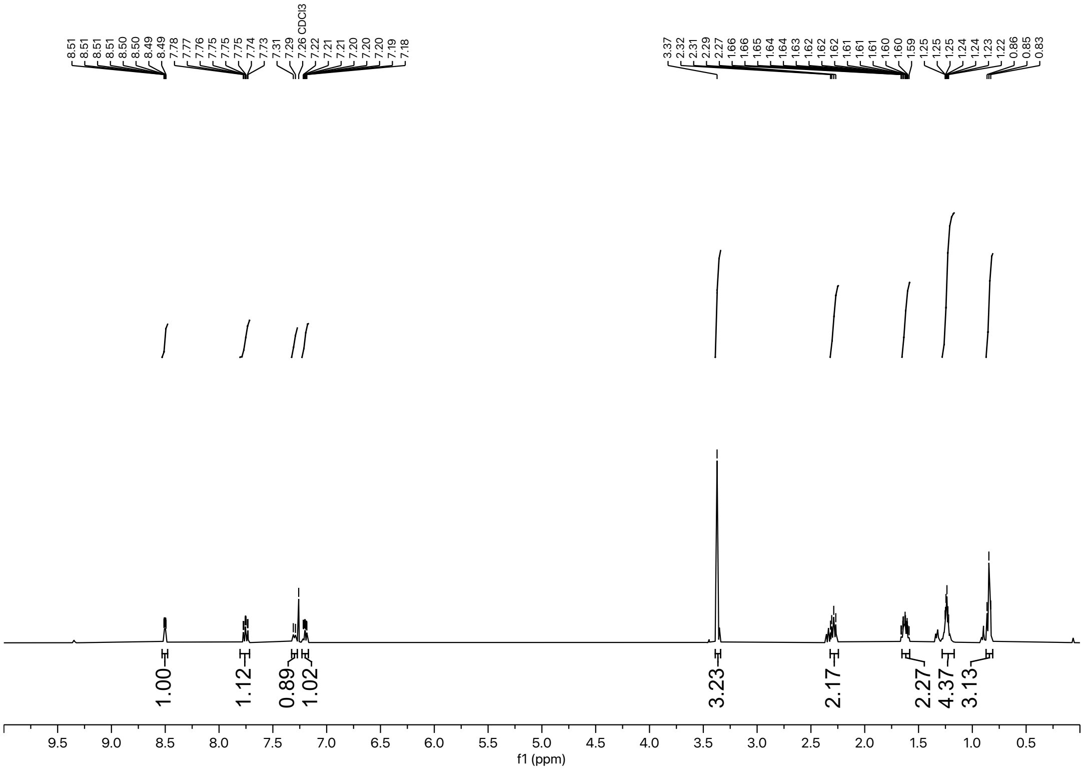

^{13}C NMR (1ag)

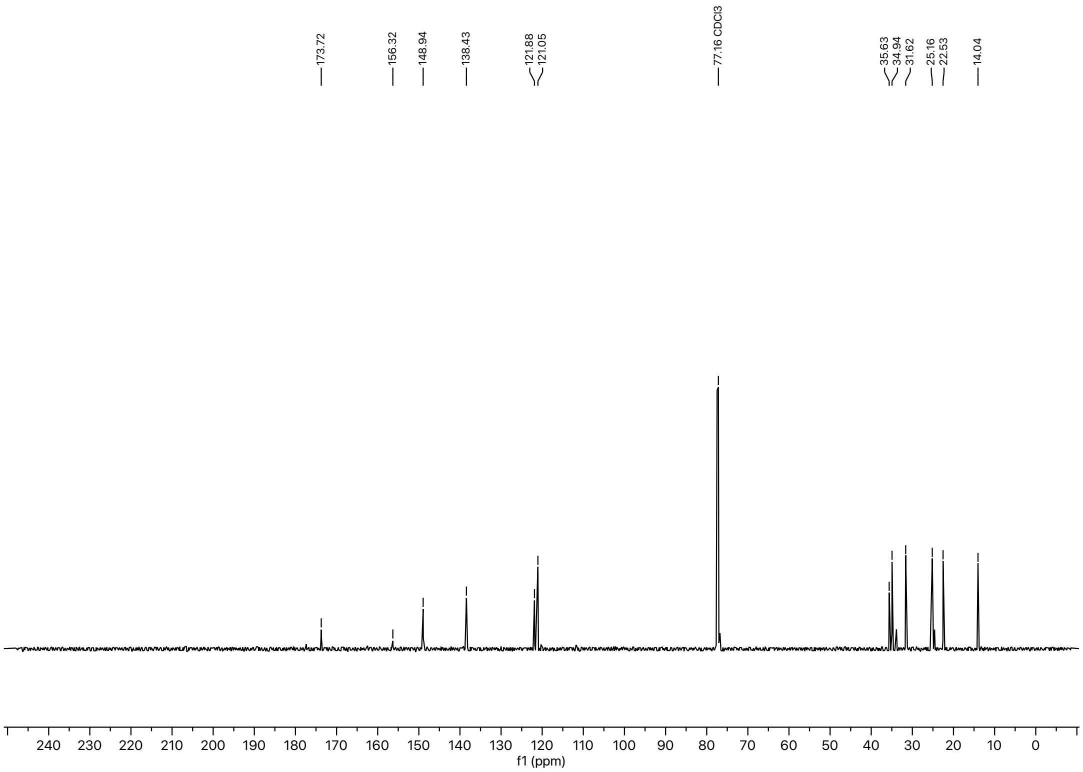

^{13}C DEPT-135 (1ag)

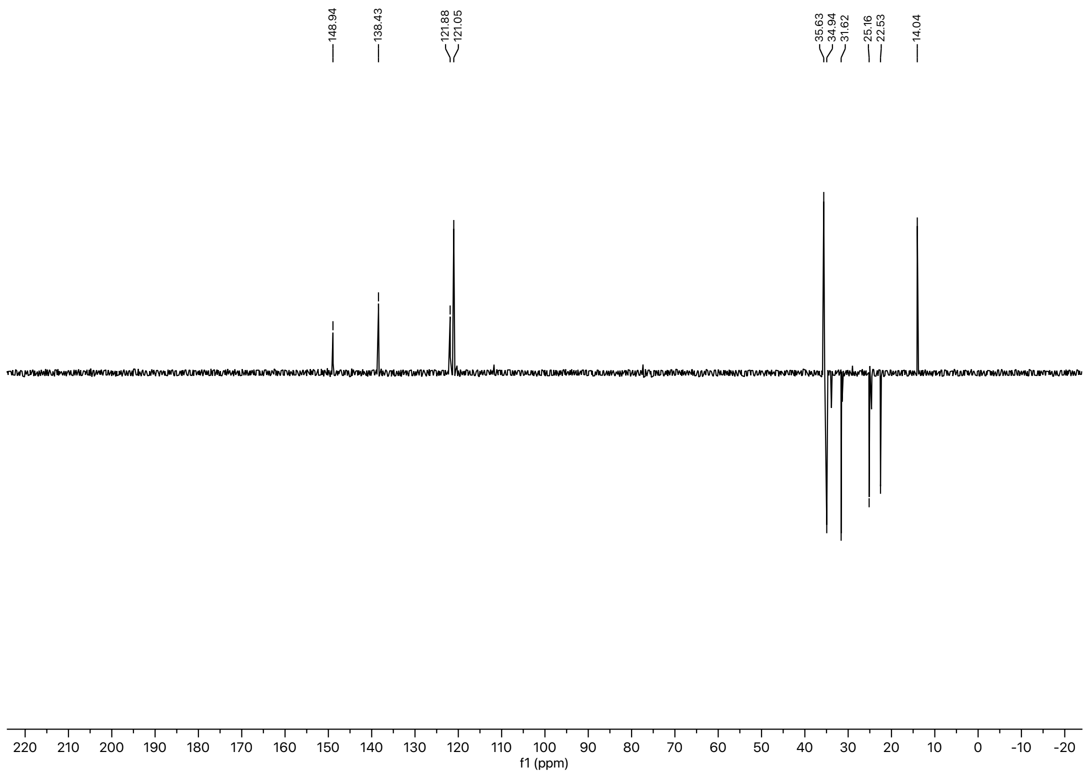

^{1}H NMR (1ah)

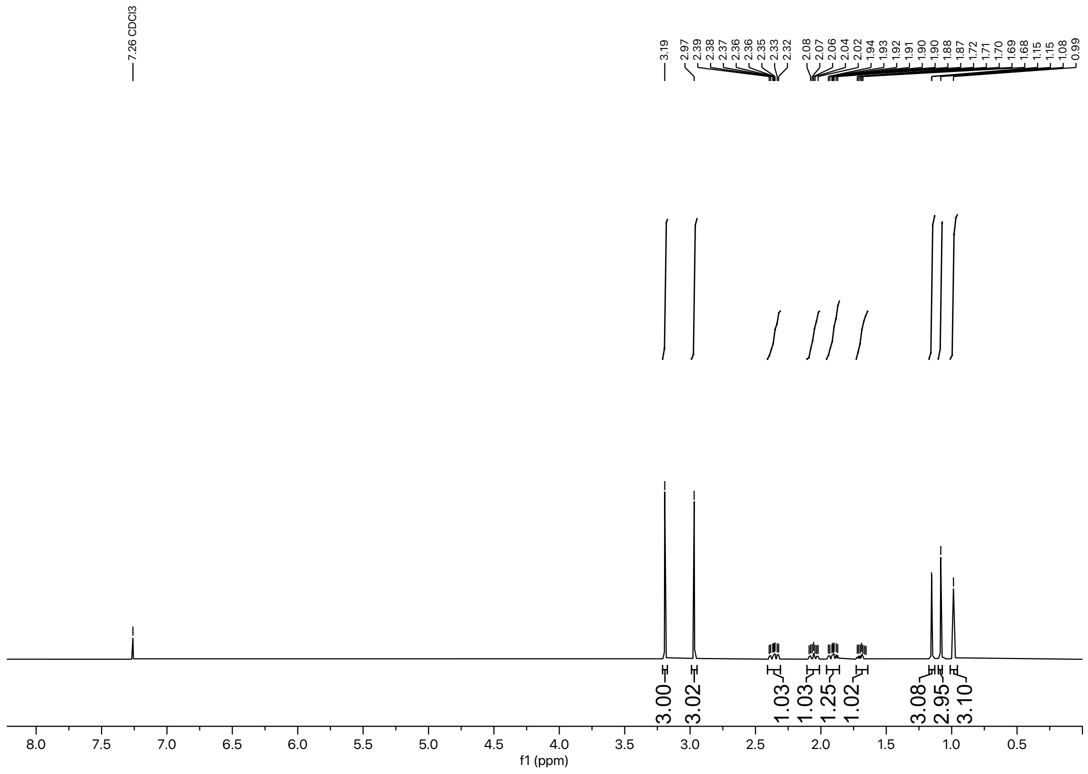

^{13}C NMR (1ah)

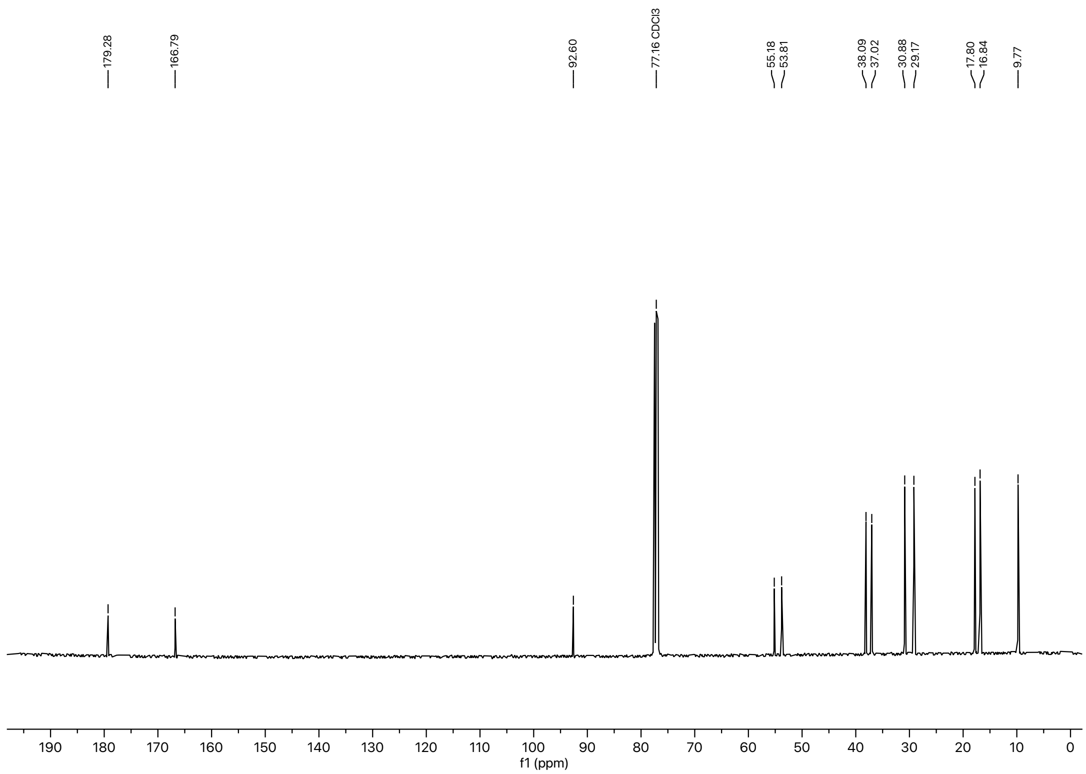

^{13}C DEPT-135 (1ah)

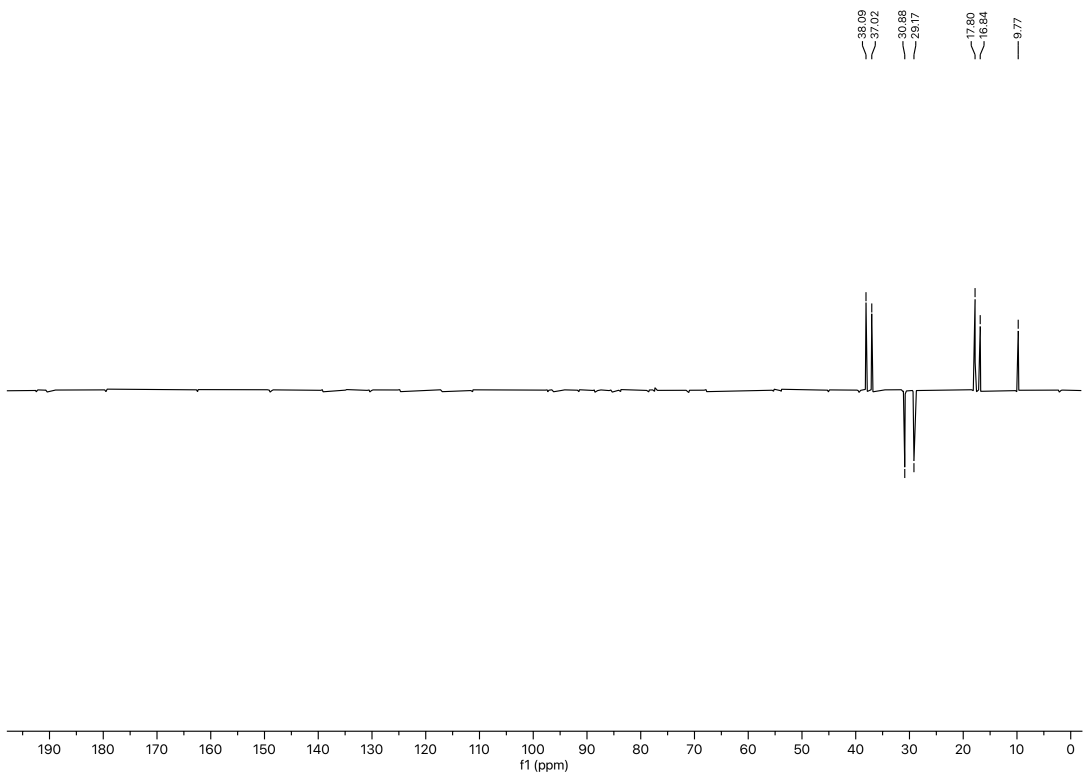

^1H NMR (1ai)

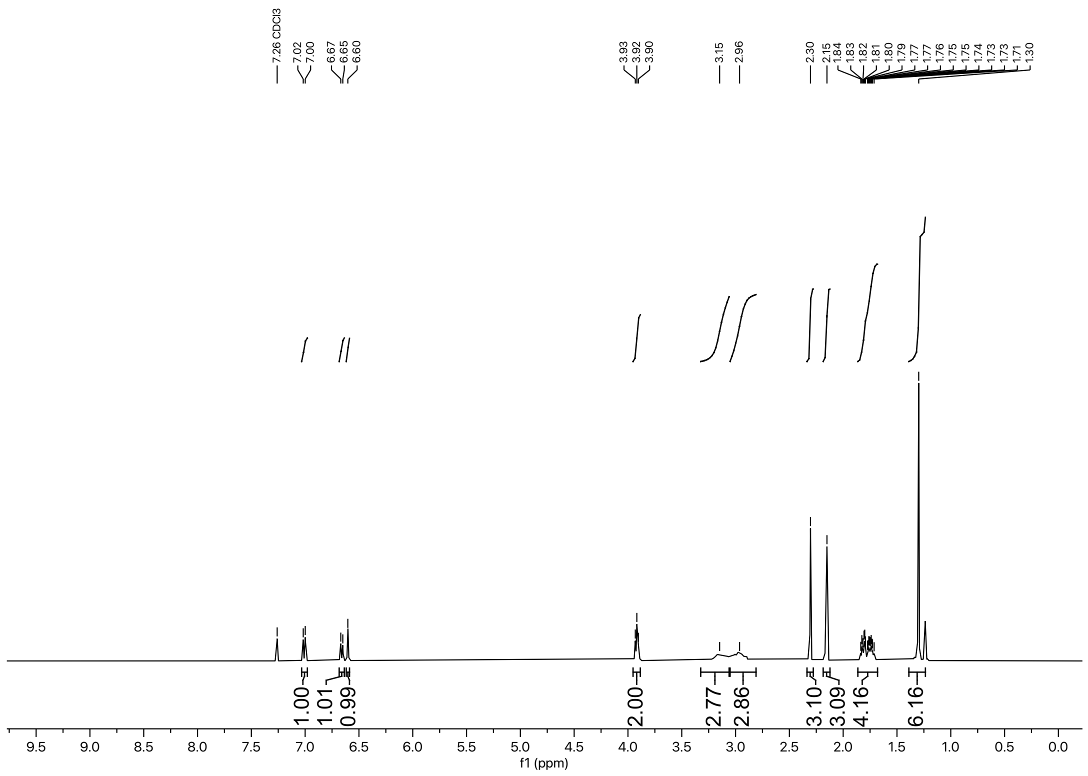

^{13}C NMR (1ai)

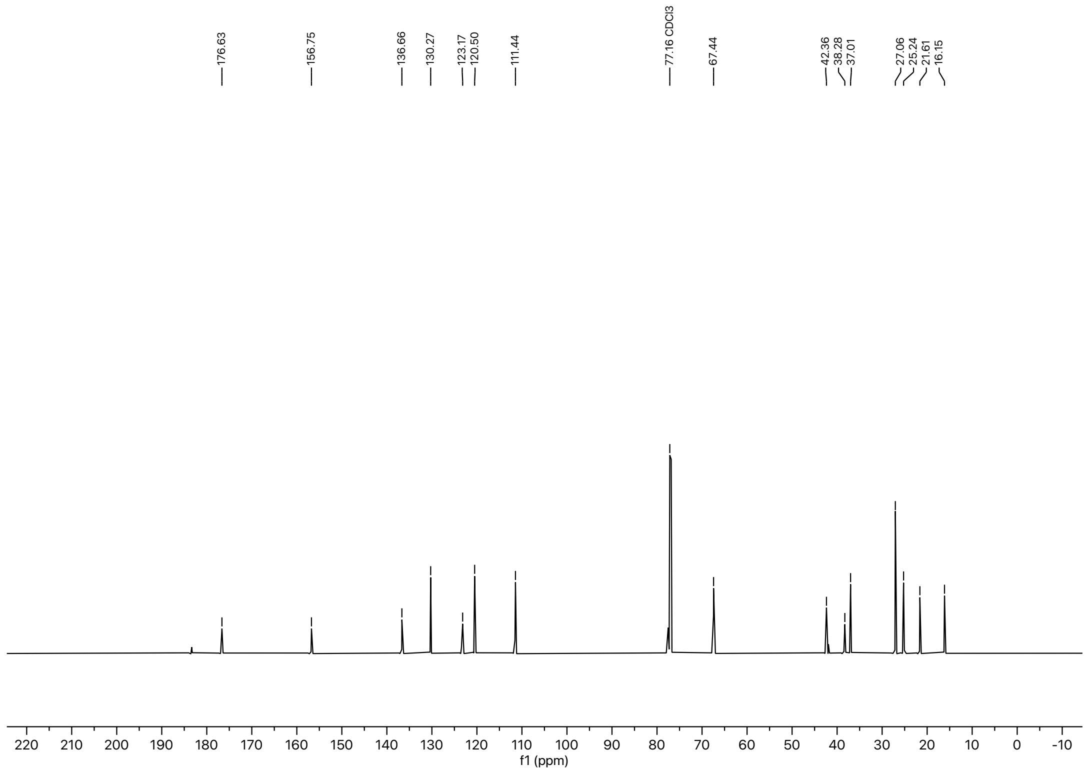

^{13}C DEPT-135 (1ai)

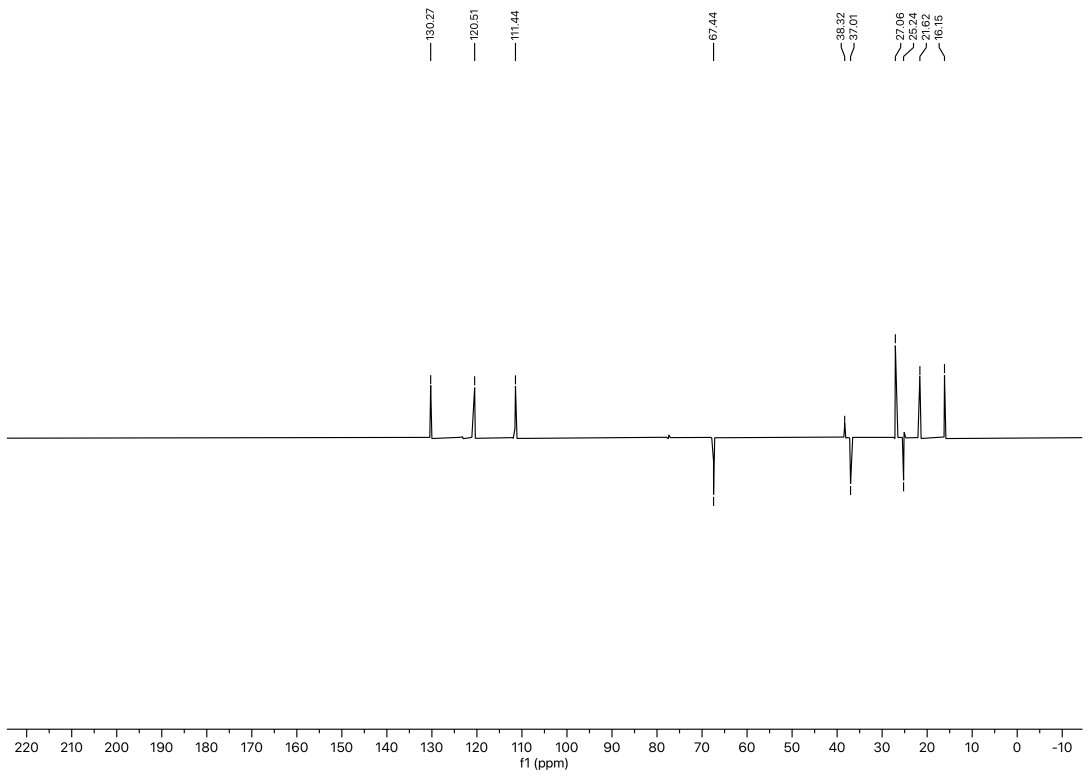

^{1}H NMR (1aj)

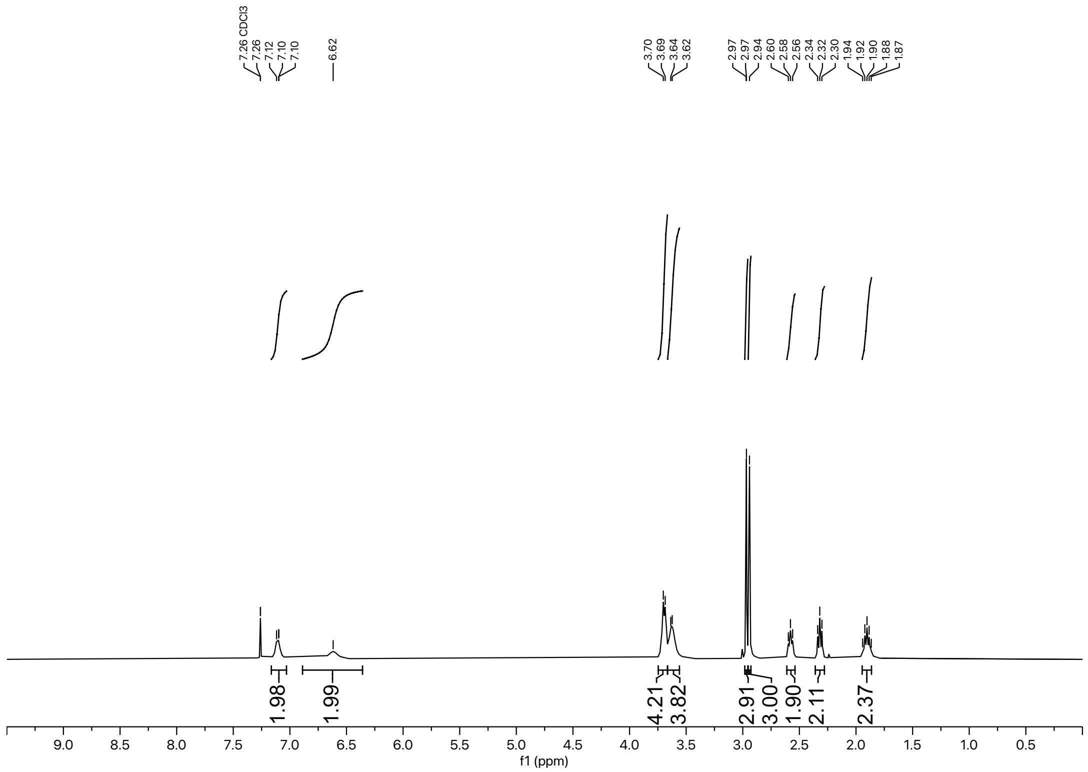

^{13}C NMR (1aj)

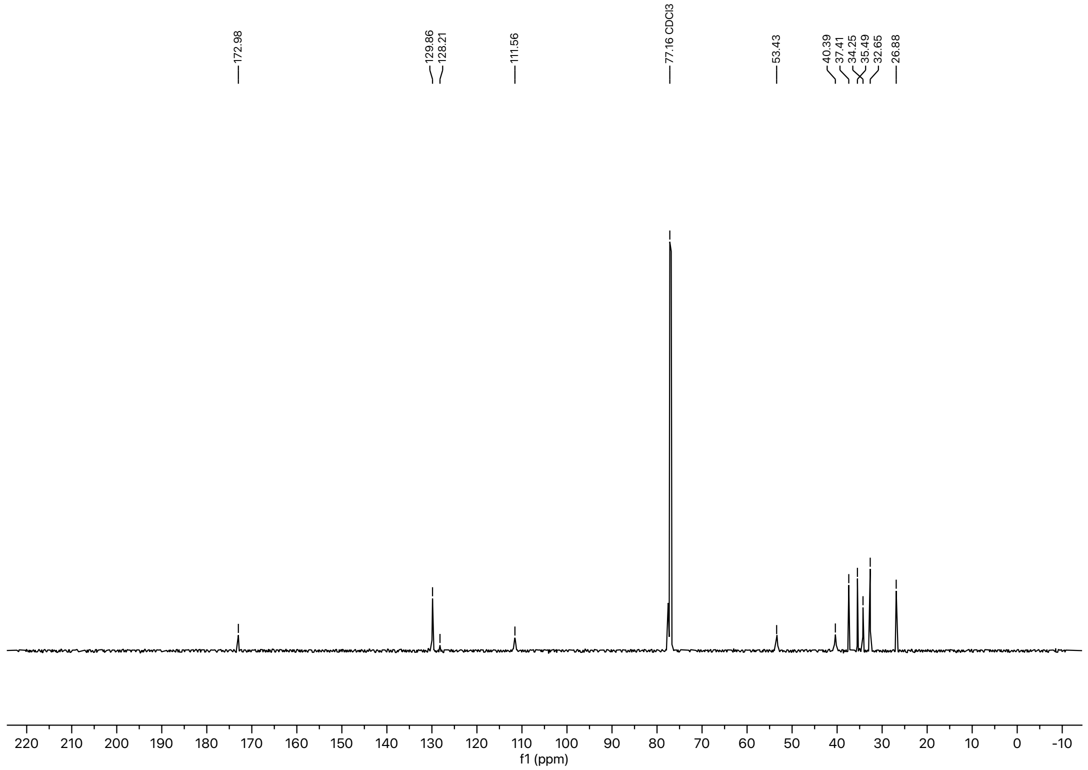

^{13}C DEPT-135 (1aj)

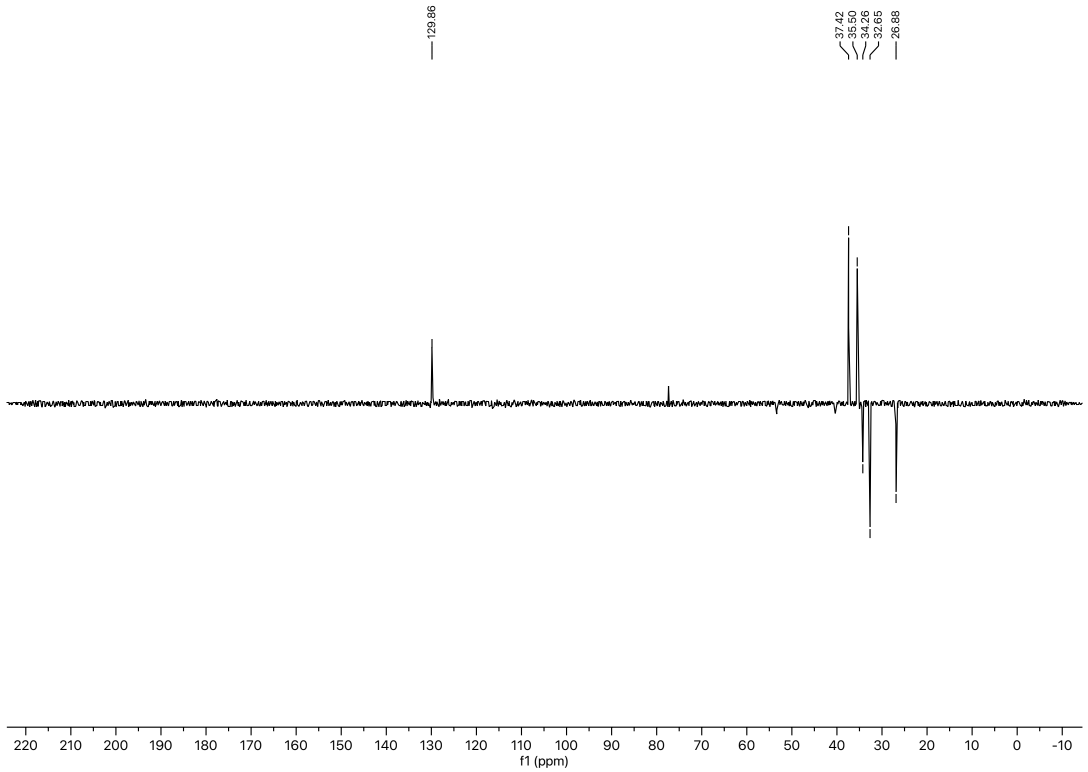

^{1}H NMR (1ak)

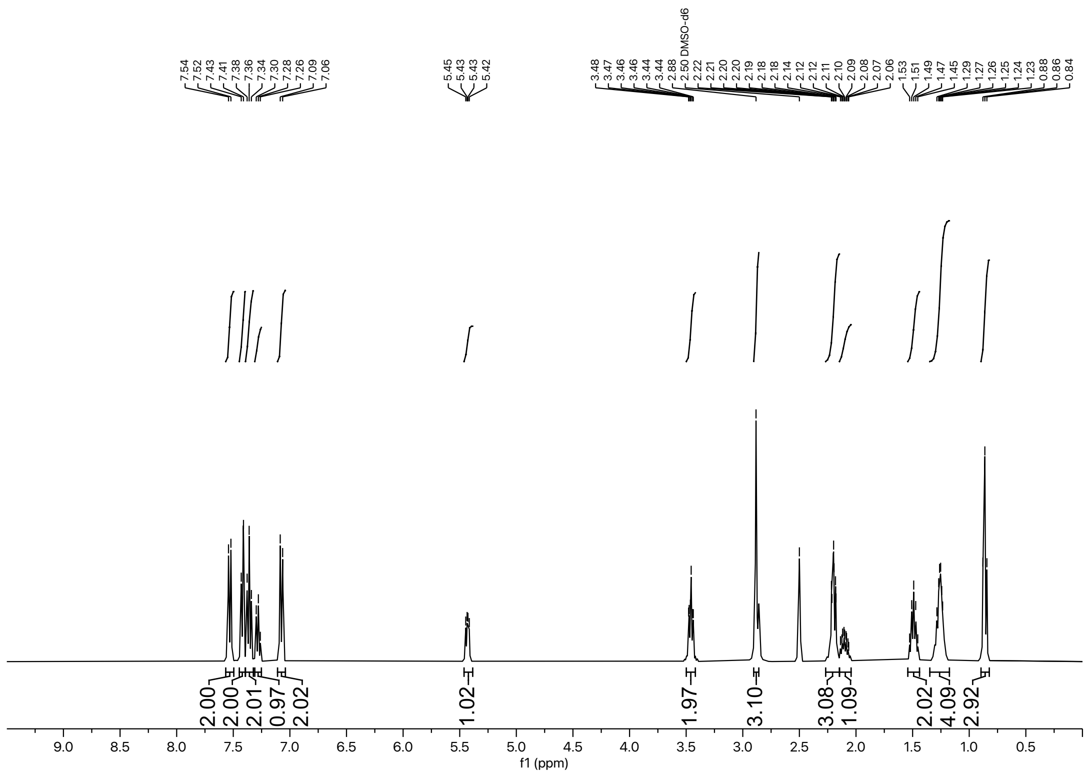

^{13}C NMR (1ak)

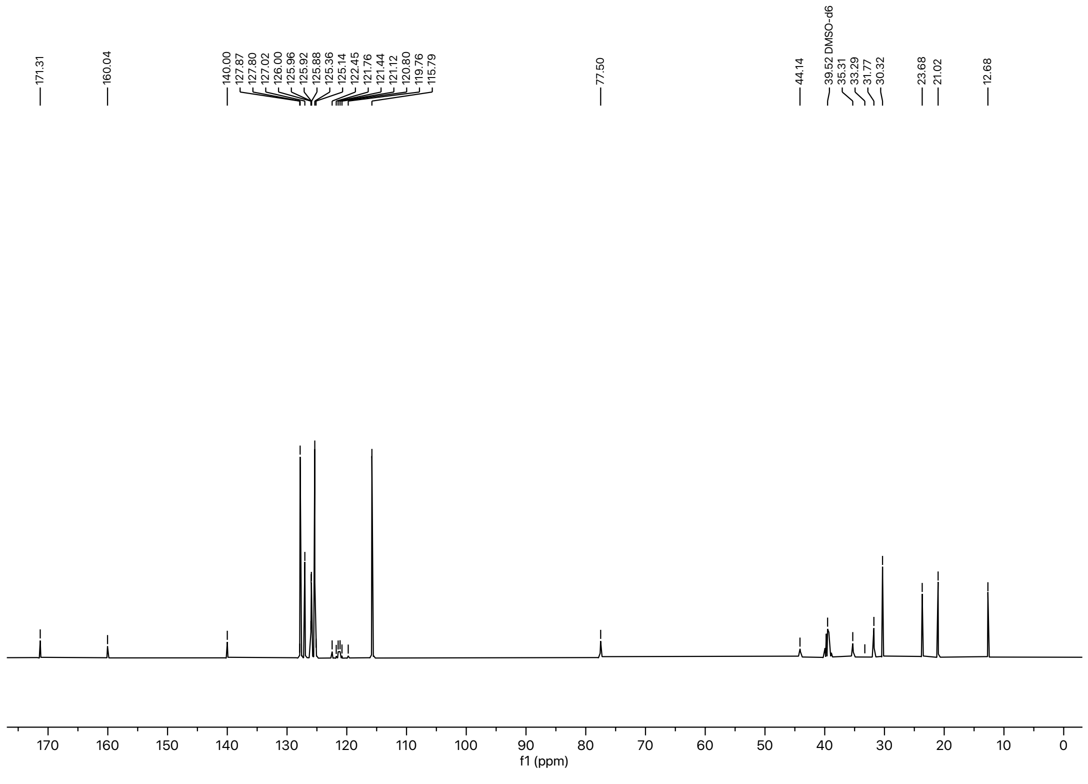

^{13}C DEPT-135 (1ak)

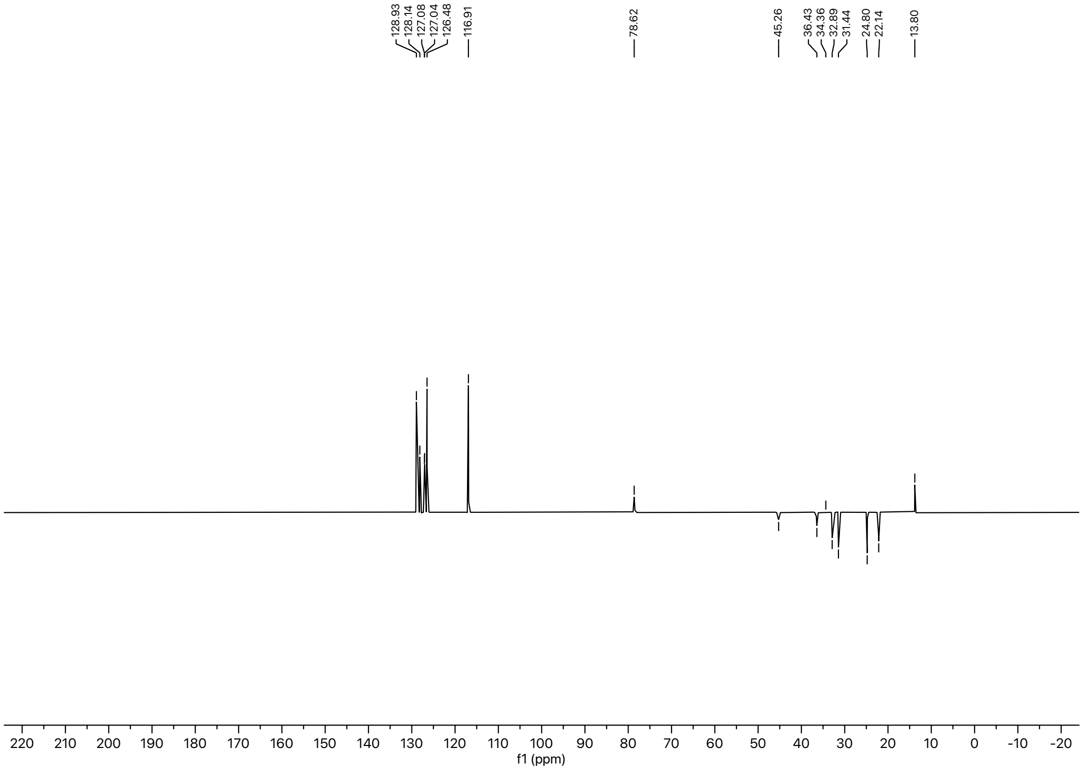

^{19}F NMR (1ak)

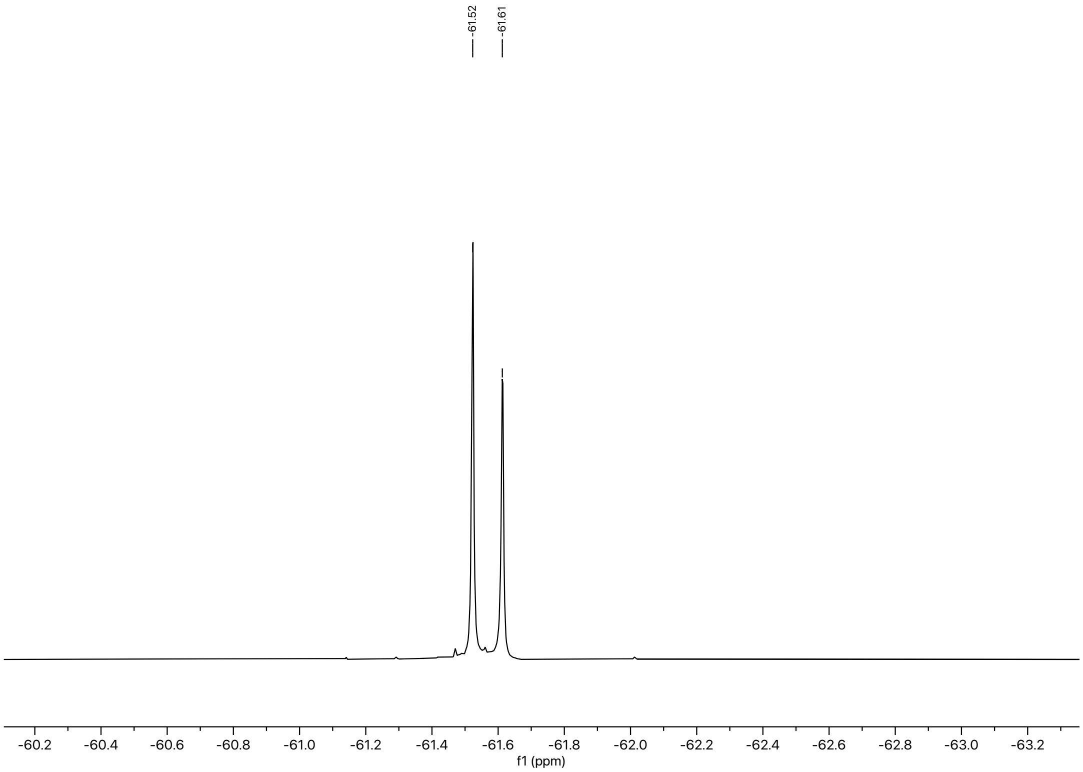

^{1}H NMR (L2)

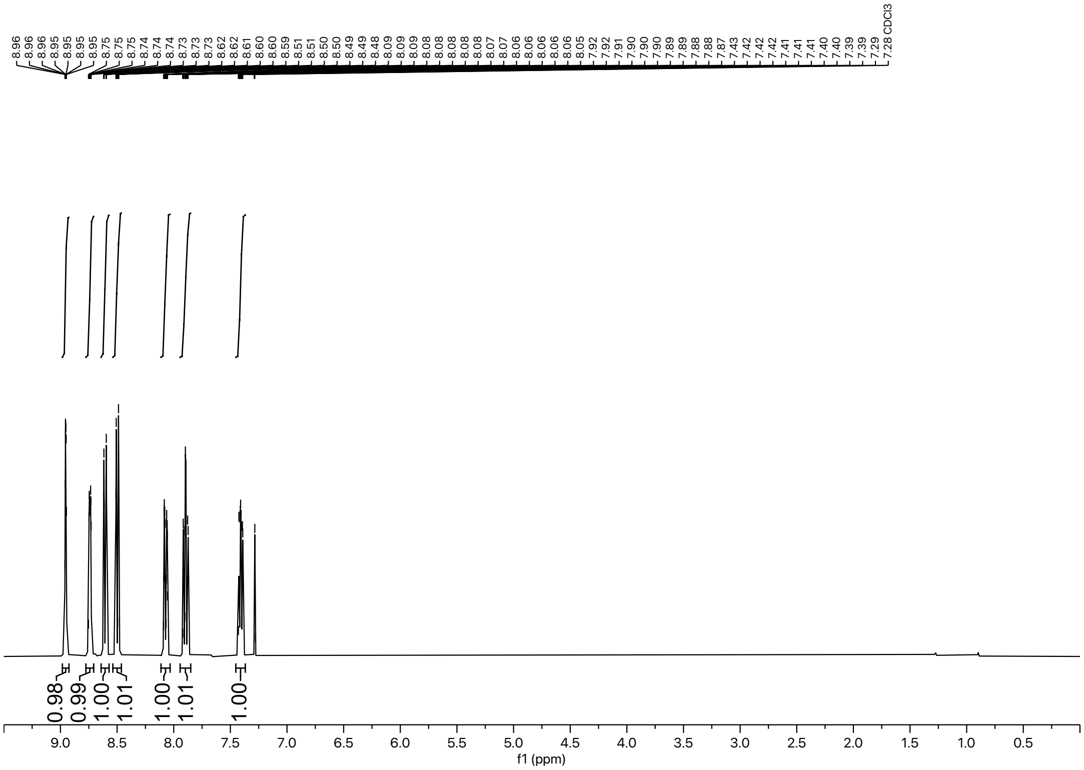

^{13}C NMR (L2)

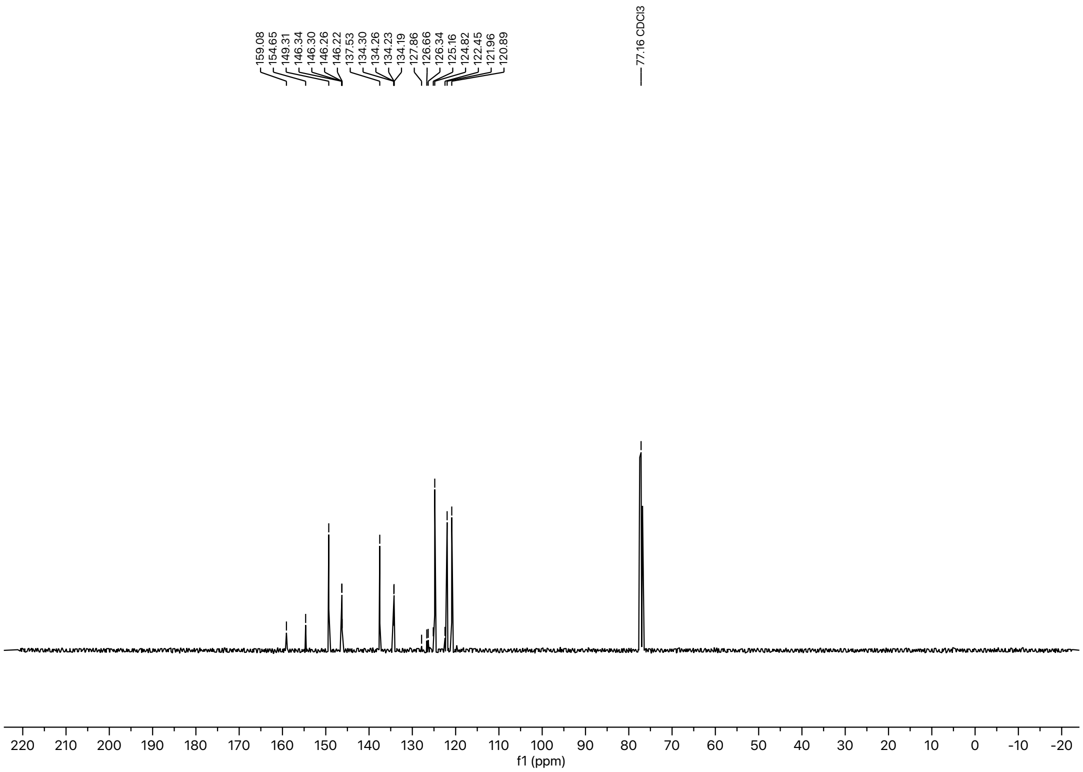

^{13}C DEPT-135 (L2)

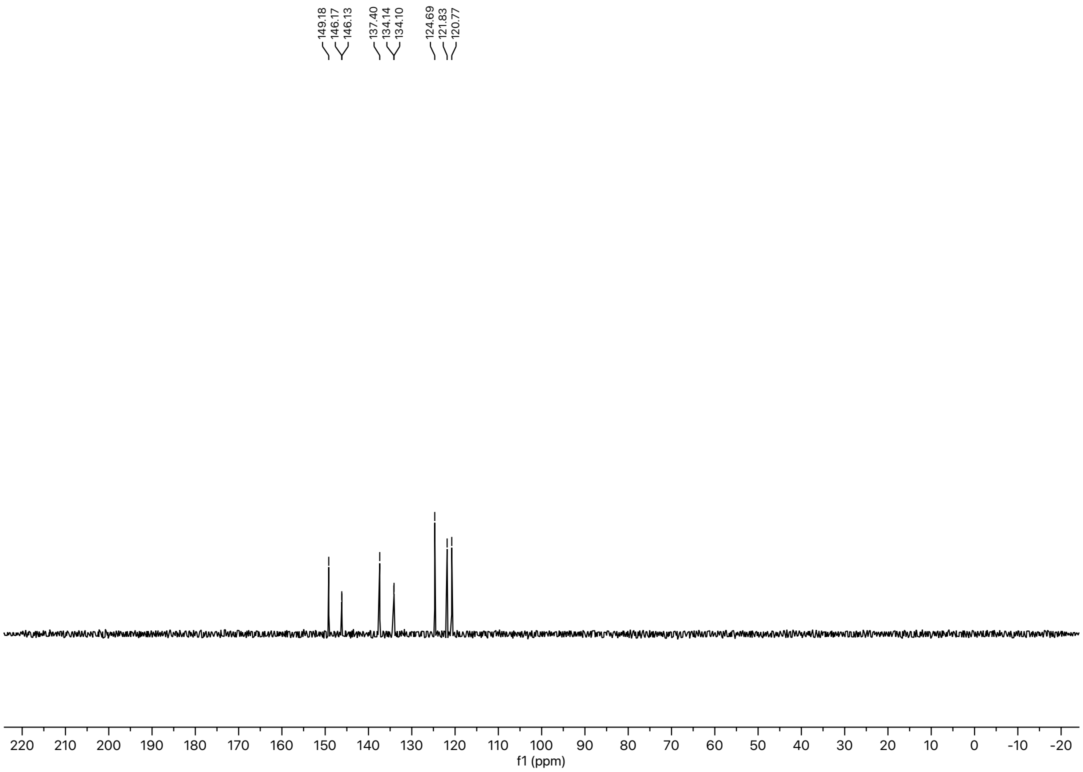

^{19}F NMR (L2)

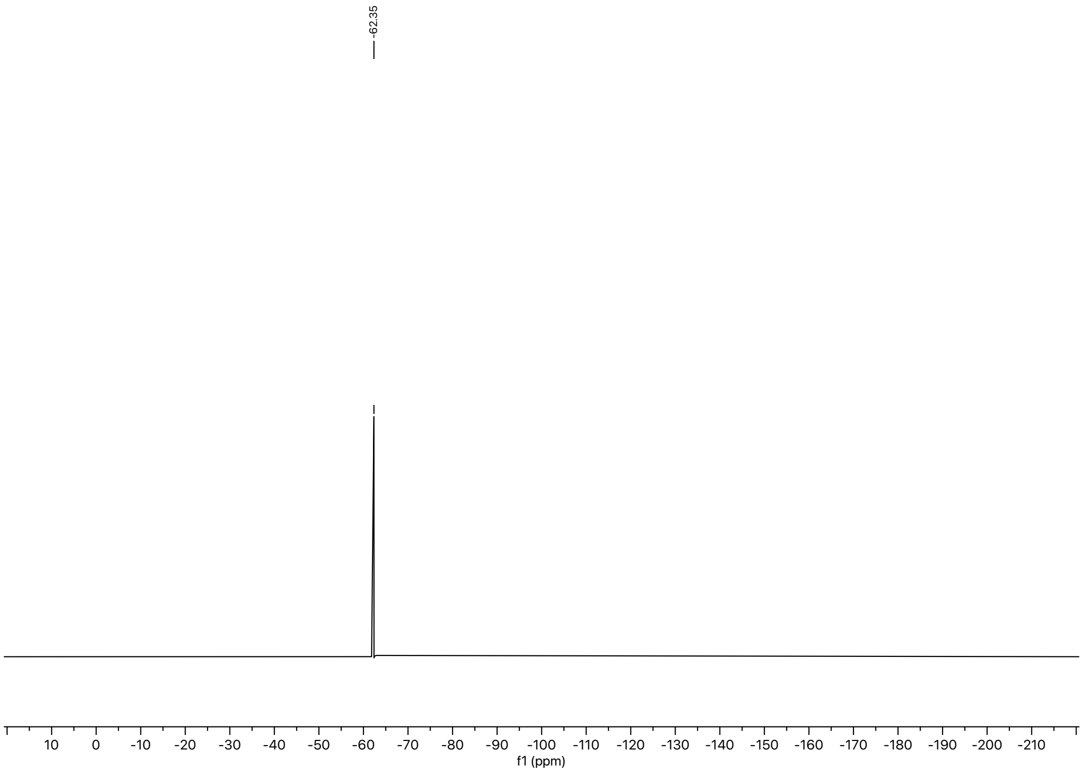

^{1}H NMR (L3)

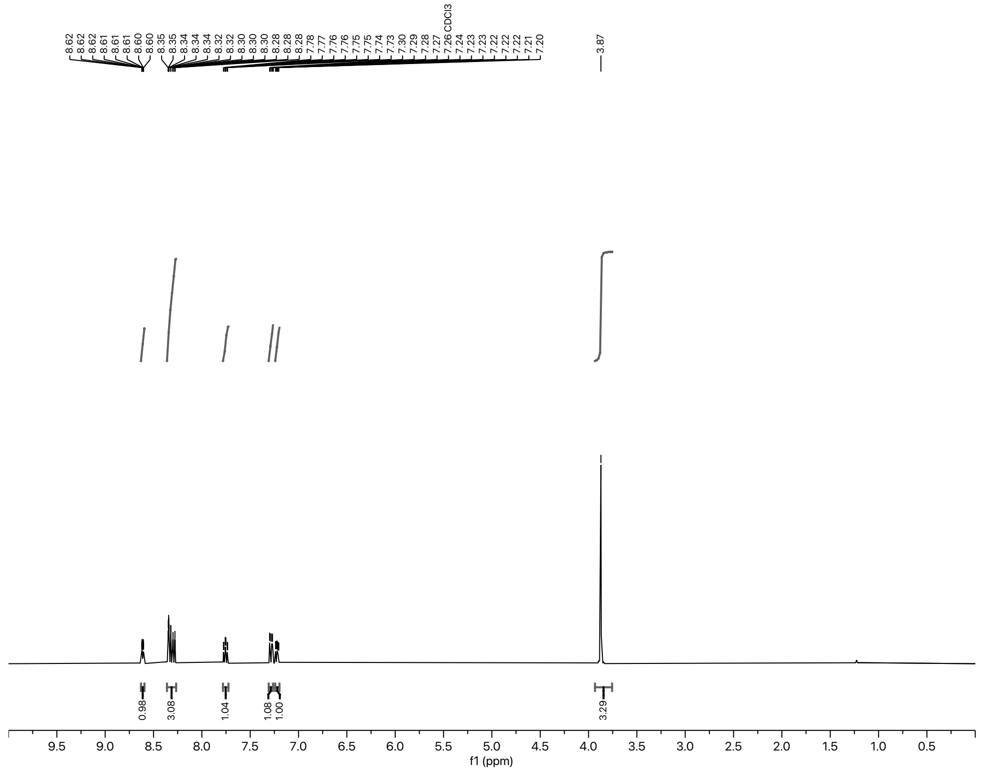

^{13}C NMR (L3)

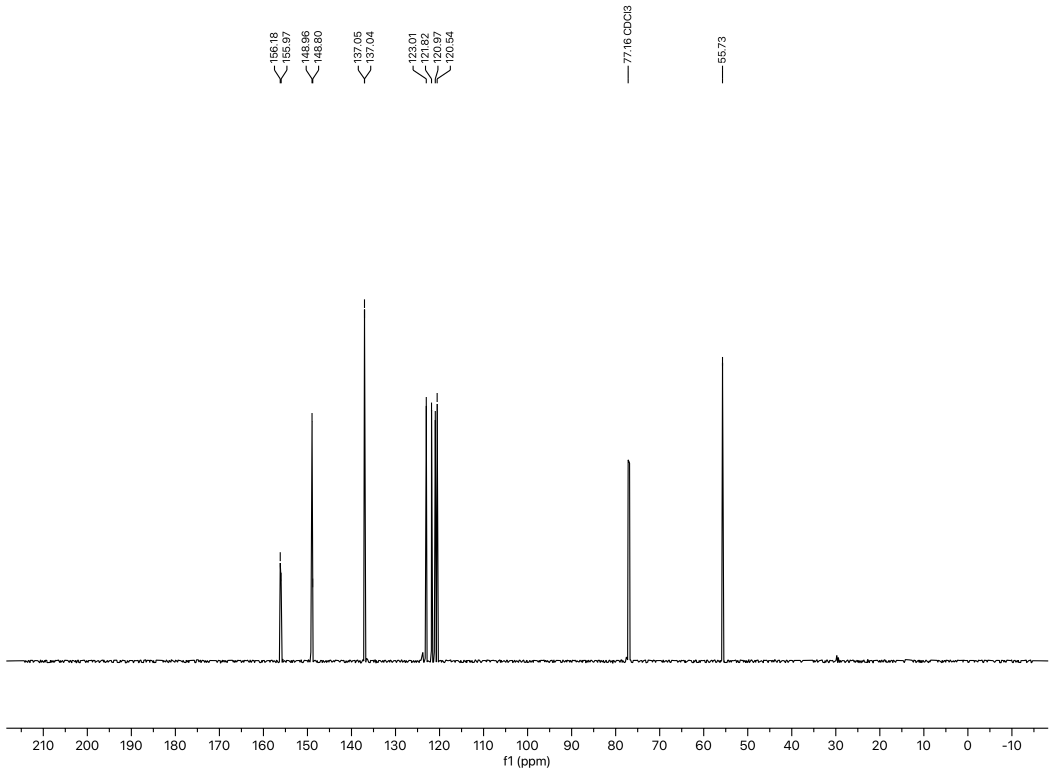

^{13}C DEPT-135 (L3)

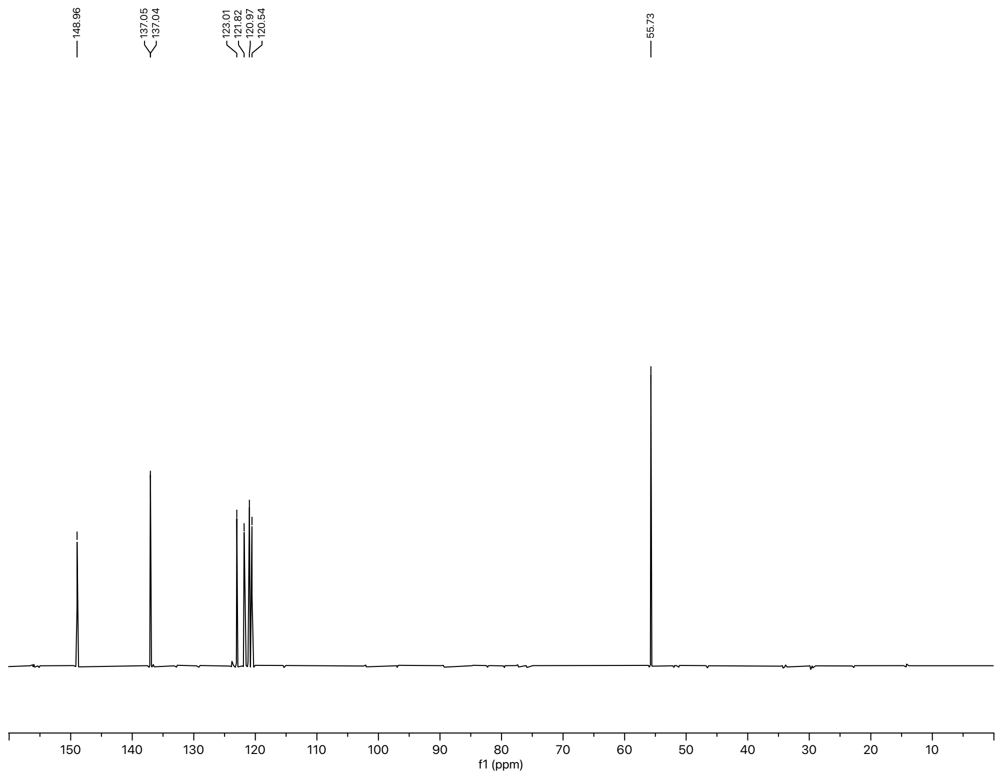

^{1}H NMR (L4)

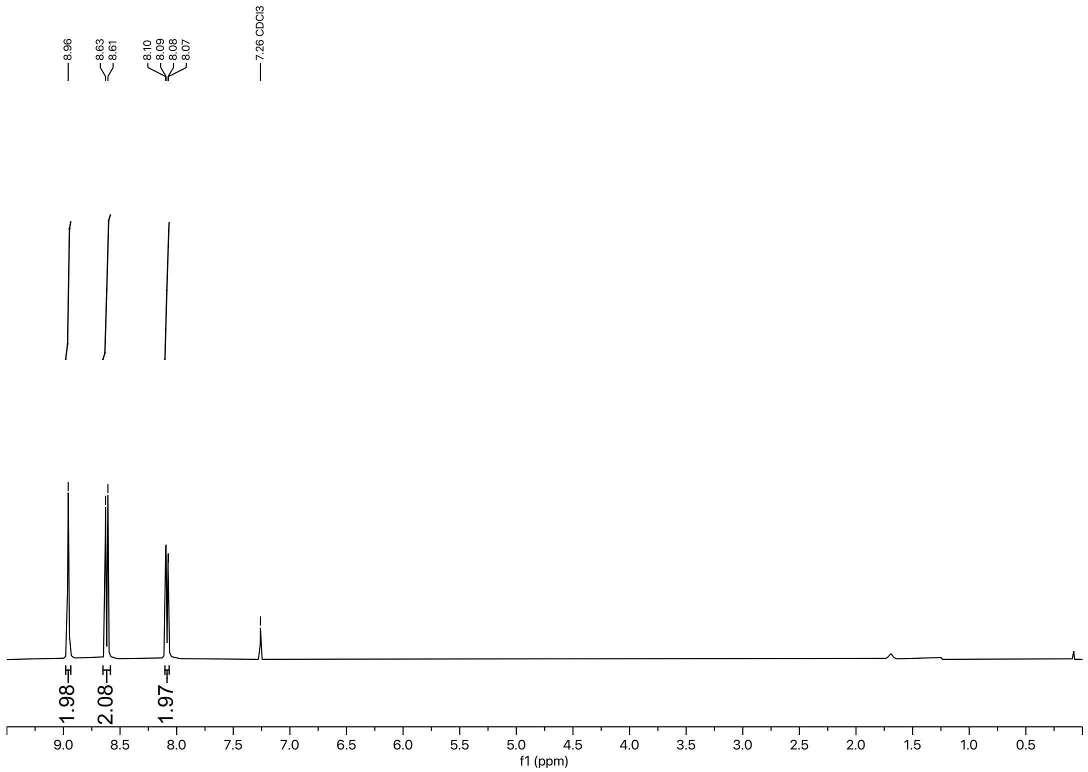

^{13}C NMR (L4)

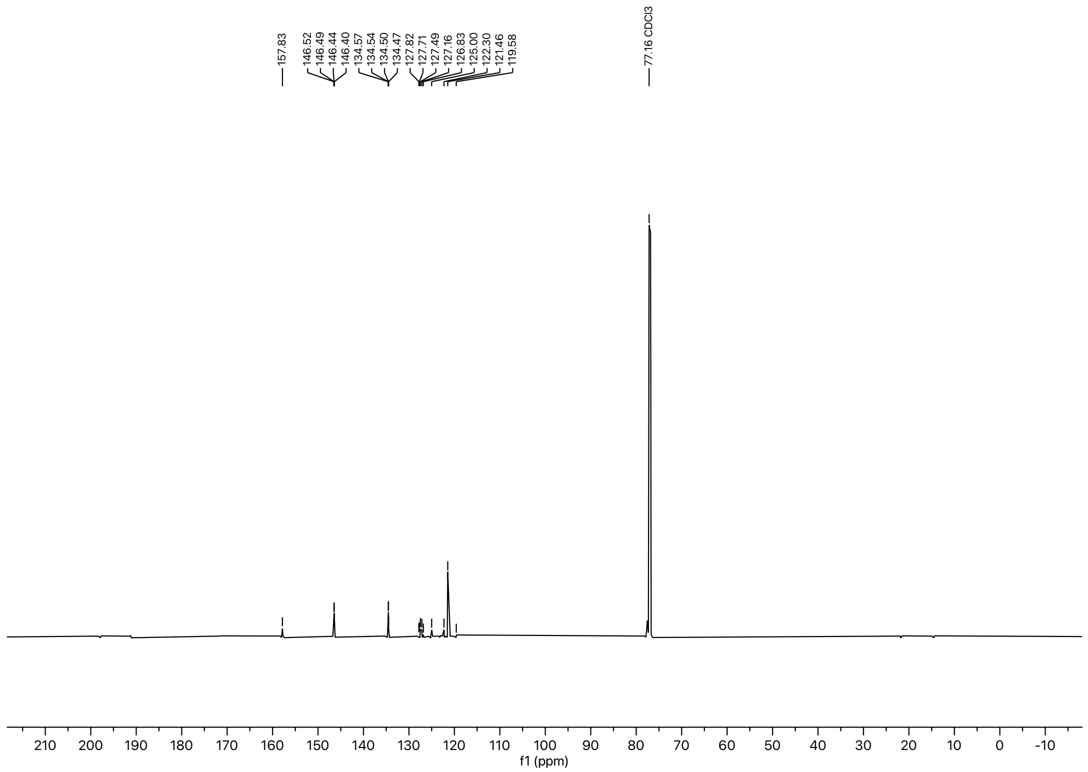

^{13}C DEPT-135 (L4)

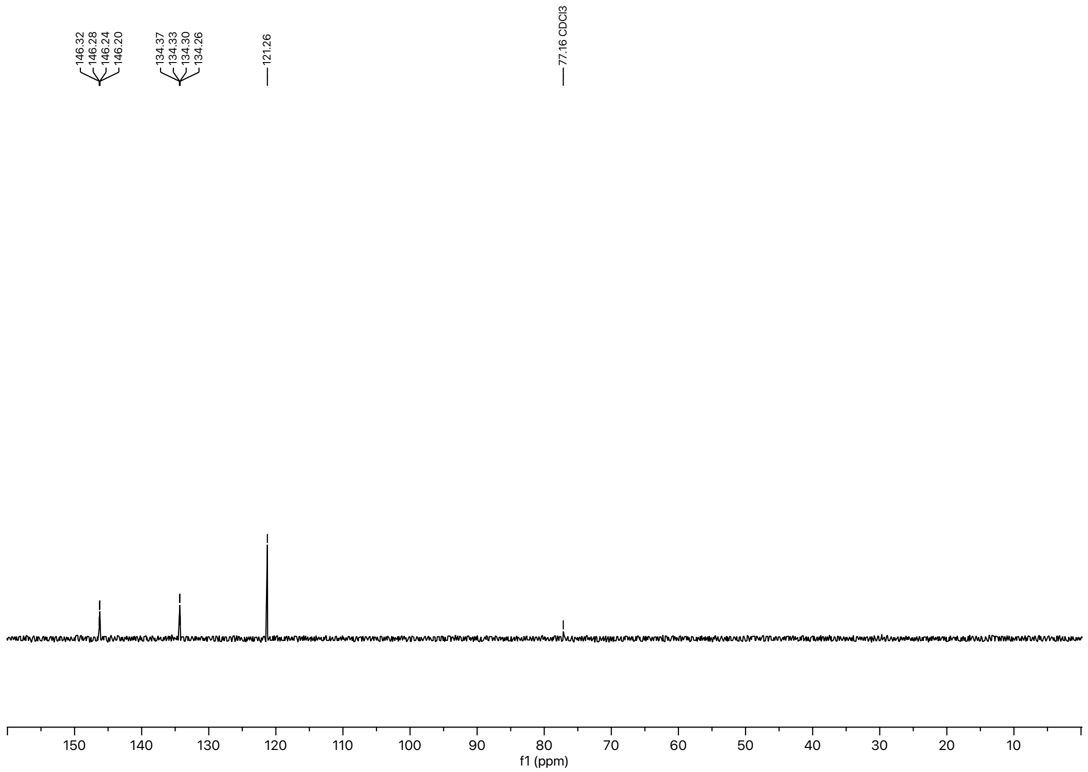

^{19}F NMR (L4)

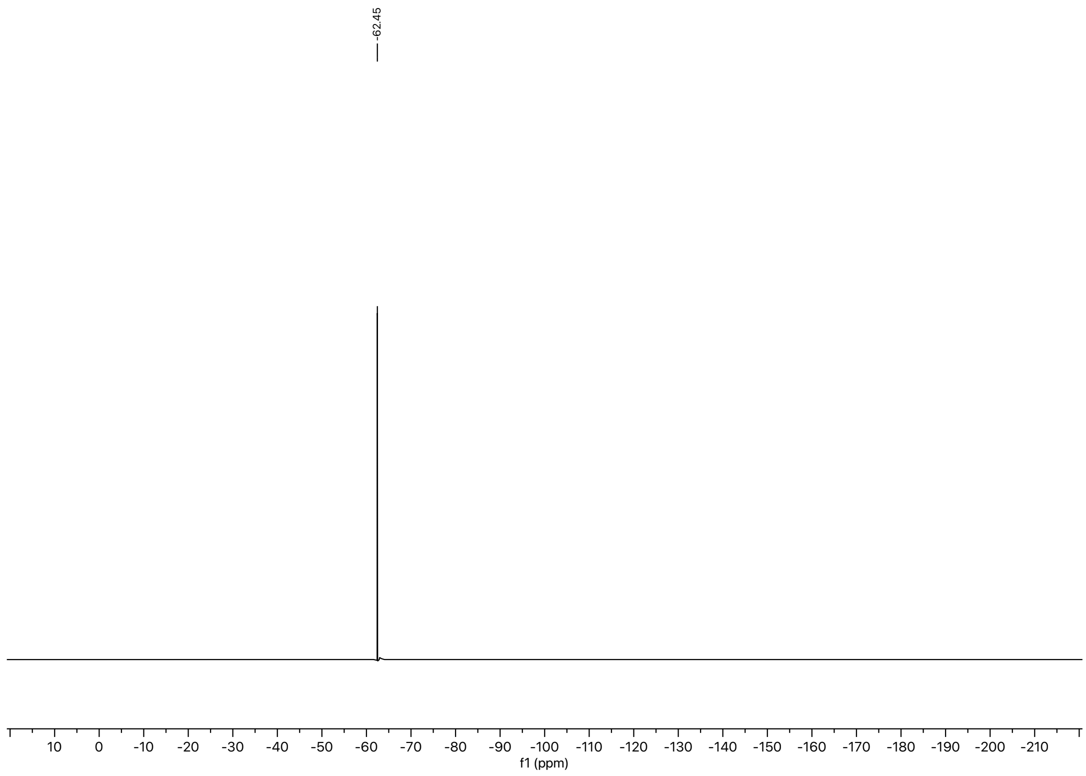

^{1}H NMR (L6)

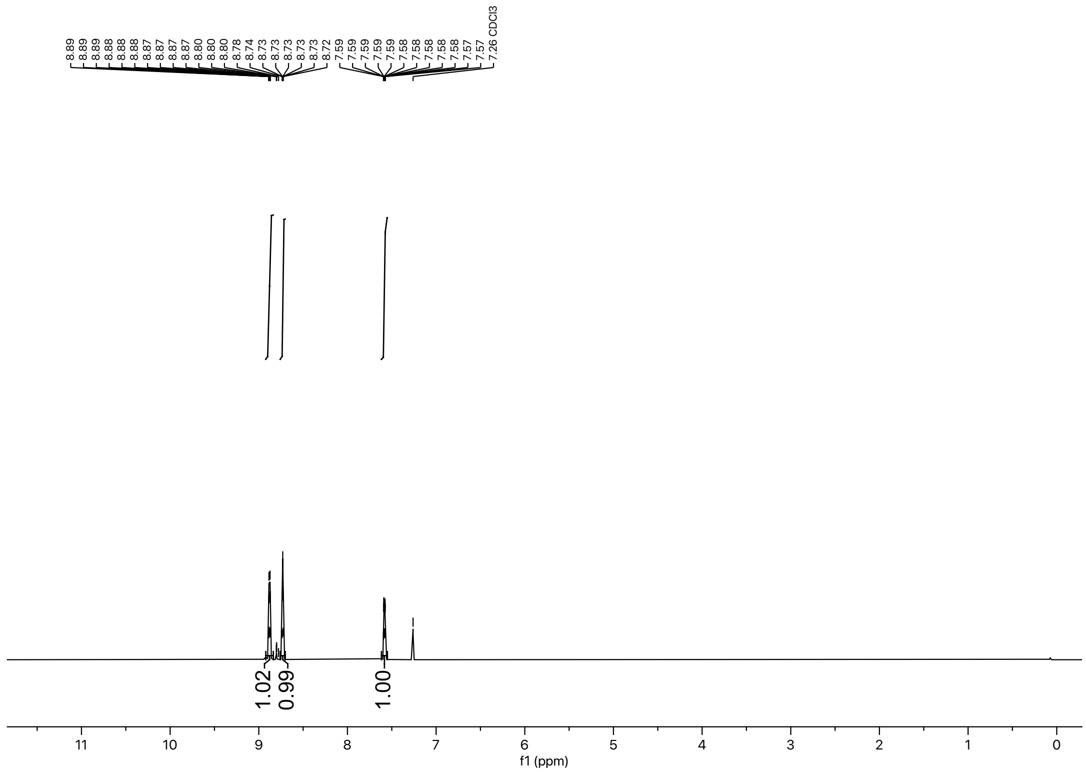

^{13}C NMR (L6)

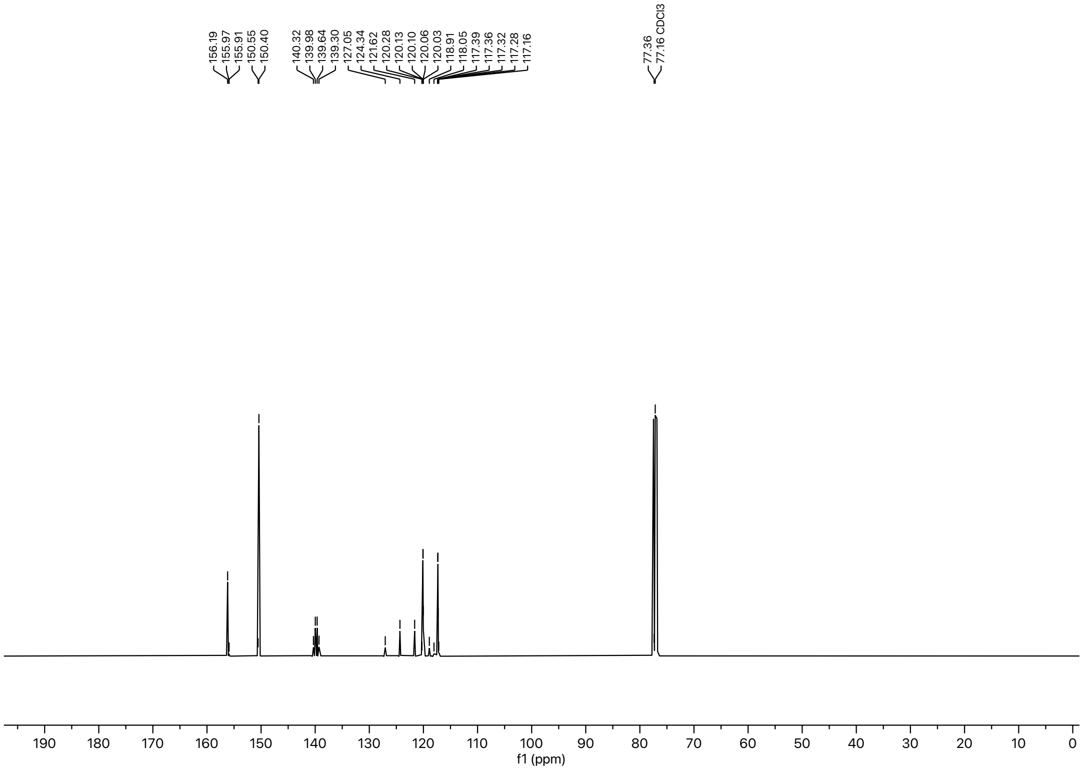

^{13}C DEPT-135 (L6)

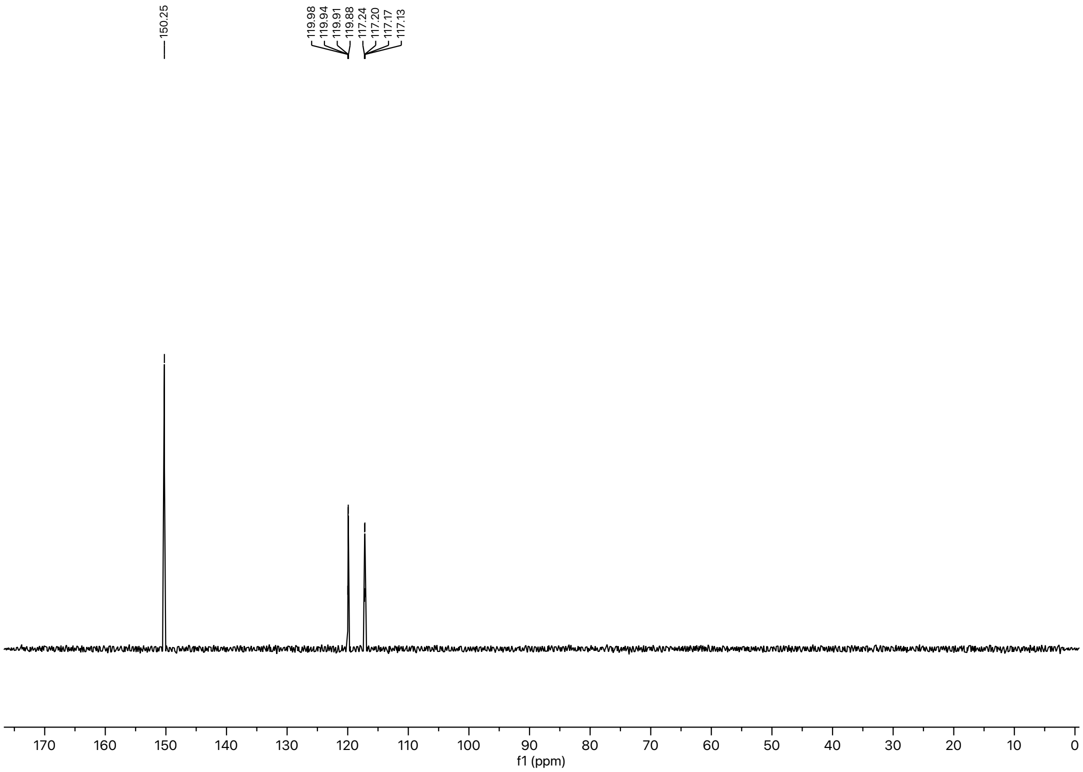

^{19}F NMR (L6)

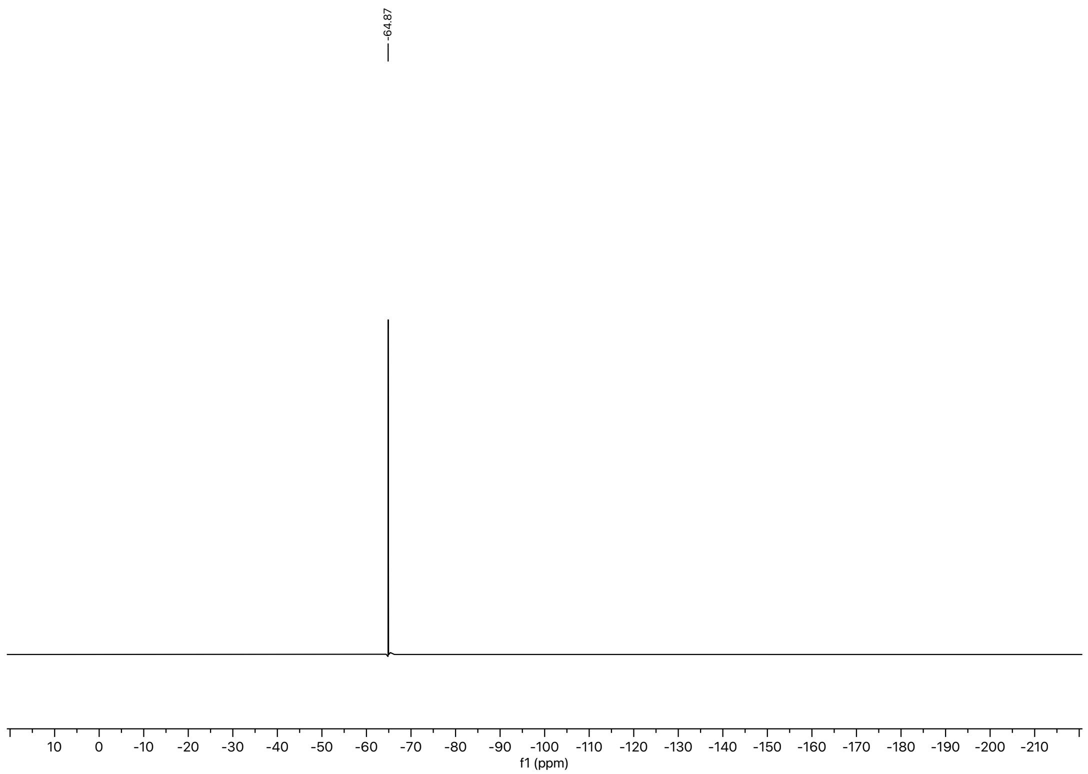

^{1}H NMR (L7)

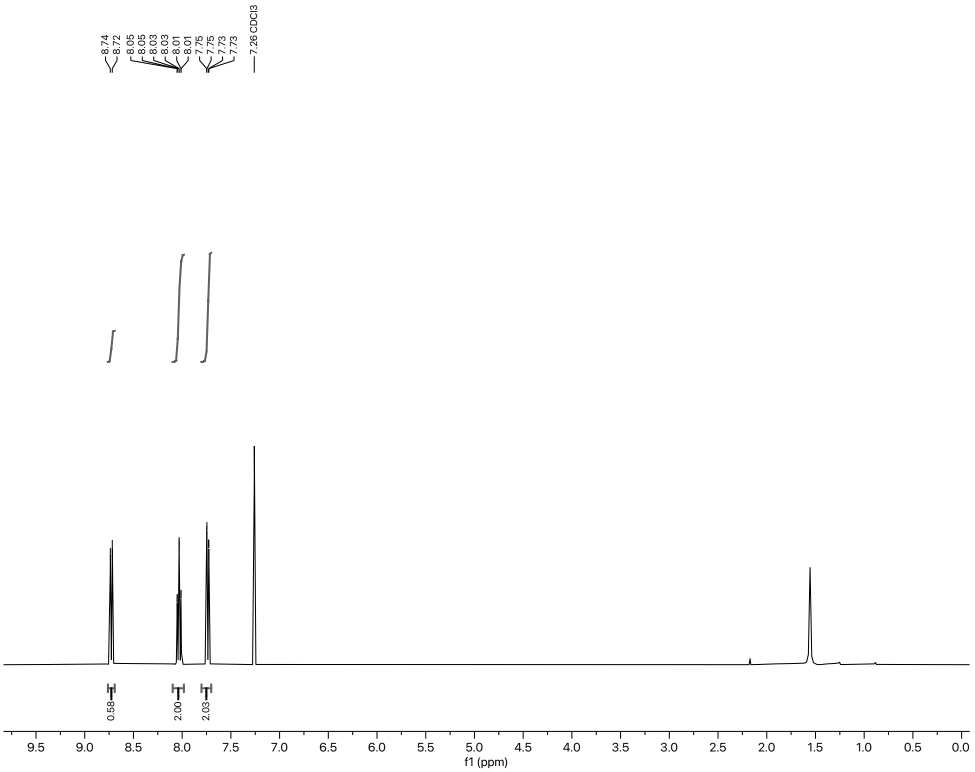

^{13}C NMR (L7)

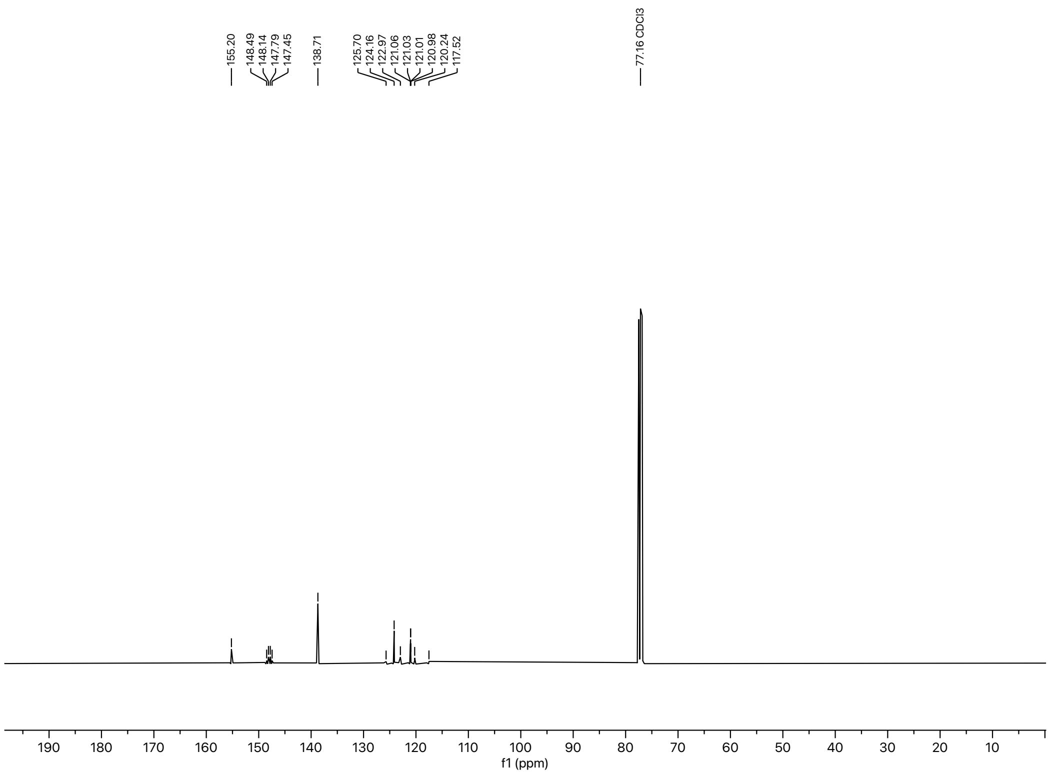

^{13}C DEPT-135 (L7)

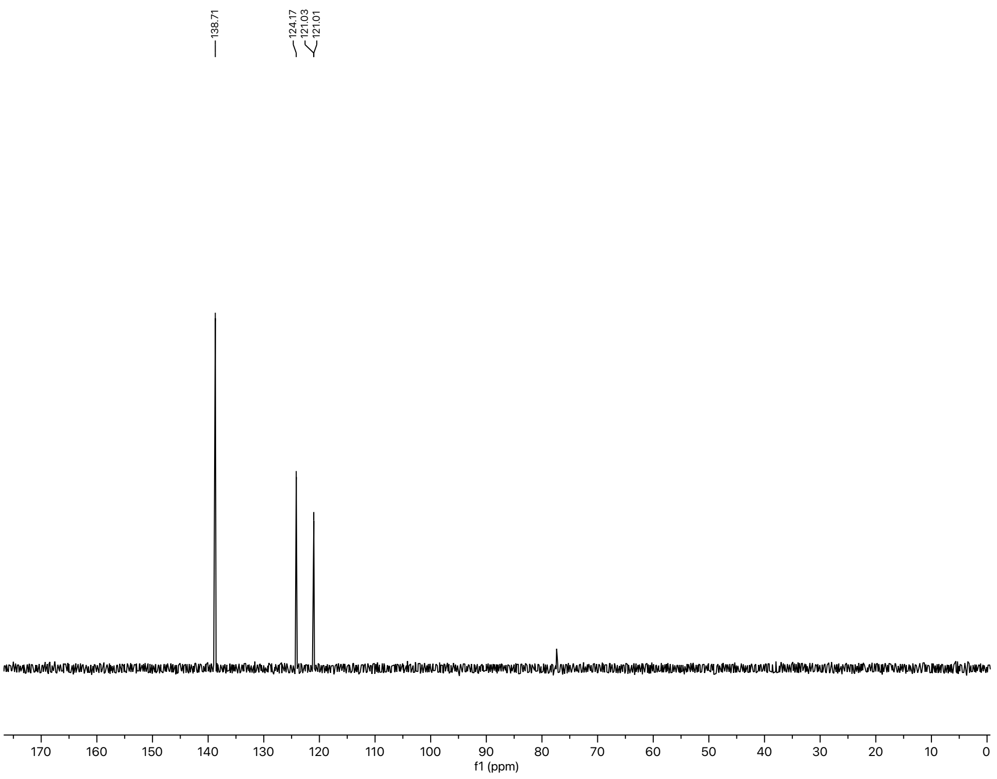

^{19}F NMR (L7)

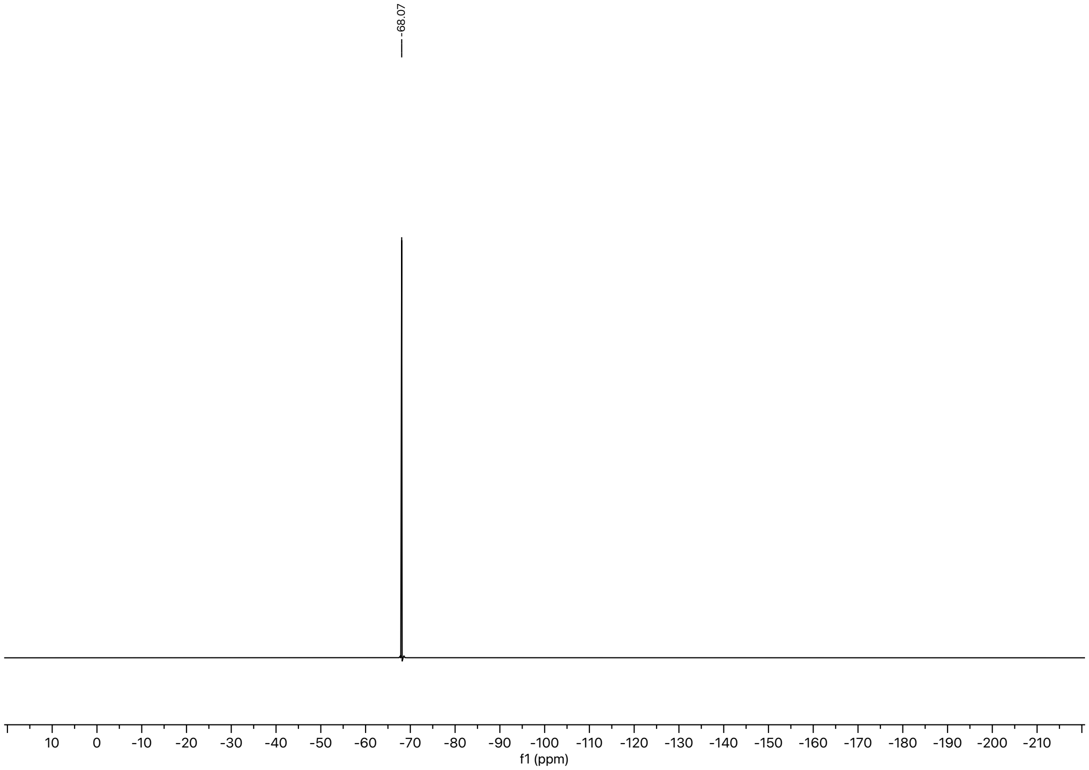

^{1}H NMR (2a)

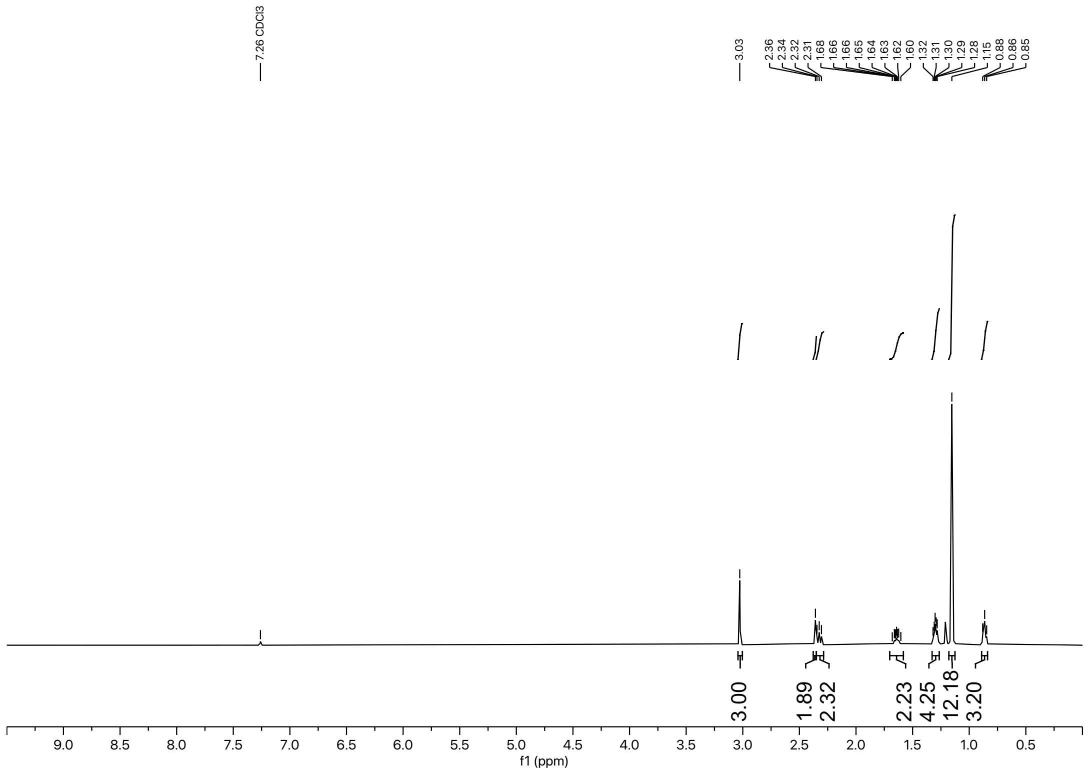

^{13}C NMR (2a)

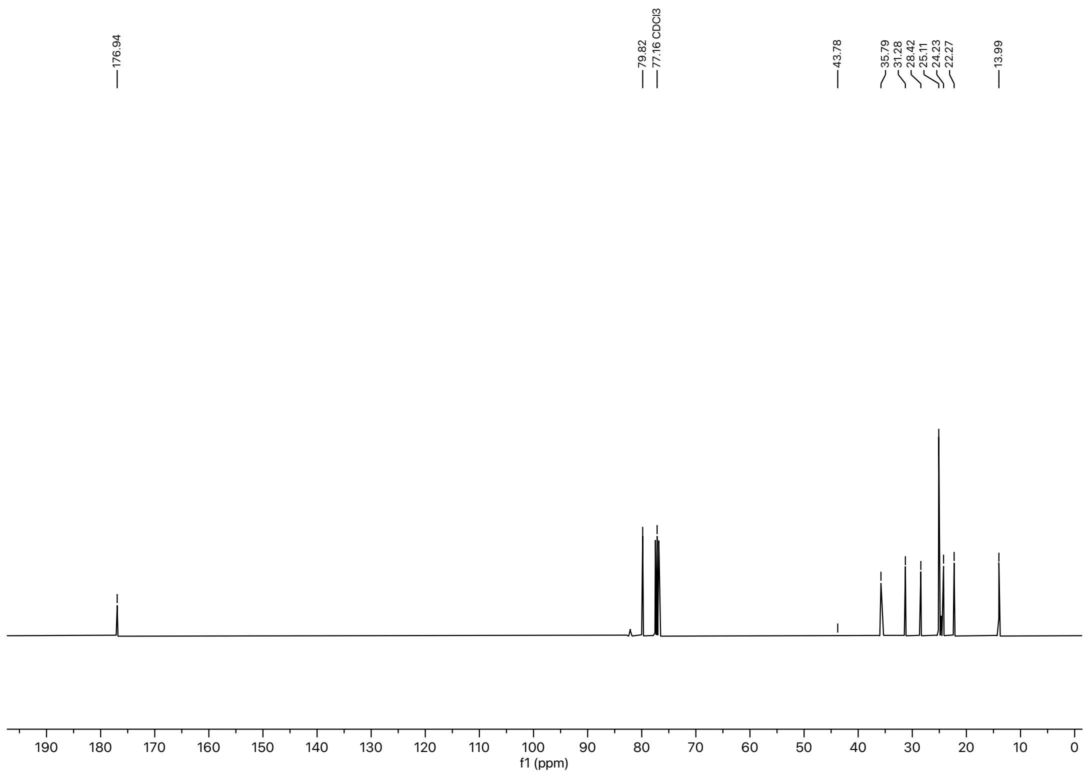

^{13}C DEPT-135 (2a)

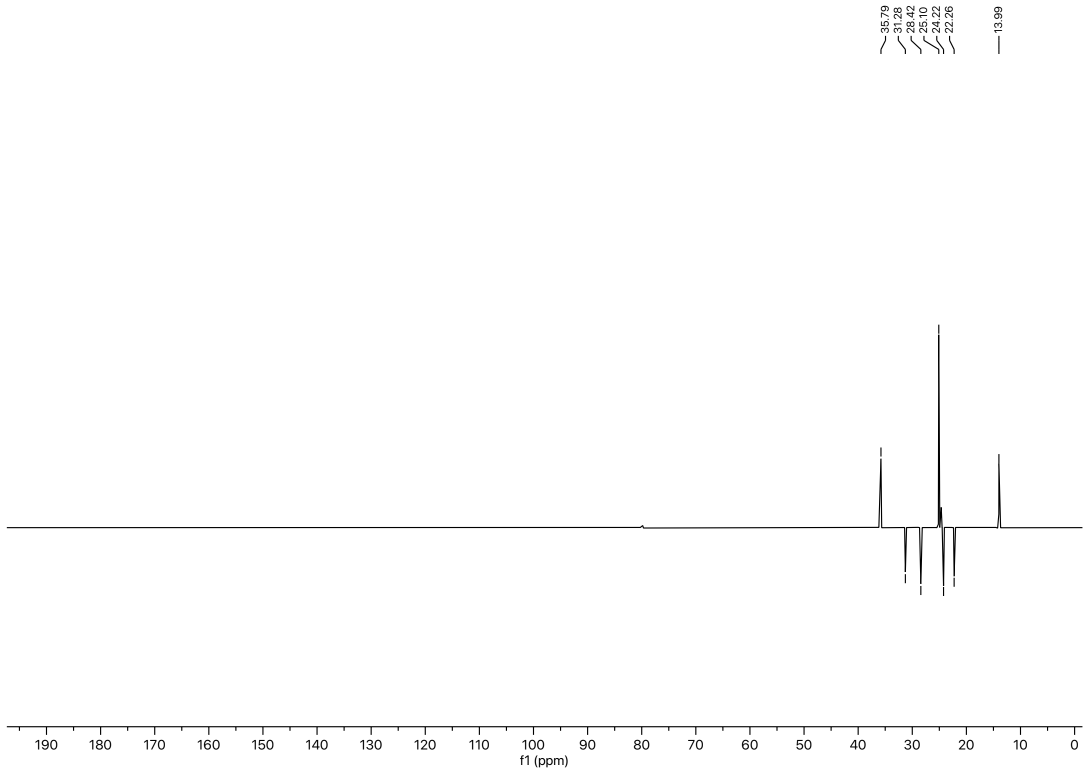

^{11}B NMR (2a)

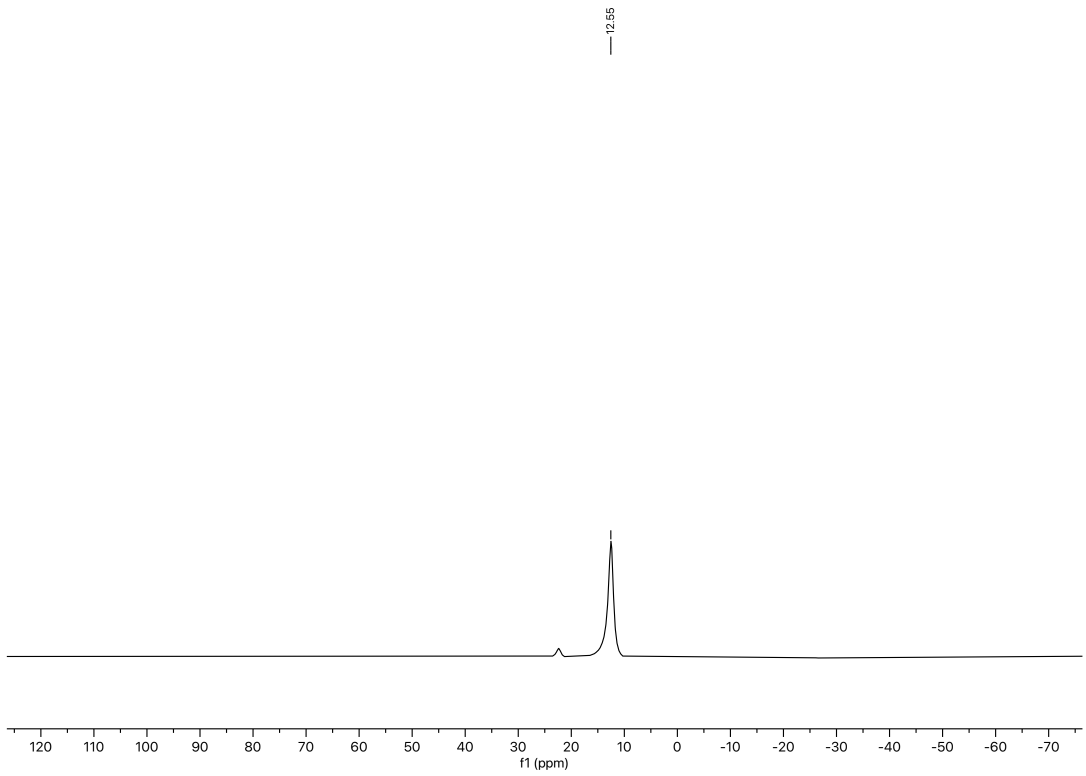

^{1}H NMR (2b)

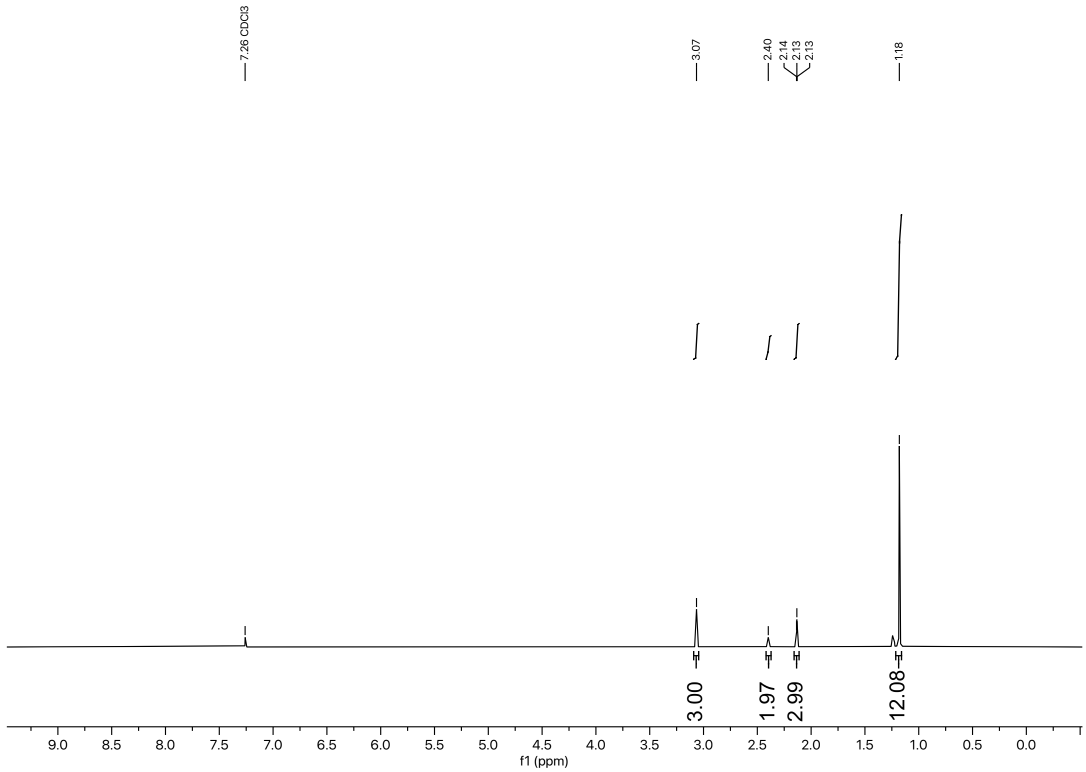

^{13}C NMR (2b)

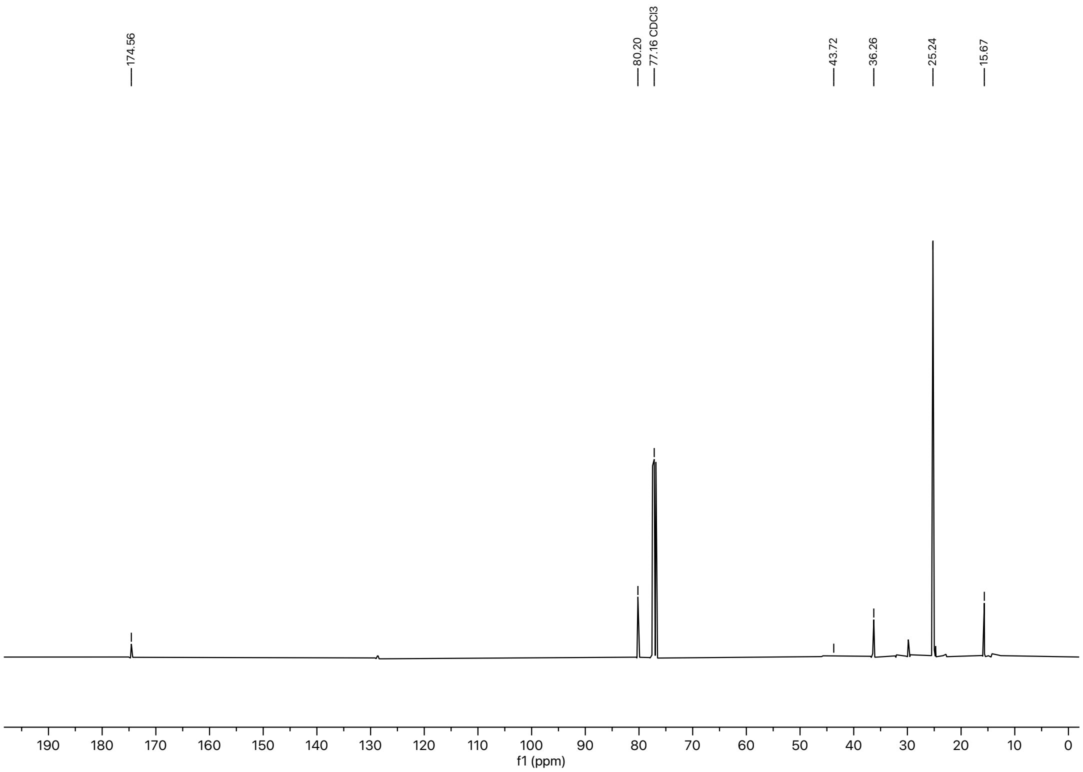

^{13}C DEPT-135 (2b)

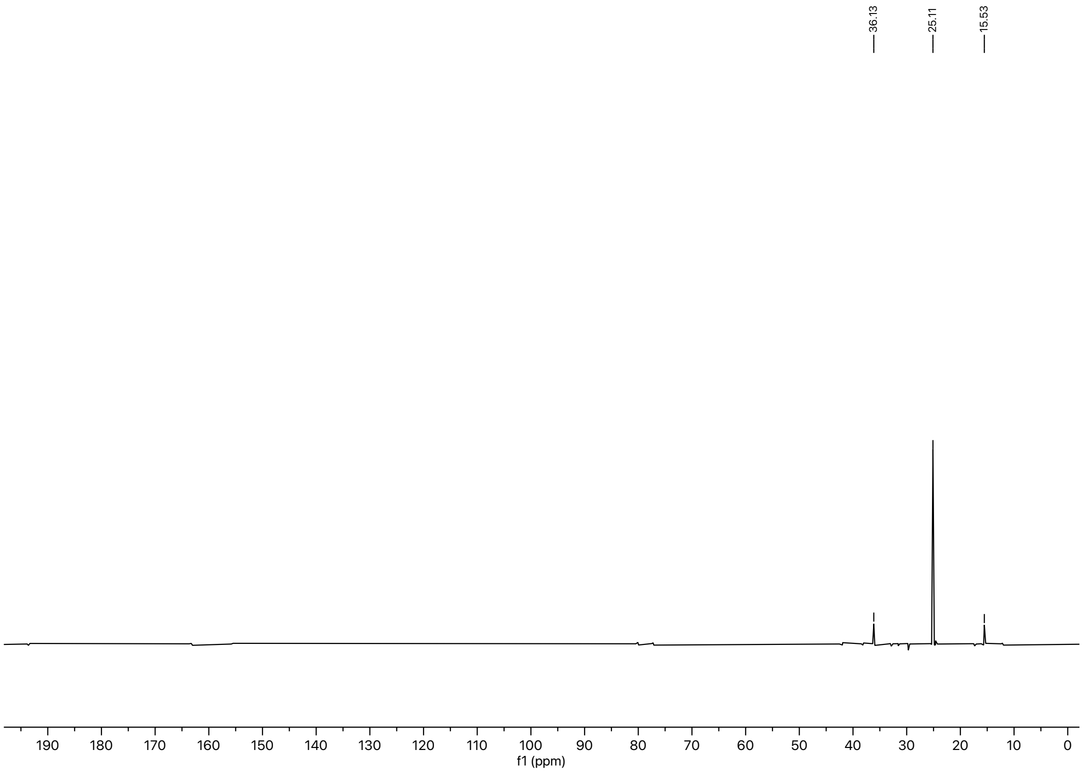

^{11}B NMR (2b)

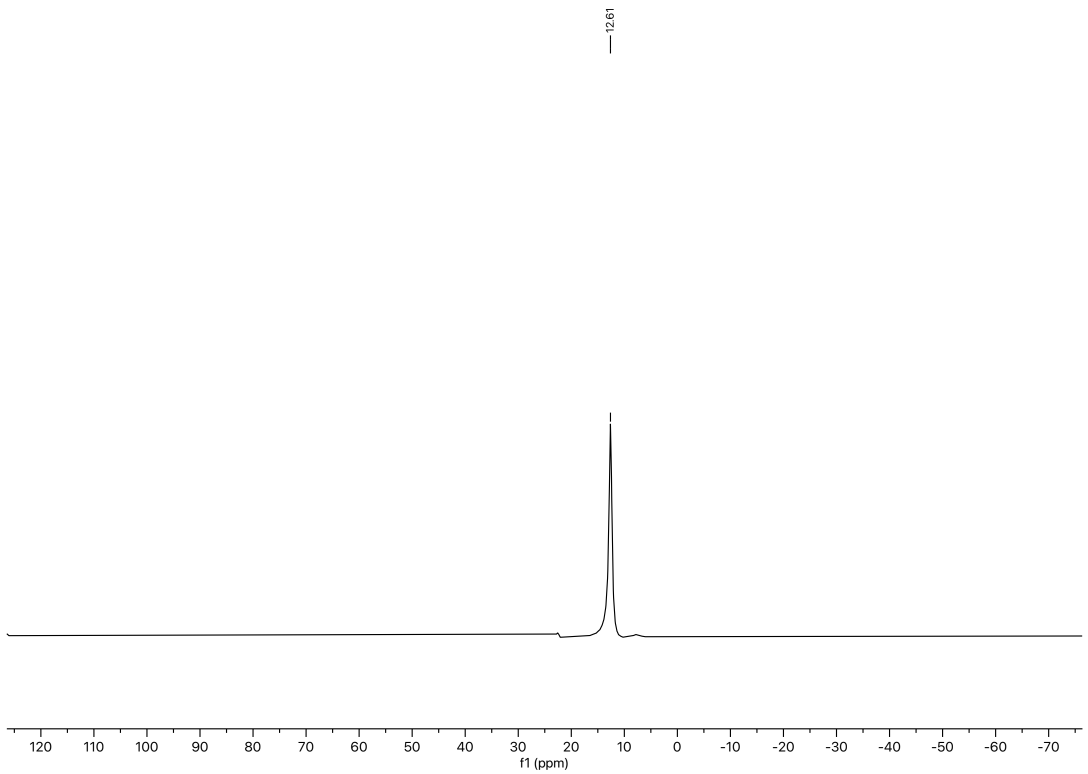

^{1}H NMR (2c)

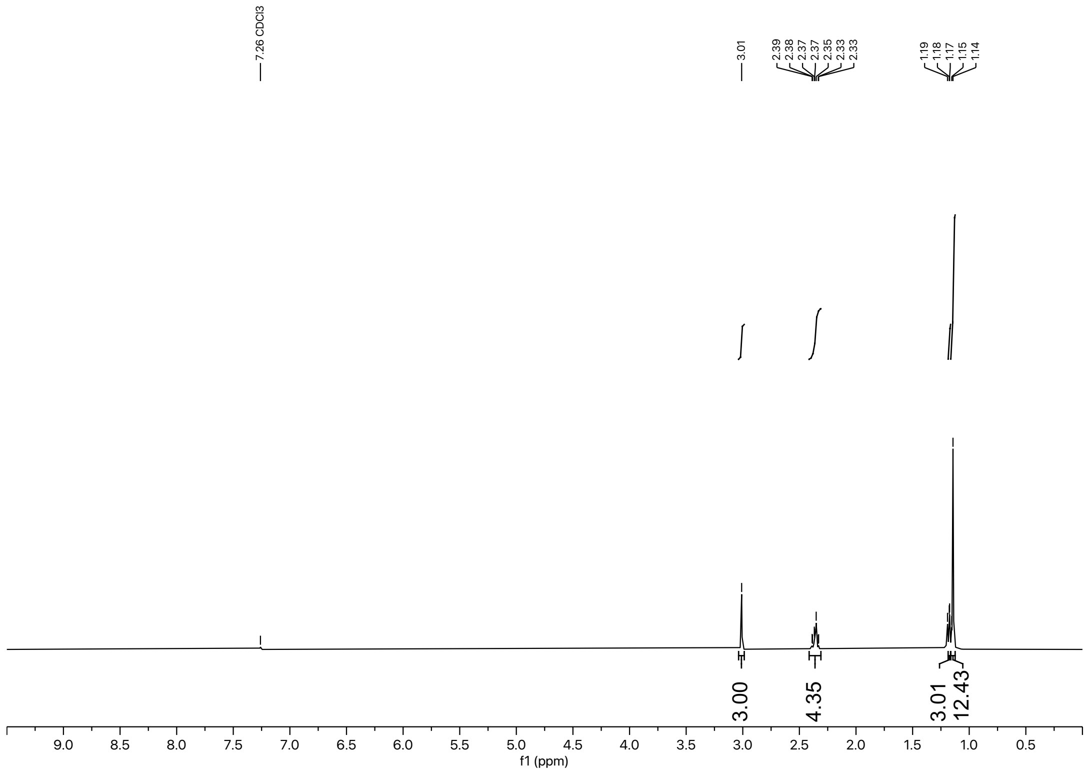

^{13}C NMR (2c)

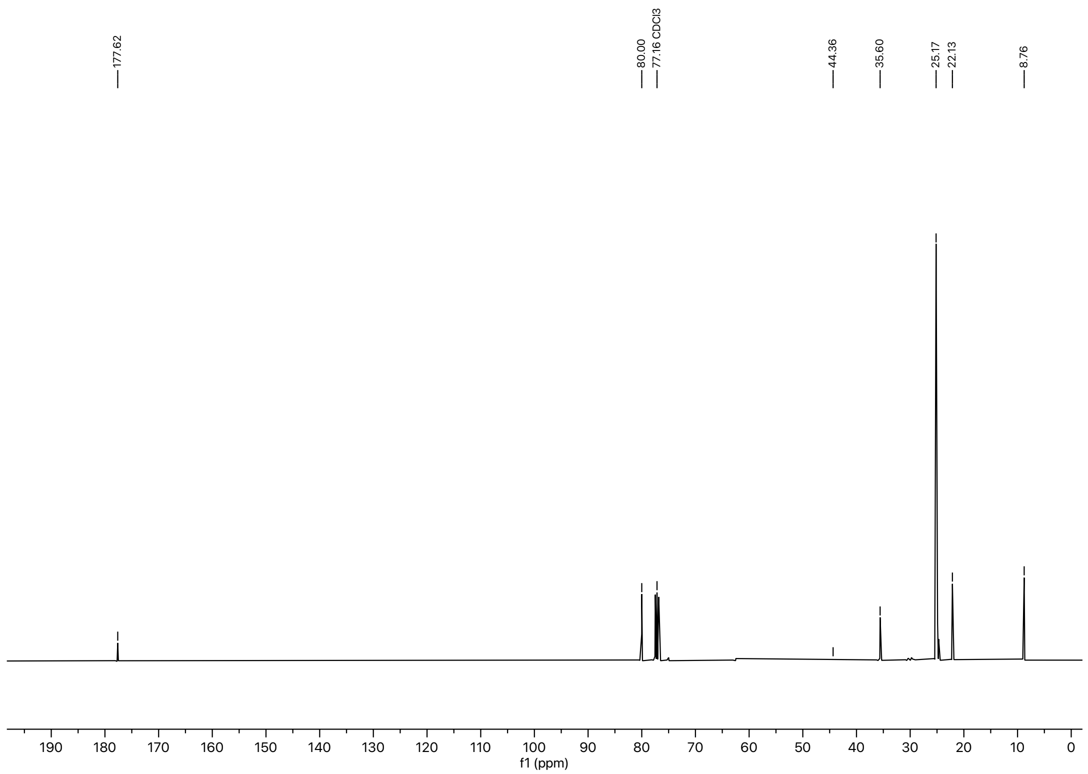

^{13}C DEPT-135 (2c)

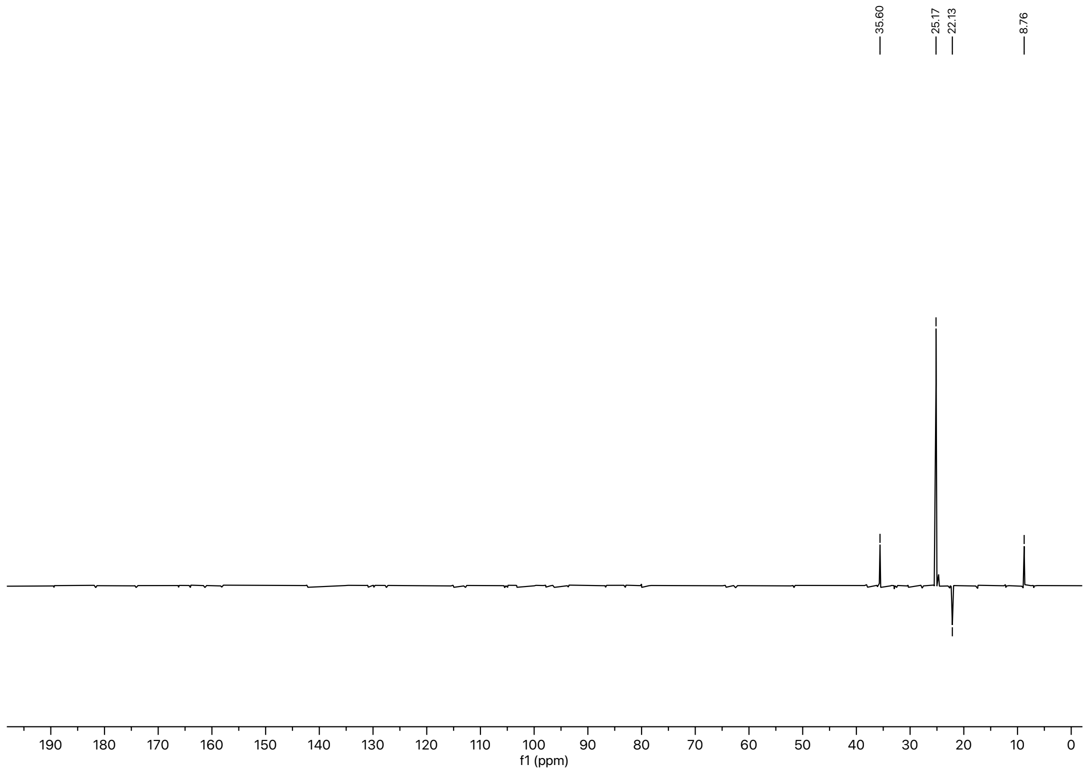

^{11}B NMR (2c)

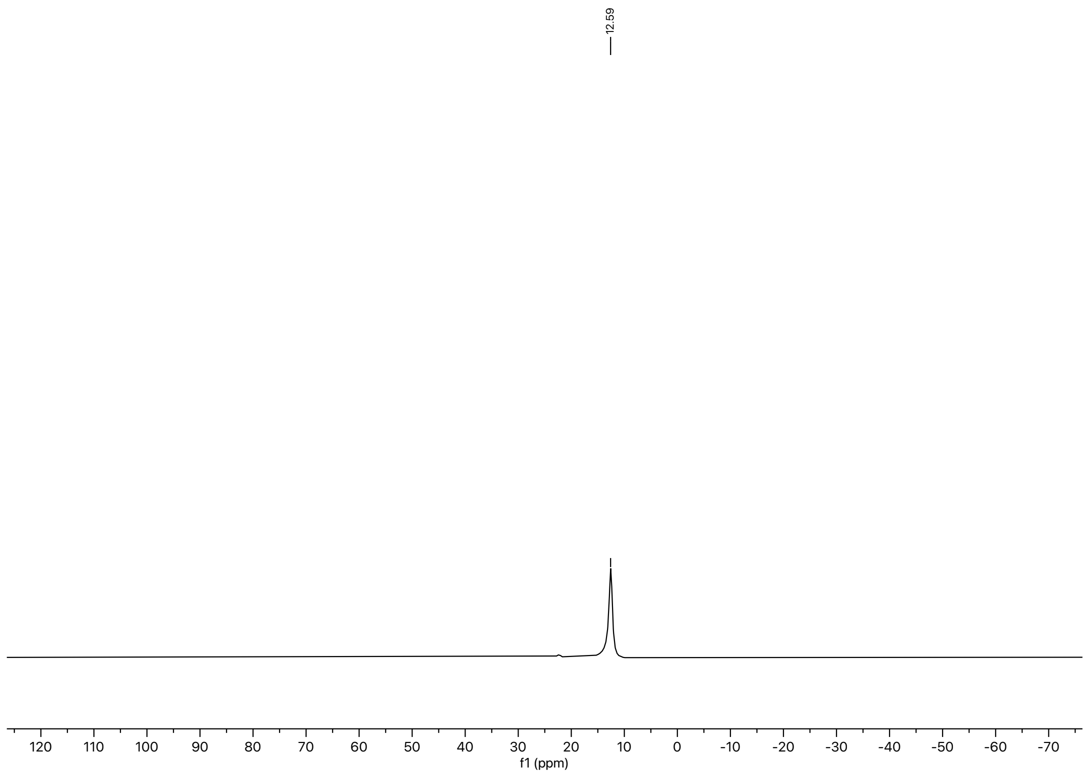

^{1}H NMR (2d)

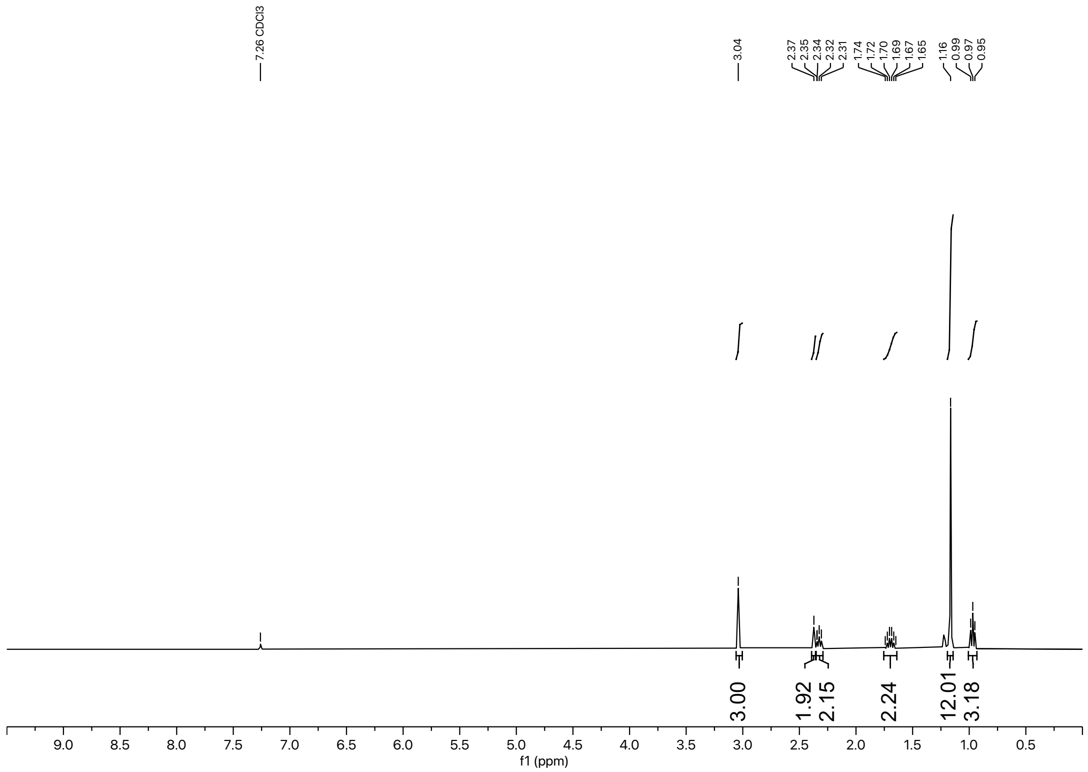

^{13}C NMR (2d)

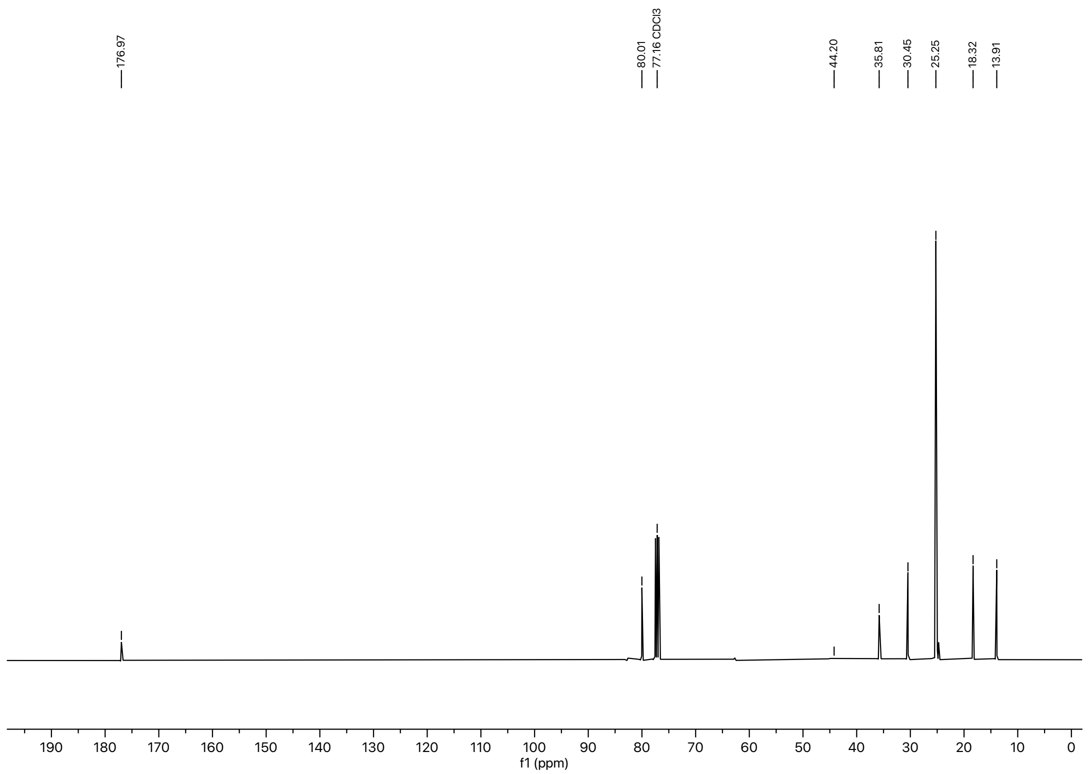

^{13}C DEPT-135 (2d)

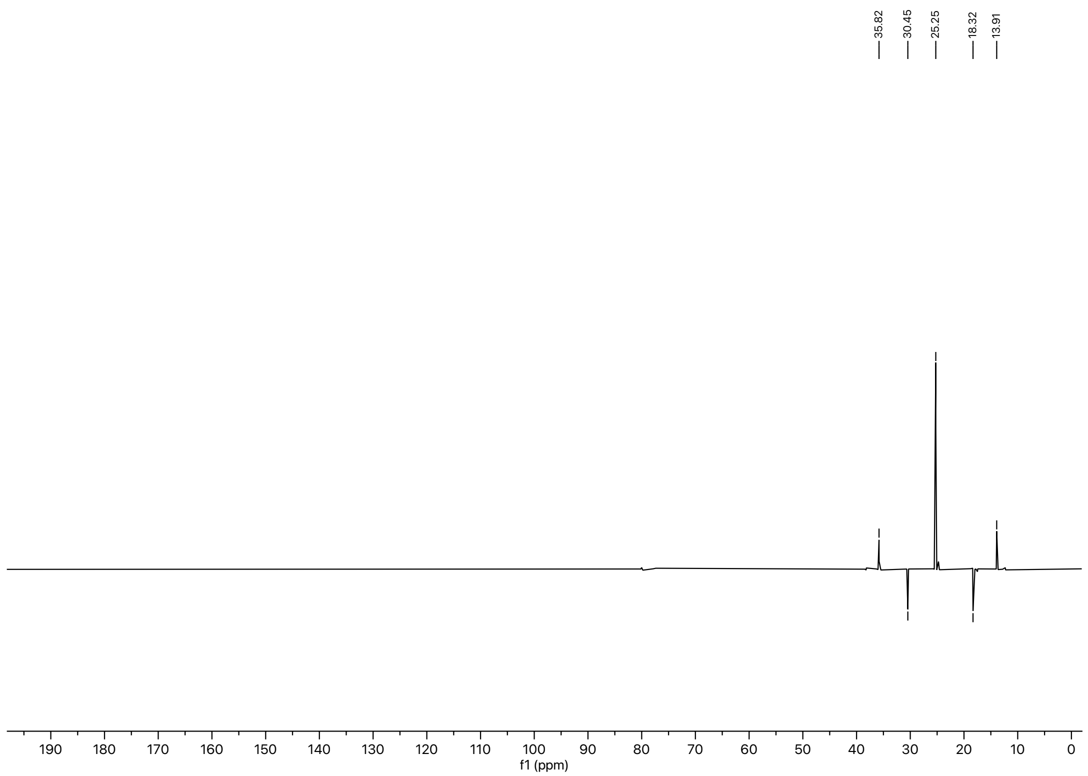

^{11}B NMR (2d)

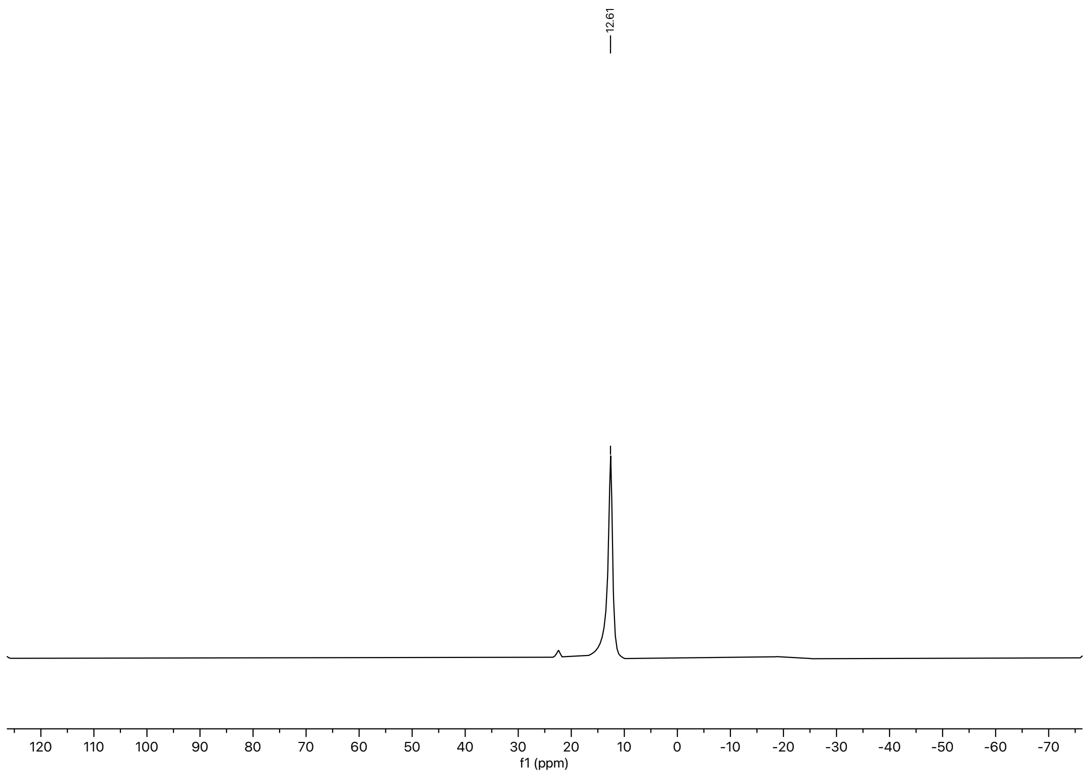

^{1}H NMR (2e)

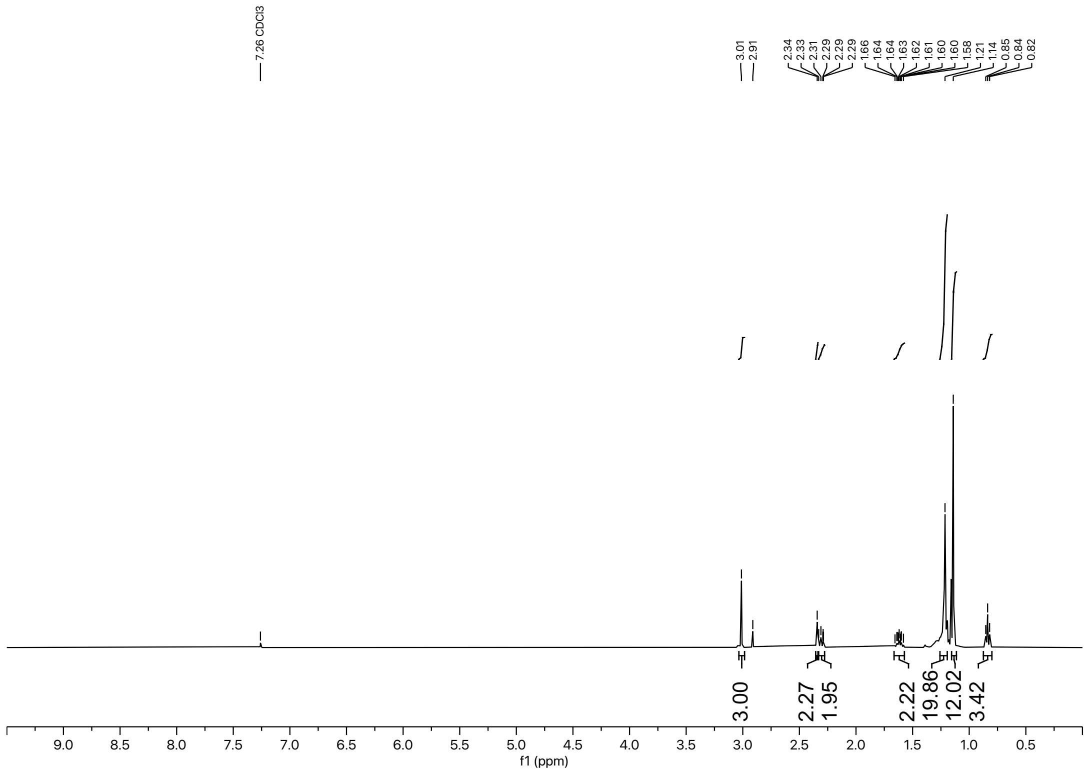

^{13}C NMR (2e)

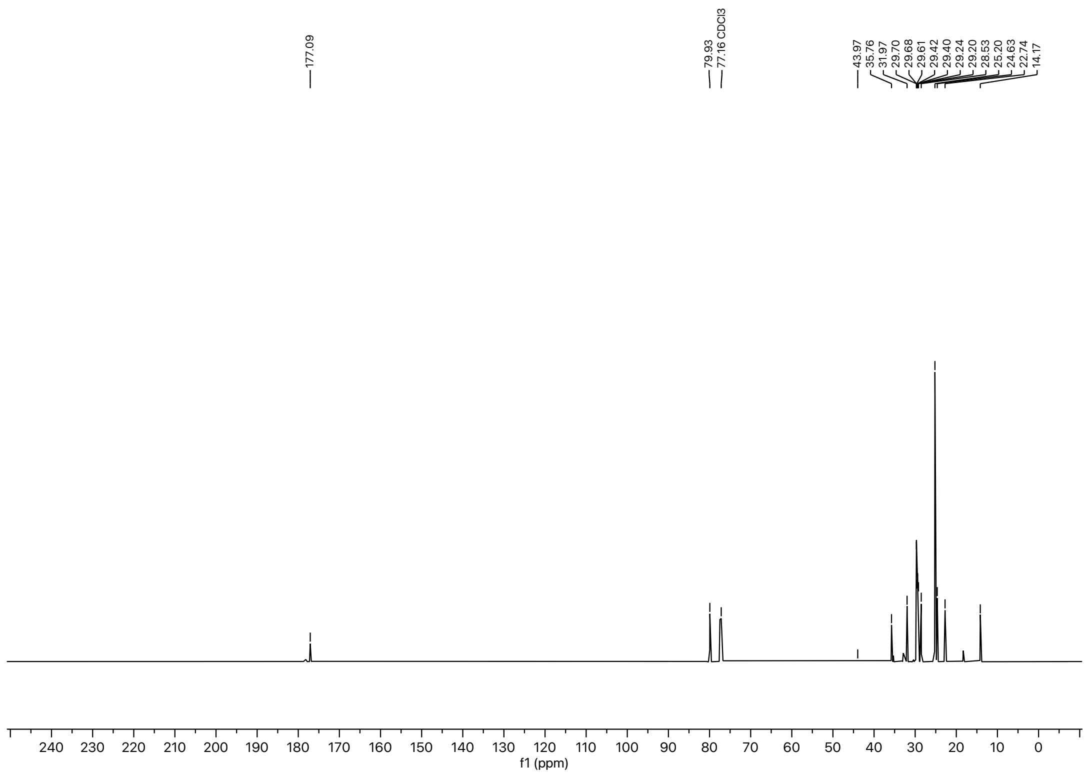

^{13}C DEPT-135 (2e)

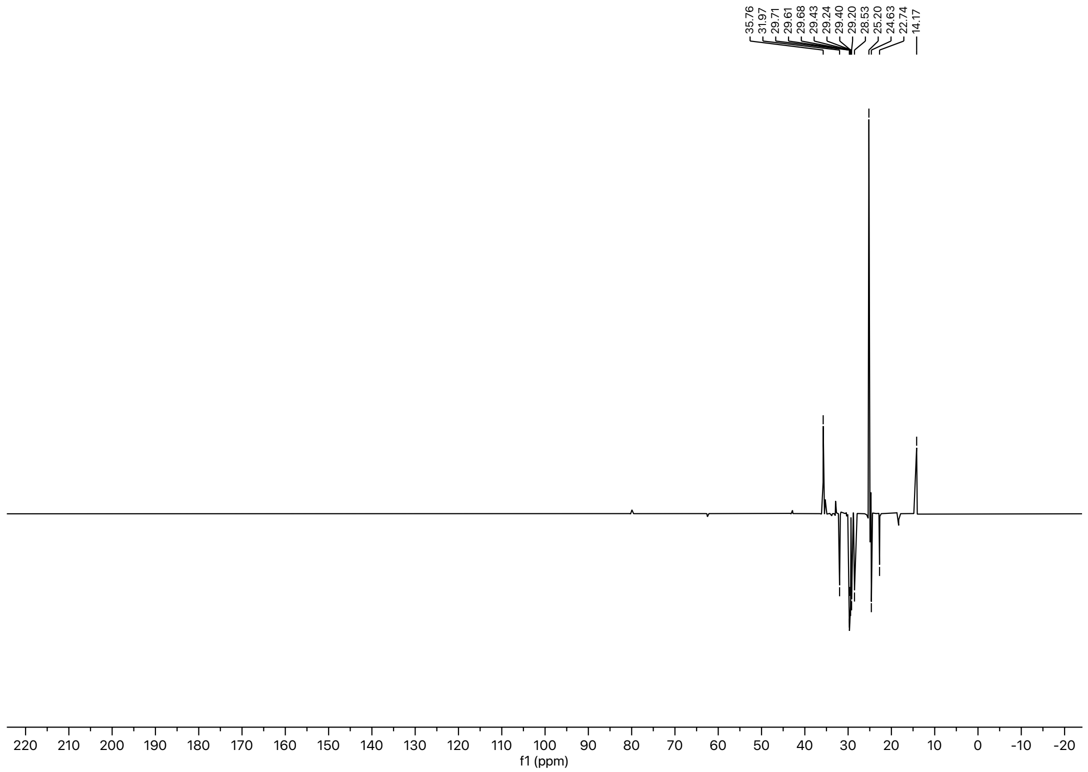

^{11}B NMR (2e)

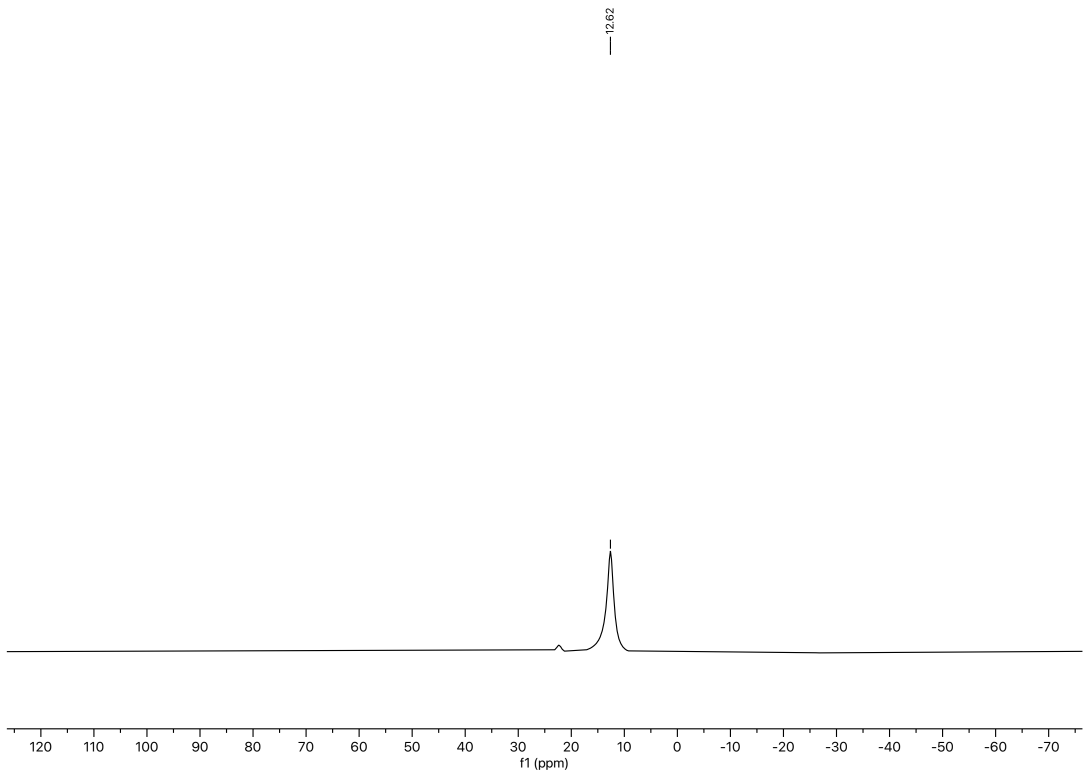

^{1}H NMR (2f)

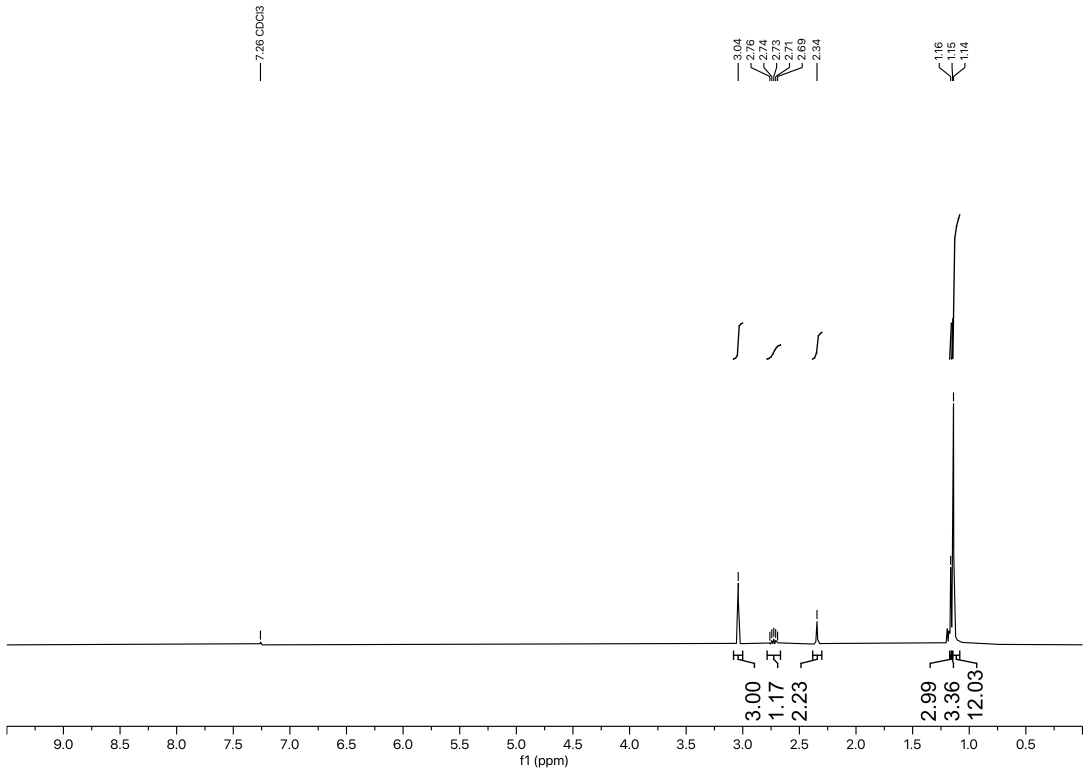

^{13}C NMR (2f)

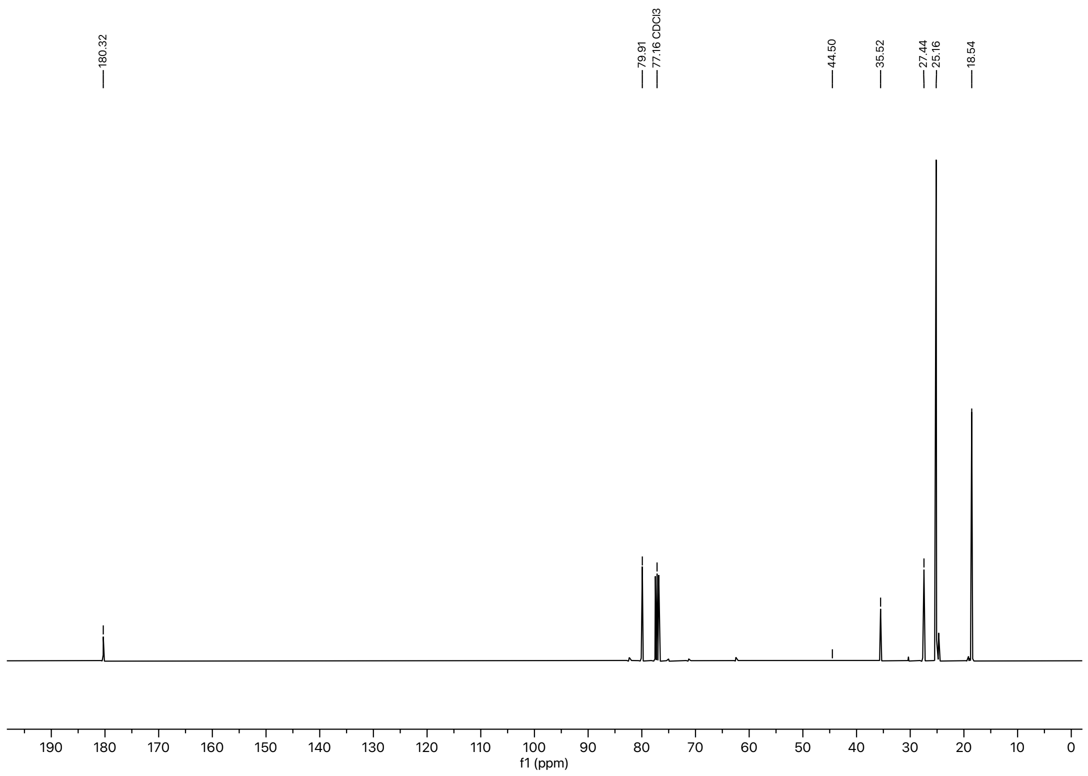

^{13}C DEPT-135 (2f)

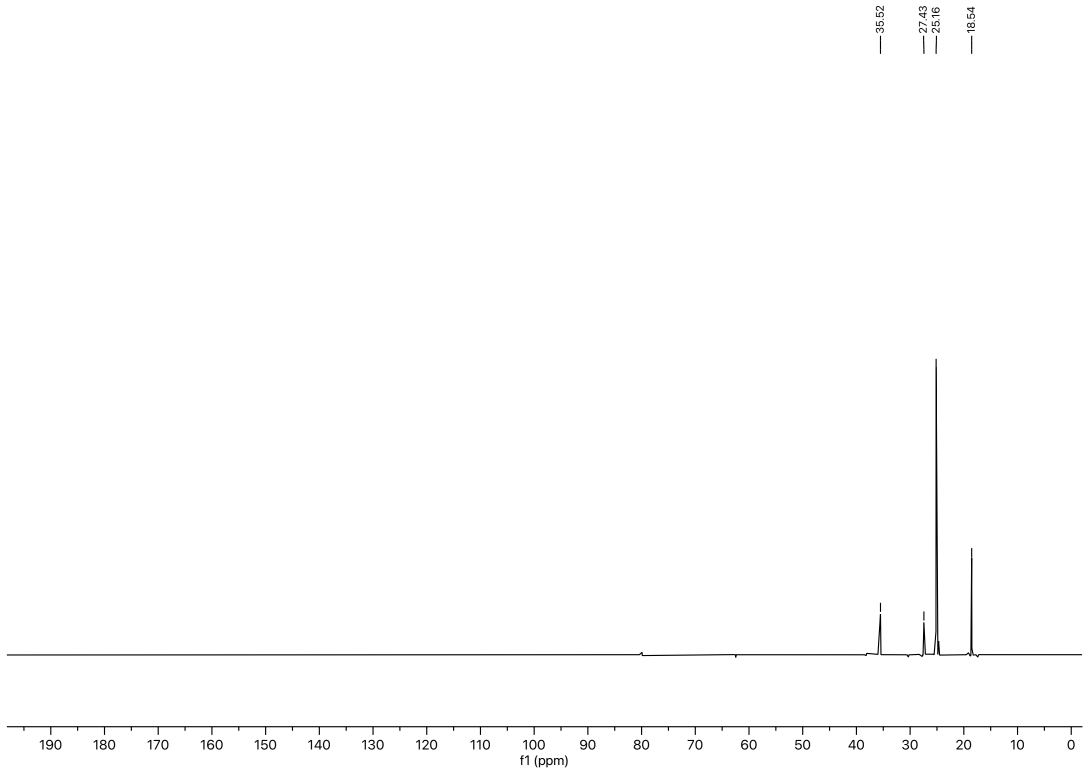

^{11}B NMR (2f)

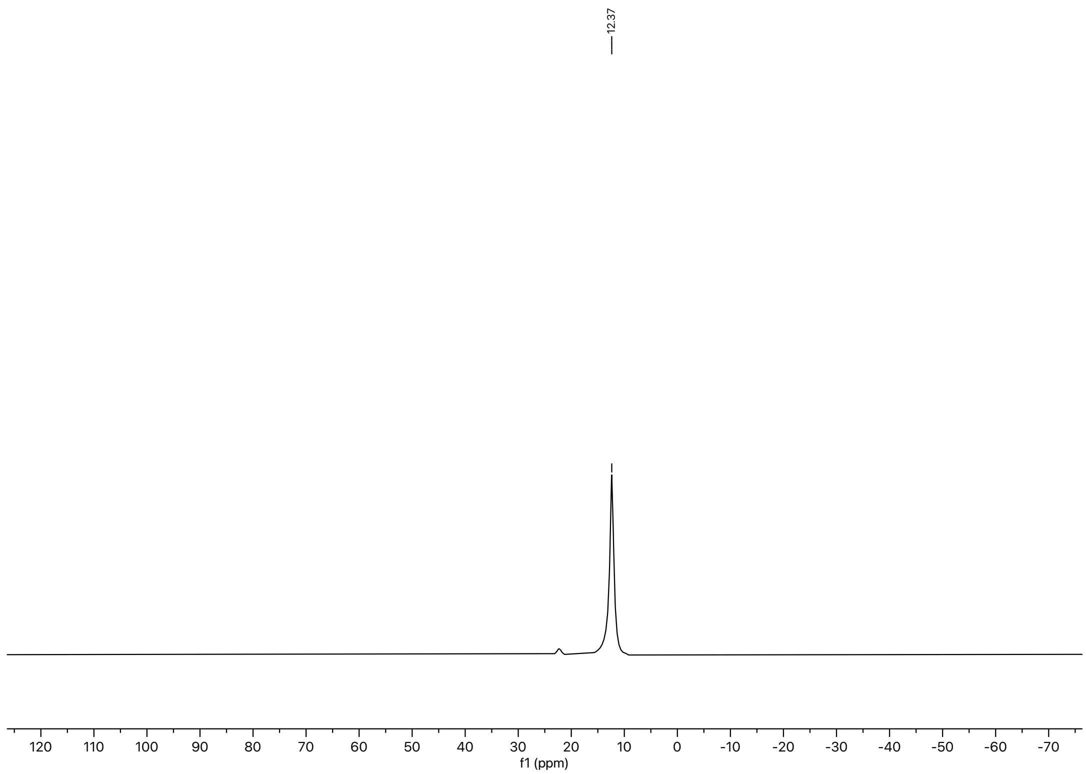

^{1}H NMR (2g)

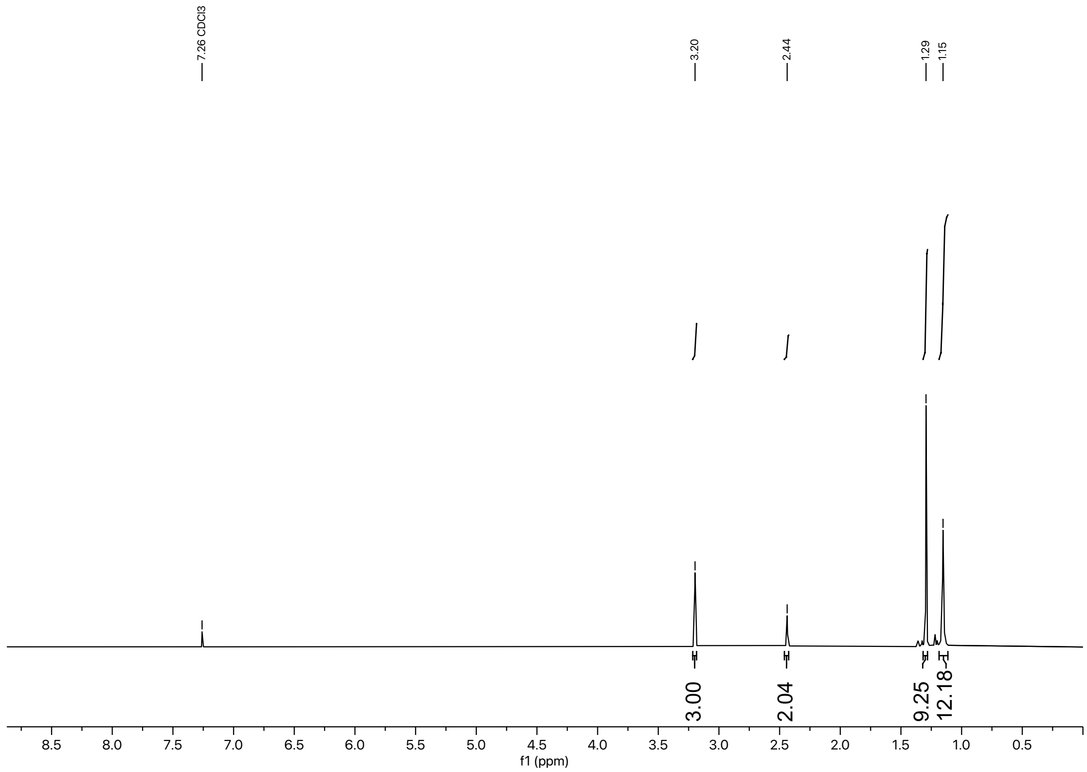

^{13}C NMR (2g)

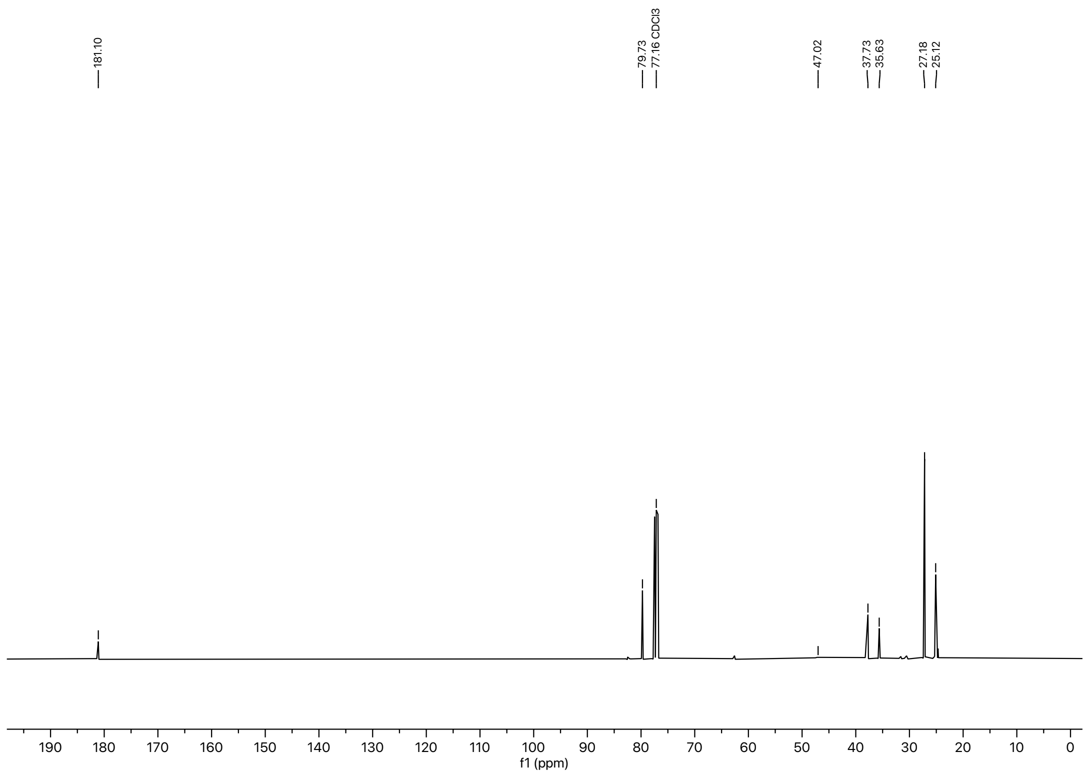

^{13}C DEPT-135 (2g)

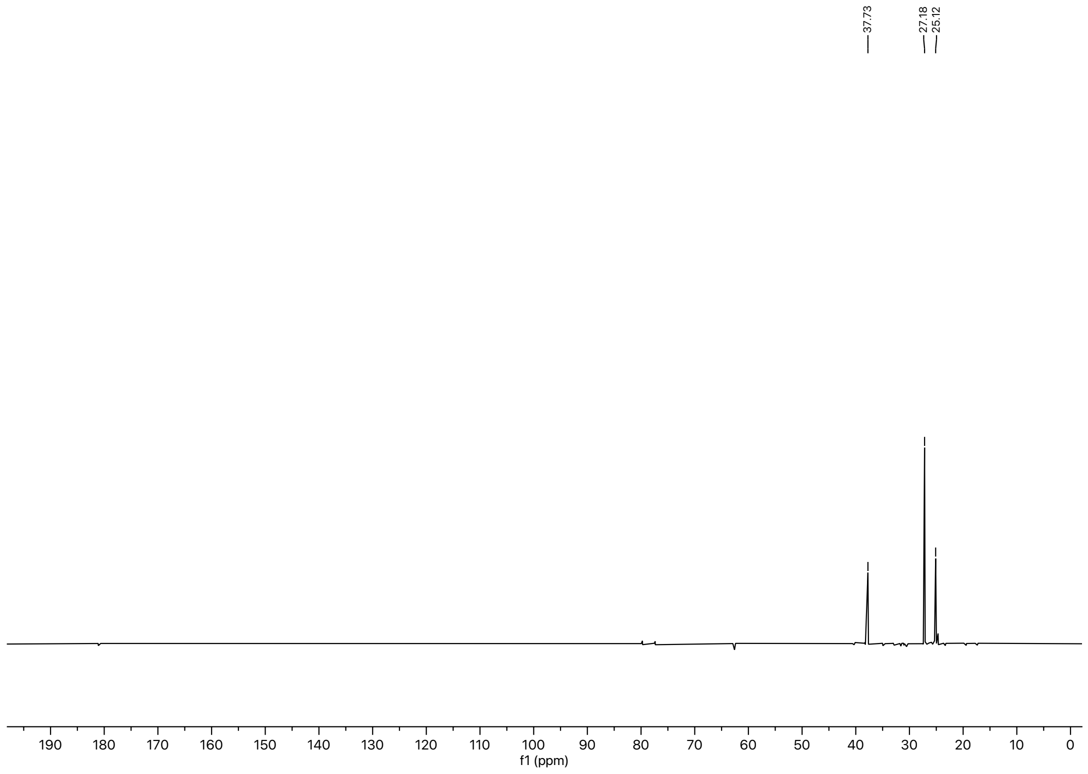

^{11}B NMR (2g)

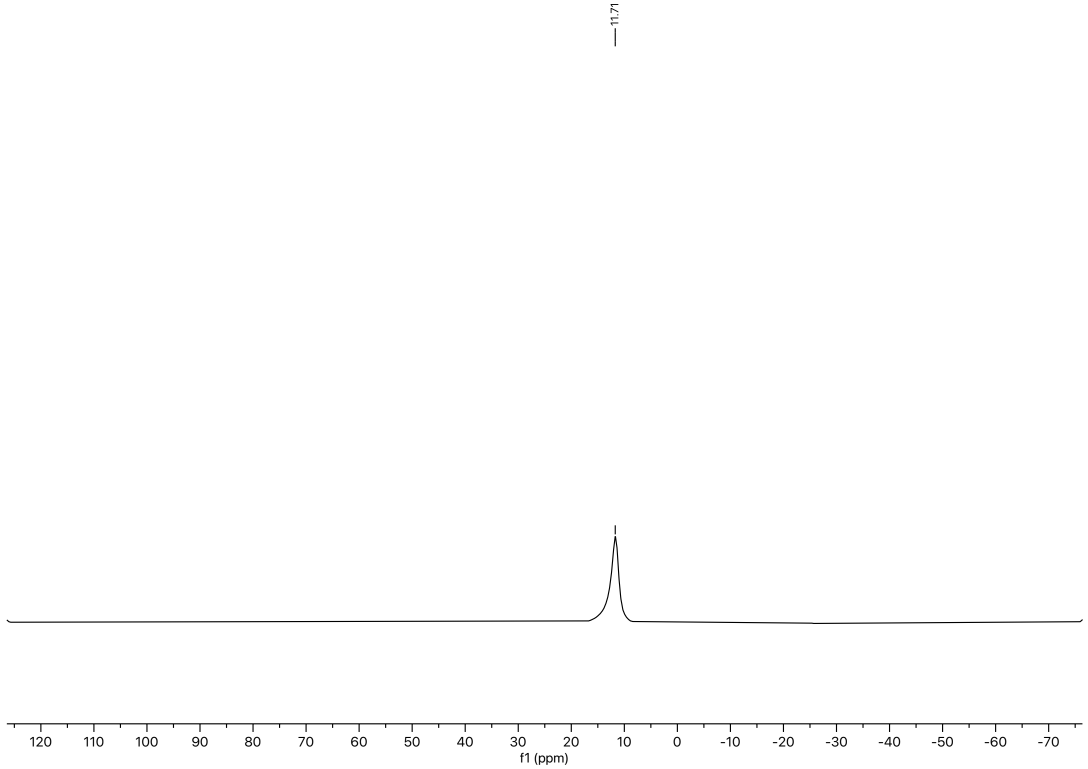

^{1}H NMR (2h)

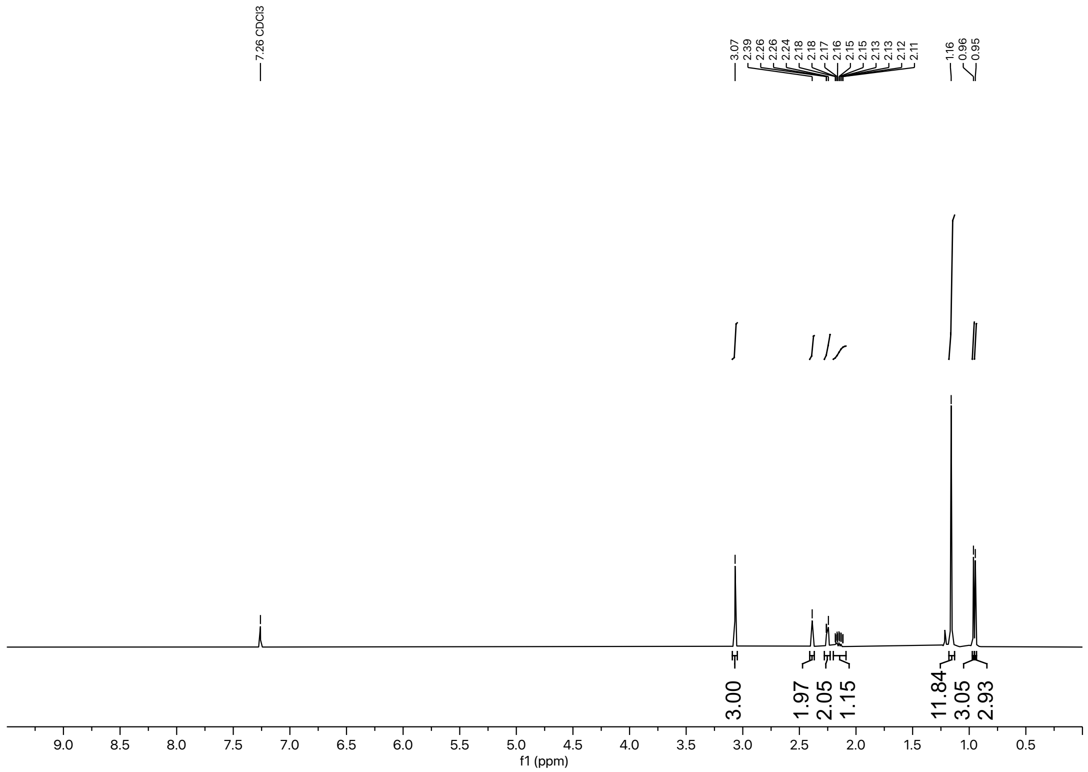

^{13}C NMR (2h)

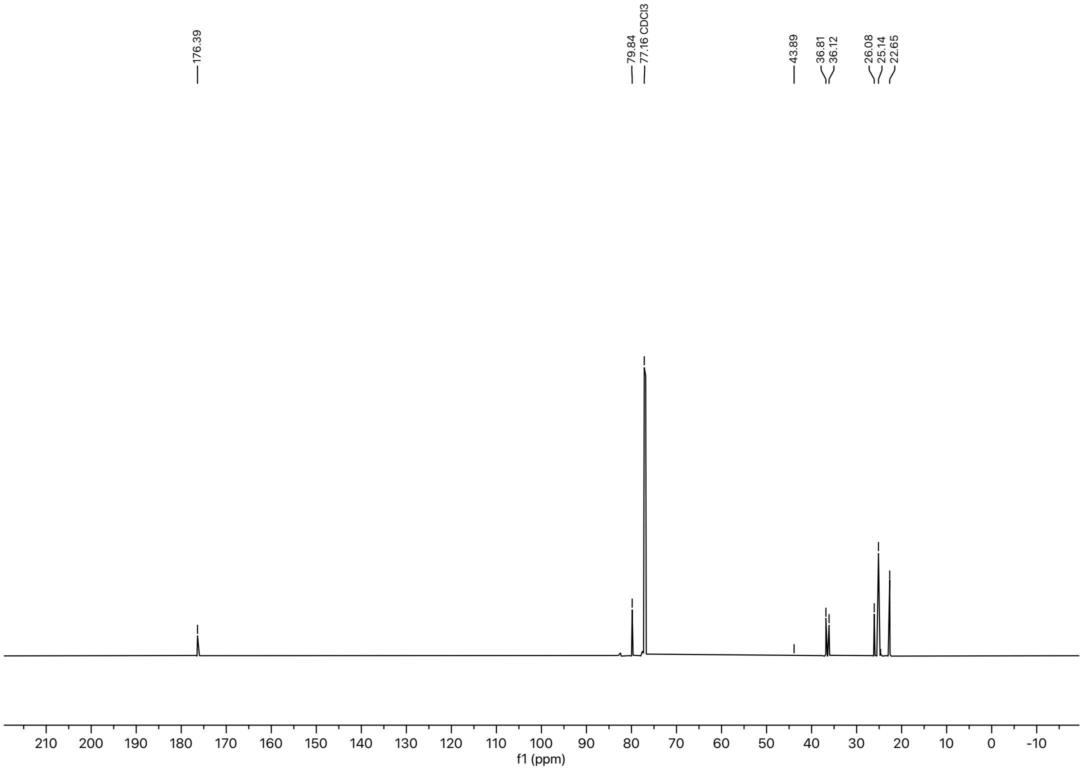

^{13}C DEPT-135 (2h)

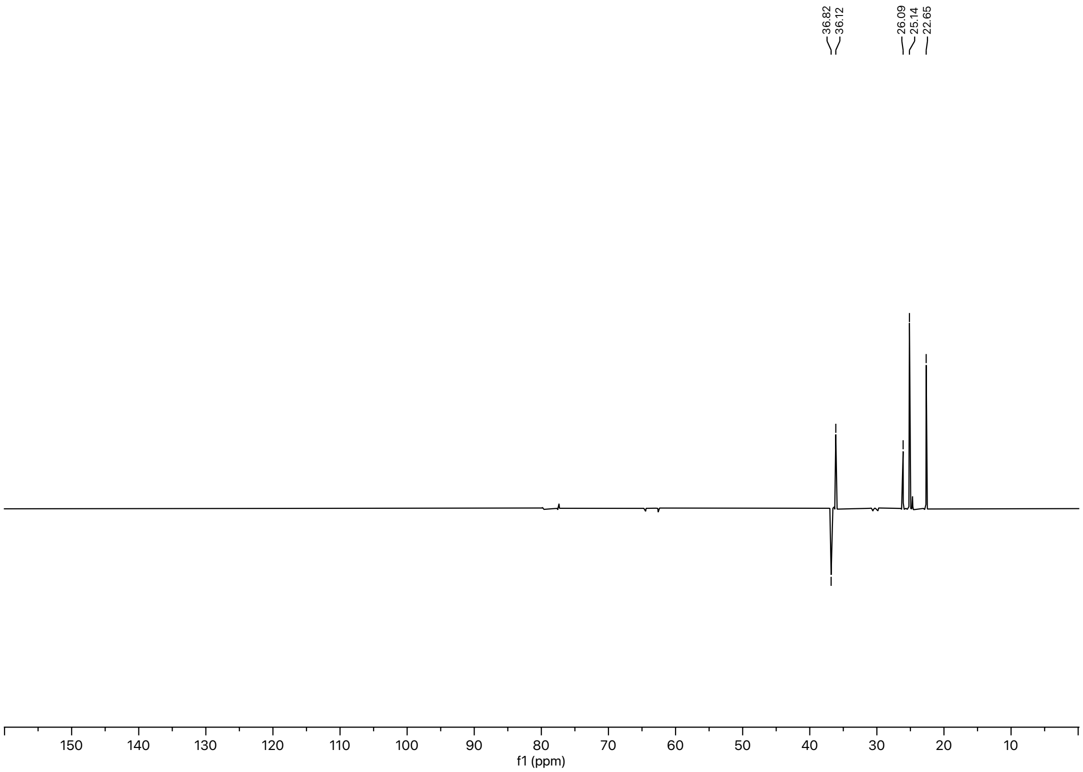

^{11}B NMR (2h)

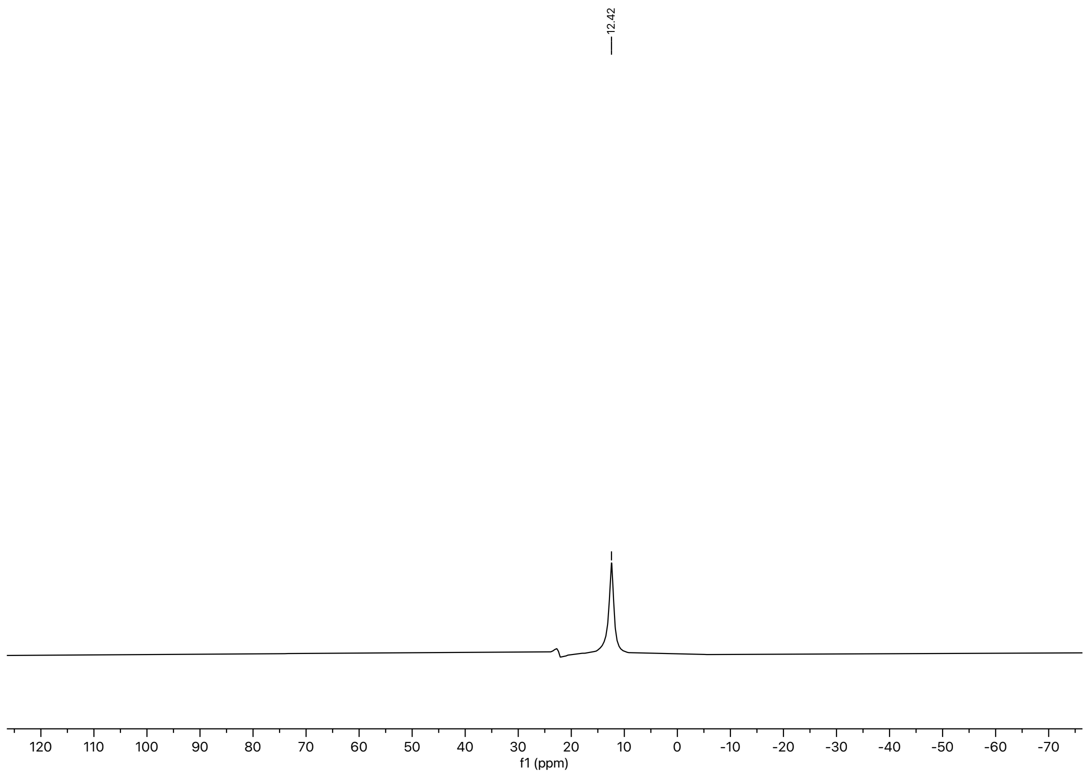

^{1}H NMR (2i)

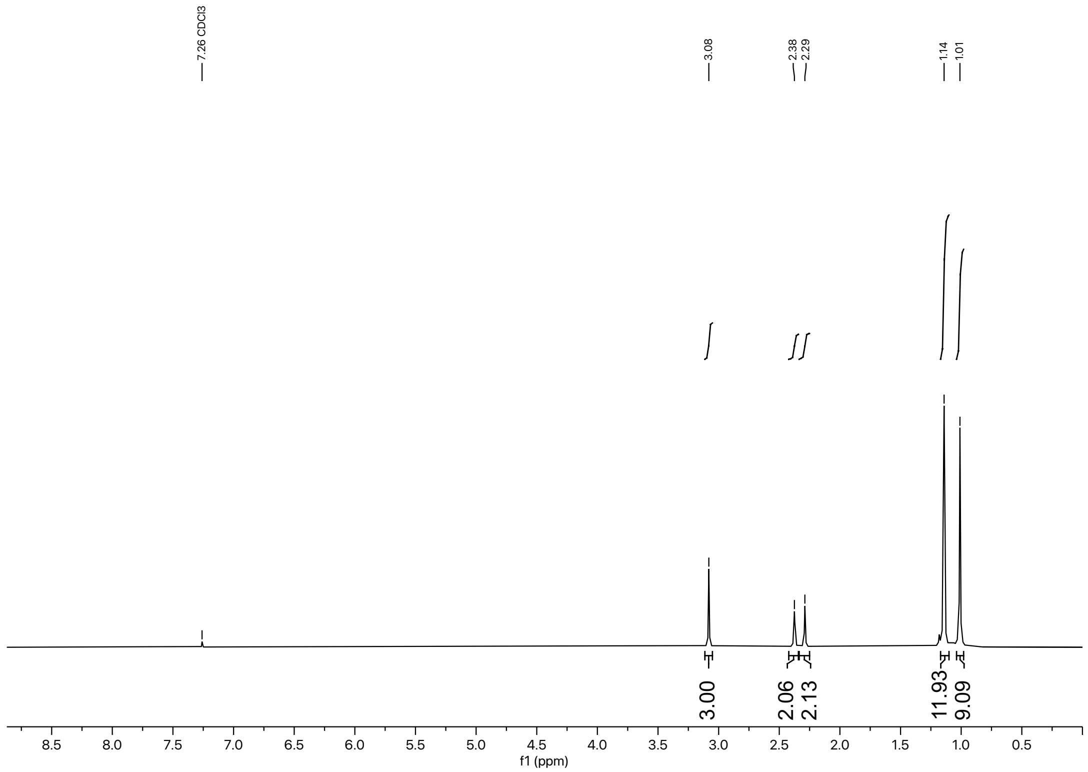

^{13}C NMR (2i)

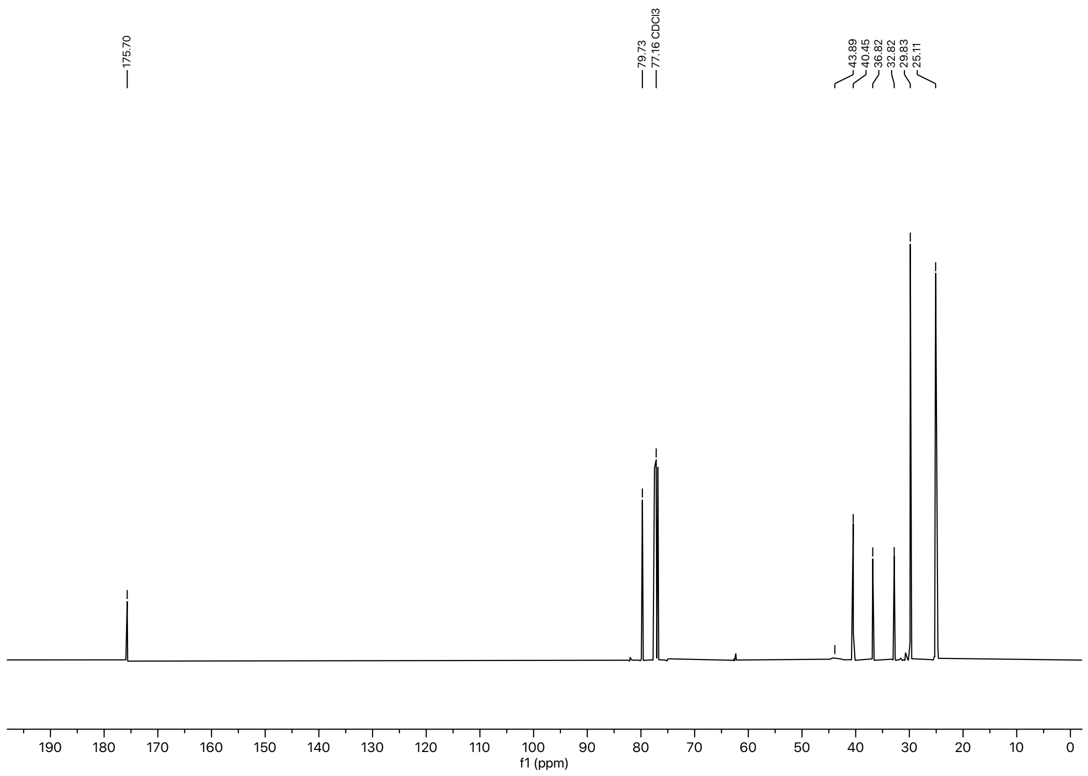

^{13}C DEPT-135 (2i)

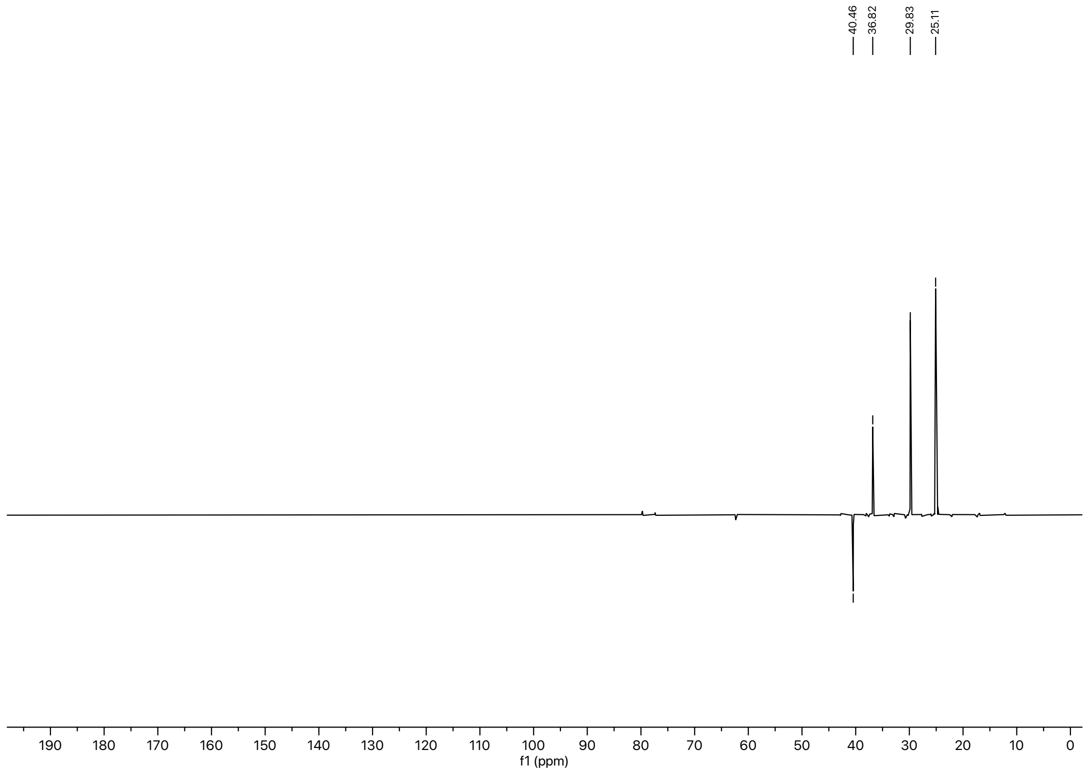

^{11}B NMR (2i)

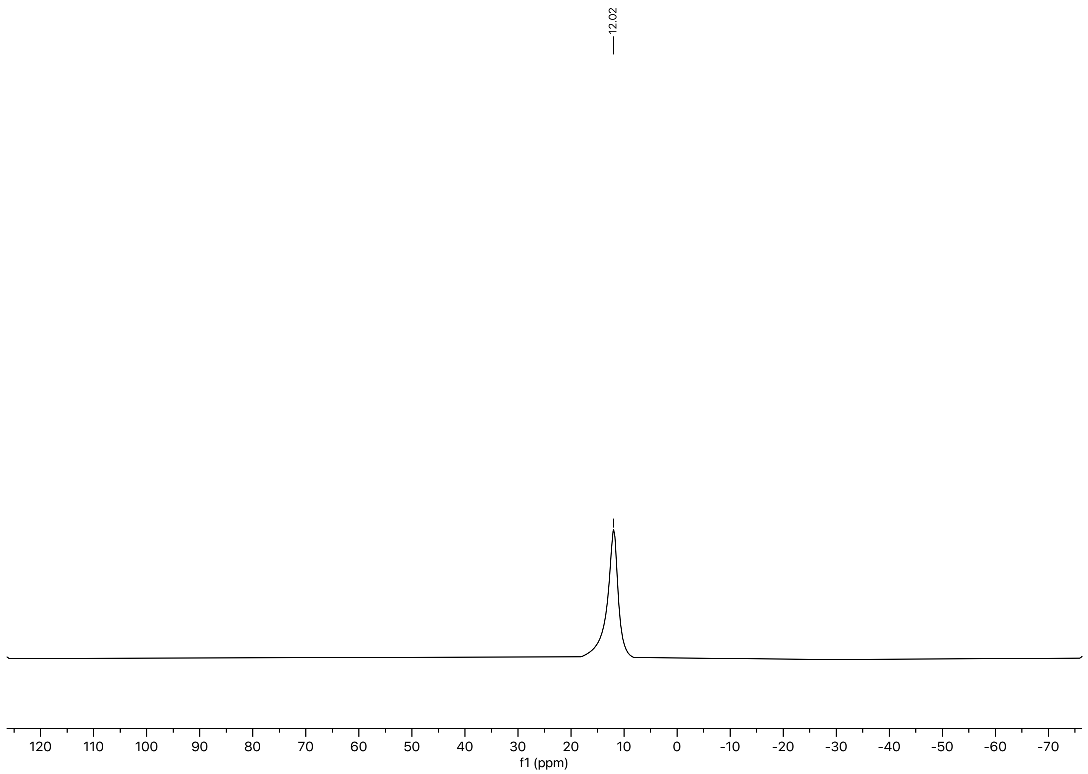

^{1}H NMR (2j)

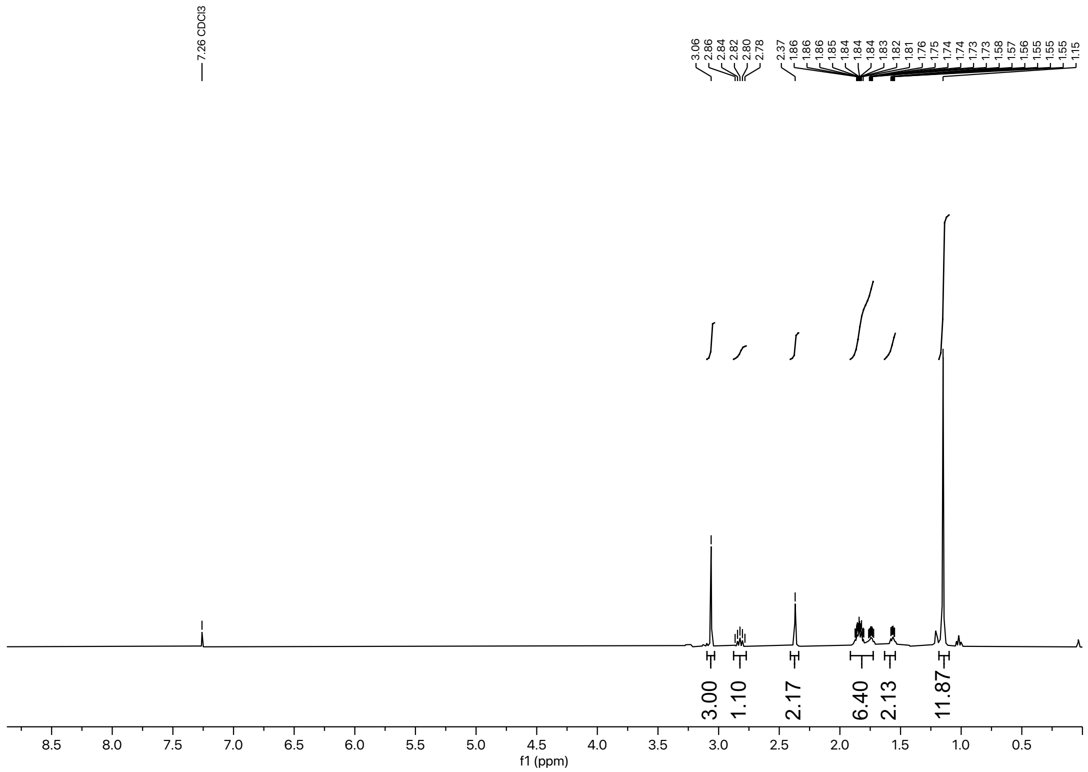

^{13}C NMR (2j)

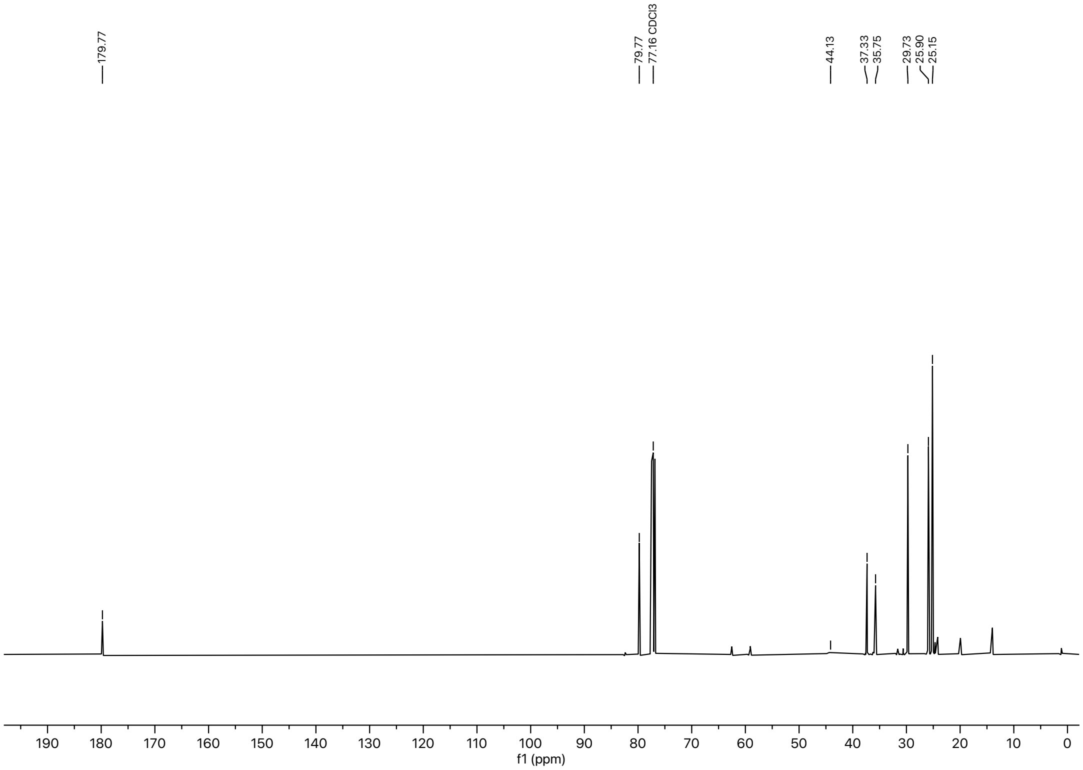

^{13}C DEPT-135 (2j)

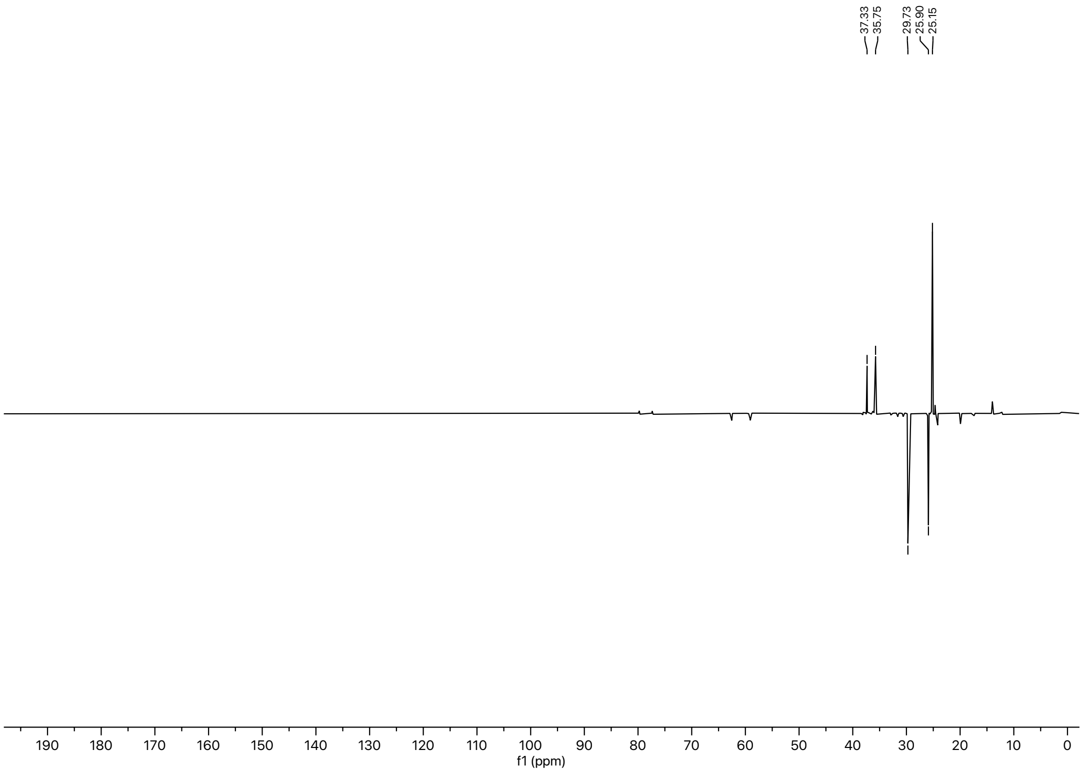

^{11}B NMR (2j)

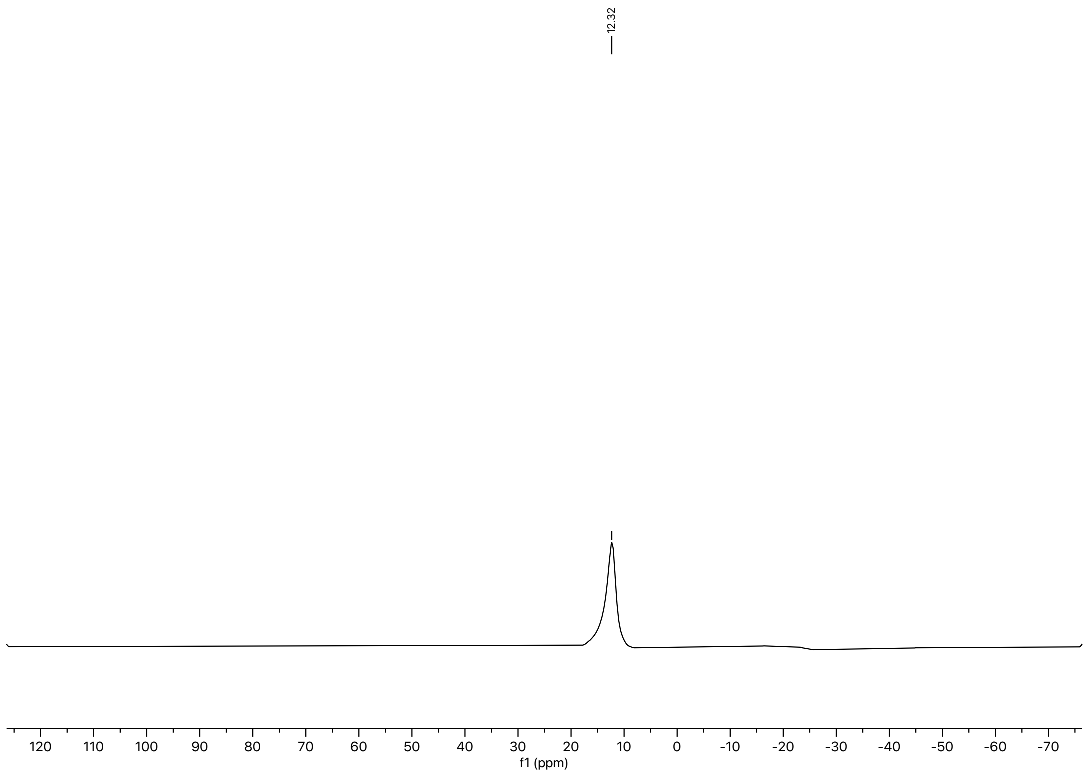

^{1}H NMR (2k)

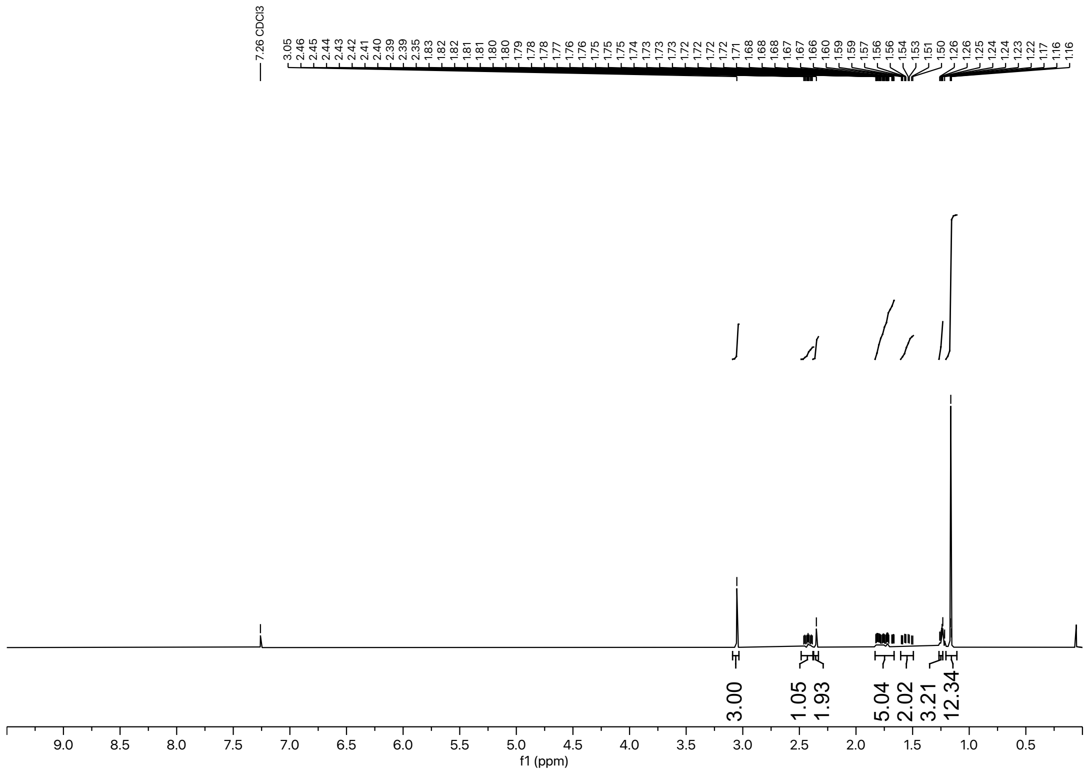

^{13}C NMR (2k)

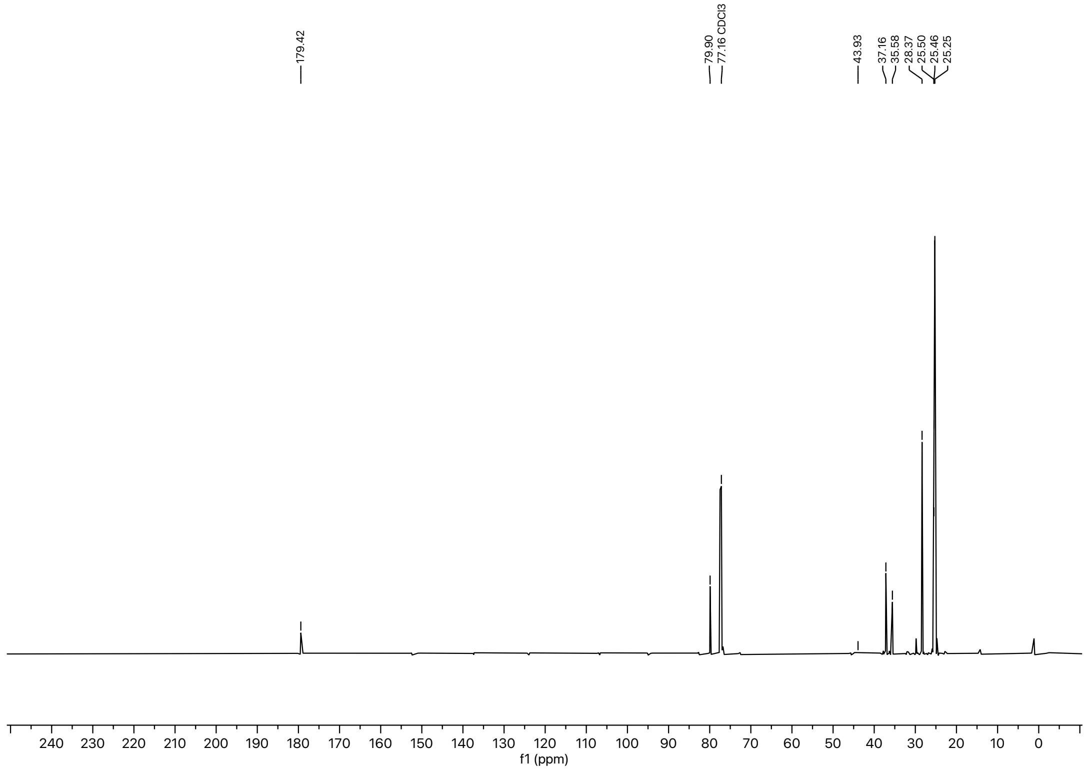

^{13}C DEPT-135 (2k)

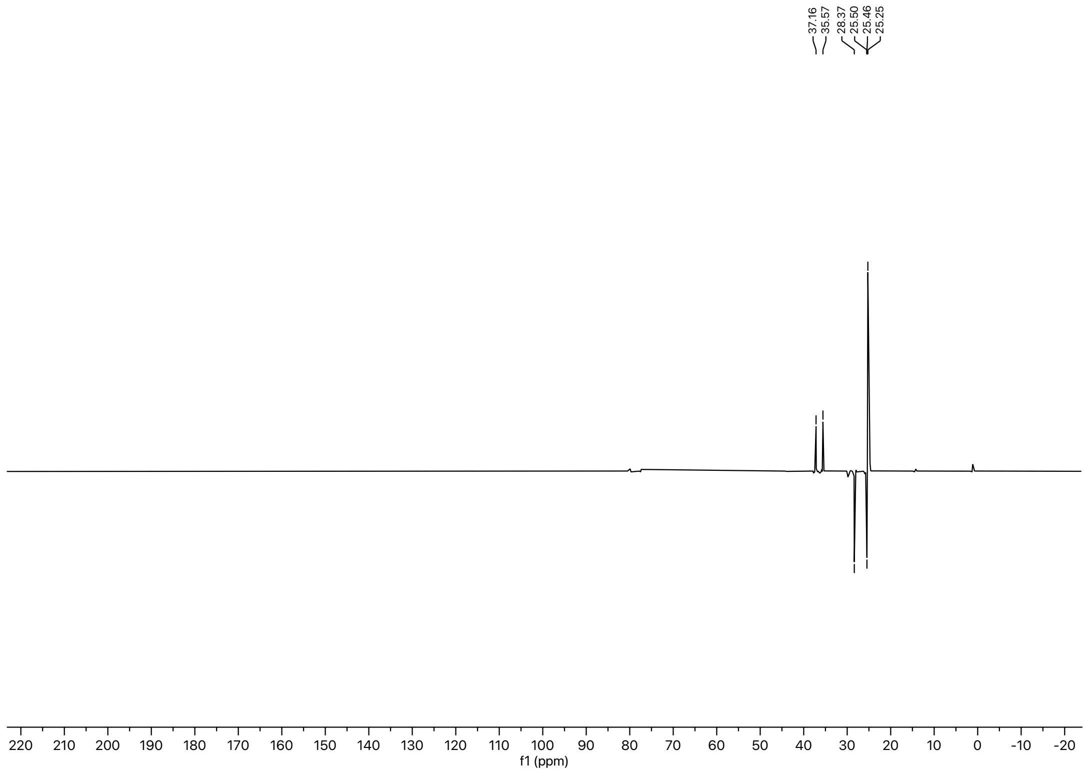

^{11}B NMR (2k)

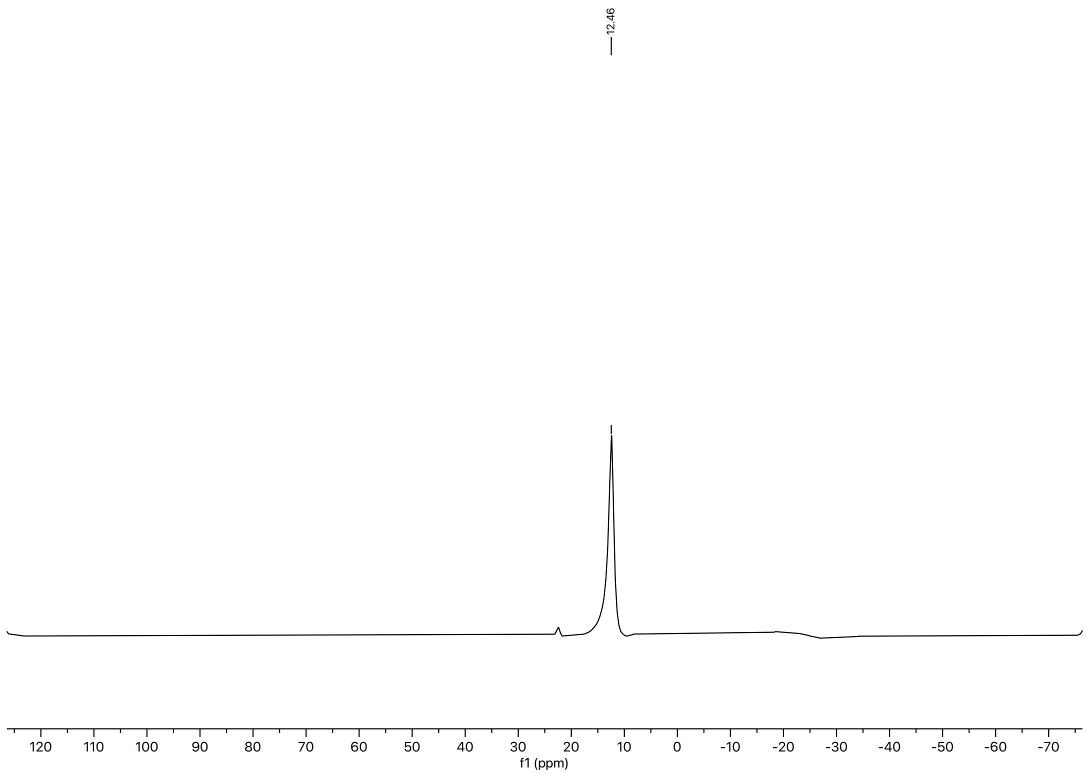

^{1}H NMR (2l)

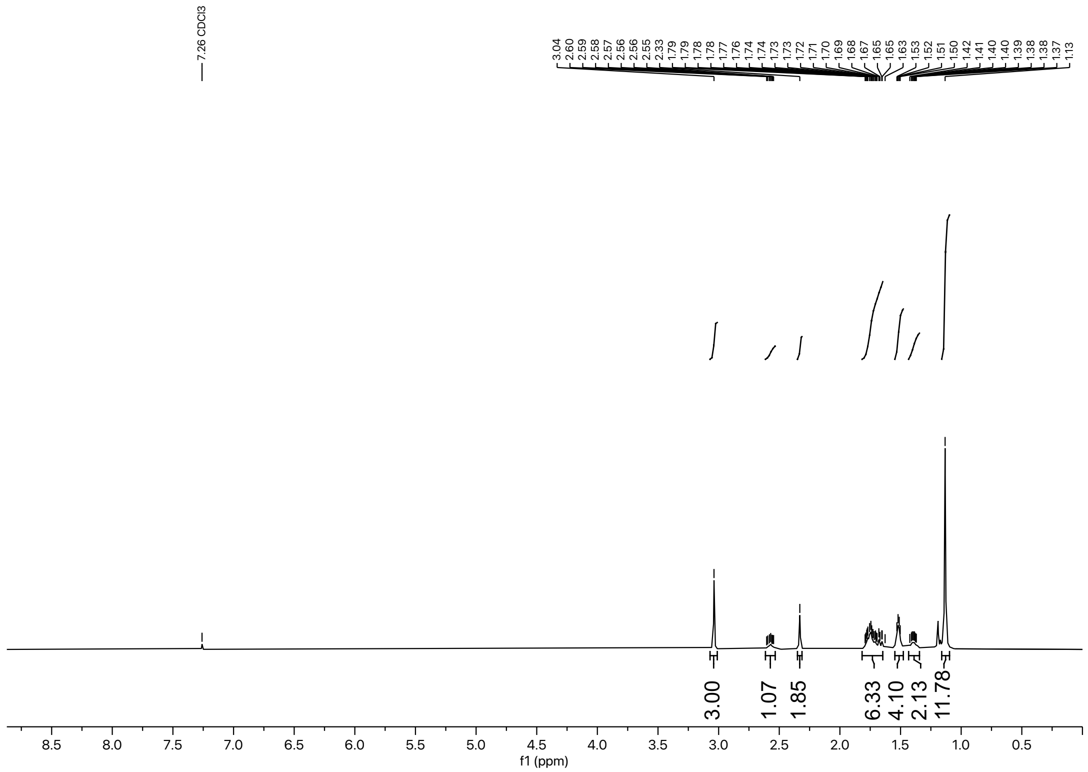

^{13}C NMR (2l)

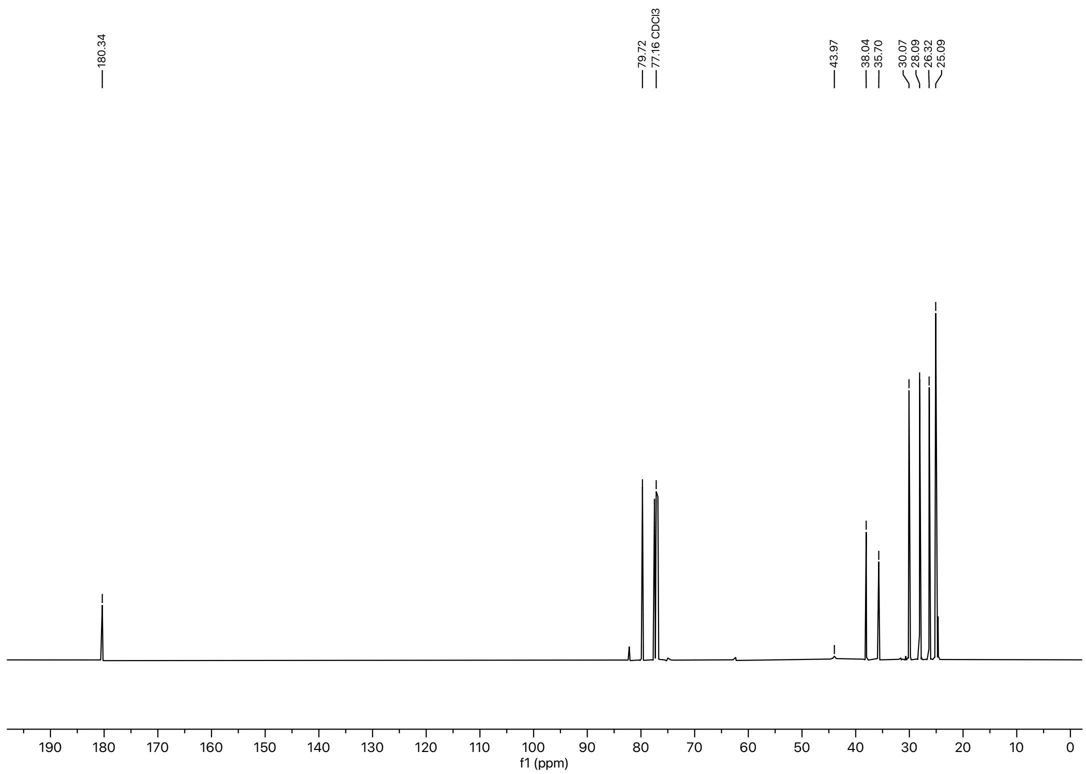

^{13}C DEPT-135 (2l)

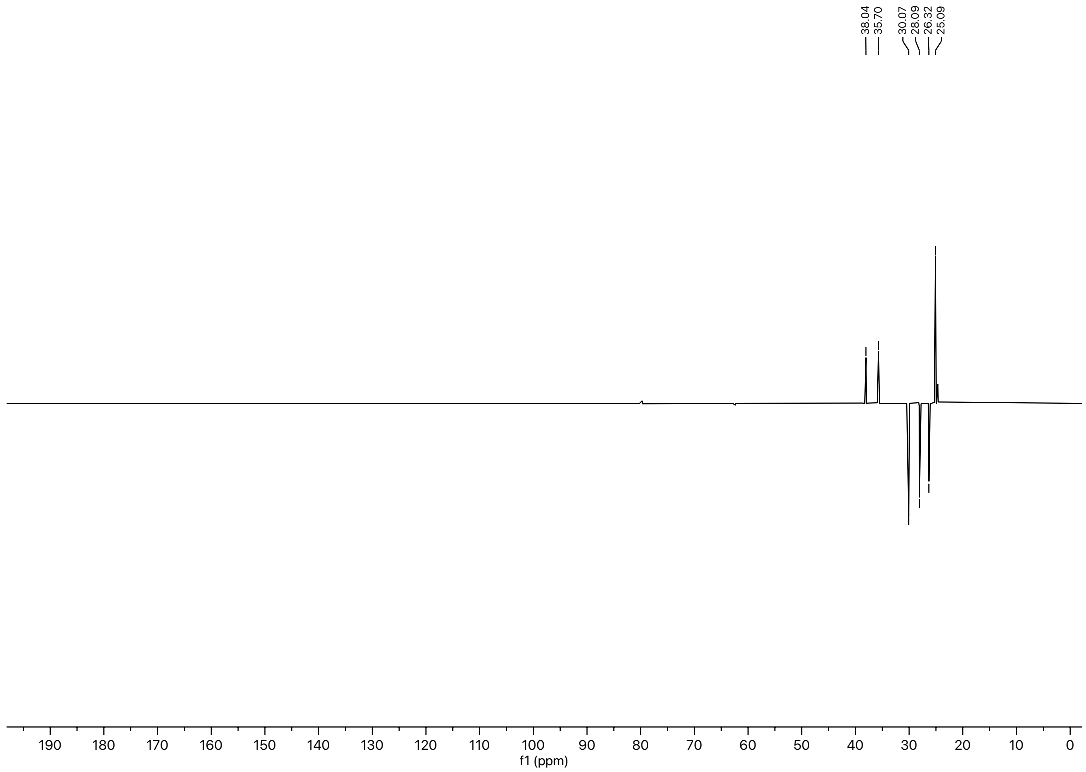

^{11}B NMR (2l)

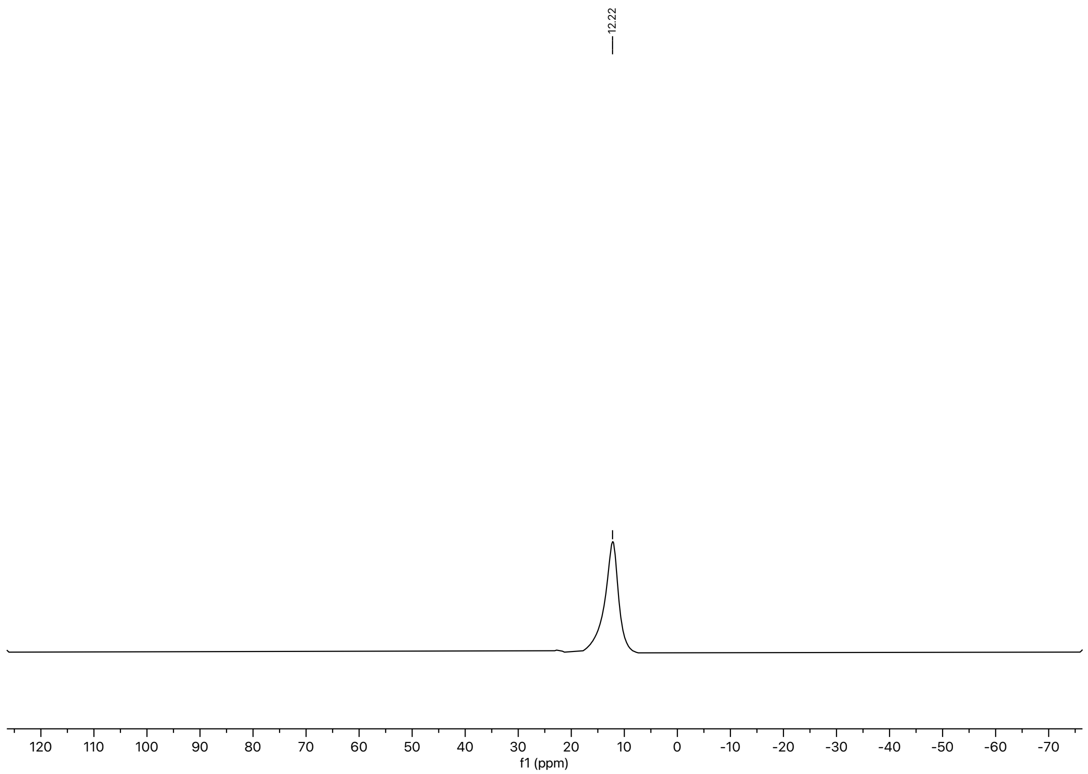

^{1}H NMR (2m)

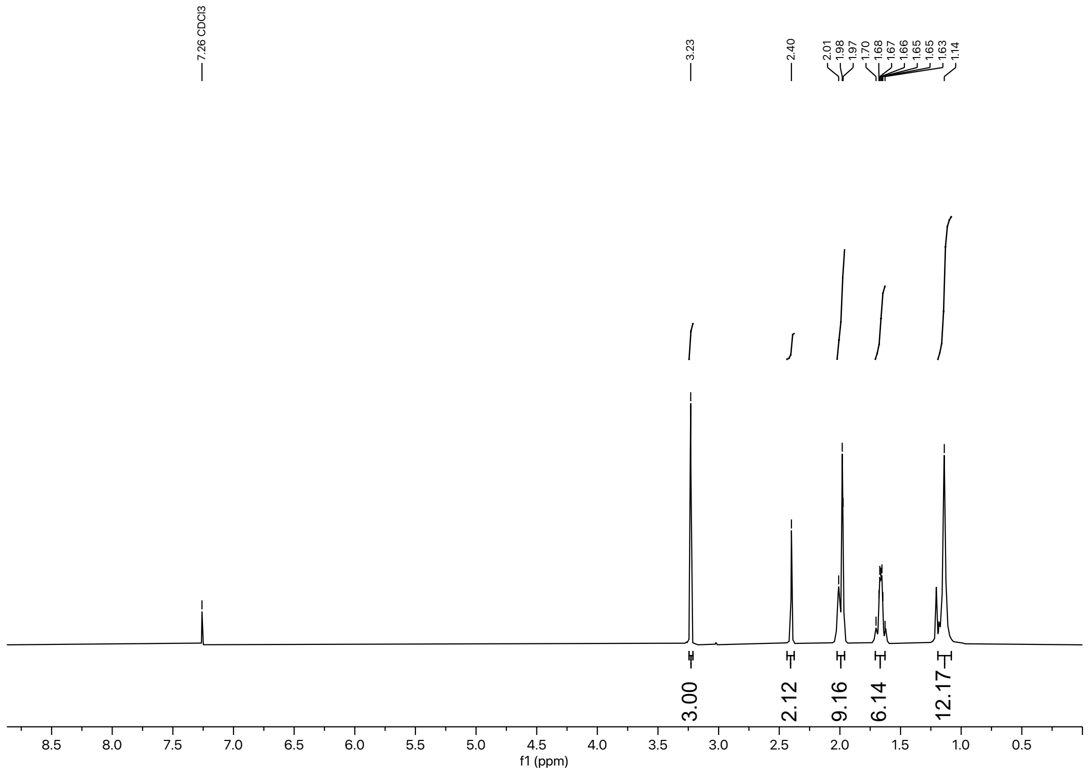

^{13}C NMR (2m)

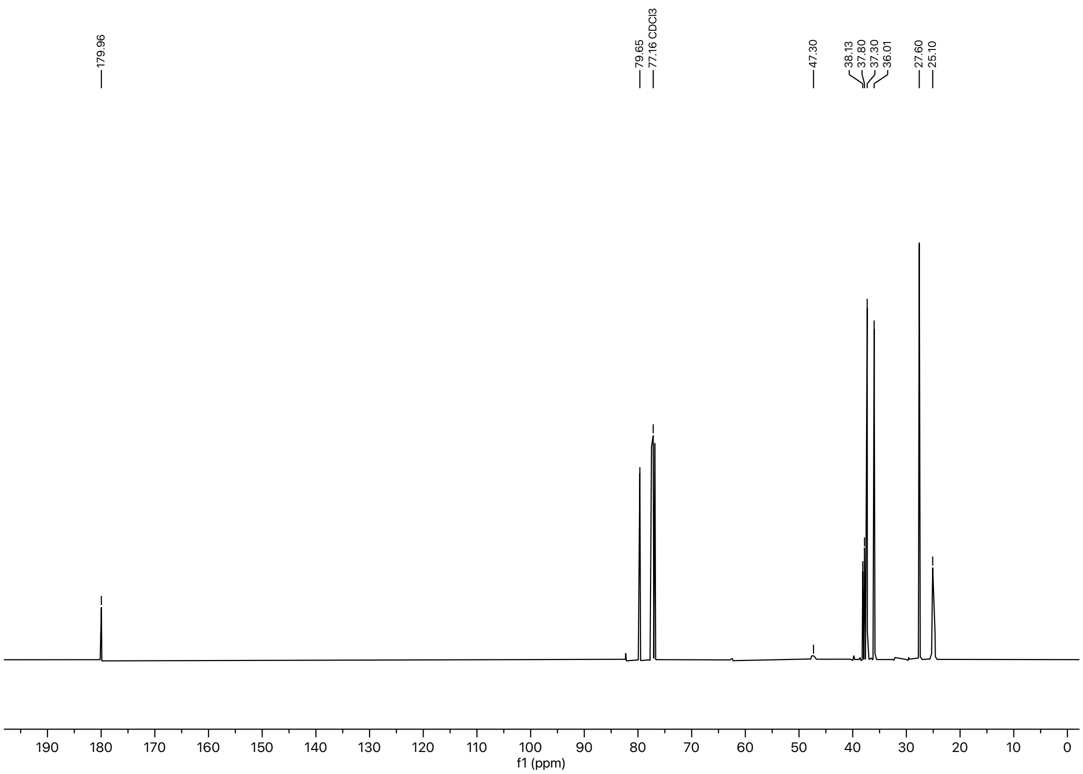

^{13}C DEPT-135 (2m)

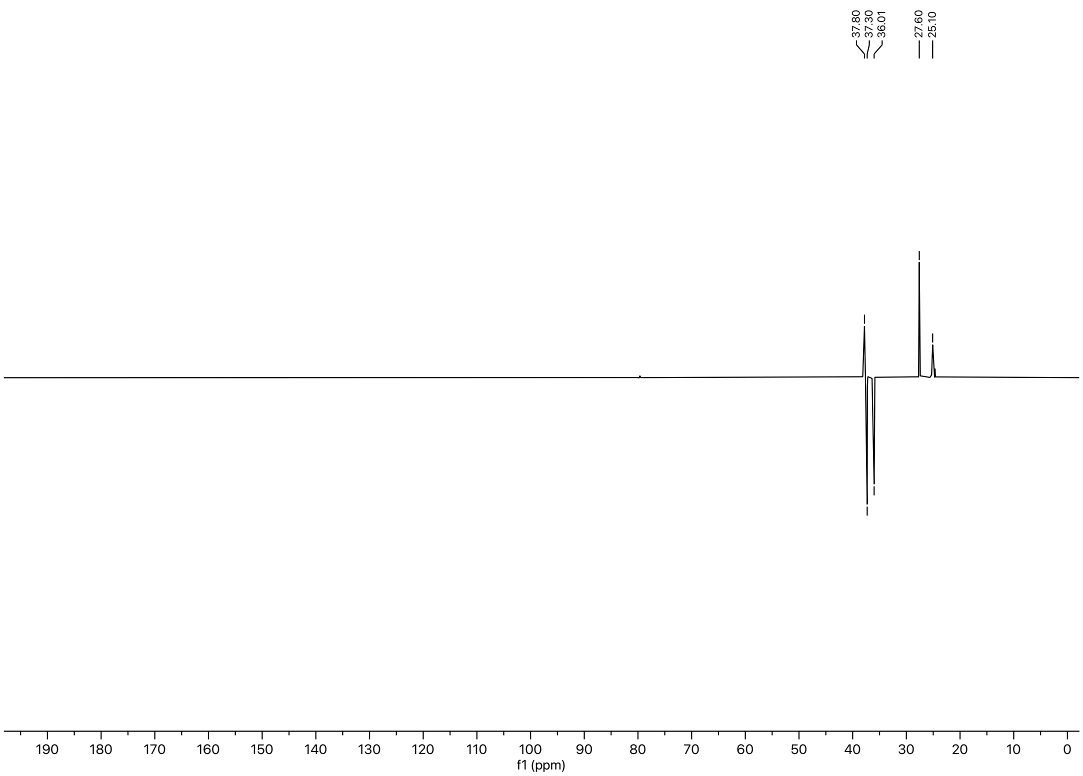

^{11}B NMR (2m)

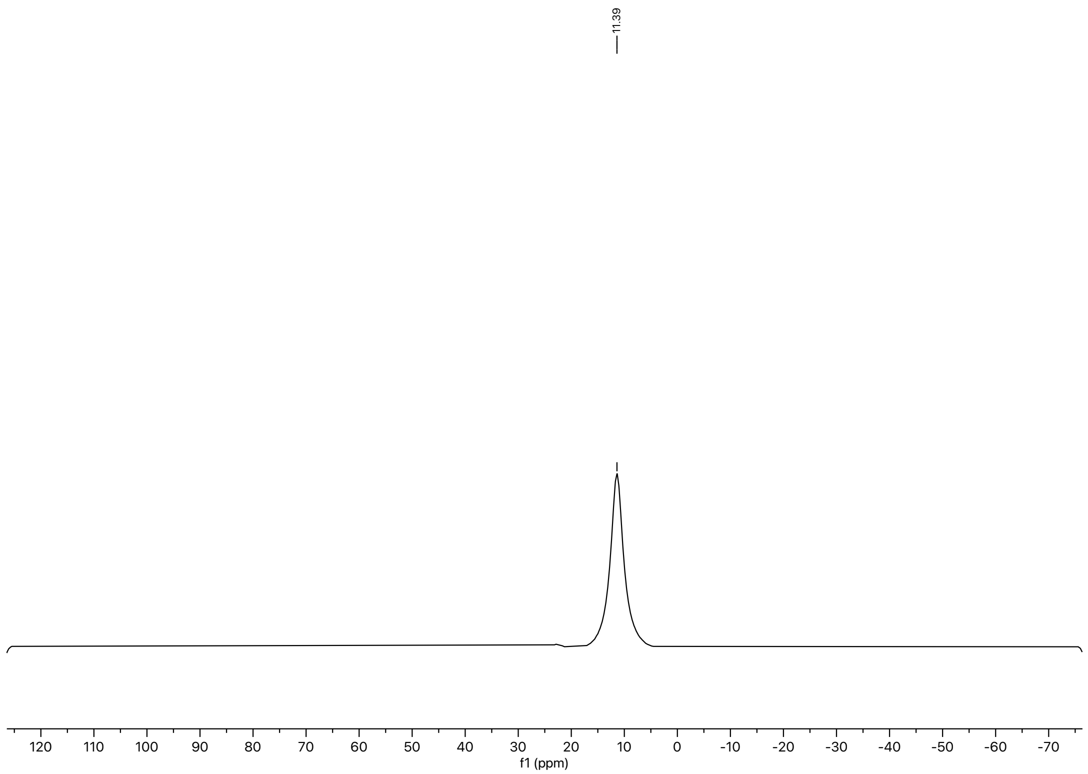

^{1}H NMR (2n)

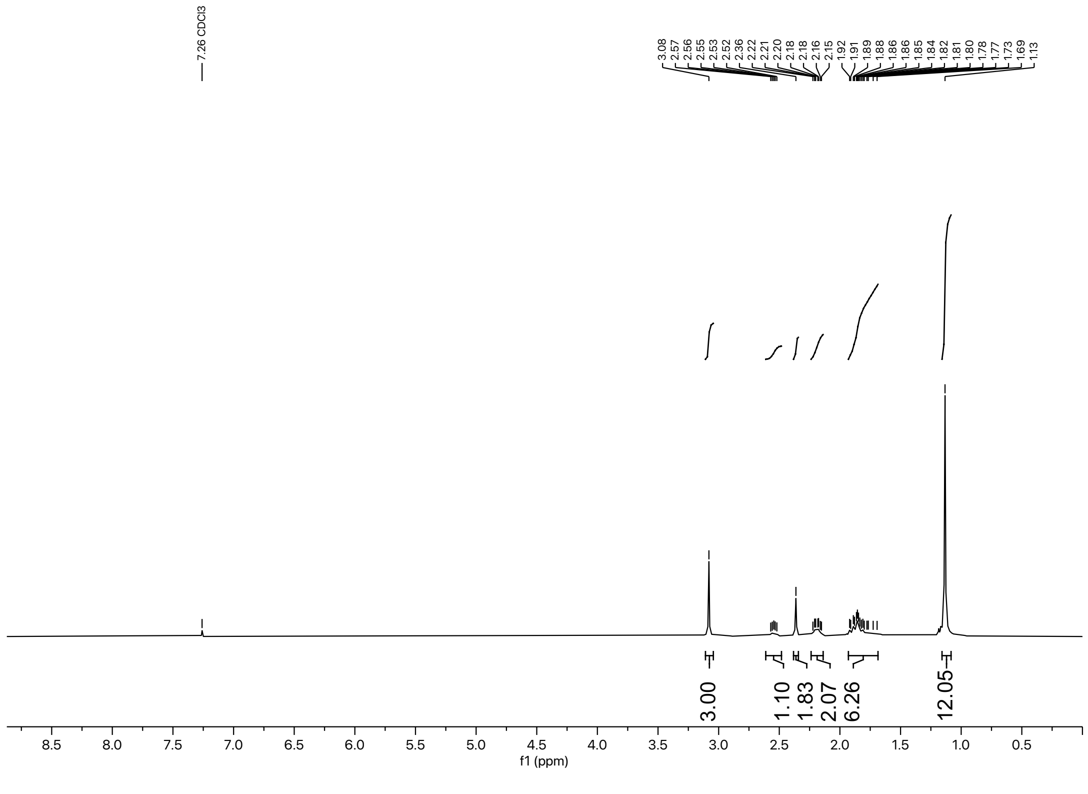

^{13}C NMR (2n)

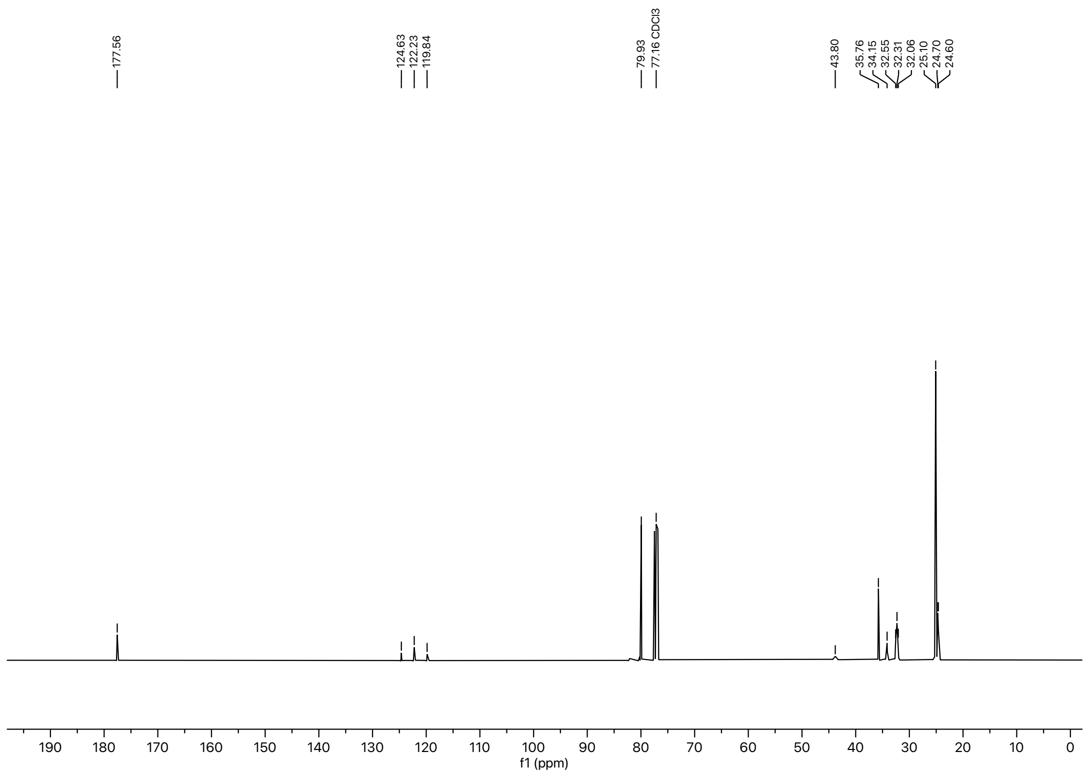

^{13}C DEPT-135 (2n)

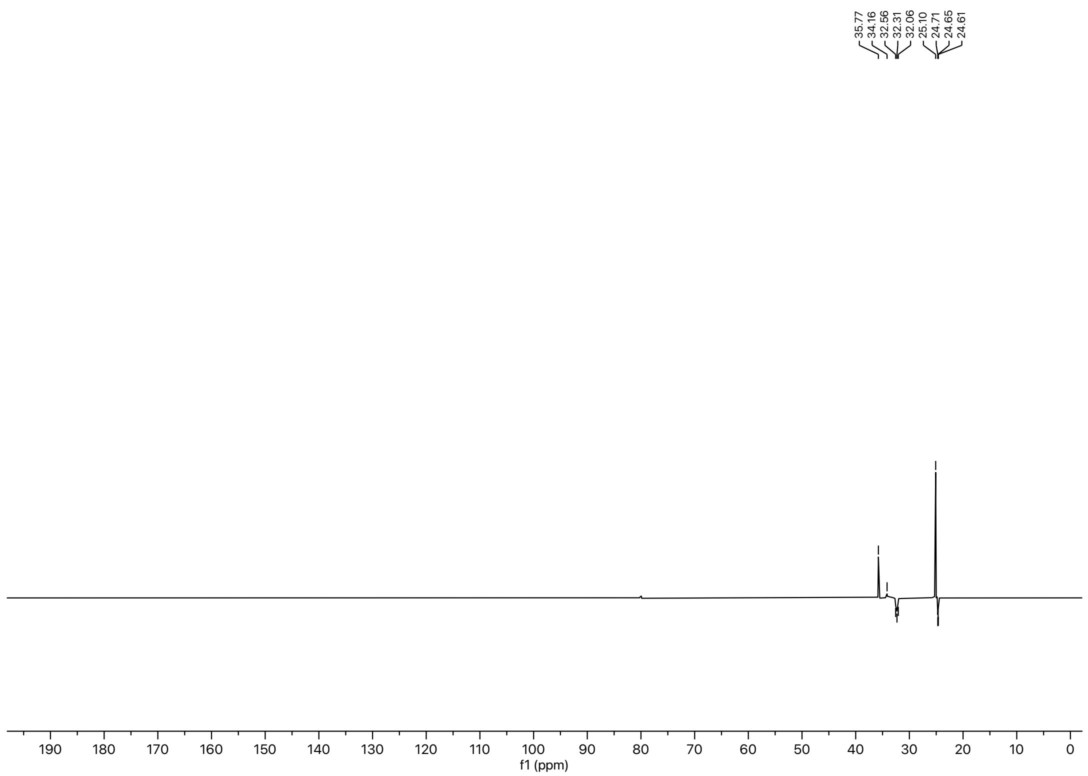

^{19}F NMR (2n)

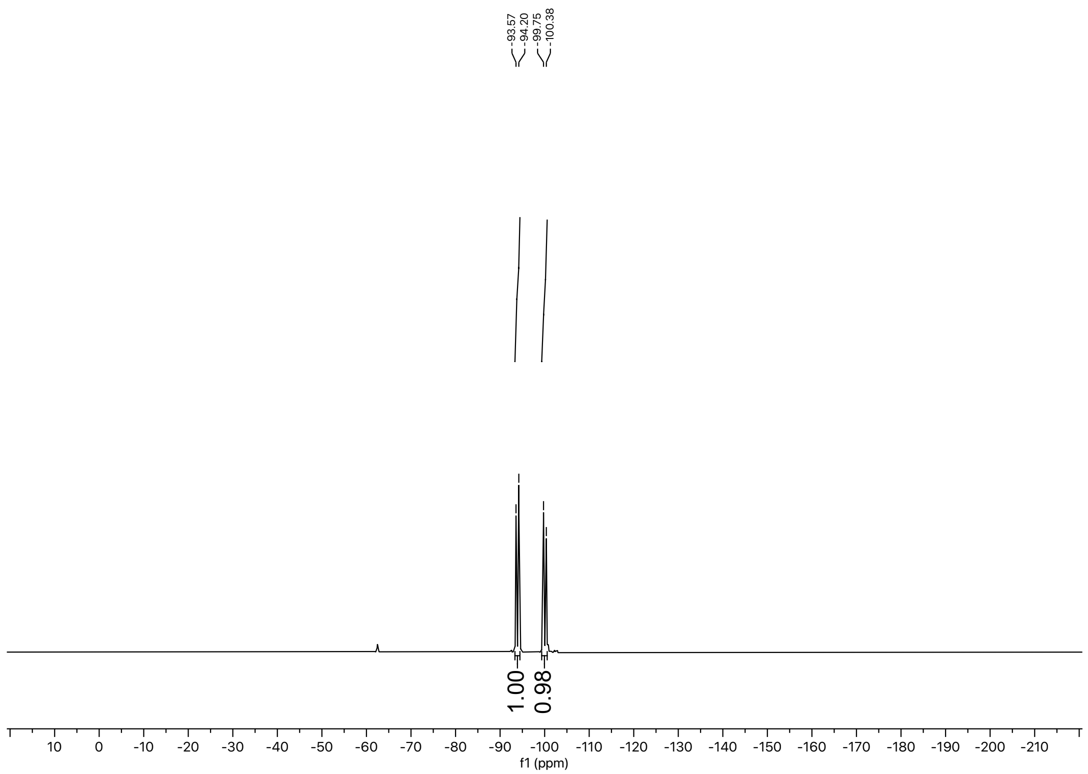

^{11}B NMR (2n)

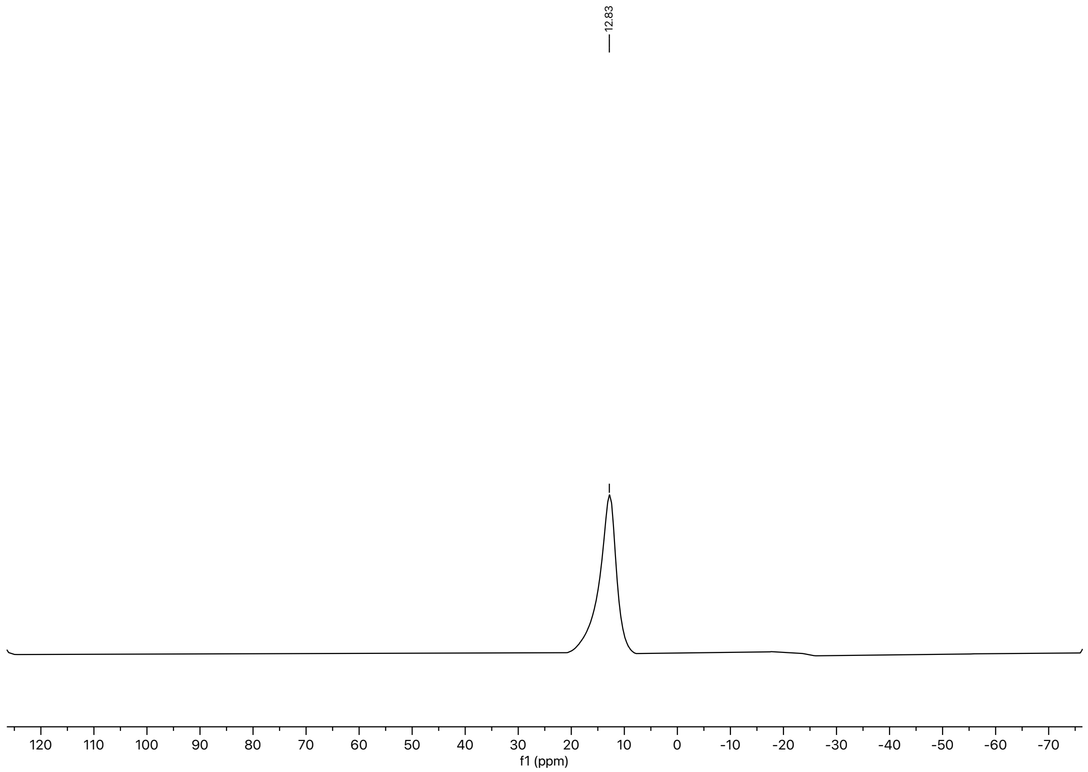

^1H NMR (2o)

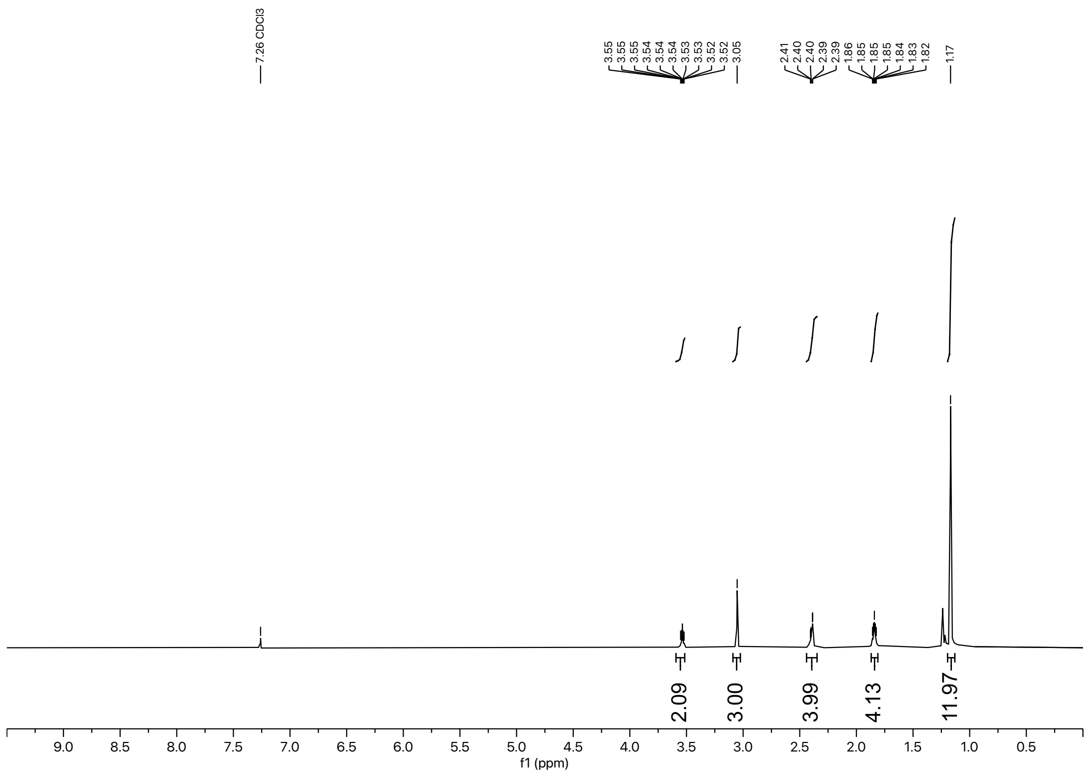

^{13}C NMR (2o)

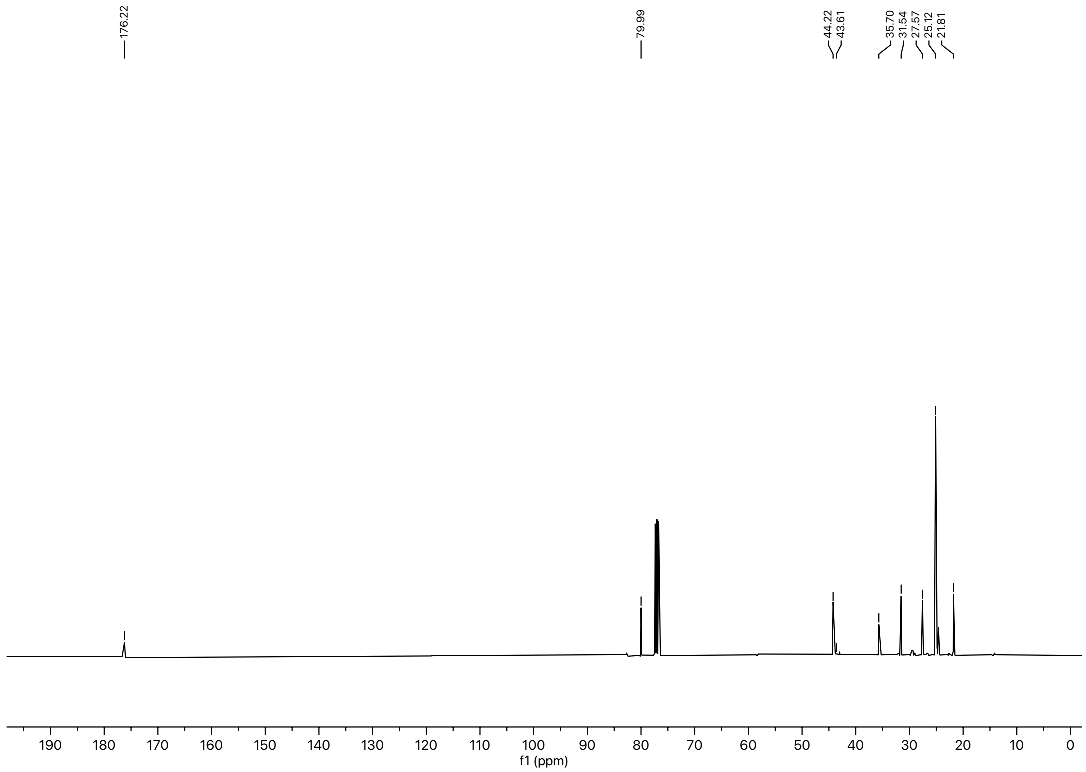

^{13}C DEPT-135 (2o)

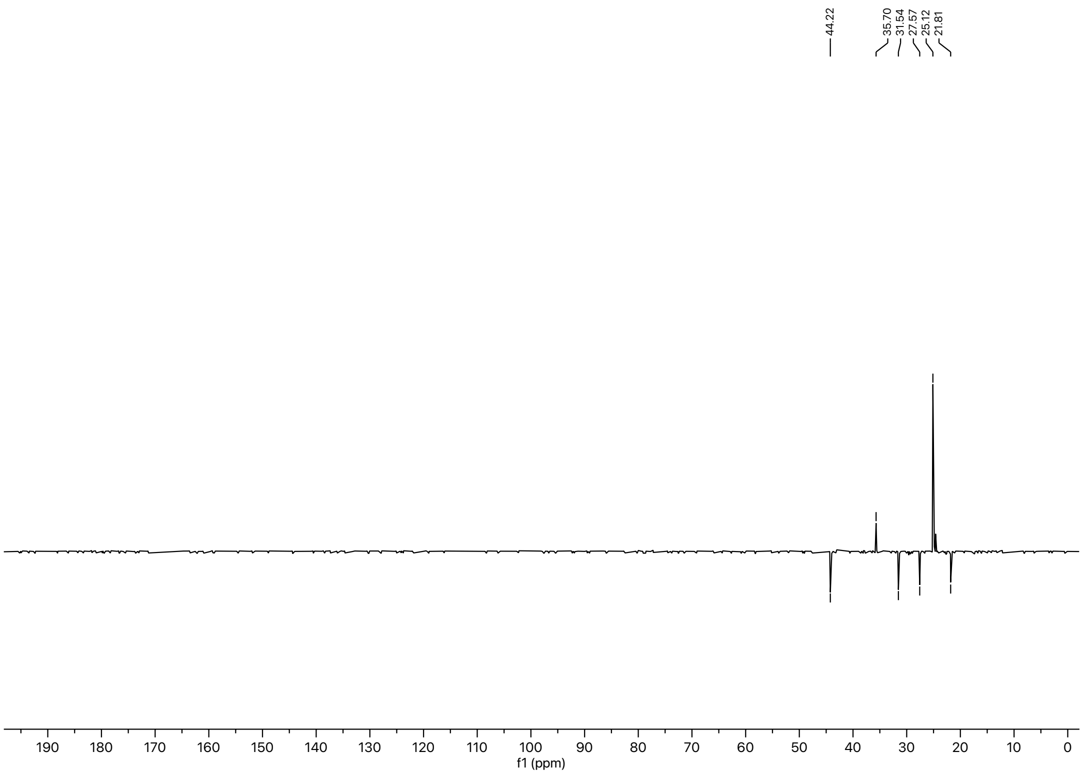

^{11}B NMR (2o)

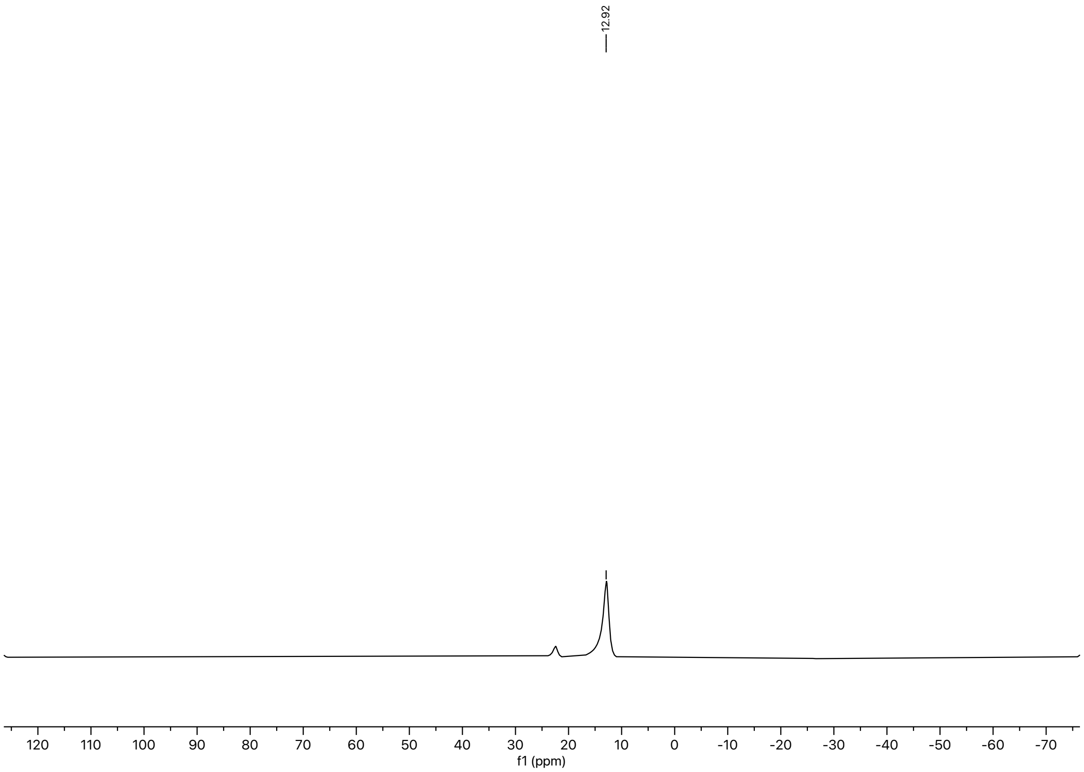

^{1}H NMR (2p)

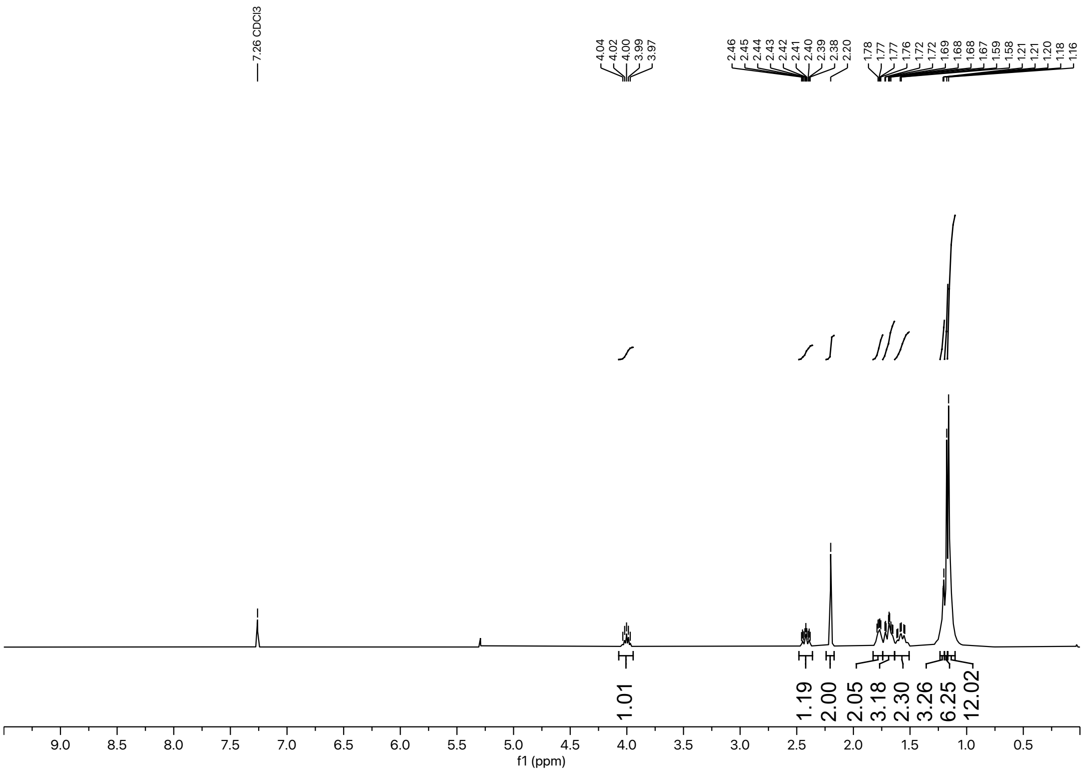

^{13}C NMR (2p)

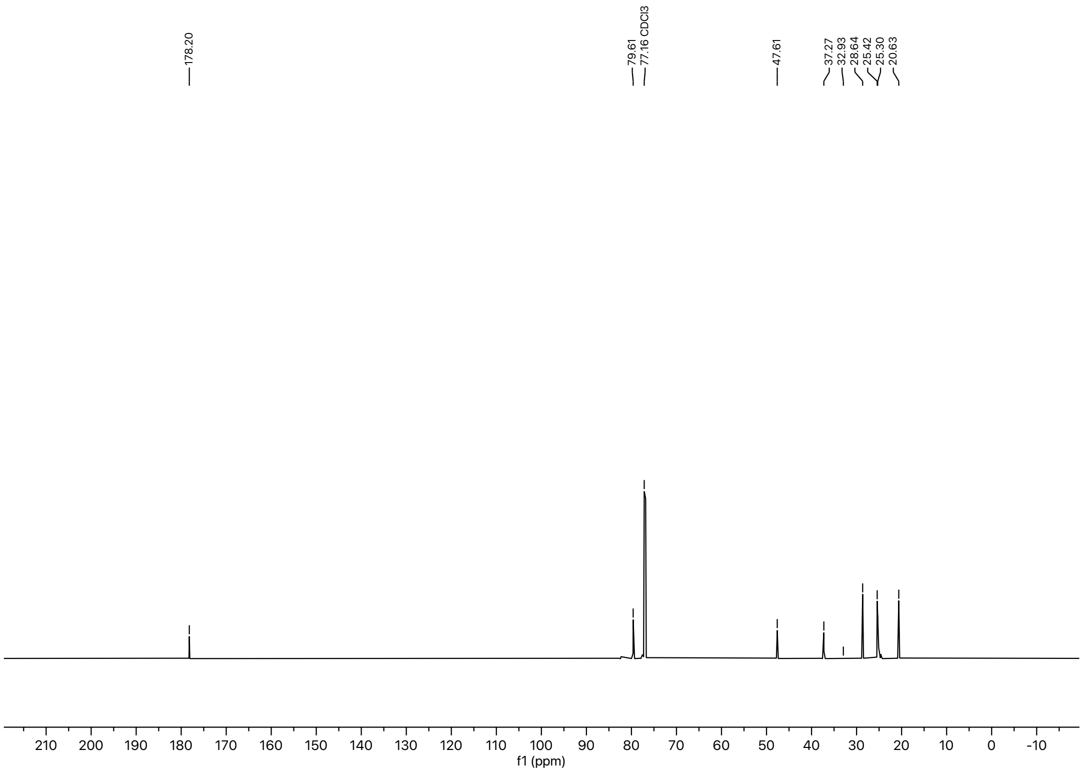

^{13}C DEPT-135 (2p)

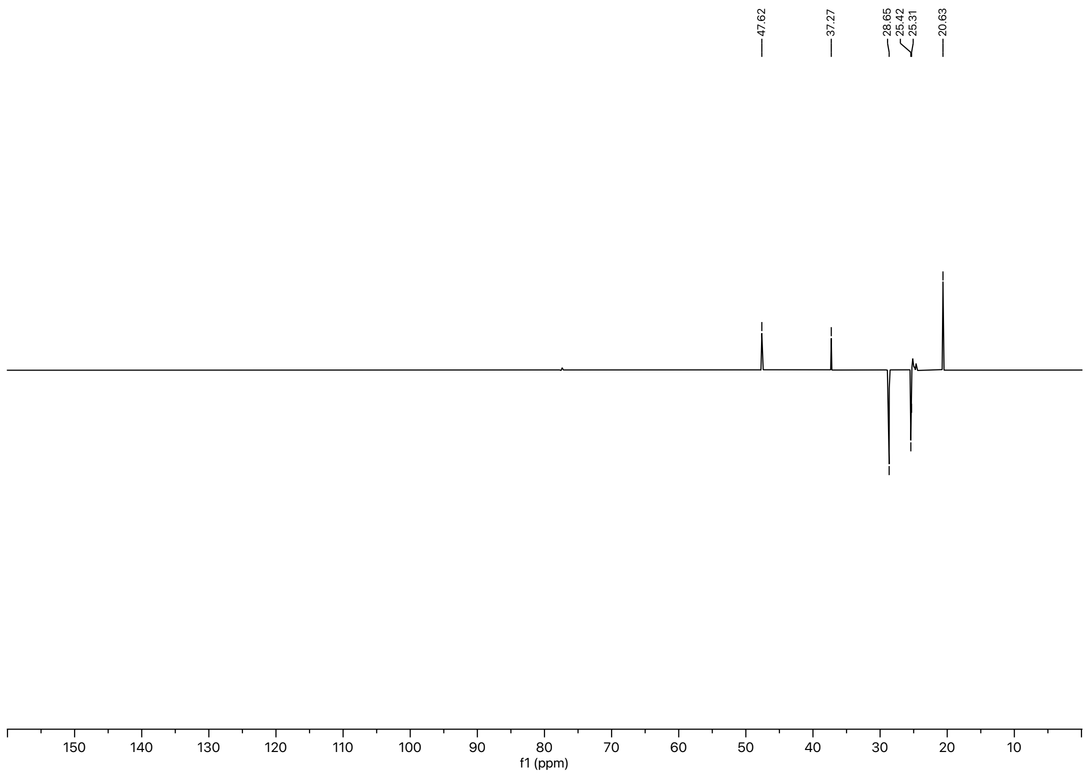

^{11}B NMR (2p)

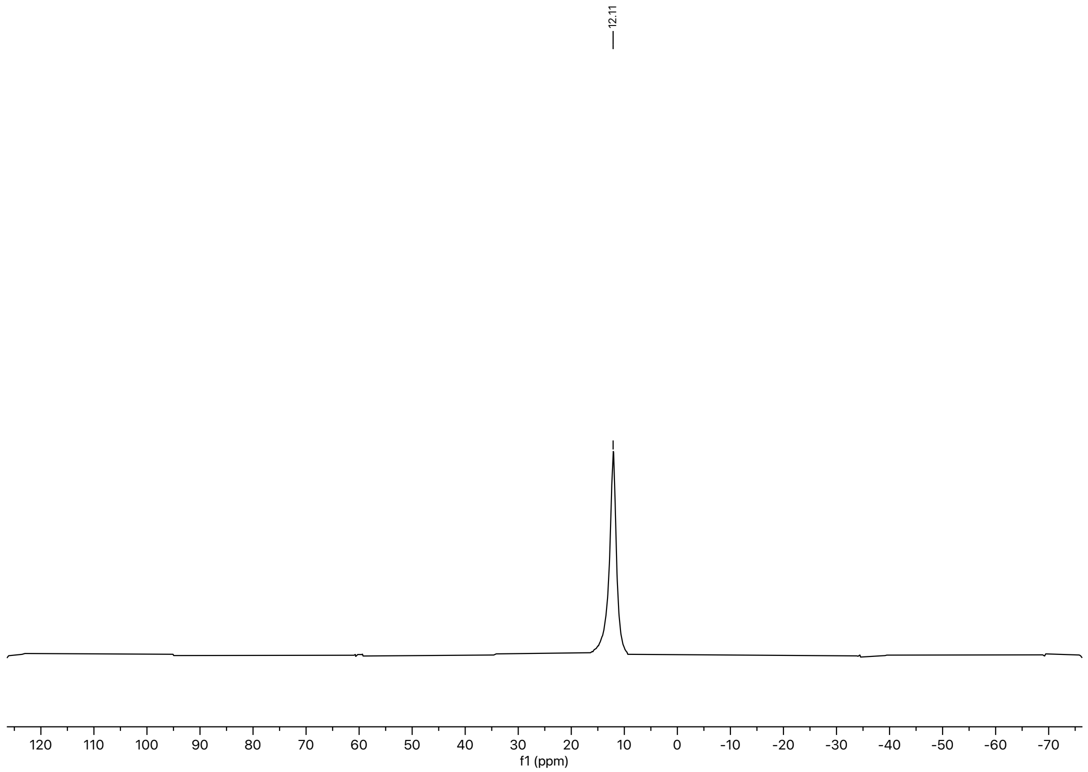

^1H NMR (2q)

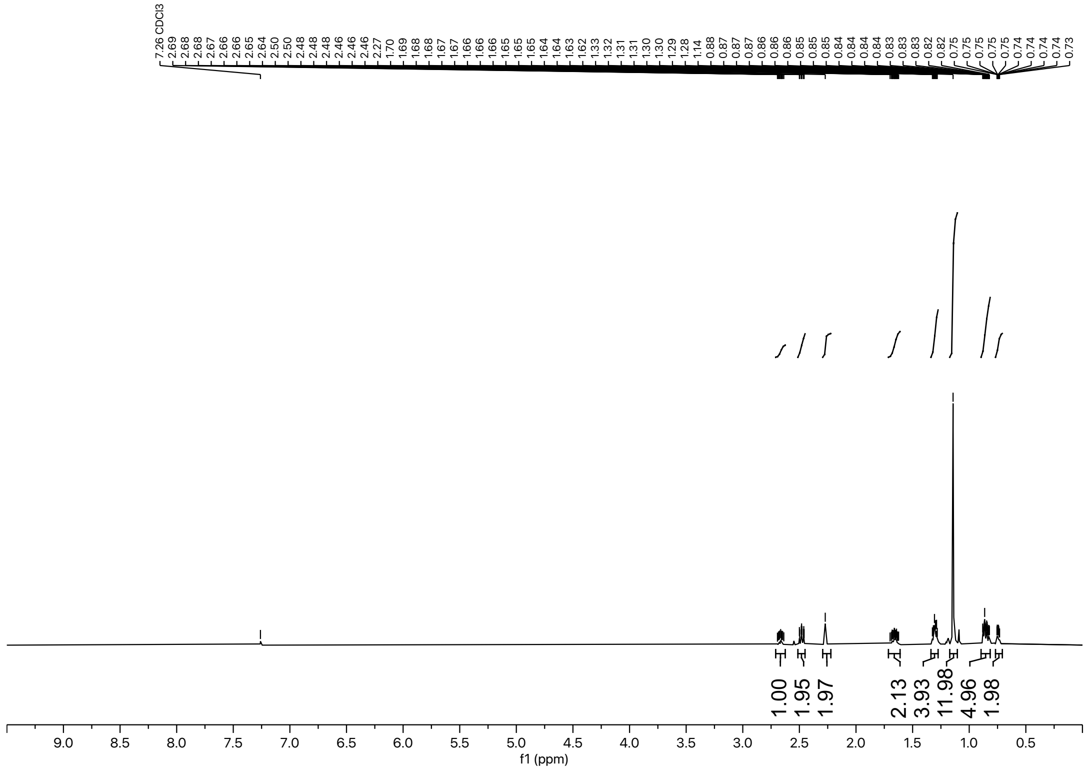

^{13}C NMR (2q)

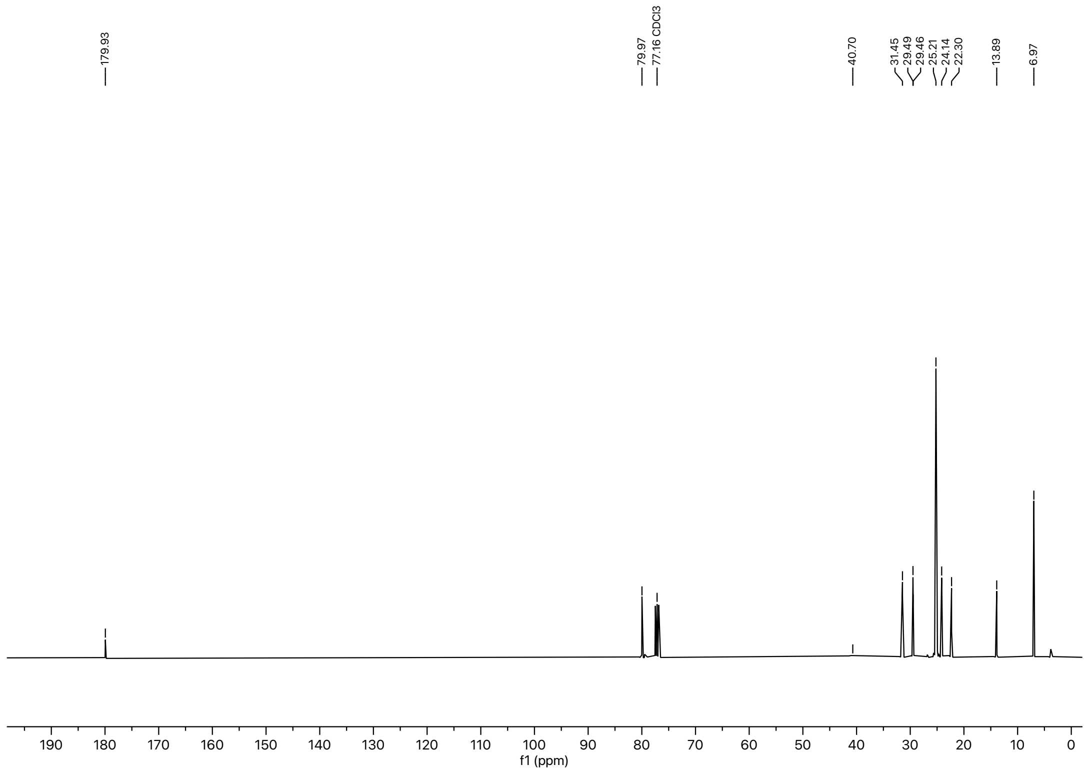

^{13}C DEPT-135 (2q)

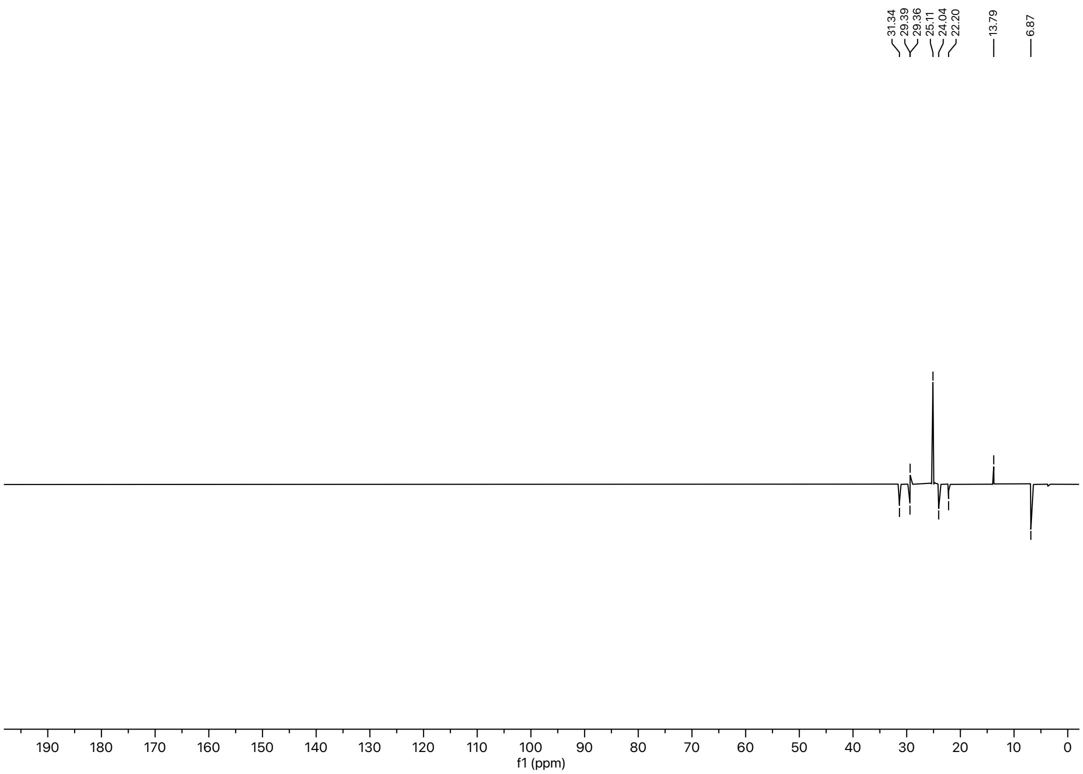

^{11}B NMR (2q)

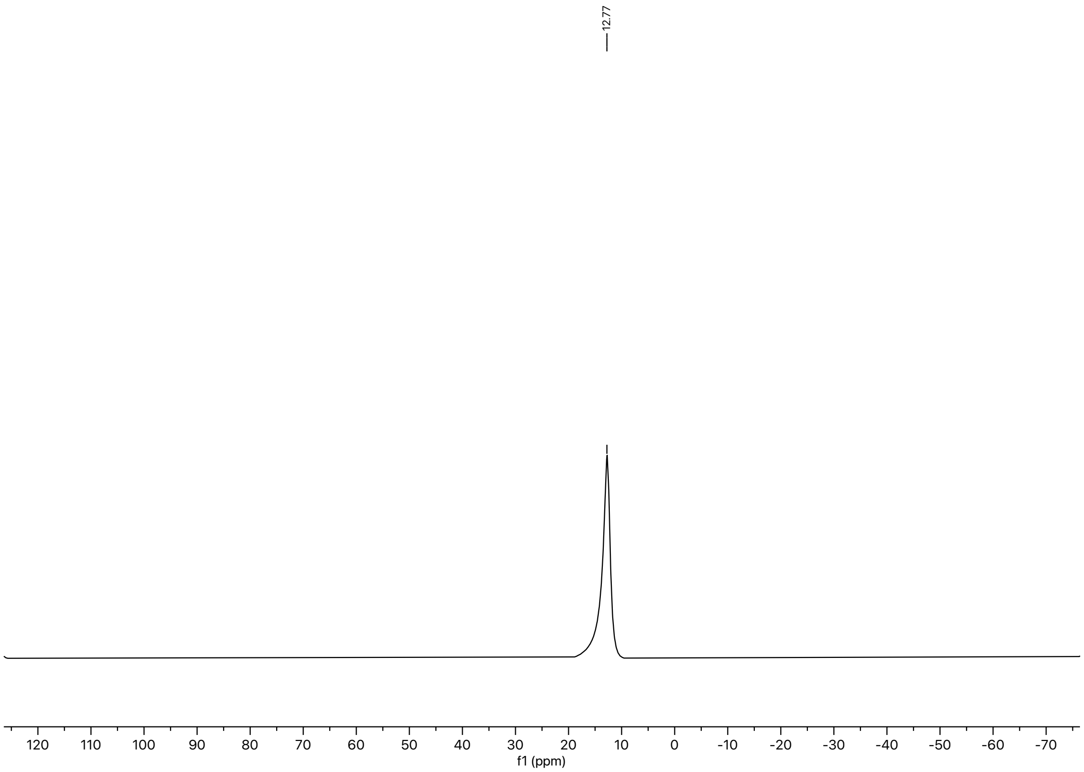

^{1}H NMR (2r)

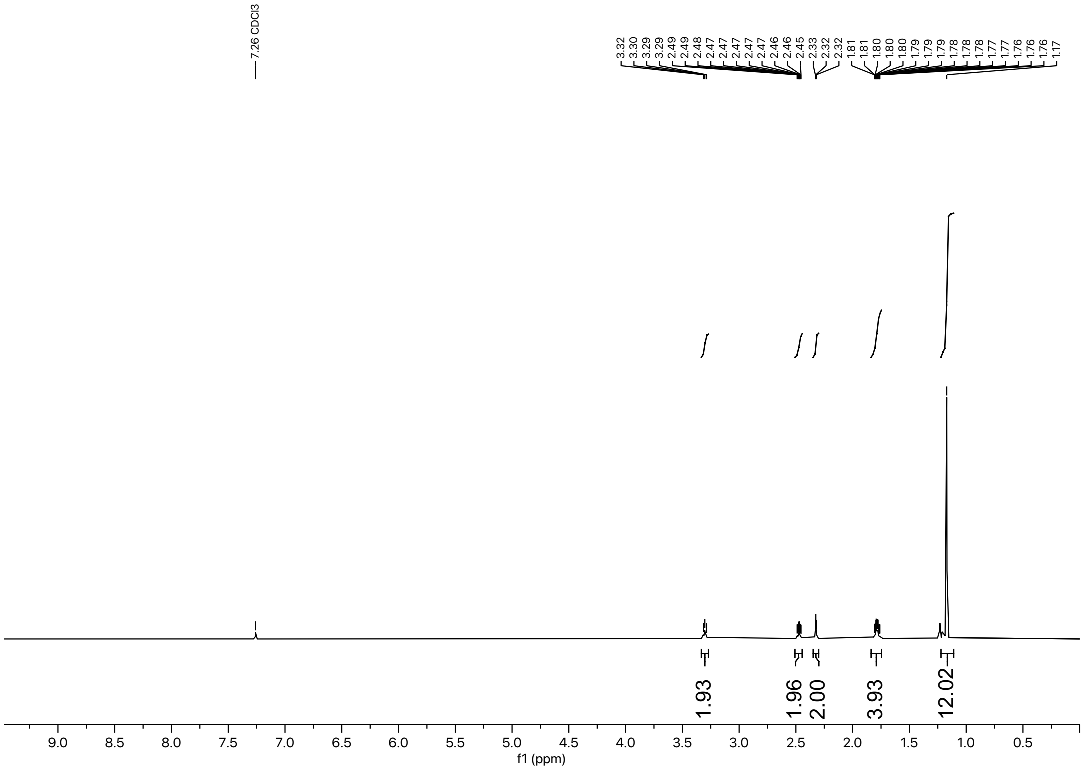

^{13}C NMR (2r)

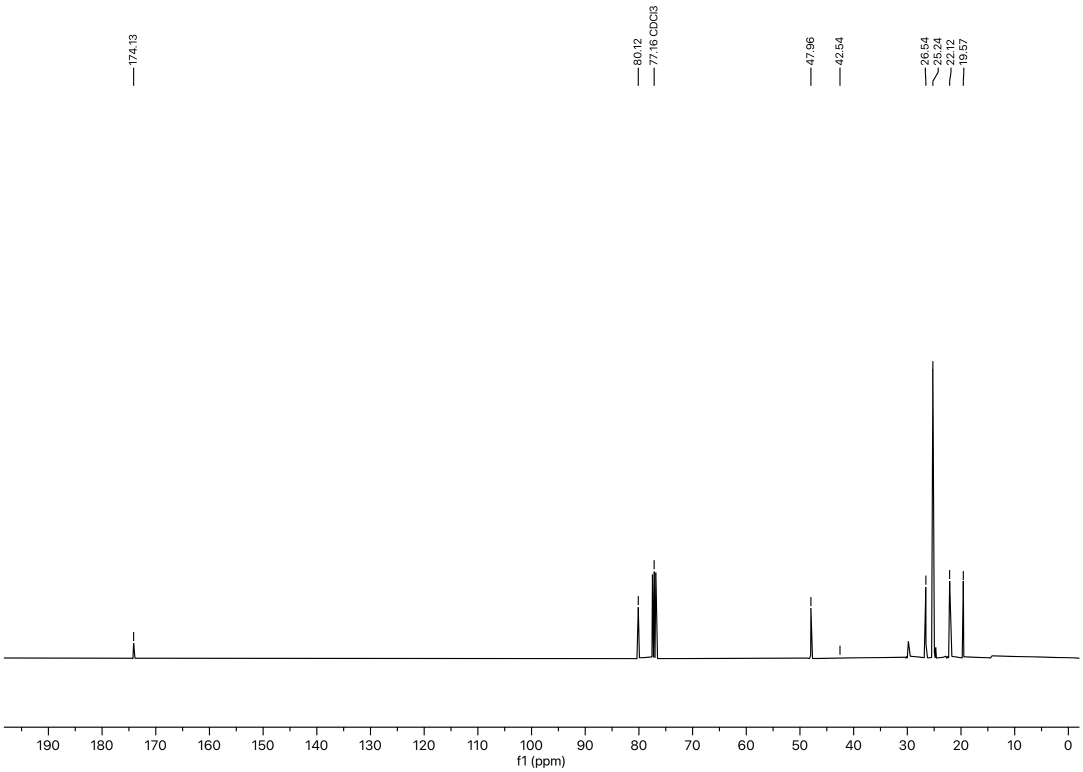

^{13}C DEPT-135 (2r)

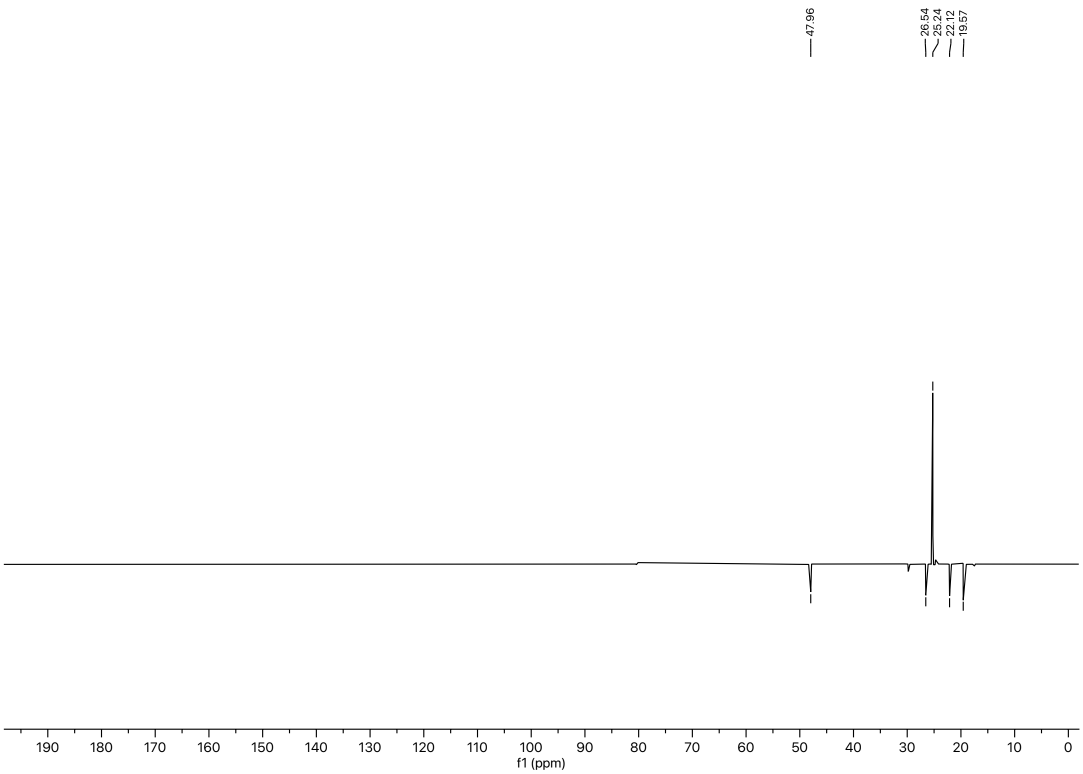

^{11}B NMR (2r)

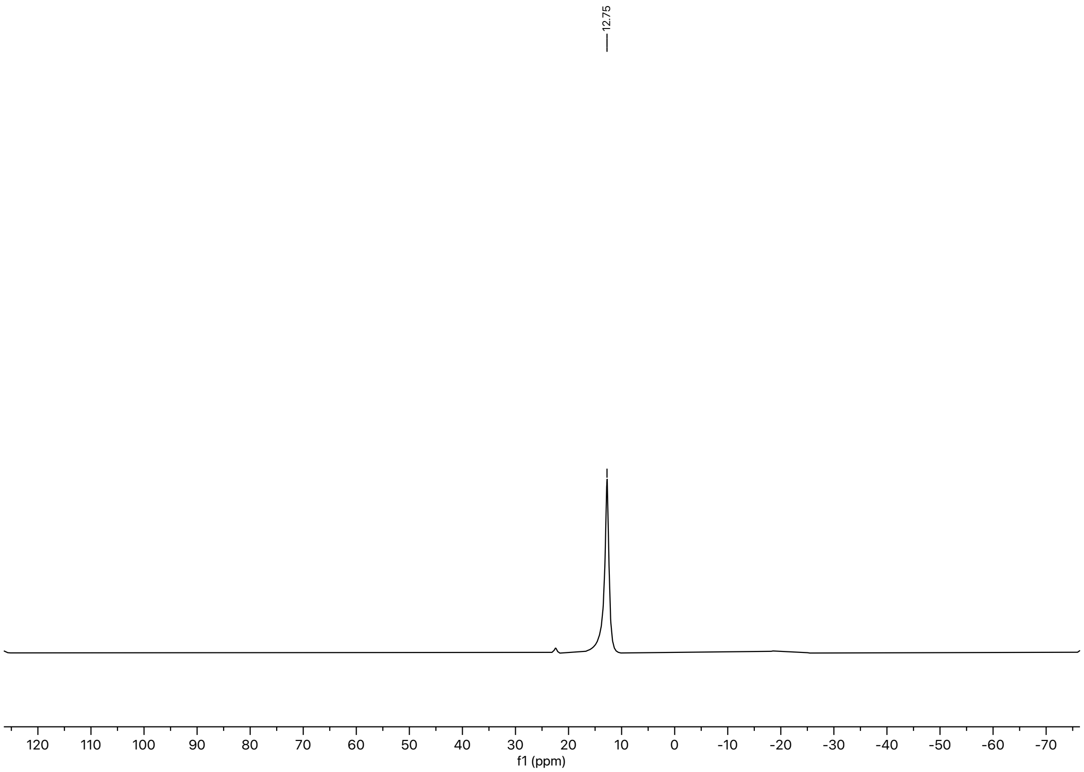

^1H NMR (2s)

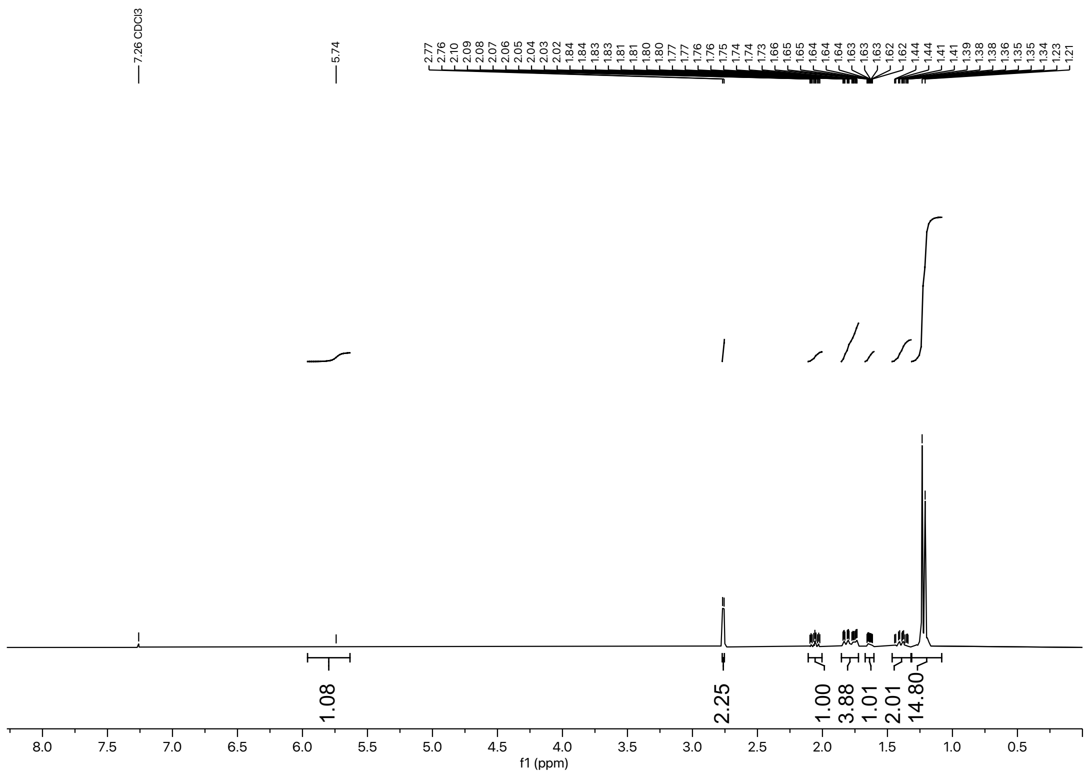

^{13}C NMR (2s)

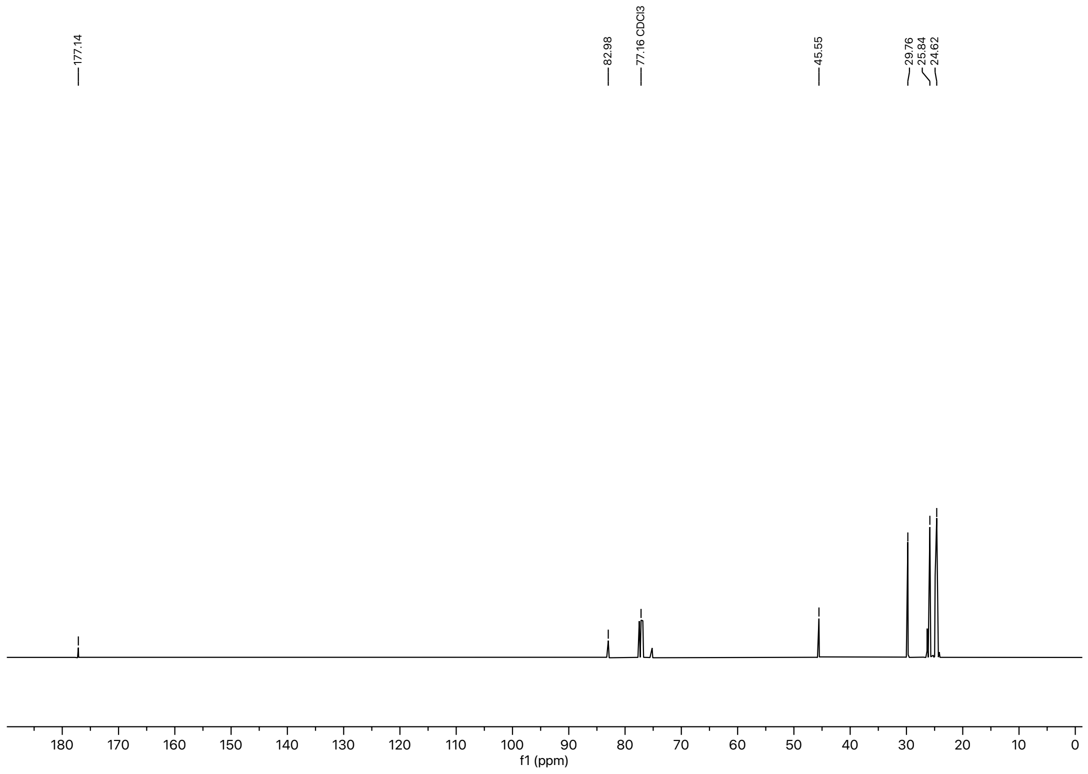

^{13}C DEPT-135 (2s)

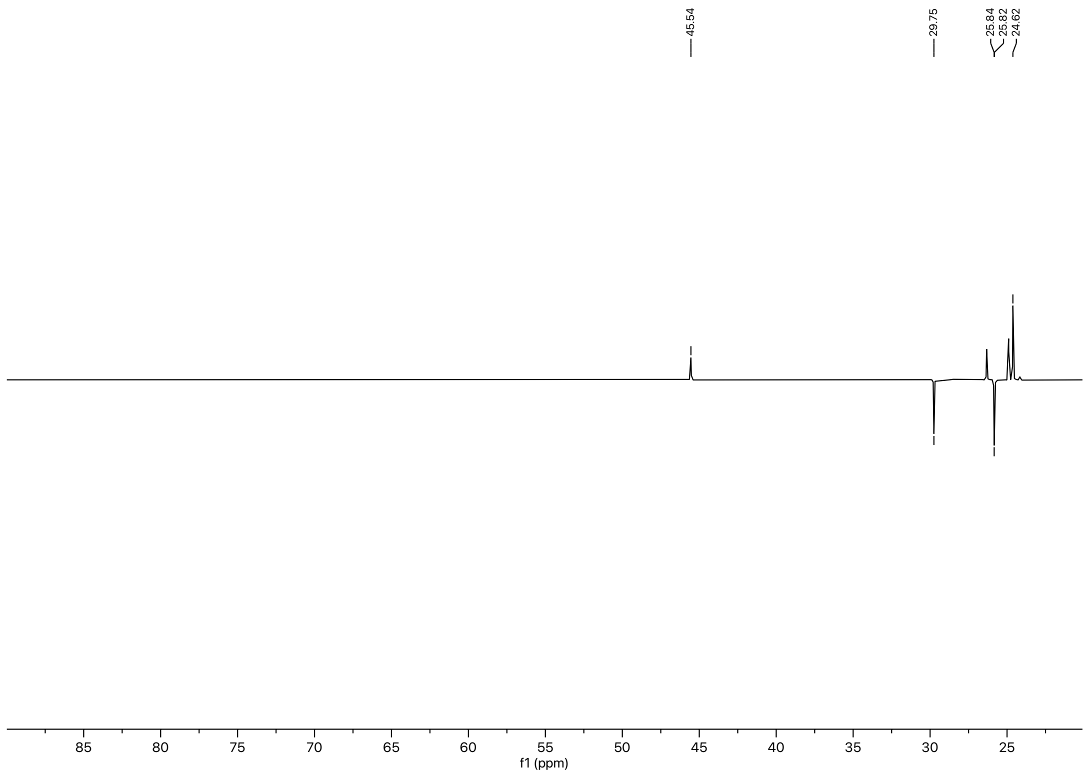

^{11}B NMR (2s)

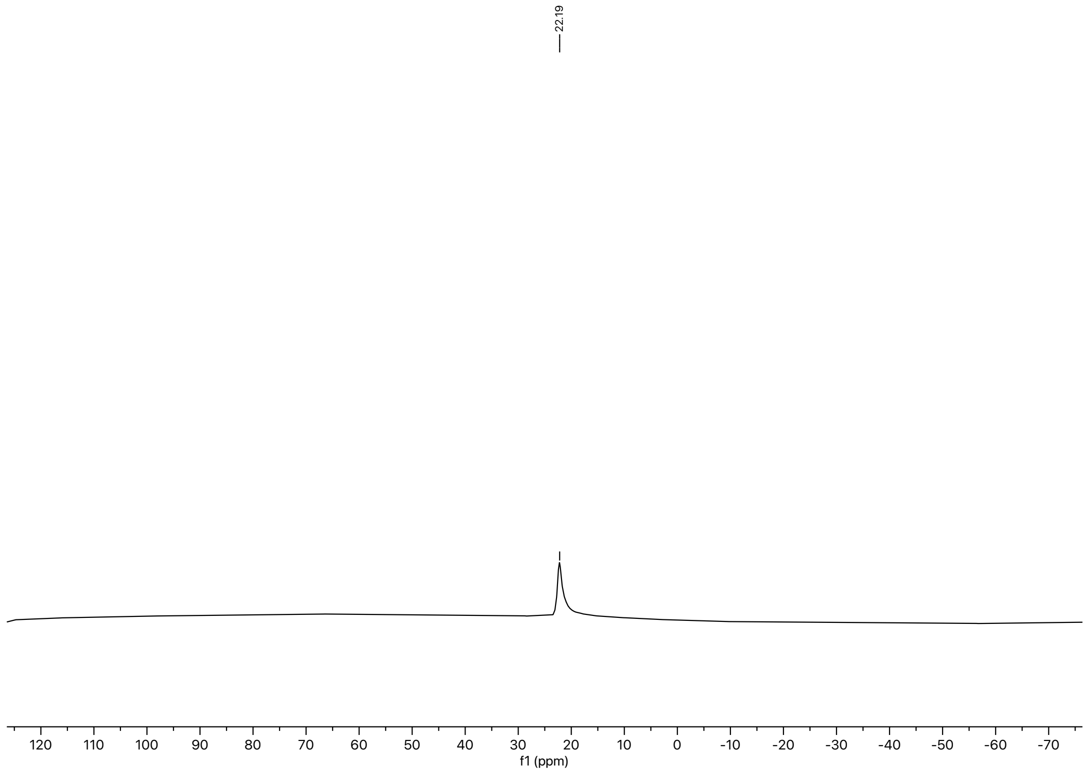

^{1}H NMR (2t)

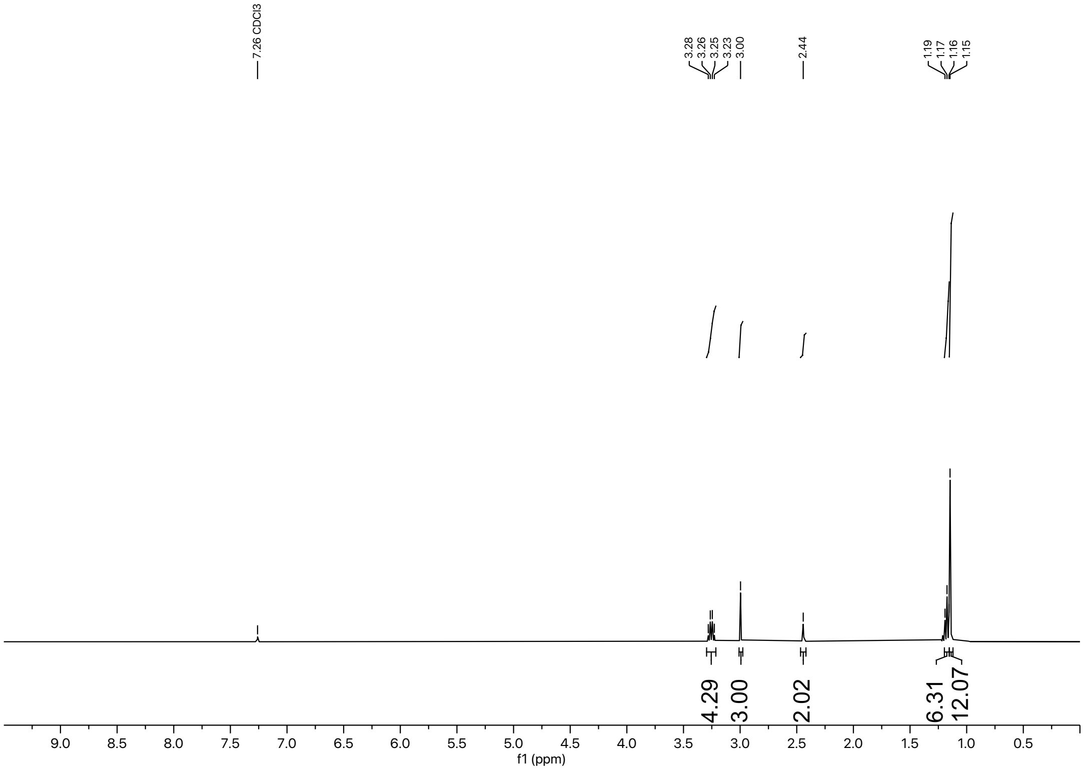

^{13}C NMR (2t)

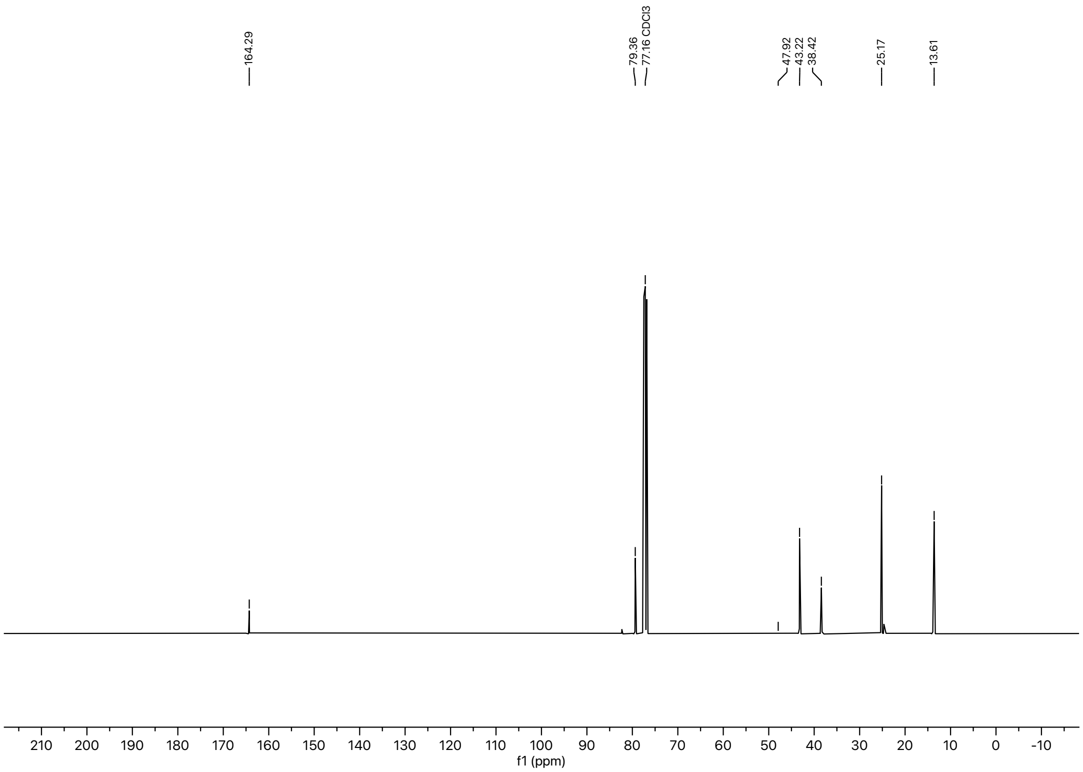

^{13}C DEPT-135 (2t)

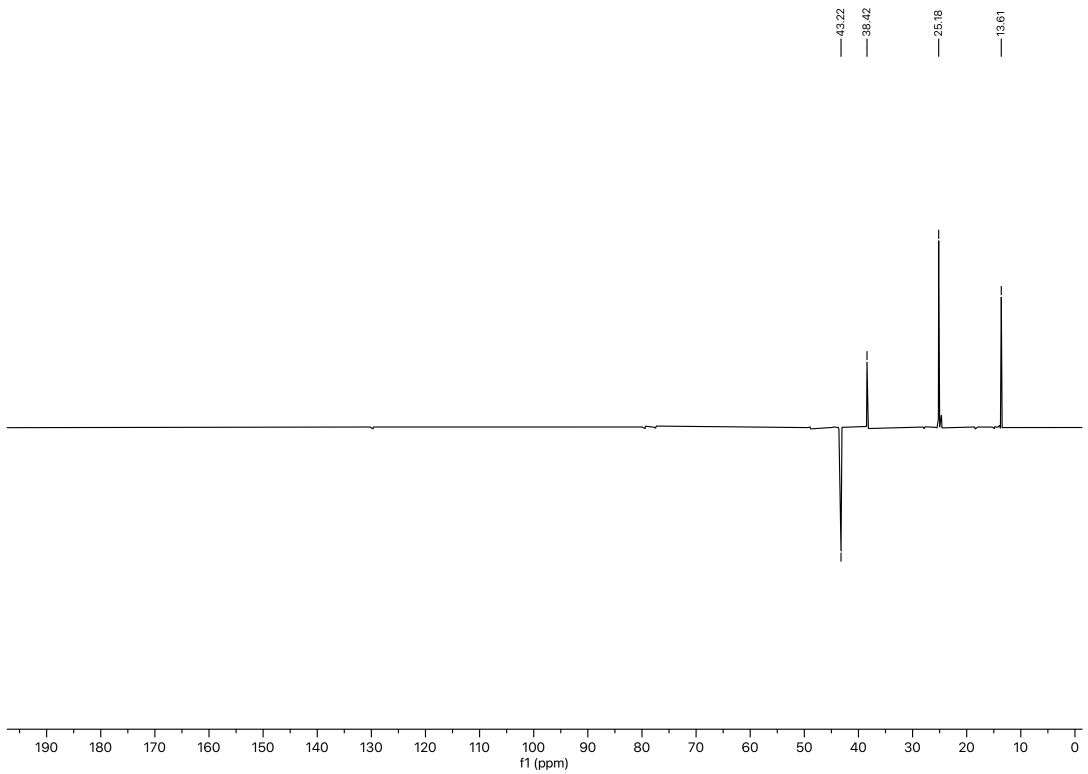

^{11}B NMR (2t)

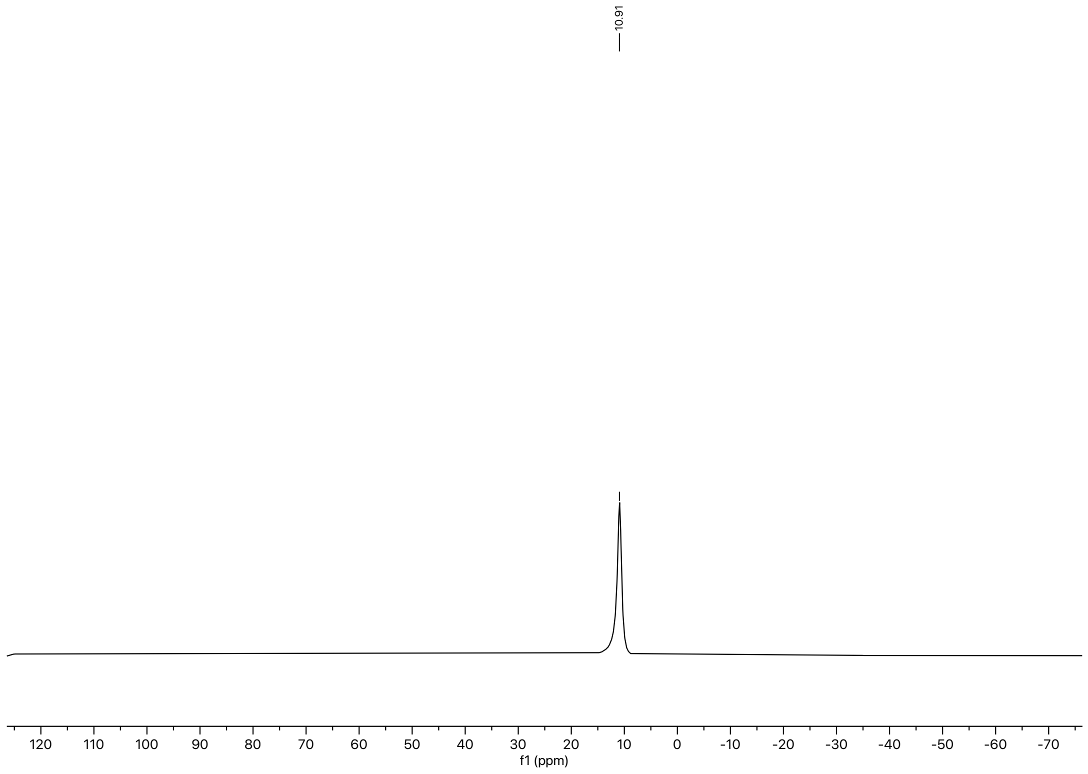

^{1}H NMR (2x)

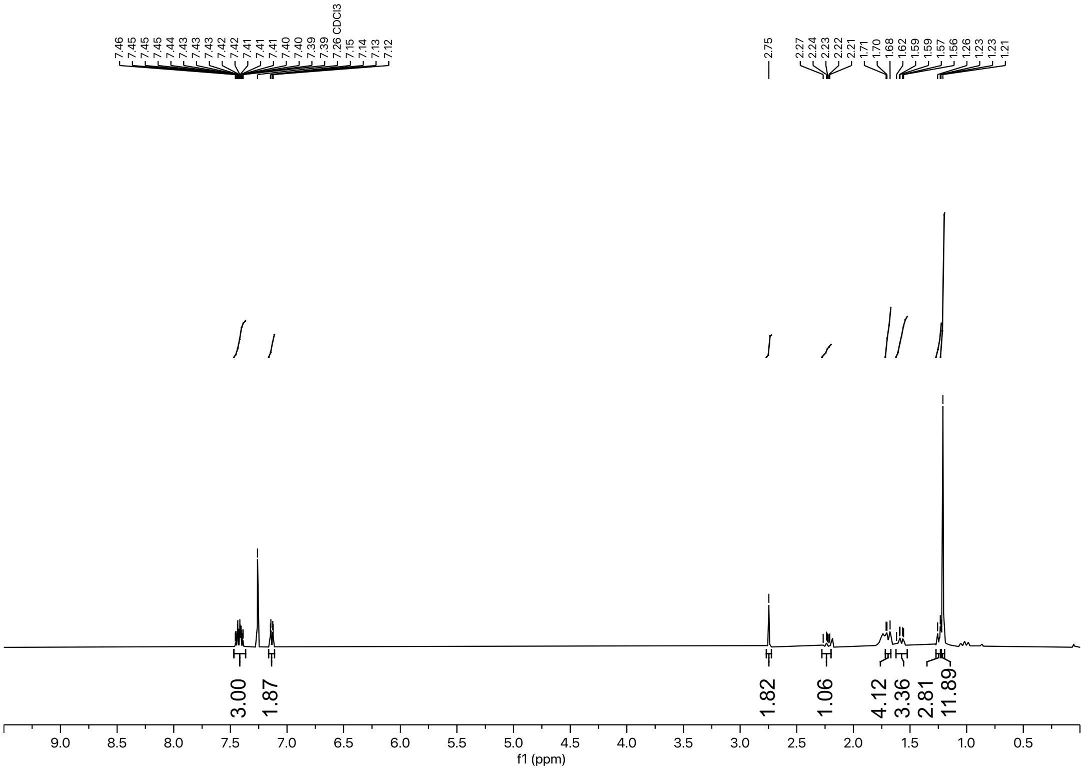

^{13}C NMR (2x)

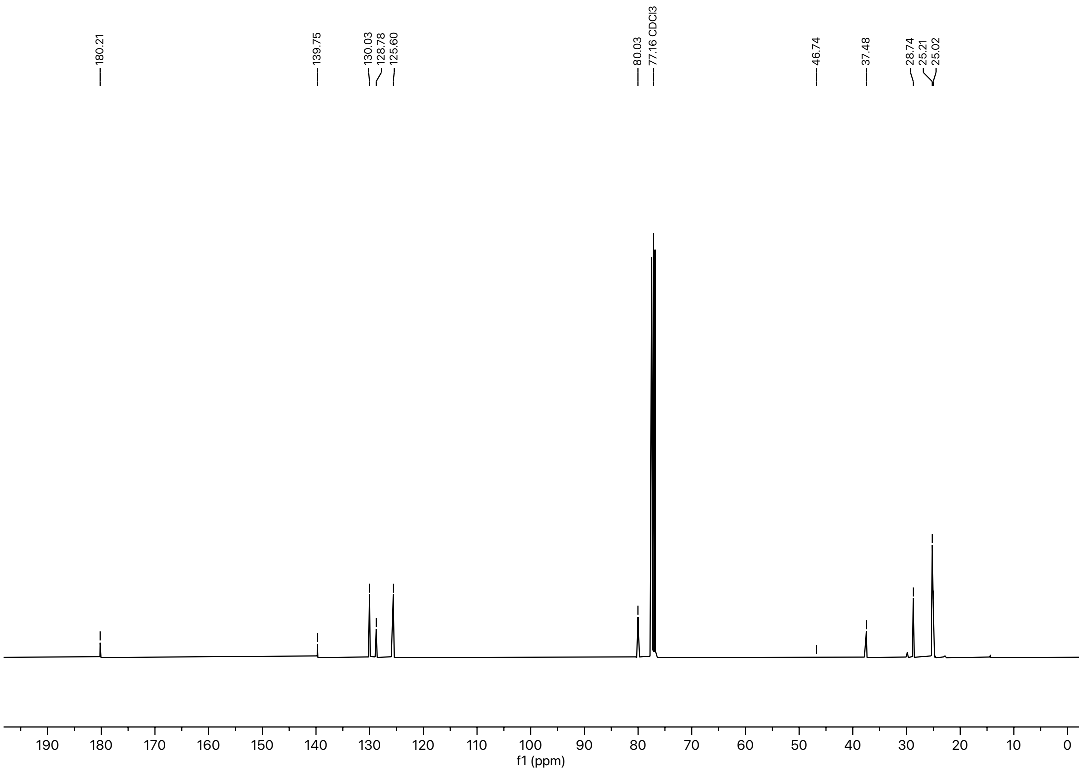

^{13}C DEPT-135 (2x)

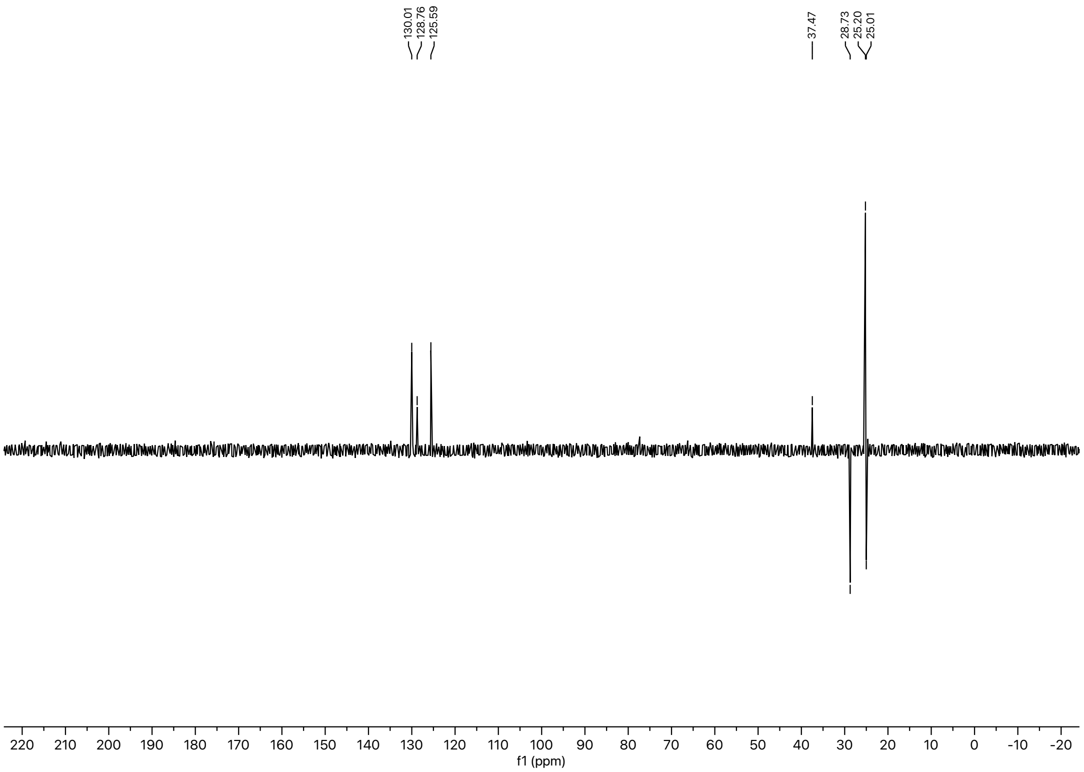

^{11}B NMR (2x)

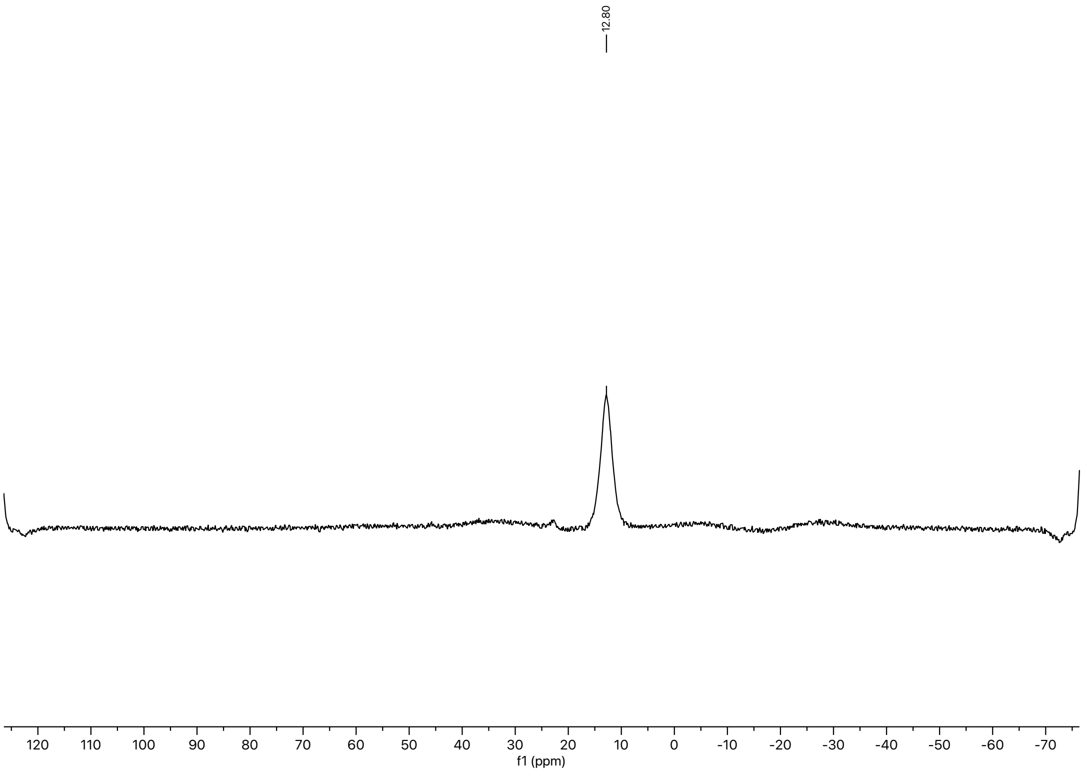

^1H NMR (2y)

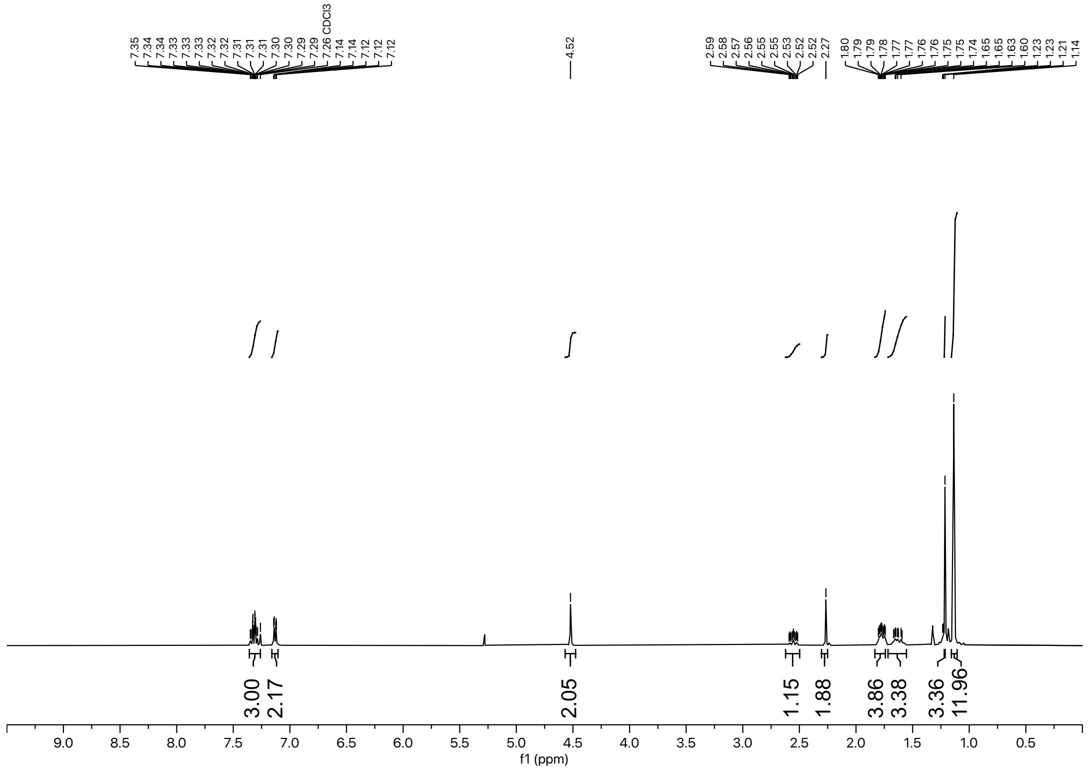

^{13}C NMR (2y)

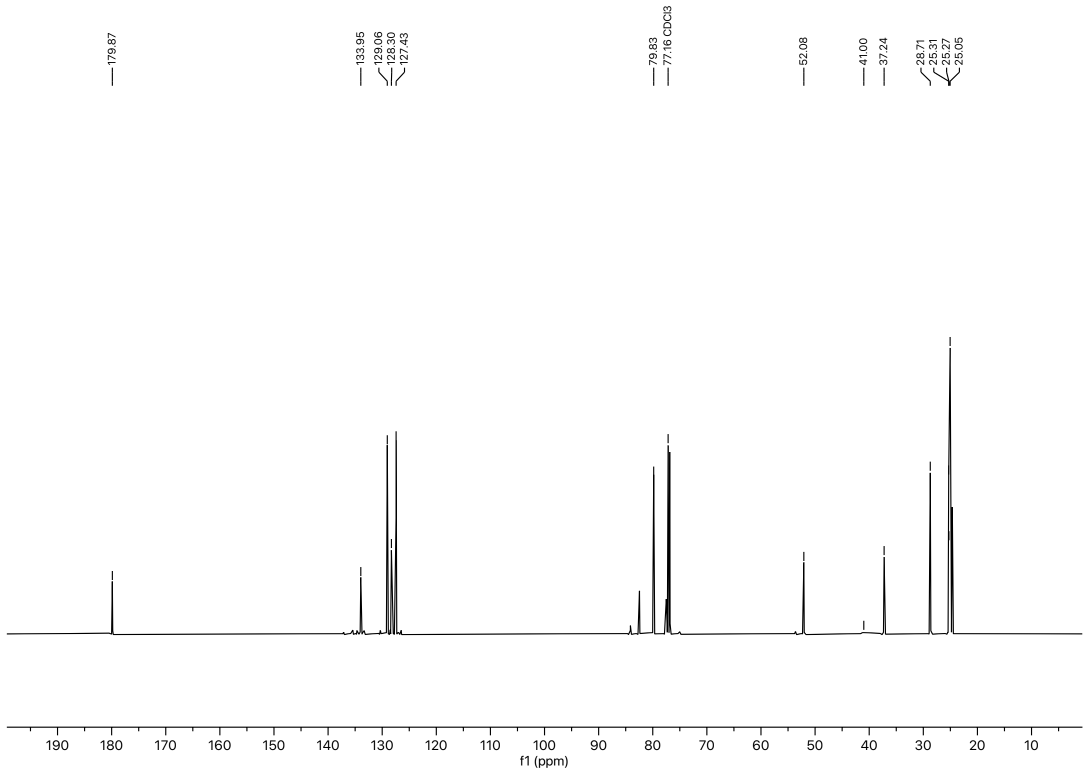

^{13}C DEPT-135 (2y)

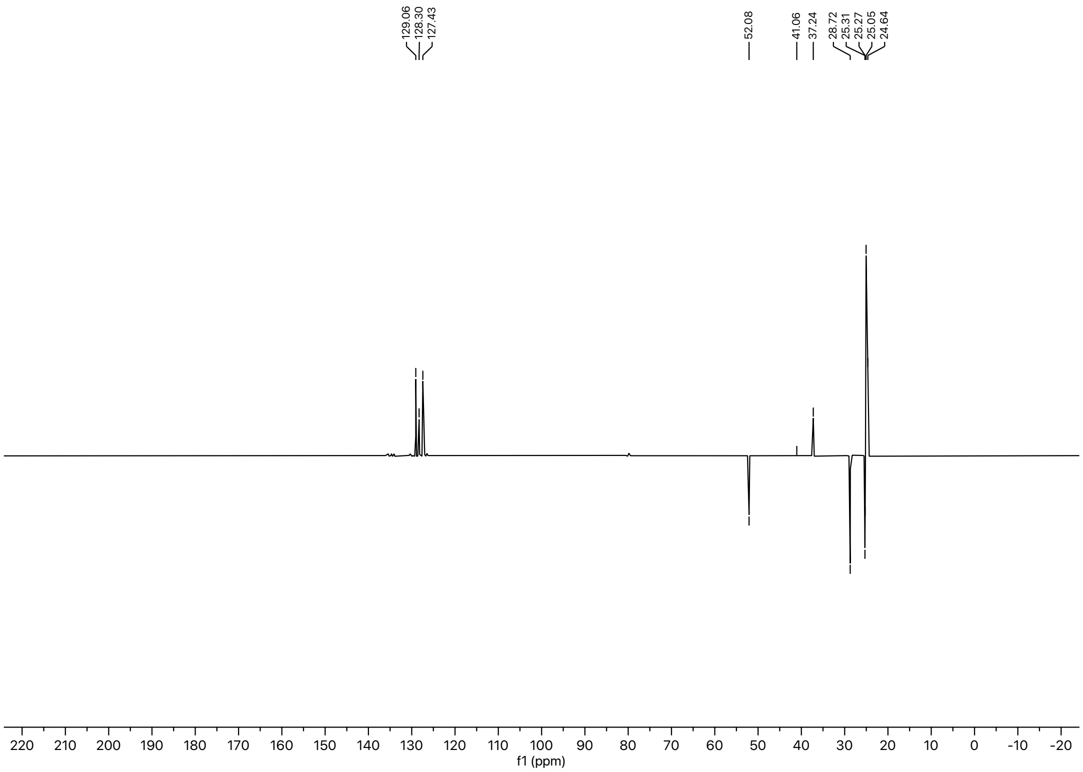

^{11}B NMR (2y)

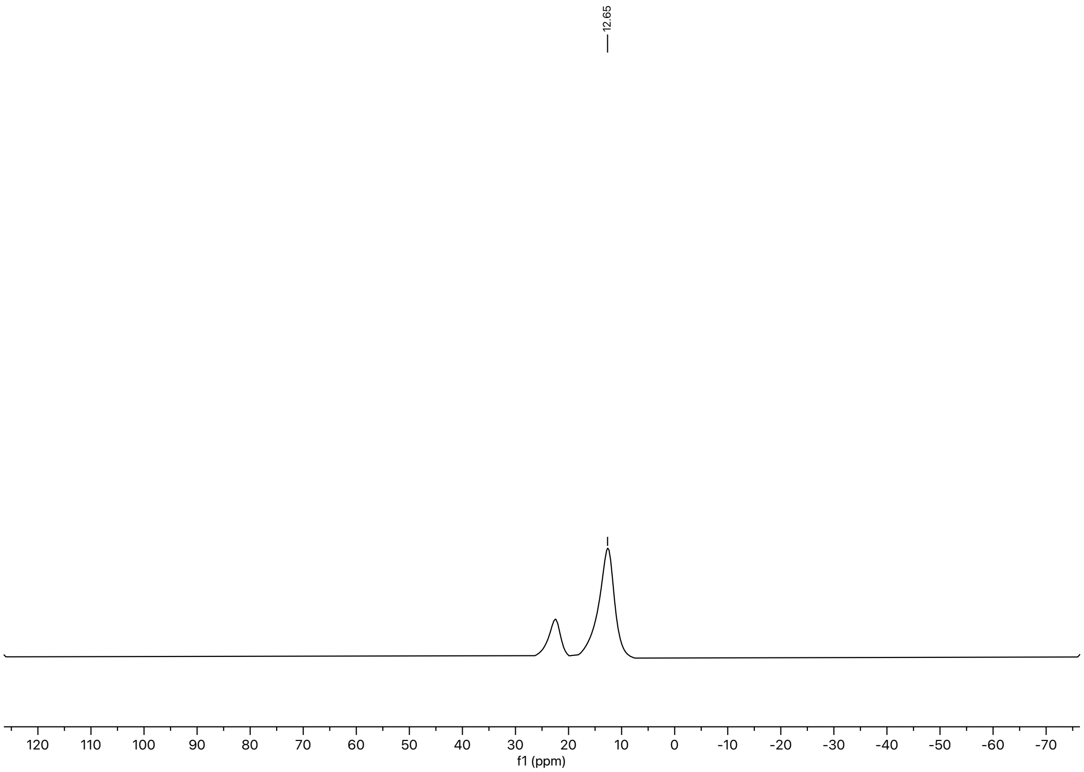

^{1}H NMR (2z)

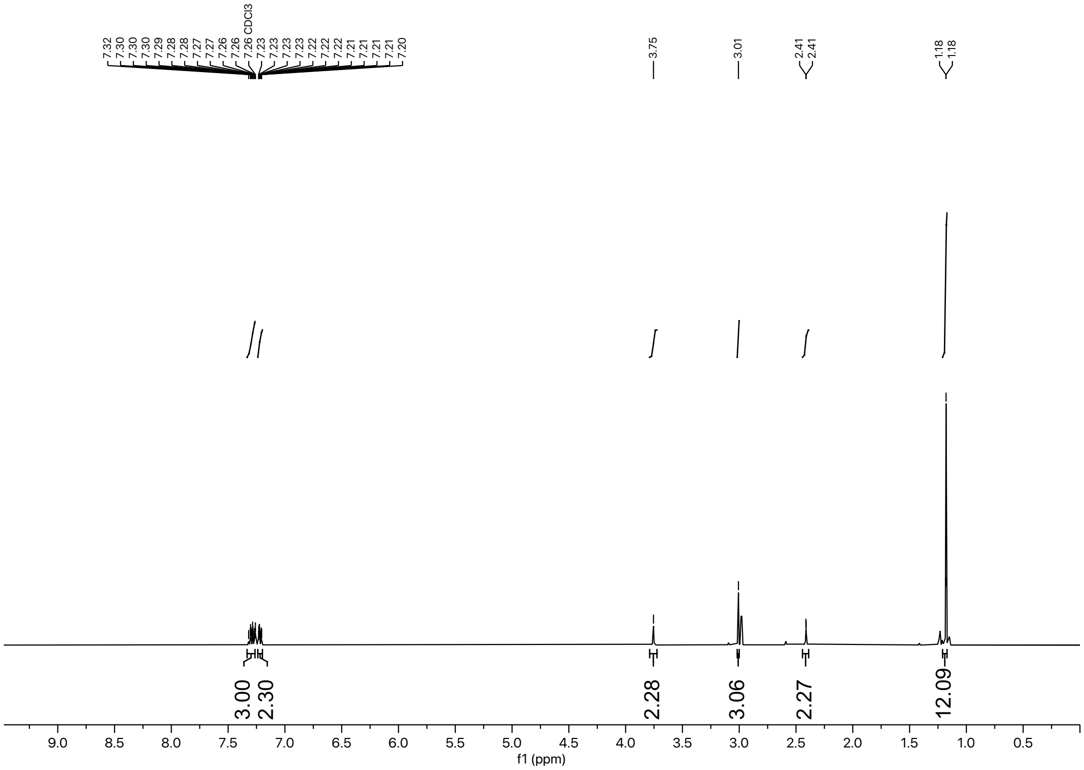

^{13}C NMR (2z)

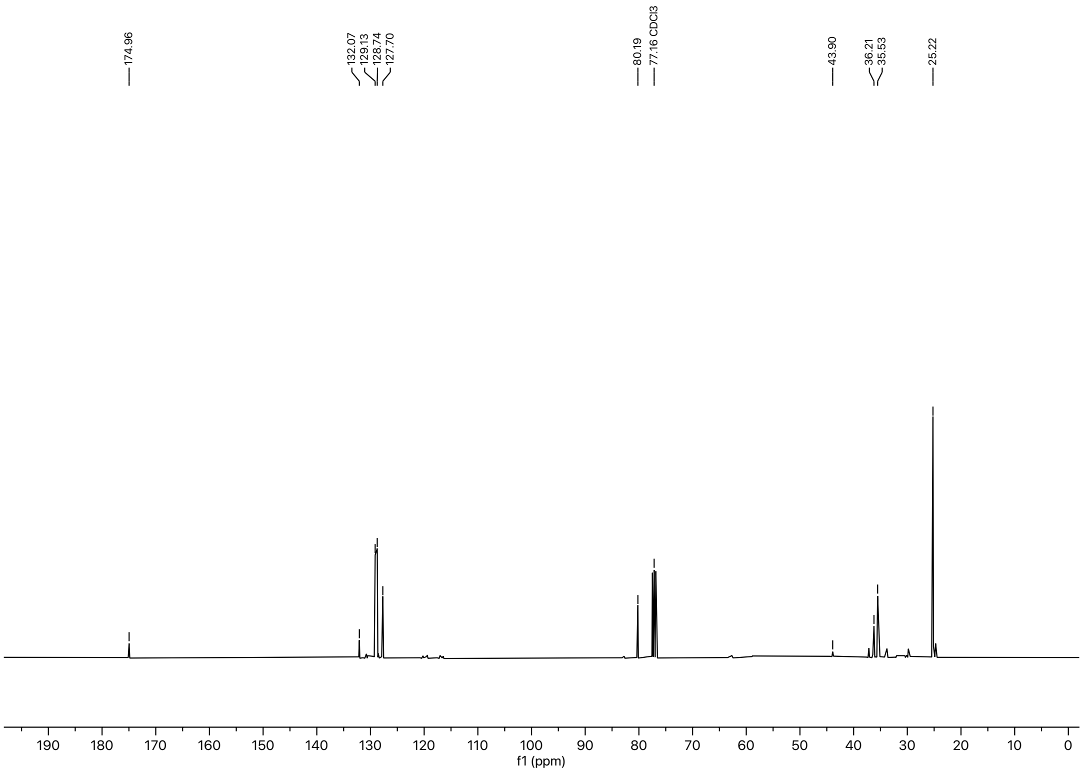

^{13}C DEPT-135 (2z)

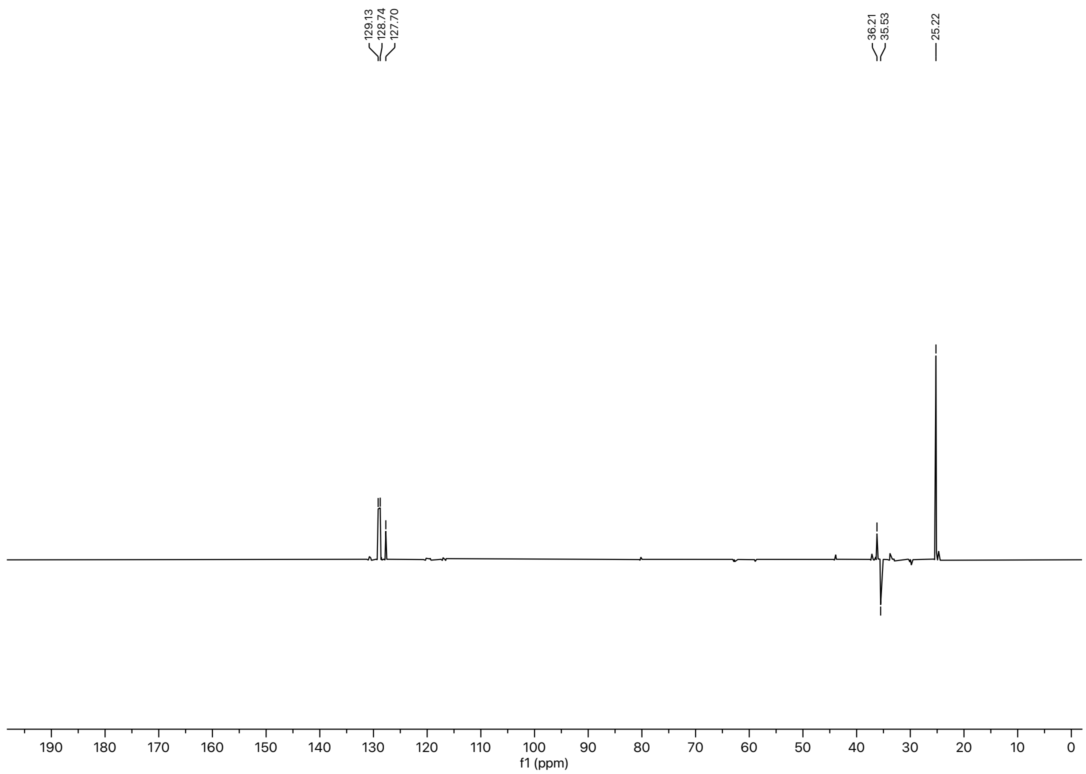

^{11}B NMR (2z)

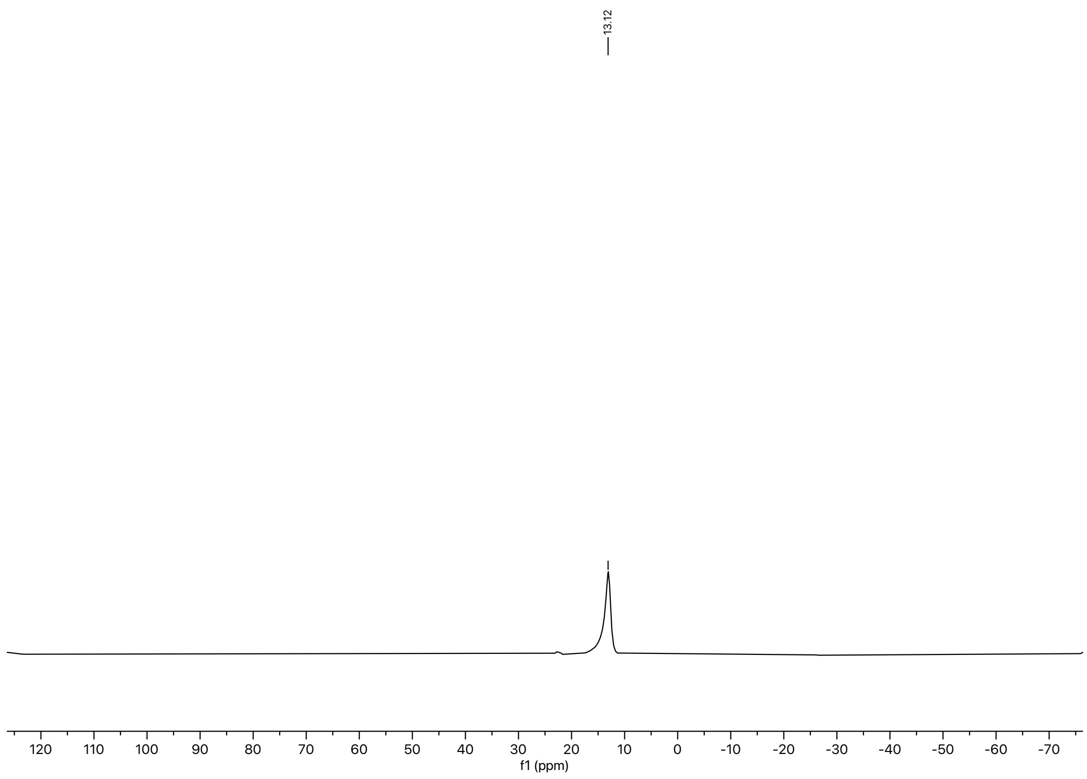

^1H NMR (2aa)

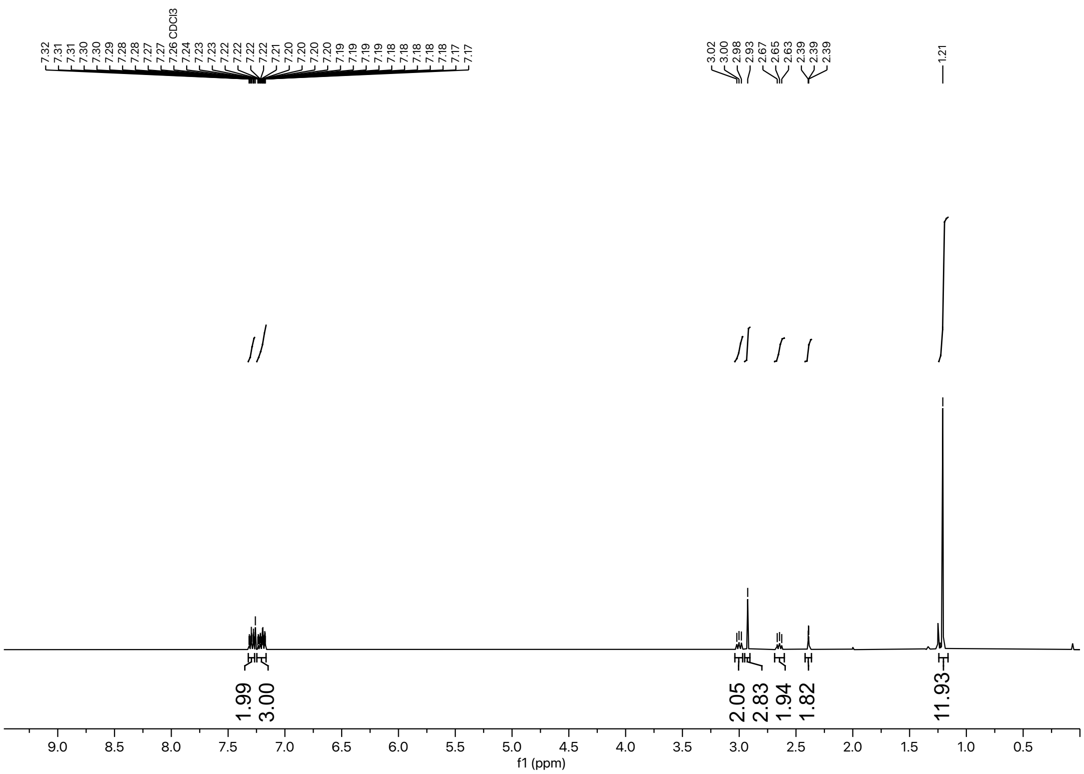

^{13}C NMR (2aa)

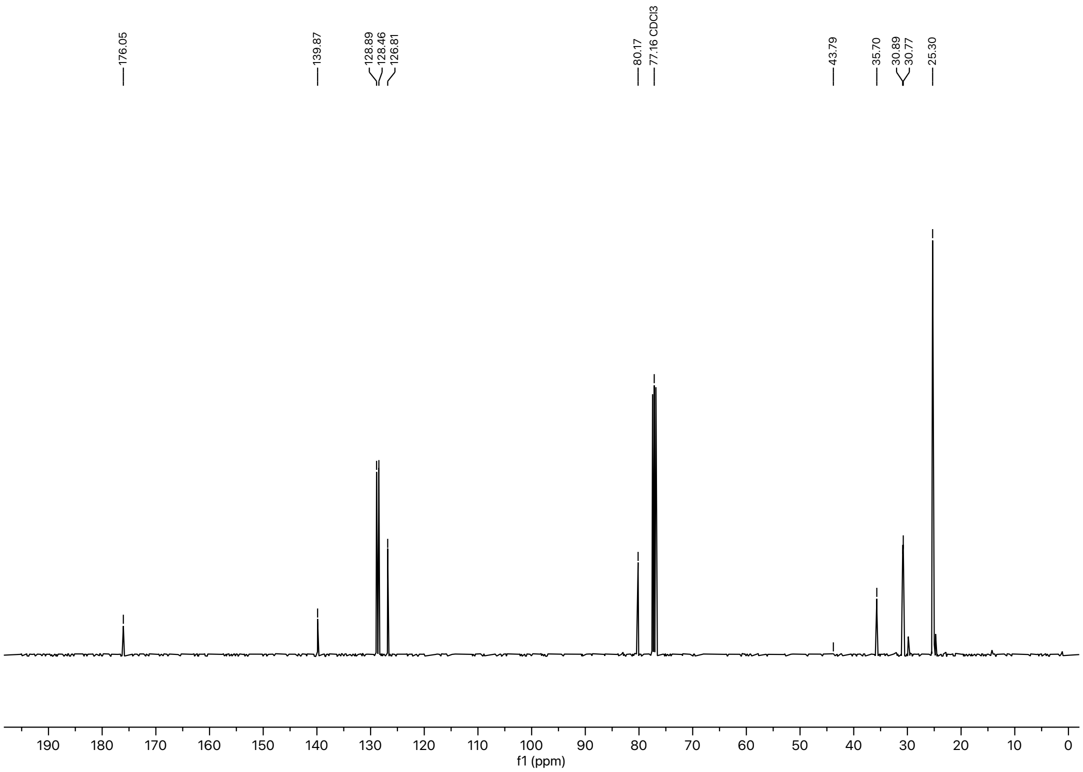

^{13}C DEPT-135 (2aa)

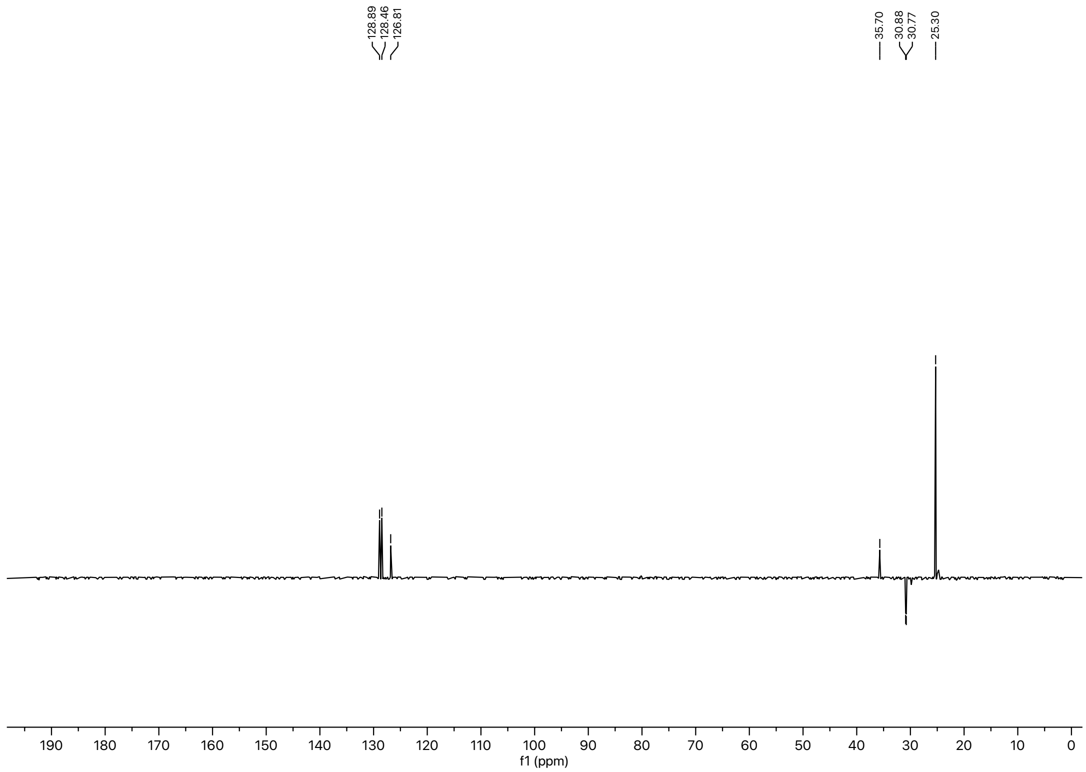

^{11}B NMR (2aa)

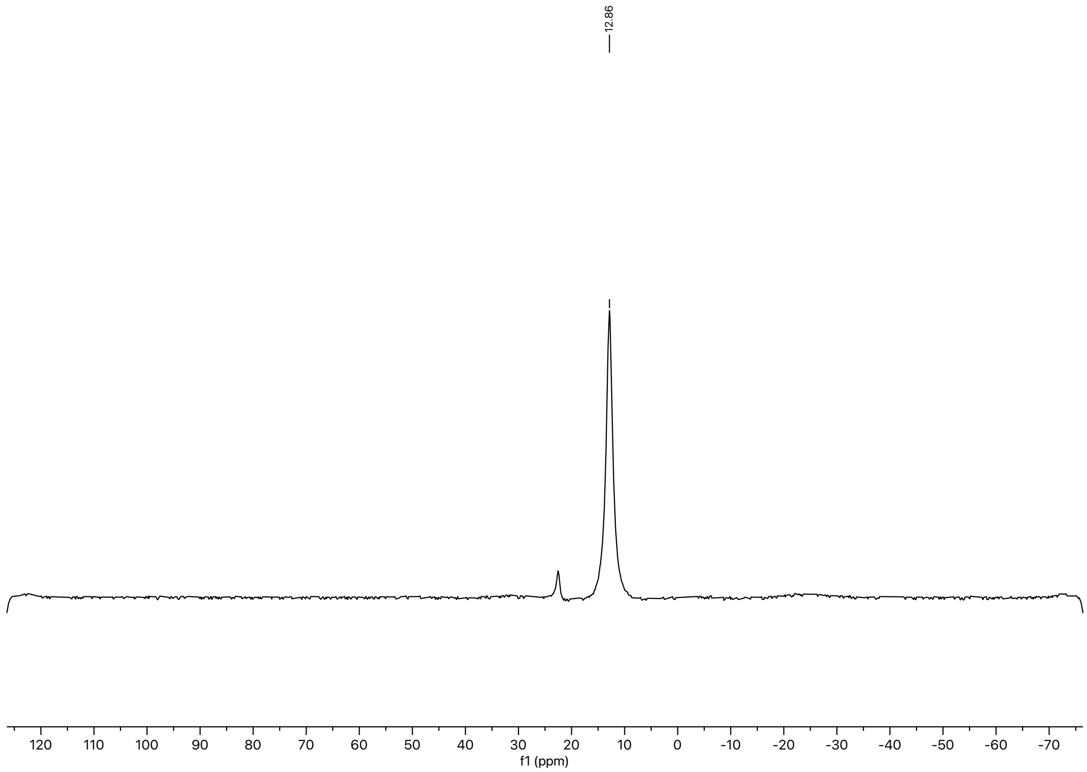

^{1}H NMR (2ab)

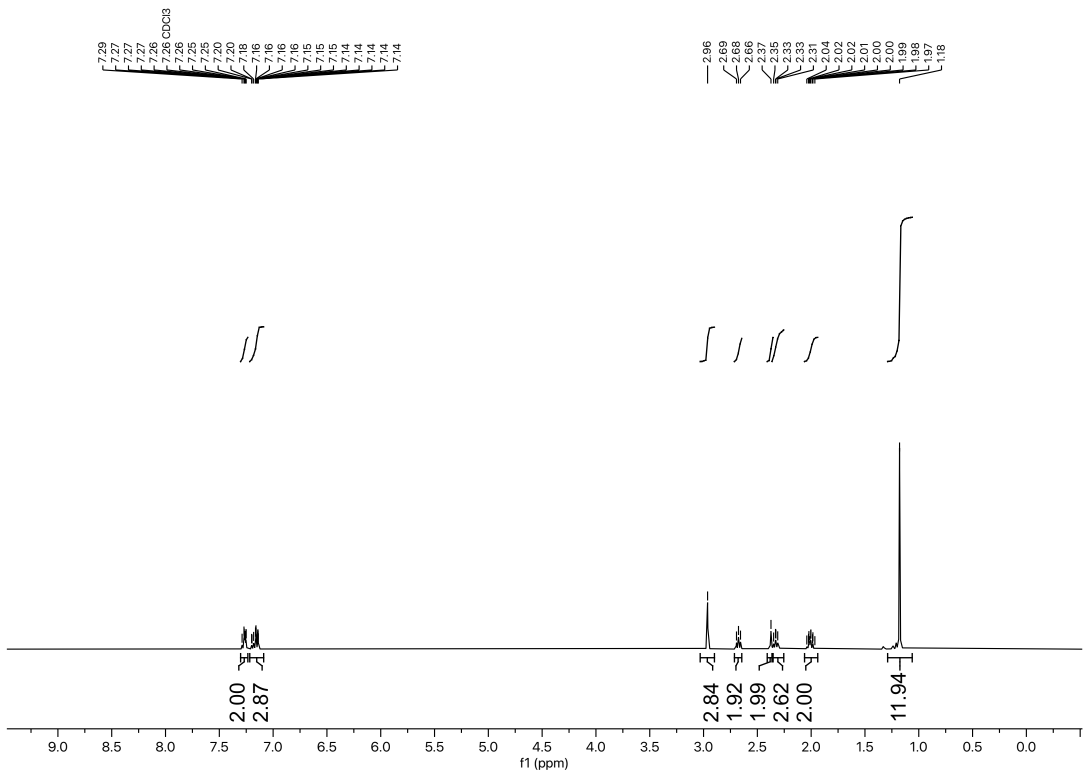

^{13}C NMR (2ab)

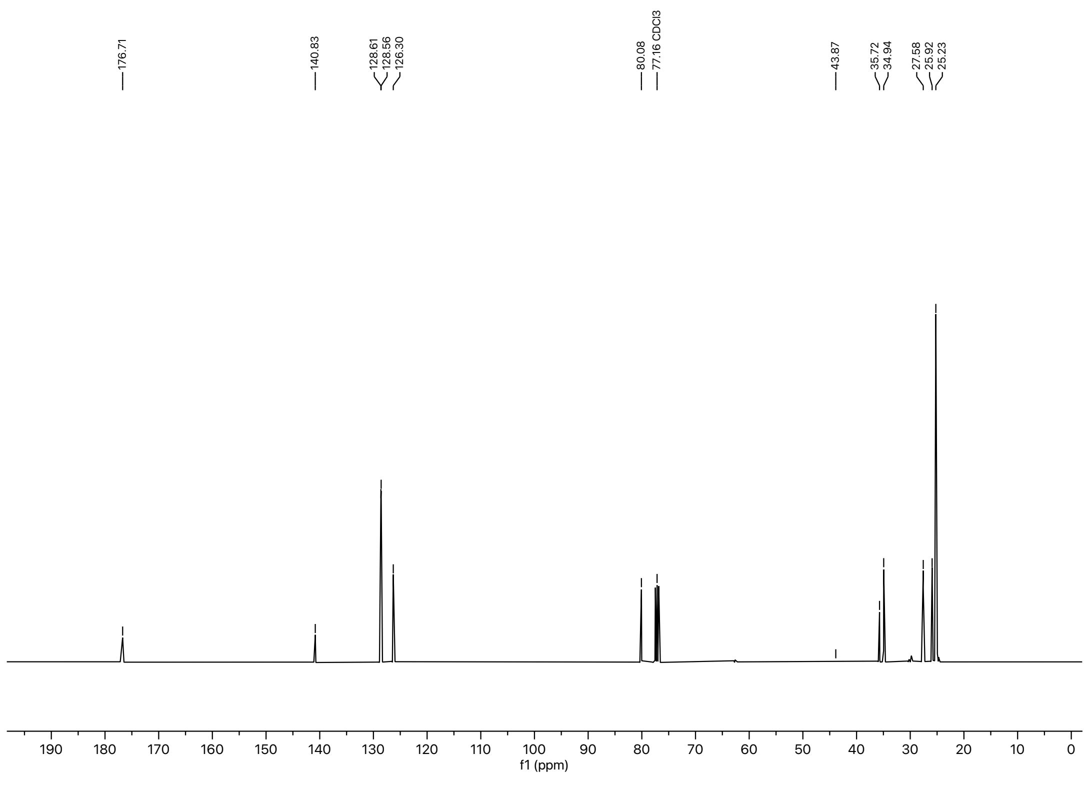

^{13}C DEPT-135 (2ab)

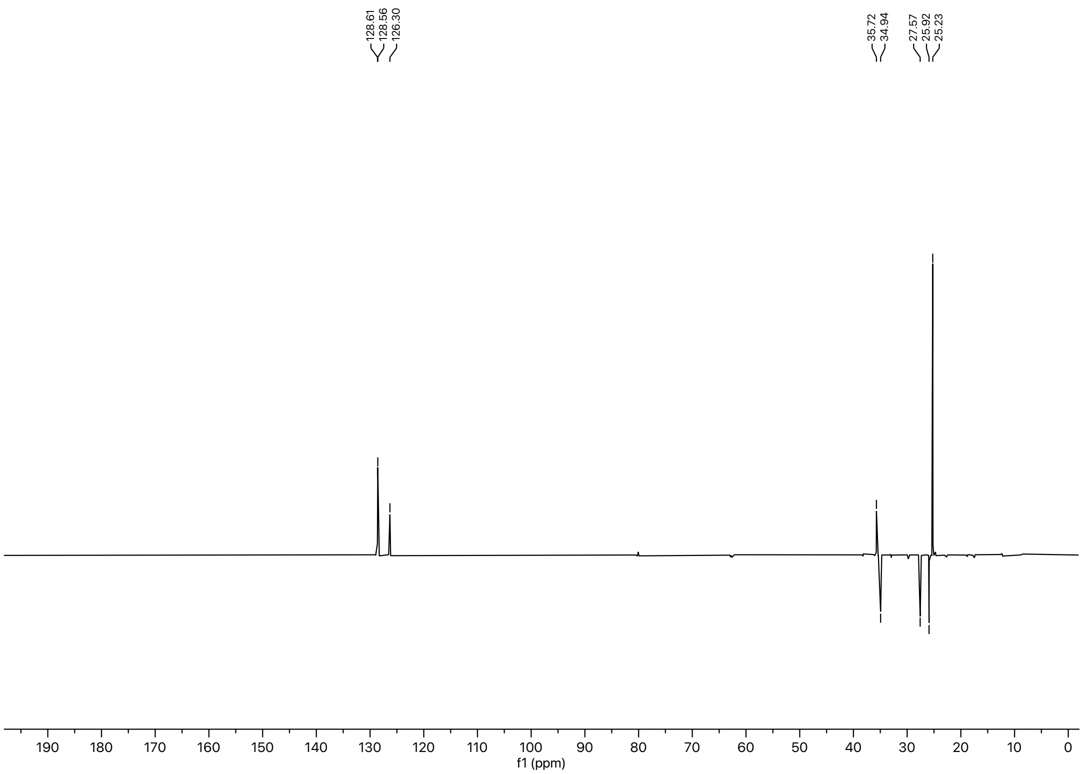

^{11}B NMR (2ab)

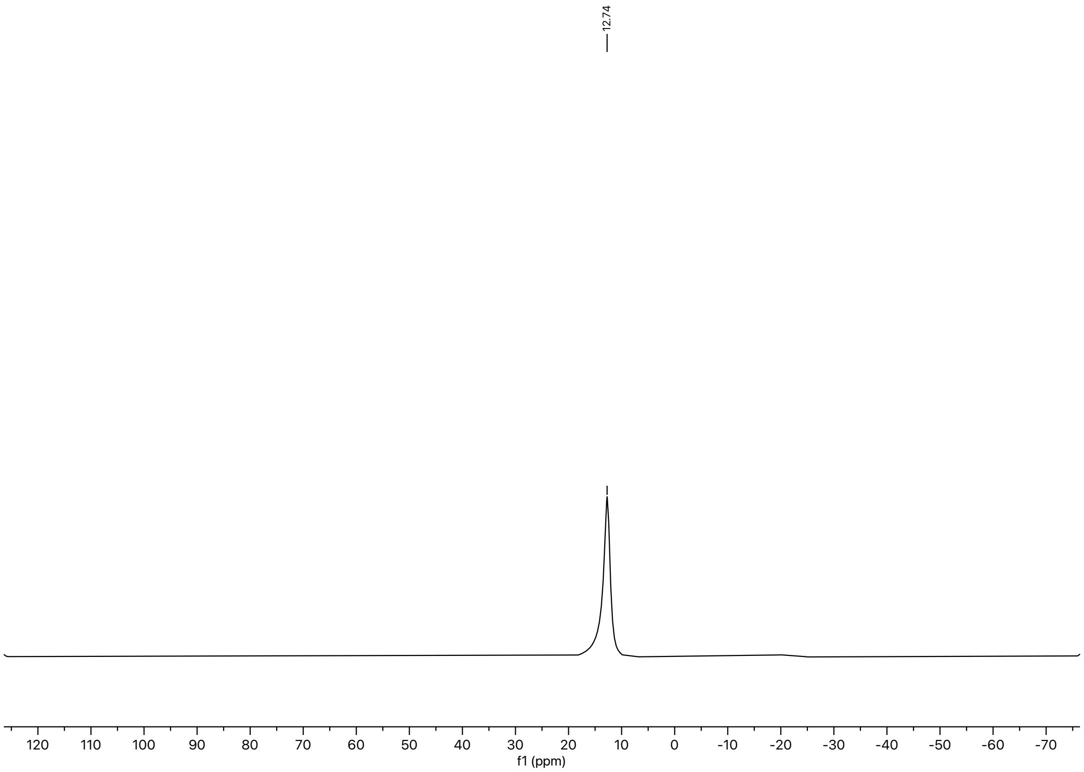

^{1}H NMR (2ac)

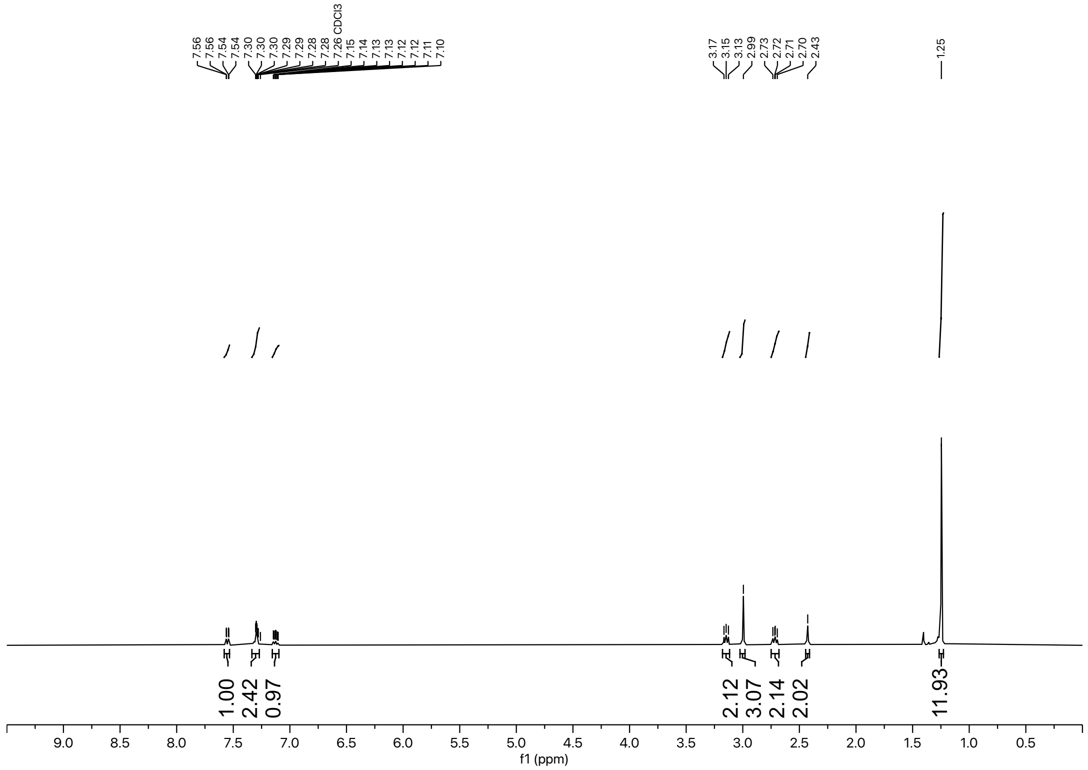

^{13}C NMR (2ac)

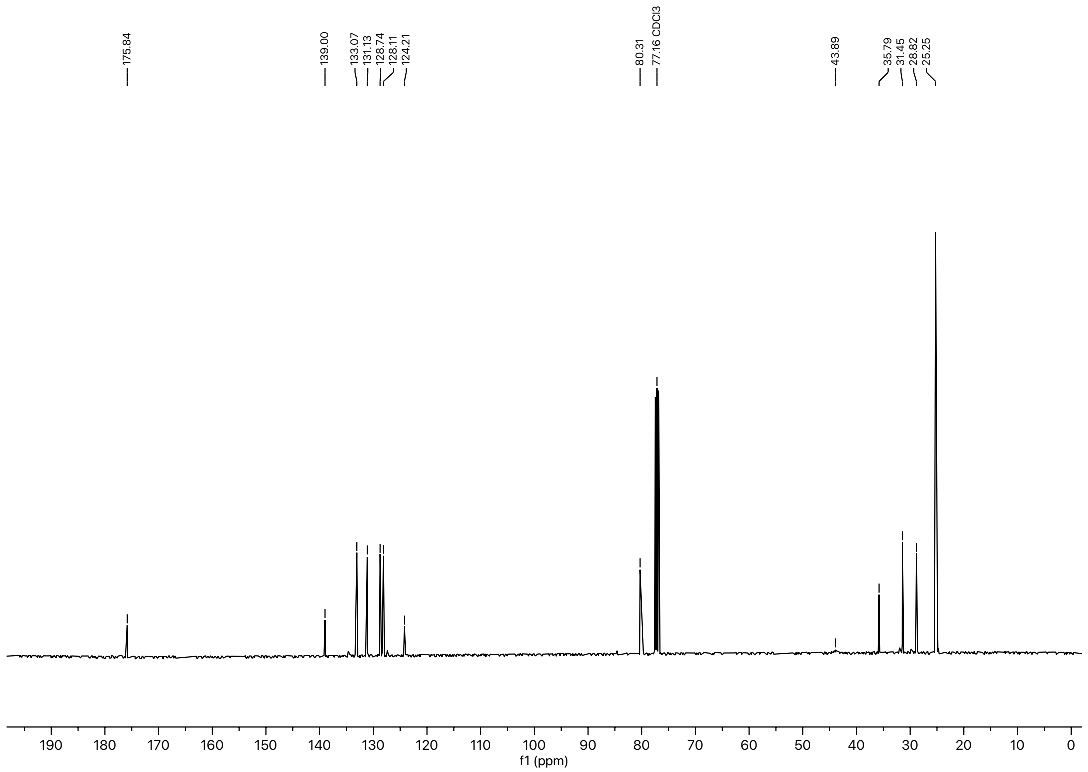

^{13}C DEPT-135 (2ac)

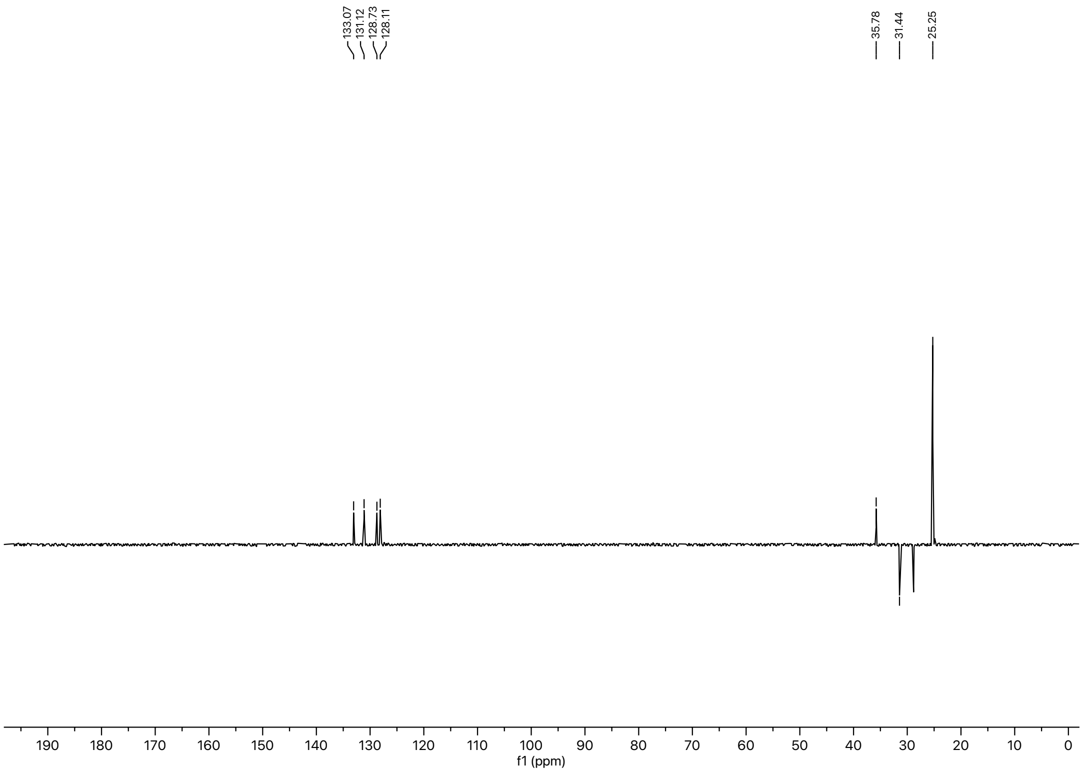

^{11}B NMR (2ac)

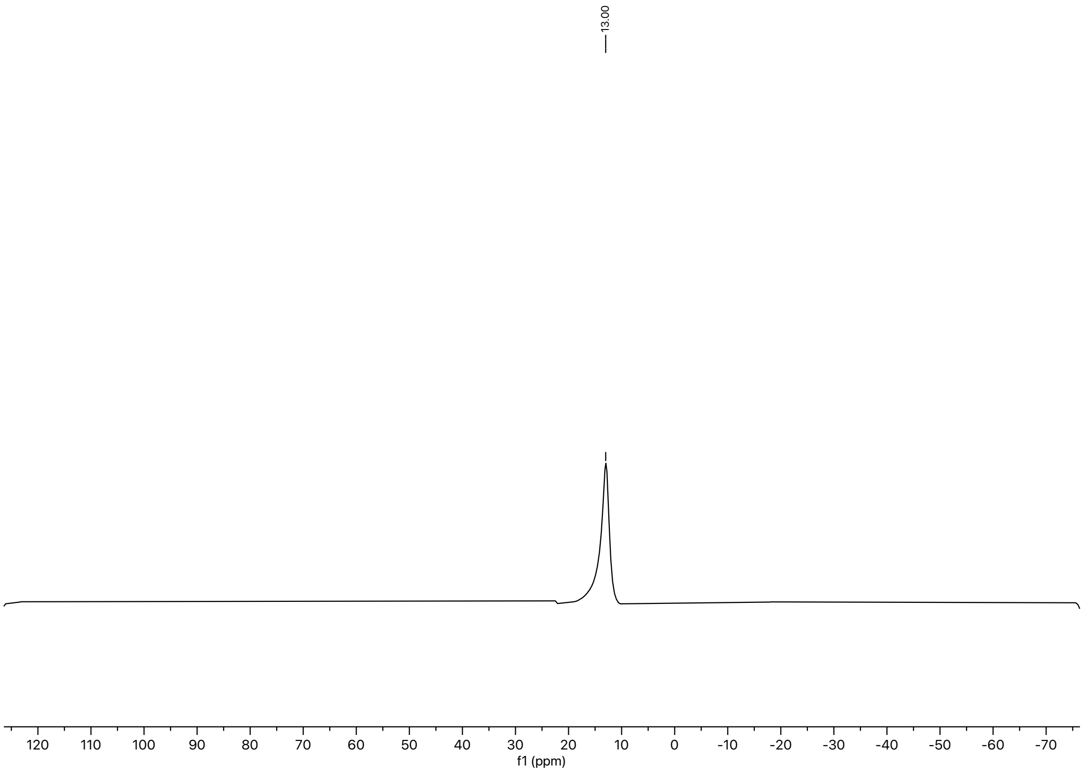

^{1}H NMR (2ad)

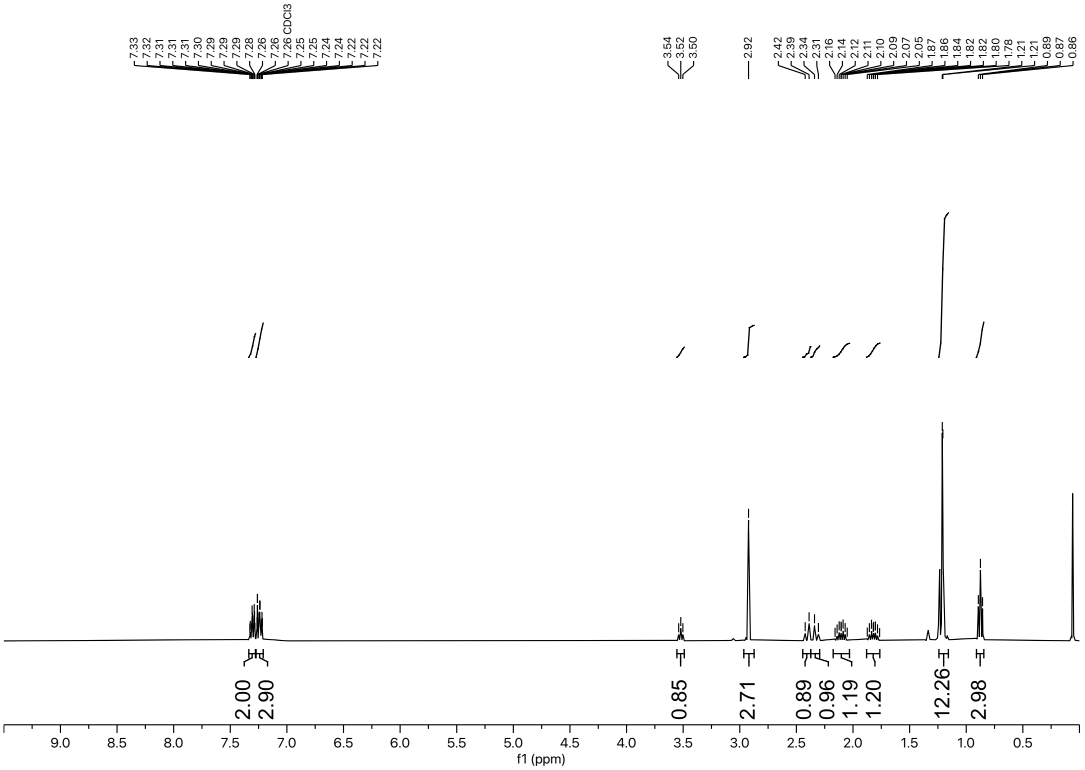

^{13}C NMR (2ad)

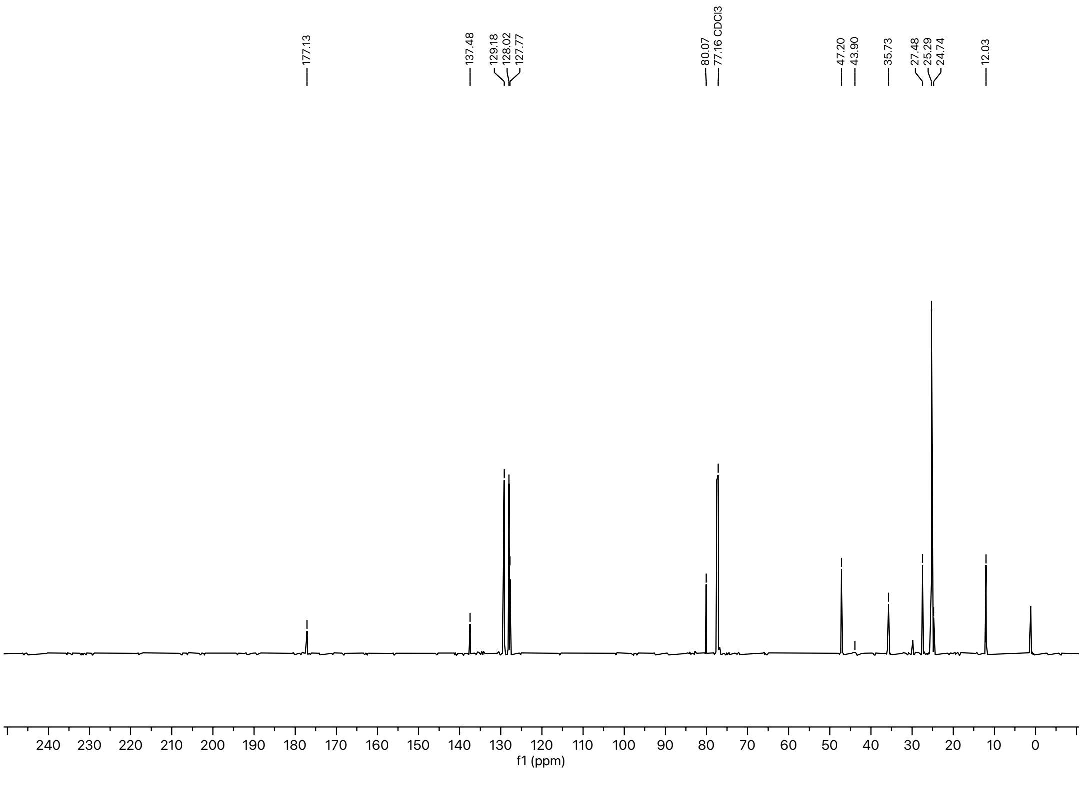

^{13}C DEPT-135 (2ad)

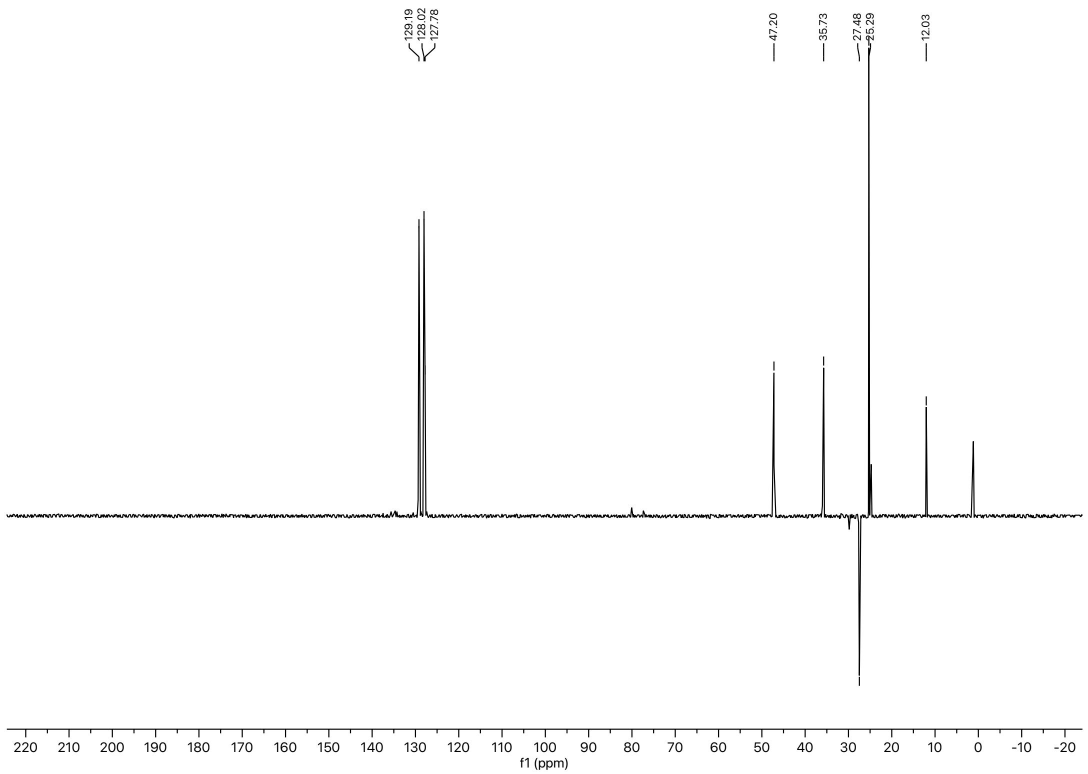

^{11}B NMR (2ad)

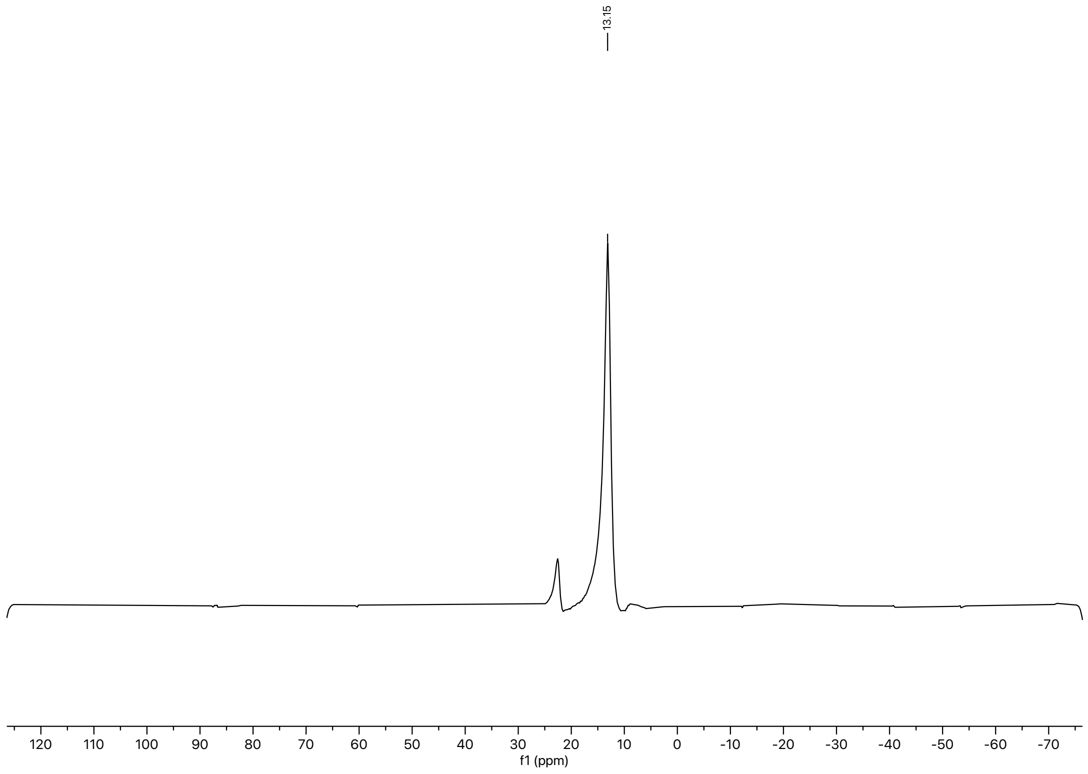

^{1}H NMR (2ae)

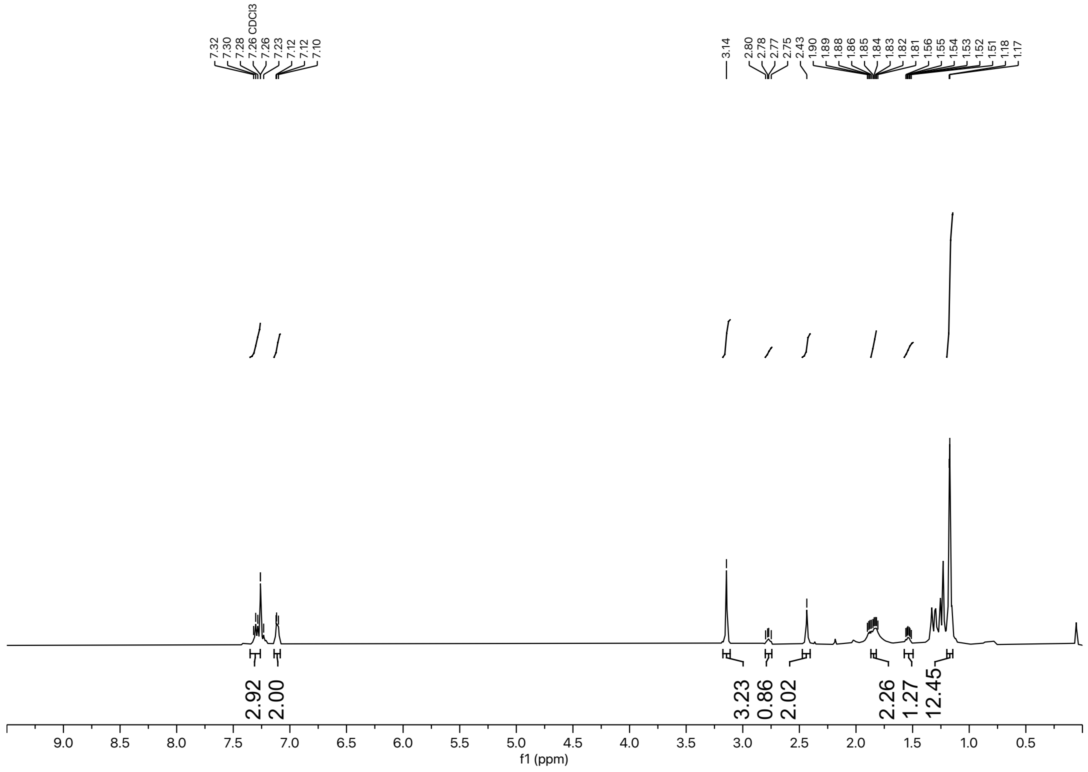

^{13}C NMR (2ae)

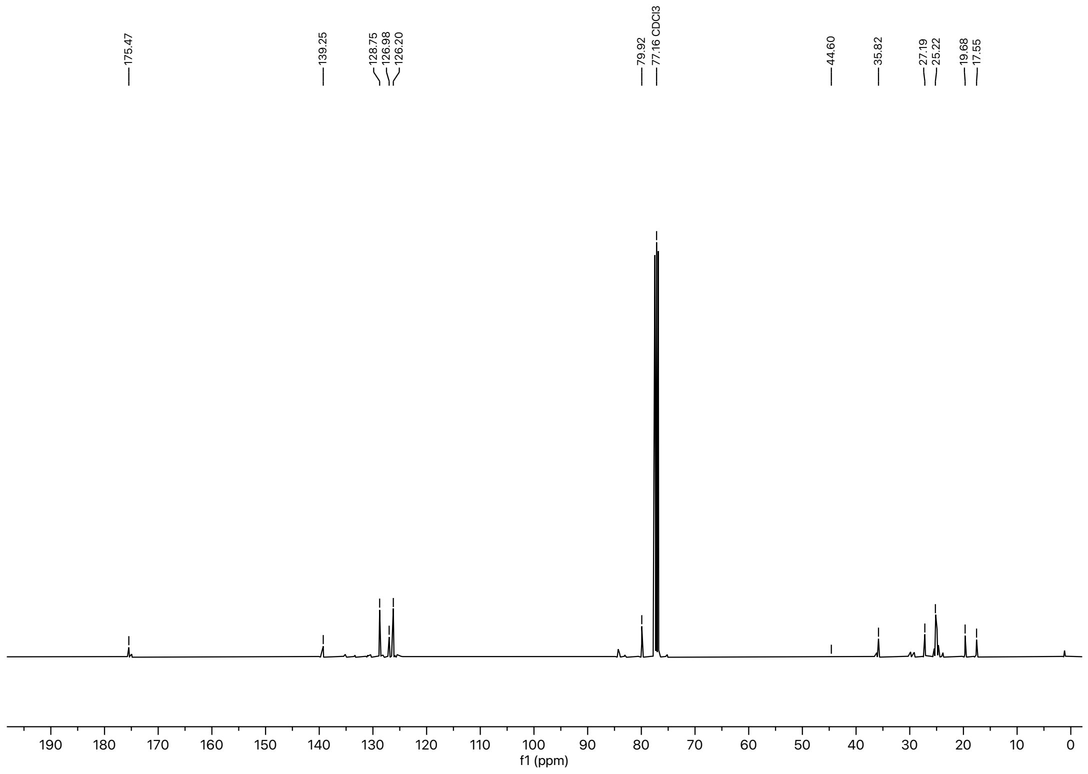

^{13}C DEPT-135 (2ae)

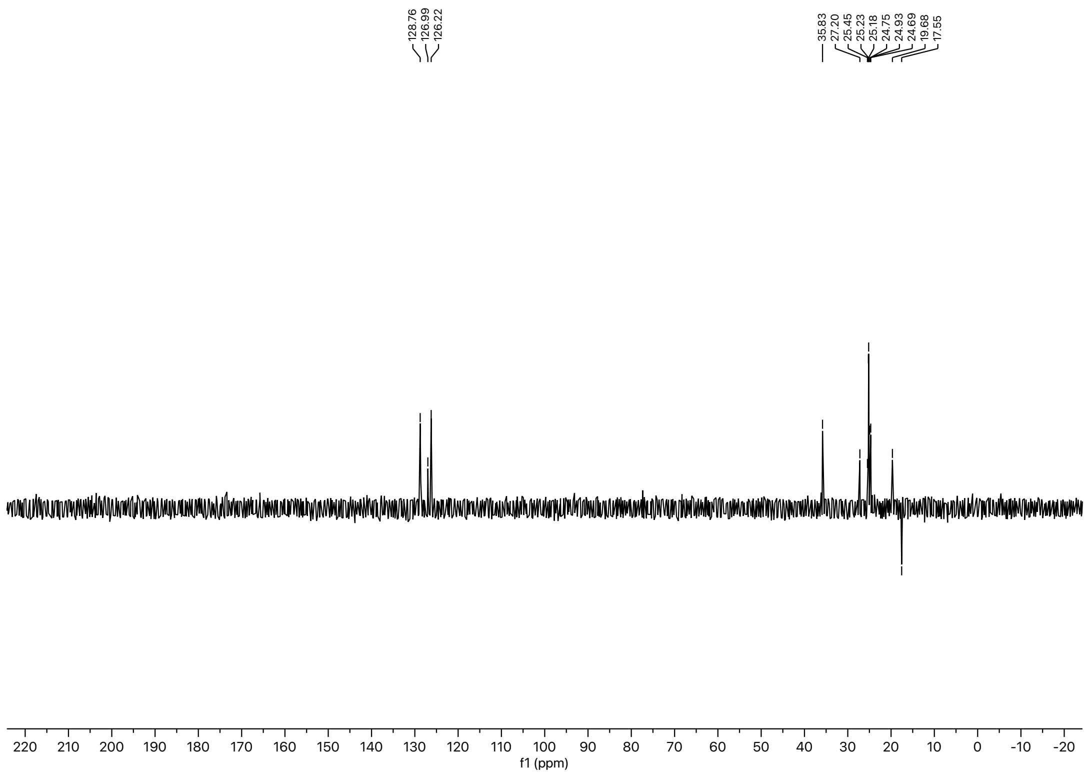

^{11}B NMR (2ae)

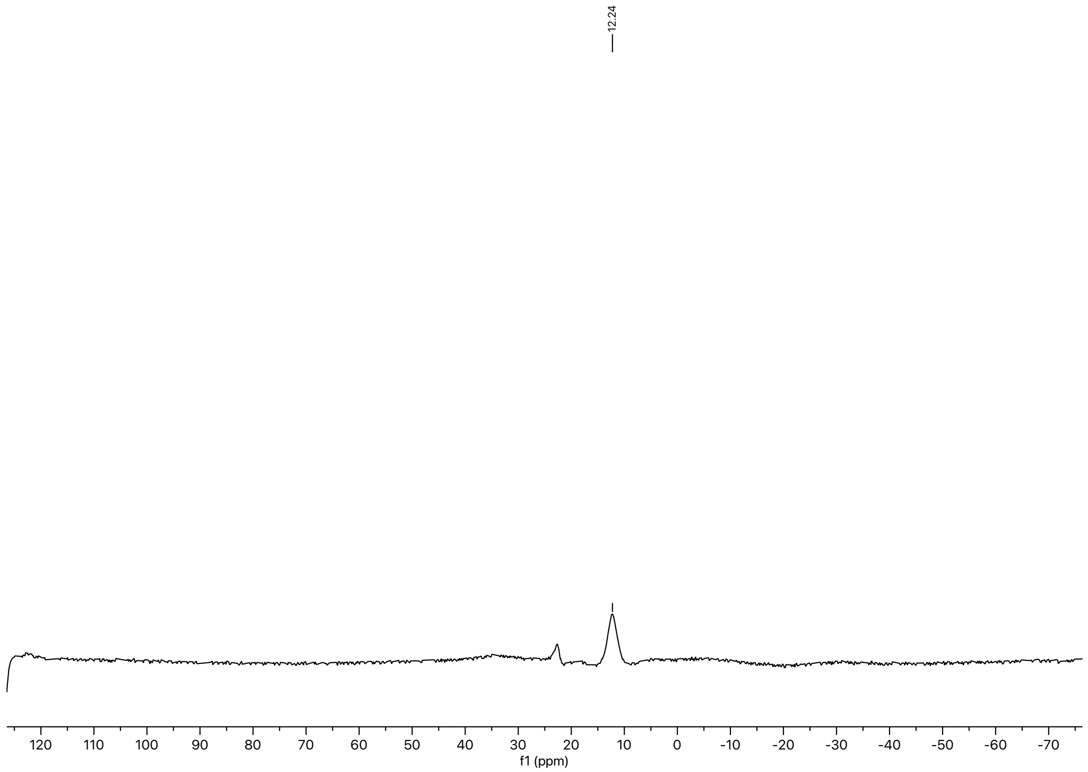

^1H NMR (2af)

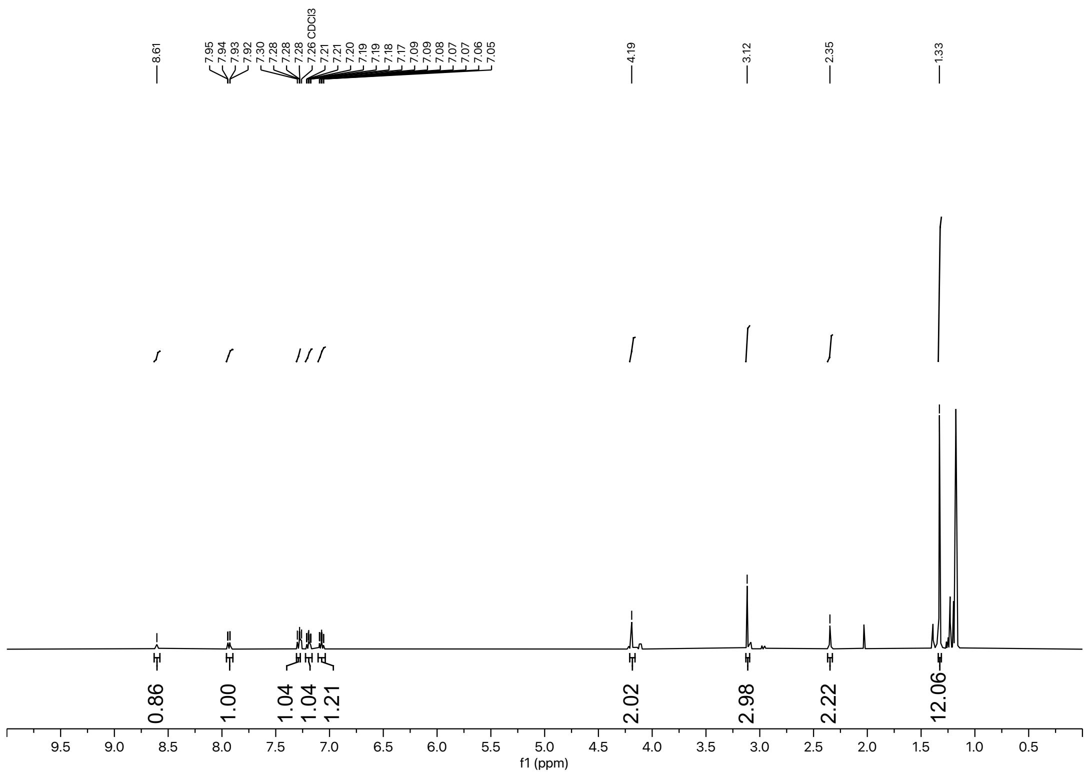

^{13}C NMR (2af)

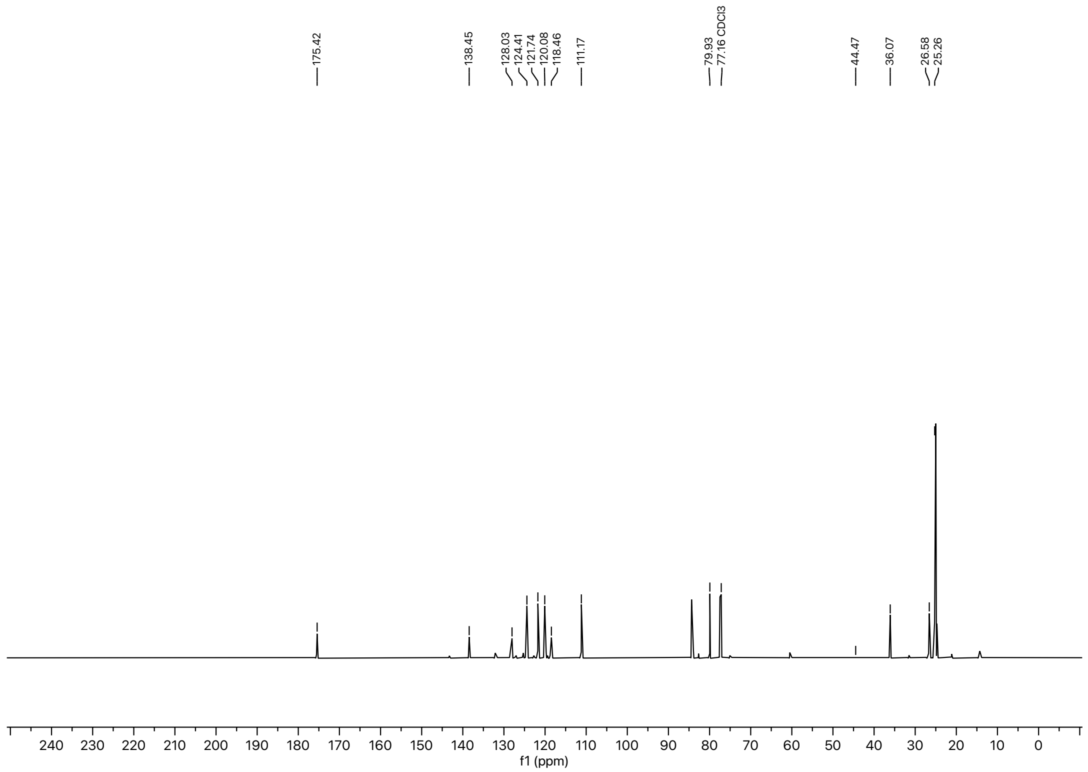

^{13}C DEPT-135 (2af)

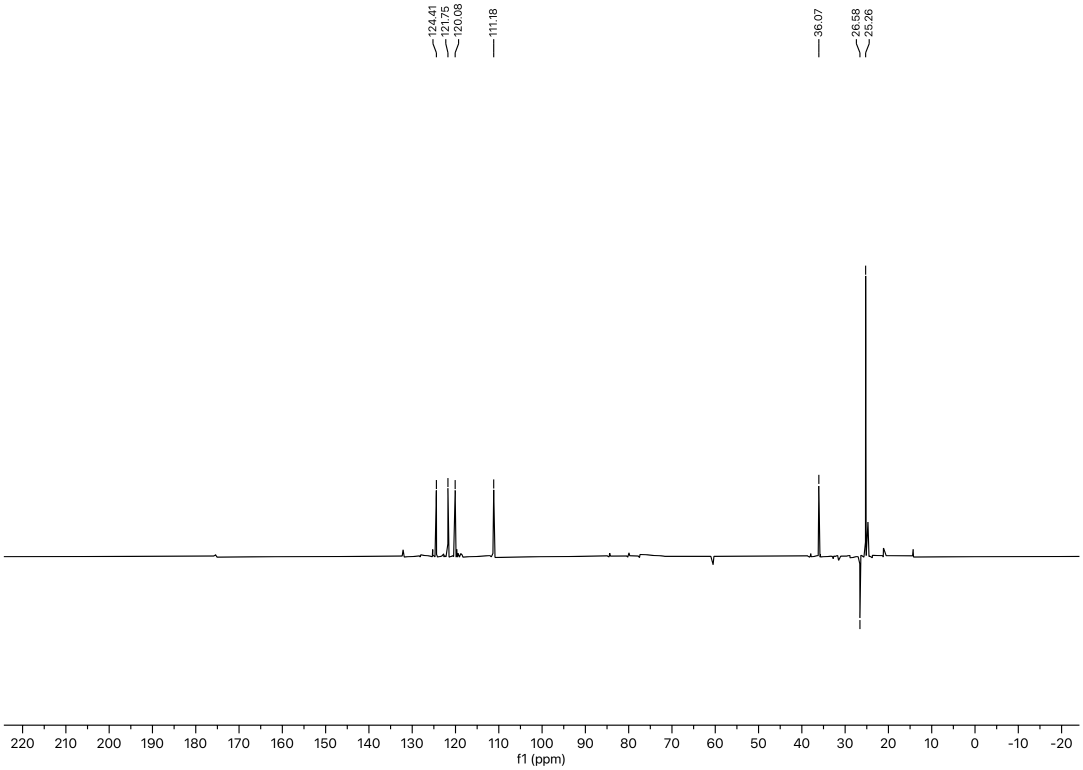

^{11}B NMR (2af)

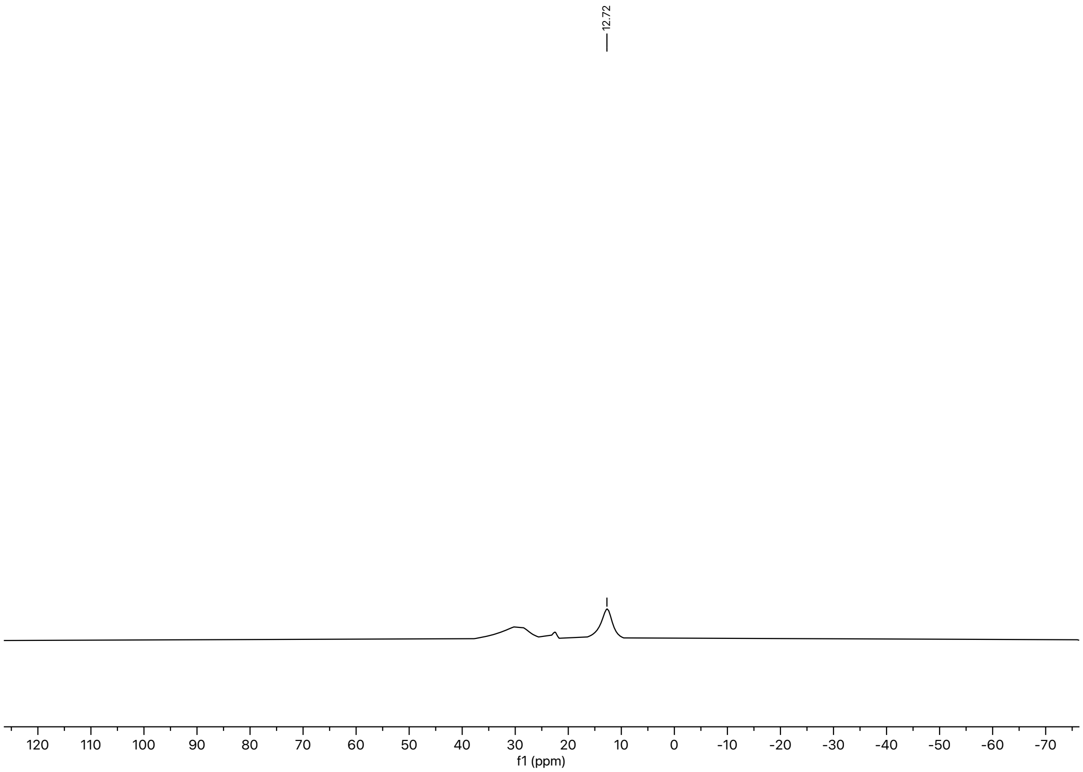

^{1}H NMR (2ag)

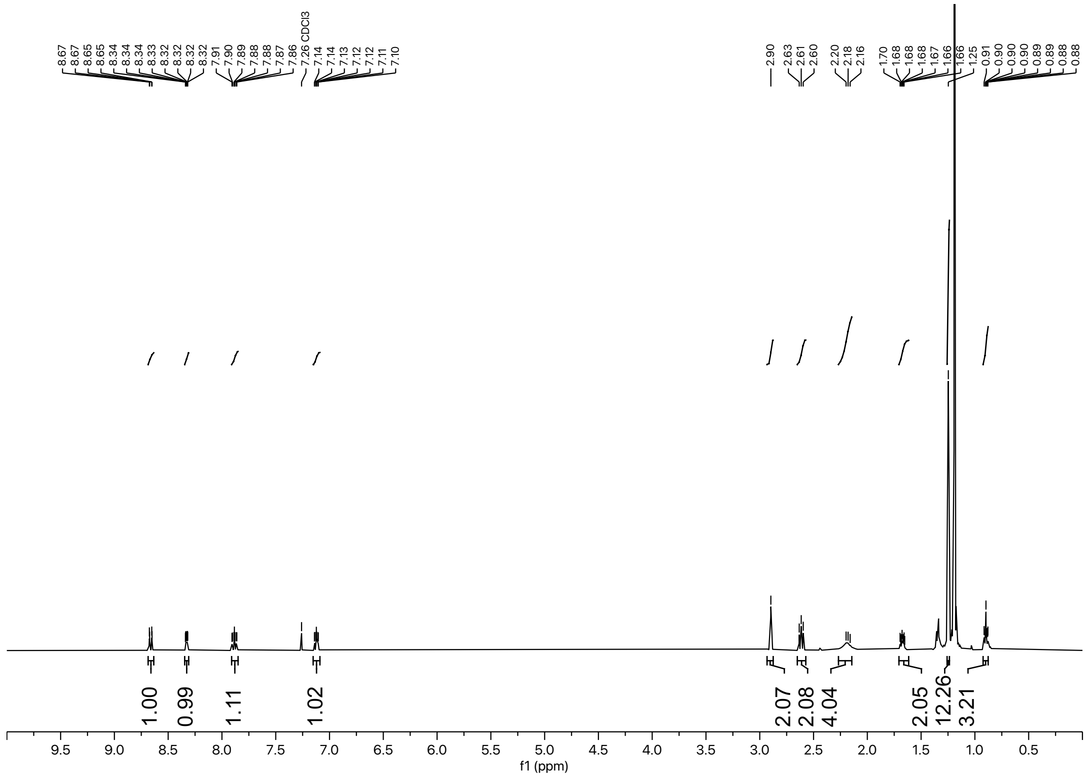

^{13}C NMR (2ag)

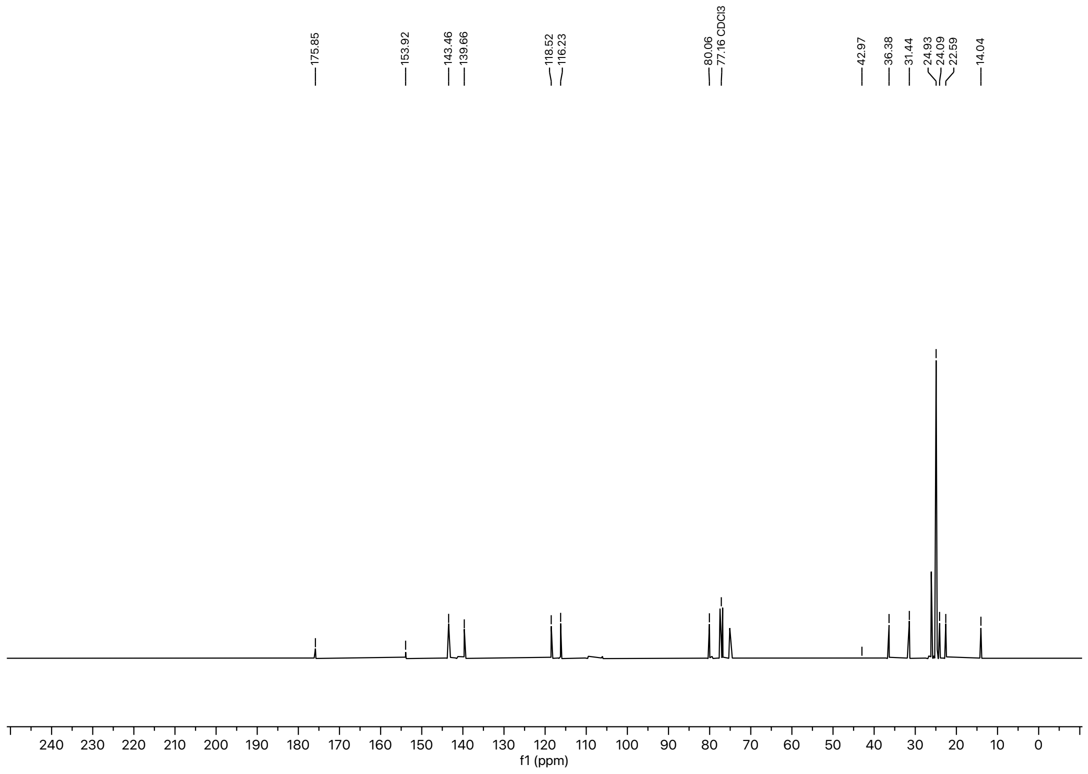

^{13}C DEPT-135 (2ag)

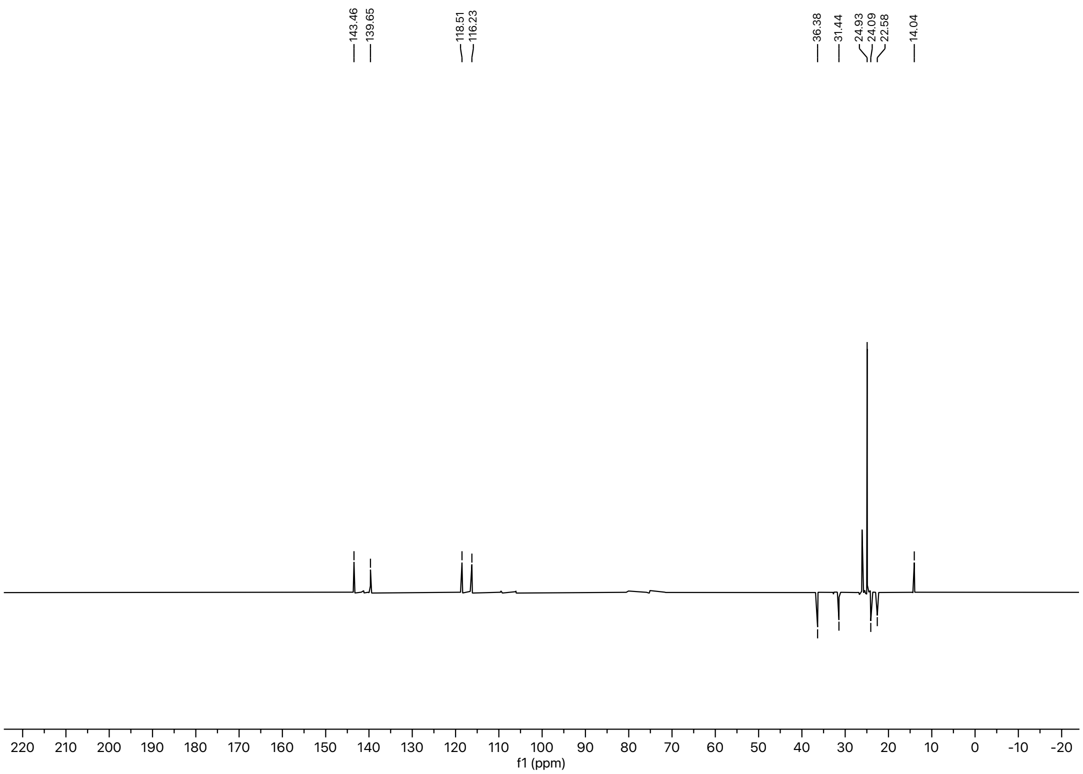

^{11}B NMR (2ag)

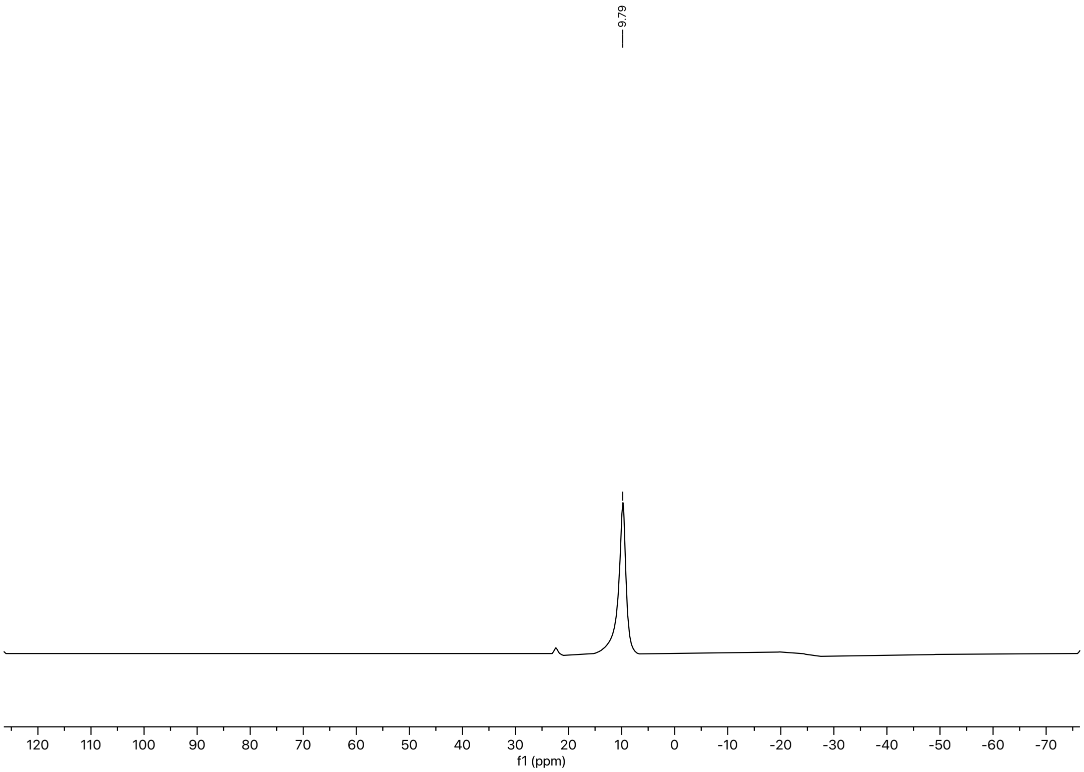

^1H NMR (2ah)

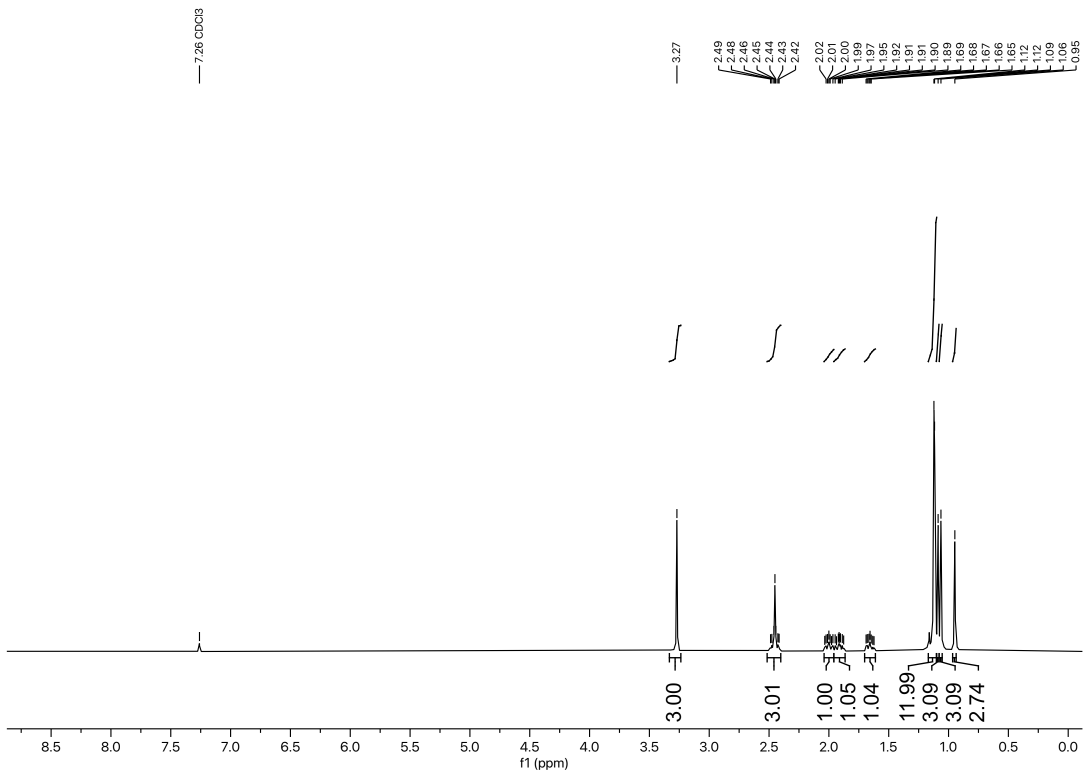

^{13}C NMR (2ah)

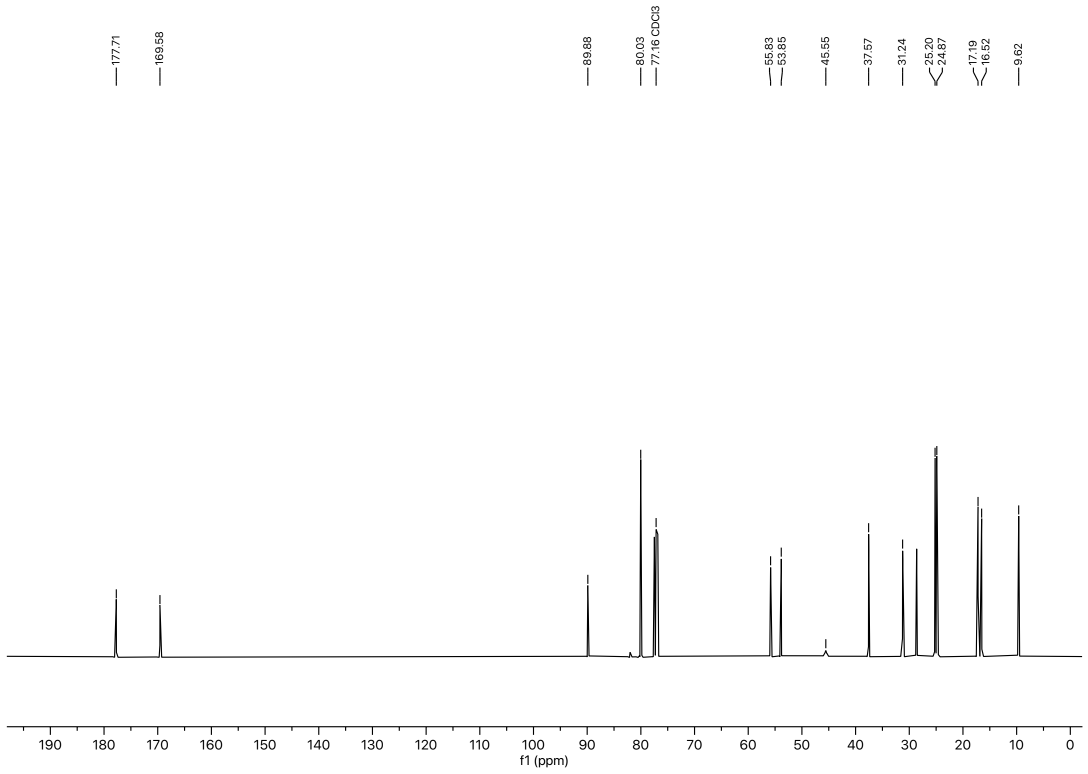

^{13}C DEPT-135 (2ah)

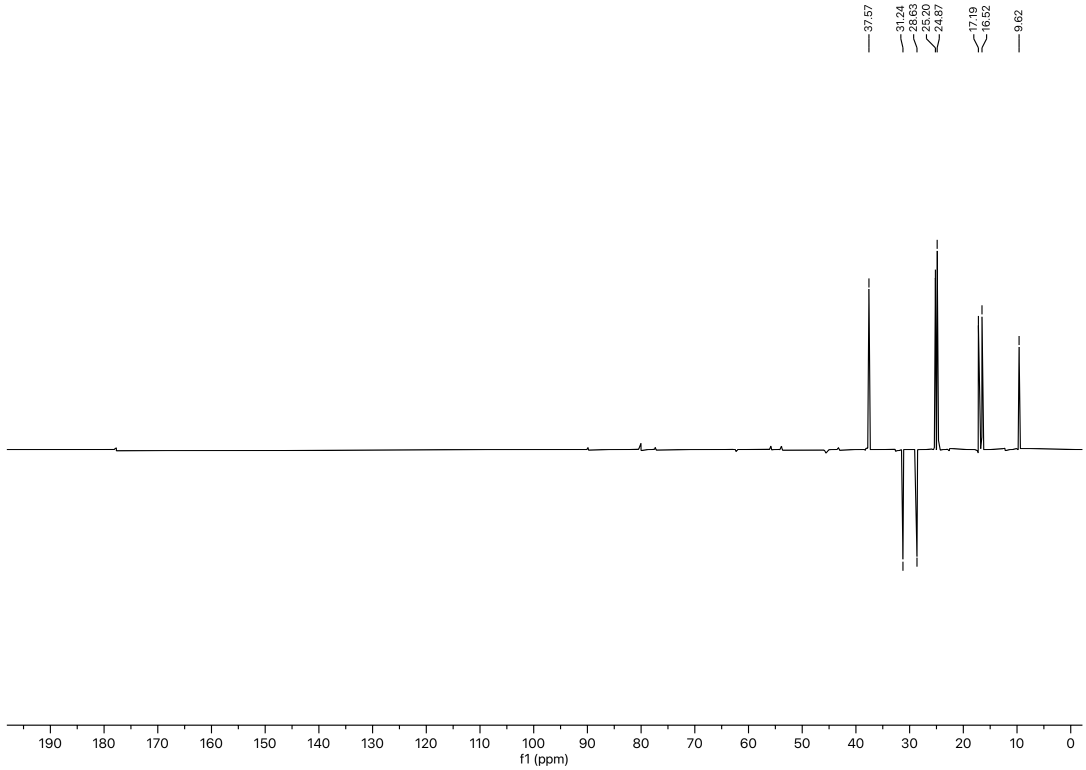

^{11}B NMR (2ah)

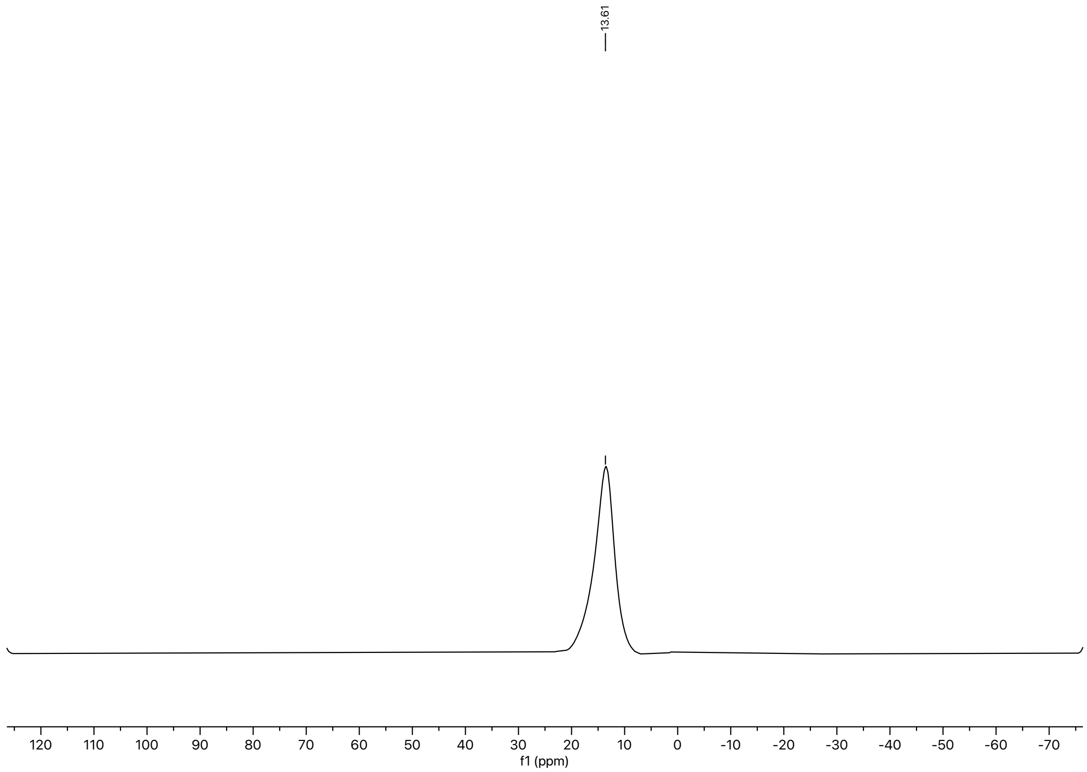

^{1}H NMR (2ai)

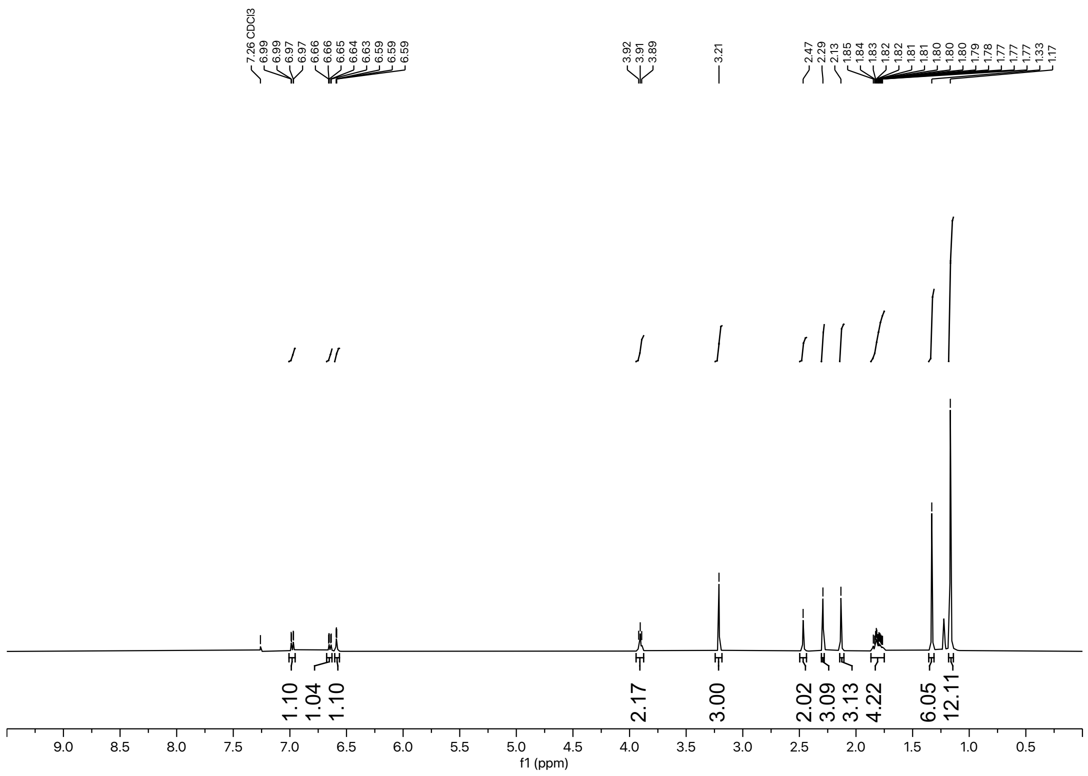

^{13}C NMR (2ai)

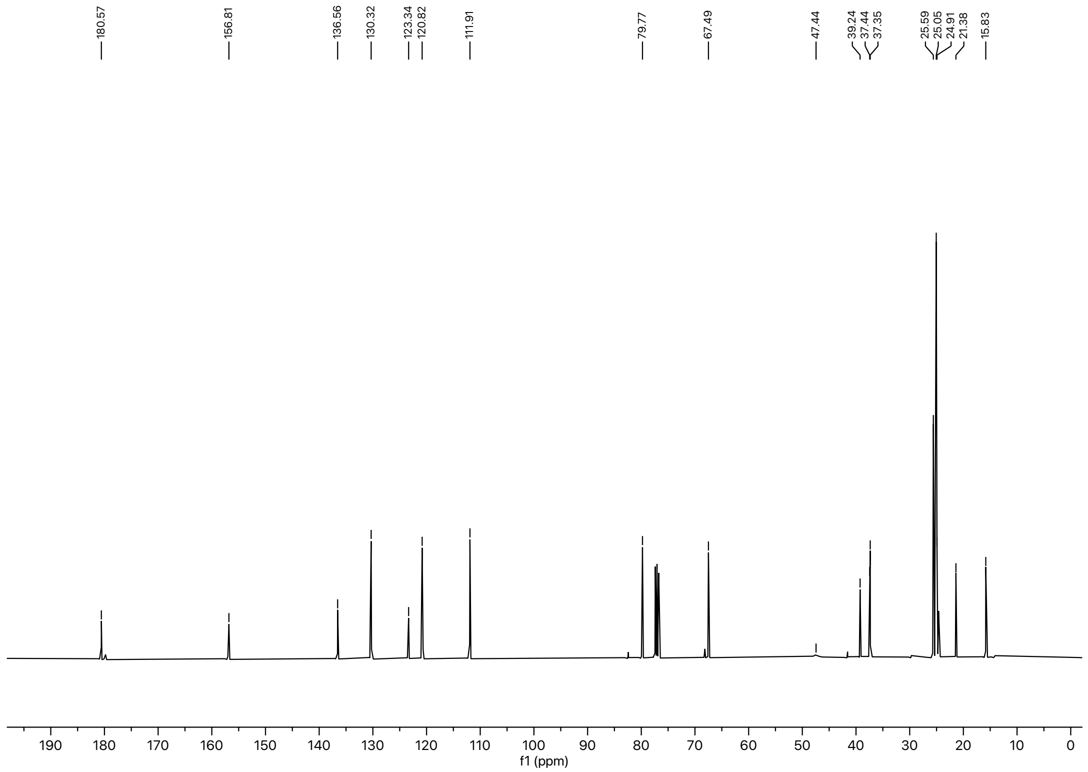

^{13}C DEPT-135 (2ai)

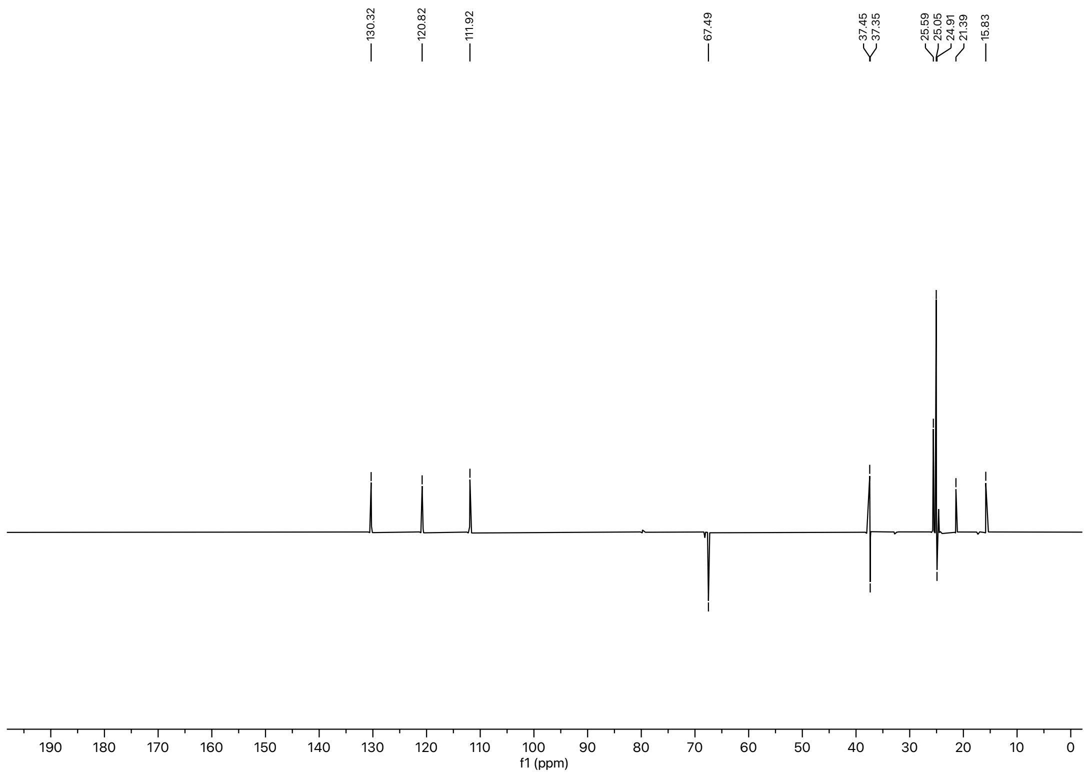

^{11}B NMR (2ai)

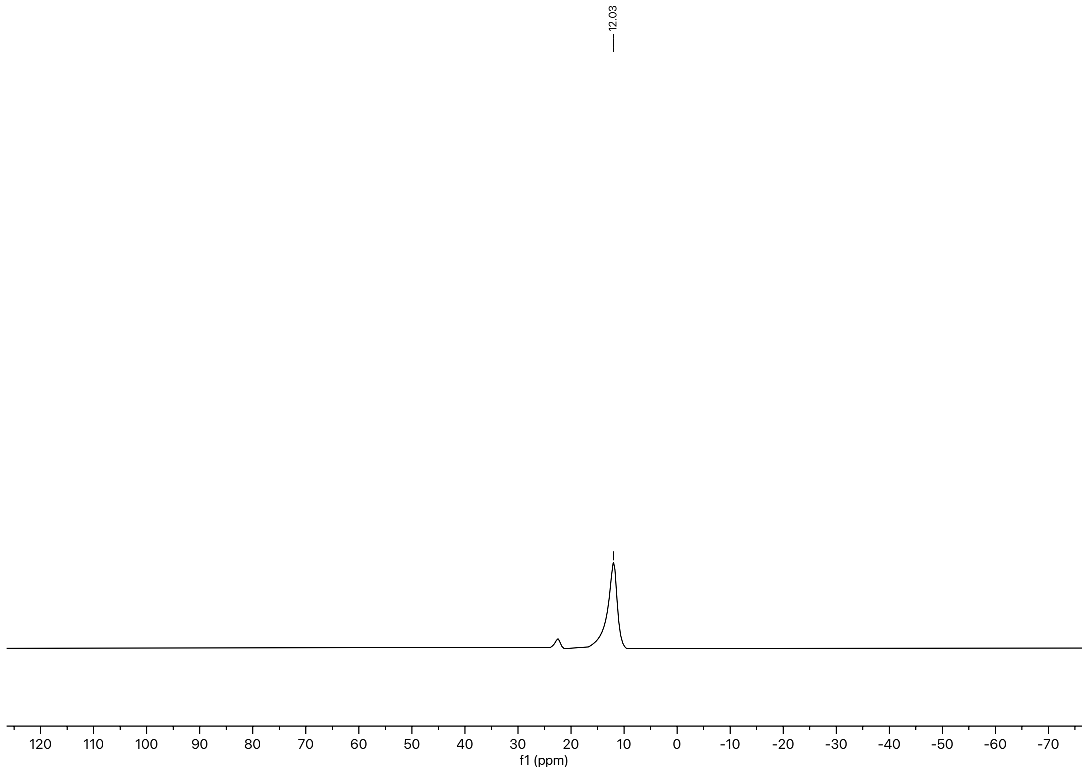

^{1}H NMR (2aj)

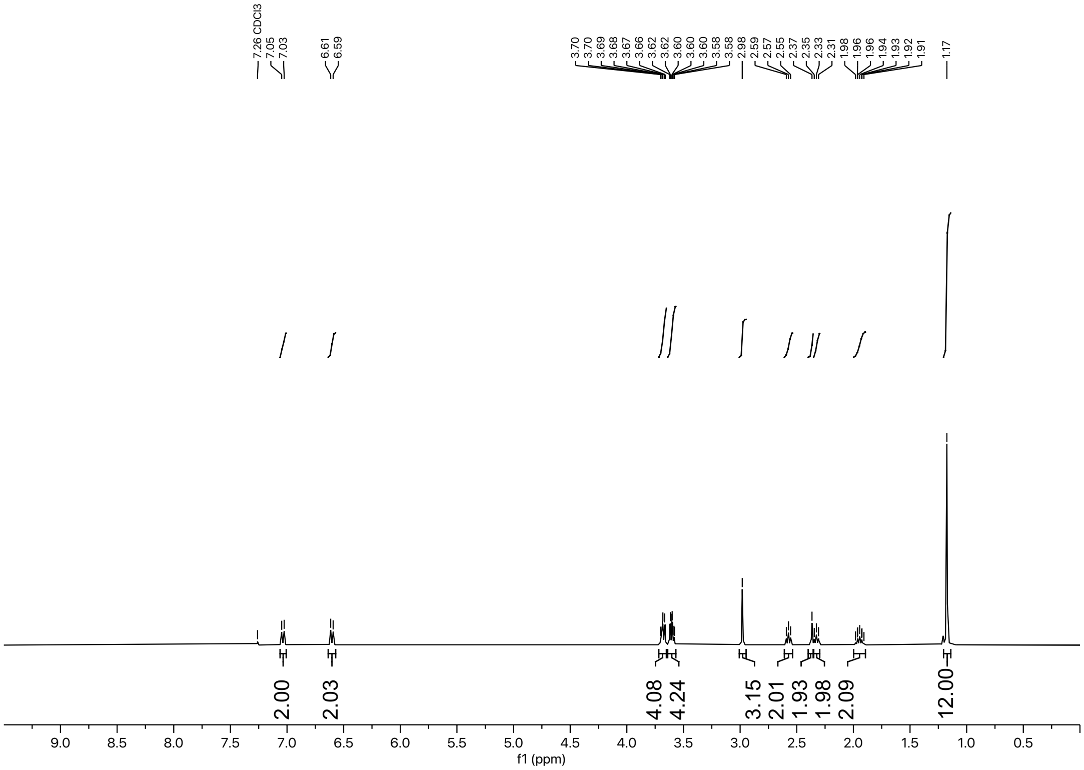

^{13}C NMR (2aj)

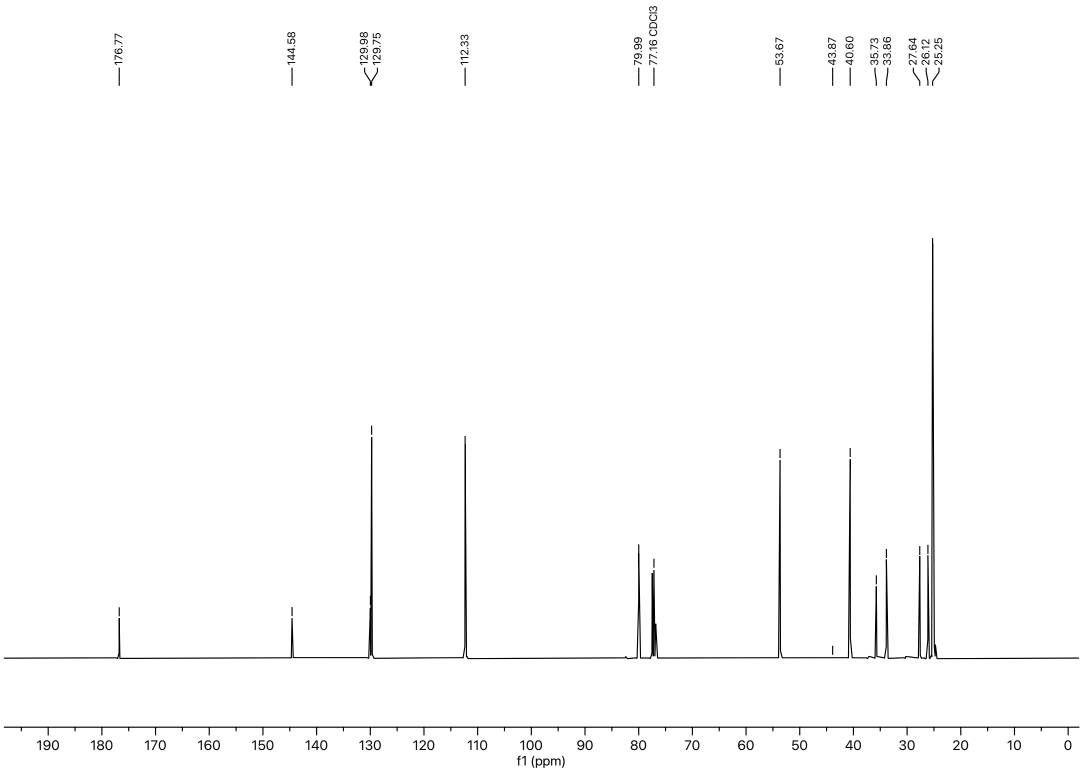

^{13}C DEPT-135 (2aj)

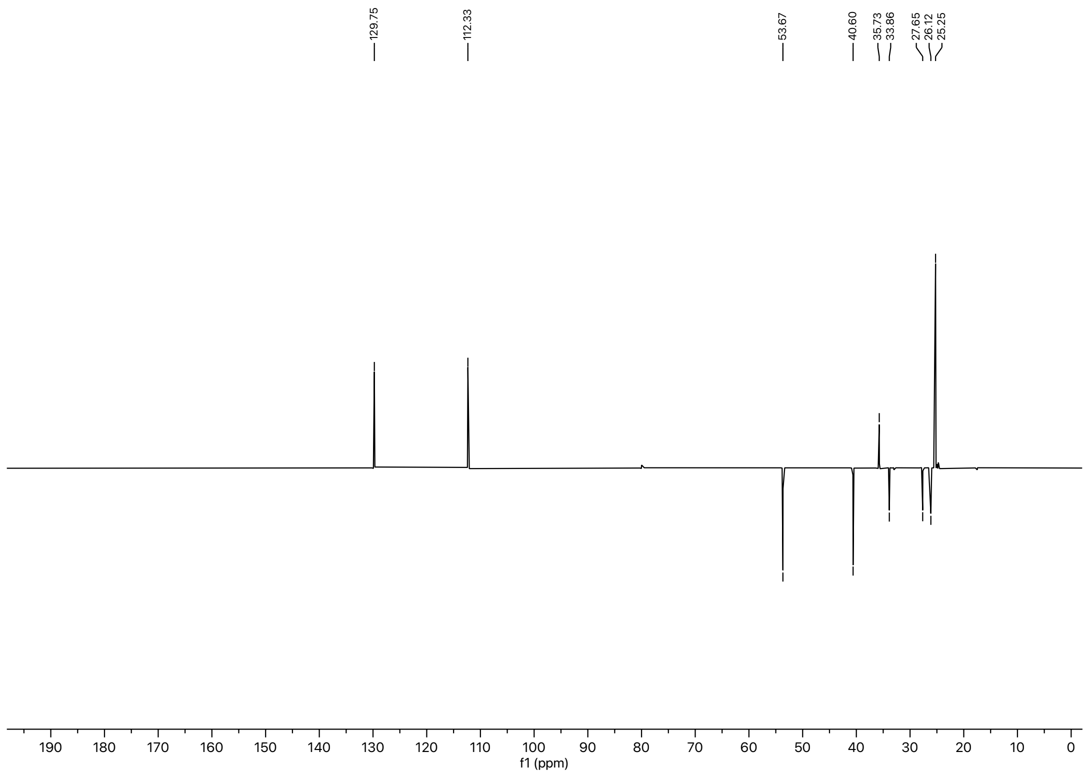

^{11}B NMR (2aj)

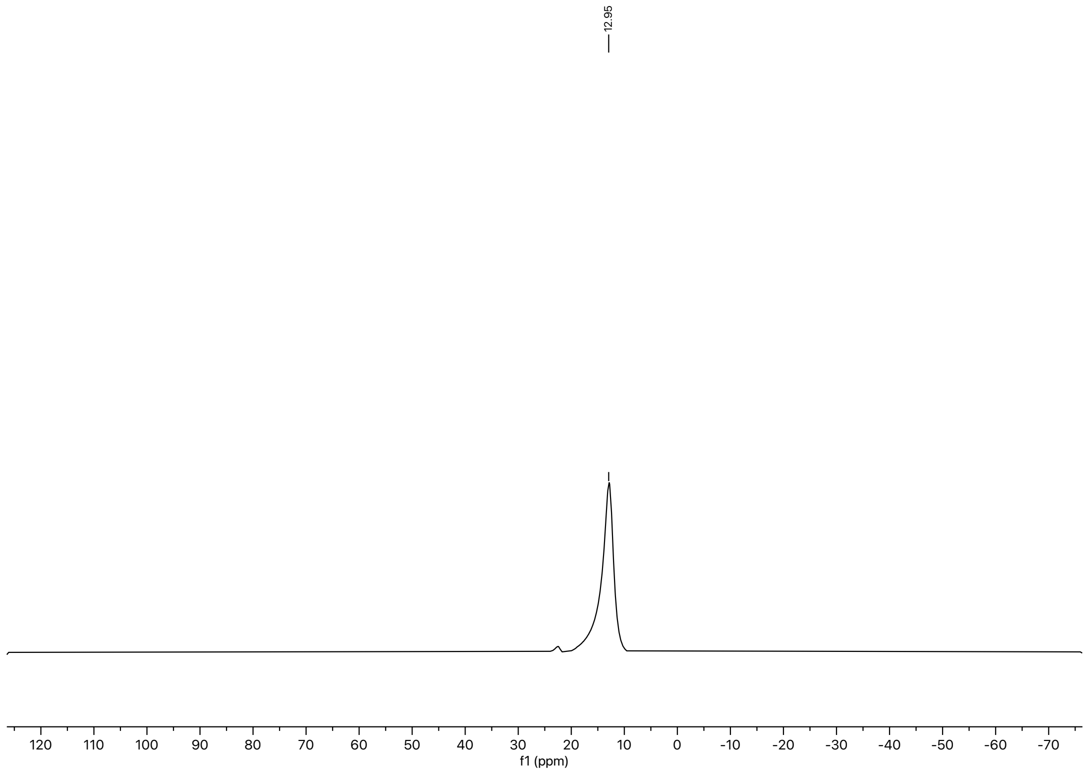

^{1}H NMR (2ak)

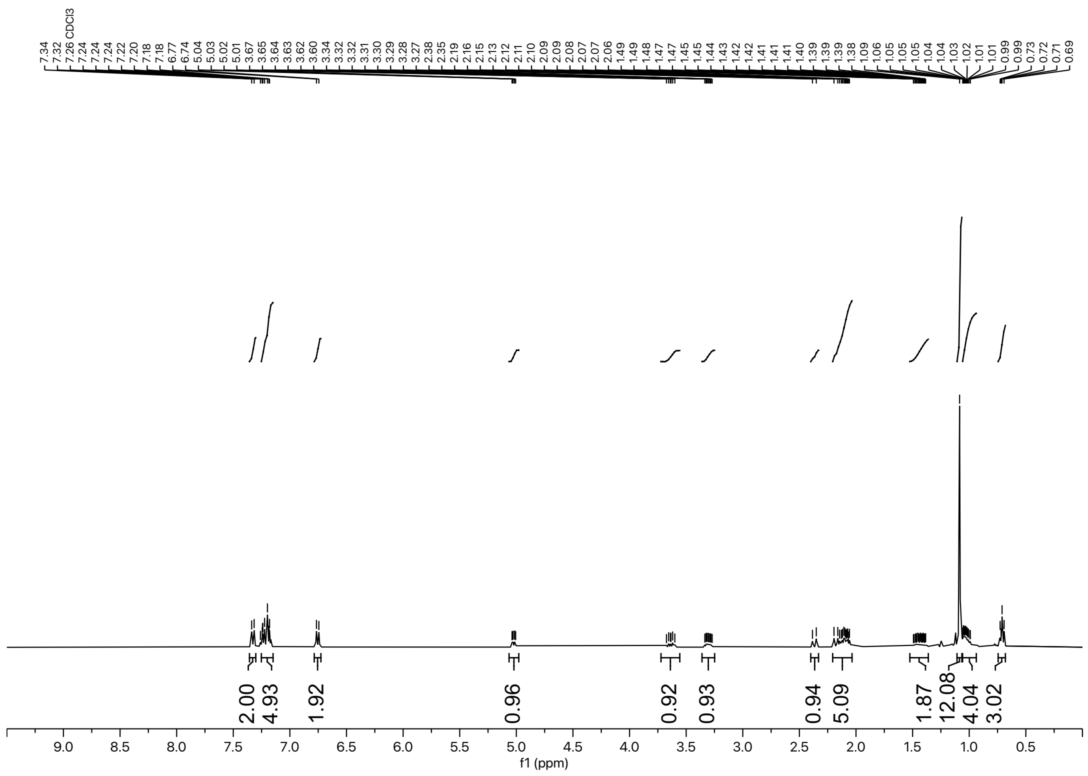

^{13}C NMR (2ak)

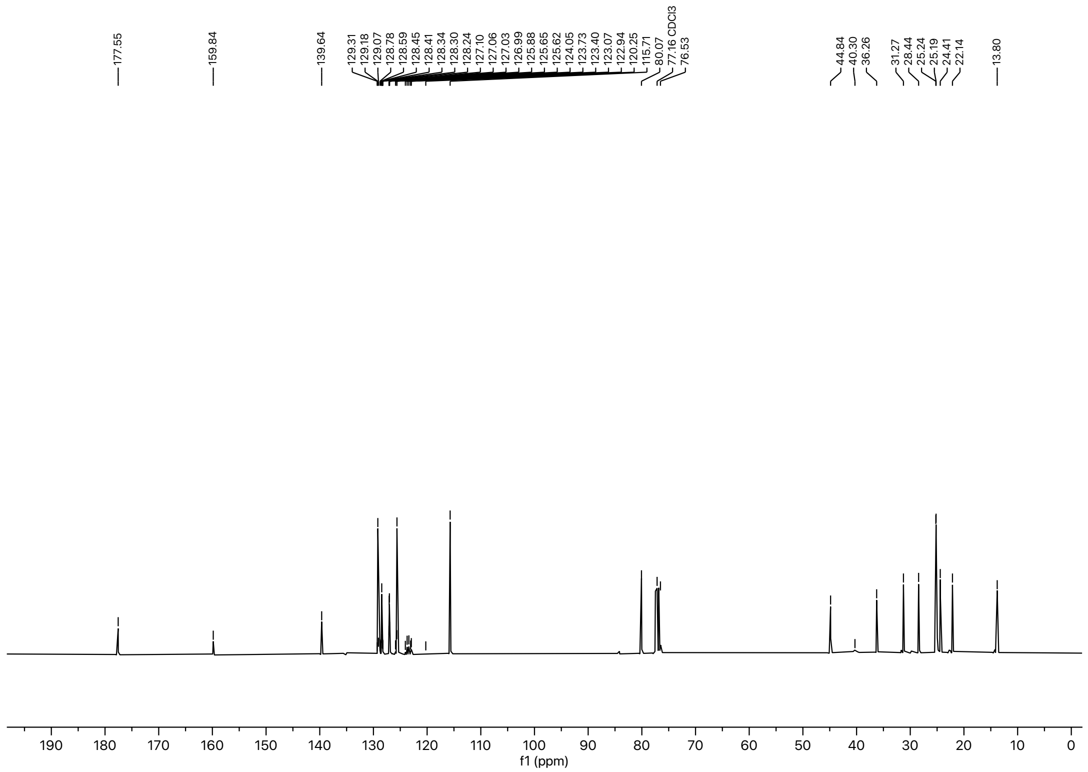

^{13}C DEPT-135 (2ak)

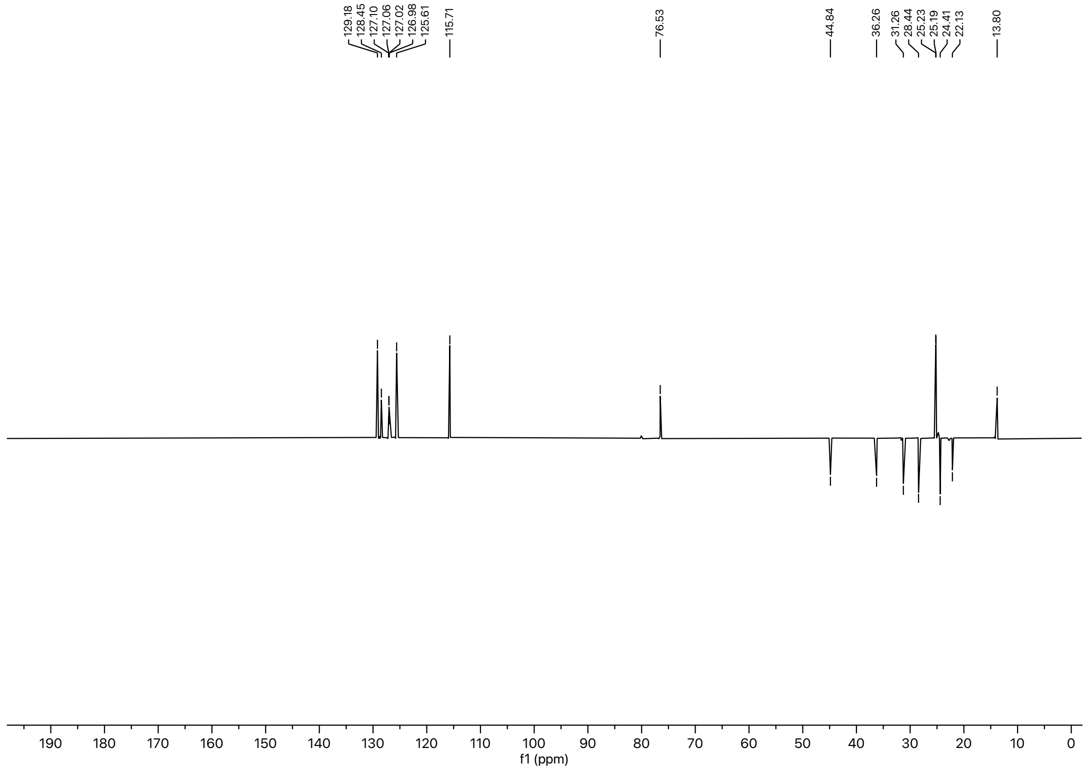

^{19}F NMR (2ak)

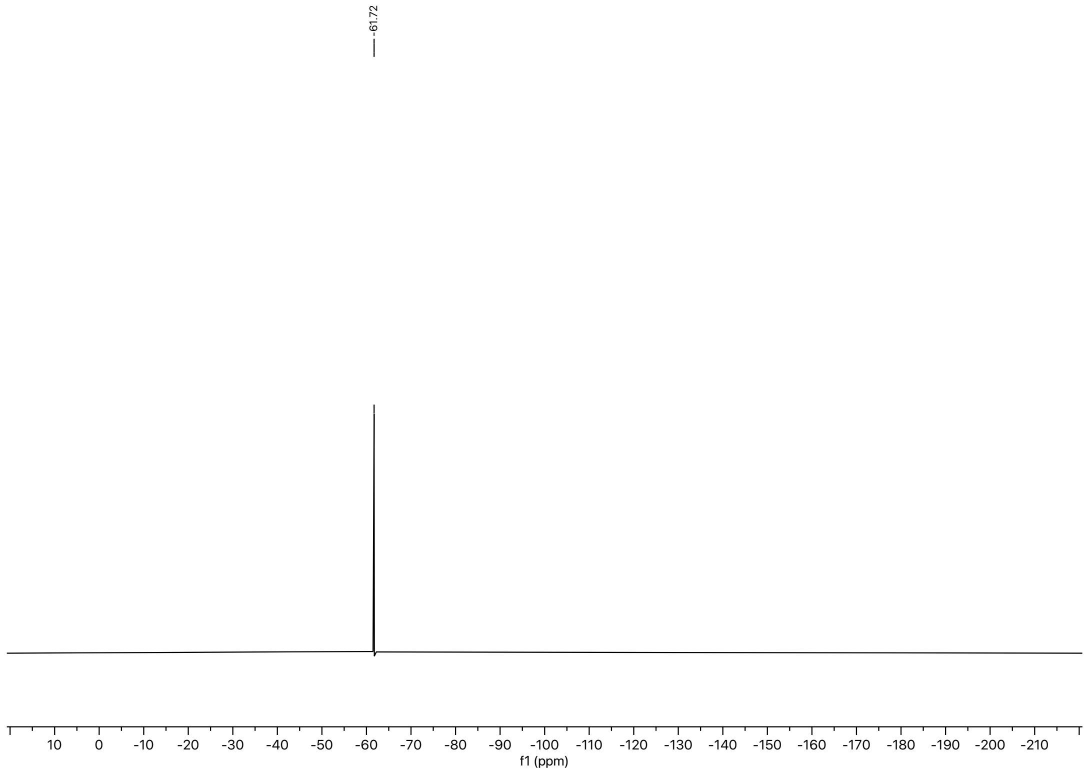

^{11}B NMR (2ak)

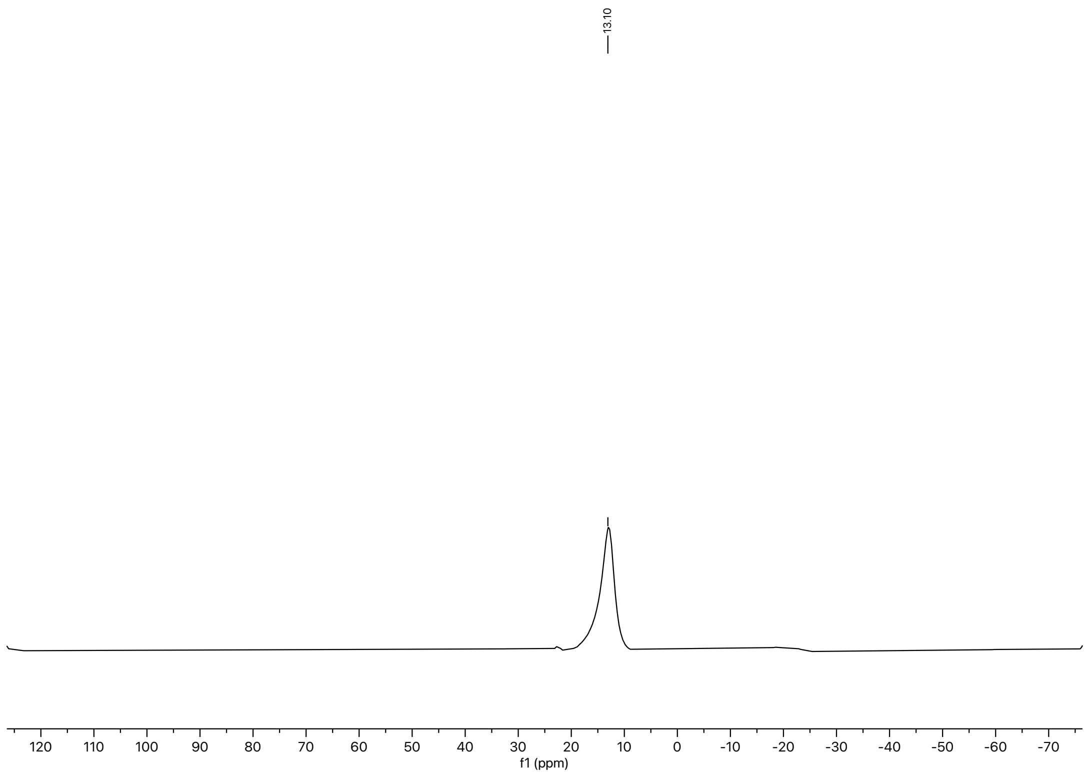

13.10
120
110
100
90
80
70
60
50
40
30
20
10
0
-10
-20
-30
-40
-50
-60
-70
f1 (ppm)

Cartesian coordinates of calculated structures

Cartesian coordinates of the lowest energy structures calculated for borylation reactions with SMD_{THF}/M06/6-311G(d,p);SDD(Ir)]//M06/6-31G(d);LANL2DZ(Ir)

Structure **L1_IrB5**

```
 Ir   -0.011146  -0.273982  -0.174917
 B    -2.025776  -0.971560  -0.079059
 C     0.538263  -0.714641  -3.308171
 C     0.979002  -1.358420  -4.455999
 C     1.172217  -3.359268  -3.159235
 C     0.716930  -2.647227  -2.049383
 C     0.596900  -3.257938  -0.711348
 C     0.690565  -4.635395  -0.507754
 C     0.415476  -4.272991   1.838577
 C     0.323398  -2.914287   1.571657
 B     2.204114  -0.361589   0.049323
 N     0.410147  -1.341674  -2.138685
 N     0.402460  -2.419347   0.329504
 O     2.918245  -0.243811   1.222189
 O     2.941331  -1.031595  -0.911629
 O    -1.247326   1.530454   2.017906
 O    -0.031241  -0.211458   2.836404
 O     1.836591   1.977693  -1.405396
 O     1.347186   2.354291   0.787985
 C     1.303745  -2.706716  -4.375376
 C     0.595823  -5.149638   0.776549
 C     4.269504  -1.243125  -0.411406
 C     4.079260  -1.088504   1.129831
 C     2.250821   3.397770   0.427148
 C     2.274893   3.322534  -1.137612
 C    -1.454996   1.521614   3.429229
 C    -0.253304   0.677191   3.940411
 C    -3.542974  -2.676249   0.048969
 B     1.191576   1.478540  -0.270063
 H     0.280253   0.342303  -3.305558
 B    -0.471668   0.441430   1.675053
 C    -4.253716  -1.324840   0.321464
 O    -3.141281  -0.444758   0.556065
 O    -2.316577  -2.247786  -0.558196
 C    -5.005622  -0.807002  -0.895824
 H    -5.345563   0.217615  -0.693548
 H    -4.351856  -0.781846  -1.777748
 H    -5.890499  -1.418129  -1.119220
 C    -5.161374  -1.301157   1.534582
 H    -5.581142  -0.293721   1.657219
 H    -5.996633  -2.006270   1.412820
 H    -4.623279  -1.550656   2.455955
 C    -3.171723  -3.417705   1.327731
 H    -4.050500  -3.851490   1.824242
 H    -2.479371  -4.232313   1.073421
 H    -2.659961  -2.750877   2.037673
 C    -4.267002  -3.610975  -0.898109
 H    -3.683553  -4.532569  -1.023986
 H    -5.254984  -3.886534  -0.501445
 H    -4.397904  -3.159053  -1.887152
 C    -1.479716   2.953250   3.927994
 H    -2.364629   3.466788   3.529911
 H    -1.528641   2.991547   5.026364
 H    -0.593583   3.502808   3.590799
 C    -2.802027   0.855753   3.681621
 H    -3.566681   1.382280   3.095435
 H    -2.792350  -0.187062   3.337045
 H    -3.087764   0.884518   4.742499
 C     1.023978   1.490753   4.111017
 H     0.971648   2.162424   4.979263
 H     1.863007   0.796537   4.253791
 H     1.230454   2.074263   3.204221
 C    -0.525593  -0.135629   5.190134
 H     0.374421  -0.699382   5.470080
 H    -0.787502   0.519881   6.033367
 H    -1.342365  -0.851295   5.039935
 C     1.716045   4.699281   0.996805
 H     1.749230   4.653445   2.093899
 H     2.323240   5.558089   0.675524
 H     0.672977   4.861461   0.701208
 C     1.281835   4.266701  -1.794209
 H     1.573687   5.318257  -1.669897
 H     1.244615   4.049931  -2.871063
 H     0.283220   4.115207  -1.374744
 C     3.640393   3.529856  -1.765789
 H     3.560270   3.431541  -2.856027
 H     4.019032   4.538119  -1.543063
 H     4.377432   2.798548  -1.417490
 C     3.591506   3.072737   1.072156
 H     3.442568   2.940502   2.152042
 H     3.992449   2.128262   0.687969
 H     4.330281   3.870183   0.913376
 C     5.230733  -0.426334   1.860625
 H     4.978968  -0.329486   2.924706
 H     6.144045  -1.033551   1.779443
 H     5.444947   0.576418   1.476037
 C     5.155645  -0.171353  -1.027102
 H     6.210730  -0.301215  -0.750704
 H     5.070232  -0.227359  -2.119384
 H     4.833299   0.832239  -0.723246
 C     4.740439  -2.612095  -0.865962
 H     4.834806  -2.620794  -1.959775
 H     5.723349  -2.855417  -0.437235
 H     4.030630  -3.399485  -0.585004
 C     3.730570  -2.397429   1.825690
 H     4.603103  -3.059180   1.911999
 H     3.362068  -2.168515   2.834527
 H     2.936403  -2.938131   1.295808
 B    -1.186819   1.221475  -1.006060
 O    -2.000835   0.920154  -2.108555
 O    -1.322978   2.563462  -0.684255
 C    -2.557983   2.149872  -2.601485
 C    -2.496856   3.057562  -1.344690
```

C	-1.651645	2.622917	-3.731428
C	-3.951773	1.903352	-3.140467
C	-2.353550	4.539117	-1.636188
C	-3.652860	2.847814	-0.373019
H	-0.634574	2.819098	-3.364609
H	-2.032682	3.530613	-4.218710
H	-1.595647	1.829273	-4.488864
H	-3.906015	1.210438	-3.991322
H	-4.399315	2.844241	-3.492512
H	-4.611495	1.468084	-2.383791
H	-2.198675	5.082173	-0.694172
H	-3.272384	4.924424	-2.101562
H	-1.513695	4.766813	-2.300791
H	-3.743870	1.790508	-0.092022
H	-4.607185	3.213072	-0.777839
H	-3.427461	3.402892	0.546797
H	0.191217	-2.171395	2.359159
H	1.664626	-3.245804	-5.249266
H	1.447224	-4.407418	-3.073750
H	0.661081	-6.223375	0.942156
H	0.817325	-5.306377	-1.353044
H	0.342478	-4.626431	2.864023
H	1.072995	-0.805480	-5.387197

Structure **L1_IrB3**

C	2.248372	3.052522	-0.329225
H	1.223371	3.395268	-0.177635
C	3.312702	3.928626	-0.503817
C	4.582723	3.399741	-0.691503
H	5.439980	4.052543	-0.844869
C	4.751990	2.021722	-0.682106
C	4.956970	-0.958191	-0.410217
C	4.957530	-2.343607	-0.325993
H	5.898834	-2.889411	-0.312195
C	3.744774	-3.017105	-0.247549
C	2.572398	-2.274005	-0.263389
H	1.590667	-2.738603	-0.176556
C	3.739753	-0.277592	-0.427632
C	3.643735	1.198926	-0.487474
C	-2.164882	3.337952	0.434128
C	-3.025908	2.447116	-0.505424
C	-2.453086	3.093111	1.910127
H	-2.413453	2.019884	2.137309
H	-3.431124	3.494488	2.209575
H	-1.678169	3.593223	2.506243
C	-2.220728	4.822919	0.134799
H	-1.585071	5.368868	0.844545
H	-3.246677	5.204644	0.240531
H	-1.868902	5.048238	-0.878349
C	-4.417689	2.136122	0.010319
H	-4.952640	1.516625	-0.722100
H	-4.999687	3.056858	0.163629
H	-4.376356	1.580209	0.953678
C	-3.107386	2.985496	-1.928763
H	-2.109271	3.229621	-2.315206
H	-3.742861	3.879242	-1.998227
H	-3.534384	2.207109	-2.574230
C	-1.996618	-1.893788	-2.771616
C	-1.935034	-2.882150	-1.573015
C	-3.266449	-1.051909	-2.779729
H	-3.429013	-0.579286	-1.803128
H	-4.149567	-1.645082	-3.055332
H	-3.143340	-0.249274	-3.518912
C	-1.792547	-2.531560	-4.131921
H	-1.849240	-1.761424	-4.911849
H	-2.573790	-3.278518	-4.336098
H	-0.813564	-3.017290	-4.211558
C	-3.273841	-3.437957	-1.129680
H	-3.129379	-4.130776	-0.289449
H	-3.759410	-3.993301	-1.945411
H	-3.946609	-2.639771	-0.797777
C	-0.947475	-4.023337	-1.787760
H	0.020761	-3.642792	-2.141580
H	-1.316678	-4.764862	-2.509335
H	-0.785671	-4.527628	-0.824997
B	-0.941256	1.513641	-0.247077
B	-0.678636	-1.018785	-1.115401
N	2.410551	1.724085	-0.325420
N	2.569428	-0.941306	-0.361259
O	-0.839347	2.851173	0.176456
O	-2.270398	1.235481	-0.552812
O	-0.899183	-1.015192	-2.494506
O	-1.399162	-2.061196	-0.529851
Ir	0.655214	0.272795	-0.349844
H	3.139528	5.001654	-0.498132
H	3.701049	-4.100375	-0.170131
B	-0.099534	-0.548393	1.293516
O	-1.220852	-0.153822	2.013603
O	0.560646	-1.585394	1.956141
C	-1.160811	-0.778558	3.300378
C	-0.288940	-2.038088	3.019783
C	-0.490401	0.208250	4.249274
C	-2.573143	-1.067732	3.768205
C	0.574691	-2.484212	4.183728
C	-1.095362	-3.219054	2.493887
H	0.536999	0.426416	3.929494
H	-0.467264	-0.162919	5.283009
H	-1.052721	1.150827	4.234824
H	-3.108101	-0.122232	3.930911
H	-2.568824	-1.622650	4.717738
H	-3.130693	-1.645112	3.022933
H	1.152962	-3.371911	3.895659
H	-0.045760	-2.752715	5.051202
H	1.282611	-1.704975	4.487042
H	-1.735332	-2.915857	1.657083
H	-1.708197	-3.677370	3.282493
H	-0.393862	-3.977555	2.118661
H	5.740018	1.595574	-0.835894
H	5.898951	-0.416910	-0.447235

Structure **L1_IrB**

C	-2.461928	1.317413	-0.045978
C	-0.326168	2.286224	-0.033430
C	-0.870293	3.552905	-0.078694
C	-2.253088	3.721176	-0.110065
C	-3.039060	2.583864	-0.092181
C	-3.248378	0.086992	-0.019688
C	-3.137425	-2.225369	0.065212
C	-4.517647	-2.349796	0.045991
C	-5.283231	-1.188330	-0.010434
C	-4.643742	0.040963	-0.043418
H	0.747492	2.122107	-0.010550
H	-2.703257	4.709998	-0.146737
H	-4.122571	2.670944	-0.113597
H	-2.488576	-3.100049	0.108061
H	-6.369953	-1.239450	-0.028463

H	-5.229573	0.955720	-0.087588
N	-1.091857	1.158941	-0.018701
N	-2.512895	-1.043750	0.032882
Ir	-0.324195	-0.614298	0.036640
O	2.406443	0.953760	-0.100590
B	1.662303	-0.226432	0.022883
O	2.542528	-1.313630	0.127871
C	3.781417	0.650664	0.171295
C	3.861521	-0.857414	-0.195593
C	4.073000	-1.094675	-1.686108
H	3.900795	-2.156987	-1.899902
H	3.360673	-0.511532	-2.283945
H	5.093460	-0.840002	-2.003903
C	4.876145	-1.654204	0.600421
H	4.863465	-2.701461	0.272031
H	5.892404	-1.262992	0.445404
H	4.652675	-1.636337	1.672612
C	4.019778	0.913482	1.653193
H	3.403385	0.251619	2.274859
H	5.074013	0.778597	1.930940
H	3.735862	1.949333	1.878434
C	4.665627	1.557783	-0.661266
H	5.724641	1.283955	-0.547547
H	4.404185	1.512476	-1.724039
H	4.549292	2.597794	-0.329255
H	-4.978898	-3.333645	0.074219
H	-0.195672	4.405562	-0.089765

Structure **L1_IrB3_RC**

C	2.542463	-1.224014	-1.020316
C	1.587314	-2.588970	0.597037
C	2.431599	-3.644235	0.272685
C	3.354799	-3.468277	-0.751116
C	3.408914	-2.246085	-1.407200
C	2.554641	0.114079	-1.649670
C	1.463975	2.146837	-1.873595
C	2.481919	2.668280	-2.658977
C	3.575540	1.861378	-2.942086
C	3.611332	0.569344	-2.436976
H	0.818088	-2.667265	1.367666
H	4.024755	-4.274909	-1.042618
H	4.106511	-2.096749	-2.228129
H	0.583898	2.732265	-1.604485
H	4.403772	2.237475	-3.538542
H	4.476754	-0.064141	-2.620319
N	1.644223	-1.406974	-0.030033
N	1.497812	0.905486	-1.380627
Ir	-0.085388	0.109290	0.037321
O	-1.052828	-1.824167	2.158958
O	-0.795914	-1.310512	-2.445652
O	-1.518956	2.761380	0.093169
B	-1.142084	-1.119560	-1.110348
B	-1.290310	-0.612662	1.490023
B	-1.598375	1.402686	-0.259083
O	-2.801505	1.167914	-0.919677
O	-2.404007	0.008783	2.055177
C	-3.083348	-0.944803	2.879066
C	-1.924157	-1.893911	3.294620
C	-2.617375	3.452202	-0.520000
C	-3.647759	2.308031	-0.746105
C	-1.501626	-2.474935	-2.902057
C	-2.736167	-2.503446	-1.956057
O	-2.191484	-1.947936	-0.750613
C	-1.139274	-1.379737	4.495600
H	-0.848361	-0.330028	4.352878
H	-1.709790	-1.462086	5.430750
H	-0.222221	-1.973888	4.600172
C	-2.333120	-3.336000	3.519361
H	-3.082898	-3.412135	4.320368
H	-2.746881	-3.778568	2.606336
H	-1.459311	-3.930001	3.818840
C	-3.759102	-0.220234	4.026700
H	-4.213533	-0.934031	4.729372
H	-3.056831	0.415489	4.577413
H	-4.559364	0.422585	3.636187
C	-4.130906	-1.626989	2.006503
H	-4.788968	-0.853678	1.588057
H	-3.658089	-2.148893	1.164978
H	-4.748188	-2.332689	2.579684
C	-1.830065	-2.319393	-4.372274
H	-2.443787	-3.160552	-4.726211
H	-2.369170	-1.386001	-4.566266
H	-0.903969	-2.304004	-4.961476
C	-0.568366	-3.662478	-2.695341
H	-0.332323	-3.796376	-1.630036
H	-0.993989	-4.598738	-3.081411
H	0.371781	-3.466208	-3.227352
C	-3.861223	-1.580734	-2.406895
H	-4.555287	-1.448406	-1.564817
H	-3.478998	-0.585787	-2.665488
H	-4.414442	-1.997009	-3.260146
C	-3.281504	-3.885294	-1.656879
H	-3.608047	-4.386891	-2.579499
H	-2.535980	-4.515165	-1.158365
H	-4.152363	-3.803332	-0.992837
C	-4.523273	2.039768	0.471658
H	-5.071429	1.103508	0.296447
H	-3.913564	1.898211	1.373101
H	-5.253369	2.843760	0.638958
C	-4.519167	2.466259	-1.977431
H	-5.205697	1.612341	-2.051469
H	-5.124575	3.382646	-1.918724
H	-3.923523	2.496599	-2.897028
C	-2.100472	4.049190	-1.823473
H	-1.766130	3.260867	-2.512048
H	-2.861181	4.656395	-2.332606
H	-1.242162	4.698686	-1.599891
C	-3.080156	4.560192	0.404299
H	-3.320986	4.175787	1.401156
H	-2.282850	5.308204	0.513853
H	-3.967352	5.067781	-0.001635
H	0.245902	1.777663	1.734077
C	3.231342	2.433588	1.098494
O	3.981542	3.047840	0.349359
C	3.602282	1.061310	1.637778
H	3.701268	1.113681	2.735277
H	2.773936	0.355800	1.452028
C	1.063092	2.242331	2.326020
H	1.538598	1.474446	2.940752
H	0.579996	2.964598	2.998749
N	2.016410	2.933979	1.486913
C	1.554180	4.200674	0.954941
H	0.557559	4.069232	0.506255
H	1.477149	4.951388	1.756124
H	2.272448	4.548830	0.208492
C	4.884101	0.516725	1.032253

H	4.788969	0.496836	-0.067004
H	5.708550	1.212643	1.241560
C	5.201179	-0.884429	1.534669
H	5.394664	-0.856076	2.621506
H	4.309456	-1.522290	1.403694
C	6.383627	-1.535472	0.826993
H	6.471315	-2.581830	1.158708
H	6.170089	-1.578882	-0.255864
C	7.704194	-0.816738	1.057573
H	7.694683	0.196798	0.635998
H	7.916021	-0.723063	2.132416
H	8.543433	-1.355074	0.599546
H	2.420821	3.690709	-3.022166
H	2.353411	-4.586043	0.810052

Structure **L1_IrB3_TS-OA**

C	2.479392	-1.817238	0.901747
C	2.066207	0.161267	2.043790
C	3.070170	-0.099856	2.961645
C	3.803634	-1.274793	2.834615
C	3.495984	-2.146102	1.802604
C	2.081339	-2.715253	-0.197917
C	0.474902	-3.220767	-1.791234
C	1.180059	-4.334319	-2.224124
C	2.396599	-4.627904	-1.616958
C	2.851326	-3.811445	-0.593505
H	1.439488	1.052759	2.106506
H	4.595857	-1.518767	3.539721
H	4.027086	-3.090150	1.711298
H	-0.490611	-2.940174	-2.212781
H	2.987944	-5.482625	-1.939223
H	3.808895	-4.013827	-0.120177
N	1.787514	-0.664068	1.023153
N	0.913808	-2.426093	-0.808591
Ir	0.052401	-0.362478	-0.339274
O	-0.886983	2.510485	-0.746881
O	-2.449212	-0.352108	1.535254
O	-2.365554	-1.856478	-1.370249
B	-1.124803	-0.730224	1.343252
B	-0.463806	1.537800	0.136794
B	-1.842282	-0.585846	-1.114915
O	-2.714915	0.389835	-1.554523
O	-0.356283	2.080985	1.424771
C	-0.592229	3.499282	1.333660
C	-1.409861	3.603854	0.016208
C	-3.492501	-1.690287	-2.245946
C	-3.945871	-0.234274	-1.933137
C	-2.903960	-0.910854	2.775404
C	-1.572309	-1.103600	3.554077
O	-0.633919	-1.342062	2.491751
C	-2.894850	3.326757	0.220513
H	-3.400513	4.156120	0.734590
H	-3.358132	3.187462	-0.764620
H	-3.048573	2.398225	0.790307
C	-1.205397	4.885131	-0.766668
H	-0.169359	4.980369	-1.112538
H	-1.852552	4.877610	-1.653465
H	-1.470408	5.764981	-0.162109
C	-1.322809	3.947467	2.583353
H	-2.219564	3.342190	2.763623
H	-0.663946	3.846585	3.456791
H	-1.621682	5.002788	2.506057
C	0.761933	4.188029	1.242522
H	1.393771	3.844911	2.074323
H	1.268222	3.958922	0.296045
H	0.666085	5.279846	1.316428
C	-3.885299	0.056124	3.406387
H	-4.204475	-0.292655	4.399344
H	-3.449310	1.057038	3.507936
H	-4.778155	0.142764	2.772592
C	-3.595098	-2.230081	2.451892
H	-2.904699	-2.923170	1.952821
H	-3.999554	-2.714338	3.350987
H	-4.431860	-2.040354	1.766673
C	-1.112494	0.158366	4.272840
H	-1.741464	0.390338	5.143225
H	-0.083610	0.001922	4.625303
H	-1.106487	1.017942	3.588839
C	-1.551821	-2.281938	4.506430
H	-1.718044	-3.229574	3.982524
H	-0.574631	-2.338369	5.003246
H	-2.321111	-2.170130	5.284456
C	-4.528604	0.519603	-3.113063
H	-3.805016	0.617969	-3.930065
H	-5.429765	0.018528	-3.495643
H	-4.812969	1.531384	-2.796224
C	-4.893022	-0.141616	-0.744905
H	-5.889343	-0.538895	-0.982529
H	-4.480939	-0.671811	0.121166
H	-4.997802	0.914445	-0.462744
C	-4.520054	-2.760738	-1.941362
H	-5.430936	-2.611088	-2.538929
H	-4.112739	-3.750309	-2.186567
H	-4.792894	-2.762802	-0.880106
C	-2.971364	-1.854851	-3.669295
H	-2.202049	-1.102021	-3.896359
H	-2.515491	-2.849369	-3.769103
H	-3.770495	-1.774118	-4.418050
H	-0.159615	0.126204	-1.886926
C	2.206221	2.626215	-1.883260
O	2.080520	3.816248	-2.151619
C	3.156710	2.171238	-0.795835
H	3.379055	3.057044	-0.187243
H	2.653246	1.446118	-0.140063
C	1.474517	0.262974	-2.138735
H	1.371642	-0.376183	-3.028979
H	2.400575	-0.041502	-1.642568
N	1.530102	1.638425	-2.556669
C	0.526501	2.027237	-3.525414
H	-0.480269	1.818394	-3.129172
H	0.616951	3.100187	-3.708002
H	0.674053	1.473645	-4.463880
C	4.465898	1.575681	-1.320602
H	4.275482	0.918086	-2.184317
H	5.106892	2.386827	-1.700096
C	5.194075	0.785961	-0.244324
H	5.304927	1.407530	0.662161
H	4.560962	-0.068807	0.060086
C	6.556967	0.266827	-0.672679
H	6.450833	-0.303410	-1.610114
H	7.213789	1.118321	-0.912022
C	7.200511	-0.605250	0.392719
H	7.303941	-0.057160	1.340355
H	6.583360	-1.492812	0.596401
H	8.198111	-0.953641	0.097843
H	0.781953	-4.952575	-3.024814

H	3.263879	0.607184	3.764302

Structure **L1_IrB3_IC**

C	-1.593113	-2.461367	-0.690397
C	0.108336	-2.915848	-2.193388
C	-0.455884	-4.136072	-2.541772
C	-1.645180	-4.512668	-1.930980
C	-2.219649	-3.669984	-0.991151
C	-2.122999	-1.510353	0.306749
C	-1.856374	0.531480	1.397676
C	-2.999466	0.330107	2.157405
C	-3.721687	-0.844126	1.976384
C	-3.271185	-1.773800	1.053799
H	1.048943	-2.568071	-2.617615
H	-2.125888	-5.455792	-2.183529
H	-3.155785	-3.948922	-0.515996
H	-1.229243	1.416426	1.507586
H	-4.629080	-1.035696	2.547507
H	-3.825250	-2.694941	0.899021
N	-0.451017	-2.102275	-1.297711
N	-1.434509	-0.361422	0.487973
Ir	0.297568	-0.003600	-0.778637
O	3.368405	0.244292	-0.156773
O	1.370940	-2.116307	1.333144
O	0.381328	2.603697	0.815260
B	1.178035	-0.775397	1.038378
B	2.332907	-0.475345	-0.743954
B	0.835750	1.885218	-0.296360
O	1.744609	2.655557	-1.010045
O	2.847509	-1.526349	-1.503382
C	4.267636	-1.570281	-1.295509
C	4.574015	-0.111973	-0.849253
C	1.330534	3.653657	1.055592
C	1.877540	3.930502	-0.375260
C	1.314584	-2.261891	2.764064
C	1.700072	-0.836989	3.257772
O	1.237271	-0.006327	2.182364
C	5.748346	0.045326	0.093856
H	5.619426	-0.539839	1.011419
H	6.684651	-0.266932	-0.390550
H	5.849376	1.101333	0.378885
C	4.712027	0.854166	-2.019832
H	4.661121	1.880763	-1.635213
H	5.663992	0.721589	-2.551697
H	3.887807	0.737249	-2.735332
C	4.538557	-2.600625	-0.208775
H	4.105981	-3.560602	-0.517512
H	5.614024	-2.742398	-0.035884
H	4.055291	-2.313438	0.733003
C	4.944243	-1.991557	-2.584887
H	4.638324	-1.362543	-3.427757
H	6.038115	-1.938389	-2.485925
H	4.678002	-3.029579	-2.822895
C	-0.118928	-2.645454	3.109512
H	-0.238578	-2.865715	4.178838
H	-0.822626	-1.846613	2.838283
H	-0.390898	-3.543173	2.538108
C	2.256290	-3.368755	3.192242
H	2.278711	-3.458263	4.288046
H	1.913124	-4.326465	2.780473
H	3.278469	-3.197900	2.837378
C	1.019607	-0.401319	4.540585
H	1.277185	-1.074240	5.371314
H	1.351530	0.609842	4.809229
H	-0.071058	-0.378051	4.432437
C	3.203310	-0.622969	3.369920
H	3.396743	0.445930	3.526990
H	3.637921	-1.182269	4.209399
H	3.713267	-0.913235	2.442308
C	1.032788	4.923640	-1.161432
H	1.346164	4.895891	-2.212723
H	-0.031482	4.658091	-1.120462
H	1.151444	5.951367	-0.791582
C	3.336212	4.344414	-0.422460
H	3.637980	4.513120	-1.464831
H	3.504583	5.276901	0.135974
H	3.981526	3.561254	-0.005900
C	2.387499	3.088863	1.998226
H	2.912102	2.237683	1.541381
H	3.119382	3.853213	2.294427
H	1.882743	2.715919	2.898955
C	0.622798	4.824000	1.707637
H	1.304111	5.679548	1.821426
H	-0.248174	5.147450	1.125560
H	0.274389	4.536180	2.708136
H	1.342035	0.316360	-1.979254
C	-3.464161	0.635812	-1.662805
O	-3.512025	-0.555227	-1.976874
C	-4.683028	1.313860	-1.046018
H	-4.387712	1.802260	-0.101682
H	-5.039619	2.128297	-1.698137
C	-1.100459	0.754126	-2.280005
H	-1.394028	-0.091118	-2.913340
H	-0.595457	1.495738	-2.913332
N	-2.337826	1.380666	-1.806228
C	-2.236373	2.770907	-1.426645
H	-1.723991	2.898332	-0.458382
H	-1.644859	3.303228	-2.185579
H	-3.217358	3.250608	-1.375236
C	-5.812690	0.336277	-0.774436
H	-6.218432	-0.038247	-1.726266
H	-5.405219	-0.551319	-0.266391
C	-6.917017	0.947372	0.071580
H	-6.486797	1.314069	1.022475
H	-7.330433	1.840432	-0.429487
C	-8.043161	-0.028033	0.378121
H	-8.464705	-0.404057	-0.567979
H	-7.623165	-0.911072	0.888958
C	-9.141916	0.588504	1.227251
H	-8.744671	0.947292	2.187425
H	-9.593759	1.452048	0.719416
H	-9.945203	-0.126189	1.445542
H	-3.308607	1.084768	2.876265
H	0.029811	-4.770447	-3.278931

Structure **L1_IrB3_TS-RE**

C	-2.456691	-1.380183	0.677912
C	-1.939262	-2.667736	-1.156540
C	-2.712476	-3.729193	-0.698637
C	-3.354118	-3.599331	0.526796
C	-3.223108	-2.410224	1.229827
C	-2.311989	-0.079666	1.359005
C	-1.054757	1.840376	1.725559
C	-1.966469	2.318664	2.653595
C	-3.108845	1.577177	2.915054
C	-3.267449	0.357340	2.275647

H	-1.374439	-2.734654	-2.084607
H	-3.945874	-4.415885	0.936488
H	-3.689686	-2.294368	2.205799
H	-0.138151	2.380366	1.493486
H	-3.867134	1.939203	3.605630
H	-4.157521	-0.243377	2.448435
N	-1.810730	-1.521650	-0.487183
N	-1.235438	0.685894	1.055812
Ir	0.121565	0.209312	-0.569517
O	2.676990	-1.584311	-0.768437
O	1.888386	0.141240	1.863834
O	1.344801	2.899724	0.231600
B	1.067282	-0.612616	1.049423
B	1.359988	-1.345481	-1.147536
B	1.058983	2.055392	-0.866635
O	2.081308	2.216745	-1.832503
O	0.892544	-2.379027	-1.952753
C	1.821385	-3.468513	-1.840901
C	3.146272	-2.727975	-1.501547
C	2.590418	3.565560	-0.007205
C	3.227671	2.707414	-1.143193
C	2.337233	-0.695464	2.942469
C	1.227289	-1.788752	3.000623
O	0.756943	-1.829131	1.642773
C	4.108511	-3.514093	-0.634900
H	3.654982	-3.793932	0.323727
H	4.434099	-4.431216	-1.146639
H	4.999317	-2.907560	-0.425432
C	3.868802	-2.197555	-2.732549
H	4.679079	-1.533653	-2.406118
H	4.307706	-3.005808	-3.332747
H	3.190960	-1.612599	-3.368383
C	1.329789	-4.367765	-0.713133
H	1.967107	-5.253569	-0.587558
H	1.276284	-3.819551	0.237789
H	0.315242	-4.712431	-0.955123
C	1.835271	-4.234241	-3.148245
H	2.597859	-5.026003	-3.129605
H	0.858826	-4.711112	-3.306949
H	2.031339	-3.577173	-4.002359
C	3.701327	-1.235309	2.538751
H	3.631188	-1.812964	1.608371
H	4.369039	-0.384337	2.347609
H	4.150420	-1.857153	3.324945
C	2.460797	0.149512	4.194726
H	1.534241	0.694626	4.408516
H	2.714373	-0.472508	5.065436
H	3.260944	0.889036	4.063064
C	1.712152	-3.169205	3.393998
H	0.870916	-3.874606	3.383404
H	2.473942	-3.541813	2.698913
H	2.138753	-3.162626	4.407409
C	0.035021	-1.391686	3.862918
H	-0.780139	-2.106031	3.684129
H	0.276847	-1.404368	4.934041
H	-0.331836	-0.389302	3.599934
C	4.090443	3.488428	-2.116913
H	4.936823	3.963467	-1.599655
H	4.497677	2.804980	-2.873046
H	3.517840	4.262783	-2.639256
C	4.013774	1.510174	-0.617252
H	4.959533	1.816672	-0.147731
H	3.428890	0.922794	0.101637
H	4.245092	0.852266	-1.465629
C	3.380320	3.583836	1.288038
H	3.460360	2.574609	1.708992
H	4.390195	3.989580	1.129661
H	2.872636	4.220644	2.025049
C	2.278466	4.989448	-0.448786
H	3.191522	5.584985	-0.584669
H	1.714733	4.996902	-1.391421
H	1.665554	5.479847	0.319283
H	1.125696	0.000048	-1.860060
C	-2.932286	2.646300	-0.842441
O	-3.731020	3.275589	-0.154347
C	-3.366203	1.391013	-1.575146
H	-3.312644	1.582247	-2.661261
H	-2.660434	0.564363	-1.396669
C	-0.590371	2.300933	-1.683773
H	-1.039930	1.533224	-2.330630
H	-0.087962	2.961053	-2.407283
N	-1.630536	3.053410	-1.001722
C	-1.274614	4.377665	-0.526322
H	-0.487801	4.330777	0.234161
H	-0.897488	4.983445	-1.365522
H	-2.172093	4.842942	-0.113132
C	-4.766349	0.955914	-1.180709
H	-4.810972	0.899939	-0.082535
H	-5.493655	1.734689	-1.456338
C	-5.154602	-0.376183	-1.804207
H	-5.084210	-0.297523	-2.901795
H	-4.417005	-1.146317	-1.511962
C	-6.550734	-0.852706	-1.418838
H	-7.292518	-0.101745	-1.735945
H	-6.786370	-1.768767	-1.981331
C	-6.707574	-1.124691	0.070631
H	-6.624887	-0.203631	0.663263
H	-7.681969	-1.574246	0.300091
H	-5.927147	-1.817736	0.422829
H	-1.781421	3.273632	3.138088
H	-2.793496	-4.639976	-1.287776

Structure **L1_IrB3_PC**

C	0.493700	3.504253	-0.505411
C	2.124931	1.856494	-0.719999
C	3.138247	2.799184	-0.850665
C	2.791044	4.141598	-0.811409
C	1.458452	4.499331	-0.641058
C	-0.944074	3.788790	-0.314402
C	-3.074276	2.898879	-0.062700
C	-3.641051	4.162600	0.019213
C	-2.814970	5.275725	-0.065958
C	-1.451542	5.084228	-0.237412
H	2.355291	0.792748	-0.751162
H	3.550656	4.914900	-0.910669
H	1.180760	5.549564	-0.612103
H	-3.673000	1.992022	-0.017865
H	-3.223469	6.282186	-0.003312
H	-0.789596	5.943147	-0.309513
N	0.843085	2.197793	-0.547527
N	-1.756326	2.709198	-0.220145
Ir	-0.807077	0.751313	-0.292173
O	-3.652535	-0.023608	-1.091979
O	-2.139965	-1.491396	1.359471
O	4.684322	0.181048	-0.489442
B	-1.954704	-0.133439	1.089151

B	-2.281178	-0.328324	-1.132722
B	4.600728	-0.435978	0.806217
O	5.874955	-0.985058	1.118750
O	-2.065863	-1.397119	-1.993356
C	-3.336438	-1.943954	-2.370098
C	-4.293397	-0.735135	-2.160825
C	5.847801	-0.349250	-1.118949
C	6.783126	-0.588645	0.102352
C	-2.806978	-1.625441	2.620074
C	-3.501117	-0.239911	2.784529
O	-2.633536	0.629871	2.044247
C	-4.335186	0.208398	-3.356741
H	-4.849512	1.131811	-3.059509
H	-3.321451	0.476470	-3.682227
H	-4.874143	-0.226768	-4.208863
C	-5.703574	-1.099895	-1.742385
H	-6.293301	-0.186823	-1.585487
H	-6.200841	-1.694556	-2.522149
H	-5.716137	-1.674985	-0.808939
C	-3.262833	-2.448083	-3.798347
H	-2.893308	-1.676518	-4.482487
H	-2.577459	-3.303362	-3.856243
H	-4.251551	-2.783021	-4.144985
C	-3.623429	-3.103487	-1.424410
H	-3.643868	-2.759205	-0.381859
H	-4.570077	-3.607284	-1.663352
H	-2.812320	-3.839178	-1.511675
C	-3.746263	-2.813938	2.553643
H	-3.165813	-3.727388	2.364101
H	-4.287375	-2.942138	3.502326
H	-4.481088	-2.711310	1.746737
C	-1.734940	-1.886123	3.670304
H	-1.047027	-1.033575	3.750367
H	-2.165168	-2.080146	4.661931
H	-1.153625	-2.767856	3.368041
C	-4.871907	-0.172582	2.123196
H	-5.209758	0.872574	2.129157
H	-4.819405	-0.498163	1.076023
H	-5.618758	-0.775993	2.656883
C	-3.599677	0.255538	4.214223
H	-2.612917	0.367655	4.676753
H	-4.091250	1.236655	4.231481
H	-4.198661	-0.433720	4.827017
C	7.820264	-1.678067	-0.096832
H	8.433988	-1.774175	0.808462
H	7.348182	-2.648516	-0.284853
H	8.489597	-1.441238	-0.936965
C	7.469784	0.694468	0.565590
H	8.262837	1.020572	-0.121982
H	6.740489	1.510319	0.673508
H	7.920102	0.512388	1.549664
C	6.358968	0.658994	-2.131668
H	5.623086	0.777693	-2.939155
H	6.527247	1.643377	-1.677576
H	7.303036	0.322519	-2.583840
C	5.476832	-1.643169	-1.838070
H	4.634987	-1.440329	-2.513042
H	6.312479	-2.031876	-2.436510
H	5.159404	-2.418069	-1.130815
H	0.034647	-0.625875	-0.382548
C	2.495289	-1.379077	1.398316
O	3.528688	-1.659122	0.716489
C	1.332613	-2.310064	1.444958
H	1.419366	-2.903826	2.373047
H	0.401335	-1.731258	1.533140
C	3.816520	0.444569	1.936721
H	3.641945	1.487109	1.631903
H	4.331091	0.452501	2.908789
N	2.526265	-0.238089	2.079765
C	1.432630	0.270965	2.883250
H	1.156733	-0.430797	3.682687
H	1.761184	1.206415	3.345687
H	0.544738	0.478364	2.260320
C	1.242496	-3.218811	0.228158
H	1.299570	-2.589000	-0.672325
H	2.111185	-3.894844	0.191691
C	-0.061365	-4.001659	0.221483
H	-0.160557	-4.558309	1.170682
H	-0.897924	-3.283037	0.195223
C	-0.181641	-4.969539	-0.950013
H	0.567310	-5.772806	-0.849791
H	-1.163724	-5.466348	-0.894410
C	-0.027997	-4.288308	-2.304344
H	-0.619416	-3.360645	-2.346759
H	1.016419	-4.011856	-2.501279
H	-0.348893	-4.945511	-3.124110
H	-4.715864	4.262811	0.147514
H	4.160376	2.441642	-0.954647

Structure **L1_IrB3_IC_TS-isoH**

C	1.563196	-0.930777	2.083598
C	2.238179	0.984247	0.970327
C	3.391173	1.078234	1.729703
C	3.636281	0.120572	2.704040
C	2.710154	-0.891900	2.873205
C	0.563261	-1.992126	2.212047
C	-1.402768	-2.888545	1.403273
C	-1.444097	-3.848289	2.400776
C	-0.419471	-3.874043	3.340499
C	0.585805	-2.928967	3.250227
H	2.008742	1.724899	0.213479
H	4.538004	0.153060	3.311840
H	2.894603	-1.676427	3.600583
H	-2.202712	-2.799118	0.672205
H	-0.412496	-4.608615	4.143232
H	1.376197	-2.908263	3.994862
N	1.320847	0.014684	1.143505
N	-0.413673	-1.993623	1.286358
Ir	-0.313188	-0.180867	-0.183560
O	-0.848664	2.264797	-1.967418
O	-1.752487	0.563411	2.441906
O	-2.965959	-1.585309	-0.849942
B	-1.770747	0.682563	1.056938
B	-0.362715	1.776800	-0.760170
B	-1.769800	-1.177231	-1.410471
O	-1.586159	-1.725620	-2.659555
O	0.087946	2.832494	0.034681
C	0.097310	4.022684	-0.769259
C	-0.956434	3.692042	-1.865591
C	-3.688153	-2.320746	-1.858864
C	-2.543336	-2.786843	-2.804048
C	-2.810013	1.382709	2.964753
C	-3.805371	1.421320	1.774728
O	-2.919912	1.354556	0.647797
C	-2.382754	4.006309	-1.436185
H	-3.071991	3.565650	-2.167535

H	-2.607223	3.553520	-0.462021
H	-2.569567	5.088034	-1.392565
C	-0.674646	4.303324	-3.223506
H	-1.465651	4.016306	-3.927714
H	-0.656545	5.401199	-3.163257
H	0.280534	3.957280	-3.634495
C	-0.245979	5.209790	0.108015
H	-1.197364	5.063534	0.631944
H	0.537300	5.352375	0.863631
H	-0.314298	6.130240	-0.489691
C	1.508471	4.182346	-1.321624
H	1.782332	3.333361	-1.963249
H	1.623850	5.107309	-1.902045
H	2.215385	4.219022	-0.481395
C	-3.350673	0.750713	4.230757
H	-2.581183	0.769104	5.013345
H	-4.225848	1.303234	4.602951
H	-3.638909	-0.293857	4.066933
C	-2.202536	2.745613	3.270734
H	-1.364791	2.608842	3.965940
H	-1.803150	3.205745	2.355840
H	-2.927762	3.429561	3.732651
C	-4.700155	0.188566	1.706266
H	-4.107428	-0.732357	1.792293
H	-5.471787	0.193106	2.488392
H	-5.193793	0.170263	0.725828
C	-4.645107	2.678656	1.679204
H	-4.023970	3.578366	1.598045
H	-5.283985	2.629513	0.787572
H	-5.295930	2.783744	2.559518
C	-1.851273	-4.061929	-2.339546
H	-0.932029	-4.192218	-2.924003
H	-1.557934	-4.000182	-1.282150
H	-2.483083	-4.949068	-2.480560
C	-2.931531	-2.909918	-4.262822
H	-3.275858	-1.954374	-4.672161
H	-2.063776	-3.234986	-4.849957
H	-3.728092	-3.656734	-4.391035
C	-4.643007	-1.334619	-2.515996
H	-5.287378	-1.824266	-3.258096
H	-5.281155	-0.891616	-1.741301
H	-4.096403	-0.519148	-3.006234
C	-4.470929	-3.432270	-1.190750
H	-3.826344	-4.106096	-0.614118
H	-5.209391	-2.997541	-0.504045
H	-5.011387	-4.030805	-1.937655
H	-1.346001	0.147836	-1.435059
C	3.539048	-1.032644	-0.925587
O	3.478315	-1.929675	-0.084361
C	4.864294	-0.334797	-1.213413
H	5.113833	-0.432094	-2.283539
H	4.763757	0.750499	-1.038210
C	1.161534	-1.283470	-1.473629
H	0.751093	-1.430337	-2.480076
H	1.354547	-2.269186	-1.031540
N	2.452945	-0.620495	-1.632441
C	2.485850	0.406191	-2.645957
H	3.285626	1.134974	-2.473486
H	2.614271	-0.017617	-3.655878
H	1.522911	0.939099	-2.629206
C	5.998272	-0.877891	-0.363966
H	5.730092	-0.782835	0.700342
H	6.099329	-1.959423	-0.538356
C	7.318072	-0.174995	-0.635589
H	7.577351	-0.268185	-1.705177
H	7.207876	0.909338	-0.452369
C	8.466061	-0.711054	0.206535
H	8.206527	-0.615925	1.273724
H	8.574718	-1.791894	0.021374
C	9.780745	-0.002224	-0.072388
H	10.600679	-0.396807	0.540746
H	10.071042	-0.111742	-1.126787
H	9.699705	1.074704	0.132644
H	-2.272325	-4.551928	2.439476
H	4.086690	1.893213	1.541620

Structure **L1_IrB3_IC-isoH**

C	-2.225831	-1.485206	0.109128
C	-0.752134	-2.144079	-1.564772
C	-1.630772	-3.108183	-2.027490
C	-2.855808	-3.256120	-1.390252
C	-3.155886	-2.435672	-0.315922
C	-2.471693	-0.591406	1.252648
C	-1.588634	1.040549	2.647509
C	-2.741380	1.085122	3.415836
C	-3.807478	0.262994	3.059765
C	-3.672318	-0.584213	1.972355
H	0.207003	-1.958455	-2.042917
H	-3.575381	-3.998979	-1.728561
H	-4.112523	-2.535157	0.191047
H	-0.734729	1.687692	2.845584
H	-4.736170	0.278223	3.627132
H	-4.497979	-1.230552	1.683678
N	-1.031169	-1.362283	-0.513381
N	-1.458334	0.230836	1.590019
Ir	0.340713	0.150831	0.211567
O	1.807856	-0.401551	-2.387589
O	1.804789	-1.598321	2.417248
O	0.661985	2.961595	1.404052
B	1.307156	-1.486941	1.132575
B	1.924554	-0.120373	-1.030145
B	1.082632	2.068581	0.417009
O	2.026566	2.664415	-0.399794
O	3.260884	-0.151870	-0.643495
C	4.062743	-0.220953	-1.829396
C	3.085410	-0.865481	-2.854431
C	1.259078	4.241727	1.123857
C	2.496375	3.841450	0.271706
C	2.565119	-2.817888	2.487665
C	1.903470	-3.675010	1.375323
O	1.500576	-2.672744	0.430226
C	3.277114	-0.420496	-4.289883
H	2.537520	-0.914906	-4.932609
H	3.144835	0.661281	-4.398032
H	4.278919	-0.692202	-4.653277
C	3.060098	-2.386925	-2.777225
H	2.881788	-2.722989	-1.746095
H	2.235804	-2.757855	-3.401808
H	3.991446	-2.834949	-3.149166
C	4.454366	1.204225	-2.199012
H	3.566505	1.819126	-2.392598
H	4.987612	1.650820	-1.348927
H	5.117499	1.233227	-3.074398
C	5.306271	-1.037106	-1.536565
H	5.057183	-2.029124	-1.140978
H	5.915965	-1.165522	-2.442818

H	5.917860	-0.519654	-0.785887
C	4.009812	-2.452629	2.174196
H	4.342022	-1.692257	2.892003
H	4.681313	-3.318455	2.253606
H	4.092723	-2.017474	1.168519
C	2.457696	-3.393032	3.884569
H	2.959644	-4.369801	3.943663
H	2.943808	-2.719142	4.601379
H	1.413893	-3.516309	4.193957
C	2.825189	-4.663222	0.691503
H	2.276594	-5.201494	-0.092818
H	3.678959	-4.160895	0.221711
H	3.207587	-5.403894	1.408669
C	0.629640	-4.375171	1.834039
H	0.836948	-5.224587	2.499071
H	-0.036903	-3.674858	2.356214
H	0.097809	-4.748409	0.948478
C	2.923358	4.866661	-0.758284
H	3.194954	5.816750	-0.275921
H	3.804063	4.500867	-1.301891
H	2.131604	5.057479	-1.490988
C	3.691267	3.420336	1.118517
H	4.430792	2.941077	0.464726
H	4.167974	4.275881	1.615761
H	3.401689	2.684081	1.880355
C	1.583501	4.929821	2.434303
H	2.187277	4.290892	3.087683
H	2.130521	5.866934	2.257033
H	0.655356	5.177195	2.965758
C	0.244897	5.066326	0.343702
H	0.004551	4.598902	-0.620637
H	-0.682380	5.138718	0.927453
H	0.609193	6.085125	0.155866
H	1.444847	0.618968	1.306689
C	-3.077969	0.802660	-1.798473
O	-2.779418	-0.007233	-2.671213
C	-4.531221	1.002115	-1.387597
H	-4.613767	0.826097	-0.301358
H	-4.831503	2.053419	-1.533513
C	-0.725064	1.349430	-1.371671
H	-0.623899	0.737560	-2.271949
H	-0.283289	2.329447	-1.599469
N	-2.147782	1.532115	-1.112454
C	-2.503873	2.610405	-0.216689
H	-3.352947	2.354451	0.429267
H	-1.644244	2.816248	0.430745
H	-2.750361	3.536791	-0.762815
C	-5.473694	0.079881	-2.141898
H	-5.422587	0.317676	-3.213107
H	-5.102988	-0.954043	-2.059568
C	-6.912176	0.157612	-1.646378
H	-7.262664	1.204651	-1.668494
H	-7.566923	-0.391096	-2.342809
C	-7.122813	-0.407457	-0.246088
H	-6.736603	-1.441819	-0.218307
H	-6.525061	0.159852	0.486642
C	-8.583532	-0.390887	0.173159
H	-8.980781	0.633745	0.168291
H	-9.198529	-0.981682	-0.519831
H	-8.730530	-0.799827	1.180760
H	-2.803413	1.759831	4.265820
H	-1.358196	-3.716973	-2.885174

Structure **L1_IrB3_IC-isoH_TS-RE**

C	1.813918	-1.948451	-0.890206
C	0.993618	-2.144869	1.275994
C	1.867327	-3.180932	1.565066
C	2.751474	-3.601038	0.581838
C	2.725777	-2.977127	-0.655661
C	1.721581	-1.250984	-2.187355
C	0.623787	0.384749	-3.415870
C	1.379733	0.119676	-4.548072
C	2.352778	-0.872149	-4.475364
C	2.526704	-1.564496	-3.286691
H	0.300412	-1.745922	2.014736
H	3.460261	-4.404153	0.773937
H	3.416673	-3.290464	-1.434118
H	-0.133410	1.167617	-3.393551
H	2.971083	-1.108520	-5.339204
H	3.280488	-2.344974	-3.220716
N	0.958850	-1.549174	0.078397
N	0.791862	-0.280420	-2.269256
Ir	-0.400745	0.075183	-0.380195
O	-0.982155	-0.048753	2.587353
O	-2.770238	-1.416394	-1.776810
O	-1.161208	2.910363	0.587829
B	-1.840026	-1.387054	-0.753315
B	-1.512034	0.160942	1.315425
B	-0.551799	2.140868	-0.402963
O	-0.344600	2.921669	-1.552543
O	-2.887628	0.351492	1.393487
C	-3.238704	0.491183	2.776367
C	-2.079641	-0.261505	3.490071
C	-1.696628	4.075388	-0.046860
C	-0.719722	4.273953	-1.242298
C	-3.705749	-2.468655	-1.488996
C	-2.857909	-3.426204	-0.607128
O	-1.969088	-2.515318	0.055217
C	-1.708649	0.279794	4.855312
H	-1.387647	1.325472	4.799812
H	-2.558594	0.207117	5.549424
H	-0.878945	-0.304741	5.273251
C	-2.304744	-1.767133	3.564541
H	-1.382888	-2.243933	3.925205
H	-3.113343	-2.030389	4.259888
H	-2.531095	-2.180019	2.570823
C	-3.262548	1.981632	3.092513
H	-3.557161	2.176211	4.132789
H	-2.285496	2.441486	2.895223
H	-3.996727	2.466191	2.434461
C	-4.614791	-0.104570	2.999760
H	-4.891953	-0.066981	4.063418
H	-5.360285	0.469002	2.433567
H	-4.666994	-1.146874	2.663123
C	-4.863496	-1.833837	-0.730296
H	-5.658958	-2.558486	-0.508405
H	-4.511835	-1.378017	0.205334
H	-5.288639	-1.032657	-1.347683
C	-4.197273	-3.068313	-2.790447
H	-4.851765	-3.931538	-2.600374
H	-4.776957	-2.320510	-3.346421
H	-3.366897	-3.391763	-3.428004
C	-3.640145	-4.202756	0.432281
H	-4.381567	-4.860751	-0.043887
H	-2.956354	-4.830132	1.019302
H	-4.163396	-3.534286	1.126168

C	-1.984180	-4.374730	-1.419695
H	-1.261831	-4.850675	-0.743378
H	-2.569615	-5.163318	-1.911604
H	-1.417866	-3.826315	-2.185218
C	-1.342584	4.903047	-2.473048
H	-0.586288	5.006824	-3.262204
H	-2.161596	4.292102	-2.867498
H	-1.731495	5.905788	-2.244616
C	0.540540	5.040135	-0.861908
H	1.264162	4.969950	-1.685045
H	0.329990	6.103375	-0.685047
H	1.011368	4.628664	0.040619
C	-1.725676	5.213337	0.953460
H	-0.744780	5.370484	1.415692
H	-2.045827	6.150379	0.475309
H	-2.440753	4.980452	1.753449
C	-3.115441	3.739311	-0.492465
H	-3.677266	3.378381	0.378051
H	-3.635034	4.613243	-0.908482
H	-3.122789	2.935278	-1.239911
H	-1.648082	0.869492	-1.030088
C	3.484152	0.417233	0.341838
O	3.303036	-0.198168	1.388811
C	4.785446	0.268286	-0.439560
H	4.549044	-0.165495	-1.426568
H	5.214005	1.261143	-0.653741
C	1.271210	1.448671	0.484444
H	1.326201	0.941744	1.452059
H	1.200197	2.518983	0.726419
N	2.537327	1.234717	-0.209429
C	2.808764	2.060566	-1.369364
H	3.282055	1.489419	-2.178892
H	1.858203	2.452234	-1.743641
H	3.460437	2.915939	-1.123740
C	5.807050	-0.593562	0.284901
H	5.321145	-1.525450	0.612750
H	6.602689	-0.877250	-0.423378
C	6.431740	0.095161	1.488725
H	6.915666	1.034151	1.164451
H	5.633906	0.379385	2.191023
C	7.454566	-0.774388	2.203512
H	6.966499	-1.706730	2.530943
H	8.239537	-1.078743	1.490867
C	8.083119	-0.074685	3.396870
H	8.814912	-0.710848	3.910552
H	8.600060	0.844687	3.087309
H	7.317098	0.213189	4.130103
H	1.210519	0.683970	-5.461506
H	1.860544	-3.630657	2.554125

Structure **L1_IrB3_PC-isoH**

C	1.508490	-1.821552	-0.001315
C	0.092941	-1.401205	1.783245
C	0.674373	-2.406238	2.543468
C	1.715390	-3.149000	1.994241
C	2.137616	-2.845876	0.708220
C	1.861552	-1.511309	-1.398405
C	1.142575	-0.601658	-3.395978
C	2.307620	-0.976634	-4.051852
C	3.295031	-1.628197	-3.321822
C	3.066086	-1.907582	-1.982647
H	-0.746370	-0.807785	2.150500
H	2.183731	-3.958502	2.551541

H	2.929876	-3.427635	0.240553
H	0.336738	-0.084940	-3.914434
H	4.232500	-1.921038	-3.790439
H	3.825888	-2.416678	-1.392809
N	0.491539	-1.108746	0.534270
N	0.930088	-0.839308	-2.100020
Ir	-0.755765	0.075011	-0.847865
O	-2.303887	0.581556	1.718085
O	-1.647477	-2.760346	-0.635516
O	0.812081	1.950470	-0.802068
B	-2.062163	-1.429766	-0.825443
B	-2.153938	0.890836	0.343089
B	1.539540	2.351057	0.379892
O	2.182187	3.582373	0.098346
O	-3.086499	1.872403	0.009552
C	-3.701350	2.367900	1.197542
C	-3.512020	1.187706	2.193267
C	0.728532	3.119306	-1.639909
C	2.076484	3.810872	-1.301106
C	-2.802707	-3.533756	-0.302024
C	-3.939437	-2.733414	-0.993308
O	-3.457511	-1.389323	-0.905006
C	-3.321187	1.595075	3.641414
H	-3.192365	0.699710	4.264948
H	-2.433639	2.227604	3.768325
H	-4.197049	2.143279	4.017727
C	-4.612900	0.141146	2.081803
H	-4.321345	-0.741834	2.668548
H	-5.573908	0.505679	2.469765
H	-4.737190	-0.173451	1.036763
C	-2.949830	3.628471	1.611495
H	-1.898488	3.410341	1.844221
H	-2.962546	4.335079	0.771614
H	-3.408634	4.117528	2.482065
C	-5.146478	2.719890	0.898025
H	-5.180614	3.559762	0.192355
H	-5.677386	1.878370	0.440718
H	-5.678349	3.021885	1.812481
C	-2.622300	-4.953464	-0.801212
H	-2.377870	-4.976632	-1.868896
H	-1.799725	-5.436450	-0.257224
H	-3.533011	-5.547659	-0.636027
C	-2.917253	-3.527486	1.219006
H	-3.744164	-4.155250	1.579008
H	-1.979655	-3.915364	1.640749
H	-3.058396	-2.502996	1.593791
C	-4.081910	-3.068153	-2.473500
H	-4.733203	-2.318819	-2.940184
H	-3.109132	-3.030476	-2.980469
H	-4.525186	-4.060962	-2.632314
C	-5.292569	-2.831012	-0.316199
H	-6.020539	-2.211134	-0.855894
H	-5.658803	-3.868227	-0.322311
H	-5.256947	-2.480809	0.721657
C	3.259772	3.146990	-2.002817
H	4.184810	3.487081	-1.519447
H	3.213383	2.052304	-1.917111
H	3.309102	3.414349	-3.067676
C	2.100823	5.306280	-1.552476
H	1.353255	5.824113	-0.941516
H	3.088134	5.708651	-1.289674
H	1.912193	5.535484	-2.611031
C	-0.482422	3.931937	-1.198591

H	-0.350156	4.311725	-0.175431
H	-0.659239	4.788743	-1.863628
H	-1.371436	3.286559	-1.210828
C	0.574354	2.723004	-3.092547
H	-0.395805	2.229967	-3.240550
H	0.608244	3.616708	-3.732498
H	1.367809	2.037729	-3.413870
H	-1.692597	0.734703	-1.978878
C	2.639089	0.851597	1.868212
O	2.682955	1.206162	0.651956
C	3.651467	-0.104779	2.416590
H	4.138921	0.356831	3.290409
H	3.112891	-0.985767	2.804197
C	0.814210	2.274700	1.837153
H	-0.204453	1.857645	1.817582
H	0.792257	3.253258	2.340297
N	1.667607	1.366599	2.608833
C	1.375806	1.072389	3.989909
H	2.050909	0.310492	4.391627
H	1.465860	1.983525	4.596525
H	0.341683	0.707293	4.069882
C	4.690271	-0.545116	1.399010
H	4.175646	-0.929839	0.505304
H	5.268300	0.324838	1.053586
C	5.617513	-1.610233	1.959999
H	6.168851	-1.210483	2.828859
H	5.017837	-2.452840	2.350242
C	6.605492	-2.135855	0.929914
H	6.044434	-2.554930	0.076979
H	7.186138	-1.294127	0.519999
C	7.542517	-3.187402	1.498907
H	6.980882	-4.047159	1.890433
H	8.243815	-3.563785	0.744426
H	8.134964	-2.778763	2.329167
H	2.435196	-0.753615	-5.108199
H	0.297413	-2.609967	3.543770

Structure **L1_IrB3_IC_TS-isoS**

C	2.479904	1.191924	0.503066
C	1.916631	2.273017	-1.458666
C	3.025822	3.099567	-1.389589
C	3.868131	2.980119	-0.291268
C	3.593593	2.018148	0.666658
C	2.178639	0.063683	1.400264
C	0.762455	-1.707194	1.887384
C	1.567652	-2.134814	2.931891
C	2.726688	-1.421414	3.213184
C	3.033362	-0.314860	2.438518
H	1.206449	2.343033	-2.280620
H	4.738334	3.625123	-0.183277
H	4.250732	1.907054	1.525965
H	-0.144618	-2.242057	1.607087
H	3.388579	-1.724582	4.022040
H	3.946653	0.243481	2.628057
N	1.639099	1.359019	-0.529323
N	1.051655	-0.635297	1.139488
Ir	-0.175573	-0.002349	-0.579452
O	-0.564792	2.985979	-0.344534
O	-1.420370	0.618217	2.192182
O	-2.285628	-1.722304	-1.992462
B	-1.708707	0.265742	0.877589
B	-1.289589	1.807985	-0.493214
B	-1.491045	-1.545363	-0.879157
O	-1.575883	-2.664379	-0.041536
O	-2.587270	2.095533	-0.885416
C	-2.618399	3.459025	-1.328661
C	-1.448131	4.098451	-0.527159
C	-3.137739	-2.854204	-1.782513
C	-2.363303	-3.672065	-0.700243
C	-2.512028	0.185603	3.021963
C	-3.695557	0.130435	2.014923
O	-3.025699	-0.167172	0.784316
C	-0.701579	5.200847	-1.254225
H	-1.365952	6.050028	-1.470097
H	0.121256	5.565190	-0.625152
H	-0.271696	4.848186	-2.198835
C	-1.844535	4.581629	0.861081
H	-2.499490	5.462356	0.823085
H	-2.347368	3.787235	1.428170
H	-0.934460	4.849852	1.412006
C	-2.368411	3.432263	-2.831829
H	-1.383010	3.004621	-3.062861
H	-3.123093	2.788879	-3.300219
H	-2.434020	4.432206	-3.281373
C	-3.985857	4.047532	-1.046383
H	-4.021742	5.106237	-1.341649
H	-4.745439	3.506816	-1.625292
H	-4.254328	3.972665	0.013219
C	-2.152017	-1.188233	3.572549
H	-2.894908	-1.542324	4.299961
H	-2.074825	-1.929981	2.764079
H	-1.179712	-1.123127	4.078676
C	-2.681342	1.172132	4.159191
H	-3.566737	0.926621	4.763190
H	-1.802237	1.136503	4.815392
H	-2.782675	2.198951	3.789920
C	-4.723711	-0.946200	2.305704
H	-5.526869	-0.906930	1.558193
H	-4.282510	-1.949321	2.277913
H	-5.178087	-0.792674	3.295337
C	-4.404623	1.464536	1.834871
H	-5.058678	1.397147	0.956299
H	-5.011583	1.727048	2.711905
H	-3.685932	2.269106	1.642034
C	-3.239680	-4.351389	0.333562
H	-3.903278	-5.091526	-0.136438
H	-2.611146	-4.876932	1.065014
H	-3.857279	-3.624951	0.874713
C	-1.398137	-4.687778	-1.296111
H	-0.765967	-5.095623	-0.495663
H	-1.928181	-5.524619	-1.769962
H	-0.741456	-4.223687	-2.044007
C	-3.327421	-3.574605	-3.104281
H	-2.371411	-3.842467	-3.567294
H	-3.925073	-4.488320	-2.971621
H	-3.863212	-2.918159	-3.801157
C	-4.479506	-2.321429	-1.299056
H	-5.210157	-3.127097	-1.143330
H	-4.357315	-1.746317	-0.374752
H	-4.874663	-1.637696	-2.060655
H	-0.886953	0.361990	-1.975536
C	3.479941	-1.478207	-1.278316
O	3.775759	-0.397471	-1.786969
C	4.545098	-2.352223	-0.620333
H	4.253058	-2.555970	0.425352
H	4.563793	-3.333970	-1.119075

C	1.115618	-1.205612	-1.883543
H	1.571099	-0.487613	-2.574348
H	0.535511	-1.915299	-2.491925
N	2.205221	-1.954426	-1.258282
C	1.852083	-3.268549	-0.772107
H	1.921174	-4.032453	-1.566384
H	2.480687	-3.584645	0.067142
H	0.812653	-3.242263	-0.423796
C	5.926773	-1.717681	-0.642083
H	6.679327	-2.487830	-0.407228
H	6.142237	-1.355583	-1.657671
C	6.065686	-0.563673	0.337683
H	5.332037	0.211698	0.068423
H	5.800402	-0.912672	1.354179
C	7.456198	0.048869	0.371724
H	8.190085	-0.713515	0.680738
H	7.744238	0.350235	-0.648012
C	7.528069	1.248597	1.302064
H	8.538242	1.672253	1.363207
H	6.853850	2.047272	0.958484
H	7.219904	0.975583	2.322888
H	1.284085	-3.013797	3.505750
H	3.214593	3.825916	-2.175810

Structure **L1_IrB3_IC-isoS**

C	1.850331	1.221181	1.670875
C	2.489345	1.316413	-0.564550
C	3.671739	1.967847	-0.250480
C	3.944635	2.239214	1.085769
C	3.026637	1.865016	2.053047
C	0.803569	0.814846	2.625515
C	-1.251162	-0.228544	2.910556
C	-1.252513	0.045018	4.270657
C	-0.174681	0.731084	4.816834
C	0.862237	1.122575	3.984951
H	2.208913	1.081725	-1.589388
H	4.868987	2.736011	1.373557
H	3.234361	2.058959	3.101394
H	-2.066830	-0.758957	2.424595
H	-0.141480	0.963322	5.879413
H	1.709178	1.667751	4.392620
N	1.603156	0.956131	0.371609
N	-0.247594	0.143106	2.107624
O	-0.172400	2.459329	-1.564997
O	-3.246365	-0.086285	0.410472
O	-1.496518	-2.686330	0.994665
B	-2.217738	0.194610	-0.495954
B	-0.758762	1.820128	-0.492049
B	-1.355017	-1.921007	-0.154497
O	-1.922700	-2.560630	-1.243318
O	-1.325171	2.733423	0.383165
C	-0.903922	4.050223	-0.018871
C	-0.538477	3.846663	-1.520633
C	-2.328178	-3.815097	0.699867
C	-2.239641	-3.908326	-0.857237
C	-4.460213	0.507771	-0.082507
C	-4.186798	0.604084	-1.611942
O	-2.756496	0.725107	-1.664720
C	-1.721301	4.019470	-2.459852
H	-1.428050	3.675756	-3.459407
H	-2.564004	3.397909	-2.136637
H	-2.045380	5.066578	-2.530493
C	0.632229	4.678554	-2.007339
H	0.817719	4.464995	-3.067657
H	0.417724	5.752802	-1.911034
H	1.551310	4.454305	-1.453285
C	-2.029561	5.031073	0.240151
H	-1.755009	6.039996	-0.100154
H	-2.954639	4.734611	-0.267882
H	-2.236686	5.079887	1.317112
C	0.297531	4.404068	0.848107
H	0.635397	5.435863	0.682281
H	0.014441	4.295984	1.903752
H	1.140709	3.727239	0.655096
C	-5.645760	-0.360811	0.289419
H	-5.712114	-0.450299	1.381857
H	-6.580017	0.093456	-0.071047
H	-5.574932	-1.370102	-0.131111
C	-4.593421	1.861901	0.602135
H	-3.735752	2.506138	0.371945
H	-5.524058	2.374291	0.323048
H	-4.600918	1.703299	1.688399
C	-4.544058	-0.660057	-2.380716
H	-4.162375	-0.562856	-3.404682
H	-4.058873	-1.536971	-1.935904
H	-5.629499	-0.821848	-2.429581
C	-4.824549	1.801660	-2.288604
H	-4.548566	2.748594	-1.810809
H	-4.505449	1.844078	-3.338034
H	-5.920703	1.715606	-2.268519
C	-1.095427	-4.784312	-1.352777
H	-0.140291	-4.501552	-0.890430
H	-1.276062	-5.849371	-1.155299
H	-0.993545	-4.644653	-2.436543
C	-3.525209	-4.336083	-1.538798
H	-4.364958	-3.676563	-1.290885
H	-3.389510	-4.314389	-2.627834
H	-3.795007	-5.361826	-1.248387
C	-3.719297	-3.477900	1.214580
H	-3.652185	-3.273386	2.291625
H	-4.099292	-2.570355	0.731400
H	-4.432796	-4.299261	1.064404
C	-1.787852	-5.027082	1.434129
H	-1.878390	-4.872319	2.516816
H	-2.356412	-5.931157	1.171787
H	-0.729334	-5.198621	1.208631
H	-0.046381	-0.388419	-1.653666
C	3.624104	-1.478074	-0.061722
O	3.764707	-1.188663	1.125079
C	4.822058	-1.487100	-1.002508
H	4.923788	-2.482455	-1.466981
H	4.638765	-0.796582	-1.844308
C	1.225129	-1.872212	0.232957
H	0.802635	-2.874822	0.080985
H	1.555305	-1.828999	1.276812
N	2.414517	-1.788308	-0.607925
C	2.246084	-2.242262	-1.970185
H	1.204718	-2.057881	-2.263133
H	2.890324	-1.697804	-2.670648
H	2.451423	-3.321133	-2.075830
C	6.112350	-1.104671	-0.301523
H	5.996220	-0.108448	0.154651
H	6.284159	-1.787702	0.543625
C	7.309494	-1.113053	-1.237837
H	7.421801	-2.114159	-1.690420
H	7.126880	-0.425899	-2.083733

C	8.609522	-0.725552	-0.549155
H	8.495573	0.273947	-0.098279
H	8.790757	-1.413467	0.292427
C	9.800055	-0.735431	-1.493121
H	10.731296	-0.454273	-0.985620
H	9.947425	-1.732807	-1.930671
H	9.649283	-0.033773	-2.325733
H	-2.089343	-0.277513	4.884900
H	4.365843	2.244319	-1.040777
Ir	-0.200126	-0.205093	-0.071596

Structure **L1_IrB3_IC-isoS_TS-RE**

C	2.254929	1.702306	-0.900006
C	1.049536	1.732196	-2.879668
C	1.998084	2.525970	-3.508357
C	3.116369	2.915497	-2.782195
C	3.246327	2.503143	-1.464798
C	2.309897	1.215629	0.494350
C	1.286289	-0.015502	2.165822
C	2.325896	0.229458	3.054486
C	3.393532	1.009483	2.631670
C	3.381832	1.510215	1.338631
H	0.143986	1.405049	-3.386634
H	3.885290	3.536549	-3.237316
H	4.119718	2.799155	-0.890558
H	0.407020	-0.587886	2.452677
H	4.226791	1.228783	3.296762
H	4.210578	2.119359	0.988579
N	1.179273	1.331539	-1.613275
N	1.274175	0.460404	0.915532
Ir	-0.402829	0.072840	-0.591754
O	-1.635677	2.749865	-1.341041
O	-3.263291	-0.180344	0.574137
O	-1.552991	-2.829868	-0.008883
B	-1.887673	-0.207189	0.775177
B	-1.219470	1.957868	-0.283263
B	-1.166552	-1.863340	-0.961997
O	-1.770000	-2.174869	-2.191233
O	-1.191792	2.695230	0.895158
C	-1.279867	4.082996	0.532186
C	-2.034842	4.027665	-0.826777
C	-2.222077	-3.899307	-0.695443
C	-2.740744	-3.197838	-1.984450
C	-3.915384	-0.099448	1.848750
C	-2.829534	-0.630835	2.834853
O	-1.613491	-0.341974	2.135223
C	-3.548171	4.000651	-0.675867
H	-3.946546	4.967969	-0.340472
H	-3.993926	3.760390	-1.648926
H	-3.857309	3.222275	0.032839
C	-1.633535	5.100555	-1.820804
H	-2.224614	4.991801	-2.739110
H	-1.820853	6.105256	-1.414743
H	-0.574454	5.021775	-2.093227
C	-1.987058	4.846975	1.632446
H	-1.386348	4.815131	2.550655
H	-2.122440	5.901426	1.351222
H	-2.970353	4.418922	1.857518
C	0.153920	4.583450	0.390964
H	0.199636	5.664053	0.199444
H	0.692987	4.372043	1.323766
H	0.675296	4.063099	-0.426291
C	-4.258731	1.366200	2.080326
H	-4.799612	1.520742	3.023710
H	-3.347964	1.980712	2.081517
H	-4.902836	1.706311	1.258567
C	-5.189806	-0.919354	1.802862
H	-5.875769	-0.490626	1.060722
H	-4.995040	-1.959905	1.517541
H	-5.695971	-0.914716	2.779214
C	-2.796954	0.071296	4.179480
H	-2.002854	-0.362325	4.801687
H	-2.594099	1.142409	4.072572
H	-3.749547	-0.058667	4.713437
C	-2.877517	-2.137101	3.044340
H	-1.992358	-2.439518	3.620026
H	-3.771365	-2.449622	3.601339
H	-2.839579	-2.661280	2.083666
C	-2.787495	-4.089841	-3.211097
H	-3.154978	-3.512227	-4.068685
H	-1.796297	-4.479011	-3.470414
H	-3.470437	-4.938017	-3.056583
C	-4.089321	-2.514189	-1.792424
H	-4.289449	-1.895063	-2.676106
H	-4.910444	-3.236954	-1.685564
H	-4.070456	-1.846858	-0.920964
C	-3.312916	-4.470872	0.189763
H	-2.871218	-4.934909	1.082077
H	-4.017690	-3.699081	0.519018
H	-3.878326	-5.244919	-0.349027
C	-1.195448	-4.994050	-0.968401
H	-1.661335	-5.885280	-1.409744
H	-0.398304	-4.660275	-1.645048
H	-0.730947	-5.287132	-0.017272
H	-1.490512	0.136920	-1.744261
C	3.080588	-1.624065	-0.625983
O	3.327204	-0.891877	-1.583985
C	4.166484	-1.958643	0.384699
H	3.845902	-1.627216	1.388064
H	4.285413	-3.051983	0.465113
C	0.762408	-1.831637	-1.331974
H	1.199356	-1.275806	-2.167434
H	0.446650	-2.777729	-1.793402
N	1.840108	-2.137090	-0.394673
C	1.546394	-3.051886	0.689167
H	0.472241	-3.009750	0.902055
H	1.804714	-4.092950	0.431218
H	2.088235	-2.781843	1.603278
C	5.491863	-1.304816	0.040034
H	5.835309	-1.661176	-0.942800
H	5.334020	-0.221217	-0.087626
C	6.555860	-1.556562	1.095029
H	6.202584	-1.183090	2.073937
H	6.700183	-2.643159	1.229575
C	7.892311	-0.909866	0.763707
H	8.242481	-1.284863	-0.211448
H	7.745666	0.174734	0.630723
C	8.947901	-1.164122	1.826486
H	9.906396	-0.692368	1.576821
H	8.628102	-0.772661	2.802650
H	9.129535	-2.240788	1.951358
H	2.284594	-0.182377	4.059816
H	1.857173	2.827251	-4.542966

Structure **L1_IrB3_PC-isoS**

C	-1.867256	-1.546089	-1.372053

C	-1.179555	-0.609785	-3.368835
C	-2.327774	-1.028782	-4.027265
C	-3.288261	-1.723205	-3.300851
C	-3.052160	-1.992414	-1.961084
C	-1.511676	-1.833659	0.029252
C	-0.106146	-1.370806	1.811041
C	-0.676996	-2.371768	2.584469
C	-1.708362	-3.134158	2.044011
C	-2.130261	-2.854844	0.752741
H	-0.392539	-0.063379	-3.885613
H	-4.210646	-2.056216	-3.772532
H	-3.791629	-2.534848	-1.375506
H	0.727701	-0.765130	2.170390
H	-2.169246	-3.940431	2.612047
H	-2.915013	-3.452638	0.293090
N	-0.959541	-0.841471	-2.072826
N	-0.505800	-1.099692	0.557501
Ir	0.737692	0.073964	-0.840104
O	1.589285	-2.774912	-0.637228
O	2.304665	0.580716	1.715187
O	-0.792852	1.977783	-0.809250
B	2.155978	0.876699	0.337049
B	2.022815	-1.449746	-0.824192
B	-1.514949	2.393420	0.370723
O	-2.138499	3.633293	0.084242
O	3.418194	-1.429567	-0.907841
C	3.880454	-2.780425	-0.998927
C	2.733983	-3.564577	-0.305556
C	-0.687820	3.141837	-1.650567
C	-2.022663	3.860962	-1.314443
C	3.527002	1.166776	2.178884
C	3.736204	2.331790	1.169688
O	3.106853	1.835694	-0.010500
C	2.532794	-4.981772	-0.803757
H	2.285755	-5.001860	-1.870923
H	3.435577	-5.588433	-0.640426
H	1.704874	-5.453222	-0.257743
C	2.850860	-3.558463	1.215280
H	1.907358	-3.930361	1.638435
H	3.667920	-4.199083	1.575111
H	3.008925	-2.535717	1.588427
C	4.014385	-3.115577	-2.479785
H	4.443081	-4.114422	-2.640725
H	3.040950	-3.063516	-2.984209
H	4.675080	-2.375102	-2.947397
C	5.233511	-2.898445	-0.325002
H	5.968573	-2.286408	-0.864023
H	5.204579	-2.551803	0.714261
H	5.585777	-3.940446	-0.335557
C	3.351244	1.593097	3.623435
H	4.239817	2.127285	3.989982
H	2.477335	2.244802	3.747734
H	3.207320	0.707102	4.256985
C	4.605151	0.096263	2.073439
H	4.718066	-0.230226	1.030824
H	5.575322	0.444012	2.453907
H	4.297359	-0.774847	2.669482
C	3.012053	3.611674	1.573050
H	1.957099	3.417797	1.811103
H	3.483704	4.099716	2.437294
H	3.036822	4.309697	0.726311
C	5.186929	2.651177	0.860853
H	5.728231	2.953249	1.769731

H	5.699149	1.793814	0.411719
H	5.235162	3.481779	0.145115
C	-2.014520	5.356956	-1.564293
H	-1.816322	5.583147	-2.621765
H	-2.994436	5.779554	-1.305587
H	-1.259247	5.858728	-0.949482
C	-3.217985	3.224459	-2.020225
H	-3.257297	3.491576	-3.085542
H	-3.196315	2.129411	-1.933162
H	-4.136814	3.585734	-1.540506
C	-0.541765	2.736845	-3.101799
H	-1.347717	2.064426	-3.419112
H	-0.560336	3.627989	-3.745944
H	0.419251	2.225805	-3.248259
C	0.538198	3.933086	-1.212330
H	0.413236	4.318546	-0.190339
H	1.414881	3.271072	-1.222534
H	0.730703	4.784474	-1.879972
H	1.674894	0.718544	-1.978927
C	-2.623013	0.901126	1.859606
O	-2.669077	1.261083	0.644960
C	-3.636030	-0.054954	2.407008
H	-3.097499	-0.934337	2.798422
H	-4.126161	0.408061	3.278651
C	-0.790481	2.314193	1.828511
H	-0.764445	3.292767	2.331403
H	0.226477	1.892869	1.809201
N	-1.647529	1.409361	2.599764
C	-1.359658	1.117119	3.982066
H	-0.325752	0.752482	4.065749
H	-1.451529	2.029273	4.586980
H	-2.035906	0.355959	4.383232
C	-4.672227	-0.499868	1.388717
H	-5.253708	0.367425	1.042422
H	-4.156234	-0.883818	0.495435
C	-5.594943	-1.567985	1.951602
H	-4.990921	-2.409165	2.338233
H	-6.143831	-1.170811	2.823238
C	-6.585933	-2.095104	0.925242
H	-7.172817	-1.254904	0.521140
H	-6.027749	-2.508559	0.067770
C	-7.514813	-3.152699	1.496415
H	-8.219148	-3.529686	0.745060
H	-6.947008	-4.011279	1.881640
H	-8.103899	-2.749535	2.331742
H	-0.299531	-2.556676	3.588239
H	-2.462071	-0.809363	-5.083512

Structure **1a-B**

C	1.011245	-1.164861	0.198160
O	0.403372	-0.193821	0.652208
C	2.501425	-1.109557	-0.056811
H	3.006108	-1.859336	0.575211
H	2.705503	-1.413528	-1.096330
C	0.931228	-3.566831	-0.462285
H	1.985465	-3.450136	-0.725230
H	0.857785	-4.292159	0.363482
N	0.334670	-2.305487	-0.095770
C	-1.084806	-2.313923	0.243541
H	-1.594443	-2.974255	-0.474282
H	-1.229266	-2.749996	1.245823
C	3.087498	0.266933	0.204989
H	2.571808	1.007321	-0.425198

H	2.870383	0.564012	1.241539
C	4.584754	0.314089	-0.052224
H	5.095687	-0.432142	0.581837
H	4.794175	0.012380	-1.093851
C	5.187763	1.687169	0.203383
H	4.675680	2.429572	-0.429733
H	4.978575	1.985328	1.243343
C	6.684026	1.727054	-0.057185
H	7.215897	1.011960	0.586048
H	6.910286	1.460002	-1.099062
H	7.107477	2.721446	0.130426
H	0.409021	-3.990961	-1.330986
C	-2.513266	1.041603	-0.744964
C	-3.294362	0.706024	0.564778
C	-1.428753	2.092394	-0.545049
H	-0.793540	2.109538	-1.440144
H	-1.859649	3.093539	-0.408785
H	-0.787008	1.852978	0.309689
C	-3.385833	1.427753	-1.923054
H	-3.968135	2.332585	-1.697338
H	-2.753373	1.641950	-2.793966
H	-4.078082	0.625210	-2.199976
C	-3.425145	1.857898	1.540426
H	-3.972552	2.695493	1.084783
H	-3.983299	1.529954	2.426648
H	-2.445446	2.216507	1.872808
C	-4.662942	0.091580	0.302241
H	-5.055641	-0.309466	1.244911
H	-5.378037	0.830299	-0.083403
H	-4.595055	-0.736488	-0.415396
O	-1.864523	-0.207314	-1.041752
O	-2.488108	-0.324027	1.162327
B	-1.719192	-0.863172	0.159916

Structure **L1_IrB2H**

C	3.106090	1.507151	-1.156983
C	4.456755	1.827599	-1.174776
C	5.361902	0.918905	-0.639296
C	4.886897	-0.266279	-0.095466
C	3.674085	-2.763855	1.026853
C	3.040682	-3.852112	1.609451
C	1.652977	-3.887742	1.637460
C	0.946705	-2.839652	1.063827
H	-0.143889	-2.801356	1.051299
C	2.911550	-1.733757	0.479866
C	3.515715	-0.521465	-0.112466
C	-3.176915	-2.257810	0.241367
C	-3.695295	-1.165022	-0.734595
C	-2.955636	-3.605268	-0.437447
H	-2.360263	-3.491468	-1.353076
H	-3.901504	-4.101892	-0.693251
H	-2.402430	-4.261788	0.248493
C	-4.007914	-2.444427	1.495236
H	-3.567471	-3.232169	2.121069
H	-5.035022	-2.747345	1.244380
H	-4.047608	-1.525397	2.089580
C	-4.610943	-1.675467	-1.830742
H	-4.923878	-0.838249	-2.467612
H	-5.515544	-2.135838	-1.406512
H	-4.108624	-2.410378	-2.469492
C	-4.357464	0.004191	-0.014126
H	-3.705924	0.399237	0.775818
H	-5.330294	-0.274655	0.414408
H	-4.521049	0.808995	-0.743404
B	-1.434135	-0.916446	-0.440798
N	2.647966	0.358537	-0.650171
N	1.556776	-1.788675	0.493269
O	-1.886966	-1.750525	0.600633
O	-2.485769	-0.679429	-1.321578
Ir	0.471944	-0.254387	-0.599740
B	-0.286929	1.462099	0.048033
O	0.433896	2.661988	0.012750
O	-1.532296	1.669761	0.630052
C	-0.290166	3.623189	0.792819
C	-1.745301	3.084879	0.698917
C	0.266108	3.577137	2.210286
C	-0.081553	5.002622	0.200436
C	-2.624779	3.398093	1.892237
C	-2.446706	3.495805	-0.589884
H	0.100312	2.592928	2.666945
H	-0.184702	4.344640	2.854073
H	1.348467	3.754006	2.170343
H	0.967682	5.303960	0.318097
H	-0.706013	5.747607	0.714529
H	-0.321180	5.022999	-0.868298
H	-3.627729	2.980753	1.731731
H	-2.728688	4.484340	2.029203
H	-2.223654	2.963778	2.814543
H	-1.816566	3.292740	-1.465758
H	-2.722609	4.559281	-0.589694
H	-3.361582	2.899266	-0.694966
H	2.343220	2.183959	-1.536823
H	-0.163674	0.748701	-1.700475
H	4.786246	2.772180	-1.600058
H	6.428876	1.132553	-0.635350
H	5.582568	-0.975755	0.345319
H	4.759543	-2.721844	0.996558
H	3.628823	-4.661419	2.037707
H	1.114890	-4.716564	2.090074

Structure **BH**

C	-0.777257	-0.185148	-0.055887
C	0.777141	-0.185593	0.055829
C	-1.473768	-0.432016	1.274469
H	-2.540008	-0.200595	1.163208
H	-1.378564	-1.477459	1.595558
H	-1.068946	0.214214	2.063990
C	-1.343257	-1.106094	-1.116220
H	-1.074298	-2.150869	-0.905698
H	-2.437842	-1.032893	-1.122988
H	-0.982618	-0.846217	-2.117044
C	1.342780	-1.108181	1.114920
H	1.073153	-2.152551	0.903216
H	2.437409	-1.035642	1.121616
H	0.982422	-0.849222	2.116072
C	1.473408	-0.430983	-1.274901
H	2.539705	-0.199955	-1.163427
H	1.377943	-1.476000	-1.597333
H	1.068636	0.216388	-2.063526
O	-1.061209	1.183543	-0.422486
O	1.061673	1.182520	0.424132
B	0.000720	1.927369	0.000675
H	0.001412	3.113546	0.000566

Structure **L2_IrB5**

Ir	-0.206202	-0.033476	-0.389091

B	0.141476	-2.147298	-0.344560
C	-1.089996	0.269381	-3.458482
C	-0.949558	0.425950	-4.830140
C	1.417051	0.259993	-4.524846
C	1.198707	0.105854	-3.155304
C	2.298656	-0.045389	-2.185684
C	3.634150	-0.162845	-2.574098
C	4.257086	-0.299816	-0.279426
C	2.913887	-0.184021	0.049147
B	0.358543	2.110241	-0.338662
N	-0.045824	0.109638	-2.644135
N	1.960492	-0.060372	-0.875468
O	0.695267	2.856813	0.767262
O	0.864460	2.681918	-1.493689
O	-1.004827	-0.781959	2.519610
O	1.040572	0.197611	2.318631
O	-2.413453	2.157083	-0.869794
O	-2.021874	1.809941	1.348950
C	0.330357	0.419325	-5.370891
C	4.628221	-0.288510	-1.616437
C	1.454068	3.948880	-1.154365
C	1.704821	3.802369	0.378083
C	-2.952039	2.891502	1.301264
C	-3.469562	2.848605	-0.176516
C	-0.374229	-0.891486	3.797342
C	0.731898	0.199358	3.719233
C	1.493599	-3.943621	-0.757296
B	-1.647596	1.459547	0.067857
H	-2.065323	0.273580	-2.976669
B	-0.105242	-0.228007	1.633546
C	0.338319	-4.365993	0.187606
O	-0.178080	-3.093529	0.616781
O	1.001664	-2.704532	-1.288690
C	-0.777553	-5.093503	-0.547583
H	-1.627797	-5.222166	0.135702
H	-1.120254	-4.511812	-1.413850
H	-0.462030	-6.090173	-0.884390
C	0.757225	-5.162940	1.406087
H	-0.125627	-5.385909	2.019793
H	1.215898	-6.117577	1.109747
H	1.467342	-4.612522	2.033236
C	2.785327	-3.630091	-0.011393
H	3.274943	-4.537425	0.367361
H	3.478631	-3.128965	-0.701738
H	2.607011	-2.951829	0.836193
C	1.777157	-4.897290	-1.899588
H	2.606826	-4.510822	-2.506061
H	2.067184	-5.888496	-1.521988
H	0.906577	-5.010513	-2.554391
C	-1.414715	-0.663510	4.876072
H	-2.146312	-1.481958	4.860618
H	-0.952582	-0.637372	5.874002
H	-1.957577	0.273887	4.710180
C	0.184662	-2.305062	3.900376
H	-0.632640	-3.014397	3.714697
H	0.947618	-2.482321	3.129712
H	0.615649	-2.510207	4.890255
C	0.217374	1.594443	4.053659
H	0.029061	1.714707	5.129614
H	0.971407	2.330614	3.742902
H	-0.701535	1.811172	3.492486
C	1.998345	-0.098678	4.495721
H	2.718383	0.719039	4.352928
H	1.792370	-0.185141	5.572370
H	2.473816	-1.026255	4.154484
C	-4.011957	2.643513	2.358890
H	-3.545930	2.690759	3.352401
H	-4.805145	3.404224	2.318263
H	-4.457993	1.648448	2.247819
C	-4.735017	2.023422	-0.339350
H	-5.602397	2.505319	0.131554
H	-4.949478	1.909657	-1.411104
H	-4.593151	1.029384	0.096422
C	-3.674097	4.205958	-0.822309
H	-4.016461	4.072403	-1.856565
H	-4.441377	4.779850	-0.282461
H	-2.752641	4.797702	-0.848619
C	-2.186641	4.164767	1.635597
H	-1.659087	4.023282	2.588497
H	-1.423781	4.375383	0.878226
H	-2.854255	5.032634	1.725456
C	1.530972	5.070953	1.189100
H	1.676414	4.843389	2.253563
H	2.275066	5.825944	0.896622
H	0.531861	5.504195	1.073602
C	0.434032	5.021400	-1.504641
H	0.822045	6.033182	-1.325548
H	0.176198	4.929878	-2.567005
H	-0.491717	4.890194	-0.929417
C	2.704674	4.139100	-1.991053
H	2.429442	4.236357	-3.049358
H	3.242482	5.051337	-1.694972
H	3.386638	3.285348	-1.898374
C	3.048212	3.167317	0.713714
H	3.879920	3.876618	0.604693
H	3.019286	2.810238	1.752281
H	3.253938	2.298506	0.076131
B	-2.069284	-0.961472	-0.358199
O	-2.393038	-1.889839	-1.358835
O	-3.144360	-0.814854	0.503359
C	-3.780248	-2.240076	-1.211715
C	-4.035497	-1.917732	0.285033
C	-4.562260	-1.347193	-2.166843
C	-3.989589	-3.690839	-1.593207
C	-5.458292	-1.511497	0.616848
C	-3.589797	-3.020427	1.239324
H	-4.439432	-0.287056	-1.906941
H	-5.634415	-1.585827	-2.175890
H	-4.171495	-1.497536	-3.182498
H	-3.730637	-3.840347	-2.650007
H	-5.043829	-3.973772	-1.459039
H	-3.371914	-4.366184	-0.993765
H	-5.510614	-1.195996	1.667392
H	-6.138790	-2.365247	0.485479
H	-5.823509	-0.686277	-0.003656
H	-2.543970	-3.300844	1.056898
H	-4.232479	-3.909655	1.174806
H	-3.639045	-2.624673	2.262146
H	2.574450	-0.166681	1.088410
H	-1.829797	0.554247	-5.454914
H	0.483877	0.542923	-6.441068
H	2.424309	0.268733	-4.932739
H	5.672983	-0.380449	-1.903535
H	3.901921	-0.164884	-3.626606
C	5.239580	-0.391710	0.843771
F	4.923377	-1.383125	1.683938

```
 F     5.252298   0.738555   1.560781
 F     6.482344  -0.607931   0.403129
```

Structure **L2_IrB3**

```
 C     3.249177  -0.132918  -0.198474
 H     2.933363   0.900839  -0.031246
 C     4.588861  -0.492508  -0.303123
 C     4.912864  -1.825698  -0.511250
 H     5.952014  -2.131088  -0.607892
 C     3.888586  -2.758061  -0.590943
 C     1.566507  -4.655376  -0.496380
 C     0.433984  -5.457764  -0.495948
 H     0.532249  -6.541385  -0.492707
 C    -0.819393  -4.858233  -0.484954
 C    -0.891780  -3.471929  -0.484108
 H    -1.842709  -2.941108  -0.445940
 C     1.416893  -3.269019  -0.501172
 C     2.565725  -2.336802  -0.465108
 C     0.974257   3.574341   0.601871
 C    -0.254617   3.831118  -0.316557
 C     0.620574   3.592838   2.083672
 H    -0.240290   2.941484   2.283655
 H     0.400312   4.608804   2.439231
 H     1.476229   3.206778   2.653476
 C     2.169769   4.468933   0.341664
 H     2.987785   4.200819   1.023519
 H     1.915378   5.524101   0.518238
 H     2.540275   4.366855  -0.684520
 C    -1.296512   4.769630   0.260310
 H    -2.116886   4.896488  -0.458897
 H    -0.867804   5.762145   0.462648
 H    -1.721578   4.370183   1.187876
 C     0.132684   4.283655  -1.718923
 H     0.894666   3.621990  -2.150950
 H     0.512250   5.314666  -1.730330
 H    -0.755801   4.235331  -2.361638
 C    -3.267060   0.631462  -2.730030
 C    -4.019585  -0.042350  -1.548089
 C    -3.291323   2.153319  -2.658160
 H    -2.966949   2.505631  -1.670809
 H    -4.287121   2.557960  -2.886804
 H    -2.580738   2.544400  -3.398287
 C    -3.707586   0.173523  -4.106582
 H    -3.121403   0.695807  -4.873466
 H    -4.769440   0.405580  -4.275517
 H    -3.557611  -0.902977  -4.245880
 C    -5.225916   0.720215  -1.037537
 H    -5.699856   0.162979  -0.217995
 H    -5.973929   0.851659  -1.832981
 H    -4.941105   1.706389  -0.655311
 C    -4.404432  -1.489962  -1.831482
 H    -3.550747  -2.050129  -2.237818
 H    -5.241712  -1.568805  -2.538212
 H    -4.704538  -1.961321  -0.885438
 B     0.157960   1.574776  -0.191571
 B    -1.763882  -0.041804  -1.140584
 N     2.266529  -1.031351  -0.281884
 N     0.196878  -2.697415  -0.504840
 O     1.318351   2.220696   0.266995
 O    -0.826504   2.525272  -0.435337
 O    -1.915020   0.211160  -2.504754
 O    -3.016441  -0.072635  -0.526563
 Ir    0.065776  -0.436081  -0.410243
 H    -1.730828  -5.450198  -0.471800
 B    -0.992337  -0.394936   1.271041
 O    -1.311038   0.705601   2.053482
 O    -1.437632  -1.570568   1.874986
 C    -1.773626   0.224789   3.321630
 C    -2.289717  -1.202369   2.970247
 C    -0.573719   0.208531   4.261125
 C    -2.829701   1.176152   3.847301
 C    -2.135150  -2.224592   4.079407
 C    -3.724180  -1.211064   2.457355
 H     0.201962  -0.478789   3.898736
 H    -0.853475  -0.082630   5.282762
 H    -0.138616   1.215580   4.297476
 H    -2.373676   2.153103   4.057074
 H    -3.269790   0.799629   4.782206
 H    -3.629896   1.328963   3.115259
 H    -2.523880  -3.195019   3.743983
 H    -2.704378  -1.923341   4.970808
 H    -1.086128  -2.362319   4.363598
 H    -3.860415  -0.472058   1.659087
 H    -4.446312  -1.022723   3.263776
 H    -3.933645  -2.202962   2.032835
 H     4.125472  -3.804830  -0.760822
 H     2.553667  -5.110197  -0.476241
 C     5.625299   0.585639  -0.219144
 F     5.472114   1.315640   0.888119
 F     5.532393   1.421381  -1.257511
 F     6.863191   0.081191  -0.212114
```

Structure **L2_IrB**

```
 C     2.405643  -0.911918  -0.020100
 C     0.174304  -1.635404  -0.023764
 C     0.577086  -2.950702  -0.016553
 C     1.934746  -3.278898  -0.013620
 C     2.838655  -2.237629  -0.017840
 C     3.322412   0.223796  -0.012995
 C     3.467826   2.534401   0.038890
 C     4.853491   2.504109   0.006936
 C     5.485593   1.264713  -0.037985
 C     4.713682   0.113493  -0.048437
 H    -0.876853  -1.359822  -0.040255
 H     2.258526  -4.317115  -0.005868
 H     3.905202  -2.446385  -0.012482
 H     2.921446   3.476691   0.074158
 H     6.570954   1.195121  -0.065926
 H     5.194092  -0.860913  -0.087895
 N     1.061742  -0.600764  -0.020621
 N     2.715981   1.429232   0.029220
 Ir    0.487654   1.241897   0.024407
 O    -2.329835  -0.079878  -0.299103
 B    -1.529050   1.031211  -0.011286
 O    -2.349709   2.129892   0.263631
 C    -3.681951   0.232507   0.061840
 C    -3.700589   1.781183  -0.073081
 C    -3.947756   2.249092  -1.501621
 H    -3.734393   3.323719  -1.561022
 H    -3.283426   1.733524  -2.207076
 H    -4.988452   2.086761  -1.813831
 C    -4.649104   2.489938   0.872955
 H    -4.597524   3.573580   0.706560
 H    -5.686850   2.169432   0.699697
 H    -4.392938   2.298562   1.920633
 C    -3.885851  -0.247036   1.493670
```

H	-3.225264	0.288048	2.188131
H	-4.924760	-0.120482	1.827027
H	-3.635837	-1.315105	1.543700
C	-4.624108	-0.506631	-0.866881
H	-5.669081	-0.227629	-0.668459
H	-4.399596	-0.301644	-1.919175
H	-4.525459	-1.588279	-0.706482
C	-0.437513	-4.052092	-0.016320
F	-0.316802	-4.808325	-1.114736
F	-0.251932	-4.865351	1.032086
F	-1.689262	-3.599460	0.033976
H	5.420934	3.431080	0.016708

Structure **L2_IrB3_RC**

C	-2.078437	1.197010	1.231506
C	-2.183740	-0.849594	0.153218
C	-3.361315	-1.182806	0.813436
C	-3.894640	-0.292710	1.737121
C	-3.238041	0.910077	1.951832
C	-1.343710	2.467600	1.377731
C	0.636136	3.597323	0.985411
C	0.111915	4.785666	1.475562
C	-1.199989	4.792767	1.933710
C	-1.938385	3.619770	1.885434
H	-1.697333	-1.534312	-0.548905
H	-4.806185	-0.537915	2.278163
H	-3.618605	1.616345	2.685672
H	1.652224	3.528643	0.593215
H	-1.651310	5.708390	2.310190
H	-2.982674	3.613675	2.188862
N	-1.562237	0.314800	0.349789
N	-0.072115	2.465748	0.940434
Ir	0.676581	0.601166	-0.131764
O	1.471087	-1.968320	1.320118
O	3.642529	0.201451	0.535040
O	2.298871	-1.305143	-1.952575
B	2.664972	0.895720	-0.172426
B	1.033058	-0.656246	1.366706
B	1.125437	-1.020364	-1.257684
O	0.199822	-2.052332	-1.483727
O	0.728598	-0.285311	2.674910
C	0.766764	-1.478534	3.474491
C	1.740013	-2.383368	2.666879
C	2.288578	-2.688368	-2.324460
C	0.762329	-3.007014	-2.394381
C	4.925973	0.647054	0.085489
C	4.616162	2.080147	-0.436495
O	3.250756	1.966125	-0.864242
C	3.207684	-2.084826	2.945859
H	3.412084	-1.008451	2.889985
H	3.523169	-2.467840	3.926146
H	3.814654	-2.567033	2.167213
C	1.476454	-3.871047	2.782892
H	2.210239	-4.424694	2.181744
H	1.573565	-4.206792	3.825566
H	0.476486	-4.134421	2.419880
C	1.234098	-1.129619	4.872250
H	1.358609	-2.037316	5.480447
H	2.185410	-0.587192	4.853062
H	0.490288	-0.490593	5.365686
C	-0.654907	-2.027376	3.519252
H	-1.321349	-1.241336	3.899309
H	-1.004251	-2.315261	2.517157
H	-0.741303	-2.900117	4.180680
C	5.900644	0.584951	1.245766
H	6.038110	-0.460977	1.550662
H	6.883442	0.987273	0.959512
H	5.533391	1.140679	2.116262
C	5.376137	-0.304354	-1.015961
H	6.381529	-0.060394	-1.385843
H	5.397962	-1.321635	-0.602361
H	4.664231	-0.304153	-1.851136
C	4.654007	3.132226	0.665580
H	4.046580	2.821922	1.527147
H	5.676491	3.332491	1.013351
H	4.239993	4.070685	0.272103
C	5.467193	2.531602	-1.606557
H	6.532683	2.549310	-1.334323
H	5.337133	1.874433	-2.472993
H	5.177530	3.547150	-1.906840
C	0.159458	-2.740044	-3.767120
H	0.471332	-3.490419	-4.506180
H	-0.935708	-2.772724	-3.687335
H	0.446229	-1.747327	-4.138751
C	0.380352	-4.397629	-1.925099
H	0.861774	-5.171247	-2.540871
H	0.663594	-4.551221	-0.877324
H	-0.707234	-4.530042	-2.003253
C	3.015958	-3.452452	-1.224156
H	4.034790	-3.051735	-1.139797
H	2.522106	-3.304637	-0.256218
H	3.086281	-4.526301	-1.446084
C	3.034628	-2.855579	-3.634703
H	2.987723	-3.897800	-3.983021
H	2.636865	-2.204808	-4.421195
H	4.091589	-2.596892	-3.488346
H	0.734153	1.972991	-2.018498
C	-2.340056	2.282079	-2.077710
O	-2.347293	3.242758	-1.315708
C	-3.565310	1.401984	-2.253017
H	-3.893271	1.428245	-3.305107
H	-3.280427	0.352035	-2.066208
C	0.013080	2.622334	-2.562186
H	0.499884	2.895286	-3.508110
H	-0.183822	3.521532	-1.974910
N	-1.238520	1.940993	-2.817671
C	-1.134059	0.691020	-3.545383
H	-2.042996	0.480058	-4.118497
H	-0.303585	0.771546	-4.257467
H	-0.922101	-0.158669	-2.873867
C	-4.705902	1.792818	-1.328885
H	-4.301281	1.982403	-0.322453
H	-5.138739	2.752612	-1.649765
C	-5.779759	0.720190	-1.249238
H	-6.233904	0.555116	-2.241646
H	-5.310105	-0.243509	-0.979725
C	-6.869098	1.036430	-0.235796
H	-6.396376	1.291154	0.728878
H	-7.412490	1.942654	-0.547971
C	-7.833668	-0.121529	-0.044398
H	-8.622070	0.110848	0.682234
H	-8.321773	-0.390287	-0.991535
H	-7.300429	-1.015186	0.311120
C	-3.992019	-2.503721	0.507310
F	-4.198604	-2.639828	-0.809171
F	-3.208997	-3.517946	0.886332

F	-5.172441	-2.647190	1.119316
H	0.721469	5.685621	1.487374

Structure **L2_IrB3_TS-OA**

C	1.713092	-1.868203	1.052492
C	2.293764	0.359123	0.815836
C	3.557773	0.136215	1.344821
C	3.889474	-1.146568	1.767322
C	2.955323	-2.156483	1.619760
C	0.682939	-2.897002	0.823086
C	-1.388712	-3.395867	-0.093564
C	-1.333590	-4.702970	0.366815
C	-0.217969	-5.108243	1.088740
C	0.796631	-4.193985	1.322734
H	1.960326	1.353039	0.517073
H	4.872888	-1.354924	2.183643
H	3.212180	-3.170753	1.910134
H	-2.258149	-3.006470	-0.623628
H	-0.140546	-6.122656	1.474581
H	1.665679	-4.487640	1.904814
N	1.397019	-0.615476	0.671112
N	-0.402463	-2.516893	0.117712
Ir	-0.579145	-0.365983	-0.427220
O	-2.799027	0.710723	1.514059
O	-2.696901	0.314069	-2.527214
O	-0.049803	2.336014	0.802780
B	-2.371516	-0.367504	-1.364199
B	-1.811767	-0.233006	1.256932
B	-0.810269	1.662832	-0.157061
O	-1.588104	2.561248	-0.863474
O	-1.792536	-1.189129	2.267576
C	-2.993979	-1.033925	3.039828
C	-3.324663	0.469814	2.826938
C	-0.166646	3.745447	0.554975
C	-1.518642	3.836919	-0.213653
C	-4.124553	0.303991	-2.641599
C	-4.493261	-1.034965	-1.947548
O	-3.469200	-1.136621	-0.946083
C	-4.801192	0.809968	2.823041
H	-5.332060	0.268205	2.031678
H	-5.267263	0.569981	3.789807
H	-4.932523	1.884987	2.639461
C	-2.575223	1.381317	3.791051
H	-2.935258	1.273550	4.823162
H	-1.496422	1.176864	3.766431
H	-2.725769	2.426523	3.490258
C	-4.031614	-1.967355	2.427941
H	-4.975300	-1.962600	2.990595
H	-4.230125	-1.698880	1.381114
H	-3.628299	-2.989311	2.440990
C	-2.715974	-1.432783	4.474788
H	-1.860923	-0.887195	4.888835
H	-3.593718	-1.246626	5.110762
H	-2.486432	-2.505233	4.521682
C	-4.640453	1.522284	-1.882617
H	-4.131731	2.412051	-2.275113
H	-5.725743	1.649706	-1.999394
H	-4.396248	1.455145	-0.812198
C	-4.510306	0.395053	-4.103461
H	-5.598250	0.295594	-4.229782
H	-4.211058	1.372409	-4.502734
H	-4.015052	-0.378187	-4.701474
C	-5.848033	-1.053975	-1.269268
H	-6.654708	-0.878006	-1.995638
H	-6.019872	-2.034022	-0.803788
H	-5.911188	-0.291039	-0.484604
C	-4.350423	-2.241563	-2.865958
H	-5.136889	-2.273062	-3.632023
H	-3.372794	-2.241568	-3.366706
H	-4.427311	-3.157787	-2.264662
C	-2.725608	3.949814	0.707515
H	-3.637500	3.822413	0.109162
H	-2.713796	3.153516	1.460259
H	-2.771619	4.928816	1.203827
C	-1.580255	4.926169	-1.267389
H	-2.562036	4.904915	-1.758095
H	-1.450268	5.919449	-0.813441
H	-0.817870	4.791103	-2.042360
C	1.030571	4.147870	-0.297903
H	1.072060	5.231730	-0.470425
H	1.956724	3.849952	0.214729
H	1.002251	3.644040	-1.274165
C	-0.126986	4.482067	1.878500
H	-0.299724	5.558364	1.734130
H	-0.879714	4.095871	2.575054
H	0.859375	4.356561	2.343953
H	-0.164829	0.514946	-1.737200
C	3.000235	-0.951786	-2.114745
O	2.904407	-2.094868	-1.677403
C	4.341378	-0.239408	-2.180389
H	4.591636	-0.010591	-3.229780
H	4.255212	0.741123	-1.683449
C	0.585221	-0.792757	-2.413090
H	0.013170	-0.616239	-3.332157
H	0.682777	-1.869898	-2.259744
N	1.916612	-0.242099	-2.556755
C	2.003310	1.151126	-2.937853
H	1.108911	1.417645	-3.512787
H	2.054028	1.820662	-2.060675
H	2.875088	1.345449	-3.571156
C	5.464462	-1.029382	-1.534213
H	5.133941	-1.369710	-0.540738
H	5.655872	-1.950753	-2.104163
C	6.734635	-0.206603	-1.391430
H	7.125253	0.067744	-2.386889
H	6.494112	0.748711	-0.893228
C	7.818322	-0.916157	-0.594635
H	7.396884	-1.231687	0.374816
H	8.109029	-1.844297	-1.112746
C	9.035575	-0.038724	-0.358569
H	9.818638	-0.560517	0.205577
H	9.474884	0.294085	-1.309470
H	8.758830	0.861976	0.208052
C	4.511083	1.281289	1.473090
F	4.426072	2.107673	0.418254
F	4.262065	2.015587	2.560787
F	5.779530	0.860570	1.554911
H	-2.158964	-5.381650	0.167135

Structure **L2_IrB3_IC**

C	1.640287	-0.700750	2.017468
C	2.303068	0.801369	0.386926
C	3.543774	0.972364	0.987724
C	3.816483	0.290864	2.168592
C	2.851304	-0.548889	2.693272
C	0.575094	-1.612111	2.473157

C	-1.537387	-2.506311	2.055631
C	-1.503435	-3.247374	3.227380
C	-0.381525	-3.162773	4.043034
C	0.662463	-2.335328	3.661980
H	2.017867	1.339556	-0.517011
H	4.782835	0.400090	2.656470
H	3.061942	-1.106877	3.600720
H	-2.392180	-2.520344	1.379665
H	-0.318642	-3.732339	4.967897
H	1.544433	-2.252465	4.290441
N	1.383721	-0.019764	0.888603
N	-0.521284	-1.710902	1.683892
Ir	-0.554281	-0.592320	-0.166396
O	-3.007577	0.360184	1.470650
O	-3.249581	-2.001488	-0.464330
O	-0.037156	2.277131	-1.028969
B	-2.226534	-1.275350	-1.071046
B	-1.713709	0.683853	1.121000
B	-1.034245	1.318326	-0.844521
O	-2.254566	1.790388	-1.311783
O	-1.199036	1.654526	1.966344
C	-2.110215	1.794288	3.072127
C	-3.452778	1.300334	2.459401
C	-0.665777	3.479397	-1.501797
C	-1.973114	2.929189	-2.140496
C	-4.388350	-1.920397	-1.336740
C	-3.710176	-1.803555	-2.730035
O	-2.503810	-1.096480	-2.419996
C	-4.373542	0.587103	3.429601
H	-3.903564	-0.312062	3.845352
H	-4.663616	1.249144	4.258256
H	-5.288048	0.276369	2.907934
C	-4.216750	2.389891	1.720175
H	-3.566731	2.910843	1.005159
H	-5.025888	1.922420	1.144378
H	-4.655657	3.125305	2.407920
C	-1.588361	0.886754	4.179015
H	-2.168544	0.990279	5.105759
H	-1.608385	-0.168093	3.871258
H	-0.545255	1.156768	4.391133
C	-2.109583	3.233439	3.545232
H	-1.117490	3.495151	3.934777
H	-2.347193	3.931637	2.735176
H	-2.841002	3.377102	4.353690
C	-5.254486	-3.147732	-1.146670
H	-6.084287	-3.154172	-1.868099
H	-4.679819	-4.072771	-1.267857
H	-5.686339	-3.145364	-0.137333
C	-5.158039	-0.664314	-0.941915
H	-4.549332	0.238191	-1.095024
H	-6.097699	-0.568065	-1.503231
H	-5.392145	-0.723961	0.129196
C	-3.304991	-3.151650	-3.310671
H	-2.755598	-3.749552	-2.571422
H	-4.171120	-3.730442	-3.659579
H	-2.638310	-2.978857	-4.165404
C	-4.495615	-1.006763	-3.752588
H	-4.656369	0.023509	-3.413759
H	-3.939179	-0.968756	-4.698387
H	-5.472751	-1.470837	-3.951076
C	-3.157430	3.872197	-2.103631
H	-3.415200	4.167048	-1.080125
H	-2.952535	4.780885	-2.687607
H	-4.033646	3.376585	-2.542296
C	-1.769870	2.403711	-3.556330
H	-2.657228	1.827805	-3.846534
H	-1.626233	3.217351	-4.279984
H	-0.907706	1.724933	-3.612094
C	0.268756	4.173811	-2.471706
H	0.596178	3.498718	-3.270222
H	-0.220448	5.046217	-2.928361
H	1.161673	4.527571	-1.940285
C	-0.916783	4.368910	-0.292707
H	-1.336499	5.342401	-0.579930
H	-1.591911	3.880399	0.421180
H	0.036315	4.539536	0.223308
H	-0.367934	-0.019482	-1.674789
C	2.929937	-2.014976	-0.984059
O	2.992267	-2.488813	0.150658
C	4.179045	-1.480362	-1.671247
H	4.341105	-2.006343	-2.626557
H	4.018270	-0.423801	-1.947720
C	0.501164	-2.295895	-1.033860
H	-0.109493	-2.799878	-1.797338
H	0.739860	-3.023652	-0.250941
N	1.760988	-1.926496	-1.678130
C	1.653991	-1.231021	-2.941457
H	0.725268	-1.543776	-3.434438
H	1.604376	-0.133942	-2.817096
H	2.490412	-1.469783	-3.607357
C	5.418502	-1.597035	-0.802841
H	5.215402	-1.143642	0.179773
H	5.622704	-2.657790	-0.591919
C	6.634145	-0.934275	-1.429150
H	6.831305	-1.371471	-2.424052
H	6.417517	0.133557	-1.609517
C	7.884323	-1.052168	-0.570605
H	7.675197	-0.634003	0.427470
H	8.113537	-2.117826	-0.407295
C	9.083698	-0.348965	-1.182968
H	9.321025	-0.763262	-2.173043
H	8.882626	0.723217	-1.317163
H	9.981145	-0.444014	-0.558834
C	4.546650	1.905025	0.386259
F	5.798726	1.531555	0.679432
F	4.439093	1.949617	-0.947279
F	4.391943	3.154497	0.837008
H	-2.347961	-3.880972	3.485465

Structure **L2_IrB3_TS-RE**

C	1.819711	-1.475671	0.602757
C	1.665024	0.840445	0.791558
C	2.823919	0.901499	1.544806
C	3.477705	-0.279393	1.881642
C	2.959851	-1.471345	1.414514
C	1.301213	-2.721436	0.005227
C	-0.376322	-3.710561	-1.222056
C	0.260841	-4.947007	-1.197861
C	1.477705	-5.050167	-0.538577
C	2.007366	-3.924172	0.074274
H	1.098552	1.737447	0.536625
H	4.377568	-0.259099	2.495485
H	3.452325	-2.404999	1.670394
H	-1.340335	-3.568174	-1.708419
H	2.016646	-5.995007	-0.505119
H	2.969289	-3.989372	0.576484

N	1.171537	-0.321128	0.326282
N	0.132460	-2.626731	-0.640244
Ir	-0.660322	-0.266509	-0.829110
O	-1.481482	-1.086549	2.032647
O	-3.685144	-0.319474	0.008104
O	-0.019092	2.712559	-0.759146
B	-2.605334	-0.939828	-0.613546
B	-1.345826	-0.066897	1.100246
B	-0.621330	1.679074	-1.515615
O	-1.656160	2.247717	-2.295128
O	-1.397682	1.169766	1.715743
C	-1.828192	0.984500	3.073374
C	-1.448124	-0.504011	3.346291
C	-0.768634	3.919822	-0.932247
C	-2.086487	3.420726	-1.610649
C	-4.850772	-1.127561	-0.225026
C	-4.232668	-2.531706	-0.477455
O	-2.976234	-2.195774	-1.084642
C	-0.026525	-0.675670	3.865165
H	0.078929	-0.330955	4.902444
H	0.236605	-1.740830	3.824575
H	0.693026	-0.125433	3.243411
C	-2.413764	-1.255279	4.240524
H	-2.460235	-0.800122	5.240367
H	-3.425567	-1.270721	3.818497
H	-2.079547	-2.294779	4.354717
C	-1.111771	1.994194	3.949475
H	-1.449658	3.007516	3.697083
H	-1.331500	1.820111	5.012797
H	-0.024875	1.960972	3.804273
C	-3.327256	1.244409	3.095334
H	-3.509580	2.261390	2.722236
H	-3.852460	0.550665	2.426582
H	-3.746292	1.167391	4.107715
C	-5.764528	-1.044319	0.980243
H	-6.136531	-0.017705	1.094385
H	-6.631980	-1.708773	0.858113
H	-5.243037	-1.321160	1.904441
C	-5.548701	-0.550533	-1.449254
H	-5.775647	0.505853	-1.258405
H	-4.901039	-0.599396	-2.334572
H	-6.490209	-1.070944	-1.670508
C	-3.918022	-3.291763	0.804596
H	-3.301720	-4.164359	0.548733
H	-3.338251	-2.669903	1.499963
H	-4.827775	-3.648989	1.306133
C	-5.018044	-3.411544	-1.428371
H	-5.116094	-2.952956	-2.418286
H	-4.504784	-4.374502	-1.550825
H	-6.023613	-3.614018	-1.032548
C	-3.162841	3.014229	-0.607578
H	-2.775640	2.318500	0.146877
H	-3.599657	3.887313	-0.101332
H	-3.961037	2.493087	-1.153089
C	-2.683121	4.387123	-2.617591
H	-1.995840	4.593000	-3.445642
H	-3.597145	3.951878	-3.041501
H	-2.952939	5.339286	-2.137716
C	0.043026	4.847692	-1.825970
H	-0.429892	5.833784	-1.929154
H	1.038189	4.991637	-1.383963
H	0.169657	4.420572	-2.830236
C	-0.955447	4.553667	0.434184
H	-1.596719	5.445337	0.377769
H	-1.394482	3.834986	1.136432
H	0.021892	4.860436	0.831891
H	-1.983550	-0.124666	-1.795018
C	2.841878	-0.104558	-2.231792
O	2.450804	-1.262222	-2.366699
C	4.275087	0.194009	-1.818879
H	4.806595	0.705710	-2.638399
H	4.273721	0.916531	-0.984306
C	0.593207	0.739737	-2.626832
H	0.257489	1.375496	-3.456905
H	0.490596	-0.288324	-3.006832
N	2.012531	0.967070	-2.385446
C	2.498792	2.332129	-2.337411
H	2.550266	2.736410	-1.315394
H	3.487522	2.411516	-2.802768
H	1.812499	2.970395	-2.902191
C	5.036277	-1.056734	-1.412401
H	4.395333	-1.663925	-0.755936
H	5.222411	-1.686266	-2.296140
C	6.345021	-0.719639	-0.714141
H	6.946963	-0.064105	-1.364766
H	6.125786	-0.118941	0.189164
C	7.170950	-1.941449	-0.327665
H	7.392918	-2.527305	-1.234202
H	8.143982	-1.612218	0.066267
C	6.494824	-2.837720	0.699948
H	5.556056	-3.260359	0.314134
H	7.137528	-3.679031	0.987435
H	6.258633	-2.275917	1.616710
C	3.408722	2.213677	1.955095
F	4.592935	2.402741	1.349244
F	2.623953	3.243013	1.641258
F	3.639600	2.253409	3.272554
H	-0.189637	-5.803367	-1.694115

Structure **L2_IrB3_PC**

C	1.252761	-3.089707	-0.520619
C	2.725456	-1.337353	-0.890445
C	3.809882	-2.201162	-0.930407
C	3.591156	-3.567090	-0.771537
C	2.299111	-4.015925	-0.565431
C	-0.141483	-3.472998	-0.224032
C	-2.234157	-2.755280	0.467207
C	-2.724887	-4.052828	0.492457
C	-1.882589	-5.093191	0.119851
C	-0.573514	-4.798629	-0.230598
H	2.842025	-0.260187	-1.023262
H	4.428140	-4.263371	-0.794573
H	2.112005	-5.074760	-0.407629
H	-2.837616	-1.900711	0.774631
H	-2.235504	-6.122358	0.110002
H	0.104808	-5.596610	-0.522502
N	1.479471	-1.774695	-0.689134
N	-0.982772	-2.462545	0.089908
Ir	-0.272142	-0.420710	-0.177971
O	0.685985	-1.325556	2.489100
O	-2.574156	0.283371	1.665485
O	1.211266	3.498194	-1.790190
B	-1.795002	0.666178	0.555289
B	0.340520	-0.223505	1.699024
B	1.546575	2.223619	-2.145655
O	2.684286	1.772754	-1.533672

O	0.585215	0.958464	2.405379
C	0.899306	0.610695	3.758854
C	1.433965	-0.842429	3.608824
C	2.014497	3.864064	-0.645939
C	3.196608	2.826908	-0.693285
C	-3.672126	1.191923	1.798210
C	-3.145482	2.456588	1.061181
O	-2.263380	1.899316	0.081788
C	2.905953	-0.903359	3.214704
H	3.111320	-0.259276	2.347723
H	3.573049	-0.610541	4.037012
H	3.144921	-1.935956	2.926526
C	1.184265	-1.739877	4.804486
H	0.113139	-1.851613	5.004726
H	1.596866	-2.738730	4.611477
H	1.672140	-1.340213	5.705527
C	1.898693	1.613573	4.300363
H	1.434158	2.608214	4.336545
H	2.214191	1.347729	5.319830
H	2.789590	1.683298	3.665073
C	-0.395840	0.682344	4.558457
H	-1.147401	-0.007245	4.150901
H	-0.237648	0.459468	5.622444
H	-0.800023	1.701393	4.481443
C	-4.869998	0.552734	1.106265
H	-4.664701	0.399435	0.038299
H	-5.058629	-0.430165	1.560484
H	-5.783708	1.155191	1.204969
C	-3.983791	1.392439	3.268479
H	-4.780735	2.138547	3.402872
H	-4.328045	0.446065	3.706371
H	-3.100493	1.718706	3.828214
C	-4.207405	3.278290	0.356359
H	-4.940205	3.679244	1.071822
H	-3.734115	4.127406	-0.154600
H	-4.744390	2.690289	-0.398385
C	-2.302582	3.355301	1.958848
H	-1.834280	4.128932	1.336677
H	-2.903575	3.853157	2.732202
H	-1.494434	2.782111	2.436621
C	4.449684	3.341791	-1.384822
H	4.219981	3.739403	-2.382023
H	4.948917	4.125851	-0.800940
H	5.150563	2.506835	-1.510980
C	3.547755	2.221454	0.651866
H	4.333528	1.464877	0.516387
H	3.930034	2.990649	1.338967
H	2.672875	1.742981	1.111806
C	1.116835	3.740129	0.575575
H	0.260815	4.413840	0.437454
H	0.723978	2.725160	0.707089
H	1.640181	4.032725	1.496895
C	2.445297	5.309117	-0.811567
H	3.094295	5.616277	0.020666
H	2.978871	5.479296	-1.753069
H	1.558865	5.955395	-0.804084
H	0.329275	1.051127	-0.449993
C	-1.343563	0.384791	-2.864568
O	-0.799020	-0.692868	-2.559288
C	-2.850239	0.525463	-2.849555
H	-3.112531	1.372907	-2.195362
H	-3.196013	0.796216	-3.861788
C	0.828013	1.396607	-3.285391
H	1.107929	0.340580	-3.210311
H	1.170062	1.751036	-4.273097
N	-0.633742	1.462010	-3.268982
C	-1.245398	2.774411	-3.397350
H	-1.525714	3.177796	-2.412950
H	-0.517670	3.455561	-3.847978
H	-2.129327	2.740811	-4.045041
C	-3.555375	-0.727408	-2.367384
H	-3.214640	-1.602455	-2.941162
H	-3.243209	-0.919653	-1.327930
C	-5.068681	-0.608825	-2.434130
H	-5.392684	0.321910	-1.932944
H	-5.395512	-0.503268	-3.483455
C	-5.780130	-1.792109	-1.794845
H	-5.527547	-2.710905	-2.348931
H	-5.383262	-1.938278	-0.775705
C	-7.286264	-1.605922	-1.733221
H	-7.708094	-1.446280	-2.735543
H	-7.793488	-2.473289	-1.292543
H	-7.542344	-0.725537	-1.125662
C	5.199496	-1.697622	-1.154373
F	6.038454	-2.175468	-0.225882
F	5.672382	-2.097023	-2.341575
F	5.267399	-0.364703	-1.118436
H	-3.752272	-4.235311	0.798033

Structure **L2_IrB3_IC_TS-isoH**

C	1.274142	0.690892	-2.137097
C	2.352209	-0.701647	-0.659183
C	3.198501	-1.175918	-1.648640
C	3.065973	-0.680469	-2.943588
C	2.091137	0.268746	-3.190364
C	0.197161	1.674024	-2.317217
C	-1.744089	2.610144	-1.479780
C	-1.777464	3.564095	-2.483120
C	-0.772247	3.566931	-3.438658
C	0.220129	2.602735	-3.355133
H	2.389094	-1.088372	0.356499
H	3.708575	-1.046750	-3.742831
H	1.936106	0.648279	-4.197085
H	-2.518456	2.569862	-0.722190
H	-0.748561	4.317613	-4.224976
H	1.045259	2.599009	-4.063056
N	1.415939	0.216038	-0.891921
N	-0.780652	1.679448	-1.383204
Ir	-0.727174	0.434807	0.359564
O	-1.372450	-2.599520	-0.083887
O	-3.514804	0.324588	-0.807089
O	1.073663	-1.405642	1.943882
B	-2.709794	-0.001162	0.284977
B	-1.004604	-1.389020	-0.652559
B	0.124933	-0.414106	2.112329
O	0.061656	0.002632	3.422469
O	-0.570456	-1.578600	-1.958199
C	-0.802363	-2.950420	-2.316317
C	-0.863194	-3.646381	-0.925900
C	1.860310	-1.450000	3.154750
C	0.848384	-0.914697	4.205766
C	-4.881088	0.217934	-0.374450
C	-4.779319	-0.820084	0.780144
O	-3.469660	-0.558261	1.302521
C	-1.788983	-4.843358	-0.847721
H	-1.780588	-5.249210	0.171973

H	-2.822952	-4.577550	-1.094153
H	-1.459182	-5.638308	-1.532266
C	0.511261	-4.020751	-0.382258
H	1.208568	-3.172895	-0.435180
H	0.407047	-4.290079	0.676462
H	0.951829	-4.870650	-0.921128
C	-2.124590	-2.992543	-3.069570
H	-2.057058	-2.325462	-3.938025
H	-2.367171	-4.002673	-3.425821
H	-2.947629	-2.629627	-2.438727
C	0.322230	-3.421322	-3.218970
H	0.294018	-2.861107	-4.163258
H	1.310023	-3.268741	-2.766378
H	0.211153	-4.489200	-3.457036
C	-5.311237	1.598930	0.104001
H	-6.370188	1.624757	0.393300
H	-4.709002	1.926896	0.962591
H	-5.165433	2.318020	-0.713490
C	-5.736627	-0.212405	-1.548140
H	-6.773522	-0.392733	-1.230011
H	-5.748318	0.577550	-2.310045
H	-5.350032	-1.124537	-2.015805
C	-5.798731	-0.642178	1.886935
H	-5.701126	0.333598	2.375046
H	-6.821628	-0.741084	1.495694
H	-5.652354	-1.415833	2.651159
C	-4.790777	-2.260520	0.287814
H	-4.487745	-2.916749	1.113412
H	-5.785862	-2.568148	-0.060953
H	-4.063206	-2.402531	-0.521110
C	-0.099464	-1.990245	4.717114
H	0.409008	-2.690108	5.393664
H	-0.915114	-1.507171	5.268436
H	-0.543450	-2.558740	3.889242
C	1.463268	-0.163268	5.367774
H	2.005810	0.727261	5.032639
H	0.672667	0.164194	6.054120
H	2.153551	-0.809527	5.928430
C	3.054499	-0.522075	2.968204
H	2.728776	0.511338	2.778759
H	3.706611	-0.523368	3.851195
H	3.658464	-0.855666	2.112847
C	2.335029	-2.873199	3.356029
H	2.901031	-2.969626	4.293328
H	1.499473	-3.581130	3.374756
H	2.998209	-3.154992	2.526344
H	-1.243085	-0.402639	1.672221
C	0.504064	4.287862	0.092067
O	0.184034	5.393517	-0.343607
C	1.841845	3.678926	-0.278251
H	1.773461	2.588114	-0.341804
H	2.096003	4.065973	-1.276806
C	0.023842	2.239798	1.488402
H	1.109467	2.166452	1.648838
H	-0.419062	2.219722	2.491167
N	-0.286493	3.560109	0.932765
C	-1.529520	4.144089	1.383052
H	-1.801548	4.972170	0.722257
H	-2.317415	3.375876	1.383969
H	-1.436884	4.524853	2.412100
C	2.946330	4.061638	0.705732
H	2.988423	5.158133	0.783703
H	2.683478	3.690183	1.710915
C	4.310953	3.504461	0.309368
H	4.663688	3.998923	-0.612046
H	5.046493	3.759483	1.089810
C	4.309248	1.994668	0.093887
H	3.753811	1.512302	0.917740
H	3.742750	1.757317	-0.820670
C	5.698087	1.389763	-0.012523
H	6.273547	1.858341	-0.823568
H	6.267982	1.530346	0.916453
H	5.655542	0.310305	-0.219085
C	4.186858	-2.256307	-1.354819
F	3.802337	-3.421155	-1.896512
F	5.389885	-1.967945	-1.865545
F	4.340424	-2.456899	-0.041688
H	-2.569485	4.307641	-2.486247

Structure **L2_IrB3_IC-isoH**

C	1.756683	-1.950062	0.606722
C	2.241557	0.070637	1.621966
C	3.521954	-0.350873	1.949633
C	3.920046	-1.633634	1.599302
C	3.026508	-2.440941	0.917902
C	0.769368	-2.724883	-0.158715
C	-1.279638	-2.734958	-1.259729
C	-1.156295	-4.074018	-1.587101
C	-0.019970	-4.761460	-1.172495
C	0.948519	-4.079444	-0.456664
H	1.897119	1.080346	1.848511
H	4.920532	-1.987544	1.838451
H	3.335063	-3.433707	0.599987
H	-2.133502	-2.143634	-1.577082
H	0.109772	-5.817277	-1.401625
H	1.836224	-4.601496	-0.110155
N	1.374211	-0.711302	0.978659
N	-0.348307	-2.070690	-0.556369
Ir	-0.593887	-0.014067	0.077610
O	-2.973193	-1.790890	0.860809
O	-3.369694	1.186799	-0.409076
O	0.662223	2.616728	1.002836
B	-2.290915	0.471448	-0.911562
B	-2.074056	-0.840249	1.341162
B	-0.275829	2.036716	0.162234
O	-0.853843	2.982508	-0.657525
O	-2.328881	-0.568719	2.671511
C	-3.580844	-1.186784	3.017839
C	-3.662552	-2.347724	1.990493
C	0.879923	3.966776	0.549515
C	-0.469092	4.277602	-0.159899
C	-4.266939	1.456104	-1.493664
C	-3.946244	0.300037	-2.484371
O	-2.555350	0.046854	-2.214841
C	-2.876151	-3.583380	2.412192
H	-3.369034	-4.134270	3.224562
H	-2.784895	-4.251914	1.545352
H	-1.862177	-3.312180	2.736599
C	-5.064831	-2.739664	1.573068
H	-5.605001	-1.896361	1.126786
H	-5.019016	-3.544608	0.827273
H	-5.642091	-3.106923	2.433793
C	-3.538469	-1.620750	4.468114
H	-3.476004	-0.738245	5.117093
H	-4.449794	-2.175014	4.736063
H	-2.668581	-2.252935	4.677685

C	-4.662030	-0.135060	2.809755
H	-5.655831	-0.501850	3.100826
H	-4.422925	0.739415	3.427504
H	-4.687954	0.195931	1.762234
C	-5.687891	1.464736	-0.965910
H	-5.931125	0.534131	-0.438844
H	-5.811472	2.294055	-0.257532
H	-6.411308	1.603786	-1.782448
C	-3.908526	2.829029	-2.047460
H	-3.979097	3.561332	-1.232642
H	-2.875119	2.847073	-2.416156
H	-4.590111	3.138732	-2.851351
C	-4.702410	-0.983479	-2.164746
H	-4.562608	-1.268743	-1.112290
H	-5.776647	-0.892987	-2.374580
H	-4.305651	-1.793096	-2.793291
C	-4.110242	0.650111	-3.949103
H	-3.857101	-0.218922	-4.570282
H	-5.150099	0.931591	-4.169582
H	-3.453910	1.476467	-4.242246
C	-1.558751	4.723098	0.805834
H	-1.642096	4.035250	1.657455
H	-1.378733	5.737115	1.187317
H	-2.520953	4.717542	0.278692
C	-0.378322	5.241150	-1.324251
H	-0.022023	6.226130	-0.990279
H	0.293999	4.871243	-2.105896
H	-1.373835	5.372263	-1.768958
C	2.070417	3.944495	-0.399537
H	1.843776	3.399402	-1.324132
H	2.394710	4.960002	-0.664397
H	2.905132	3.427431	0.092238
C	1.194446	4.834795	1.750252
H	1.267440	5.892251	1.458531
H	0.434044	4.737270	2.532634
H	2.160297	4.534491	2.177405
H	-1.089315	1.008907	1.234261
C	2.790792	-0.649591	-1.992283
O	3.371522	0.052715	-1.164983
C	3.521698	-1.775546	-2.716336
H	2.971801	-2.719164	-2.562258
H	3.494238	-1.596890	-3.805058
C	0.729138	0.606944	-1.664729
H	1.484338	1.322106	-1.324073
H	0.102915	1.082854	-2.429452
N	1.471290	-0.480163	-2.289505
C	0.803908	-1.209262	-3.348742
H	0.948317	-2.295049	-3.263495
H	-0.269612	-1.001762	-3.282968
H	1.150929	-0.894386	-4.345752
C	4.958105	-1.916734	-2.235866
H	4.947006	-1.963126	-1.137084
H	5.368742	-2.876948	-2.586703
C	5.866119	-0.779803	-2.695212
H	6.114987	-0.928021	-3.758937
H	5.314251	0.168367	-2.626864
C	7.144565	-0.655759	-1.873066
H	7.675316	-1.623120	-1.861143
H	7.823451	0.053470	-2.370335
C	6.891279	-0.185865	-0.446458
H	6.322305	-0.925663	0.133527
H	7.829570	-0.002230	0.092337
H	6.300992	0.740684	-0.436789

C	4.436275	0.617364	2.630396
F	5.671117	0.123795	2.768374
F	4.528672	1.760537	1.943460
F	3.987936	0.933643	3.851764
H	-1.941115	-4.561864	-2.159163

Structure **L2_IrB3_IC-isoH_TS-RE**

C	2.296381	-0.850652	0.987449
C	1.979509	1.357260	1.625365
C	3.277769	1.450426	2.104705
C	4.108712	0.335422	2.021374
C	3.612155	-0.827875	1.461635
C	1.708829	-2.019967	0.309888
C	-0.002214	-2.802007	-1.048092
C	0.591246	-4.051489	-1.152472
C	1.769833	-4.292024	-0.461502
C	2.337379	-3.262252	0.275006
H	1.295480	2.206159	1.632556
H	5.133270	0.386715	2.385065
H	4.253480	-1.700026	1.362119
H	-0.930584	-2.553683	-1.559860
H	2.250749	-5.267776	-0.498409
H	3.267783	-3.426732	0.813717
N	1.504656	0.234374	1.084165
N	0.530928	-1.817330	-0.319536
Ir	-0.504208	0.059135	0.046081
O	-1.944658	-0.503761	2.758716
O	-2.379031	-0.989197	-2.079413
O	-0.063472	3.105469	-0.048691
B	-2.277417	-0.314351	-0.863533
B	-1.538987	-0.982186	1.527107
B	-0.756878	2.012266	-0.602118
O	-1.833053	2.475281	-1.356591
O	-1.921767	-2.313519	1.370180
C	-2.388349	-2.785671	2.642900
C	-2.839926	-1.469058	3.334446
C	-0.914934	4.255763	-0.174824
C	-1.749392	3.902947	-1.439339
C	-3.754203	-1.375025	-2.249277
C	-4.498029	-0.309651	-1.395672
O	-3.541368	-0.026110	-0.365557
C	-4.251396	-1.047500	2.948851
H	-4.423049	-0.027634	3.315257
H	-4.368251	-1.028104	1.857005
H	-5.013230	-1.705563	3.388905
C	-2.683917	-1.455271	4.841467
H	-1.640499	-1.596717	5.144142
H	-3.020463	-0.489248	5.238556
H	-3.294943	-2.243541	5.304979
C	-3.488057	-3.803710	2.421551
H	-3.915816	-4.134808	3.378960
H	-4.295894	-3.396214	1.802245
H	-3.080752	-4.684933	1.908458
C	-1.196096	-3.443249	3.327206
H	-1.466702	-3.900757	4.288404
H	-0.803670	-4.229168	2.668500
H	-0.392199	-2.713972	3.498300
C	-4.093635	-1.363611	-3.725189
H	-5.161413	-1.574747	-3.882147
H	-3.854967	-0.398947	-4.185507
H	-3.515173	-2.137597	-4.245913
C	-3.890092	-2.785188	-1.687600
H	-3.606787	-2.812555	-0.625305

H	-4.909382	-3.178520	-1.800219
H	-3.211059	-3.452417	-2.236388
C	-4.748323	0.989917	-2.149636
H	-3.825395	1.360484	-2.613328
H	-5.525336	0.875179	-2.917837
H	-5.080817	1.749584	-1.430259
C	-5.785619	-0.792328	-0.757772
H	-6.519801	-1.079294	-1.524646
H	-5.617758	-1.652114	-0.097947
H	-6.222312	0.014168	-0.154310
C	-3.153303	4.475472	-1.450994
H	-3.660585	4.196457	-2.384053
H	-3.749961	4.090693	-0.616651
H	-3.129807	5.573407	-1.394092
C	-1.042688	4.262872	-2.740345
H	-1.563160	3.767016	-3.569913
H	-1.053576	5.345774	-2.923871
H	0.001984	3.924782	-2.747075
C	-0.052498	5.495720	-0.297762
H	0.505290	5.653387	0.634583
H	0.673458	5.409150	-1.114694
H	-0.672009	6.386448	-0.474993
C	-1.761139	4.334452	1.089892
H	-2.415426	3.458577	1.189538
H	-1.093234	4.356471	1.960651
H	-2.382313	5.239814	1.111330
H	-1.339724	1.172761	0.858440
C	2.728516	-0.076437	-2.270382
O	2.237651	-1.140785	-2.639337
C	4.237307	0.143000	-2.280938
H	4.463344	1.028258	-2.895621
H	4.575784	0.403671	-1.262401
C	0.514568	0.892391	-1.895554
H	0.157403	1.728477	-2.513344
H	0.257844	-0.005158	-2.467327
N	1.972533	0.975022	-1.832071
C	2.563695	2.269966	-1.545203
H	1.830921	2.878079	-1.008402
H	3.451848	2.184570	-0.905963
H	2.854426	2.803523	-2.466090
C	5.015716	-1.061298	-2.785917
H	4.575065	-1.406850	-3.732428
H	6.048462	-0.747889	-3.008605
C	5.043022	-2.217123	-1.799298
H	5.420582	-1.855941	-0.822833
H	4.009706	-2.557570	-1.634768
C	5.896996	-3.388831	-2.255404
H	5.546784	-3.728381	-3.243172
H	6.937042	-3.054120	-2.401817
C	5.856646	-4.544420	-1.269458
H	4.830608	-4.925826	-1.160289
H	6.490512	-5.383466	-1.581928
H	6.196043	-4.224927	-0.272532
C	3.823564	2.754351	2.591524
F	2.860189	3.602282	2.949458
F	4.635694	2.582306	3.640489
F	4.548180	3.348120	1.631466
H	0.122612	-4.820249	-1.761121

Structure **L2_IrB3_PC-isoH**

C	1.192553	-3.014940	-0.938265
C	2.648452	-1.227210	-1.134887
C	3.751401	-2.072203	-1.160694
C	3.549716	-3.446104	-1.094914
C	2.251767	-3.922172	-0.990627
C	-0.202301	-3.430172	-0.696467
C	-2.285281	-2.802846	0.098118
C	-2.780986	-4.091226	-0.043130
C	-1.945740	-5.076190	-0.556045
C	-0.637308	-4.742386	-0.873333
H	2.754746	-0.142215	-1.204573
H	4.394802	-4.130665	-1.113814
H	2.070705	-4.990520	-0.903447
H	-2.882436	-1.996299	0.523599
H	-2.303721	-6.093658	-0.699187
H	0.039185	-5.494104	-1.273497
N	1.402566	-1.687930	-1.023937
N	-1.037146	-2.465413	-0.249641
Ir	-0.323304	-0.400628	-0.285418
O	0.765106	0.605786	2.383904
O	-2.463518	0.043519	1.809833
O	2.587120	1.901905	-1.525047
B	-1.769817	0.576405	0.706030
B	0.452872	-0.464119	1.539817
B	1.433416	2.460115	-2.005323
O	1.174123	3.693703	-1.480157
O	0.874077	-1.664382	2.122793
C	1.716361	-1.341590	3.233778
C	1.197584	0.068310	3.639522
C	3.196449	2.836280	-0.610503
C	2.055055	3.885047	-0.350837
C	-3.530144	0.931298	2.159563
C	-3.042913	2.284521	1.566924
O	-2.254602	1.866187	0.448024
C	-0.023309	0.009234	4.549619
H	-0.810149	-0.619612	4.111241
H	0.224940	-0.365505	5.551932
H	-0.429027	1.024802	4.657643
C	2.241990	0.992797	4.232997
H	2.641415	0.586218	5.173586
H	3.076867	1.160451	3.542315
H	1.785187	1.967692	4.450473
C	1.566137	-2.406239	4.302268
H	0.515417	-2.557348	4.572408
H	1.959597	-3.361826	3.931711
H	2.128648	-2.136922	5.208176
C	3.150117	-1.330110	2.714536
H	3.289300	-0.564914	1.937856
H	3.883300	-1.154424	3.513685
H	3.364790	-2.307159	2.259893
C	-4.792335	0.405280	1.486652
H	-5.683991	0.991704	1.748618
H	-4.680140	0.407668	0.393946
H	-4.958943	-0.632136	1.808895
C	-3.716252	0.922081	3.664371
H	-2.782015	1.153478	4.187548
H	-4.481482	1.650261	3.971055
H	-4.045440	-0.073501	3.990413
C	-4.143823	3.205061	1.077202
H	-4.752151	2.733495	0.295374
H	-4.807345	3.505030	1.901453
H	-3.698824	4.114944	0.652727
C	-2.111726	3.040364	2.507970
H	-1.279166	2.398396	2.831063
H	-1.680329	3.890228	1.963413
H	-2.637244	3.428804	3.391203

C	1.226577	3.617048	0.896687
H	0.817586	2.599814	0.917951
H	1.808275	3.777437	1.815377
H	0.380314	4.316937	0.898671
C	2.523300	5.327484	-0.349707
H	3.002606	5.609188	-1.293680
H	1.662416	5.989975	-0.196409
H	3.233839	5.501526	0.470756
C	4.409279	3.398123	-1.336129
H	4.983482	4.088400	-0.704626
H	5.062763	2.563086	-1.619881
H	4.116726	3.923440	-2.254745
C	3.629703	2.073545	0.625901
H	4.402010	1.338026	0.358190
H	4.061841	2.756669	1.372068
H	2.782201	1.546195	1.083963
H	0.271436	1.093699	-0.400001
C	-1.560240	0.790069	-2.740997
O	-1.030454	-0.331580	-2.630772
C	-3.057041	0.960568	-2.592496
H	-3.244970	1.708747	-1.805099
H	-3.465010	1.382257	-3.526935
C	0.607221	1.804301	-3.183175
H	0.858298	0.741898	-3.269097
H	0.885591	2.279800	-4.139618
N	-0.846992	1.897868	-3.045267
C	-1.424123	3.227623	-2.937620
H	-0.717929	3.950044	-3.357158
H	-2.362725	3.300255	-3.499176
H	-1.603901	3.496528	-1.885750
C	-3.765894	-0.333050	-2.241786
H	-3.495120	-1.124189	-2.957258
H	-3.386816	-0.678010	-1.266229
C	-5.275487	-0.174795	-2.175482
H	-5.532956	0.675896	-1.517806
H	-5.673571	0.094495	-3.169462
C	-5.976024	-1.426485	-1.667728
H	-5.791609	-2.256312	-2.369439
H	-5.513375	-1.731965	-0.713575
C	-7.467561	-1.221933	-1.466850
H	-7.968652	-2.135474	-1.123162
H	-7.652739	-0.437992	-0.717922
H	-7.954190	-0.905117	-2.400057
C	5.119624	-1.468806	-1.162587
F	5.471965	-1.075678	0.071650
F	6.046798	-2.336635	-1.580012
F	5.183888	-0.387518	-1.945323
H	-3.806741	-4.309064	0.244347

Structure **L2_IrB3_IC_TS-isoS**

C	1.760650	-0.673105	-1.487550
C	-0.089425	-2.043255	-1.270434
C	0.388396	-2.917520	-2.230962
C	1.598503	-2.638435	-2.856818
C	2.291931	-1.504924	-2.475093
C	2.485987	0.491868	-0.952126
C	2.588929	2.094011	0.712000
C	3.807678	2.573996	0.258446
C	4.359158	1.995557	-0.879944
C	3.694224	0.942548	-1.487122
H	-1.018573	-2.234756	-0.736929
H	1.999240	-3.312158	-3.611804
H	3.261948	-1.292871	-2.915439
H	2.089636	2.537347	1.572814
H	5.301943	2.355362	-1.289551
H	4.112404	0.476774	-2.376048
N	0.570985	-0.938158	-0.920092
N	1.938374	1.090359	0.120322
Ir	-0.125699	0.382538	0.702527
O	-3.176325	0.810552	0.005011
O	0.274891	3.010321	-0.793762
O	-2.179929	-1.836480	0.910831
B	-0.663860	2.342472	-0.012882
B	-1.870532	0.688847	-0.453160
B	-1.674524	-0.692711	1.530186
O	-2.152710	-0.594743	2.817436
O	-1.861372	0.468922	-1.826847
C	-3.208134	0.190248	-2.246279
C	-4.051174	0.876314	-1.130152
C	-2.814821	-2.649163	1.909463
C	-3.163137	-1.598892	3.007877
C	-0.167219	4.360577	-0.990421
C	-1.153328	4.558786	0.196759
O	-1.632547	3.226756	0.427143
C	-4.324251	2.349849	-1.395492
H	-5.056027	2.493896	-2.201766
H	-4.713285	2.804799	-0.475763
H	-3.399419	2.879650	-1.651678
C	-5.355350	0.174822	-0.806090
H	-5.191969	-0.856953	-0.473723
H	-5.877910	0.713550	-0.004793
H	-6.013986	0.155236	-1.686473
C	-3.411639	0.757342	-3.637238
H	-4.456222	0.638196	-3.959137
H	-3.149595	1.820397	-3.685475
H	-2.774326	0.220399	-4.351275
C	-3.372541	-1.324570	-2.277840
H	-4.359192	-1.618053	-2.661148
H	-2.612457	-1.751546	-2.946629
H	-3.235717	-1.761773	-1.278846
C	1.040958	5.277353	-0.978009
H	1.695089	5.034865	-1.825904
H	0.736812	6.329807	-1.072305
H	1.628553	5.167128	-0.059333
C	-0.833810	4.415455	-2.357686
H	-1.693918	3.735160	-2.408380
H	-1.167403	5.429725	-2.614712
H	-0.111053	4.085696	-3.114313
C	-0.463375	4.996820	1.483248
H	-0.109915	6.035445	1.432850
H	-1.178611	4.910843	2.310187
H	0.389563	4.344047	1.715782
C	-2.327961	5.472499	-0.088932
H	-2.984149	5.512630	0.789736
H	-1.982961	6.494225	-0.304311
H	-2.925126	5.120934	-0.937241
C	-4.505827	-0.919574	2.786925
H	-5.347567	-1.608027	2.943054
H	-4.598656	-0.090239	3.499000
H	-4.560619	-0.494690	1.777498
C	-3.070473	-2.121753	4.427364
H	-2.060163	-2.473740	4.664813
H	-3.321976	-1.319612	5.132468
H	-3.778022	-2.948591	4.585993
C	-1.785650	-3.682383	2.350135
H	-1.437801	-4.227859	1.461518

H	-0.910482	-3.206699	2.811608
H	-2.206815	-4.408155	3.058572
C	-3.999299	-3.351403	1.276637
H	-3.642048	-4.010178	0.472450
H	-4.532912	-3.968771	2.013382
H	-4.710311	-2.639608	0.840686
H	-0.475614	1.250478	2.010137
C	3.278505	-1.343528	1.648182
O	3.059582	-2.070878	0.677372
C	4.704227	-1.063073	2.109436
H	4.924313	0.008711	1.958370
H	4.773732	-1.223903	3.195759
C	0.879666	-1.052023	2.026496
H	0.857238	-2.040379	1.548343
H	0.351816	-1.122607	2.988017
N	2.275803	-0.760014	2.352648
C	2.477091	0.104342	3.492314
H	3.416473	0.664247	3.425081
H	1.651212	0.827782	3.525661
H	2.470696	-0.454882	4.442316
C	5.730633	-1.928979	1.388082
H	6.648562	-1.972083	1.996316
H	5.337004	-2.951905	1.330897
C	6.095698	-1.450766	-0.013035
H	6.644886	-2.252074	-0.534236
H	5.170238	-1.291234	-0.591277
C	6.950196	-0.190583	-0.034194
H	6.392547	0.652059	0.408959
H	7.832380	-0.337287	0.610609
C	7.401975	0.180739	-1.437070
H	8.018752	-0.615348	-1.876508
H	6.539996	0.322450	-2.106842
H	7.993056	1.105893	-1.452994
C	-0.419795	-4.112679	-2.622800
F	-1.346638	-4.413949	-1.708773
F	-1.058214	-3.899511	-3.783524
F	0.352749	-5.189525	-2.799209
H	4.296994	3.396602	0.774142

Structure **L2_IrB3_IC-isoS**

C	-0.611619	0.684224	2.500525
C	1.406791	-0.454395	2.357868
C	1.658956	-0.264497	3.708253
C	0.730501	0.430068	4.476512
C	-0.421814	0.894785	3.867475
C	-1.809375	1.140775	1.774057
C	-2.889156	1.253493	-0.275387
C	-3.983623	1.902774	0.275060
C	-3.974039	2.170747	1.638701
C	-2.878511	1.784634	2.394205
H	2.086407	-1.017310	1.720509
H	0.903517	0.590830	5.538043
H	-1.166528	1.423147	4.455671
H	-2.836202	1.014696	-1.337251
H	-4.815643	2.671256	2.112800
H	-2.866198	1.973392	3.463968
N	0.312701	0.027945	1.766365
N	-1.829560	0.891508	0.451111
O	2.659311	0.160239	-1.955876
O	0.567545	2.482915	-2.170026
O	2.001552	-2.387104	-0.283209
B	0.354956	1.912210	-0.931131
B	1.903435	0.424949	-0.820599
B	1.039533	-1.736909	-1.051870
O	0.767443	-2.453566	-2.199267
O	2.711352	0.990870	0.168281
C	4.083594	0.869013	-0.239041
C	3.957079	0.754758	-1.787900
C	2.255472	-3.681370	-0.860895
C	1.718860	-3.518574	-2.321104
C	0.120837	3.848401	-2.107919
C	0.235342	4.176190	-0.589115
O	0.067712	2.885301	0.014193
C	3.921294	2.103103	-2.492792
H	3.610927	1.947047	-3.533595
H	3.180684	2.765811	-2.031845
H	4.902748	2.596136	-2.485489
C	5.006949	-0.122341	-2.440863
H	4.825273	-0.176185	-3.522216
H	6.011002	0.298191	-2.285288
H	4.997565	-1.143932	-2.043193
C	4.849518	2.080137	0.255789
H	5.894847	2.046418	-0.083590
H	4.402570	3.019782	-0.087232
H	4.849110	2.087224	1.353725
C	4.642054	-0.385981	0.419237
H	5.706209	-0.530214	0.188391
H	4.546579	-0.285941	1.508838
H	4.081665	-1.277142	0.109426
C	0.983771	4.699285	-3.017690
H	0.683825	5.755719	-2.960956
H	2.047058	4.626727	-2.764805
H	0.862239	4.366108	-4.056133
C	-1.316225	3.856894	-2.613780
H	-1.725170	4.874095	-2.678235
H	-1.338701	3.407958	-3.614348
H	-1.968765	3.260067	-1.961366
C	1.607234	4.688817	-0.177504
H	1.797236	5.704015	-0.551097
H	1.663167	4.704366	0.918006
H	2.402310	4.023007	-0.536154
C	-0.840010	5.102712	-0.054281
H	-1.840665	4.670140	-0.173398
H	-0.675850	5.277547	1.016884
H	-0.813546	6.076502	-0.564312
C	2.773559	-3.048815	-3.311570
H	2.278648	-2.820330	-4.263598
H	3.246664	-2.124436	-2.960727
H	3.542743	-3.811441	-3.493930
C	1.005759	-4.740250	-2.868520
H	1.683451	-5.604786	-2.917826
H	0.135948	-5.010329	-2.257786
H	0.650376	-4.529978	-3.885254
C	1.485468	-4.699235	-0.030208
H	0.403322	-4.521722	-0.064697
H	1.678807	-5.727497	-0.363559
H	1.803133	-4.611728	1.016517
C	3.738494	-3.983931	-0.762314
H	4.035773	-4.037912	0.293439
H	3.969854	-4.951537	-1.230404
H	4.348900	-3.212709	-1.246720
H	-0.494616	-0.203866	-1.932208
C	-3.734129	-1.625821	0.196560
O	-3.701945	-1.365025	1.398387
C	-5.055681	-1.635334	-0.559075
H	-4.983959	-0.983496	-1.447489

H	-5.244152	-2.644446	-0.963034
C	-1.307198	-1.895028	0.106795
H	-1.483059	-1.878269	1.187431
H	-0.829972	-2.855725	-0.128590
N	-2.617874	-1.911703	-0.531285
C	-2.655558	-2.198186	-1.951331
H	-1.681407	-2.599417	-2.250681
H	-3.430795	-2.935658	-2.195771
H	-2.830127	-1.295558	-2.559670
C	-6.222505	-1.206043	0.310940
H	-6.282254	-1.867131	1.188044
H	-6.020278	-0.203877	0.722650
C	-7.543490	-1.206127	-0.440468
H	-7.474851	-0.538499	-1.318251
H	-7.738233	-2.212846	-0.850699
C	-8.721770	-0.780540	0.422739
H	-8.788343	-1.448753	1.296419
H	-8.526699	0.225050	0.830436
C	-10.038195	-0.785611	-0.335866
H	-10.264397	-1.788184	-0.725144
H	-10.879853	-0.477155	0.296719
H	-10.000528	-0.103044	-1.196657
C	2.955400	-0.739715	4.283497
F	2.877815	-0.904923	5.607643
F	3.341685	-1.897992	3.744221
F	3.935307	0.146834	4.052279
H	-4.824687	2.181703	-0.355573
Ir	-0.074478	-0.125062	-0.390197

Structure **L2_IrB3_IC-isoS_TS-RE**

C	1.510393	-0.998212	1.868986
C	-0.557324	-0.227199	2.565781
C	-0.305419	-0.599721	3.879680
C	0.915824	-1.179968	4.189584
C	1.839051	-1.367898	3.171085
C	2.435948	-1.175270	0.730611
C	2.895460	-0.655597	-1.475824
C	4.048308	-1.431213	-1.478992
C	4.382984	-2.112219	-0.314346
C	3.570752	-1.980412	0.803684
H	-1.498088	0.237786	2.276585
H	1.146130	-1.469369	5.212077
H	2.817636	-1.781451	3.397918
H	2.565625	-0.117812	-2.365259
H	5.267874	-2.744669	-0.275605
H	3.809240	-2.519778	1.716552
N	0.316004	-0.441158	1.585727
N	2.109894	-0.534247	-0.405350
Ir	-0.082709	0.156554	-0.526250
O	-2.538606	-1.026539	0.641345
O	0.388376	-2.790279	-0.645501
O	-2.058319	2.514112	0.107743
B	-0.332981	-1.755966	-1.238612
B	-2.072168	-0.267230	-0.441184
B	-0.960508	2.139079	-0.695436
O	-0.967992	2.886994	-1.877511
O	-3.117185	-0.007465	-1.309975
C	-4.264514	-0.759273	-0.884866
C	-3.972391	-0.981841	0.626020
C	-2.627388	3.706362	-0.451818
C	-2.182612	3.637211	-1.947130
C	-0.000634	-4.022282	-1.268932
C	-0.530227	-3.532105	-2.645935
O	-1.036864	-2.225539	-2.332765
C	-4.388700	0.192534	1.504316
H	-3.998498	0.028210	2.519257
H	-3.965275	1.132755	1.125240
H	-5.480520	0.289839	1.576581
C	-4.511757	-2.273286	1.207216
H	-4.212638	-2.351131	2.260988
H	-5.610288	-2.293888	1.162692
H	-4.125286	-3.153556	0.681129
C	-5.523539	0.029553	-1.182052
H	-5.516608	1.013961	-0.701712
H	-5.619331	0.179197	-2.265642
H	-6.412944	-0.515858	-0.834319
C	-4.278806	-2.054643	-1.684434
H	-5.182118	-2.647503	-1.485675
H	-4.244335	-1.809105	-2.753620
H	-3.390799	-2.658253	-1.465082
C	1.209162	-4.934156	-1.334139
H	0.991569	-5.842481	-1.914298
H	2.070022	-4.424059	-1.783430
H	1.495038	-5.241844	-0.319593
C	-1.081330	-4.644660	-0.396363
H	-0.682390	-4.779061	0.616872
H	-1.955049	-3.983059	-0.320369
H	-1.404298	-5.624143	-0.774142
C	0.580253	-3.334644	-3.671427
H	0.171197	-2.789419	-4.530748
H	1.400521	-2.734682	-3.252667
H	0.989937	-4.289593	-4.027217
C	-1.637688	-4.372382	-3.248640
H	-1.973506	-3.916702	-4.189003
H	-1.278173	-5.387727	-3.470319
H	-2.506239	-4.448915	-2.585823
C	-3.151098	2.872401	-2.839253
H	-4.092682	3.420536	-2.985228
H	-2.679553	2.735595	-3.821022
H	-3.361978	1.877201	-2.430775
C	-1.881110	4.986591	-2.576278
H	-2.777569	5.623396	-2.589887
H	-1.082921	5.521686	-2.048280
H	-1.556965	4.839602	-3.614489
C	-2.043345	4.901920	0.296188
H	-2.200142	4.754574	1.372452
H	-0.965278	5.018946	0.123931
H	-2.530405	5.841345	0.001540
C	-4.128024	3.688351	-0.242667
H	-4.356910	3.716994	0.831336
H	-4.599201	4.563078	-0.713827
H	-4.574761	2.783551	-0.667492
H	-0.242263	0.466538	-2.086168
C	3.007256	2.172697	0.358508
O	2.923149	1.703818	1.492166
C	4.362098	2.279535	-0.326792
H	4.292011	1.885253	-1.354115
H	4.646560	3.339482	-0.436876
C	0.592719	2.370919	0.240235
H	0.740053	1.971744	1.245153
H	0.158912	3.365923	0.421775
N	1.910764	2.570674	-0.348962
C	1.956273	3.197513	-1.653181
H	1.289052	4.069343	-1.664269
H	2.967579	3.535058	-1.890993
H	1.596952	2.516998	-2.438311

C	5.443029	1.525919	0.428404
H	5.586115	1.978071	1.420623
H	5.095064	0.499262	0.624996
C	6.758706	1.479821	-0.330084
H	6.592267	1.046067	-1.333447
H	7.128963	2.504634	-0.509171
C	7.828915	0.672165	0.388331
H	7.988277	1.094279	1.393635
H	7.453879	-0.352668	0.551128
C	9.143735	0.629127	-0.371581
H	9.904840	0.039077	0.153917
H	9.009163	0.187351	-1.369115
H	9.548168	1.640681	-0.515843
C	-1.390886	-0.383714	4.886089
F	-1.795526	0.889097	4.900536
F	-2.466590	-1.128053	4.592584
F	-0.998645	-0.706567	6.121127
H	4.656518	-1.504847	-2.377143

Structure **L2_IrB3_PC-isoS**

C	-0.654961	-1.825181	-1.915755
C	-2.267777	-0.494761	-0.911665
C	-3.245516	-1.458893	-1.121505
C	-2.901774	-2.655270	-1.737616
C	-1.585086	-2.838368	-2.133459
C	0.739987	-1.910598	-2.391210
C	2.685455	-0.731002	-2.845110
C	3.290604	-1.844295	-3.411482
C	2.587775	-3.043069	-3.413655
C	1.299396	-3.082000	-2.897949
H	-2.495766	0.461712	-0.439706
H	-3.647174	-3.429176	-1.905800
H	-1.286589	-3.767653	-2.608543
H	3.193183	0.227281	-2.773587
H	3.040531	-3.950539	-3.808871
H	0.757886	-4.021440	-2.838238
N	-0.999455	-0.667240	-1.300291
N	1.448331	-0.761632	-2.346180
Ir	0.578465	0.802455	-1.007950
O	0.386486	2.339874	1.702148
O	-1.898511	2.480714	-0.467257
O	3.748166	-0.762709	-0.043025
B	-0.566177	2.442569	-0.918679
B	0.116153	1.242856	0.892334
B	3.271259	-1.005061	1.217335
O	3.957831	-0.337450	2.193778
O	-0.562882	0.263807	1.634107
C	-0.977861	0.839812	2.880972
C	0.032903	2.014863	3.054926
C	4.994885	-0.049349	0.117643
C	4.834303	0.598993	1.525499
C	-2.507515	3.663670	-1.002668
C	-1.278003	4.598185	-1.181636
O	-0.223447	3.668880	-1.465446
C	-0.545239	3.248950	3.720261
H	-1.404214	3.645009	3.166145
H	-0.866652	3.025848	4.748041
H	0.219017	4.035515	3.764871
C	1.313302	1.605229	3.769429
H	1.795652	0.742456	3.287677
H	2.020911	2.443101	3.723511
H	1.135682	1.366301	4.826583
C	-2.418731	1.296997	2.697177
H	-2.491772	2.052762	1.904236
H	-3.022298	0.431604	2.389498
H	-2.846258	1.700125	3.625171
C	-0.912879	-0.227867	3.955952
H	-1.173504	0.187041	4.940357
H	-1.633551	-1.024620	3.722858
H	0.083354	-0.681886	4.022858
C	-3.159952	3.262183	-2.320467
H	-3.721398	4.089362	-2.775083
H	-2.408730	2.910208	-3.040397
H	-3.859486	2.436569	-2.130355
C	-3.561370	4.160420	-0.034159
H	-3.146201	4.327818	0.966685
H	-4.006311	5.101488	-0.388091
H	-4.364937	3.416419	0.052524
C	-1.395586	5.583329	-2.327040
H	-2.252413	6.256487	-2.177412
H	-0.487522	6.197016	-2.380684
H	-1.513101	5.073394	-3.289589
C	-0.895100	5.325864	0.100387
H	-1.623688	6.103489	0.368248
H	-0.791771	4.618485	0.934279
H	0.081300	5.803122	-0.048799
C	6.120199	0.710164	2.318646
H	6.579847	-0.268090	2.496957
H	6.844138	1.349014	1.793288
H	5.912186	1.167938	3.293489
C	4.120272	1.940765	1.491070
H	4.747422	2.718322	1.035742
H	3.167620	1.891658	0.943566
H	3.900135	2.245289	2.521626
C	5.162316	0.947437	-1.009998
H	6.037374	1.587370	-0.828468
H	5.335478	0.417326	-1.957267
H	4.275571	1.584100	-1.118486
C	6.091254	-1.103858	0.054132
H	7.093747	-0.657367	0.083821
H	6.004735	-1.824043	0.879254
H	5.987853	-1.658443	-0.888706
H	1.825323	1.827511	-0.882264
C	0.564483	-3.623930	0.535475
O	0.363762	-4.546656	-0.252274
C	-0.545760	-3.070021	1.404720
H	-0.190884	-2.974588	2.444251
H	-0.768480	-2.033082	1.098281
C	2.066950	-1.944973	1.591881
H	1.185593	-1.286288	1.652352
H	2.233736	-2.325045	2.618280
N	1.798818	-3.038409	0.664985
C	2.947350	-3.719695	0.096159
H	2.606729	-4.655704	-0.353390
H	3.444858	-3.104460	-0.664585
H	3.677917	-3.942607	0.890545
C	-1.809299	-3.909751	1.365662
H	-2.090514	-4.100098	0.319415
H	-1.615170	-4.904329	1.797564
C	-2.960195	-3.235500	2.094321
H	-2.703292	-3.101039	3.160804
H	-3.098942	-2.212914	1.693991
C	-4.268844	-4.002562	1.982144
H	-4.522764	-4.122274	0.915348
H	-4.125150	-5.024023	2.370700
C	-5.415276	-3.321495	2.709852

H	-5.191635	-3.209383	3.780132
H	-5.592756	-2.316417	2.304035
H	-6.351232	-3.887246	2.622614
C	-4.653884	-1.132198	-0.732642
F	-5.167217	-0.193767	-1.537760
F	-5.452327	-2.201397	-0.799208
F	-4.723733	-0.648347	0.514236
H	4.297855	-1.771714	-3.815116

Structure **L2_IrB2H**

C	2.765732	-0.488988	-1.118500
C	4.040033	-0.529765	-0.566141
C	4.277912	-1.368756	0.516237
C	3.229821	-2.127485	1.014701
C	0.859939	-3.795938	1.819033
C	-0.306609	-4.367979	2.307297
C	-1.528633	-3.832242	1.921086
C	-1.542690	-2.748832	1.052489
H	-2.470075	-2.290413	0.703874
C	0.777223	-2.704298	0.957927
C	1.970993	-2.018965	0.423821
C	-4.635482	-0.143223	-0.707826
C	-4.159393	1.249539	-0.211618
C	-5.149823	-0.118114	-2.140640
H	-4.446160	0.403199	-2.801702
H	-6.131261	0.370033	-2.214948
H	-5.250458	-1.150351	-2.499230
C	-5.639917	-0.838133	0.190014
H	-5.916683	-1.808936	-0.242001
H	-6.557136	-0.239813	0.290379
H	-5.230978	-1.016746	1.191564
C	-4.965453	2.426228	-0.723245
H	-4.541057	3.359309	-0.329240
H	-6.012639	2.362210	-0.393187
H	-4.943338	2.485250	-1.816863
C	-4.043826	1.319436	1.308518
H	-3.461844	0.474442	1.701533
H	-5.029006	1.335101	1.794952
H	-3.504921	2.235077	1.584421
B	-2.341746	0.021609	-0.821202
N	1.757174	-1.216233	-0.637927
N	-0.417454	-2.197394	0.573966
O	-3.408633	-0.890791	-0.703382
O	-2.836366	1.320061	-0.747458
Ir	-0.433243	-0.587357	-0.926140
B	0.040785	1.010610	0.144013
O	1.196663	1.750067	-0.101362
O	-0.602959	1.463096	1.283413
C	1.433869	2.558255	1.060377
C	-0.002498	2.720719	1.632293
C	2.352514	1.761309	1.980088
C	2.117926	3.840250	0.632798
C	-0.081839	2.902544	3.133682
C	-0.793796	3.815294	0.927525
H	1.871494	0.828042	2.306363
H	2.643982	2.334080	2.870933
H	3.264620	1.503077	1.425487
H	3.106749	3.604104	0.217653
H	2.257495	4.516994	1.488179
H	1.544700	4.363143	-0.140230
H	-1.133168	2.990640	3.438252
H	0.440630	3.818357	3.445997
H	0.351948	2.050381	3.668775
H	-0.741726	3.703666	-0.162752
H	-0.437655	4.817379	1.203134
H	-1.850161	3.732708	1.214484
H	2.528833	0.162636	-1.956846
H	-0.343804	0.478567	-2.133761
H	5.264851	-1.413974	0.971106
H	3.381916	-2.761628	1.884497
H	1.828886	-4.208514	2.090234
H	-0.259064	-5.223963	2.977252
H	-2.467305	-4.245783	2.280892
C	5.088751	0.414106	-1.063308
F	4.998516	0.606781	-2.379392
F	4.964741	1.613909	-0.476414
F	6.320146	-0.031434	-0.793311

Structure **L4_IrB5**

Ir	0.060043	0.044244	-0.012805
B	-0.347921	2.113447	-0.390742
C	2.298067	-0.369896	-2.251084
C	2.836883	-0.578023	-3.513052
C	0.611438	-0.655787	-4.371890
C	0.137820	-0.432021	-3.078029
C	-1.297403	-0.340564	-2.753546
C	-2.294597	-0.404455	-3.726802
C	-3.931700	-0.143341	-2.016935
C	-2.898904	-0.094849	-1.090424
B	-0.382925	-2.126253	0.033867
N	0.986566	-0.292950	-2.042078
N	-1.617458	-0.186499	-1.447347
O	-1.171145	-2.803649	0.935601
O	-0.253829	-2.838071	-1.146090
O	-0.571507	1.126417	2.823659
O	-2.307225	0.042793	1.826321
O	2.314835	-1.965615	0.840272
O	0.940654	-1.446661	2.583508
C	1.976669	-0.723336	-4.594889
C	-3.628279	-0.303745	-3.360870
C	-0.864397	-4.128145	-0.970284
C	-1.814315	-3.889236	0.244247
C	1.829241	-2.441304	3.094126
C	2.963157	-2.491933	2.015079
C	-1.709634	1.322344	3.665463
C	-2.661748	0.179766	3.210829
C	-1.453402	3.715241	-1.584376
B	1.182387	-1.253986	1.239902
H	2.933255	-0.260683	-1.374177
B	-0.971208	0.444052	1.695737
C	-0.858794	4.342350	-0.296442
O	-0.529967	3.175051	0.479493
O	-0.712843	2.488708	-1.682059
C	0.434047	5.099441	-0.561866
H	0.887858	5.377594	0.398773
H	1.149378	4.470770	-1.108856
H	0.257193	6.022343	-1.130159
C	-1.811430	5.209740	0.500511
H	-1.308134	5.568862	1.407918
H	-2.124123	6.086283	-0.085449
H	-2.706586	4.659922	0.812197
C	-2.923003	3.337278	-1.437988
H	-3.578547	4.218499	-1.431461
H	-3.209647	2.704331	-2.289809
H	-3.103484	2.762424	-0.516955
C	-1.251056	4.521112	-2.850761

H	-1.705508	3.994623	-3.700381
H	-1.729247	5.507902	-2.769515
H	-0.187792	4.662183	-3.072385
C	-1.263684	1.240144	5.111832
H	-0.606292	2.088748	5.342462
H	-2.124134	1.279363	5.795994
H	-0.699592	0.319671	5.299921
C	-2.257177	2.711051	3.363868
H	-1.442279	3.437489	3.480460
H	-2.603007	2.776621	2.323562
H	-3.080769	2.987208	4.036973
C	-2.348948	-1.154489	3.876857
H	-2.657302	-1.168688	4.931423
H	-2.887217	-1.948287	3.341822
H	-1.276233	-1.378230	3.803952
C	-4.141926	0.486526	3.308586
H	-4.721112	-0.373513	2.945195
H	-4.437184	0.678036	4.350274
H	-4.417108	1.356982	2.701193
C	2.277720	-2.001133	4.475319
H	1.410198	-1.994211	5.148981
H	3.027792	-2.688934	4.892057
H	2.689400	-0.985542	4.453296
C	4.131264	-1.575439	2.336582
H	4.703880	-1.930585	3.203887
H	4.807834	-1.545215	1.471270
H	3.770012	-0.561217	2.533608
C	3.491239	-3.879499	1.704965
H	4.259245	-3.813238	0.923776
H	3.952762	-4.325588	2.597968
H	2.706705	-4.554688	1.346704
C	1.041926	-3.739887	3.203207
H	0.141632	-3.561383	3.806416
H	0.703789	-4.077634	2.217355
H	1.630889	-4.537752	3.675725
C	-1.960765	-5.064236	1.191031
H	-2.602526	-4.775206	2.033564
H	-2.431649	-5.916915	0.680731
H	-0.999960	-5.393043	1.600593
C	0.257388	-5.114139	-0.682908
H	-0.112957	-6.143821	-0.589475
H	0.980236	-5.077063	-1.507192
H	0.794183	-4.846734	0.236491
C	-1.563164	-4.510096	-2.261089
H	-0.818240	-4.653130	-3.054673
H	-2.120132	-5.450968	-2.145256
H	-2.259946	-3.729626	-2.590144
C	-3.196991	-3.392572	-0.156563
H	-3.818846	-4.196516	-0.573154
H	-3.697234	-2.985986	0.732551
H	-3.138202	-2.585724	-0.897850
B	1.669034	1.142628	0.719102
O	2.376873	1.966214	-0.168895
O	2.253747	1.196090	1.971526
C	3.555512	2.440823	0.506713
C	3.132643	2.331872	1.996375
C	4.689608	1.498337	0.122571
C	3.883280	3.844397	0.041817
C	4.272018	2.088429	2.966247
C	2.292761	3.506000	2.487501
H	4.484355	0.472489	0.459018
H	5.654599	1.816883	0.539275
H	4.782163	1.485484	-0.972350
H	4.114617	3.837355	-1.031582
H	4.764309	4.228986	0.575789
H	3.050878	4.535692	0.206207
H	3.865161	1.917594	3.971801
H	4.927586	2.970035	3.011175
H	4.884811	1.222199	2.695651
H	1.448166	3.699946	1.813251
H	2.889319	4.421444	2.605710
H	1.866633	3.232254	3.461527
H	-3.088868	0.002188	-0.017649
H	2.370901	-0.894785	-5.593697
H	-0.073628	-0.780456	-5.205677
H	-4.417835	-0.346909	-4.107182
H	-2.035133	-0.524394	-4.774599
C	-5.330541	-0.028191	-1.500024
F	-5.506631	1.125364	-0.846766
F	-5.604451	-1.012855	-0.637310
F	-6.232011	-0.083411	-2.484258
C	4.324701	-0.576557	-3.673514
F	4.807780	0.673258	-3.655756
F	4.925356	-1.240875	-2.684059
F	4.697388	-1.131243	-4.831102

Structure **L4_IrB3**

C	-2.996726	1.749732	-0.282840
H	-3.361487	0.723128	-0.182343
C	-3.855766	2.842934	-0.341076
C	-3.317413	4.116865	-0.457416
H	-3.968533	4.985682	-0.515859
C	-1.937522	4.259906	-0.492148
C	1.062171	4.384333	-0.285471
C	2.448508	4.343913	-0.232754
H	3.029554	5.258910	-0.148415
C	3.082622	3.108878	-0.273207
C	2.310850	1.959080	-0.376532
H	2.757921	0.962245	-0.378741
C	0.348230	3.191794	-0.399037
C	-1.129985	3.127201	-0.416236
C	-3.295628	-2.669747	0.441540
C	-2.427841	-3.555070	-0.496982
C	-3.023578	-2.932287	1.917159
H	-1.944892	-2.909089	2.121732
H	-3.434512	-3.897567	2.243613
H	-3.497734	-2.137938	2.508959
C	-4.785909	-2.713733	0.171130
H	-5.310639	-2.053309	0.874182
H	-5.177846	-3.731960	0.308629
H	-5.025986	-2.382909	-0.845529
C	-2.120856	-4.943141	0.029875
H	-1.498369	-5.479993	-0.698568
H	-3.042294	-5.523381	0.184532
H	-1.566844	-4.898529	0.974089
C	-2.987208	-3.649116	-1.911255
H	-3.233638	-2.654602	-2.305296
H	-3.884820	-4.280807	-1.959887
H	-2.219813	-4.085162	-2.563181
C	2.142988	-2.756270	-2.419766
C	3.176127	-2.219350	-1.389102
C	1.520600	-4.082004	-1.997581
H	1.139219	-4.031145	-0.969567
H	2.231063	-4.915537	-2.085856
H	0.661347	-4.283692	-2.650256
C	2.657643	-2.852111	-3.841785

H	1.863502	-3.233251	-4.496238
H	3.510202	-3.544152	-3.904416
H	2.970112	-1.875005	-4.226998
C	3.972185	-3.287105	-0.665097
H	4.684719	-2.817176	0.026402
H	4.545705	-3.899736	-1.375736
H	3.318155	-3.945609	-0.081990
C	4.121007	-1.172288	-1.966152
H	3.567716	-0.396621	-2.514180
H	4.866221	-1.611866	-2.642811
H	4.657423	-0.688082	-1.136784
B	-1.474163	-1.480869	-0.299121
B	1.137717	-1.200040	-1.088868
N	-1.671080	1.890429	-0.326679
N	0.982071	2.003549	-0.457108
O	-2.799617	-1.352117	0.149196
O	-1.207258	-2.814411	-0.572658
O	1.108274	-1.765710	-2.358289
O	2.333158	-1.542562	-0.446200
Ir	-0.241185	0.120417	-0.459369
B	0.502729	-0.482999	1.285027
O	0.259818	-1.653970	1.983578
O	1.352206	0.355528	2.003187
C	0.799690	-1.496390	3.304496
C	1.893065	-0.405497	3.095373
C	-0.338585	-1.030895	4.203908
C	1.317854	-2.839538	3.777098
C	2.105826	0.518643	4.278142
C	3.230801	-0.971095	2.636664
H	-0.735060	-0.062885	3.870208
H	-0.023491	-0.940064	5.252221
H	-1.154814	-1.762782	4.154159
H	0.478963	-3.540350	3.883969
H	1.812767	-2.749656	4.754967
H	2.025773	-3.269990	3.060249
H	2.888798	1.249474	4.037155
H	2.433333	-0.047210	5.162296
H	1.194847	1.071128	4.533558
H	3.098983	-1.661537	1.796144
H	3.758682	-1.481439	3.454029
H	3.857732	-0.138852	2.286598
H	-1.499982	5.250257	-0.586127
H	0.548289	5.339956	-0.221764
C	-5.333532	2.596135	-0.309123
F	-5.671259	1.836033	0.734388
F	-5.734887	1.956387	-1.410972
F	-6.021880	3.738643	-0.227998
C	4.572135	2.958094	-0.230894
F	5.024717	2.403964	-1.360593
F	4.949771	2.161794	0.773144
F	5.186811	4.133777	-0.076917

Structure **L4_IrB**

C	1.017102	-1.999918	-0.017350
C	-1.311717	-1.710369	-0.014968
C	-1.501559	-3.072543	-0.024534
C	-0.409938	-3.944989	-0.032826
C	0.849030	-3.385032	-0.029637
C	2.327433	-1.361757	-0.007330
C	3.434335	0.670463	0.029884
C	4.676801	0.050942	0.016853
C	4.729943	-1.338778	-0.010067
C	3.541650	-2.050800	-0.021408
H	-2.148377	-1.016349	-0.014709
H	-0.556445	-5.022530	-0.040035
H	1.726539	-4.026148	-0.034244
H	3.351740	1.757821	0.053298
H	5.687804	-1.852532	-0.019420
H	3.566426	-3.137000	-0.041694
N	-0.068852	-1.147835	-0.010365
N	2.289053	-0.009510	0.018588
Ir	0.188775	0.762936	0.021267
O	-2.941044	0.775190	-0.171216
B	-1.723670	1.435075	0.008597
O	-1.954946	2.802047	0.180255
C	-4.000987	1.692509	0.140076
C	-3.330213	3.067590	-0.138586
C	-3.385254	3.468460	-1.607035
H	-2.717958	4.325253	-1.763036
H	-3.041604	2.651007	-2.253979
H	-4.398436	3.760114	-1.915353
C	-3.831042	4.206572	0.726379
H	-3.303432	5.131874	0.461976
H	-4.906723	4.373157	0.569458
H	-3.657541	4.012960	1.790417
C	-4.356373	1.487751	1.607061
H	-3.505859	1.728116	2.257950
H	-5.216510	2.099454	1.910624
H	-4.614245	0.432297	1.762459
C	-5.199719	1.373323	-0.730138
H	-6.003569	2.107125	-0.573883
H	-4.937282	1.361347	-1.793425
H	-5.591081	0.381326	-0.469196
C	-2.885303	-3.647023	-0.030094
F	-3.082722	-4.389020	-1.126760
F	-3.062242	-4.457915	1.021007
F	-3.831373	-2.711999	0.009049
C	5.907338	0.903382	0.005940
F	6.004314	1.591709	-1.136326
F	7.017104	0.173319	0.137479
F	5.882314	1.797919	0.998776

Structure **L4_IrB3_RC**

C	-1.893083	1.373179	0.982745
C	-2.450022	-0.747169	0.243534
C	-3.676542	-0.696597	0.898264
C	-3.995545	0.425583	1.651347
C	-3.084650	1.471919	1.698415
C	-0.899119	2.463180	0.931189
C	1.271309	3.055314	0.377423
C	1.029922	4.385622	0.679996
C	-0.237140	4.756118	1.122859
C	-1.213807	3.784633	1.247899
H	-2.132152	-1.624844	-0.328633
H	-4.940860	0.482787	2.187019
H	-3.300686	2.356387	2.292989
H	2.237143	2.713591	0.002311
H	-0.451230	5.798380	1.353807
H	-2.223203	4.057387	1.545142
N	-1.583689	0.264193	0.279693
N	0.330007	2.117952	0.510671
Ir	0.653149	-0.007913	-0.179570
O	1.278637	-2.357190	1.686483
O	3.572971	-0.703257	0.532012
O	1.865714	-2.542084	-1.468140
B	2.655765	-0.060776	-0.287196

B	0.774070	-1.083790	1.485153
B	0.754879	-1.851807	-0.999519
O	-0.403807	-2.611013	-1.258293
O	0.207300	-0.584839	2.659637
C	0.125619	-1.683893	3.582062
C	1.289728	-2.592777	3.101810
C	1.426008	-3.673353	-2.224384
C	0.006521	-3.933606	-1.643697
C	4.871861	-0.172459	0.254253
C	4.711649	0.361958	-1.196869
O	3.326508	0.744335	-1.221951
C	2.651763	-2.145099	3.617255
H	2.780041	-2.368753	4.685251
H	3.427574	-2.673904	3.049602
H	2.803606	-1.070142	3.453434
C	1.093119	-4.072009	3.363083
H	0.191698	-4.456666	2.872058
H	1.953722	-4.632818	2.975588
H	1.016898	-4.271261	4.441811
C	0.261424	-1.162977	4.996976
H	-0.601451	-0.532063	5.247412
H	0.294539	-1.992834	5.717593
H	1.167588	-0.559075	5.116111
C	-1.243112	-2.323574	3.379508
H	-1.336855	-2.741203	2.366003
H	-1.442042	-3.122606	4.106469
H	-2.013240	-1.548683	3.500325
C	5.134008	0.932359	1.271586
H	5.008087	0.515294	2.279270
H	6.150910	1.339806	1.189953
H	4.418055	1.758404	1.158598
C	5.898138	-1.275980	0.419374
H	5.942034	-1.582584	1.472886
H	5.633717	-2.157160	-0.175002
H	6.900012	-0.932914	0.122399
C	5.564794	1.568101	-1.532835
H	5.390587	1.868551	-2.574142
H	5.324958	2.425030	-0.891975
H	6.634061	1.336070	-1.422080
C	4.892639	-0.724623	-2.249940
H	4.268692	-1.599075	-2.019178
H	4.569476	-0.327520	-3.221206
H	5.941228	-1.040324	-2.339229
C	-0.995406	-4.498274	-2.630517
H	-1.966100	-4.637463	-2.136495
H	-1.145470	-3.831759	-3.488067
H	-0.665178	-5.477833	-3.005461
C	0.025729	-4.777005	-0.375163
H	0.253997	-5.831008	-0.584753
H	0.754241	-4.381870	0.344590
H	-0.968920	-4.727578	0.090971
C	2.411927	-4.808522	-2.032103
H	2.569649	-5.025644	-0.970373
H	2.065295	-5.723050	-2.535137
H	3.381776	-4.529604	-2.465386
C	1.409522	-3.245684	-3.687240
H	2.408518	-2.875485	-3.952503
H	1.154719	-4.073681	-4.362697
H	0.696857	-2.425265	-3.849801
H	0.806045	0.633956	-2.437509
C	-2.055856	1.828124	-2.449461
O	-1.743347	2.847264	-1.841485
C	-3.491139	1.327546	-2.462704
H	-3.856006	1.266752	-3.500639
H	-3.508687	0.288707	-2.088941
C	0.265126	1.407149	-3.025820
H	0.739500	1.429803	-4.016684
H	0.373248	2.383309	-2.548714
N	-1.141721	1.083817	-3.144393
C	-1.423349	-0.222120	-3.707706
H	-1.250501	-1.028235	-2.972800
H	-2.450109	-0.286979	-4.077568
H	-0.754535	-0.387217	-4.562802
C	-4.422691	2.187202	-1.624378
H	-3.915150	2.454103	-0.684840
H	-4.607891	3.146217	-2.132058
C	-5.735520	1.486420	-1.314272
H	-6.278581	1.256476	-2.247247
H	-5.523353	0.505285	-0.849954
C	-6.635209	2.283985	-0.383046
H	-6.062272	2.553532	0.521512
H	-6.904635	3.240215	-0.859460
C	-7.887148	1.517866	0.009335
H	-8.484138	1.257464	-0.875610
H	-7.625872	0.575505	0.512404
H	-8.530023	2.093394	0.686738
C	-4.598653	-1.866511	0.761040
F	-4.810228	-2.152468	-0.530173
F	-4.086174	-2.963740	1.325714
F	-5.787917	-1.631779	1.324457
C	2.092953	5.427381	0.525176
F	2.239890	6.127174	1.657155
F	3.275786	4.894925	0.215337
F	1.771535	6.303217	-0.432319

Structure **L4_IrB3_TS-OA**

C	1.536541	-1.908199	0.951755
C	2.582458	0.150481	0.829485
C	3.759886	-0.365172	1.353072
C	3.808616	-1.711464	1.698940
C	2.681921	-2.489534	1.495702
C	0.300178	-2.666358	0.696650
C	-1.816037	-2.646176	-0.250025
C	-2.111346	-3.889944	0.276935
C	-1.148151	-4.550947	1.035079
C	0.070400	-3.932294	1.241564
H	2.476543	1.206200	0.581162
H	4.722641	-2.147206	2.097477
H	2.710543	-3.551260	1.723533
H	-2.554226	-2.069854	-0.806490
H	-1.363035	-5.526007	1.469508
H	0.825636	-4.414051	1.856166
N	1.500801	-0.600654	0.629379
N	-0.638279	-2.046604	-0.045330
Ir	-0.354030	0.120148	-0.467399
O	-1.788769	-0.663212	2.128758
O	-3.303066	-0.102817	-1.097711
O	0.658375	2.548861	0.994741
B	-2.092252	0.531234	-1.418126
B	-1.594434	0.359288	1.204592
B	-0.187492	2.124578	-0.033058
O	-0.744562	3.212417	-0.675591
O	-2.404696	1.441912	1.514019
C	-3.033603	1.193099	2.780934
C	-2.977231	-0.358573	2.878968
C	0.833988	3.968750	0.864265

C	-0.447196	4.390656	0.085227
C	-4.259880	0.227205	-2.117090
C	-3.693147	1.570914	-2.647026
O	-2.279873	1.374268	-2.500254
C	-2.828120	-0.908400	4.282983
H	-2.783440	-2.004664	4.244835
H	-1.911965	-0.550519	4.765289
H	-3.688116	-0.627006	4.907840
C	-4.135815	-1.049793	2.169962
H	-4.230928	-0.695683	1.133886
H	-3.937280	-2.130547	2.140711
H	-5.088964	-0.897919	2.694417
C	-2.193443	1.883443	3.848184
H	-1.164736	1.498785	3.848815
H	-2.150212	2.959371	3.633810
H	-2.621005	1.758780	4.852101
C	-4.426838	1.787142	2.745168
H	-4.975890	1.570547	3.673029
H	-4.358462	2.878049	2.635497
H	-5.003517	1.400369	1.896784
C	-4.218929	-0.888757	-3.153393
H	-3.227964	-0.954398	-3.623104
H	-4.425716	-1.845711	-2.655411
H	-4.970736	-0.746883	-3.941416
C	-5.635247	0.301194	-1.487213
H	-6.387298	0.632648	-2.217634
H	-5.927958	-0.694365	-1.125282
H	-5.649082	0.988813	-0.633414
C	-4.008846	1.871616	-4.097052
H	-5.095063	1.935111	-4.256508
H	-3.567999	2.836407	-4.378108
H	-3.599974	1.106855	-4.766847
C	-4.068230	2.756504	-1.764660
H	-3.436287	3.608604	-2.046427
H	-5.122593	3.041645	-1.886060
H	-3.873403	2.537674	-0.704398
C	-1.639169	4.658202	0.993621
H	-1.800497	3.815103	1.674674
H	-1.515021	5.583903	1.571994
H	-2.539307	4.753949	0.372126
C	-0.260096	5.554820	-0.868121
H	-1.209869	5.767217	-1.375623
H	0.044889	6.461293	-0.325193
H	0.488061	5.337600	-1.638260
C	2.114235	4.188224	0.067265
H	2.373395	5.252193	-0.016256
H	2.946750	3.674979	0.569907
H	2.019284	3.776338	-0.947262
C	0.976843	4.575530	2.245029
H	1.023819	5.672622	2.188722
H	0.141490	4.292280	2.894678
H	1.904078	4.220237	2.713260
H	0.250613	0.997029	-1.706281
C	3.065661	-1.027824	-2.163479
O	2.732882	-2.148709	-1.790246
C	4.522843	-0.595754	-2.175969
H	4.836370	-0.378714	-3.210885
H	4.623714	0.360315	-1.636325
C	0.739548	-0.379241	-2.478449
H	0.224566	-0.027591	-3.380550
H	0.619023	-1.461950	-2.399477
N	2.153117	-0.095477	-2.581245
C	2.515237	1.271423	-2.887157
H	2.635497	1.883283	-1.975364
H	3.442844	1.325502	-3.465350
H	1.719569	1.722475	-3.491264
C	5.453467	-1.621071	-1.554856
H	5.066912	-1.901399	-0.562461
H	5.440115	-2.549471	-2.144817
C	6.870606	-1.092589	-1.405975
H	7.292314	-0.846282	-2.396073
H	6.844611	-0.140338	-0.847861
C	7.794019	-2.063134	-0.685664
H	7.324834	-2.363139	0.266593
H	7.888251	-2.987791	-1.277596
C	9.164785	-1.468222	-0.413214
H	9.831416	-2.178949	0.090842
H	9.654090	-1.156200	-1.346603
H	9.079729	-0.577871	0.226231
C	4.927713	0.546842	1.559602
F	5.020743	1.451552	0.572521
F	4.824444	1.229636	2.702682
F	6.081268	-0.130271	1.598631
C	-3.459292	-4.509056	0.086025
F	-3.373059	-5.669104	-0.574416
F	-4.023890	-4.780244	1.271216
F	-4.291905	-3.712806	-0.587123

Structure **L4_IrB3_IC**

C	-1.462887	-1.700067	-1.303757
C	-2.632489	0.242385	-0.863874
C	-3.773476	-0.255162	-1.480991
C	-3.737439	-1.526878	-2.039063
C	-2.565215	-2.256462	-1.951958
C	-0.188795	-2.417742	-1.141082
C	1.939360	-2.416466	-0.199733
C	2.255504	-3.616680	-0.812029
C	1.305795	-4.244093	-1.612806
C	0.073073	-3.638225	-1.768308
H	-2.598281	1.244932	-0.439013
H	-4.620904	-1.945915	-2.515178
H	-2.529706	-3.264501	-2.354191
H	2.639897	-1.897240	0.453570
H	1.531608	-5.188260	-2.103793
H	-0.681624	-4.106214	-2.393101
N	-1.508109	-0.464503	-0.775144
N	0.747064	-1.824982	-0.362953
Ir	0.304584	0.108327	0.511269
O	2.987718	0.528554	-0.908345
O	2.686510	-0.734951	2.125869
O	1.305987	3.056993	-0.071623
B	1.890604	0.345790	1.744530
B	1.623025	0.639000	-1.085451
B	0.311329	2.094909	-0.126022
O	-0.880339	2.651789	-0.591970
O	1.305139	0.742920	-2.429171
C	2.505172	0.494227	-3.189713
C	3.630483	0.817341	-2.161845
C	0.667836	4.337647	-0.197562
C	-0.595772	3.989936	-1.033067
C	3.847107	-0.208367	2.790587
C	3.302464	1.135769	3.351664
O	2.310913	1.498392	2.380934
C	4.872412	-0.044024	-2.277461
H	5.334337	0.060886	-3.269755
H	5.610082	0.271410	-1.527917

H	4.652782	-1.104183	-2.102336
C	4.012519	2.289713	-2.130482
H	3.123495	2.924351	-2.026543
H	4.644262	2.469751	-1.251058
H	4.571024	2.588184	-3.027864
C	2.473168	-0.971929	-3.601304
H	2.525536	-1.638445	-2.729712
H	1.529707	-1.170168	-4.126600
H	3.304153	-1.225958	-4.272817
C	2.495990	1.370548	-4.425690
H	2.375014	2.430059	-4.173800
H	3.429523	1.252426	-4.994478
H	1.662024	1.081795	-5.078136
C	4.309406	-1.205553	3.832960
H	3.500656	-1.482233	4.517971
H	4.657932	-2.120724	3.336095
H	5.145626	-0.799116	4.419959
C	4.927241	-0.025543	1.731302
H	5.881973	0.291255	2.172729
H	5.085235	-0.988718	1.224071
H	4.616667	0.701421	0.970254
C	2.581334	0.973566	4.683891
H	2.045075	1.904508	4.907354
H	1.842866	0.161390	4.637132
H	3.276692	0.768385	5.509128
C	4.327987	2.247002	3.448093
H	5.151408	1.964693	4.120125
H	4.742791	2.495138	2.465178
H	3.855375	3.151815	3.851644
C	-1.801502	4.865090	-0.757508
H	-1.585346	5.915006	-1.001934
H	-2.646436	4.541438	-1.379368
H	-2.112788	4.807672	0.291469
C	-0.327059	3.923589	-2.530051
H	0.535232	3.279464	-2.747663
H	-1.200697	3.480467	-3.024450
H	-0.155713	4.919245	-2.960901
C	1.623712	5.310052	-0.856117
H	1.973026	4.946206	-1.829299
H	1.142853	6.287648	-1.004110
H	2.501901	5.455280	-0.214133
C	0.345315	4.800720	1.217748
H	-0.368073	4.122379	1.705804
H	1.270580	4.784877	1.806947
H	-0.068392	5.818029	1.235689
H	-0.153234	1.392611	1.398462
C	-3.043472	-1.341797	1.880211
O	-2.865352	-2.364121	1.218143
C	-4.449452	-0.810952	2.125334
H	-4.657086	-0.791127	3.208367
H	-4.510564	0.240938	1.798933
C	-0.633960	-1.022787	2.140674
H	-0.069981	-0.921707	3.079181
H	-0.649044	-2.084972	1.874389
N	-2.012116	-0.631150	2.418948
C	-2.194505	0.641823	3.078017
H	-3.107721	0.658372	3.681140
H	-1.343613	0.815522	3.748830
H	-2.225733	1.483997	2.363216
C	-5.514130	-1.627589	1.415983
H	-5.277200	-1.678678	0.341167
H	-5.473004	-2.669397	1.767292
C	-6.910287	-1.057389	1.604282

H	-7.150312	-1.001076	2.680832
H	-6.935864	-0.016729	1.236090
C	-7.983314	-1.864791	0.889343
H	-7.745016	-1.903604	-0.186306
H	-7.949443	-2.907920	1.243604
C	-9.376863	-1.293349	1.088986
H	-9.644885	-1.270871	2.154703
H	-9.435673	-0.261252	0.715852
H	-10.142160	-1.880459	0.566037
C	-4.994992	0.605381	-1.565639
F	-5.179573	1.305645	-0.439302
F	-4.900838	1.490146	-2.563845
F	-6.097368	-0.121475	-1.778139
C	3.646900	-4.155535	-0.704609
F	3.657927	-5.491389	-0.720086
F	4.388036	-3.745411	-1.746829
F	4.261363	-3.746890	0.405427

Structure **L4_IrB3_TS-RE**

C	0.006619	2.641010	0.359433
C	-1.899509	2.864627	-0.907400
C	-2.218432	4.070456	-0.297768
C	-1.376502	4.568691	0.691904
C	-0.245251	3.846270	1.023805
C	1.174158	1.807534	0.691024
C	2.157782	-0.298091	0.675341
C	3.266286	0.168991	1.365234
C	3.334891	1.511840	1.716161
C	2.270885	2.329988	1.377154
H	-2.549302	2.421011	-1.656836
H	-1.616673	5.500842	1.200902
H	0.409574	4.197834	1.816948
H	2.058534	-1.348414	0.396903
H	4.205523	1.910049	2.234190
H	2.306127	3.389229	1.616136
N	-0.820337	2.157615	-0.575572
N	1.139560	0.504071	0.323203
Ir	-0.486504	-0.320400	-0.828046
O	-0.547829	-1.818666	1.783564
O	1.308259	-2.782911	-0.684994
O	-3.252438	-1.529337	0.035555
B	0.319294	-2.123019	-1.445927
B	-1.039770	-0.715056	1.115432
B	-2.542275	-0.530392	-0.619306
O	-3.409719	0.436216	-1.113967
O	-1.611352	0.185922	2.004593
C	-1.311994	-0.252449	3.341364
C	-1.008767	-1.769818	3.143723
C	-4.654904	-1.290602	-0.185346
C	-4.688932	0.234253	-0.492719
C	1.105584	-4.199190	-0.770951
C	-0.308243	-4.312246	-1.433768
O	-0.409496	-3.092383	-2.166187
C	0.082263	-2.321067	4.041500
H	1.046579	-1.831289	3.857538
H	-0.180742	-2.199639	5.102283
H	0.212886	-3.393531	3.848119
C	-2.247164	-2.650455	3.216980
H	-3.024015	-2.287512	2.531405
H	-1.973483	-3.664624	2.896063
H	-2.654451	-2.708924	4.235263
C	-0.100056	0.547030	3.803097
H	0.152319	0.344887	4.852410

H	0.782452	0.324920	3.187593
H	-0.323628	1.617972	3.700470
C	-2.497492	0.051565	4.234508
H	-2.648382	1.137457	4.293079
H	-3.419962	-0.396576	3.847100
H	-2.329096	-0.324732	5.253792
C	2.216939	-4.780876	-1.633127
H	2.161266	-4.408010	-2.664265
H	3.188457	-4.488367	-1.212338
H	2.175273	-5.878082	-1.662568
C	1.198864	-4.770194	0.632600
H	2.219646	-4.631363	1.015671
H	0.510315	-4.251677	1.310045
H	0.975169	-5.846883	0.640625
C	-0.463339	-5.476880	-2.394316
H	-1.477692	-5.469402	-2.813051
H	0.243578	-5.418421	-3.229224
H	-0.318411	-6.435837	-1.875776
C	-1.447637	-4.344964	-0.419531
H	-1.472836	-5.293357	0.136211
H	-1.383680	-3.512405	0.291400
H	-2.395051	-4.236200	-0.963862
C	-5.771607	0.672834	-1.457710
H	-6.769788	0.467031	-1.045299
H	-5.692033	1.754544	-1.628363
H	-5.681244	0.170509	-2.426862
C	-4.723327	1.104794	0.757805
H	-3.913376	0.839142	1.451625
H	-4.579518	2.152183	0.458613
H	-5.687411	1.035208	1.279416
C	-5.427940	-1.706457	1.048917
H	-5.329207	-2.789184	1.201743
H	-5.057079	-1.200733	1.948717
H	-6.496691	-1.474767	0.935596
C	-5.065094	-2.150409	-1.372684
H	-4.508378	-1.869763	-2.276493
H	-4.831242	-3.198234	-1.146367
H	-6.140404	-2.074914	-1.582378
H	-1.630928	-0.981920	-1.809473
C	2.857736	0.768523	-2.242660
O	2.073241	1.713097	-2.244252
C	4.337112	0.972495	-1.950857
H	4.660270	0.248527	-1.182192
H	4.936448	0.727222	-2.843217
C	1.029811	-0.795459	-2.624669
H	0.553639	0.132817	-2.978627
H	0.894930	-1.510983	-3.445381
N	2.446844	-0.516762	-2.452123
C	3.386199	-1.616909	-2.555536
H	2.914929	-2.428607	-3.118041
H	4.285099	-1.313631	-3.104335
H	3.681469	-2.018038	-1.576385
C	4.644668	2.386664	-1.490225
H	4.530275	3.086058	-2.331922
H	3.885410	2.697564	-0.757300
C	6.028659	2.513398	-0.875892
H	6.134005	1.779023	-0.056106
H	6.801408	2.241242	-1.615989
C	6.309108	3.907698	-0.336311
H	6.195016	4.642639	-1.149340
H	5.534387	4.165055	0.407086
C	7.688547	4.031741	0.287924
H	8.474239	3.809609	-0.447470
H	7.875191	5.039539	0.679202
H	7.811172	3.322438	1.118638
C	-3.449812	4.834005	-0.667364
F	-4.242449	4.145773	-1.493134
F	-4.166990	5.136445	0.423873
F	-3.145520	5.991934	-1.263314
C	4.331442	-0.796641	1.779697
F	5.535809	-0.211391	1.801349
F	4.100023	-1.269921	3.009767
F	4.402076	-1.845680	0.955256

Structure **L4_IrB3_PC**

C	0.878268	-3.022619	-0.500114
C	2.648657	-1.675963	0.160543
C	3.536410	-2.736726	0.086272
C	3.071401	-3.984011	-0.324193
C	1.725598	-4.127931	-0.605344
C	-0.578767	-3.112400	-0.718249
C	-2.563218	-1.930193	-0.907042
C	-3.302149	-3.102903	-1.006406
C	-2.637000	-4.323479	-0.962142
C	-1.259123	-4.327719	-0.816225
H	2.951179	-0.686998	0.507119
H	3.755577	-4.826911	-0.404618
H	1.336894	-5.093543	-0.917807
H	-3.035551	-0.945512	-0.938248
H	-3.192318	-5.256708	-1.026817
H	-0.725949	-5.272618	-0.752502
N	1.353837	-1.804990	-0.159337
N	-1.238020	-1.939593	-0.762747
Ir	0.000869	-0.108831	-0.242280
O	-0.313400	-1.411875	2.408673
O	-2.838661	3.180425	-1.402266
O	2.110434	1.211099	1.483195
B	-2.750615	1.896188	-1.853905
B	-0.450542	-0.220672	1.688674
B	1.155901	1.378307	0.461777
O	1.173100	2.712959	0.040628
O	-1.010306	0.765496	2.503968
C	-1.047277	0.261907	3.845784
C	-1.068331	-1.276533	3.617422
C	2.885808	2.411123	1.593934
C	1.927436	3.476620	0.989331
C	-3.613193	3.171017	-0.182443
C	-4.361246	1.787068	-0.242452
O	-3.602420	1.037324	-1.213492
C	-0.413176	-2.093280	4.712873
H	0.646720	-1.839185	4.822733
H	-0.916342	-1.932728	5.677484
H	-0.480600	-3.161919	4.470532
C	-2.463143	-1.820687	3.326602
H	-3.097852	-1.848245	4.222911
H	-2.969801	-1.222376	2.556188
H	-2.362354	-2.843633	2.939145
C	0.221323	0.737886	4.542119
H	0.254736	0.430435	5.596137
H	1.116528	0.364404	4.027050
H	0.248566	1.835960	4.507636
C	-2.266695	0.826092	4.547227
H	-3.190490	0.609873	3.997949
H	-2.360645	0.418340	5.564238
H	-2.170552	1.917251	4.626290
C	-2.623229	3.301726	0.964764

H	-2.067028	4.238776	0.829679
H	-3.132463	3.340665	1.938005
H	-1.897268	2.480805	0.987770
C	-4.532691	4.377476	-0.196226
H	-3.929641	5.293833	-0.190118
H	-5.173954	4.401489	-1.084097
H	-5.170924	4.386892	0.698582
C	-4.344100	1.014767	1.062627
H	-4.831550	0.040443	0.917636
H	-3.316874	0.845789	1.412030
H	-4.896331	1.557603	1.843861
C	-5.777454	1.864655	-0.791173
H	-5.801856	2.376491	-1.761985
H	-6.150530	0.843593	-0.941836
H	-6.457542	2.383563	-0.103366
C	0.933357	4.022540	2.007719
H	1.418342	4.652389	2.766031
H	0.190606	4.632788	1.477812
H	0.394498	3.202641	2.505523
C	2.613484	4.619526	0.267160
H	1.856854	5.300363	-0.145270
H	3.250461	5.196850	0.953023
H	3.233451	4.262650	-0.564655
C	4.150510	2.203961	0.769927
H	3.903399	2.040142	-0.287631
H	4.840955	3.055822	0.839965
H	4.672757	1.310015	1.137774
C	3.257374	2.636833	3.046403
H	3.915104	1.827448	3.389720
H	3.796808	3.587389	3.169596
H	2.374034	2.649438	3.694289
H	-1.064895	1.096554	-0.351704
C	0.496020	1.129970	-2.912198
O	0.324669	-0.081090	-2.672400
C	1.890568	1.713555	-2.993635
H	2.046190	2.122813	-4.006633
H	1.955539	2.567583	-2.300556
C	-1.905614	1.448674	-3.113176
H	-2.419487	1.771950	-4.034778
H	-1.842660	0.356052	-3.148091
N	-0.536792	1.964238	-3.164528
C	-0.364042	3.408060	-3.176307
H	-0.136521	3.784004	-2.167089
H	0.432002	3.708175	-3.867827
H	-1.298722	3.868151	-3.509969
C	2.967258	0.699612	-2.657562
H	2.821991	0.381234	-1.612070
H	2.834378	-0.210902	-3.261080
C	4.376302	1.240766	-2.828887
H	4.567978	1.475117	-3.890597
H	4.477119	2.203098	-2.294083
C	5.429819	0.269237	-2.318136
H	5.179855	-0.022521	-1.284488
H	5.382366	-0.660849	-2.907880
C	6.834176	0.844986	-2.350306
H	6.900455	1.737920	-1.711373
H	7.579308	0.123532	-1.992664
H	7.119776	1.146719	-3.367982
C	4.987755	-2.570590	0.410302
F	5.257692	-1.376687	0.941576
F	5.395427	-3.506011	1.275902
F	5.742280	-2.706267	-0.688246
C	-4.781450	-3.035199	-1.224902
F	-5.074778	-2.974826	-2.528226
F	-5.398290	-4.114608	-0.730768
F	-5.320405	-1.956123	-0.647970

Structure **L4_IrB3_IC_TS-isoH**

C	-1.143480	-1.507709	-1.456654
C	-2.426990	0.228437	-0.630701
C	-3.532896	-0.186504	-1.354909
C	-3.435436	-1.320006	-2.151837
C	-2.225707	-1.988380	-2.189924
C	0.168346	-2.167526	-1.481526
C	2.250900	-2.309409	-0.520070
C	2.711790	-3.148037	-1.522500
C	1.840226	-3.520114	-2.542460
C	0.549441	-3.026100	-2.518048
H	-2.471479	1.111853	-0.004724
H	-4.295672	-1.682174	-2.710539
H	-2.129110	-2.907527	-2.760187
H	2.905606	-1.960704	0.275297
H	2.176741	-4.170656	-3.347902
H	-0.140753	-3.266011	-3.322298
N	-1.244823	-0.399333	-0.689896
N	1.007551	-1.833445	-0.492759
Ir	0.339815	0.286098	0.561473
O	1.590795	0.411648	-2.233467
O	3.078553	-0.415805	1.635548
O	-0.937209	2.669003	-0.776577
B	1.868485	0.121433	2.034982
B	1.581241	1.003753	-0.976856
B	-0.067324	2.248253	0.232251
O	0.362027	3.331080	0.983844
O	2.636463	1.894715	-0.850277
C	3.545802	1.638343	-1.933426
C	2.598801	1.063614	-3.024118
C	-1.300030	4.028354	-0.486845
C	-0.062511	4.526950	0.312453
C	3.712215	-0.946979	2.820589
C	3.113599	-0.037425	3.929650
O	1.805236	0.254353	3.403303
C	1.895256	2.141580	-3.836722
H	1.106801	1.671806	-4.437864
H	1.414051	2.878418	-3.178739
H	2.583052	2.664710	-4.514623
C	3.233141	0.044636	-3.949360
H	3.620414	-0.821567	-3.400410
H	2.485505	-0.316381	-4.668195
H	4.060065	0.493524	-4.518544
C	4.250365	2.925615	-2.309888
H	4.906313	2.771816	-3.179053
H	3.540791	3.726249	-2.546327
H	4.872222	3.264510	-1.471486
C	4.562283	0.621682	-1.423856
H	5.342385	0.406615	-2.166709
H	5.037544	1.026357	-0.521205
H	4.077855	-0.322961	-1.138185
C	3.282668	-2.402530	2.959813
H	2.188479	-2.492284	3.022296
H	3.623885	-2.975540	2.088056
H	3.722430	-2.868360	3.851022
C	5.211815	-0.861617	2.632479
H	5.740502	-1.214095	3.529367
H	5.508560	-1.499654	1.788388
H	5.536032	0.160813	2.410809

C	2.958456	-0.695492	5.284659
H	2.537806	0.023963	5.998042
H	2.284402	-1.557754	5.239846
H	3.932886	-1.026786	5.671040
C	3.845616	1.289435	4.068902
H	3.245059	1.959095	4.696271
H	4.828740	1.161526	4.541252
H	3.982804	1.774589	3.093729
C	1.082917	4.974356	-0.585304
H	0.868411	5.933312	-1.076241
H	1.986366	5.086366	0.027301
H	1.302678	4.217634	-1.349325
C	-0.360366	5.593684	1.346140
H	-1.063880	5.236153	2.106060
H	0.566938	5.882784	1.856591
H	-0.780370	6.491906	0.870781
C	-2.575334	3.987578	0.346921
H	-2.958727	4.993250	0.564395
H	-3.350078	3.446191	-0.213573
H	-2.411142	3.465614	1.300459
C	-1.563889	4.751940	-1.791056
H	-2.440811	4.312088	-2.284358
H	-1.771185	5.816843	-1.612808
H	-0.714695	4.670339	-2.478267
H	1.229332	1.286668	1.514699
C	-2.995154	-1.650104	1.618627
O	-2.564798	-2.622351	0.997747
C	-4.491594	-1.455516	1.811327
H	-4.736472	-1.584852	2.879690
H	-4.782293	-0.421962	1.576500
C	-0.720495	-0.904616	2.142253
H	-0.360379	-0.668567	3.150995
H	-0.539541	-1.968014	1.946621
N	-2.168295	-0.709841	2.157576
C	-2.620537	0.450043	2.892618
H	-1.846083	1.225391	2.804581
H	-3.554959	0.860081	2.490949
H	-2.765993	0.235041	3.963666
C	-5.318243	-2.401066	0.958336
H	-4.990947	-2.314742	-0.090812
H	-5.108236	-3.442849	1.240783
C	-6.806779	-2.107930	1.046215
H	-7.155725	-2.235241	2.086107
H	-6.986715	-1.046622	0.797302
C	-7.638504	-2.980164	0.118513
H	-7.297796	-2.819154	-0.918070
H	-7.439492	-4.041347	0.339899
C	-9.127478	-2.697047	0.223032
H	-9.715805	-3.325424	-0.457333
H	-9.492428	-2.878932	1.243666
H	-9.346019	-1.647507	-0.019319
C	-4.785680	0.629754	-1.309158
F	-4.756295	1.616377	-2.212568
F	-5.867024	-0.115159	-1.562600
F	-4.963698	1.209847	-0.112993
C	4.146667	-3.563434	-1.564995
F	4.275303	-4.852538	-1.894177
F	4.822521	-2.858588	-2.485557
F	4.761370	-3.374065	-0.392679

Structure **L4_IrB3_IC-isoH**

C	0.317082	-2.721372	0.258115
C	1.995681	-1.124347	0.405968
C	2.964222	-2.096122	0.587081
C	2.587692	-3.433629	0.599335
C	1.249273	-3.743617	0.430488
C	-1.122154	-2.972684	0.082931
C	-3.224208	-2.009916	-0.103159
C	-3.826684	-3.252130	-0.254401
C	-3.032763	-4.393088	-0.236159
C	-1.665145	-4.249888	-0.069594
H	2.248370	-0.067047	0.355933
H	3.328648	-4.217769	0.735249
H	0.932244	-4.782431	0.439521
H	-3.798895	-1.083598	-0.145520
H	-3.478566	-5.376423	-0.362719
H	-1.025967	-5.128371	-0.080274
N	0.704102	-1.426003	0.251748
N	-1.910170	-1.875532	0.068402
Ir	-0.808069	0.106377	0.106145
O	-1.339642	0.302728	3.229144
O	-3.446707	0.951838	-1.177208
O	1.619828	1.874877	-0.345268
B	-2.141553	1.333001	-0.889825
B	-0.443809	0.256675	2.181327
B	0.355272	1.765714	0.235747
O	0.077704	2.898750	0.989963
O	0.864313	0.254692	2.656461
C	0.814191	0.020104	4.071369
C	-0.592251	0.571360	4.431902
C	2.280016	3.016698	0.230793
C	1.075273	3.885473	0.696333
C	-4.023736	1.957495	-2.032547
C	-3.154460	3.203123	-1.695052
O	-1.888150	2.605664	-1.372225
C	-1.264670	-0.117241	5.600623
H	-2.251919	0.328114	5.776455
H	-1.407194	-1.187452	5.413905
H	-0.669727	0.003448	6.517571
C	-0.604535	2.081563	4.623986
H	-0.098353	2.383786	5.550858
H	-0.132872	2.587937	3.769854
H	-1.646959	2.419609	4.674692
C	0.944995	-1.486943	4.259172
H	0.095530	-2.016127	3.804938
H	1.863008	-1.818886	3.753422
H	1.010190	-1.772824	5.317590
C	1.980549	0.725485	4.731505
H	1.988926	1.798161	4.505015
H	1.949613	0.599903	5.823303
H	2.922421	0.296158	4.363554
C	-3.861706	1.492511	-3.472900
H	-4.381766	2.159536	-4.173462
H	-2.804969	1.440677	-3.767167
H	-4.289940	0.486884	-3.571363
C	-5.495582	2.091862	-1.699439
H	-6.021105	1.165615	-1.966143
H	-5.654294	2.273098	-0.630985
H	-5.951071	2.915664	-2.267363
C	-2.955503	4.181188	-2.834256
H	-2.463549	3.709755	-3.692970
H	-3.916168	4.600377	-3.165844
H	-2.321270	5.011822	-2.497413
C	-3.624554	3.939683	-0.447527
H	-4.552424	4.499832	-0.624738
H	-3.785712	3.246565	0.388824

H	-2.842630	4.646940	-0.143020
C	1.334653	4.711015	1.940544
H	1.615912	4.081688	2.793392
H	2.137347	5.442397	1.767305
H	0.425366	5.262038	2.213435
C	0.514997	4.773285	-0.408255
H	1.193346	5.600756	-0.656917
H	0.301933	4.191766	-1.314810
H	-0.437353	5.197177	-0.062999
C	3.163821	3.657930	-0.820238
H	3.636298	4.570422	-0.428703
H	3.964830	2.961609	-1.105272
H	2.602499	3.919708	-1.724459
C	3.136352	2.500821	1.380136
H	3.713934	3.304473	1.856516
H	2.517077	2.000533	2.136734
H	3.848415	1.762052	0.986062
H	-1.944996	1.038767	0.791782
C	1.659019	-0.769015	-2.736317
O	1.517699	-1.954299	-2.432199
C	3.030261	-0.210223	-3.073423
H	3.027211	0.280629	-4.060367
H	3.243739	0.594171	-2.345474
C	-0.643994	-0.288641	-2.138890
H	-1.450807	0.100952	-2.773251
H	-0.698018	-1.380549	-2.201296
N	0.624638	0.116875	-2.711041
C	0.729828	1.466235	-3.226906
H	-0.100843	2.058576	-2.827205
H	1.657203	1.953053	-2.907068
H	0.675827	1.482247	-4.327706
C	4.121212	-1.263331	-3.004707
H	4.032458	-1.810717	-2.053658
H	3.959221	-2.021577	-3.786215
C	5.515456	-0.670746	-3.121025
H	5.637005	-0.175743	-4.100717
H	5.637637	0.125386	-2.364893
C	6.614478	-1.705227	-2.930571
H	6.496274	-2.168849	-1.937225
H	6.478984	-2.519294	-3.661338
C	8.007382	-1.113630	-3.064861
H	8.153902	-0.671024	-4.060296
H	8.168411	-0.315704	-2.326271
H	8.791615	-1.866656	-2.917023
C	4.373354	-1.661677	0.835840
F	4.701114	-0.591748	0.099947
F	4.551170	-1.320453	2.119328
F	5.248315	-2.632079	0.552384
C	-5.312124	-3.305627	-0.434718
F	-5.697602	-2.565222	-1.478993
F	-5.742880	-4.553975	-0.636285
F	-5.944663	-2.824418	0.639913

Structure **L4_IrB3_IC-isoH_TS-RE**

C	0.402043	-2.601710	0.708394
C	2.036283	-0.957586	0.623009
C	3.026546	-1.862833	0.966660
C	2.683332	-3.189545	1.188965
C	1.354401	-3.556782	1.057974
C	-1.026004	-2.920521	0.545318
C	-3.147388	-2.064313	0.140014
C	-3.713408	-3.327244	0.204748
C	-2.889810	-4.425616	0.446437
C	-1.533594	-4.221898	0.613529
H	2.263344	0.086564	0.414001
H	3.441192	-3.922210	1.455501
H	1.063428	-4.588998	1.229361
H	-3.736277	-1.174468	-0.078776
H	-3.314637	-5.427079	0.492077
H	-0.875079	-5.070007	0.778103
N	0.756674	-1.313934	0.497400
N	-1.837618	-1.869740	0.316965
Ir	-0.790151	0.129402	0.083719
O	0.686495	0.659457	2.709530
O	-1.770273	2.276215	-1.921909
O	1.606793	1.879371	-0.525199
B	-2.025072	1.065409	-1.287040
B	-0.571152	0.616392	2.112320
B	0.318981	1.827329	0.014818
O	-0.015848	3.058987	0.563268
O	-1.556109	0.906351	3.035252
C	-0.911902	1.392772	4.227163
C	0.493088	0.735933	4.129774
C	2.219434	3.109432	-0.099473
C	0.979983	4.011725	0.165805
C	-3.040885	2.822894	-2.306353
C	-3.887665	1.537667	-2.534075
O	-3.320640	0.632582	-1.568858
C	1.628074	1.532618	4.738965
H	2.578274	1.011307	4.559620
H	1.705640	2.532917	4.296757
H	1.495368	1.641009	5.825012
C	0.525508	-0.697180	4.647381
H	0.467208	-0.746645	5.743053
H	-0.301271	-1.285883	4.226102
H	1.469323	-1.160859	4.329270
C	-0.869025	2.910379	4.113731
H	-1.894829	3.281128	3.995982
H	-0.432945	3.378590	5.006762
H	-0.302955	3.220634	3.224556
C	-1.724364	0.979602	5.436828
H	-1.211682	1.266190	6.366628
H	-2.698740	1.483807	5.418522
H	-1.906036	-0.100667	5.455454
C	-3.534359	3.662910	-1.134749
H	-4.452920	4.213205	-1.379155
H	-3.720687	3.044217	-0.247102
H	-2.750357	4.383665	-0.869944
C	-2.852008	3.695155	-3.530281
H	-2.332117	3.159976	-4.333420
H	-3.819828	4.049146	-3.913256
H	-2.251053	4.575790	-3.267968
C	-5.367708	1.690614	-2.247287
H	-5.814226	2.456557	-2.897444
H	-5.884196	0.741287	-2.439863
H	-5.550219	1.967340	-1.203443
C	-3.692160	0.937615	-3.919817
H	-4.098012	-0.081957	-3.925777
H	-4.215262	1.520966	-4.689427
H	-2.630813	0.882464	-4.195914
C	1.163694	5.028056	1.275175
H	1.417426	4.549277	2.228563
H	1.958566	5.744992	1.023106
H	0.231487	5.589686	1.418342
C	0.466276	4.703797	-1.090572
H	1.154436	5.485287	-1.440297

H	0.292978	3.980967	-1.898946
H	-0.498243	5.175316	-0.859387
C	3.150625	3.598248	-1.190402
H	3.601197	4.563205	-0.916690
H	3.965016	2.874098	-1.330559
H	2.632629	3.717395	-2.148887
C	3.017020	2.793734	1.159284
H	3.548484	3.674567	1.543783
H	2.363358	2.391816	1.945625
H	3.767673	2.027363	0.920833
H	-2.007750	1.133036	0.440884
C	1.692473	-1.173233	-2.527949
O	1.480519	-2.291035	-2.056854
C	3.096534	-0.738517	-2.907528
H	3.133244	-0.403382	-3.957242
H	3.344545	0.155751	-2.306379
C	-0.612859	-0.513297	-2.141802
H	-1.349683	-0.237151	-2.906931
H	-0.707090	-1.596142	-2.016727
N	0.707629	-0.243188	-2.676212
C	0.918962	1.006986	-3.380087
H	1.794319	1.543449	-2.996369
H	1.040169	0.844399	-4.462794
H	0.047506	1.649815	-3.216780
C	4.129131	-1.822286	-2.659790
H	4.044825	-2.166254	-1.616908
H	3.895690	-2.706868	-3.271588
C	5.548666	-1.351454	-2.928446
H	5.668436	-1.106318	-3.998480
H	5.734126	-0.409768	-2.382658
C	6.596080	-2.374635	-2.516255
H	6.489097	-2.571790	-1.436827
H	6.388706	-3.332500	-3.020867
C	8.013363	-1.921888	-2.823276
H	8.759988	-2.662259	-2.509722
H	8.147817	-1.748714	-3.900350
H	8.244420	-0.977935	-2.309795
C	4.423298	-1.355306	1.139645
F	4.566218	-0.725951	2.311849
F	5.318782	-2.347405	1.100857
F	4.750016	-0.474514	0.184125
C	-5.180019	-3.548836	0.007822
F	-5.713856	-4.159557	1.073141
F	-5.843081	-2.411049	-0.189316
F	-5.403566	-4.344755	-1.044861

Structure **L4_IrB3_PC-isoH**

C	-1.826223	2.671971	0.426846
C	-2.968790	0.650246	0.435720
C	-4.184251	1.294583	0.599978
C	-4.214499	2.685430	0.673952
C	-3.020768	3.377845	0.585432
C	-0.510145	3.340937	0.401204
C	1.785667	3.046991	0.548205
C	2.010766	4.411933	0.441950
C	0.917820	5.262457	0.293087
C	-0.356184	4.723791	0.274303
H	-2.891281	-0.432007	0.344112
H	-5.160116	3.209600	0.798743
H	-3.016000	4.461936	0.661521
H	2.601160	2.330643	0.647513
H	1.071781	6.335634	0.192746
H	-1.218023	5.372358	0.141041
N	-1.810953	1.321311	0.347775
N	0.556461	2.531115	0.527856
Ir	0.121564	0.346701	0.143467
O	1.047106	-0.814143	2.789374
O	4.431221	-0.267663	-0.631316
O	-1.603237	-1.800806	-1.082860
B	3.458635	-0.614849	-1.525491
B	0.001826	-0.596481	1.898094
B	-0.415794	-1.520691	-0.376851
O	0.245026	-2.721811	-0.107215
O	-1.182209	-1.142288	2.419112
C	-0.928680	-1.546376	3.771685
C	0.610644	-1.768060	3.764200
C	-1.835244	-3.215623	-1.020827
C	-0.393593	-3.767560	-0.846178
C	4.714094	-1.439579	0.167265
C	4.197654	-2.604103	-0.742016
O	3.203138	-1.958105	-1.567438
C	1.310894	-1.483538	5.077704
H	0.954747	-2.158750	5.869412
H	2.390716	-1.642345	4.954987
H	1.160347	-0.448521	5.403924
C	1.009025	-3.148183	3.253259
H	2.096235	-3.163586	3.096032
H	0.754678	-3.943062	3.968029
H	0.533298	-3.359423	2.285703
C	-1.359344	-0.396432	4.673805
H	-0.764694	0.504444	4.473268
H	-2.411577	-0.161411	4.467518
H	-1.265168	-0.649095	5.738739
C	-1.751753	-2.780067	4.085619
H	-2.821149	-2.535851	4.034027
H	-1.556874	-3.590833	3.373671
H	-1.535722	-3.148237	5.099137
C	6.202434	-1.473650	0.453921
H	6.469288	-0.623477	1.094141
H	6.473210	-2.395744	0.987712
H	6.802156	-1.408459	-0.460636
C	3.940849	-1.301535	1.467819
H	4.181359	-0.330865	1.921808
H	2.855666	-1.328299	1.320554
H	4.219674	-2.089230	2.182848
C	5.255910	-3.139781	-1.695923
H	4.770065	-3.801059	-2.425035
H	5.746941	-2.328272	-2.249357
H	6.026209	-3.717417	-1.168224
C	3.546439	-3.744558	0.014088
H	3.301433	-4.560175	-0.680987
H	4.231130	-4.149573	0.773244
H	2.616580	-3.416786	0.494223
C	-0.298126	-5.063795	-0.065889
H	0.753677	-5.367273	0.024593
H	-0.704751	-4.957397	0.947014
H	-0.841743	-5.871959	-0.576784
C	0.354214	-3.898884	-2.168137
H	0.270970	-2.973547	-2.756766
H	1.420645	-4.047321	-1.957226
H	-0.012462	-4.740017	-2.772564
C	-2.548683	-3.661933	-2.281414
H	-2.693719	-4.752124	-2.285639
H	-3.539440	-3.189026	-2.329926
H	-1.997860	-3.383509	-3.187974
C	-2.723601	-3.465927	0.191637

H	-2.239454	-3.109132	1.111613
H	-3.656464	-2.899856	0.064836
H	-2.984895	-4.526892	0.304966
H	1.617062	-0.246243	0.061800
C	0.474676	0.516643	-2.957448
O	0.352143	1.390599	-2.079376
C	-0.742487	-0.004496	-3.694588
H	-0.637192	0.179234	-4.776682
H	-0.782089	-1.098622	-3.566043
C	2.865979	0.448091	-2.534615
H	3.644516	0.723168	-3.267547
H	2.602297	1.367093	-2.001779
N	1.685333	0.008021	-3.274653
C	1.930003	-0.999052	-4.286744
H	2.358637	-1.898365	-3.824904
H	1.011810	-1.274565	-4.808511
H	2.644272	-0.612981	-5.029477
C	-2.035463	0.585125	-3.166266
H	-2.066812	0.398143	-2.085413
H	-2.028896	1.677631	-3.295833
C	-3.264227	-0.038764	-3.811249
H	-3.375289	0.319822	-4.849854
H	-3.118780	-1.129935	-3.876810
C	-4.542313	0.235441	-3.027838
H	-5.389911	-0.266082	-3.518895
H	-4.445736	-0.234914	-2.033252
C	-4.850818	1.716759	-2.869920
H	-5.807152	1.877516	-2.356687
H	-4.073657	2.229980	-2.284410
H	-4.905032	2.214310	-3.849406
C	-5.460284	0.526890	0.750966
F	-5.876670	0.539183	2.022403
F	-6.440911	1.074016	0.018862
F	-5.336667	-0.746990	0.376957
C	3.391332	4.989025	0.440781
F	3.695763	5.492999	-0.761237
F	3.486483	5.997142	1.317304
F	4.316301	4.083864	0.751943

Structure **L4_IrB3_IC_TS-isoS**

C	-1.456645	-0.786135	-1.934538
C	-2.659568	0.513220	-0.441979
C	-3.806086	0.428501	-1.217093
C	-3.756567	-0.289652	-2.407508
C	-2.572682	-0.908334	-2.762630
C	-0.179074	-1.461290	-2.205689
C	1.960022	-1.911009	-1.427490
C	2.236821	-2.707637	-2.523813
C	1.261029	-2.877509	-3.503929
C	0.038690	-2.256236	-3.335719
H	-2.647110	1.058597	0.502019
H	-4.642239	-0.379934	-3.032682
H	-2.527738	-1.501037	-3.671310
H	2.686915	-1.753073	-0.631635
H	1.457564	-3.498609	-4.375697
H	-0.742325	-2.391429	-4.078184
N	-1.510186	-0.059024	-0.800888
N	0.785054	-1.288562	-1.281220
Ir	0.324366	-0.021856	0.466417
O	2.779805	2.077340	0.516373
O	1.717855	-0.505898	3.154544
O	-0.248427	2.933464	1.184340
B	1.830331	-0.484753	1.779309
B	2.003406	1.128573	-0.136474
B	0.130212	2.083919	0.163608
O	0.001304	2.711845	-1.068260
O	2.582754	0.782369	-1.345872
C	3.680130	1.659685	-1.616343
C	4.022514	2.228753	-0.203110
C	-0.371628	4.260173	0.647844
C	-0.636585	3.988562	-0.864176
C	2.950877	-0.986506	3.709966
C	3.570916	-1.778574	2.522582
O	3.035922	-1.074676	1.386156
C	5.070282	1.407177	0.531025
H	6.058550	1.497346	0.060935
H	5.150286	1.776030	1.562811
H	4.781792	0.350286	0.568046
C	4.429857	3.689085	-0.188735
H	4.575712	4.019662	0.847730
H	5.377795	3.828314	-0.728469
H	3.676324	4.338660	-0.648437
C	4.782990	0.844973	-2.266683
H	5.047517	-0.032051	-1.662826
H	4.440292	0.481643	-3.244400
H	5.686343	1.450582	-2.427658
C	3.168647	2.712058	-2.589649
H	3.955263	3.419169	-2.885397
H	2.801310	2.206338	-3.491347
H	2.323566	3.263418	-2.158287
C	3.762549	0.238602	4.104272
H	3.974060	0.864174	3.226828
H	3.169288	0.840107	4.803766
H	4.708768	-0.030311	4.592922
C	2.639469	-1.825977	4.932616
H	2.210789	-1.190538	5.717770
H	1.912788	-2.614472	4.704474
H	3.551487	-2.291826	5.332820
C	5.085006	-1.768180	2.461454
H	5.494534	-0.752994	2.437319
H	5.508595	-2.291972	3.330316
H	5.419277	-2.291717	1.555286
C	3.071736	-3.214485	2.413846
H	3.394473	-3.625066	1.447257
H	3.476247	-3.853723	3.209755
H	1.975305	-3.265264	2.447955
C	-2.111337	3.811683	-1.195888
H	-2.202479	3.369666	-2.197282
H	-2.609699	3.140573	-0.485704
H	-2.650277	4.768409	-1.189189
C	-0.027041	5.001857	-1.812344
H	-0.228661	4.705574	-2.850033
H	-0.464931	5.997332	-1.650855
H	1.059213	5.079744	-1.688470
C	0.948025	4.968603	0.914779
H	1.779402	4.441978	0.431942
H	0.929557	6.013384	0.576511
H	1.139010	4.955175	1.994799
C	-1.493033	4.981133	1.369646
H	-2.433036	4.419520	1.334395
H	-1.222807	5.115887	2.424313
H	-1.662158	5.975920	0.932740
H	-0.141197	0.653982	1.834549
C	-2.946409	-2.130888	1.052029
O	-2.803988	-2.690509	-0.035467
C	-4.334345	-1.856285	1.612214

H	-4.463074	-2.389117	2.569204
H	-4.425035	-0.786538	1.867113
C	-0.529664	-1.874777	1.314084
H	0.064346	-2.242775	2.161097
H	-0.557509	-2.655621	0.546074
N	-1.891995	-1.712103	1.806752
C	-2.051066	-0.994162	3.054575
H	-2.288361	0.071715	2.902190
H	-2.832320	-1.440972	3.680079
H	-1.103891	-1.036209	3.603034
C	-5.444081	-2.248416	0.653806
H	-5.258522	-1.790184	-0.330630
H	-5.409999	-3.333380	0.473935
C	-6.815931	-1.834204	1.160133
H	-7.005375	-2.291777	2.147346
H	-6.829265	-0.742482	1.330060
C	-7.940712	-2.206512	0.206435
H	-7.749433	-1.736150	-0.771651
H	-7.919967	-3.293917	0.027915
C	-9.308011	-1.790741	0.722119
H	-10.111306	-2.061449	0.025496
H	-9.528257	-2.269629	1.686612
H	-9.355083	-0.703959	0.879171
C	-5.051216	1.154368	-0.814339
F	-5.109179	2.365797	-1.381530
F	-6.149393	0.492353	-1.194439
F	-5.115614	1.333617	0.509596
C	3.591710	-3.317648	-2.694614
F	3.505361	-4.570008	-3.157120
F	4.318124	-2.624689	-3.581264
F	4.278511	-3.347530	-1.549994

Structure **L4_IrB3_IC-isoS**

C	1.527046	0.155325	2.015646
C	2.685216	0.586520	0.055964
C	3.835567	0.923012	0.752141
C	3.817301	0.863180	2.141376
C	2.653578	0.466771	2.775920
C	0.245241	-0.267324	2.603102
C	-1.938477	-0.926576	2.174738
C	-2.190945	-1.113444	3.525465
C	-1.177985	-0.860580	4.444534
C	0.055095	-0.442992	3.974631
H	2.647890	0.612148	-1.032883
H	4.706624	1.114228	2.714845
H	2.628206	0.400953	3.859621
H	-2.688045	-1.142686	1.414812
H	-1.349077	-1.005547	5.508533
H	0.866314	-0.264404	4.674611
N	1.562255	0.227257	0.672181
N	-0.756158	-0.499427	1.726680
Ir	-0.308402	-0.188581	-0.396889
O	-2.856216	1.046268	-1.800894
O	-2.831723	-1.880367	-0.769999
O	-0.299511	2.807949	-1.582404
B	-1.724805	-1.313425	-1.396773
B	-2.092483	0.888997	-0.652318
B	-0.251415	1.962521	-0.494125
O	0.247563	2.620813	0.619810
O	-2.775725	1.399406	0.452383
C	-4.129446	1.678092	0.057399
C	-3.988316	1.872389	-1.481912
C	0.460608	3.986865	-1.259261
C	0.407041	4.011239	0.298082
C	-3.342299	-2.935232	-1.606298
C	-2.741751	-2.582605	-3.006536
O	-1.581364	-1.807363	-2.675209
C	-5.190591	1.417150	-2.284708
H	-5.418527	0.357504	-2.119730
H	-6.079220	2.006626	-2.016566
H	-4.998108	1.561087	-3.355857
C	-3.625186	3.295762	-1.881649
H	-3.329013	3.297997	-2.938201
H	-4.468634	3.986535	-1.748763
H	-2.765799	3.659780	-1.306784
C	-4.968846	0.467157	0.444065
H	-6.034763	0.621396	0.228362
H	-4.623679	-0.435333	-0.076720
H	-4.865399	0.297102	1.524772
C	-4.621198	2.897809	0.811197
H	-5.630989	3.179312	0.479440
H	-4.668141	2.673155	1.884363
H	-3.958151	3.759488	0.676429
C	-2.830370	-4.246693	-1.025849
H	-1.734446	-4.299844	-1.040666
H	-3.152155	-4.316366	0.021259
H	-3.227544	-5.115887	-1.566646
C	-4.857385	-2.921770	-1.543268
H	-5.271537	-1.949748	-1.836155
H	-5.282165	-3.691781	-2.203134
H	-5.186056	-3.134746	-0.517195
C	-2.300773	-3.783579	-3.820854
H	-1.525296	-4.361475	-3.303895
H	-3.149979	-4.447794	-4.037089
H	-1.884444	-3.443434	-4.777446
C	-3.638700	-1.687181	-3.847784
H	-3.078680	-1.371223	-4.736581
H	-4.550554	-2.203503	-4.177050
H	-3.908703	-0.780484	-3.293794
C	1.666812	4.522217	0.971308
H	1.872450	5.563433	0.684039
H	1.538137	4.492191	2.061159
H	2.542521	3.912236	0.718714
C	-0.807236	4.738380	0.856037
H	-0.869384	4.543978	1.933936
H	-0.745736	5.823788	0.700886
H	-1.734569	4.365462	0.402297
C	-0.164730	5.186106	-1.943048
H	-0.111452	5.057103	-3.031408
H	-1.218276	5.313880	-1.672057
H	0.376247	6.107149	-1.682079
C	1.864208	3.765052	-1.809343
H	1.790689	3.548325	-2.882019
H	2.504878	4.646726	-1.674309
H	2.357873	2.910133	-1.327262
H	0.150671	-0.006063	-1.919682
C	2.956850	-2.400435	-0.187607
O	2.903109	-2.420701	1.042131
C	4.298100	-2.411619	-0.906630
H	4.394393	-3.334986	-1.502439
H	4.331610	-1.590476	-1.642450
C	0.525016	-2.239468	-0.363068
H	-0.109769	-2.985603	-0.858385
H	0.633838	-2.541006	0.684230
N	1.844160	-2.341466	-0.973258
C	1.902124	-2.281730	-2.420048

H	2.611661	-3.013214	-2.825243
H	0.905142	-2.502765	-2.816898
H	2.178848	-1.281222	-2.790734
C	5.468643	-2.290540	0.052347
H	5.328665	-1.399946	0.686844
H	5.458332	-3.139281	0.752107
C	6.808105	-2.205265	-0.660138
H	6.956469	-3.101440	-1.288244
H	6.802293	-1.348708	-1.356805
C	7.980800	-2.058857	0.297943
H	7.832263	-1.152702	0.907904
H	7.978764	-2.903011	1.006545
C	9.318121	-1.985999	-0.419419
H	9.497541	-2.893964	-1.012256
H	9.347053	-1.133214	-1.112189
H	10.155883	-1.874877	0.280290
C	5.027979	1.427384	0.003425
F	4.925629	2.745670	-0.222532
F	6.161370	1.226655	0.680483
F	5.149417	0.835207	-1.189412
C	-3.563171	-1.523697	3.960924
F	-4.377682	-0.462313	4.042915
F	-4.118363	-2.378742	3.099666
F	-3.545224	-2.101617	5.165710

Structure **L4_IrB3_IC-isoS_TS-RE**

C	2.143863	0.087770	1.443978
C	2.851039	0.428455	-0.725935
C	4.070435	0.941016	-0.297601
C	4.310841	1.041036	1.065853
C	3.338789	0.595548	1.948036
C	1.044116	-0.366529	2.317456
C	-1.203644	-0.895268	2.457296
C	-1.094437	-1.190309	3.810183
C	0.144129	-1.080853	4.425695
C	1.230005	-0.676264	3.662551
H	2.609939	0.347238	-1.787583
H	5.251420	1.446797	1.431784
H	3.502523	0.670949	3.019972
H	-2.153565	-0.973666	1.932741
H	0.259954	-1.319361	5.480120
H	2.218850	-0.622398	4.109696
N	1.910933	0.024929	0.120574
N	-0.167654	-0.473576	1.735117
Ir	-0.331595	0.009242	-0.437194
O	-3.164302	1.121156	-1.209174
O	-2.707771	-1.990059	-0.947761
O	0.813273	2.584760	0.532377
B	-1.481311	-1.581866	-1.512870
B	-2.210886	0.764967	-0.273427
B	-0.116963	2.037580	-0.351901
O	-0.663525	3.025131	-1.149533
O	-2.675823	1.076210	1.012969
C	-4.078736	1.359924	0.919605
C	-4.201386	1.862051	-0.546644
C	0.739635	4.015475	0.422301
C	0.155227	4.196016	-1.005773
C	-3.448837	-2.671857	-1.971352
C	-2.866812	-2.068804	-3.289682
O	-1.541615	-1.694336	-2.902403
C	-5.530856	1.570269	-1.212731
H	-5.760656	0.499050	-1.223101
H	-6.347624	2.092153	-0.693225
H	-5.509416	1.923293	-2.252163
C	-3.875129	3.342034	-0.697919
H	-3.742247	3.565514	-1.764265
H	-4.678761	3.976999	-0.300335
H	-2.931633	3.593161	-0.198808
C	-4.816150	0.050288	1.174798
H	-4.518349	-0.714901	0.445057
H	-4.551537	-0.315739	2.177266
H	-5.907041	0.175079	1.141885
C	-4.447660	2.372269	1.985085
H	-3.840694	3.281652	1.913303
H	-5.508288	2.652354	1.909355
H	-4.282225	1.932698	2.977802
C	-3.175617	-4.165968	-1.821532
H	-3.798620	-4.763762	-2.500270
H	-2.126234	-4.419542	-2.022584
H	-3.404904	-4.463896	-0.790373
C	-4.929354	-2.423191	-1.769081
H	-5.152089	-1.351147	-1.756794
H	-5.516622	-2.891106	-2.572237
H	-5.255234	-2.856023	-0.813608
C	-2.767559	-3.055963	-4.440515
H	-2.132186	-3.915308	-4.196595
H	-3.762409	-3.426710	-4.727025
H	-2.331750	-2.553770	-5.313598
C	-3.586726	-0.815842	-3.766429
H	-4.599037	-1.041195	-4.130771
H	-3.637714	-0.062421	-2.972866
H	-3.013433	-0.384391	-4.597129
C	-0.695847	5.433502	-1.203802
H	-1.094414	5.444677	-2.226436
H	-1.545926	5.464827	-0.513546
H	-0.096620	6.344405	-1.061321
C	1.219142	4.119793	-2.094740
H	1.856457	3.233584	-1.965141
H	0.719584	4.031952	-3.067127
H	1.860307	5.011278	-2.111227
C	2.125905	4.594318	0.624933
H	2.125771	5.681176	0.458255
H	2.456282	4.411972	1.656294
H	2.859362	4.139216	-0.051202
C	-0.197246	4.500546	1.519346
H	-0.270915	5.596035	1.544670
H	-1.203392	4.077983	1.393200
H	0.186134	4.158222	2.488835
H	-0.338681	0.402981	-1.988221
C	2.258282	-2.892163	-0.667110
O	2.184657	-2.937390	0.559270
C	3.574624	-3.187459	-1.378653
H	3.419112	-4.035389	-2.063147
H	3.854842	-2.334515	-2.020408
C	-0.119135	-2.433254	-0.799491
H	-0.696475	-3.356993	-0.979503
H	0.055575	-2.467145	0.277926
N	1.179169	-2.581860	-1.449344
C	1.190127	-2.592234	-2.899141
H	0.558148	-3.406754	-3.286766
H	0.777118	-1.654993	-3.286519
H	2.202526	-2.723543	-3.283630
C	4.717464	-3.496732	-0.423381
H	4.377564	-4.224080	0.327844
H	5.528171	-3.978892	-0.992720
C	5.267667	-2.266206	0.277767

H	5.548204	-1.508594	-0.477992
H	4.468097	-1.818570	0.888753
C	6.474734	-2.557416	1.154694
H	6.207175	-3.323736	1.899833
H	7.274794	-3.000682	0.539575
C	6.993272	-1.312005	1.854868
H	7.884854	-1.515987	2.460704
H	7.256693	-0.532466	1.124440
H	6.226621	-0.891913	2.523425
C	5.066180	1.398681	-1.314398
F	5.137695	0.538013	-2.337057
F	4.730696	2.589815	-1.825170
F	6.290348	1.514561	-0.792080
C	-2.341419	-1.566189	4.547919
F	-2.082446	-1.958918	5.797545
F	-3.183035	-0.525454	4.614669
F	-2.994207	-2.553326	3.930018

Structure **L4_IrB3_PC-isoS**

C	1.744620	0.236419	1.254991
C	2.130767	1.736303	-0.459486
C	3.259249	2.234193	0.169765
C	3.646324	1.691935	1.396013
C	2.876576	0.682118	1.945273
C	0.793818	-0.725477	1.834833
C	-1.430342	-1.345743	1.958948
C	-1.161211	-2.248745	2.979077
C	0.148746	-2.418199	3.411227
C	1.135516	-1.625002	2.843321
H	1.778803	2.141031	-1.407602
H	4.525708	2.074044	1.912751
H	3.126006	0.269642	2.920782
H	-2.443377	-1.168677	1.591252
H	0.387864	-3.143262	4.185402
H	2.169919	-1.718137	3.168433
N	1.393244	0.753654	0.063123
N	-0.472039	-0.632044	1.364525
O	-3.605678	0.119744	0.368154
O	1.231051	-3.135359	-2.925118
O	-2.205834	3.069789	-0.264322
B	1.247991	-1.876510	-2.284864
B	-2.759977	0.300134	-0.744813
B	-1.210717	2.185589	0.143224
O	-0.456460	2.765683	1.176672
O	-3.534467	0.367715	-1.897762
C	-4.911702	0.480686	-1.524266
C	-4.929094	-0.160235	-0.106092
C	-2.268110	4.136272	0.688416
C	-0.822209	4.147976	1.259857
C	0.269989	-3.940972	-2.255197
C	-0.783203	-2.888261	-1.808993
O	0.061182	-1.768309	-1.470011
C	-5.935043	0.435949	0.858356
H	-5.871331	-0.079588	1.825998
H	-5.742865	1.500765	1.030485
H	-6.960839	0.320089	0.479201
C	-5.089103	-1.676180	-0.139444
H	-4.843196	-2.084165	0.852158
H	-6.116327	-1.976855	-0.386839
H	-4.410562	-2.133131	-0.872839
C	-5.252792	1.965328	-1.512383
H	-4.619895	2.503474	-0.795085
H	-5.041390	2.378384	-2.506633
H	-6.311698	2.143878	-1.279120
C	-5.763310	-0.235578	-2.554884
H	-6.821216	-0.245821	-2.253939
H	-5.689816	0.285430	-3.517890
H	-5.431209	-1.268968	-2.708835
C	-0.251428	-4.983538	-3.225873
H	-1.080739	-5.556390	-2.786921
H	-0.595718	-4.523601	-4.158422
H	0.552546	-5.688720	-3.474481
C	0.941725	-4.635739	-1.071816
H	1.251116	-3.916256	-0.302362
H	0.282644	-5.383007	-0.608590
H	1.840732	-5.148519	-1.437533
C	-1.704716	-2.459364	-2.942860
H	-2.426177	-3.249089	-3.196820
H	-2.263085	-1.561108	-2.647253
H	-1.127027	-2.213065	-3.844248
C	-1.594043	-3.301563	-0.599795
H	-2.295621	-2.502258	-0.328067
H	-2.182126	-4.205080	-0.817427
H	-0.954702	-3.505012	0.267928
C	0.157991	4.921750	0.384344
H	0.077009	4.610931	-0.666521
H	0.000537	6.007394	0.441297
H	1.180965	4.707552	0.725541
C	-0.706595	4.611454	2.698013
H	-1.071485	5.642915	2.809179
H	-1.274364	3.963865	3.375089
H	0.345254	4.589512	3.012525
C	-3.313153	3.750015	1.728983
H	-4.274609	3.594286	1.221451
H	-3.045103	2.809397	2.228505
H	-3.452848	4.532602	2.487170
C	-2.688624	5.408353	-0.019805
H	-2.036128	5.631605	-0.870957
H	-3.710932	5.294669	-0.403950
H	-2.677060	6.265207	0.669673
H	-0.868303	1.047637	-2.027601
C	3.387826	-1.004914	-1.657609
O	2.537536	-1.843808	-1.240623
C	4.709544	-0.864534	-0.967267
H	5.509923	-1.098296	-1.688056
H	4.858853	0.194435	-0.700810
C	1.713110	-0.630678	-3.218776
H	1.046765	0.242671	-3.146465
H	1.804610	-0.921668	-4.274856
N	3.044024	-0.272560	-2.710592
C	3.855054	0.729270	-3.362196
H	3.244758	1.624319	-3.540816
H	4.712514	1.017626	-2.745685
H	4.216043	0.363148	-4.333039
C	4.835135	-1.735989	0.272743
H	3.947494	-1.588035	0.907730
H	4.815781	-2.797705	-0.014258
C	6.089988	-1.422242	1.069281
H	6.986342	-1.602217	0.451008
H	6.109476	-0.344404	1.314340
C	6.186394	-2.229410	2.354807
H	5.292003	-2.029337	2.969930
H	6.147680	-3.303605	2.113438
C	7.442628	-1.919508	3.150609
H	8.344740	-2.142738	2.564589
H	7.485848	-0.855875	3.423899

H	7.495743	-2.503677	4.077192
C	4.101742	3.288707	-0.467987
F	5.296065	2.777521	-0.832174
F	3.538706	3.796366	-1.564613
F	4.355511	4.292554	0.373576
C	-2.294364	-3.079525	3.494681
F	-2.641719	-4.015540	2.602232
F	-1.976019	-3.705010	4.631975
F	-3.381026	-2.336761	3.721598
Ir	-0.755189	0.380189	-0.563536

Structure **L4_IrB2H**

C	-2.993117	1.131834	-0.679011
C	-4.016647	1.992468	-0.298384
C	-3.696270	3.241101	0.216712
C	-2.356332	3.577997	0.349550
C	0.592376	4.154582	0.430536
C	1.957528	4.294142	0.639702
C	2.754952	3.160038	0.586182
C	2.169933	1.931626	0.305815
H	2.746193	1.002969	0.278532
C	0.061281	2.895948	0.160822
C	-1.381877	2.657812	-0.031675
C	3.780737	-1.846030	-0.098317
C	3.075534	-3.005142	-0.857918
C	4.677439	-0.998656	-0.994620
H	4.146620	-0.688829	-1.905138
H	5.592613	-1.530405	-1.287731
H	4.974643	-0.091838	-0.448733
C	4.539881	-2.256142	1.147185
H	4.985284	-1.368815	1.616897
H	5.353228	-2.953762	0.900001
H	3.879922	-2.730584	1.881220
C	3.891076	-3.615712	-1.980618
H	3.316875	-4.419483	-2.458594
H	4.826132	-4.048701	-1.595820
H	4.136938	-2.876937	-2.751376
C	2.571110	-4.100963	0.072651
H	1.962497	-3.677086	0.881826
H	3.394606	-4.688400	0.501659
H	1.930687	-4.778267	-0.507688
B	1.628324	-1.243944	-0.637212
N	-1.706006	1.452008	-0.545755
N	0.857254	1.799382	0.084963
O	2.670399	-1.021892	0.280133
O	1.924235	-2.356393	-1.412020
Ir	0.020322	-0.023831	-0.752218
B	-0.948110	-1.541319	0.099321
O	-2.340979	-1.650456	0.062460
O	-0.405499	-2.552326	0.876403
C	-2.724504	-2.592779	1.073409
C	-1.455886	-3.487668	1.169896
C	-2.998822	-1.798503	2.344659
C	-3.985421	-3.304291	0.627203
C	-1.204792	-4.098104	2.533105
C	-1.407992	-4.565330	0.095265
H	-2.092189	-1.288924	2.697000
H	-3.382397	-2.433393	3.154653
H	-3.754440	-1.033427	2.120427
H	-4.813088	-2.584640	0.570190
H	-4.266753	-4.090211	1.342782
H	-3.864751	-3.757442	-0.362652
H	-0.287498	-4.700808	2.503667
H	-2.034854	-4.757350	2.825658
H	-1.076916	-3.329830	3.303496
H	-1.589347	-4.140007	-0.900518
H	-2.140099	-5.363283	0.279547
H	-0.405155	-5.010684	0.089660
H	-3.200310	0.134609	-1.064085
H	-0.577029	-1.177166	-1.712265
H	-4.479390	3.930087	0.522982
H	-2.078797	4.535795	0.782321
H	-0.052571	5.029001	0.457665
H	2.394308	5.268929	0.842909
C	-5.428104	1.508490	-0.427794
F	-5.699844	1.139438	-1.681455
F	-5.636301	0.439364	0.349935
F	-6.309909	2.447364	-0.074218
C	4.240813	3.200819	0.777511
F	4.634036	2.307869	1.688607
F	4.875513	2.903529	-0.361303
F	4.655148	4.407302	1.172913

Cartesian coordinates of the lowest energy structures for BDE calculations with SCS-MP2/aug-cc-pVTZ//M06-2X/6-311+G(2d,p)

Structure **iPrNMe2**

```
 C    -0.023101  -0.515136  -0.009346
 O     0.087813  -1.664501   0.378425
 N    -1.251650   0.065899  -0.172630
 C     1.219094   0.324046  -0.301424
 H     1.040241   0.954674  -1.174601
 C    -2.435258  -0.689044   0.195902
 H    -2.174340  -1.738763   0.282601
 H    -2.837136  -0.339488   1.152207
 H    -3.202046  -0.559235  -0.570536
 C    -1.497715   1.463927  -0.456479
 H    -2.257876   1.549351  -1.236700
 H    -1.868496   1.981588   0.435056
 H    -0.602226   1.970382  -0.798884
 C     1.515847   1.210218   0.914860
 H     2.407807   1.812627   0.735878
 H     0.690934   1.884054   1.152153
 H     1.699751   0.581389   1.788233
 C     2.400336  -0.590836  -0.602980
 H     2.605182  -1.236510   0.250090
 H     2.194585  -1.232025  -1.460044
 H     3.287452   0.007611  -0.817648
```

Structure **iPrNMe2_N·**

```
 C     0.068046  -0.467095  -0.011721
 O     0.057982  -1.627047   0.359380
 N     1.275997   0.195164  -0.144061
 C    -1.208332   0.304900  -0.297172
 H    -1.050567   0.941927  -1.170914
 C     2.460582  -0.592016   0.189359
 H     2.470884  -1.507636  -0.398431
 H     3.344712   0.001588  -0.025536
 H     2.443453  -0.866771   1.243517
 C     1.431071   1.512559  -0.497134
 H     2.432203   1.878644  -0.641233
 H     0.575783   2.103681  -0.765360
 C    -2.344077  -0.666386  -0.596796
 H    -3.260550  -0.111959  -0.804721
 H    -2.111770  -1.293897  -1.457238
 H    -2.513920  -1.323276   0.255709
 C    -1.543448   1.182242   0.917250
 H    -1.730670   0.547038   1.785280
 H    -0.734097   1.869793   1.167010
 H    -2.444338   1.765865   0.722586
```

Structure **iPrNMe2_C·**

```
 C     0.004996  -0.522466  -0.031482
 O     0.126496  -1.684831   0.306539
 N    -1.222462   0.057898  -0.194137
 C     1.240458   0.353228  -0.283495
 H     1.101247   0.900240  -1.218905
 C    -2.401840  -0.706757   0.170281
 H    -2.148546  -1.761818   0.190694
 H    -2.769838  -0.409213   1.157625
 H    -3.190135  -0.529142  -0.563091
 C    -1.446721   1.479712  -0.364301
 H    -2.334036   1.622791  -0.983563
 H    -1.611470   1.976746   0.597753
 H    -0.609654   1.961665  -0.859289
 C     1.401130   1.322580   0.842333
 H     1.730667   2.336618   0.667293
 H     1.383925   0.955595   1.860177
 C     2.477718  -0.541490  -0.427034
 H     2.636114  -1.111197   0.487102
 H     2.345874  -1.251246  -1.244134
 H     3.356667   0.073486  -0.622824
```

Structure **iPrNEt2**

```
 C    -0.415968  -0.482495  -0.327828
 O    -0.501301  -1.593624  -0.825571
 N     0.796080   0.100727  -0.110005
 C    -1.671487   0.268725   0.106815
 H    -1.495650   1.345454   0.071298
 C     1.988146  -0.646074  -0.501094
 H     1.764552  -1.179598  -1.423350
 H     2.777958   0.078842  -0.710149
 C     1.005139   1.382866   0.547330
 H     1.918734   1.297286   1.141540
 H     0.201764   1.572823   1.256828
 C    -2.824027  -0.069493  -0.832958
 H    -3.731117   0.445778  -0.512339
 H    -2.599217   0.227861  -1.857920
 H    -3.004791  -1.143700  -0.830286
 C    -2.003789  -0.144257   1.545858
 H    -2.184579  -1.220063   1.582214
 H    -1.191649   0.083911   2.239151
 H    -2.903563   0.367624   1.890744
 C     2.423899  -1.628074   0.578707
 H     1.630487  -2.355245   0.750352
 H     3.325560  -2.161251   0.273729
 H     2.634878  -1.109738   1.516487
 C     1.131880   2.537629  -0.439952
 H     1.941481   2.351716  -1.147326
 H     0.209097   2.656703  -1.009866
 H     1.341144   3.472554   0.082227
```

Structure **iPrNEt2_N·**

```
 C    -0.618612  -0.471295  -0.282320
 O    -0.830293  -1.670131  -0.340132
 N     0.677867   0.014721  -0.337651
 C    -1.757186   0.520795  -0.104511
 H    -1.530382   1.437031  -0.653313
 C     1.735954  -0.996221  -0.440926
 H     1.338434  -1.818781  -1.029036
 H     2.570976  -0.558985  -0.985133
 C     1.033917   1.331093  -0.084933
 H     0.230541   2.033507   0.047021
 C    -3.049314  -0.071264  -0.656518
 H    -3.866976   0.639698  -0.526754
 H    -2.955818  -0.305785  -1.717083
 H    -3.295976  -0.994110  -0.132908
 C    -1.900227   0.840631   1.390223
 H    -2.162514  -0.070120   1.932242
 H    -0.978424   1.236955   1.819076
```

H	-2.695935	1.570716	1.545699
C	2.168028	-1.487205	0.933839
H	1.322892	-1.954533	1.439530
H	2.967473	-2.223514	0.842348
H	2.527559	-0.658478	1.546851
C	2.405873	1.831445	-0.372052
H	3.178988	1.297940	0.188731
H	2.670182	1.756080	-1.435425
H	2.465655	2.882513	-0.094039

Structure **iPrOMe**

C	-0.294618	-0.295563	-0.012489
O	-0.513858	-1.304473	0.601252
C	1.073777	0.216541	-0.403080
H	1.000649	0.552372	-1.440932
C	-2.589071	0.151601	-0.042475
H	-2.674517	0.088255	1.041460
H	-3.246838	0.921343	-0.435101
H	-2.831846	-0.818633	-0.473588
C	1.418403	1.427557	0.472897
H	2.393187	1.825281	0.188304
H	0.676322	2.218159	0.366641
H	1.466794	1.132208	1.523210
C	2.113940	-0.886853	-0.274776
H	2.173188	-1.231896	0.757937
H	1.861883	-1.745712	-0.896512
H	3.093617	-0.513114	-0.575258
O	-1.267019	0.540978	-0.413329

Structure **iPrOMe_C·**

C	-0.334548	-0.322674	-0.004917
O	-0.539819	-1.347676	0.576828
C	1.012726	0.231589	-0.397082
H	0.919915	0.588086	-1.426639
C	-2.610555	0.196673	-0.030794
H	-2.792127	-0.786811	0.368801
H	-3.354702	0.833991	-0.474941
C	1.337718	1.431925	0.502149
H	2.298521	1.858834	0.212814
H	0.575746	2.206610	0.423150
H	1.406882	1.114679	1.544654
C	2.079119	-0.850254	-0.303998
H	2.159467	-1.214973	0.720535
H	1.839509	-1.701160	-0.941351
H	3.045753	-0.446862	-0.607595
O	-1.335897	0.525682	-0.378275

Structure **iPrCO**

C	0.742901	-0.262239	-0.048730
O	0.858117	-1.217486	-0.774951
C	-0.621837	0.233418	0.407861
H	-0.503951	0.660353	1.409383
C	-1.062643	1.353829	-0.545745
H	-2.022733	1.760891	-0.226064
H	-0.343421	2.173656	-0.580571
H	-1.178837	0.956382	-1.555890
C	-1.642218	-0.896463	0.433594
H	-1.741901	-1.332400	-0.560576
H	-1.337123	-1.691593	1.114705
H	-2.615957	-0.520988	0.751749
C	1.945641	0.511837	0.438257
H	1.772439	1.587398	0.378110
H	2.108591	0.269845	1.491918
H	2.826890	0.234052	-0.134580

Structure **iPrCO_C·**

C	0.697090	-0.300147	-0.027384
O	0.747978	-1.339716	-0.631164
C	-0.648066	0.324956	0.383738
H	-0.515818	0.735300	1.390254
C	-0.924535	1.443790	-0.564126
H	-0.637146	2.461695	-0.345430
H	-1.296534	1.212588	-1.553343
C	-1.756268	-0.723208	0.369688
H	-1.870664	-1.129939	-0.635023
H	-1.517528	-1.552121	1.036507
H	-2.701151	-0.276962	0.679692
C	1.938870	0.469944	0.352766
H	1.823357	1.528423	0.115287
H	2.084962	0.389555	1.433146
H	2.804154	0.057172	-0.159871
